# Supplementary material for: Dinickel-catalyzed enantioselective reductive addition of imines with vinyl halides
Source: Nat Commun. 2025 Oct 6;16:8871. doi: 10.1038/s41467-025-63940-y (PMC12501250; doi:10.1038/s41467-025-63940-y)
Supplement: Supplementary file 1 — Supplementary Information [file 41467_2025_63940_MOESM1_ESM.pdf]

Supplementary Information for

Dinickel-Catalyzed Enantioselective Reductive Addition of Imines with Vinyl Halides

Peng Zhou[1], Peigen Wang[1], Hongdan Zhu[2], Jian Zhang[1], Qian Peng[2,3*], Zhonglin Tao[1,4*]

[1] State Key Laboratory of Chemo and Biosensing, College of Chemistry and Chemical Engineering, Hunan University, 410082, Changsha, China.

[2] State Key Laboratory of Elemento-Organic Chemistry, Frontiers Science Center for New Organic Matter, College of Chemistry, Nankai University, 300071, Tianjin, China.

[3] Tianjin Key Laboratory of Biosensing and Molecular Recognition, Tianjin, China.

[4] Greater Bay Area Institute for Innovation, Hunan University, 511300, Guangzhou, China.

*e-mail: qpeng@nankai.edu.cn; taozl@hnu.edu.cn

Table of Contents

1. General Information....2
2. Optimization of reaction conditions....3
3. Reactions with poor results....8
4. Synthesis of Substrates....9
5. Synthesis of ligands....10
6. General procedure for synthesis of chiral product....14
7. Characterization of Products....15
8. Transformations of Chiral Products....45
9. Mechanistic study....48
10. Determination of the Absolute Configuration....51
11. Density functional theory calculations....52
12. NMR Spectra....57
13. HPLC Spectra....154
14. Supplementary Reference....236

1. General Information

Unless otherwise noted, all reactions were conducted in oven-dried Schlenk tube with a magnetic stirrer under nitrogen atmosphere. Starting materials were purchased from commercial suppliers (Energy Chemical, Adamas, Bide Pharmatech Co.,Ltd., etc.) and used as supplied unless otherwise stated. Ni(cod)$_2$ and NiBr$_2$·glyme were purchased from Bide Pharmatech Co.,Ltd. Zn powder (600 mesh) was purchased form Aladdin and used as received. Solvents were purified under nitrogen using a solvent purification system. Analytical thin layer chromatography (TLC) was performed using silica gel plates. Visualization was accomplished by ultraviolet fluorescence, and/or phosphomolybdic acid, and/or $KMnO_4$. Flash column chromatography was performed using EM Science (200-300 mesh) silica gel.

^{1}H Nuclear Magnetic Resonance (^{1}H NMR), ^{13}C Nuclear Magnetic Resonance (^{13}C NMR), and ^{19}F Nuclear Magnetic Resonance (^{19}F NMR) spectra were recorded on Bruker 400 MHz at 20 °C with $CDCl_3$ or DMSO-d_6 as solvent. All ^{1}H NMR spectra are reported in parts per million (ppm) downfield of TMS and were measured relative to the signals for tetramethylsilane (δ = 0 ppm). All ^{13}C NMR spectra were reported in ppm relative to $CDCl_3$ (77.16 ppm) or DMSO-d_6 (39.52 ppm). ^{19}F NMR spectra were recorded on Bruker AVANCE NEO instrument (376 MHz) and referenced relative to $CFCl_3$ (δ 0.0 ppm). The data are reported as follows: chemical shift (ppm), multiplicity (s = singlet, d = doublet, t = triplet, q = quartet, m = multiplet, br = broad), coupling constant *J* (Hz), and integration. High resolution mass spectra were recorded on a Thermo Scientific Orbitrap Exploris 120. Chiral HPLC (High Pressure Liquid Chromatography) was performed on an SHIMADZU HPLC apparatus with columns (Chiralpak AD-H, IC-H, IBN5, Chiralcel OD-H, OJ-H, Chirobiotic ® T Astec, 250 mm × 4.6 mm). Optical rotations were recorded on an Anton Paar MCP-4100 polarimeter.

2. Optimization of reaction conditions

2.1. Optimization of reaction conditions for the imines

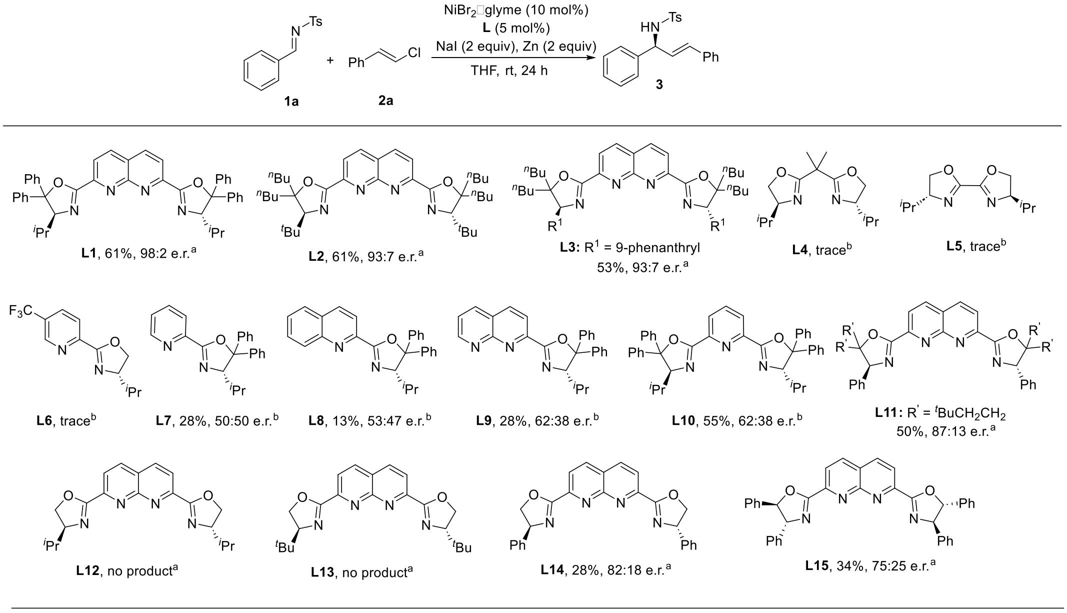

Supplementary Figure S1. Evaluation of different ligands for the reductive addition reaction of aromatic imines. [a]Reactions were carried out with imine **1a** (0.1 mmol, 1.0 equiv), vinyl chloride **2a** (1.2 equiv), $NiBr_2$•glyme (10 mol %), **L** (5 mol %), NaI (2.0 equiv), Zn (2.0 equiv) in THF (1.0 mL) under nitrogen at 25 °C for 24 h; [b]Imine **1a** (0.2 mmol, 1.0 equiv), vinyl chloride **2a** (1.5 equiv), $NiBr_2$•glyme (10 mol %), **L** (10 mol %), NaI (2.0 equiv), Zn (2.0 equiv) in THF (1.0 mL) under nitrogen at 25 °C for 48 h Isolated yields.

Supplementary Table S1. Screening of Ni-sources for the reductive addition reaction of aromatic imines[a]

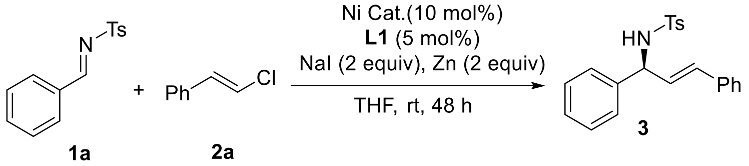

Entry	Ni Cat.	Yield (%)[b]	e.r. (%)[c]
1	$Ni(acac)_2$	9	93:7
2	$Ni(OTf)_2$	No reaction	--
3	$Ni(OAc)_2$	No reaction	--
4	$Ni(ClO_4)_2$•$6H_2O$	Trace	--
5	$NiBr_2$•glyme	91	98:2
6	$NiCl_2$•glyme	74	98:2

[a]Reactions were carried out with imine **1a** (0.2 mmol, 1.0 equiv), vinyl chloride **2a** (1.5 equiv), Ni Cat. (10 mol %), **L1** (5 mol %), NaI (2.0 equiv), Zn (2.0 equiv) in THF (1.0 mL) under nitrogen at 25 °C for 48 h; [b]Isolated yields; [c]Determined by HPLC on a chiral stationary phase.

Supplementary Table S2. Optimization of NaI loading for the reductive addition reaction of aromatic imines[a]

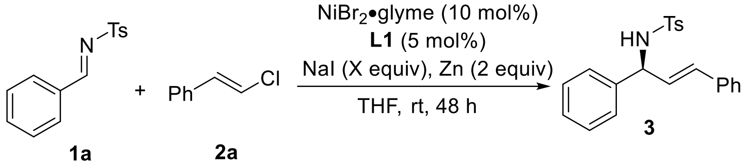

Entry	NaI (X eq)	Yield (%)[b]	e.r. (%)[c]
1	0	54	98:2
2	0.5	56	98:2
3	1	77	98:2
4	2	91	98:2

[a]Reactions were carried out with imine **1a** (0.2 mmol, 1.0 equiv), vinyl chloride **2a** (1.5 equiv), $NiBr_2$•glyme (10 mol %), **L1** (5 mol %), NaI (X equiv), Zn (2.0 equiv) in THF (1.0 mL) under nitrogen at 25 °C for 48 h; [b]Isolated yields; [c]Determined by HPLC on a chiral stationary phase.

Supplementary Table S3. Screening of solvents for the reductive addition reaction of aromatic imines[a]

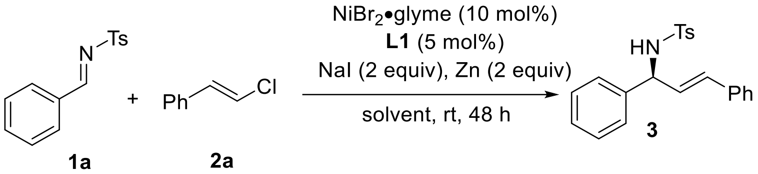

Entry	Solvent	Yield (%)[b]	e.r. (%)[c]
1	DMF	39	83:17
2	DMA	36	77:23
3	MeCN	48	96:4
4	1,4-dioxane	21	95:5
5	2-MeTHF	71	92:8
6	THF	91	98:2

[a]Reactions were carried out with imine **1a** (0.2 mmol, 1.0 equiv), vinyl chloride **2a** (1.5 equiv), $NiBr_2$•glyme (10 mol %), **L1** (5 mol %), NaI (2.0 equiv), Zn (2.0 equiv) in Solvent (1.0 mL) under nitrogen at 25 °C for 48 h; [b]Isolated yields; [c]Determined by HPLC on a chiral stationary phase.

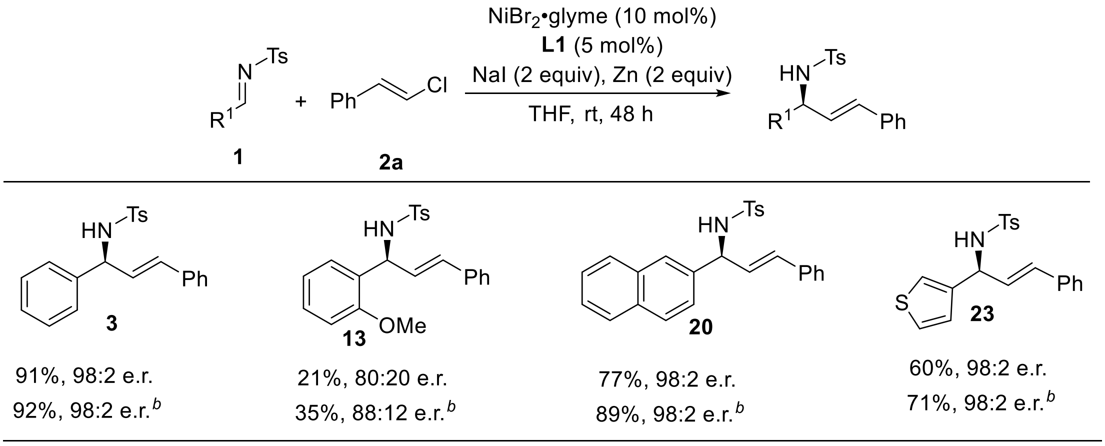

Supplementary Figure S2. Screening of $ZnBr_2$ for the reductive addition reaction of aromatic imines. Unless noted, reactions were carried out with imine **1** (0.2 mmol, 1.0 equiv), vinyl chloride **2a** (1.5 equiv), $NiBr_2$•glyme (10 mol %), **L1** (5 mol %), NaI (2.0 equiv), Zn (2.0 equiv) in THF (1.0 mL) under nitrogen at 25 °C for 48 h; Isolated yields; Determined by HPLC on a chiral stationary phase. [b]$ZnBr_2$ (20 mol%) was added.

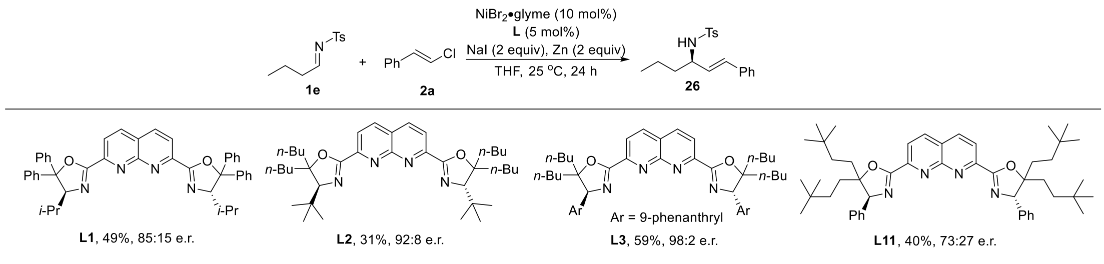

Supplementary Figure S3. Evaluation of different ligands for the reductive addition reaction of aliphatic imines. Reactions were carried out with imine **1e** (0.1 mmol), **2a** (0.15 mmol), $NiBr_2$•glyme (10 mol%), **L** (5 mol%), Zn (0.2 mmol), NaI (0.2 mmol), THF (1 ml), 25 °C, 24 h; Isolated yields.

Supplementary Table S4. Screening of additives for the reductive addition reaction of aliphatic imines [a]

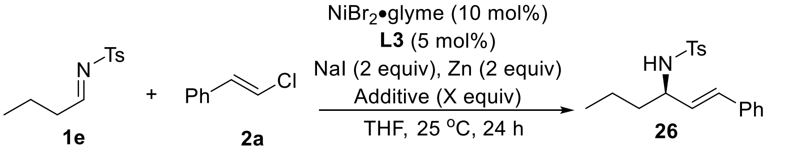

Entry	Additive	Yield (%)[b]	e.r. (%)[c]
1	$MgBr_2$•Et_2O (1.0 equiv)	31	63:37
2	$ZnBr_2$ (1.0 equiv)	76	98:2
3	$ZnBr_2$ (0.2 equiv)	53	98:2

[a]Reactions were carried out with imine **1e** (0.2 mmol, 1.0 equiv), vinyl chloride **2a** (1.5 equiv), $NiBr_2$•glyme (10 mol %), **L3** (5 mol %), NaI (2.0 equiv), Zn (2.0 equiv), additive (X equiv) in THF (2.0 mL) under nitrogen at 25 °C for 24 h; [b]Isolated yields; [c]Determined by HPLC on a chiral stationary phase.

2.2. Optimization of reaction conditions for the aldehydes

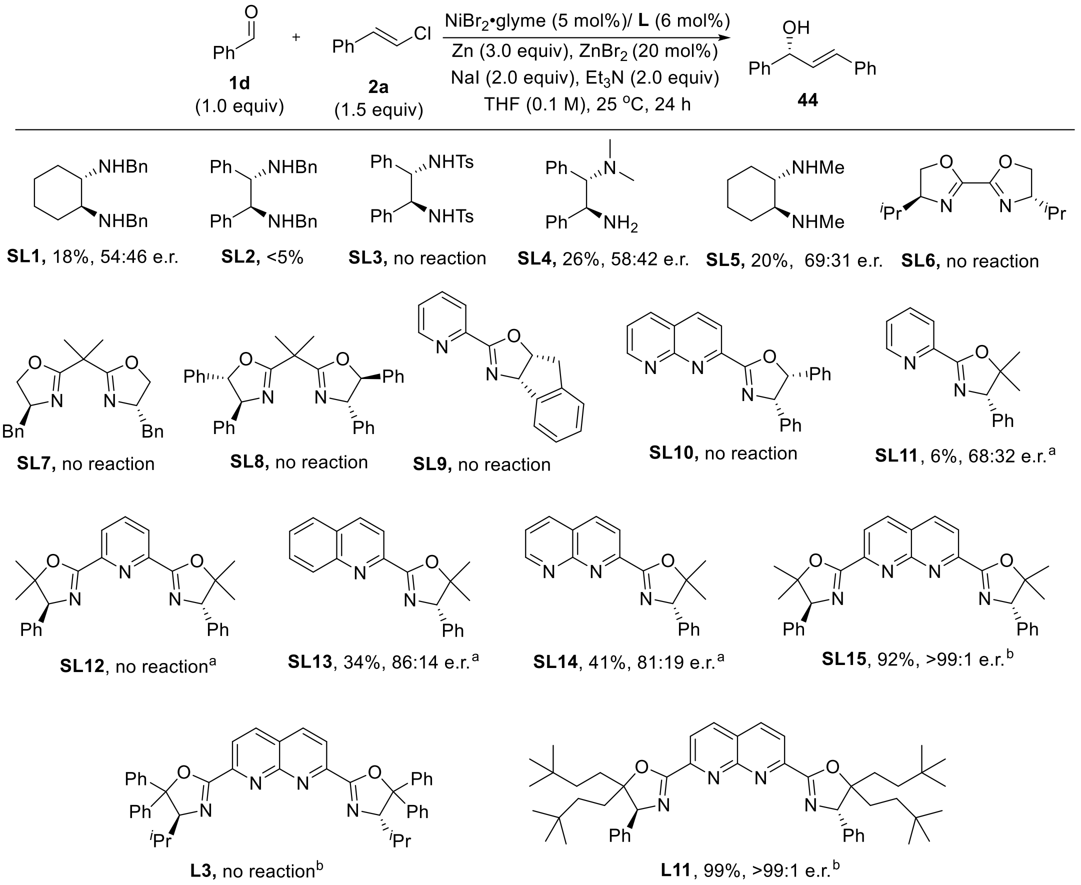

Supplementary Figure S4. Evaluation of different ligands for the reductive addition reaction of aldehydes. Standard conditions: **1d** (0.1 mmol), **2a** (0.15 mmol), $NiBr_2$•glyme (5 mol%), **L** (6 mol%), $ZnBr_2$ (20 mol%), Zn (0.3 mmol), NaI (2.0 equiv), Et_3N (0.2 mmol), THF (1 ml), 25 °C, 24 h; [a]**1d** (0.2 mmol), **2a** (0.3 mmol), $NiBr_2$•glyme (5 mol%), **L** (6 mol%), $ZnBr_2$ (20 mol%), Zn (0.6 mmol), Et_3N (0.4 mmol), 2-Me-THF (1 ml), 25 °C, 16 h; [b]**1d** (0.2 mmol), **2a** (0.3 mmol), $NiBr_2$•glyme (5 mol%), **L** (2.5 mol%), $ZnBr_2$ (20 mol%), Zn (0.6 mmol), Et_3N (0.4 mmol), 2-Me-THF (1 ml), 25 °C, 16 h.

Supplementary Table S5. Screening Ni-sources and Reductants for the reductive addition reaction of aldehydes[a]

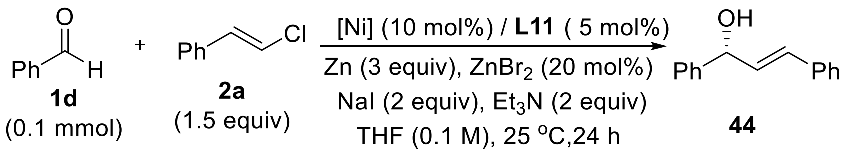

Entry	Ni Cat.	Yield (%)[b]	e.r.[c]
1	$Ni(cod)_2$	46	98:2
2	$Ni(acac)_2$	No reaction	--
3	$Ni(OAc)_2$	No reaction	--
4	$Ni(OTf)_2$	Trace	--
5	$NiCl_2$	No reaction	--

6	$NiBr_2$	No reaction	--
7	NiI_2	20	97:3
8	$NiCl_2$•glyme	50	>99:1
9	$NiBr_2$•glyme	61	>99:1
10[d]	$NiBr_2$•glyme	No reaction	--

[a]Reactions were carried out with aldehyde **1d** (0.1 mmol, 1.0 equiv), vinyl chloride **2a** (1.5 equiv), Ni Cat. (10 mol %), **L11** (5 mol %), NaI (2.0 equiv), $ZnBr_2$ (20 mol%), Et_3N (2.0 equiv), Zn (3.0 equiv) in THF (1.0 mL) under nitrogen at 25 °C for 24 h; [b]Isolated yields; [c]Determined by HPLC on a chiral stationary phase; [d]Mn was used instead of Zn.

Supplementary Table S6. Screening base for the reductive addition reaction of aldehydes[a]

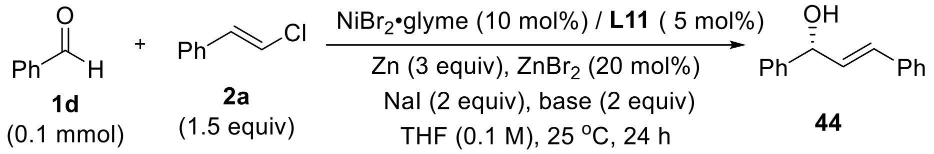

Entry	Bases	Yield (%)[b]	e.r.[c]
1	--	15	97:3
**2**	Et_3N	61	>99:1
3	2,6-Lutidine	34	93:7
4	Pyridine	No reaction	--
5	DABCO	No reaction	--
6	DBU	No reaction	--
7	$NaHCO_3$	Trace	--
8	DIPEA	Trace	--

[a]Reactions were carried out with aldehyde **1d** (0.1 mmol, 1.0 equiv), vinyl chloride **2a** (1.5 equiv), $NiBr_2$•glyme (5 mol%), **L11** (5 mol %), NaI (2.0 equiv), $ZnBr_2$ (20 mol%), Zn (3.0 equiv), base (2.0 equiv) in THF (1.0 mL) under nitrogen at 25 °C for 24 h; [b]Isolated yields; [c]Determined by HPLC on a chiral stationary phase.

Supplementary Table S7. Screening Solvents for the reductive addition reaction of aldehydes[a]

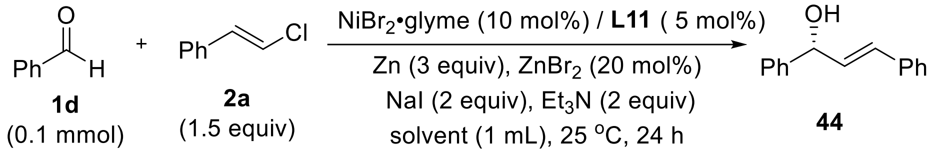

Entry	Solvent	Yield (%)[b]	e.r.[c]
1	THF	61	>99:1
2	DMF	No reaction	--
3	MeCN	No reaction	--
4	DMA	No reaction	--
5	DCE	Trace	--
6	2-Me-THF	88	>99:1
7[d]	2-Me-THF	98	>99:1
8[e]	2-Me-THF	99	>99:1
9[e,f]	2-Me-THF	43	99:1

10[e,g]	2-Me-THF	**7**	93:7
11[e,h]	2-Me-THF	31	99:1

[a]Reactions were carried out with aldehyde **1d** (0.1 mmol, 1.0 equiv), vinyl chloride **2a** (1.5 equiv), $NiBr_2$•glyme (10 mol%), **L11** (5 mol %), NaI (2.0 equiv), $ZnBr_2$ (20 mol%), Zn (3.0 equiv), Et_3N (2.0 equiv) in solvent (1.0 mL) under nitrogen at 25 °C for 24 h; [b]Isolated yields; [c]Determined by HPLC on a chiral stationary phase; [d]**1d** (0.2 mmol), **2a** (1.5 equiv), $NiBr_2$•glyme (10 mol%), **L11** (5 mol%), Zn (3.0 equiv), $ZnBr_2$ (20 mol%), NaI (2.0 equiv), Et_3N (2.0 equiv), 2-Me-THF (1 mL), 24 h; [e]**1d** (0.2 mmol), **2a** (1.5 equiv), $NiBr_2$•glyme (5 mol%), **L11** (2.5 mol%), Zn (3.0 equiv), $ZnBr_2$ (20 mol%), NaI (2.0 equiv), Et_3N (2.0 equiv), 2-Me-THF (1 mL), 16 h;[f] Without $ZnBr_2$;[g]Without Et_3N; [h]Without NaI.

3. Reactions with poor results

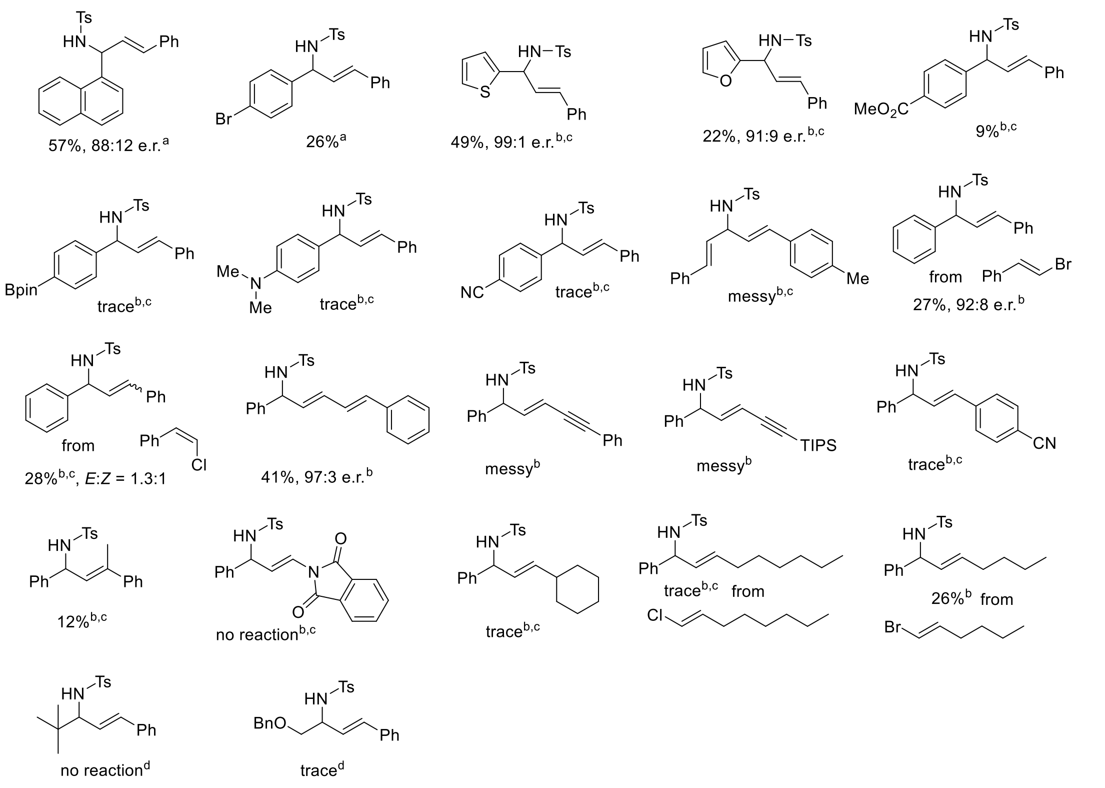

Supplementary Figure S5. Poor results in reductive alkenylation of imines. [a]Imine (0.2 mmol, 1.0 equiv), vinyl chloride (1.5 equiv), $NiBr_2$•glyme (10 mol %), **L1** (5 mol %), NaI (2.0 equiv), Zn (2.0 equiv) in THF (0.2 M) under nitrogen at 25 °C for 48 h. [b]$ZnBr_2$ (20 mol%) was added. [c]40 °C for 48 h. [d]$ZnBr_2$ (1.0 equiv) was added, **L3** as ligand, in THF (0.1 M) at 40 °C for 24 h.

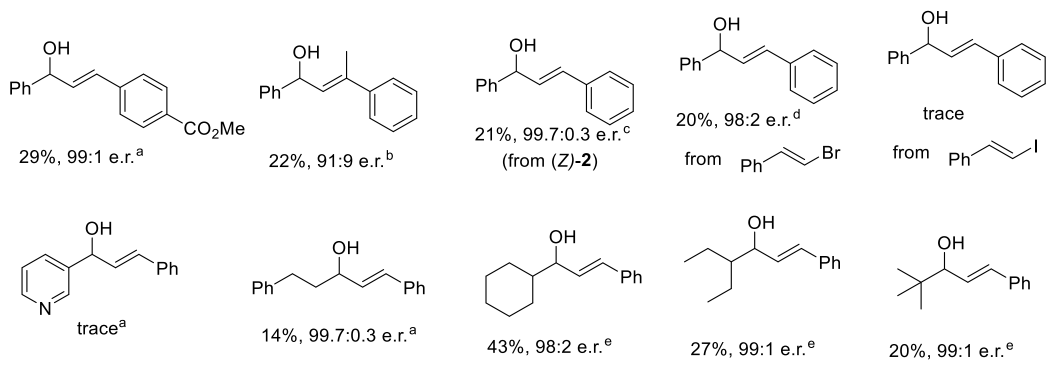

Supplementary Figure S6. Poor results in reductive alkenylation of aldehydes. [a]Aldehyde (0.2 mmol), vinyl chloride (1.5 equiv), $NiBr_2$•glyme (10 mol%), **L11** (5 mol%), Zn (3.0 equiv), $ZnBr_2$ (20 mol%), NaI (2.0 equiv), Et_3N (2.0 equiv), 2-Me-THF (1 mL) under nitrogen at 25 °C for 24 h.; [b]48 h; [c]40 °C, 24 h; [d]0.1 mmol scale in THF (0.1 M) under nitrogen at 25 °C for 24 h; [e]Et_3N (50 mol%), proton sponge (50 mol%), BHT (1.0 equiv), 25 °C, 48 h.

4. Synthesis of Substrates

Aromatic *N*-sulfonyl imines: was synthesized according to the literatures.[1,2] **Aliphatic *N*-tosyl imines**: was synthesized according to the literatures.[3] **Vinyl chloride**: was synthesized according to the literatures.[4]

(*E*)-1-(2-chlorovinyl)-4-(trifluoromethyl)benzene (S1), **methyl (*E*)-4-(2-chlorovinyl)benzoate (S2)**, **(*E*)-4-(2-chlorovinyl)benzonitrile (S3)**: were synthesized according to the literatures.[5]

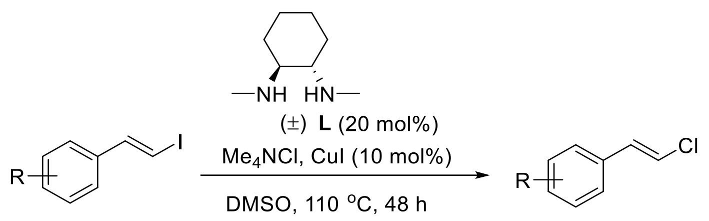

An oven dried 15 mL resealable pressure tube was charged with copper iodide (19 mg, 0.1 mmol), tetramethylammonium chloride (219 mg, 2.0 mmol) and the iodoalkene or bromoalkene (1.0 mmol). The tube was fitted with a rubber septum, evacuated under vacuum, backfilled with argon and trans-N,N'-dimethylcyclohexane-1,2-diamine (31 μL, 0.2 mmol) and ethanol (or DMSO when specified) (2 mL) were next added. The tube was closed with a Teflon-coated screw cap and the resulting suspension was stirred and heated at 110 °C in a preheated oil bath for 48 h (unless specified otherwise). When the reaction was run in ethanol, the crude reaction mixture was cooled to rt, diluted with ethyl acetate, filtered on a plug of silica gel and concentrated. When the reaction was run in DMSO, the crude reaction mixture was cooled to rt, diluted with water (15 mL), extracted thrice with diethyl ether and the combined organic layers were dried over anhydrous magnesium sulfate, filtered and concentrated. The crude residue was finally purified by flash column chromatography over silica gel to afford the desired chloroalkene.

(*E*)-4-(2-chlorovinyl)benzonitrile (S3)

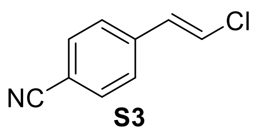

S3

White solid, R_f = 0.8 (petroleum ether/ ethyl acetate = 10/1), 50% yield.

^{1}H NMR (400 MHz, $CDCl_3$) δ 7.63 (d, J = 8.4 Hz, 2H), 7.41 (d, J = 8.5 Hz, 2H), 6.87 (d, J = 13.8 Hz, 1H), 6.82 (d, J = 13.7 Hz, 1H).

^{13}C NMR (101 MHz, $CDCl_3$) δ 139.2, 132.6, 131.8, 126.6, 122.8, 118.6, 111.5.

HRMS (ESI) *m/z*: $[M + Na]^+$ calcd. for C_9H_6ClNNa 186.0081, found 186.0081.

5. Synthesis of ligands

Synthesis of bimetallic ligands

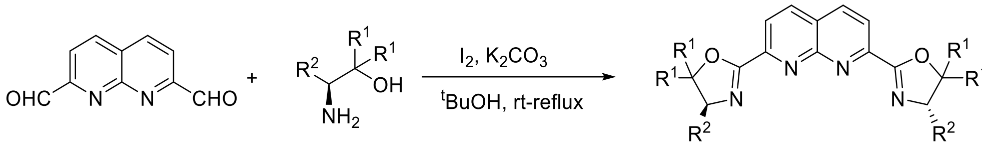

L1-L2, **L11**, **SL15**: were prepared according to a previous reported procedure[6]

A round-bottom flask was charged with amino alcohol (7.28 mmol, 2.03 equiv); 1,8-naphthyridine-2,7-dicarboxaldehyde (665.7 mg, 3.579 mmol, 1.00 equiv) and tert butanol (50 mL). The flask was equipped with a stir bar and a reflux condenser, placed under N_2, warmed to 35 °C, and stirring was commenced. After 4 h, the flask was opened to air, and K_2CO_3 (2.95 g, 21.4 mmol, 5.97 equiv) and I_2 (3.61 g, 14.2 mmol, 3.97 equiv) were sequentially added as solids. The flask was placed back under N_2 and heated to reflux. After 18 h, the reaction mixture was diluted with CH_2Cl_2 (250 mL), poured into a separatory funnel, and shaken vigorously with saturated aqueous $Na_2S_2O_3$ (2 × 20 mL). The yellow organic phase was separated, washed with brine (20 mL), dried over sodium sulfate, filtered, and concentrated to give the crude product. The crude product was purified by column chromatography to give the pure product.

2,7-bis((*S*)-4-isopropyl-5,5-diphenyl-4,5-dihydrooxazol-2-yl)-1,8-naphthyridine (L1)

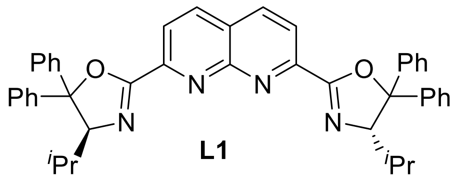

L1

Pale yellow solid, R_f = 0.3 (petroleum ether/ ethyl acetate = 3/1), 55% yield.

^{1}H NMR (400 MHz, $CDCl_3$) δ 8.46 (d, J = 8.4 Hz, 2H), 8.33 (d, J = 8.4 Hz, 2H), 7.71 (d, J = 7.2 Hz, 4H), 7.50 (d, J = 7.1 Hz, 4H), 7.41 – 7.26 (m, 12H), 4.96 (d, J = 4.6 Hz, 2H), 2.06 – 1.94 (m, 2H), 1.12 (d, J = 6.8 Hz, 6H), 0.69 (d, J = 6.5 Hz, 6H).

^{13}C NMR (101 MHz, $CDCl_3$) δ 161.2, 155.2, 151.1, 145.3, 140.3, 137.4, 128.4, 127.9, 127.8, 127.3, 127.2, 126.5, 124.4, 123.1, 93.9, 80.6, 30.4, 22.0, 17.1.

HRMS (ESI) *m/z*: $[M + Na]^+$ calcd. for $C_{44}H_{40}N_4NaO_2$ 679.3043, found 679.3044.

2,7-bis((*S*)-4-(*tert*-butyl)-5,5-dibutyl-4,5-dihydrooxazol-2-yl)-1,8-naphthyridine (L2)

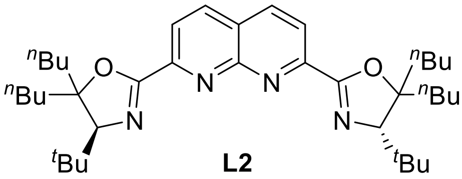

L2

Pale yellow solid, R_f = 0.6 (petroleum ether/ ethyl acetate = 2/1), 52% yield.

^{1}H NMR (400 MHz, CDCl$_3$) δ 8.37 (d, *J* = 8.4 Hz, 2H), 8.25 (d, *J* = 8.4 Hz, 2H), 3.81 (s, 2H), 2.03 (td, *J* = 12.3, 11.6, 2.7 Hz, 2H), 1.89 – 1.75 (m, 8H), 1.51 – 1.27 (m, 14H), 1.14 (s, 18H), 0.93 (dt, *J* = 17.7, 7.1 Hz, 12H).

^{13}C NMR (101 MHz, CDCl$_3$) δ 161.4, 155.0, 151.6, 136.9, 124.0, 122.9, 93.3, 80.9, 37.6, 35.0, 33.4, 28.3, 26.6, 25.6, 23.5, 23.1, 14.1, 14.0.

HRMS (ESI) *m/z*: [M + Na]$^+$ calcd. for $C_{38}H_{60}N_4NaO_2$ 627.4608, found 627.4608.

2,7-bis((*S*)-5,5-bis(3,3-dimethylbutyl)-4-phenyl-4,5-dihydrooxazol-2-yl)-1,8-naphthyridine (L11)

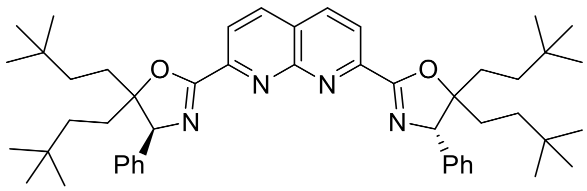

L11

White soild, R_f = 0.7 (petroleum ether/ethyl acetate = 2/1), 50% yield.

^{1}H NMR (400 MHz, CDCl$_3$) δ 8.51 (d, *J* = 7.2 Hz, 2H), 8.34 (d, *J* = 8.5 Hz, 2H), 7.38 – 7.27 (m, 10H), 5.20 (s, 2H), 2.01 – 1.85 (m, 4H), 1.54 (td, *J* = 13.5, 3.9 Hz, 2H), 1.44 (t, *J* = 8.6 Hz, 4H), 1.38 – 1.29 (m, 2H), 1.21 (td, *J* = 12.8, 3.7 Hz, 2H), 0.96 (s, 20H), 0.61 (s, 18H).

^{13}C NMR (101 MHz, CDCl$_3$) δ 163.4, 155.0, 151.3, 138.1, 137.3, 128.3, 128.1, 127.7, 124.4, 123.2, 93.3, 36.9, 36.5, 33.7, 30.2, 29.7, 29.4, 29.4, 28.9.

HRMS (ESI) *m/z*: [M + H]$^+$ calcd. for $C_{50}H_{69}N_4O_2$ 757.5415, found 757.5414.

2,7-bis((*S*)-5,5-dimethyl-4-phenyl-4,5-dihydrooxazol-2-yl)-1,8-naphthyridine (SL15)

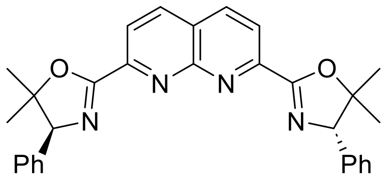

SL15

White soild, R_f = 0.3 (petroleum ether/ethyl acetate = 4/1), 69% yield.

^{1}H NMR (400 MHz, CDCl$_3$) δ 8.53 (d, *J* = 8.5 Hz, 2H), 8.36 (d, *J* = 8.4 Hz, 2H), 7.41 – 7.29 (m, 10H), 5.19 (s, 2H), 1.75 (s, 6H), 1.04 (s, 6H).

^{13}C NMR (101 MHz, CDCl$_3$) δ 163.3, 154.8, 151.2, 138.3, 137.4, 128.4, 127.7, 127.3, 124.4, 123.1, 88.9, 78.9, 29.3, 23.9.

HRMS (ESI) *m/z*: [M + H]$^+$ calcd. for $C_{30}H_{29}N_4O_2$ 477.2885, found 477.2884.

Synthesis of monomeric ligands

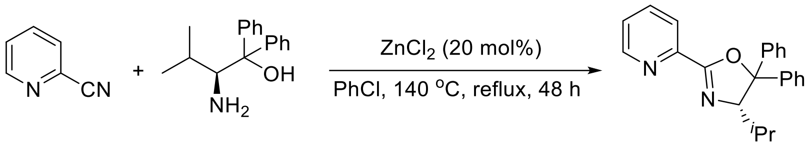

L7 was prepared according to a previous reported procedure[7]

To a 20 mL round bottom flask was added $ZnCl_2$ (28 mg, 0.2 mmol), and it was heated with a hot gun under high vacuum for 5 min and then cooled to room temperature, followed by the charge of argon. To this were added chlorobenzene (2 mL), picolinonitrile (0.104 g, 1 mmol, 1.0 equiv) and chiral aminoalcohol (1.5 mmol). The resulting solution was stirred 48 h at 140 °C (oil bath), and cooled to room temperature. After removing all volatiles under reduced pressure, the resulting solid was dissolved in the $CHCl_3$ and the organic layer was washed with water. The aqueous layer was extracted with $CHCl_3$ (3x) and the combined organic layers were washed with brine, dried over Na_2SO_4, filtered and concentrated in vacuo to afford the crude material, which was purified by silica gel column chromatography, eluting with hexane/ethyl acetate.

(*S*)-4-isopropyl-5,5-diphenyl-2-(pyridin-2-yl)-4,5-dihydrooxazole (L7)

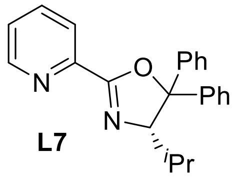

L7

White solid, R_f = 0.2 (petroleum ether/ ethyl acetate = 6/1), 35% yield.

^{1}H NMR (400 MHz, CDCl$_3$) δ 8.81 (ddd, *J* = 4.8, 1.8, 0.9 Hz, 1H), 8.18 (dt, *J* = 7.9, 1.1 Hz, 1H), 7.81 (td, *J* = 7.7, 1.8 Hz, 1H), 7.63 – 7.58 (m, 2H), 7.45 – 7.25 (m, 9H), 4.89 (d, *J* = 4.7 Hz, 1H), 1.92 (m, 1H), 1.09 (d, *J* = 6.8 Hz, 3H), 0.69 (d, *J* = 6.5 Hz, 3H).

^{13}C NMR (101 MHz, CDCl$_3$) δ 160.7, 150.1, 147.0, 145.3, 140.5, 136.6, 128.3, 127.9, 127.7, 127.3, 127.1, 126.4, 125.5, 124.0, 93.4, 80.4, 30.4, 22.1, 17.3.

HRMS (ESI) *m/z*: $[M + Na]^+$ calcd. for $C_{23}H_{22}N_2NaO$ 365.1624, found 365.1624.

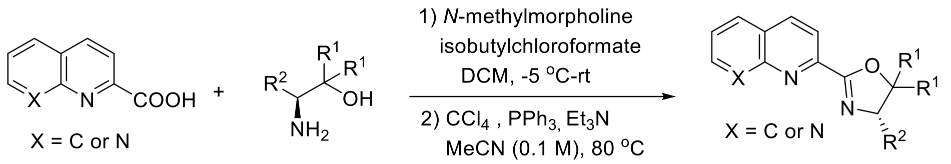

L8-L9, **SL13-SL14**: were prepared according to a previous reported procedure[8,9]

To a 50 mL flame dried round bottom flask equipped with a stir bar was placed of quinaldic acid (or 1,8-Naphthyridine-2-carboxylicacid, 2 mmol, 1 equiv.) and 18 mL of dichloromethane under a nitrogen atmosphere. Cooled to -5 °C in an ice bath. To this was added 330 μL of N-methylmorpholine (3 mmol, 1.5 equiv.) dropwise via a syringe. After 15 minutes, 301 μL of isobutylchloroformate (2.3 mmol, 1.15 equiv.) was added dropwise over a period of 2 minutes. Stirred for 30 minutes at -5 °C, 2.4 mmol of amino alcohol (1.2 equiv. and 253 μL of N-methylmorpholine (2.3 mmol, 1.15 equiv.) in dichloromethane was added dropwise via a syringe. The reaction solution was warmed up to room temperature after 1 hour and stirred for 12 hours.

Diluting the reaction solution with 50 mL dichloromethane and washed once with 2 mL of 1 M HCl, 10 mL of H_2O, 10 mL of brine, dried over Na_2SO_4, filtered and the solvent removed in vacuo. The crude product does not need to be purified and can be used directly in the next step.

An oven-dried, 100-mL round-bottom flask was charged with the crude product (2 mmol, 1.0 equiv), PPh_3 (1.31 g, 5 mmol, 2.5 equiv), dry CH_3CN (40 mL), Et_3N (0.7 mL, 5 mmol, 2.5 equiv), and CCl_4 (0.5mL, 5 mmol, 2.5 equiv) under N_2. The reaction mixture was refluxed in an oil bath at 80 °C for 24 h, then the reaction mixture was cooled to room temperature and quenched with water. The solvent was removed under reduced pressure and the resulting mixture was extracted with CH_2Cl_2. The combined organic layer was dried over anhydrous Na_2SO_4, filtered, and concentrated under reduced pressure. The crude product was purified by silica gel column chromatography to afford desired ligand.

(*S*)-4-isopropyl-5,5-diphenyl-2-(quinolin-2-yl)-4,5-dihydrooxazole (L8)

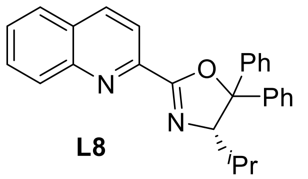

L8

White solid, R_f = 0.5 (petroleum ether/ ethyl acetate = 10/1), 70% yield

^{1}H NMR (400 MHz, CDCl$_3$) δ 8.38 (d, *J* = 8.5 Hz, 1H), 8.28 (s, 2H), 7.88 (d, *J* = 7.5 Hz, 1H), 7.78 (ddd, *J* = 8.5, 6.8, 1.5 Hz, 1H), 7.70 – 7.66 (m, 2H), 7.62 (ddd, *J* = 8.1, 6.9, 1.2 Hz, 1H), 7.47 – 7.43 (m, 2H), 7.42 – 7.37 (m, 2H), 7.36 – 7.27 (m, 4H), 4.93 (d, *J* = 5.2 Hz, 1H), 2.03 – 1.92 (m, 1H), 1.10 (d, *J* = 6.8 Hz, 3H), 0.77 (d, *J* = 6.5 Hz, 3H).

^{13}C NMR (101 MHz, CDCl$_3$) δ 161.2, 148.0, 147.1, 145.3, 140.6, 136.6, 130.7, 129.9, 128.8, 128.4, 127.9, 127.8, 127.8, 127.5, 127.4, 127.2, 126.5, 120.9, 93.5, 80.9, 30.5, 22.0, 17.6.

HRMS (ESI) *m/z*: $[M + Na]^+$ calcd. for $C_{27}H_{24}N_2NaO$ 415.1781, found 415.1781.

(*S*)-4-isopropyl-2-(1,8-naphthyridin-2-yl)-5,5-diphenyl-4,5-dihydrooxazole (L9)

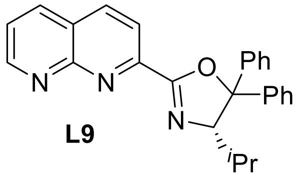

L9

White solid, R_f = 0.2 (petroleum ether/ ethyl acetate = 1/1), 45% yield

^{1}H NMR (400 MHz, CDCl$_3$) δ 9.22 (dd, *J* = 4.2, 2.0 Hz, 1H), 8.40 (d, *J* = 8.4 Hz, 1H), 8.30 – 8.21 (m, 2H), 7.72 (dd, *J* = 7.5, 1.7 Hz, 2H), 7.58 – 7.49 (m, 3H), 7.39 – 7.21 (m, 6H), 4.95 (d, *J* = 4.3 Hz, 1H), 2.01 (m, 1H), 1.13 (d, *J* = 6.8 Hz, 3H), 0.64 (d, *J* = 6.5 Hz, 3H).

^{13}C NMR (101 MHz, CDCl$_3$) δ 161.4, 155.6, 154.3, 150.4, 145.5, 140.4, 137.6, 136.9, 128.3, 127.8, 127.8, 127.2, 127.0, 126.3, 123.6, 123.1, 122.2, 93.7, 80.3, 30.3, 22.0, 16.8.

HRMS (ESI) *m/z*: $[M + Na]^+$ calcd. for $C_{26}H_{23}N_3NaO$ 416.1733, found 416.1731.

(*S*)-5,5-dimethyl-4-phenyl-2-(quinolin-2-yl)-4,5-dihydrooxazole (SL13)

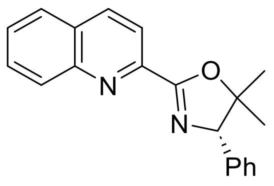

SL13

Yellow oil, R_f = 0.3 (petroleum ether/ethyl acetate = 1/1) , 83% yield

^{1}H NMR (400 MHz, CDCl$_3$) δ 8.41 – 8.33 (m, 2H), 8.28 (d, J = 8.6 Hz, 1H), 7.89 (d, J = 8.0 Hz, 1H), 7.78 (t, J = 8.5 Hz, 1H), 7.63 (t, J = 7.6 Hz, 1H), 7.41 – 7.30 (m, 5H), 5.19 (s, 1H), 1.79 (s, 3H), 1.08 (s, 3H).

^{13}C NMR (101 MHz, CDCl$_3$) δ 163.4, 147.7, 147.4, 138.4, 136.7, 130.6, 129.9, 128.9, 128.3, 127.9, 127.7, 127.6, 127.3, 121.1, 88.9, 78.7, 29.2, 24.0.

HRMS (ESI) *m/z*: [M + H]$^+$ calcd. for $C_{20}H_{19}N_2O$ 303.1492, found 303.1495.

(*S*)-5,5-dimethyl-2-(1,8-naphthyridin-2-yl)-4-phenyl-4,5-dihydrooxazole (SL14)

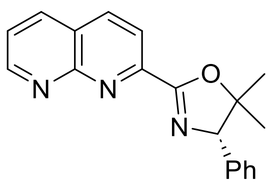

SL14

Yellow soild, R_f = 0.5 (dichloromethane/methanol = 20/1) , 47% yield

^{1}H NMR (400 MHz, CDCl$_3$) δ 9.20 (d, J = 2.2 Hz, 1H), 8.46 (d, J = 8.4 Hz, 1H), 8.29 (dd, J = 23.3, 8.3 Hz, 2H), 7.56 (dd, J = 8.4, 4.2 Hz, 1H), 7.39 – 7.28 (m, 5H), 5.18 (s, 1H), 1.76 (s, 3H), 1.04 (s, 3H).

^{13}C NMR (101 MHz, CDCl$_3$) δ 163.3, 155.5, 154.3, 150.5, 138.4, 137.8, 136.7, 128.3, 127.67, 127.3, 123.6, 123.1, 122.1, 88.9, 78.9, 29.3, 23.9.

HRMS (ESI) *m/z*: [M + H]$^+$ calcd. for $C_{19}H_{18}N_3O$ 304.1444, found 304.1447.

6. General procedure for synthesis of chiral product

General procedure A

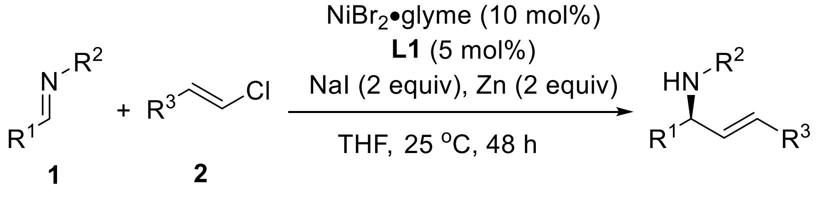

The NiBr$_2$•glyme (6.1 mg, 0.02 mmol, 10 mol %), **L1** (6.6 mg, 0.01 mmol, 5 mol %), NaI (60.0 mg, 0.4 mmol, 2.0 equiv) and Zn powder (26.0 mg, 0.4 mmol, 2.0 equiv) were introduced into a flame-dried Schlenk tube in an N_2-filled glove box. After taking out from the glove box, Schlenk tube was connected to Schlenk line under N_2. 1 mL dry THF was injected into the Schlenk tube, the mixture was stirred at r.t. for 30 min. The vinyl chloride (0.3 mmol, 1.5 equiv) and imine (0.2 mmol, 1.0 equiv) were sequentially added under nitrogen. The resulting mixture was stirred under particular temperature for 48 hours. The reaction mixture was cooled to room temperature, quenched with

saturated NH_4Cl (aq.) solution and extracted with EtOAc (3x). The organic phase was washed with brine, dried over anhydrous Na_2SO_4, filtered, and concentrated under reduced pressure. The crude material was purified using column chromatography to give the pure product.

General procedure B

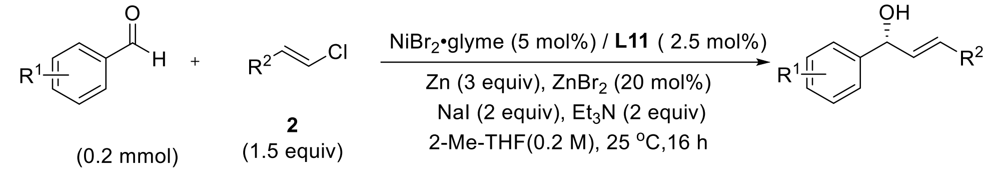

The $NiBr_2$•glyme (3.1 mg, 0.01 mmol, 5 mol %), **L11** (3.8 mg, 0.005 mmol, 2.5 mol %), NaI (60.0 mg, 0.4 mmol, 2.0 equiv) and Zn powder (39.0 mg, 0.6 mmol, 3.0 equiv) were introduced into a flame-dried Schlenk tube in an N_2-filled glove box. After taking out from the glove box, Schlenk tube was connected to Schlenk line under N_2. 1 mL dry 2-Me-THF was injected into the Schlenk tube, then Et_3N (56 μL, 0.4 mmol, 2.0 equiv) was added, the mixture was stirred at r.t. for 30 min. The vinyl chloride (0.3 mmol, 1.5 equiv), aldehyde (0.2 mmol, 1.0 equiv) were sequentially added under nitrogen. The resulting mixture was stirred under particular temperature for 16 hours. The reaction mixture was quenched with saturated NH_4Cl (aq.) solution and extracted with EtOAc (3x). The organic phase was washed with brine, dried over anhydrous Na_2SO_4, filtered, and concentrated under reduced pressure. The crude material was purified using column chromatography to give the pure product.

7. Characterization of Products

(*S*,*E*)-*N*-(1,3-diphenylallyl)-4-methylbenzenesulfonamide (3)

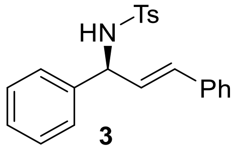

3

White solid, R_f = 0.3 (petroleum ether/ ethyl acetate = 6/1), 65.8 mg (91% yield), 98:2 e.r.
$[\alpha]_D^{20}$ = +32.6 (c = 1.03, CH_2Cl_2).
^{1}H NMR (400 MHz, $CDCl_3$) δ 7.70 (d, J = 7.9 Hz, 2H), 7.34 – 7.06 (m, 12H), 6.37 (d, J = 15.8 Hz, 1H), 6.11 (dd, J = 15.9, 6.8 Hz, 1H), 5.36 (d, J = 7.4 Hz, 1H), 5.15 (t, J = 7.2 Hz, 1H), 2.34 (s, 3H).
^{13}C NMR (101 MHz, $CDCl_3$) δ 143.3, 139.0, 137.3, 136.1, 132.8, 130.3, 128.7, 128.5, 128.2, 127.9, 127.8, 127.3, 127.1, 126.6, 60.9, 17.9.
HPLC (ChiralPak OD-H column), hexane/*i*-PrOH = 90:10, flow rate = 1.0 mL/min, λ = 254 nm, t_R = 16.767 min (minor), t_R = 24.439 min (major).
HRMS (ESI) *m/z*: $[M + Na]^+$ calcd. for $C_{22}H_{21}NNaO_2S$ 386.1185, found 386.1183.

(*S*,*E*)-*N*-(1,3-diphenylallyl)-4-(trifluoromethyl)benzenesulfonamide (4)

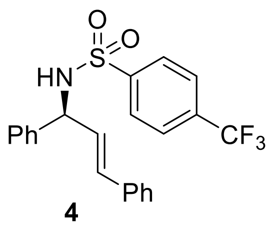

4

White solid, R_f = 0.2 (petroleum ether/ ethyl acetate = 6/1), 43.0 mg (52% yield), 91:9 e.r..

$[\alpha]_D^{20}$ = +12.3 (c = 0.86, CH_2Cl_2).

^{1}H NMR (400 MHz, $CDCl_3$) δ 7.85 (d, J = 8.0 Hz, 2H), 7.56 (d, J = 8.0 Hz, 2H), 7.33 – 7.15 (m, 10H), 6.41 (d, J = 15.9 Hz, 1H), 6.11 (dd, J = 15.8, 6.7 Hz, 1H), 5.58 (d, J = 7.4 Hz, 1H), 5.24 (t, J = 7.2 Hz, 1H).

^{13}C NMR (101 MHz, $CDCl_3$) δ 144.4, 138.9, 135.7, 134.0 (q, J = 33.3 Hz), 132.7, 128.8, 128.6, 128.2, 128.1, 127.7, 127.6, 127.1, 126.5, 125.9 (q, J = 4.0 Hz), 123.2 (q, J = 274.7 Hz), 60.2.

^{19}F NMR (376 MHz, $CDCl_3$) δ -63.2 (s, 3F).

HPLC (ChiralPak OD-H column), hexane/i-PrOH = 80:20, flow rate = 1.0 mL/min, λ = 254 nm, t_R = 9.073 min (minor), t_R = 14.772 min (major).

HRMS (ESI) m/z: $[M + Na]^+$ calcd. for $C_{22}H_{18}F_3NNaO_2S$ 440.0903, found 440.0901.

(S,E)-N-(1,3-diphenylallyl)methanesulfonamide (5)

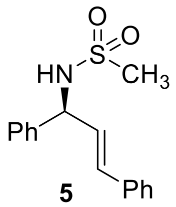

5

White solid, R_f = 0.2 (petroleum ether/ ethyl acetate = 6/1), 42.1 mg (73% yield), 88:12 e.r.

$[\alpha]_D^{20}$ = -3.5 (c = 0.84, CH_2Cl_2).

^{1}H NMR (400 MHz, $CDCl_3$) δ 7.48 – 7.25 (m, 10H), 6.65 (d, J = 15.8 Hz, 1H), 6.37 (dd, J = 15.8, 5.7 Hz, 1H), 5.34 – 5.24 (m, 2H), 2.79 (s, 3H).

^{13}C NMR (101 MHz, $CDCl_3$) δ 140.0, 136.0, 132.4, 129.1, 128.7, 128.5, 128.3, 128.2, 127.2, 126.7, 59.8, 42.2.

HPLC (ChiralPak OD-H column), hexane/i-PrOH = 80:20, flow rate = 1.0 mL/min, λ = 254 nm, t_R = 11.814 min (minor), t_R = 18.220 min (major).

HRMS (ESI) m/z: $[M + Na]^+$ calcd. for $C_{16}H_{17}NNaO_2S$ 310.0872, found 310.0869.

(S,E)-N-(1,3-diphenylallyl)benzenesulfonamide (6)

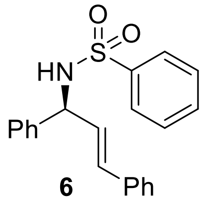

6

White solid, R_f = 0.2 (petroleum ether/ ethyl acetate = 6/1), 56.4 mg (81% yield), 89:11 e.r.

$[\alpha]_D^{20}$ = +16.4 (c = 1.12, CH_2Cl_2).

^{1}H NMR (400 MHz, $CDCl_3$) δ 7.81 (d, *J* = 7.7 Hz, 2H), 7.47 (t, *J* = 7.4 Hz, 1H), 7.37 (t, *J* = 7.7 Hz, 2H), 7.31 – 7.18 (m, 10H), 6.41 (d, *J* = 15.9 Hz, 1H), 6.15 (dd, *J* = 15.8, 6.6 Hz, 1H), 5.49 (d, *J* = 7.4 Hz, 1H), 5.19 (t, *J* = 7.1 Hz, 1H).
^{13}C NMR (101 MHz, $CDCl_3$) δ 140.8, 139.6, 136.1, 132.4, 132.2, 128.9, 128.8, 128.5, 128.3, 128.0, 127.9, 127.2, 127.1, 126.6, 59.9.
HPLC (ChiralPak OD-H column), hexane/*i*-PrOH = 90:10, flow rate = 1.0 mL/min, λ = 254 nm, t_R = 16.194 min (minor), t_R = 25.261 min (major).
HRMS (ESI) *m/z*: $[M + Na]^+$ calcd. for $C_{21}H_{19}NNaO_2S$ 372.1029, found 372.1028.

(*S*,*E*)-*N*-(1,3-diphenylallyl)-4-methoxybenzenesulfonamide (7)

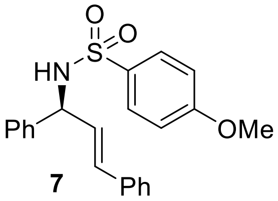

7

White solid, R_f = 0.2 (petroleum ether/ ethyl acetate = 5/1), 63.5 mg (84% yield), 90:10 e.r.
$[\alpha]_D^{20}$ = +21.1 (*c* = 1.27, CH_2Cl_2).
^{1}H NMR (400 MHz, $CDCl_3$) δ 7.74 (d, *J* = 8.4 Hz, 2H), 7.32 – 7.16 (m, 10H), 6.81 (d, *J* = 8.3 Hz, 2H), 6.39 (d, *J* = 15.8 Hz, 1H), 6.12 (dd, *J* = 15.8, 6.8 Hz, 1H), 5.44 (d, *J* = 7.3 Hz, 1H), 5.14 (t, *J* = 7.2 Hz, 1H), 3.76 (s, 3H).
^{13}C NMR (101 MHz, $CDCl_3$) δ 162.7, 139.8, 136.2, 132.4, 132.1, 129.5, 128.7, 128.5, 128.4, 127.9, 127.8, 127.1, 126.6, 114.0, 59.8, 55.5.
HPLC (ChiralPak OD-H column), hexane/*i*-PrOH = 80:20, flow rate = 1.0 mL/min, λ = 254 nm, t_R = 11.910 min (minor), t_R = 16.653 min (major).
HRMS (ESI) *m/z*: $[M + Na]^+$ calcd. for $C_{22}H_{21}NNaO_3S$ 402.1134, found 402.1139.

(*S*,*E*)-*N*-(1,3-diphenylallyl)-4-fluorobenzenesulfonamide (8)

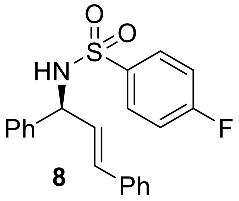

8

White solid, R_f = 0.2 (petroleum ether/ ethyl acetate = 6/1), 60.0 mg (82% yield), 98:2 e.r.
$[\alpha]_D^{20}$ = +17.1 (*c* = 1.20, CH_2Cl_2).
^{1}H NMR (400 MHz, $CDCl_3$) δ 7.81 – 7.73 (m, 2H), 7.34 – 7.18 (m, 10H), 6.99 (t, *J* = 8.5 Hz, 2H), 6.43 (d, *J* = 15.8 Hz, 1H), 6.15 (dd, *J* = 15.9, 6.7 Hz, 1H), 5.59 (d, *J* = 7.4 Hz, 1H), 5.19 (t, *J* = 7.2 Hz, 1H).
^{13}C NMR (101 MHz, $CDCl_3$) δ 164.9 (d, *J* = 255.5 Hz), 139.3, 136.9 (d, *J* = 3.0 Hz), 135.9, 132.3, 130.0 (d, *J* = 9.1 Hz), 128.8, 128.6, 128.0 (d, *J* = 2.0 Hz), 127.1, 126.6, 116.1, 115.8, 60.0.
^{19}F NMR (376 MHz, $CDCl_3$) δ -105.6 (s, 1F).
HPLC (ChiralPak OD-H column), hexane/*i*-PrOH = 80:20, flow rate = 1.0 mL/min, λ = 254 nm, t_R = 10.008 min (minor), t_R = 17.477 min (major).
HRMS (ESI) *m/z*: $[M + Na]^+$ calcd. for $C_{21}H_{18}FNNaO_2S$ 390.0934, found 390.0929.

(*S*,*E*)-4-methyl-*N*-(3-phenyl-1-(*p*-tolyl)allyl)benzenesulfonamide (9)

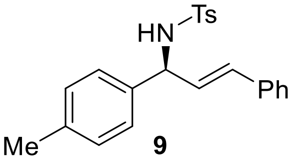

9

White solid, R_f = 0.2 (petroleum ether/ ethyl acetate = 10/1), 60.3 mg (80% yield), 98:2 e.r.
$[\alpha]_D^{20}$ = +20.8 (c = 0.90, CH_2Cl_2).
^{1}H NMR (400 MHz, $CDCl_3$) δ 7.70 (d, J = 7.9 Hz, 2H), 7.33 – 7.07 (m, 11H), 6.37 (d, J = 15.9 Hz, 1H), 6.10 (dd, J = 15.9, 6.7 Hz, 1H), 5.29 (d, J = 7.3 Hz, 1H), 5.11 (t, J = 7.1 Hz, 1H), 2.35 (s, 3H), 2.33 (s, 3H).
^{13}C NMR (101 MHz, $CDCl_3$) δ 143.2, 138.7, 137.6, 136.8, 136.2, 131.9, 129.4, 129.4, 128.5, 127.8, 127.4, 127.0, 126.6, 58.5, 22.0, 20.6.
HPLC (ChiralPak OD-H column), hexane/*i*-PrOH = 90:10, flow rate = 1.0 mL/min, λ = 254 nm, t_R = 18.101 min (minor), t_R = 24.439 min (major).
HRMS (ESI) *m/z*: $[M + Na]^+$ calcd. for $C_{23}H_{23}NNaO_2S$ 400.1342, found 400.1342.

(*S*,*E*)-*N*-(1-(4-fluorophenyl)-3-phenylallyl)-4-methylbenzenesulfonamide (10)

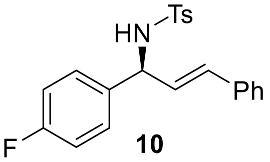

10

White solid, R_f = 0.3 (petroleum ether/ ethyl acetate = 6/1), 63.5 mg (83% yield), 95:5 e.r.
$[\alpha]_D^{20}$ = +27.4 (c = 1.26, CH_2Cl_2).
^{1}H NMR (400 MHz, $CDCl_3$) δ 7.68 (d, J = 7.8 Hz, 2H), 7.32 – 7.24 (m, 3H), 7.22 – 7.14 (m, 6H), 6.93 (t, J = 8.6 Hz, 2H), 6.33 (d, J = 15.8 Hz, 1H), 6.08 (dd, J = 15.9, 6.7 Hz, 1H), 5.53 (d, J = 7.5 Hz, 1H), 5.13 (t, J = 7.2 Hz, 1H), 2.34 (s, 3H).
^{13}C NMR (101 MHz, $CDCl_3$) δ 162.2 (d, J = 247.5 Hz), 143.4, 137.7, 136.0, 135.6 (d, J = 4.0 Hz), 132.3, 129.5, 128.9 (d, J = 8.1 Hz), 128.5, 128.0 (d, J = 12.1 Hz), 127.3, 126.6, 115.6, 115.4, 59.1, 21.4.
^{19}F NMR (376 MHz, $CDCl_3$) δ -114.4 (s, 1F).
HPLC (ChiralPak OD-H column), hexane/*i*-PrOH = 90:10, flow rate = 1.0 mL/min, λ = 254 nm, t_R = 19.106 min (minor), t_R = 27.112 min (major).
HRMS (ESI) *m/z*: $[M + Na]^+$ calcd. for $C_{22}H_{20}FNNaO_2S$ 404.1091, found 404.1088.

(*S*,*E*)-*N*-(1-(4-chlorophenyl)-3-phenylallyl)-4-methylbenzenesulfonamide (11)

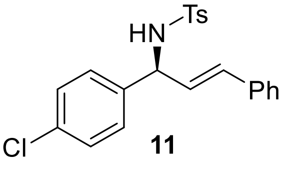

11

White solid, R_f = 0.2 (petroleum ether/ ethyl acetate = 6/1), 49.1mg (62% yield), 97:3 e.r.

[α]$_D$^{20} = +13.7 (c = 0.85, CH_2Cl_2).
^{1}H NMR (400 MHz, $CDCl_3$) δ 7.66 (d, J = 7.9 Hz, 2H), 7.35 – 7.13 (m, 11H), 6.32 (d, J = 15.9 Hz, 1H), 6.06 (dd, J = 15.9, 6.7 Hz, 1H), 5.52 (d, J = 7.6 Hz, 1H), 5.11 (t, J = 7.2 Hz, 1H), 2.34 (s, 3H).
^{13}C NMR (101 MHz, $CDCl_3$) δ 143.5, 138.2, 137.5, 135.9, 133.6, 132.5, 129.5, 128.8, 128.6, 128.5, 128.1, 127.5, 127.3, 126.6, 59.2, 21.4.
HPLC (ChiralPak OD-H column), hexane/*i*-PrOH = 80:20, flow rate = 1.0 mL/min, λ = 254 nm, t_R = 10.572 min (minor), t_R = 14.434 min (major).
HRMS (ESI) *m/z*: $[M + K]^+$ calcd. for $C_{22}H_{20}ClKNO_2S$ 436.0535, found 436.0536.

(*S*,*E*)-*N*-(1-(4-methoxyphenyl)-3-phenylallyl)-4-methylbenzenesulfonamide (12)

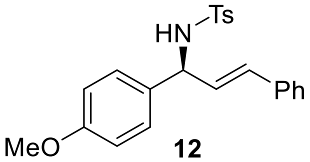

12

White solid, R_f = 0.2 (petroleum ether/ ethyl acetate = 5/1), 57.0 mg (72% yield), 99:1 e.r..
[α]$_D$^{20} = +8.9 (c = 1.14, CH_2Cl_2).
^{1}H NMR (400 MHz, $CDCl_3$) δ 7.69 (d, J = 7.9 Hz, 2H), 7.33 – 7.09 (m, 9H), 6.79 (d, J = 8.3 Hz, 2H), 6.36 (d, J = 15.8 Hz, 1H), 6.10 (dd, J = 15.8, 6.7 Hz, 1H), 5.30 (d, J = 7.3 Hz, 1H), 5.09 (t, J = 7.0 Hz, 1H), 3.78 (s, 3H), 2.34 (s, 3H).
^{13}C NMR (101 MHz, $CDCl_3$) δ 154.8, 141.8, 137.9, 136.2, 133.1, 131.8, 129.4, 128.5, 128.3, 127.8, 127.4, 126.6, 113.6, 57.9, 54.7, 23.9.
HPLC (ChiralPak OD-H column), hexane/*i*-PrOH = 80:20, flow rate = 1.0 mL/min, λ = 254 nm, t_R = 14.806 min (minor), t_R = 17.180 min (major).
HRMS (ESI) *m/z*: $[M + Na]^+$ calcd. for $C_{23}H_{23}NNaO_3S$ 416.1291, found 416.1290.

(*S*,*E*)-*N*-(1-(2-methoxyphenyl)-3-phenylallyl)-4-methylbenzenesulfonamide (13)

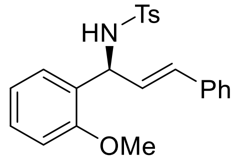

13

White solid, R_f = 0.2 (petroleum ether/ ethyl acetate = 5/1), 31.3 mg (40% yield), 88:12 e.r.
[α]$_D$^{20} = +19.8 (c = 0.62, CH_2Cl_2).
^{1}H NMR (400 MHz, $CDCl_3$) δ 7.60 (d, J = 7.9 Hz, 2H), 7.30 – 7.15 (m, 6H), 7.06 (t, J = 8.0 Hz, 3H), 6.83 (t, J = 7.5 Hz, 1H), 6.72 (d, J = 8.3 Hz, 1H), 6.36 (d, J = 15.9 Hz, 1H), 6.25 (dd, J = 15.9, 6.0 Hz, 1H), 5.82 (d, J = 9.3 Hz, 1H), 5.22 (t, J = 7.1 Hz, 1H), 3.74 (s, 3H), 2.31 (s, 3H).
^{13}C NMR (101 MHz, $CDCl_3$) δ 156.4, 142.8, 137.9, 136.5, 131.0, 129.1, 129.1, 129.0, 128.4, 127.6, 127.2, 127.1, 126.5, 120.9, 110.9, 58.4, 55.3, 21.4.
HPLC (ChiralPak OD-H column), hexane/*i*-PrOH = 80:20, flow rate = 1.0 mL/min, λ = 254 nm, t_R = 8.685 min (minor), t_R = 12.558 min (major).
HRMS (ESI) *m/z*: $[M + Na]^+$ calcd. for $C_{23}H_{23}NNaO_3S$ 416.1291, found 416.1291.

(*S*,*E*)-*N*-(1-(4-(benzyloxy)phenyl)-3-phenylallyl)-4-methylbenzenesulfonamide (14)

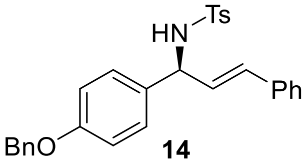

14

White solid, R_f = 0.2 (petroleum ether/ ethyl acetate = 5/1), 59.0 mg (63% yield), 98:2 e.r.
$[\alpha]_D^{20}$ = +4.4 (c = 0.98, CH_2Cl_2).
^{1}H NMR (400 MHz, $CDCl_3$) δ 7.70 (d, J = 7.7 Hz, 2H), 7.47 – 7.35 (m, 5H), 7.32 – 7.12 (m, 9H), 6.88 (d, J = 8.2 Hz, 2H), 6.37 (d, J = 15.8 Hz, 1H), 6.10 (dd, J = 15.8, 6.6 Hz, 1H), 5.26 (d, J = 7.2 Hz, 1H), 5.10 (t, J = 7.1 Hz, 1H), 5.05 (s, 2H), 2.35 (s, 3H).
^{13}C NMR (101 MHz, $CDCl_3$) δ 158.4, 143.2, 137.9, 136.9, 136.2, 132.1, 131.8, 129.5, 128.7, 128.5, 128.4, 128.1, 127.9, 127.5, 127.4, 126.6, 115.0, 70.1, 59.3, 21.5.
HPLC (ChiralPak AD-H column), hexane/*i*-PrOH = 70:30, flow rate = 1.0 mL/min, λ = 254 nm, t_R = 14.278 min (minor), t_R = 21.117 min (major).
HRMS (ESI) *m/z*: $[M + Na]^+$ calcd. for $C_{29}H_{27}NNaO_3S$ 492.1604, found 492.1605.

(*S*,*E*)-4-methyl-*N*-(3-phenyl-1-(4-(trifluoromethoxy)phenyl)allyl)benzenesulfonamide (15)

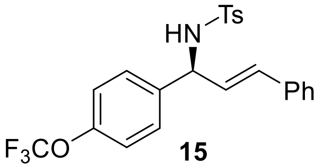

15

White solid, R_f = 0.2 (petroleum ether/ ethyl acetate = 8/1), 51.1 mg (57% yield), 97:3 e.r.
$[\alpha]_D^{20}$ = +15.6 (c = 0.97, CH_2Cl_2).
^{1}H NMR (400 MHz, $CDCl_3$) δ 7.65 (d, J = 8.0 Hz, 2H), 7.30 – 7.19 (m, 7H), 7.13 (d, J = 8.0 Hz, 2H), 7.07 (d, J = 8.3 Hz, 2H), 6.35 (d, J = 15.9 Hz, 1H), 6.09 (dd, J = 15.8, 6.7 Hz, 1H), 5.65 (d, J = 7.6 Hz, 1H), 5.16 (t, J = 7.2 Hz, 1H), 2.32 (s, 3H).
^{13}C NMR (101 MHz, $CDCl_3$) δ 148.6, 143.5, 138.4, 137.5, 135.8, 132.6, 129.5, 128.6, 128.5, 128.1, 127.5, 127.2, 126.6, 121.1, 120.4 (q, J = 257.6 Hz), 11, 59.1, 21.3.
^{19}F NMR (376 MHz, $CDCl_3$) δ -57.9 (s, 3F).
HPLC (ChiralPak OD-H column), hexane/*i*-PrOH = 80:20, flow rate = 1.0 mL/min, λ = 254 nm, t_R = 8.798 min (minor), t_R = 13.066 min (major).
HRMS (ESI) *m/z*: $[M + K]^+$ calcd. for $C_{23}H_{20}F_3KNO_3S$ 486.0748, found 486.0747.

(*S*,*E*)-4-methyl-*N*-(1-(4-(methylthio)phenyl)-3-phenylallyl)benzenesulfonamide (16)

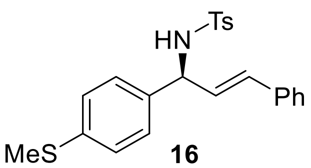

16

White solid, R_f = 0.2 (petroleum ether/ ethyl acetate = 5/1), 61.1 mg (74% yield), 98:2 e.r.
$[\alpha]_D^{20}$ = -2.3 (c = 1.20, CH_2Cl_2).

^{1}H NMR (400 MHz, CDCl$_3$) δ 7.66 (d, J = 7.9 Hz, 2H), 7.30 – 7.25 (m, 3H), 7.22 – 7.07 (m, 8H), 6.35 (d, J = 15.8 Hz, 1H), 6.08 (dd, J = 15.9, 6.6 Hz, 1H), 5.22 (d, J = 7.3 Hz, 1H), 5.09 (t, J = 7.1 Hz, 1H), 2.47 (s, 3H), 2.35 (s, 3H).
^{13}C NMR (101 MHz, CDCl$_3$) δ 143.3, 138.3, 137.7, 136.4, 136.0, 132.2, 129.5, 128.5, 128.0, 128.0, 127.6, 127.3, 126.7, 126.6, 59.4, 21.5, 15.8.
HPLC (ChiralPak OD-H column), hexane/*i*-PrOH = 80:20, flow rate = 1.0 mL/min, λ = 254 nm, t_R = 16.341 min (minor), t_R = 19.155 min (major).
HRMS (ESI) *m/z*: [M + Na]$^+$ calcd. for $C_{23}H_{23}NNaO_2S_2$ 432.1062, found 432.1060.

(*S*,*E*)-*N*-(1-(2,3-dihydrobenzo[*b*][1,4]dioxin-6-yl)-3-phenylallyl)-4 methylbenzenesulfonamide (17)

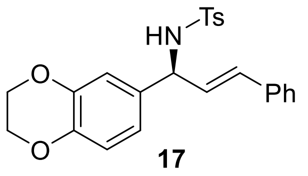

17

White solid, R_f = 0.2 (petroleum ether/ ethyl acetate = 4/1), 67.4 mg (80% yield), 99:1 e.r.
[α]$_D$^{20} = +10.9 (c = 1.14, CH_2Cl_2).
^{1}H NMR (400 MHz, CDCl$_3$) δ 7.69 (d, J = 8.1 Hz, 2H), 7.33 – 7.13 (m, 7H), 6.80 – 6.62 (m, 3H), 6.36 (d, J = 15.8 Hz, 1H), 6.06 (dd, J = 15.9, 6.7 Hz, 1H), 5.23 (d, J = 7.1 Hz, 1H), 5.02 (t, J = 7.0 Hz, 1H), 4.21 (s, 4H), 2.35 (s, 3H).
^{13}C NMR (101 MHz, CDCl$_3$) δ 143.5, 143.2, 137.8, 136.2, 133.0, 131.8, 129.4, 128.5, 128.3, 127.8, 127.4, 126.6, 120.1, 117.4, 116.1, 64.3, 64.3, 59.3, 21.5.
HPLC (ChiralPak OD-H column), hexane/*i*-PrOH = 80:20, flow rate = 1.0 mL/min, λ = 254 nm, t_R = 18.645 min (minor), t_R = 21.613 min (major).
HRMS (ESI) *m/z*: [M + Na]$^+$ calcd. for $C_{24}H_{23}NNaO_4S$ 444.1240, found 444.1242.

(*S*,*E*)-*N*-(4-(1-((4-methylphenyl)sulfonamido)-3-phenylallyl)phenyl)acetamide (18)

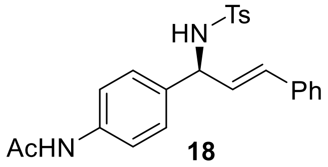

18

White solid, R_f = 0.2 (petroleum ether/ ethyl acetate = 2/1), 56.5 mg (67% yield), 99:1 e.r.
[α]$_D$^{20} = +0.9 (c = 0.42, CH_2Cl_2).
^{1}H NMR (400 MHz, DMSO-d_6) δ 9.91 (s, 1H), 8.29 (d, J = 8.6 Hz, 1H), 7.62 (d, J = 8.1 Hz, 2H), 7.47 (d, J = 8.3 Hz, 2H), 7.32 – 7.12 (m, 9H), 6.20 (d, J = 15.9 Hz, 1H), 6.05 (dd, J = 15.9, 7.2 Hz, 1H), 4.93 (t, J = 7.8 Hz, 1H), 2.23 (s, 3H), 2.03 (s, 3H).
^{13}C NMR (101 MHz, DMSO) δ 168.7, 142.7, 139.2, 138.8, 136.6, 135.7, 130.5, 129.7, 129.6, 128.9, 128.0, 127.7, 127.1, 126.7, 119.3, 59.2, 24.4, 21.3.
HPLC (ChiralPak AD-H column), hexane/*i*-PrOH = 80:20, flow rate = 1.0 mL/min, λ = 254 nm, t_R = 17.566 min (minor), t_R = 22.688 min (major).
HRMS (ESI) *m/z*: [M + K]$^+$ calcd. for $C_{24}H_{24}KN_2O_3S$ 459.1139, found 459.1139.

(*S*,*E*)-4-methyl-*N*-(3-phenyl-1-(4-(trifluoromethyl)phenyl)allyl)benzenesulfonamide (19)

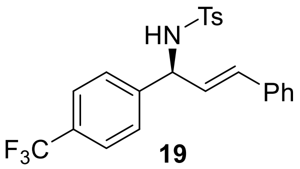

19

White solid, R_f = 0.2 (petroleum ether/ ethyl acetate = 10/1), 18.2 mg (21% yield), 98:2 e.r.

[α]$_D^{20}$ = +17.1 (*c* = 0.84, CH_2Cl_2).

^{1}H NMR (400 MHz, $CDCl_3$) δ7.63 (d, *J* = 7.9 Hz, 2H), 7.46 (d, *J* = 7.9 Hz, 2H), 7.38 – 7.24 (m, 5H), 7.20 (d, *J* = 7.1 Hz, 2H), 7.11 (d, *J* = 7.9 Hz, 2H), 6.35 (d, *J* = 15.8 Hz, 1H), 6.10 (dd, *J* = 15.8, 6.8 Hz, 1H), 5.72 (d, *J* = 7.7 Hz, 1H), 5.20 (t, *J* = 7.3 Hz, 1H), 2.32 (s, 3H).

^{13}C NMR (101 MHz, $CDCl_3$) δ 143.6, 143.5, 137.4, 135.7, 132.9, 129.6 (q, *J* = 24.9 Hz), 129.5, 128.6, 128.2, 127.6, 127.2, 127.1, 126.6, 125.6 (q, *J* = 4.0 Hz), 124.0 (q, *J* = 272.7 Hz), 59.5, 21.3.

^{19}F NMR (376 MHz, $CDCl_3$) δ -62.6 (s, 3F).

HPLC (ChiralPak OD-H column), hexane/*i*-PrOH = 80:20, flow rate = 1.0 mL/min, λ = 254 nm, t_R = 10.344 min (minor), t_R = 14.451 min (major).

HRMS (ESI) *m/z*: [M + Na]$^+$ calcd. for $C_{23}H_{20}F_3NNaO_2S$ 454.1059, found 454.1058.

(*S*,*E*)-4-methyl-*N*-(1-(naphthalen-2-yl)-3-phenylallyl)benzenesulfonamide (20)

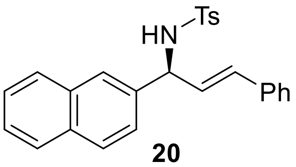

20

White solid, R_f = 0.3 (petroleum ether/ ethyl acetate = 5/1), 73.5 mg (89% yield), 98:2 e.r.

[α]$_D^{20}$ = -5.2 (*c* = 1.46, CH_2Cl_2).

^{1}H NMR (400 MHz, $CDCl_3$) δ 7.86 – 7.56 (m, 6H), 7.51 – 7.44 (m, 2H), 7.35 – 7.18 (m, 6H), 7.04 (d, *J* = 7.8 Hz, 2H), 6.43 (d, *J* = 15.8 Hz, 1H), 6.21 (dd, *J* = 15.9, 6.4 Hz, 1H), 5.39 (d, *J* = 7.5 Hz, 1H), 5.31 (t, *J* = 7.1 Hz, 1H), 2.23 (s, 3H).

^{13}C NMR (101 MHz, $CDCl_3$) δ 141.8, 137.7, 136.8, 136.1, 133.8, 132.8, 132.4, 129.8, 128.6, 128.5, 128.1, 128.0, 127.6, 127.3, 126.6, 126.3, 126.2, 126.1, 124.9, 58.1, 18.9.

HPLC (ChiralPak IBN5 column), hexane/*i*-PrOH = 80:20, flow rate = 1.0 mL/min, λ = 254 nm, t_R = 14.944 min (minor), t_R = 18.200 min (major).

HRMS (ESI) *m/z*: [M + Na]$^+$ calcd. for $C_{26}H_{23}NNaO_2S$ 436.1342, found 436.1345.

(*S*,*E*)-4-methyl-*N*-(1-(1-methyl-1*H*-indol-5-yl)-3-phenylallyl)benzenesulfonamide (21)

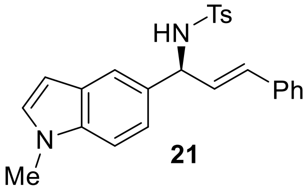

21

White solid, R_f = 0.3 (petroleum ether/ ethyl acetate = 3/1), 47.0 mg (56% yield), 99:1 e.r.

[α]$_D^{20}$ = -4.0 (*c* = 0.57, CH_2Cl_2).

^{1}H NMR (400 MHz, $CDCl_3$) δ 7.70 (d, J = 8.3 Hz, 2H), 7.41 (s, 1H), 7.32 – 7.20 (m, 6H), 7.14 (d, J = 8.0 Hz, 2H), 7.09 – 7.04 (m, 2H), 6.45 (d, J = 15.9 Hz, 1H), 6.41 (d, J = 2.8 Hz, 1H), 6.21 (dd, J = 15.8, 6.4 Hz, 1H), 5.23 (t, J = 6.5 Hz, 1H), 4.98 (d, J = 6.8 Hz, 1H), 3.78 (s, 3H), 2.33 (s, 3H).
^{13}C NMR (101 MHz, $CDCl_3$) δ 143.1, 137.9, 136.4, 136.3, 131.4, 130.7, 129.6, 129.4, 129.2, 128.5, 128.4, 127.7, 127.4, 126.6, 120.8, 119.5, 109.6, 101.1, 60.2, 32.9, 21.4.
HPLC (ChiralPak IBN5 column), hexane/*i*-PrOH = 80:20, flow rate = 1.0 mL/min, λ = 254 nm, t_R = 23.977 min (minor), t_R = 25.482 min (major).
HRMS (ESI) *m/z*: [M + Na]$^+$ calcd. for $C_{25}H_{24}N_2NaO_2S$ 439.1451, found 439.1453.

(*S*,*E*)-*N*-(1-(furan-3-yl)-3-phenylallyl)-4-methylbenzenesulfonamide (22)

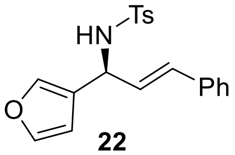

22

White solid, R_f = 0.2 (petroleum ether/ ethyl acetate = 6/1), 43.2 mg (61% yield), 96:4 e.r.
$[\alpha]_D^{20}$ = +53.0 (c = 0.86, CH_2Cl_2).
^{1}H NMR (400 MHz, $CDCl_3$) δ 7.74 (d, J = 7.9 Hz, 2H), 7.37 – 7.16 (m, 9H), 6.40 (d, J = 15.8 Hz, 1H), 6.24 (d, J = 2.3 Hz, 1H), 6.03 (dd, J = 15.8, 6.1 Hz, 1H), 5.14 – 4.98 (m, 2H), 2.35 (s, 3H).
^{13}C NMR (101 MHz, $CDCl_3$) δ 143.7, 143.5, 139.9, 137.8, 136.0, 132.3, 129.6, 128.5, 128.0, 127.3, 126.6, 125.1, 109.6, 52.2, 21.4.
HPLC (ChiralPak OD-H column), hexane/*i*-PrOH = 80:20, flow rate = 1.0 mL/min, λ = 254 nm, t_R = 9.580 min (minor), t_R = 11.925 min (major).
HRMS (ESI) *m/z*: [M + Na]$^+$ calcd. for $C_{20}H_{19}NNaO_3S$ 376.0978, found 376.0979.

(*S*,*E*)-4-methyl-*N*-(3-phenyl-1-(thiophen-3-yl)allyl)benzenesulfonamide (23)

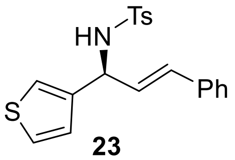

23

White solid, R_f = 0.2 (petroleum ether/ ethyl acetate = 5/1), 52.5 mg (71% yield), 98:2 e.r.
$[\alpha]_D^{20}$ = +31.9 (c = 1.04, CH_2Cl_2).
^{1}H NMR (400 MHz, $CDCl_3$) δ 7.72 (d, J = 7.9 Hz, 2H), 7.36 – 7.13 (m, 8H), 7.08 (s, 1H), 6.93 (d, J = 4.9 Hz, 1H), 6.37 (d, J = 15.8 Hz, 1H), 6.08 (dd, J = 15.8, 7.0 Hz, 1H), 5.43 (d, J = 7.9 Hz, 1H), 5.22 (t, J = 7.5 Hz, 1H), 2.33 (s, 3H).
^{13}C NMR (101 MHz, $CDCl_3$) δ 143.8, 141.5, 138.4, 136.1, 132.8, 129.5, 128.5, 127.9, 127.7, 127.3, 126.6, 122.3, 55.8, 21.5.
HPLC (ChiralPak OD-H column), hexane/*i*-PrOH = 80:20, flow rate = 1.0 mL/min, λ = 254 nm, t_R = 10.325 min (minor), t_R = 13.478 min (major).
HRMS (ESI) *m/z*: [M + K]$^+$ calcd. for $C_{20}H_{19}KNO_2S_2$ 408.0489, found 408.0488.

(*S*,*E*)-4-methyl-*N*-(3-phenyl-1-(pyridin-3-yl)allyl)benzenesulfonamide (24)

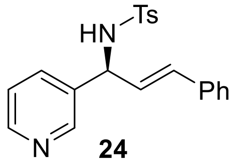

White solid, R_f = 0.3 (petroleum ether/ ethyl acetate = 2/1), 37.3 mg (51% yield), 71:29 e.r..
[α]$_D^{20}$ = +1.1 (c = 0.48, CH_2Cl_2).
^{1}H NMR (400 MHz, $CDCl_3$) δ 8.49 – 8.41 (m, 2H), 7.66 (d, J = 7.9 Hz, 2H), 7.60 (d, J = 8.0 Hz, 1H), 7.33 – 7.14 (m, 8H), 6.32 (d, J = 15.8 Hz, 1H), 6.08 (dd, J = 15.9, 6.8 Hz, 1H), 5.99 (d, J = 7.4 Hz, 1H), 5.16 (t, J = 7.2 Hz, 1H), 2.33 (s, 3H).
^{13}C NMR (101 MHz, $CDCl_3$) δ 149.0, 148.6, 143.6, 137.5, 135.7, 135.5, 134.9, 133.0, 129.6, 128.6, 128.2, 127.2, 127.1, 126.6, 123.5, 57.6, 21.4.
HPLC (ChiralPak OD-H column), hexane/*i*-PrOH = 70:30, flow rate = 1.0 mL/min, λ = 254 nm, t_R = 9.196 min (minor), t_R = 13.206 min (major).
HRMS (ESI) *m/z*: [M + Na]$^+$ calcd. for $C_{21}H_{20}N_2NaO_2S$ 387.1138, found 387.1139.

(*S*,*E*)-*N*-(1-(6-methoxypyridin-3-yl)-3-phenylallyl)-4-methylbenzenesulfonamide (25)

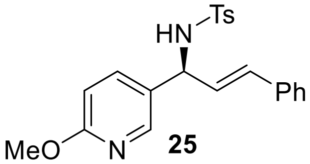

White solid, R_f = 0.2 (petroleum ether/ ethyl acetate = 5/1), 44.2 mg (56% yield), 98:2 e.r.
[α]$_D^{20}$ = +8.3 (c = 0.88, CH_2Cl_2).
^{1}H NMR (400 MHz, $CDCl_3$) δ 7.96 (d, J = 2.5 Hz, 1H), 7.69 – 7.64 (m, 2H), 7.44 (dd, J = 8.6, 2.6 Hz, 1H), 7.30 – 7.23 (m, 3H), 7.21 – 7.13 (m, 4H), 6.61 (d, J = 8.6 Hz, 1H), 6.34 (d, J = 15.7 Hz, 1H), 6.08 (dd, J = 15.8, 6.6 Hz, 1H), 5.53 (d, J = 7.3 Hz, 1H), 5.09 (t, J = 6.6 Hz, 1H), 3.90 (s, 3H), 2.33 (s, 3H).
^{13}C NMR (101 MHz, $CDCl_3$) δ 163.8, 145.6, 143.5, 137.6, 137.5, 135.8, 132.5, 129.5, 128.5, 128.1, 127.5, 127.3, 126.6, 111.0, 57.2, 53.5, 21.4.
HPLC (ChiralPak OD-H column), hexane/*i*-PrOH = 80:20, flow rate = 1.0 mL/min, λ = 254 nm, t_R = 12.322 min (minor), t_R = 16.040 min (major).
HRMS (ESI) *m/z*: [M + K]$^+$ calcd. for $C_{22}H_{22}KN_2O_3S$ 433.0983, found 433.0980.

(*R*,*E*)-4-methyl-*N*-(1-phenylhex-1-en-3-yl)benzenesulfonamide (26)

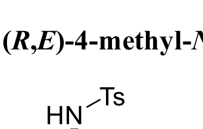

White solid, R_f = 0.2 (petroleum ether/ ethyl acetate = 8/1), 49.8 mg (76% yield), 98:2 e.r.
[α]$_D^{20}$ = +94.0 (c = 0.94, $CHCl_3$).
^{1}H NMR (400 MHz, $CDCl_3$) δ 7.78 (d, J = 7.9 Hz, 2H), 7.30 – 7.15 (m, 5H), 7.11 (d, J = 6.4 Hz, 2H), 6.20 (d, J = 15.9 Hz, 1H), 5.73 (dd, J = 15.9, 7.7 Hz, 1H), 5.21 (d, J = 8.0 Hz, 1H), 3.94 (p, J = 7.5 Hz, 1H), 2.29 (s, 3H), 1.66 – 1.47 (m, 2H), 1.44 – 1.26 (m, 2H), 0.88 (t, J = 7.4 Hz, 3H).

^{13}C NMR (101 MHz, CDCl$_3$) δ 143.2, 138.2, 136.4, 131.3, 129.5, 129.0, 128.4, 127.6, 127.3, 126.3, 56.3, 38.0, 21.4, 18.7, 13.7.

HPLC (ChiralPak AD-H column), hexane/*i*-PrOH = 90:10, flow rate = 1.0 mL/min, λ = 254 nm, t_R = 12.067 min (major), t_R = 13.661 min (minor).

HRMS (ESI) *m/z*: [M + H]$^+$ calcd. for $C_{19}H_{24}NO_2S$ 330.1522, found 330.1517.

(*R*,*E*)-4-methyl-*N*-(1-phenyloct-1-en-3-yl)benzenesulfonamide (27)

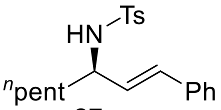

27

White solid, R_f = 0.2 (petroleum ether/ ethyl acetate = 8/1), 51.0 mg (71% yield), 99:1 e.r.

[α]$_D^{20}$ = +75.4 (*c* = 1.02, CHCl$_3$).

^{1}H NMR (400 MHz, CDCl$_3$) δ 7.77 (d, *J* = 8.3 Hz, 2H), 7.29 – 7.17 (m, 5H), 7.14 – 7.10 (m, 2H), 6.22 (d, *J* = 15.8 Hz, 1H), 5.75 (dd, *J* = 15.9, 7.6 Hz, 1H), 5.02 (d, *J* = 7.9 Hz, 1H), 3.93 (p, *J* = 7.3 Hz, 1H), 2.31 (s, 3H), 1.64 – 1.50 (m, 2H), 1.37 – 1.19 (m, 6H), 0.86 (t, *J* = 6.6 Hz, 3H).

^{13}C NMR (101 MHz, CDCl$_3$) δ 143.2, 138.2, 136.4, 131.3, 129.5, 129.1, 128.4, 127.6, 127.3, 126.4, 56.4, 35.9, 31.4, 25.1, 22.5, 21.4, 14.0.

HPLC (ChiralPak OJ-H column), hexane/*i*-PrOH = 90:10, flow rate = 1.0 mL/min, λ = 254 nm, t_R = 11.567 min (major), t_R = 17.614 min (minor).

HRMS (ESI) *m/z*: [M + Na]$^+$ calcd. for $C_{21}H_{27}NNaO_2S$ 380.1655, found 380.1650.

(*R*,*E*)-*N*-(1,5-diphenylpent-1-en-3-yl)-4-methylbenzenesulfonamide (28)

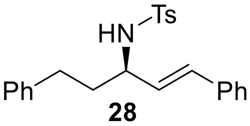

28

White solid, R_f = 0.2 (petroleum ether/ ethyl acetate = 5/1), 62.9 mg (80% yield), 97:3 e.r..

[α]$_D^{20}$ = +35.7 (*c* = 1.04, CHCl$_3$).

^{1}H NMR (400 MHz, CDCl$_3$) δ 7.80 – 7.71 (m, 2H), 7.32 – 7.25 (m, 5H), 7.24 – 7.11 (m, 7H), 6.23 (d, *J* = 15.9 Hz, 1H), 5.78 (dd, *J* = 15.9, 7.6 Hz, 1H), 5.09 (d, *J* = 8.0 Hz, 1H), 3.99 (p, *J* = 7.2 Hz, 1H), 2.76 – 2.61 (m, 2H), 2.32 (s, 3H), 2.03 – 1.82 (m, 2H).

^{13}C NMR (101 MHz, CDCl$_3$) δ 143.3, 141.1, 138.1, 136.2, 131.8, 129.6, 128.5, 128.5, 128.4, 127.8, 127.3, 126.4, 126.1, 56.0, 37.5, 31.7, 21.4.

HPLC (ChiralPak IBN5 column), hexane/*i*-PrOH = 70:30, flow rate = 1.0 mL/min, λ = 254 nm, t_R = 8.576 min (major), t_R = 9.745 min (minor).

HRMS (ESI) *m/z*: [M + H]$^+$ calcd. for $C_{24}H_{26}NO_2S$ 392.1679, found 392.1674.

(*R*,*E*)-*N*-(5-((tert-butyldiphenylsilyl)oxy)-1-phenylpent-1-en-3-yl)-4-methylbenzenesulfonamide (29)

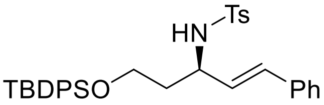

29

Colorless oil, R_f = 0.2 (petroleum ether/ ethyl acetate = 6/1), 58.1 mg (51% yield), 99:1 e.r.
[α]$_D^{20}$ = +40.8 (*c* = 0.94, $CHCl_3$).
^{1}H NMR (400 MHz, $CDCl_3$) δ 7.76 (d, *J* = 8.0 Hz, 2H), 7.69 – 7.63 (m, 4H), 7.50 – 7.23 (m, 9H), 7.23 – 7.17 (m, 4H), 6.45 (d, *J* = 15.8 Hz, 1H), 5.88 (dd, *J* = 15.8, 6.9 Hz, 1H), 5.81 (d, *J* = 6.9 Hz, 1H), 4.30 (p, *J* = 6.4 Hz, 1H), 3.84 – 3.75 (m, 1H), 3.72 – 3.63 (m, 1H), 2.37 (s, 3H), 1.87 – 1.79 (m, 2H), 1.13 (s, 9H).
^{13}C NMR (101 MHz, $CDCl_3$) δ 143.0, 138.3, 136.4, 135.6, 132.9, 132.8, 131.7, 129.9, 129.5, 128.5, 128.4, 127.8, 127.8, 127.7, 127.4, 126.5, 61.1, 54.8, 37.1, 26.9, 21.5, 19.1.
HPLC (ChiralPak AD-H column), hexane/*i*-PrOH = 90:10, flow rate = 1.0 mL/min, λ = 254 nm, t_R = 6.774 min (minor), t_R = 7.254 min (major).
HRMS (ESI) *m/z*: [M + K]$^+$ calcd. for $C_{34}H_{39}KNO_3SSi$ 608.2051, found 608.2047.

(*R*,*E*)-*N*-(6-(benzyloxy)-1-phenylhex-1-en-3-yl)-4-methylbenzenesulfonamide (30)

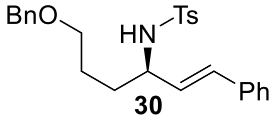

30

White solid, R_f = 0.2 (petroleum ether/ ethyl acetate = 6/1), 64.5 mg (74% yield), 98:2 e.r.
[α]$_D^{20}$ = +70.2 (*c* = 0.98, $CHCl_3$).
^{1}H NMR (400 MHz, $CDCl_3$) δ 7.73 (d, *J* = 8.3 Hz, 2H), 7.42 – 7.30 (m, 5H), 7.29 – 7.20 (m, 3H), 7.20 – 7.09 (m, 4H), 6.21 (d, *J* = 15.8 Hz, 1H), 5.75 (dd, *J* = 15.9, 7.5 Hz, 1H), 5.32 (d, *J* = 7.8 Hz, 1H), 4.51 (s, 2H), 3.96 (q, *J* = 6.7 Hz, 1H), 3.47 (t, *J* = 5.7 Hz, 2H), 2.30 (s, 3H), 1.76 – 1.64 (m, 4H).
^{13}C NMR (101 MHz, $CDCl_3$) δ 143.1, 138.3, 138.2, 136.3, 131.5, 129.5, 128.8, 128.4, 128.4, 127.8, 127.7, 127.6, 127.3, 126.4, 73.0, 69.7, 56.2, 32.8, 25.7, 21.4.
HPLC (ChiralPak AD-H column), hexane/*i*-PrOH = 80:20, flow rate = 1.0 mL/min, λ = 254 nm, t_R = 10.571 min (minor), t_R = 12.038 min (major).
HRMS (ESI) *m/z*: [M + K]$^+$ calcd. for $C_{26}H_{29}KNO_3S$ 474.1500, found 474.1501.

(*R*,*E*)-4-methyl-*N*-(5-methyl-1-phenylhex-1-en-3-yl)benzenesulfonamide (31)

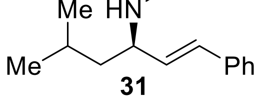

31

White solid, R_f = 0.2 (petroleum ether/ ethyl acetate = 8/1), 48.1 mg (70% yield), 99:1 e.r.
[α]$_D^{20}$ = +77.3 (*c* = 0.94, $CHCl_3$).
^{1}H NMR (400 MHz, $CDCl_3$) δ 7.76 (d, *J* = 8.3 Hz, 2H), 7.28 – 7.08 (m, 7H), 6.21 (d, *J* = 15.8 Hz, 1H), 5.69 (dd, *J* = 15.8, 7.8 Hz, 1H), 5.04 (d, *J* = 7.9 Hz, 1H), 4.00 (p, *J* = 7.7 Hz, 1H), 2.28 (s, 3H), 1.70 (tt, *J* = 13.3, 7.1 Hz, 1H), 1.51 – 1.33 (m, 2H), 0.89 (dd, *J* = 6.7, 5.0 Hz, 6H).
^{13}C NMR (101 MHz, $CDCl_3$) δ 143.1, 138.3, 136.4, 131.2, 129.5, 129.2, 128.3, 127.6, 127.3, 126.3, 54.8, 45.1, 24.4, 22.4, 22.4, 21.4.
HPLC (ChiralPak OJ-H column), hexane/*i*-PrOH = 90:10, flow rate = 1.0 mL/min, λ = 254 nm, t_R = 9.887 min (major), t_R = 11.572 min (minor).
HRMS (ESI) *m/z*: [M + Na]$^+$ calcd. for $C_{20}H_{25}NNaO_2S$ 366.1498, found 366.1493.

(*R*,*E*)-*N*-(4-ethyl-1-phenylhex-1-en-3-yl)-4-methylbenzenesulfonamide (32)

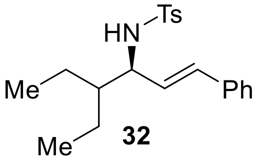

White solid, R_f = 0.2 (petroleum ether/ ethyl acetate = 8/1), 53.5 mg (75% yield), 99:1 e.r.

$[\alpha]_D^{20}$ = +72.1 (c = 1.02, $CHCl_3$).

^{1}H NMR (400 MHz, $CDCl_3$) δ 7.76 (d, J = 8.3 Hz, 2H), 7.31 – 7.13 (m, 5H), 7.12 – 7.05 (m, 2H), 6.12 (d, J = 15.8 Hz, 1H), 5.75 (dd, J = 15.9, 7.8 Hz, 1H), 5.11 (d, J = 8.5 Hz, 1H), 4.00 (td, J = 8.0, 4.1 Hz, 1H), 2.27 (s, 3H), 1.48 – 1.22 (m, 5H), 0.89 (dt, J = 11.5, 7.1 Hz, 6H).

^{13}C NMR (101 MHz, $CDCl_3$) δ 143.1, 138.2, 136.4, 131.8, 129.5, 128.3, 127.5, 127.4, 127.3, 126.3, 58.1, 46.6, 22.0, 21.8, 21.4, 11.5.

HPLC (ChiralPak IBN5 column), hexane/*i*-PrOH = 90:10, flow rate = 1.0 mL/min, λ = 254 nm, t_R = 8.302 min (major), t_R = 8.836 min (minor).

HRMS (ESI) *m/z*: $[M + Na]^+$ calcd. for $C_{21}H_{27}NNaO_2S$ 380.1655, found 380.1653.

(*R*,*E*)-*N*-(1-cyclohexyl-3-phenylallyl)-4-methylbenzenesulfonamide (33)

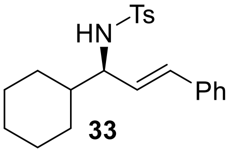

White solid, R_f = 0.2 (petroleum ether/ ethyl acetate = 10/1), 63.1 mg (85% yield), 99:1 e.r.

$[\alpha]_D^{20}$ = +60.1 (c = 1.00, $CHCl_3$).

^{1}H NMR (400 MHz, $CDCl_3$) δ 7.75 (d, J = 7.9 Hz, 2H), 7.30 – 7.20 (m, 3H), 7.15 (d, J = 7.9 Hz, 2H), 7.08 (d, J = 7.3 Hz, 2H), 6.07 (d, J = 15.8 Hz, 1H), 5.73 (dd, J = 15.9, 8.1 Hz, 1H), 5.13 (d, J = 8.5 Hz, 1H), 3.74 (q, J = 7.7 Hz, 1H), 2.27 (s, 3H), 1.90 – 1.82 (m, 1H), 1.79 – 1.61 (m, 4H), 1.56 – 1.42 (m, 1H), 1.25 – 1.09 (m, 3H), 1.09 – 0.95 (m, 2H).

^{13}C NMR (101 MHz, $CDCl_3$) δ 143.1, 138.3, 136.4, 132.0, 129.4, 128.3, 127.5, 127.4, 127.3, 126.3, 61.5, 42.9, 29.1, 29.1, 26.3, 26.0, 26.0, 21.3.

HPLC (ChiralPak AD-H column), hexane/*i*-PrOH = 90:10, flow rate = 1.0 mL/min, λ = 254 nm, t_R = 13.563 min (minor), t_R = 14.523 min (major).

HRMS (ESI) *m/z*: $[M + H]^+$ calcd. for $C_{22}H_{28}NO_2S$ 370.1835, found 370.1831.

(*S*,*E*)-4-methyl-*N*-(1-phenyl-3-(*o*-tolyl)allyl)benzenesulfonamide (34)

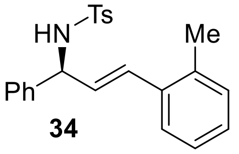

White solid, R_f = 0.2 (petroleum ether/ ethyl acetate = 6/1), 49.9 mg (66% yield), 96:4 e.r.

$[\alpha]_D^{20}$ = +32.6 (c = 0.96, CH_2Cl_2).

^{1}H NMR (400 MHz, $CDCl_3$) δ 7.72 (d, J = 7.9 Hz, 2H), 7.32 – 7.23 (m, 5H), 7.22 – 7.08 (m, 6H), 6.63 (d, J = 15.7 Hz, 1H), 6.02 (dd, J = 15.7, 6.9 Hz, 1H), 5.45 (d, J = 7.4 Hz, 1H), 5.17 (t, J = 7.2 Hz, 1H), 2.35 (s, 3H), 2.23 (s, 3H).

^{13}C NMR (101 MHz, CDCl$_3$) δ 143.3, 139.9, 137.9, 135.6, 135.2, 130.2, 130.0, 129.6, 129.5, 128.7, 127.8, 127.3, 127.1, 126.0, 125.8, 60.0, 21.4, 19.7.

HPLC (ChiralPak OD-H column), hexane/*i*-PrOH = 80:20, flow rate = 1.0 mL/min, λ = 254 nm, t_R = 8.265 min (minor), t_R = 10.671 min (major).

HRMS (ESI) *m/z*: [M + Na]$^+$ calcd. for $C_{23}H_{23}NNaO_2S$ 400.1342, found 400.1333.

(*S*,*E*)-4-methyl-*N*-(1-phenyl-3-(*m*-tolyl)allyl)benzenesulfonamide (35)

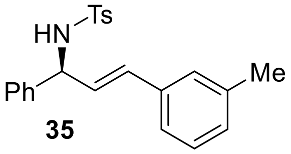

35

White solid, R_f = 0.2 (petroleum ether/ ethyl acetate = 6/1), 60.2 mg (80% yield), 96:4 e.r.

[α]$_D^{20}$ = +24.8 (*c* = 1.18, CH_2Cl_2).

^{1}H NMR (400 MHz, CDCl$_3$) δ 7.71 (d, *J* = 7.9 Hz, 2H), 7.33 – 7.13 (m, 8H), 7.10 – 6.97 (m, 3H), 6.33 (d, *J* = 15.8 Hz, 1H), 6.10 (dd, *J* = 15.8, 6.7 Hz, 1H), 5.43 (d, *J* = 7.4 Hz, 1H), 5.14 (t, *J* = 7.1 Hz, 1H), 2.35 (s, 3H), 2.33 (s, 3H).

^{13}C NMR (101 MHz, CDCl$_3$) δ 143.2, 139.8, 138.0, 137.8, 136.1, 132.2, 129.5, 128.7, 128.7, 128.4, 128.1, 127.8, 127.4, 127.1, 123.8, 59.8, 21.4, 21.4.

HPLC (ChiralPak OD-H column), hexane/*i*-PrOH = 80:20, flow rate = 1.0 mL/min, λ = 254 nm, t_R = 7.673 min (minor), t_R = 10.264 min (major).

HRMS (ESI) *m/z*: [M + Na]$^+$ calcd. for $C_{23}H_{23}NNaO_2S$ 400.1342, found 400.1335.

(*S*,*E*)-*N*-(3-(4-fluorophenyl)-1-phenylallyl)-4-methylbenzenesulfonamide (36)

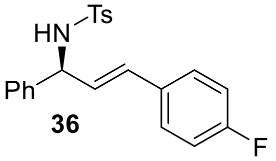

36

White solid, R_f = 0.3 (petroleum ether/ ethyl acetate = 6/1), 65.2 mg (85% yield), 96:4 e.r.

[α]$_D^{20}$ = +24.2 (*c* = 1.30, CH_2Cl_2).

^{1}H NMR (400 MHz, CDCl$_3$) δ 7.69 (d, *J* = 7.8 Hz, 2H), 7.31 – 7.10 (m, 9H), 6.96 (t, *J* = 8.5 Hz, 2H), 6.34 (d, *J* = 15.8 Hz, 1H), 6.03 (dd, *J* = 15.9, 6.8 Hz, 1H), 5.51 (d, *J* = 7.5 Hz, 1H), 5.12 (t, *J* = 7.2 Hz, 1H), 2.34 (s, 3H).

^{13}C NMR (101 MHz, CDCl$_3$) δ 162.4 (d, *J* = 248.5 Hz), 143.3, 139.6, 137.8, 132.3 (d, *J* = 3.0 Hz), 130.8, 129.4, 128.7, 128.1 (d, *J* =8.1 Hz), 127.9, 127.3, 127.1, 115.5, 115.3, 59.8, 21.4.

^{19}F NMR (376 MHz, CDCl$_3$) δ -113.9 (s, 1F).

HPLC (ChiralPak IBN5 column), hexane/*i*-PrOH = 80:20, flow rate = 1.0 mL/min, λ = 254 nm, t_R = 9.112 min (minor), t_R = 14.999 min (major).

HRMS (ESI) *m/z*: [M + Na]$^+$ calcd. for $C_{22}H_{20}FNNaO_2S$ 404.1091, found 404.1090.

(*S*,*E*)-*N*-(3-(4-chlorophenyl)-1-phenylallyl)-4-methylbenzenesulfonamide (37)

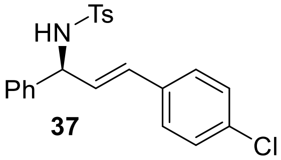

37

White solid, R_f = 0.3 (petroleum ether/ ethyl acetate = 6/1), 61.5 mg (77% yield), 98:2 e.r.
[α]$_D^{20}$ = +24.6 (c = 1.22, CH_2Cl_2).
^{1}H NMR (400 MHz, $CDCl_3$) δ 7.68 (d, J = 7.8 Hz, 2H), 7.29 – 7.08 (m, 11H), 6.34 (d, J = 15.8 Hz, 1H), 6.09 (dd, J = 15.9, 6.6 Hz, 1H), 5.54 (d, J = 7.5 Hz, 1H), 5.12 (t, J = 7.2 Hz, 1H), 2.34 (s, 3H).
^{13}C NMR (101 MHz, $CDCl_3$) δ 143.3, 139.5, 137.7, 134.7, 133.5, 130.7, 129.5, 129.0, 128.8, 128.6, 127.9, 127.8, 127.3, 127.1, 59.7, 21.5.
HPLC (ChiralPak IBN5 column), hexane/*i*-PrOH = 80:20, flow rate = 1.0 mL/min, λ = 254 nm, t_R = 9.812 min (minor), t_R = 18.742 min (major).
HRMS (ESI) *m/z*: $[M + K]^+$ calcd. for $C_{22}H_{20}ClKNO_2S$ 436.0535, found 436.0535.

(*S*,*E*)-*N*-(3-(4-bromophenyl)-1-phenylallyl)-4-methylbenzenesulfonamide (38)

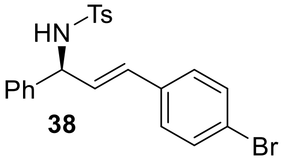

38

White solid, R_f = 0.3 (petroleum ether/ ethyl acetate = 6/1), 61.5 mg (70% yield), 99:1 e.r.
[α]$_D^{20}$ = +29.2 (c = 0.60, CH_2Cl_2).
^{1}H NMR (400 MHz, $CDCl_3$) δ 7.67 (d, J = 8.2 Hz, 2H), 7.39 (d, J = 8.1 Hz, 2H), 7.25 – 7.13 (m, 7H), 7.05 (d, J = 8.0 Hz, 2H), 6.33 (d, J = 15.8 Hz, 1H), 6.10 (dd, J = 15.8, 6.6 Hz, 1H), 5.34 (d, J = 7.4 Hz, 1H), 5.11 (t, J = 7.2 Hz, 1H), 2.35 (s, 3H).
^{13}C NMR (101 MHz, $CDCl_3$) δ 143.3, 139.4, 137.7, 135.1, 131.6, 130.8, 129.5, 129.1, 128.8, 128.1, 128.0, 127.3, 127.0, 121.7, 59.7, 21.9.
HPLC (ChiralPak IBN5 column), hexane/*i*-PrOH = 80:20, flow rate = 1.0 mL/min, λ = 254 nm, t_R = 10.019 min (minor), t_R = 19.562 min (major).
HRMS (ESI) *m/z*: $[M + K]^+$ calcd. for $C_{22}H_{20}BrKNO_2S$ 480.0030, found 480.0029.

(*S*,*E*)-*N*-(3-(4-methoxyphenyl)-1-phenylallyl)-4-methylbenzenesulfonamide (39)

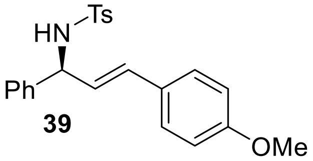

39

White solid, R_f = 0.2 (petroleum ether/ ethyl acetate = 5/1), 63.1 mg (80% yield), 92:8 e.r.
[α]$_D^{20}$ = +30.1 (c = 1.08, CH_2Cl_2).
^{1}H NMR (400 MHz, $CDCl_3$) δ 7.68 (d, J = 7.9 Hz, 2H), 7.33 – 7.19 (m, 5H), 7.17 – 7.10 (m, 4H), 6.81 (d, J = 8.6 Hz, 2H), 6.30 (d, J = 15.8 Hz, 1H), 5.96 (dd, J = 15.8, 6.9 Hz, 1H), 5.39 (d, J = 7.4 Hz, 1H), 5.12 (t, J = 7.2 Hz, 1H), 3.81 (s, 3H), 2.34 (s, 3H).
^{13}C NMR (101 MHz, $CDCl_3$) δ 159.4, 143.2, 140.0, 137.8, 131.6, 129.4, 128.9, 128.7, 127.8, 127.7, 127.3, 127.1, 126.1, 113.9, 59.9, 55.3, 21.5.
HPLC (ChiralPak IBN5 column), hexane/*i*-PrOH = 80:20, flow rate = 1.0 mL/min, λ = 254 nm, t_R = 12.933 min (minor), t_R = 18.130 min (major).
HRMS (ESI) *m/z*: $[M + Na]^+$ calcd. for $C_{23}H_{23}NNaO_3S$ 416.1291, found 416.1288.

(*S*,*E*)-*N*-(3-(benzo[*d*][1,3]dioxol-5-yl)-1-phenylallyl)-4-methylbenzenesulfonamide (40)

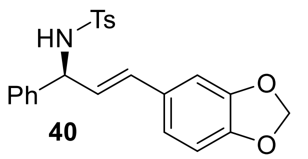

40

White solid, R_f = 0.2 (petroleum ether/ ethyl acetate = 4/1), 70.2 mg (86% yield), 97:3 e.r.

[α]$_D^{20}$ = +25.2 (*c* = 0.82, CH_2Cl_2).

^{1}H NMR (400 MHz, $CDCl_3$) δ 7.68 (d, *J* = 7.9 Hz, 2H), 7.31 – 7.13 (m, 7H), 6.72 – 6.67 (m, 2H), 6.63 (d, *J* = 7.9 Hz, 1H), 6.26 (d, *J* = 15.8 Hz, 1H), 5.98 – 5.88 (m, 3H), 5.29 (d, *J* = 7.2 Hz, 1H), 5.10 (t, *J* = 7.2 Hz, 1H), 2.36 (s, 3H).

^{13}C NMR (101 MHz, $CDCl_3$) δ 147.9, 147.4, 143.2, 139.8, 137.8, 131.7, 130.6, 129.5, 128.7, 127.8, 127.3, 127.1, 126.5, 121.4, 108.2, 105.8, 101.1, 59.8, 21.4.

HPLC (ChiralPak IBN5 column), hexane/*i*-PrOH = 80:20, flow rate = 1.0 mL/min, λ = 254 nm, t_R = 14.202 min (minor), t_R = 19.727 min (major).

HRMS (ESI) *m/z*: $[M + Na]^+$ calcd. for $C_{23}H_{21}NNaO_4S$ 430.1083, found 430.1074.

(*S*,*E*)-4-methyl-*N*-(1-phenyl-3-(4-(trifluoromethyl)phenyl)allyl)benzenesulfonamide (41)

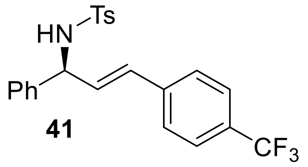

41

White solid, R_f = 0.2 (petroleum ether/ ethyl acetate = 6/1), 63.7 mg (74% yield), 99:1 e.r.

[α]$_D^{20}$ = +25.3 (*c* = 1.26, CH_2Cl_2).

^{1}H NMR (400 MHz, $CDCl_3$) δ 7.70 (d, *J* = 8.1 Hz, 2H), 7.51 (d, *J* = 7.9 Hz, 2H), 7.33 – 7.19 (m, 7H), 7.14 (d, *J* = 7.9 Hz, 2H), 6.44 (d, *J* = 15.9 Hz, 1H), 6.23 (dd, *J* = 15.9, 6.6 Hz, 1H), 5.68 (d, *J* = 7.6 Hz, 1H), 5.15 (t, *J* = 7.2 Hz, 1H), 2.32 (s, 3H).

^{13}C NMR (101 MHz, $CDCl_3$) δ 143.4, 139.7, 139.2, 137.7, 131.1, 130.5, 129.6 (q, *J* = 7.9 Hz), 129.5, 128.8, 127.3, 127.1, 126.7, 125.3 (q, *J* = 4.0 Hz), 124.1 (q, *J* = 272.7 Hz), 59.7, 21.4.

^{19}F NMR (376 MHz, $CDCl_3$) δ -62.5 (s, 3F).

HPLC (ChiralPak OD-H column), hexane/*i*-PrOH = 80:20, flow rate = 1.0 mL/min, λ = 254 nm, t_R = 7.515 min (minor), t_R = 22.015 min (major).

HRMS (ESI) *m/z*: $[M + Na]^+$ calcd. for $C_{23}H_{20}F_3NNaO_2S$ 454.1059, found 454.1059.

methyl (*S*,*E*)-4-(3-((4-methylphenyl)sulfonamido)-3-phenylprop-1-en-1-yl)benzoate (42)

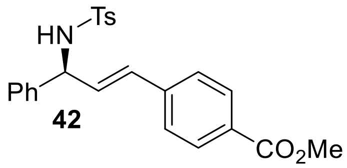

42

White solid, R_f = 0.2 (petroleum ether/ ethyl acetate = 5/1), 48.0 mg (57% yield), 99:1 e.r.

[α]$_D^{20}$ = +27.2 (*c* = 0.96, CH_2Cl_2).

^{1}H NMR (400 MHz, $CDCl_3$) δ 7.93 (d, *J* = 8.4 Hz, 2H), 7.68 (d, *J* = 8.3 Hz, 2H), 7.30 – 7.18 (m, 7H), 7.15 (d, *J* = 8.1 Hz, 2H), 6.42 (d, *J* = 15.9 Hz, 1H), 6.23 (dd, *J* = 15.9, 6.6 Hz, 1H), 5.50 (d, *J* = 7.5 Hz, 1H), 5.15 (t, *J* = 7.1 Hz, 1H), 3.92 (s, 3H), 2.32 (s, 3H).

^{13}**C NMR (101 MHz, CDCl$_3$) δ** 166.8, 143.4, 140.6, 139.3, 137.7, 131.0, 131.0, 129.8, 129.5, 129.2, 128.8, 128.0, 127.3, 127.1, 126.4, 59.7, 52.1, 21.4.
HPLC (ChiralPak OD-H column), hexane/*i*-PrOH = 80:20, flow rate = 1.0 mL/min, λ = 254 nm, t_R = 13.371 min (minor), t_R = 25.107 min (major).
HRMS (ESI) *m/z*: [M + K]$^+$ calcd. for $C_{24}H_{23}KNO_4S$ 460.0979, found 460.0975.

(*S*,*E*)-4-methyl-N-(3-(naphthalen-2-yl)-1-phenylallyl)benzenesulfonamide (43)

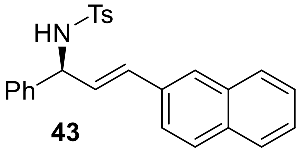

43

White solid, R_f = 0.2 (petroleum ether/ ethyl acetate = 5/1), 74.6 mg (90% yield), 95:5 e.r.
[α]$_D$^{20} = +47.6 (*c* = 0.80, CH_2Cl_2).
^{1}**H NMR (400 MHz, CDCl$_3$) δ** 7.84 – 7.68 (m, 5H), 7.60 – 7.53 (m, 1H), 7.51 – 7.44 (m, 2H), 7.38 (d, *J* = 8.6 Hz, 1H), 7.33 – 7.22 (m, 5H), 7.15 (d, *J* = 7.9 Hz, 2H), 6.52 (d, *J* = 15.8 Hz, 1H), 6.22 (dd, *J* = 15.9, 6.7 Hz, 1H), 5.28 (d, *J* = 7.4 Hz, 1H), 5.20 (t, *J* = 7.1 Hz, 1H), 2.29 (s, 3H).
^{13}**C NMR (101 MHz, CDCl$_3$) δ** 143.3, 139.7, 137.8, 133.6, 133.4, 133.1, 132.2, 129.5, 128.8, 128.6, 128.1, 128.0, 127.9, 127.7, 127.4, 127.1, 126.7, 126.4, 126.1, 123.5, 60.3, 21.4.
HPLC (ChiralPak OD-H column), hexane/*i*-PrOH = 80:20, flow rate = 1.0 mL/min, λ = 254 nm, t_R = 10.736 min (minor), t_R = 20.179 min (major).
HRMS (ESI) *m/z*: [M + Na]$^+$ calcd. for $C_{26}H_{23}NNaO_2S$ 436.1342, found 436.1334.

(*R*,*E*)-1,3-diphenylprop-2-en-1-ol (44)

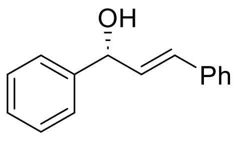

44

Colorless oil, R_f = 0.6 (petroleum ether/ethyl acetate = 5/1), 41.6 mg (99% yield), 99.6:0.4 e.r.
[α]$_D$^{20} = + 25.5 (c = 0.78, $CHCl_3$).
^{1}**H NMR (400 MHz, CDCl$_3$) δ** 7.56 – 7.20 (m, 10H), 6.73 (d, *J* = 15.8 Hz, 1H), 6.43 (dd, *J* = 15.9, 6.4 Hz, 1H), 5.41 (d, *J* = 6.5 Hz, 1H), 2.30 (s, 1H).
^{13}**C NMR (101 MHz, CDCl$_3$) δ** 142.8, 136.6, 131.6, 130.6, 128.7, 128.6, 127.8, 126.7, 126.4, 75.1.
HRMS (ESI) *m/z*: [M - OH]$^+$ calcd. for $C_{15}H_{13}$ 193.1012, found 193.1012.
HPLC (ChiralPak OD-H column), hexane/*i*-PrOH = 90:10, flow rate = 1.0 mL/min, λ = 254 nm, t_R = 20.535 min (major), t_R = 16.335 min (minor).

(*R*,*E*)-3-phenyl-1-(*p*-tolyl)prop-2-en-1-ol (45)

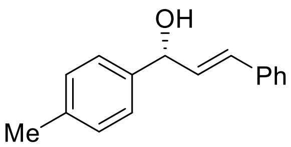

45

White soild, R_f = 0.6 (petroleum ether/ethyl acetate = 5/1), 44.4 mg (99% yield), 99.9:0.1 e.r.

[α]$_D^{20}$ = + 25.3 (c = 0.79, EtOH).

^{1}H NMR (400 MHz, CDCl$_3$) δ 7.48 – 7.18 (m, 9H), 6.72 (d, *J* = 15.8 Hz, 1H), 6.42 (dd, *J* = 15.9, 6.4 Hz, 1H), 5.39 (d, *J* = 6.4 Hz, 1H), 2.40 (s, 3H), 2.21 (s, 1H).

^{13}C NMR (101 MHz, CDCl$_3$) δ 139.9, 137.6, 136.7, 131.7, 130.3, 129.4, 128.6, 127.8, 126.7, 126.4, 75.0, 21.2.

HRMS (ESI) *m/z*: [M - OH]$^+$ calcd. for $C_{16}H_{15}$ 207.1168, found 207.1169.

HPLC (ChiralPak OD-H column), hexane/*i*-PrOH = 80:20, flow rate = 1.0 mL/min, λ = 254 nm, t_R = 11.163 min (major), t_R = 8.582 min (minor).

(*R,E*)-1-(4-fluorophenyl)-3-phenylprop-2-en-1-ol (46)

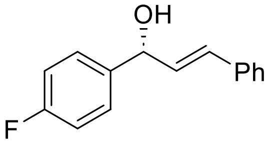

46

Colorless oil, R_f = 0.4 (petroleum ether/ethyl acetate = 5/1), 45.1 mg (99% yield), 99:1 e.r..

[α]$_D^{20}$ = + 19.7 (c = 0.91, EtOH).

^{1}H NMR (400 MHz, CDCl$_3$) δ 7.52 – 7.25 (m, 7H), 7.08 (t, *J* = 8.6 Hz, 2H), 6.69 (d, *J* = 15.8 Hz, 1H), 6.37 (dd, *J* = 15.9, 6.5 Hz, 1H), 5.38 (d, *J* = 6.5 Hz, 1H), 2.37 (s, 1H).

^{13}C NMR (101 MHz, CDCl$_3$) δ 162.5 (d, *J* = 247.4 Hz), 138.5 (d, *J* = 3.0 Hz), 136.4, 131.3, 130.8, 128.7, 128.1, 128.0 (d, *J* = 8.1 Hz), 128.0, 126.7, 115.5 (d, *J* = 21.2 Hz), 74.5.

^{19}F NMR (376 MHz, CDCl$_3$) δ -114.6 (s, 1F).

HRMS (ESI) *m/z*: [M - OH]$^+$ calcd. for $C_{15}H_{12}F$ 211.0918, found 211.0918.

HPLC (ChiralPak OD-H column), hexane/*i*-PrOH = 80:20, flow rate = 1.0 mL/min, λ = 254 nm, t_R = 11.185 min (major), t_R = 8.284 min (minor).

(*R,E*)-1-(4-chlorophenyl)-3-phenylprop-2-en-1-ol (47)

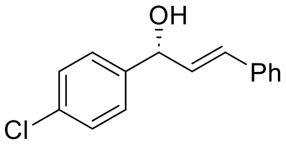

47

White soild, R_f = 0.4 (petroleum ether/ethyl acetate = 5/1), 46.4 mg (95% yield), 99.8:0.2 e.r.

[α]$_D^{20}$ = + 15.2 (c = 0.82, EtOH).

^{1}H NMR (400 MHz, CDCl$_3$) δ 7.50 – 7.20 (m, 9H), 6.69 (d, *J* = 15.8 Hz, 1H), 6.35 (dd, *J* = 15.8, 6.5 Hz, 1H), 5.37 (d, *J* = 6.6 Hz, 1H), 2.32 (s, 1H).

^{13}C NMR (101 MHz, CDCl$_3$) δ 141.2, 136.3, 133.5, 131.1, 131.0, 128.8, 128.7, 128.0, 127.8, 126.7, 74.5.

HRMS (ESI) *m/z*: [M - OH]$^+$ calcd. for $C_{15}H_{12}Cl$ 227.0622, found 227.0622.

HPLC (ChiralPak OD-H column), hexane/*i*-PrOH = 80:20, flow rate = 1.0 mL/min, λ = 254 nm, t_R = 12.922 min (major), t_R = 9.308 min (minor).

(*R,E*)-1-(4-bromophenyl)-3-phenylprop-2-en-1-ol (48)

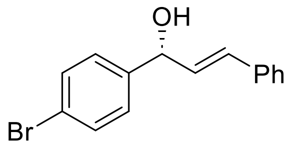

48

White soild, R_f = 0.4 (petroleum ether/ethyl acetate = 5/1), 44.1 mg (77% yield), 99:1 e.r..

[α]$_D$^{20} = + 17.1 (c = 0.76, EtOH).

^{1}H NMR (400 MHz, CDCl$_3$) δ 7.52 (d, *J* = 8.0 Hz, 2H), 7.42 – 7.28 (m, 7H), 6.69 (d, *J* = 15.8 Hz, 1H), 6.34 (dd, *J* = 15.9, 6.7 Hz, 1H), 5.36 (d, *J* = 6.6 Hz, 1H), 2.27 (s, 1H).

^{13}C NMR (101 MHz, CDCl$_3$) δ 141.7, 136.2, 131.7, 131.1, 131.0, 128.7, 128.1, 128.0, 126.7, 121.6, 74.6.

HRMS (ESI) *m/z*: [M - OH]$^+$ calcd. for $C_{15}H_{12}Br$ 271.0117, found 271.0117.

HPLC (ChiralPak OD-H column), hexane/*i*-PrOH = 80:20, flow rate = 1.0 mL/min, λ = 254 nm, t_R = 13.362 min (major), t_R = 9.595 min (minor).

(*R,E*)-1-(4-iodophenyl)-3-phenylprop-2-en-1-ol (49)

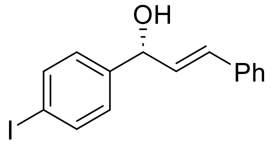

49

White soild, R_f = 0.4 (petroleum ether/ethyl acetate = 5/1), 49.0 mg (73% yield), 99.7:0.3 e.r..

[α]$_D$^{20} = + 15.5 (c = 0.58, EtOH).

^{1}H NMR (400 MHz, CDCl$_3$) δ 7.72 (d, *J* = 7.8 Hz, 2H), 7.40 (d, *J* = 6.8 Hz, 2H), 7.34 (t, *J* = 7.4 Hz, 2H), 7.29 (d, *J* = 5.4 Hz, 1H), 7.21 (d, *J* = 8.0 Hz, 2H), 6.69 (d, *J* = 15.8 Hz, 1H), 6.34 (dd, *J* = 15.9, 6.7 Hz, 1H), 5.35 (d, *J* = 6.6 Hz, 1H), 2.14 (s, 1H).

^{13}C NMR (101 MHz, CDCl$_3$) δ 142.4, 137.7, 136.2, 131.2, 131.0, 128.7, 128.3, 128.0, 126.7, 93.3.

HRMS (ESI) *m/z*: [M - OH]$^+$ calcd. for $C_{15}H_{12}I$ 318.9978, found 318.9978.

HPLC (ChiralPak OD-H column), hexane/*i*-PrOH = 80:20, flow rate = 1.0 mL/min, λ = 254 nm, t_R = 14.785 min (major), t_R = 10.604 min (minor).

(*R,E*)-1-(4-methoxyphenyl)-3-phenylprop-2-en-1-ol (50)

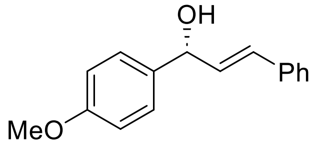

50

White soild, R_f = 0.5 (petroleum ether/ethyl acetate = 5/1), 43.9 mg (91% yield), 99:1 e.r..

[α]$_D$^{20} = + 27.5 (c = 0.85, EtOH).

^{1}H NMR (400 MHz, CDCl$_3$) δ 7.45 – 7.23 (m, 7H), 6.94 (d, *J* = 8.2 Hz, 2H), 6.70 (d, *J* = 15.9 Hz, 1H), 6.42 (dd, *J* = 15.9, 6.3 Hz, 1H), 5.37 (d, *J* = 6.2 Hz, 1H), 3.84 (s, 3H), 2.22 (s, 1H).

^{13}C NMR (101 MHz, CDCl$_3$) δ 159.3, 136.7, 135.1, 131.8, 130.2, 128.6, 127.8, 126.6, 114.0, 74.7, 55.4.

HRMS (ESI) *m/z*: [M - OH]$^+$ calcd. for $C_{16}H_{15}O$ 223.1117, found 223.1116.

HPLC (ChiralPak OD-H column), hexane/*i*-PrOH = 80:20, flow rate = 1.0 mL/min, λ = 254 nm, t_R = 15.374 min (major), t_R = 11.866 min (minor).

(*R,E*)-1-(3-methoxyphenyl)-3-phenylprop-2-en-1-ol (51)

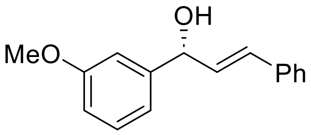

51

White soild, R_f = 0.4 (petroleum ether/ethyl acetate = 5/1), 47.5 mg (99% yield), 99.7:0.3 e.r..
$[\alpha]_D^{20}$ = + 7.4 (c = 0.96, EtOH).
^{1}H NMR (400 MHz, $CDCl_3$) δ 7.48 – 7.23 (m, 6H), 7.04 (d, *J* = 6.5 Hz, 2H), 6.88 (d, *J* = 8.3 Hz, 1H), 6.71 (d, *J* = 15.8 Hz, 1H), 6.40 (dd, *J* = 15.9, 6.4 Hz, 1H), 5.38 (d, *J* = 6.1 Hz, 1H), 3.84 (s, 3H), 2.33 (s, 1H).
^{13}C NMR (101 MHz, $CDCl_3$) δ 159.9, 144.5, 136.5, 131.4, 130.6, 129.7, 128.6, 127.8, 126.7, 118.7, 113.4, 111.78, 75.1, 55.3.
HRMS (ESI) *m/z*: [M - OH]$^+$ calcd. for $C_{16}H_{15}O$ 223.1117, found 223.1117.
HPLC (ChiralPak OD-H column), hexane/*i*-PrOH = 80:20, flow rate = 1.0 mL/min, λ = 254 nm, t_R = 15.562 min (major), t_R = 11.736 min (minor).

(*R,E*)-1-(2-methoxyphenyl)-3-phenylprop-2-en-1-ol (52)

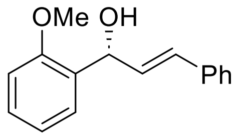

52

White soild, R_f = 0.6 (petroleum ether/ethyl acetate = 5/1), 29.1 mg (61% yield), 98:2 e.r..
$[\alpha]_D^{20}$ = + 85.6 (c = 0.51, EtOH).
^{1}H NMR (400 MHz, $CDCl_3$) δ 7.49 – 7.24 (m, 7H), 7.01 (t, *J* = 7.6 Hz, 1H), 6.95 (d, *J* = 8.2 Hz, 1H), 6.69 (d, *J* = 15.8 Hz, 1H), 6.52 (dd, *J* = 15.7, 5.8 Hz, 1H), 5.62 (s, 1H), 3.91 (s, 3H), 2.97 (s, 1H).
^{13}C NMR (101 MHz, $CDCl_3$) δ 156.8, 137.0, 130.9, 130.0, 128.9, 128.5, 127.6, 127.5, 126.6, 121.0, 110.8, 71.6, 55.5.
HRMS (ESI) *m/z*: [M - OH]$^+$ calcd. for $C_{16}H_{15}O$ 223.1117, found 223.1116.
HPLC (ChiralPak AD-H column), hexane/*i*-PrOH = 90:10, flow rate = 1.0 mL/min, λ = 254 nm, t_R = 15.721 min (major), t_R = 13.365 min (minor).

(*R,E*)-3-phenyl-1-(4-(trifluoromethoxy)phenyl)prop-2-en-1-ol (53)

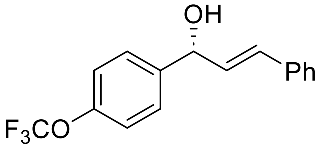

53

Colorless oil, R_f = 0.5 (petroleum ether/ethyl acetate = 5/1), 57.9 mg (98% yield), 99.9:0.1 e.r..

$[\alpha]_D^{20}$ = + 10.9 (c = 1.13, EtOH).
^{1}H NMR (400 MHz, $CDCl_3$) δ 7.48 (d, *J* = 8.2 Hz, 2H), 7.42 (d, *J* = 7.6 Hz, 2H), 7.39 – 7.33 (m, 2H), 7.30 (d, *J* = 7.1 Hz, 1H), 7.25 (d, *J* = 8.3 Hz, 2H), 6.72 (d, *J* = 15.8 Hz, 1H), 6.36 (dd, *J* = 15.8, 6.8 Hz, 1H), 5.41 (d, *J* = 6.7 Hz, 1H), 2.39 (s, 1H).
^{13}C NMR (101 MHz, $CDCl_3$) δ 148.7, 141.4, 136.2, 131.2, 131.0, 128.7, 128.1, 127.8, 126.7, 121.1, 120.5 (q, *J* = 257.6 Hz), 74.4.
^{19}F NMR (376 MHz, $CDCl_3$) δ -57.8 (s, 3F).
HRMS (ESI) *m/z*: $[M - OH]^+$ calcd. for $C_{16}H_{12}F_3O$ 277.0835, found 277.0835.
HPLC (ChiralPak OD-H column), hexane/*i*-PrOH = 80:20, flow rate = 1.0 mL/min, λ = 254 nm, t_R = 11.164 min (major), t_R = 7.565 min (minor).

(*R,E*)-1-(4-(methylthio)phenyl)-3-phenylprop-2-en-1-ol (54)

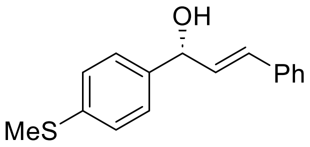

54

White soild, R_f = 0.3 (petroleum ether/ethyl acetate = 5/1), 48.3 mg (94% yield), 99:1 e.r..
$[\alpha]_D^{20}$ = + 26.2 (c = 0.95, EtOH).
^{1}H NMR (400 MHz, $CDCl_3$) δ 7.45 – 7.24 (m, 9H), 6.69 (d, *J* = 15.8 Hz, 1H), 6.38 (dd, *J* = 15.9, 6.5 Hz, 1H), 5.36 (d, *J* = 6.2 Hz, 1H), 2.51 (s, 3H), 2.34 (s, 1H).
^{13}C NMR (101 MHz, $CDCl_3$) δ 139.7, 138.0, 136.5, 131.4, 130.6, 128.6, 127.9, 127.0, 126.8, 126.7, 74.7, 15.9.
HRMS (ESI) *m/z*: $[M - OH]^+$ calcd. for $C_{16}H_{15}S$ 239.0889, found 239.0889.
HPLC (ChiralPak OD-H column), hexane/*i*-PrOH = 80:20, flow rate = 1.0 mL/min, λ = 254 nm, t_R = 15.945 min (major), t_R = 11.464 min (minor).

(*R,E*)-3-phenyl-1-(4-(4,4,5,5-tetramethyl-1,3,2-dioxaborolan-2-yl)phenyl)prop-2-en-1-ol (55)

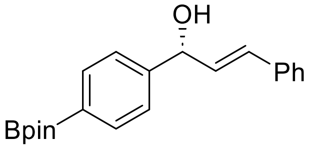

55

White soild, R_f = 0.6 (petroleum ether/ethyl acetate = 5/1), 47.0 mg (70% yield), 99.7:0.3 e.r.
$[\alpha]_D^{20}$ = + 14.7 (c = 0.71, EtOH).
^{1}H NMR (400 MHz, $CDCl_3$) δ 7.85 (d, *J* = 7.5 Hz, 2H), 7.47 (d, *J* = 7.5 Hz, 2H), 7.40 (d, *J* = 6.8 Hz, 2H), 7.36 – 7.30 (m, 2H), 7.27 (d, *J* = 9.5 Hz, 1H), 6.70 (d, *J* = 15.9 Hz, 1H), 6.39 (dd, *J* = 15.9, 6.5 Hz, 1H), 5.43 (d, *J* = 6.5 Hz, 1H), 2.14 (s, 1H), 1.37 (s, 12H).
^{13}C NMR (101 MHz, $CDCl_3$) δ 145.9, 136.5, 135.2, 131.4, 130.8, 128.6, 127.8, 126.7, 125.6, 83.9, 74.2, 24.9.
HRMS (ESI) *m/z*: $[M - OH]^+$ calcd. for $C_{21}H_{24}BO_2$ 319.1864, found 319.1865.
HPLC (ChiralPak OD-H column), hexane/*i*-PrOH = 80:20, flow rate = 1.0 mL/min, λ = 254 nm, t_R = 10.749 min (major), t_R = 7.471 min (minor).

(*R,E*)-3-phenyl-1-(4-(trifluoromethyl)phenyl)prop-2-en-1-ol (56)

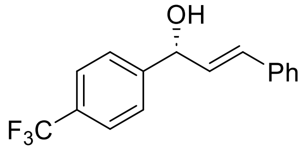

56

Colorless oil, R_f = 0.5 (petroleum ether/ethyl acetate = 5/1), 44.5 mg (80% yield), 99:1 e.r.

[α]$_D^{20}$ = + 6.4 (c = 0.86, EtOH).

^{1}H NMR (400 MHz, CDCl$_3$) δ 7.66 (d, *J* = 8.0 Hz, 2H), 7.58 (d, *J* = 8.0 Hz, 2H), 7.45 – 7.28 (m, 5H), 6.73 (d, *J* = 15.8 Hz, 1H), 6.36 (dd, *J* = 15.9, 6.9 Hz, 1H), 5.46 (d, *J* = 6.8 Hz, 1H), 2.35 (s, 1H).

^{13}C NMR (101 MHz, CDCl$_3$) δ 146.6, 136.1, 131.6, 130.7, 129.9 (q, *J* = 32.3 Hz), 128.7, 128.2, 126.7, 126.6, 125.6 (q, *J* = 3.7 Hz), 122.46 (q, *J* = 272.7 Hz), 74.6.

^{19}F NMR (376 MHz, CDCl$_3$) δ -62.4 (s, 3F).

HRMS (ESI) *m/z*: [M - OH]$^+$ calcd. for $C_{16}H_{12}F_3$ 261.0886, found 261.0885.

HPLC (ChiralPak OD-H column), hexane/*i*-PrOH = 80:20, flow rate = 1.0 mL/min, λ = 254 nm, t_R = 11.821 min (major), t_R = 8.234 min (minor).

methyl (*R,E*)-4-(1-hydroxy-3-phenylallyl)benzoate (57)

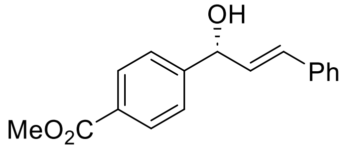

57

Colorless oil, R_f = 0.2 (petroleum ether/ethyl acetate = 5/1), 41.8 mg (78% yield), 99.7:0.3 e.r.

[α]$_D^{20}$ = + 4.8 (c = 0.58, EtOH).

^{1}H NMR (400 MHz, CDCl$_3$) δ 8.05 (d, *J* = 8.5 Hz, 2H), 7.52 (d, *J* = 8.0 Hz, 2H), 7.43 – 7.26 (m, 5H), 6.71 (d, *J* = 15.8 Hz, 1H), 6.36 (dd, *J* = 15.8, 6.8 Hz, 1H), 5.45 (d, *J* = 6.7 Hz, 1H), 3.93 (s, 3H), 2.41 (s, 1H).

^{13}C NMR (101 MHz, CDCl$_3$) δ 167.0, 147.8, 136.2, 131.4, 130.8, 129.9, 129.4, 128.6, 128.1, 126.7, 126.2, 74.8, 52.2.

HRMS (ESI) *m/z*: [M - OH]$^+$ calcd. for $C_{17}H_{15}O_2$ 251.1067, found 251.1066.

HPLC (ChiralPak OD-H column), hexane/*i*-PrOH = 80:20, flow rate = 1.0 mL/min, λ = 254 nm, t_R = 18.296 min (major), t_R = 14.740 min (minor).

(*R,E*)-4-(1-hydroxy-3-phenylallyl)benzonitrile (58)

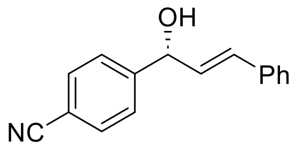

58

White soild, R_f = 0.3 (petroleum ether/ethyl acetate = 3/1), 33.4 mg (71% yield), 99.7:0.3 e.r.

[α]$_D^{20}$ = + 7.0 (c = 0.64, EtOH).

^{1}H NMR (400 MHz, CDCl$_3$) δ 7.67 (d, J = 7.9 Hz, 2H), 7.57 (d, J = 7.8 Hz, 2H), 7.43 – 7.27 (m, 5H), 6.72 (d, J = 15.8 Hz, 1H), 6.31 (dd, J = 15.9, 7.0 Hz, 1H), 5.45 (d, J = 7.0 Hz, 1H), 2.41 (s, 1H).
^{13}C NMR (101 MHz, CDCl$_3$) δ 147.9, 135.9, 132.4, 132.0, 130.3, 128.7, 128.3, 126.9, 126.7, 118.9, 111.3, 74.5.
HRMS (ESI) *m/z*: [M - OH]$^+$ calcd. for $C_{16}H_{12}N$ 218.0964, found 218.0964.
HPLC (ChiralPak OD-H column), hexane/*i*-PrOH = 80:20, flow rate = 1.0 mL/min, λ = 254 nm, t_R = 16.711 min (major), t_R = 14.179 min (minor).

(*R,E*)-1-(naphthalen-1-yl)-3-phenylprop-2-en-1-ol (59)

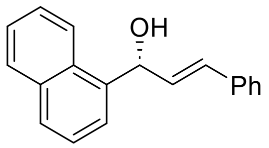

59

Colorless oil, R_f = 0.4 (petroleum ether/ethyl acetate = 5/1), 39.4 mg (76% yield), 99:1 e.r.
$[\alpha]_D^{20}$ = + 126.3 (c = 0.89, EtOH).
^{1}H NMR (400 MHz, CDCl$_3$) δ 8.26 (d, J = 6.8 Hz, 1H), 7.93 (d, J = 9.3 Hz, 1H), 7.86 (d, J = 8.3 Hz, 1H), 7.73 (d, J = 7.1 Hz, 1H), 7.62 – 7.49 (m, 3H), 7.42 (d, J = 7.5 Hz, 2H), 7.37 – 7.26 (m, 3H), 6.82 (d, J = 15.9 Hz, 1H), 6.62 (dd, J = 16.0, 5.8 Hz, 1H), 6.13 (d, J = 5.9 Hz, 1H), 2.38 (s, 1H).
^{13}C NMR (101 MHz, CDCl$_3$) δ 138.4, 136.6, 134.0, 131.1, 131.0, 130.7, 128.9, 128.6, 127.8, 126.7, 126.3, 125.8, 125.5, 124.1, 123.8, 72.2.
HRMS (ESI) *m/z*: [M - OH]$^+$ calcd. for $C_{19}H_{15}$ 243.1168, found 243.1166.
HPLC (ChiralPak OD-H column), hexane/*i*-PrOH = 70:30, flow rate = 1.0 mL/min, λ = 254 nm, t_R = 15.733 min (major), t_R = 9.842 min (minor).

(*R,E*)-3-phenyl-1-(thiophen-2-yl)prop-2-en-1-ol (60)

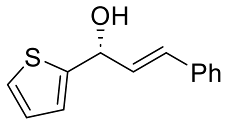

60

Colorless oil, R_f = 0.4 (petroleum ether/ethyl acetate = 5/1), 40.4 mg (94% yield), 99.7:0.3 e.r.
$[\alpha]_D^{20}$ = + 1.2 (c = 0.74, EtOH).
^{1}H NMR (400 MHz, CDCl$_3$) δ 7.45 (d, J = 7.4 Hz, 2H), 7.38 – 7.28 (m, 4H), 7.14 – 6.97 (m, 2H), 6.76 (d, J = 15.8 Hz, 1H), 6.48 (dd, J = 15.9, 6.4 Hz, 1H), 5.65 (d, J = 6.6 Hz, 1H), 2.43 (s, 1H).
^{13}C NMR (101 MHz, CDCl$_3$) δ 147.0, 136.3, 131.2, 130.6, 128.7, 128.0, 126.9, 126.8, 125.4, 124.4, 71.0.
HRMS (ESI) *m/z*: [M - OH]$^+$ calcd. for $C_{13}H_{11}S$ 199.0576, found 199.0575.
HPLC (ChiralPak OD-H column), hexane/*i*-PrOH = 80:20, flow rate = 1.0 mL/min, λ = 254 nm, t_R = 11.436 min (major), t_R = 8.817 min (minor).

(*R,E*)-1-(furan-2-yl)-3-phenylprop-2-en-1-ol (61)

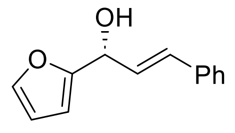

61

Brown oil, R_f = 0.5 (petroleum ether/ethyl acetate = 4/1), 30.2 mg (76% yield), 99:1 e.r.
[α]$_D$^{20} = + 14.1 (c = 0.55, EtOH).
^{1}H NMR (400 MHz, CDCl$_3$) δ 7.45 (d, J = 5.9 Hz, 3H), 7.36 (t, J = 7.5 Hz, 2H), 7.30 (d, J = 8.1 Hz, 1H), 6.77 (d, J = 15.9 Hz, 1H), 6.49 (dd, J = 15.9, 6.2 Hz, 1H), 6.36 (d, J = 18.0 Hz, 2H), 5.43 (d, J = 6.3 Hz, 1H), 2.31 (s, 1H).
^{13}C NMR (101 MHz, CDCl$_3$) δ 155.1, 142.5, 136.4, 131.9, 128.6, 128.0, 128.0, 126.7, 110.4, 106.8, 68.6.
HRMS (ESI) *m/z*: [M - OH]$^+$ calcd. for $C_{13}H_{11}O$ 183.0804, found 183.0805.
HPLC (ChiralPak OD-H column), hexane/*i*-PrOH = 80:20, flow rate = 1.0 mL/min, λ = 254 nm, t_R = 11.140 min (major), t_R = 8.335 min (minor).

***tert*-butyl (*R,E)*-6-(1-hydroxy-3-phenylallyl)-1*H*-indole-1-carboxylate (62)**

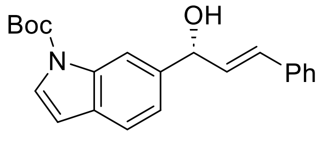

62

White soild, R_f = 0.5 (petroleum ether/ethyl acetate = 4/1), 51.8 mg (74% yield), 99:1 e.r.
[α]$_D$^{20} = + 11.7 (c = 0.82, EtOH).
^{1}H NMR (400 MHz, DMSO-d_6) δ 8.20 (s, 1H), 7.64 (d, J = 3.8 Hz, 1H), 7.58 (d, J = 8.1 Hz, 1H), 7.43 (d, J = 7.6 Hz, 2H), 7.31 (t, J = 6.8 Hz, 3H), 7.21 (t, J = 7.3 Hz, 1H), 6.74 – 6.64 (m, 2H), 6.45 (dd, J = 15.9, 6.3 Hz, 1H), 5.73 (d, J = 5.1 Hz, 1H), 5.40 (t, J = 5.3 Hz, 1H), 1.59 (s, 9H).
^{13}C NMR (101 MHz, DMSO-d_6) δ 149.6, 141.4, 137.2, 135.2, 134.2, 129.6, 129.1, 128.5, 127.8, 126.8, 126.5, 122.0, 121.1, 113.0, 107.8, 84.1, 74.0, 28.1.
HRMS (ESI) *m/z*: [M - OH]$^+$ calcd. for $C_{22}H_{22}NO_2$ 332.1645, found 332.1646.
HPLC (ChiralPak AD-H column), hexane/*i*-PrOH = 85:15, flow rate = 1.0 mL/min, λ = 254 nm, t_R = 10.035 min (major), t_R = 9.288 min (minor).

(*R,E*)-1-(6-methoxypyridin-3-yl)-3-phenylprop-2-en-1-ol (63)

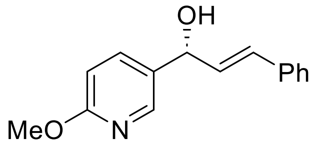

63

Colorless oil, R_f = 0.5 (petroleum ether/ethyl acetate = 2/1), 44.8 mg (93% yield), 99:1 e.r.
[α]$_D$^{20} = + 33.4 (c = 0.86, EtOH).
^{1}H NMR (400 MHz, Chloroform-*d*) δ 8.18 (d, J = 2.5 Hz, 1H), 7.66 (dd, J = 8.6, 2.5 Hz, 1H), 7.39 (d, J = 6.9 Hz, 2H), 7.33 (t, J = 7.3 Hz, 2H), 7.28 – 7.25 (m, 1H), 6.76 (d, J = 8.6 Hz, 1H), 6.67 (d, J = 14.6 Hz, 1H), 6.36 (dd, J = 15.8, 6.4 Hz, 1H), 5.35 (d, J = 6.4 Hz, 1H), 3.94 (s, 3H), 2.84 (s,

1H).

^{13}C NMR (101 MHz, $CDCl_3$) δ 163.9, 145.0, 137.4, 136.3, 131.2, 131.0, 130.8, 128.6, 128.0, 126.6, 111.0, 72.5, 53.6.

HRMS (ESI) *m/z*: $[M - OH]^+$ calcd. for $C_{15}H_{14}NO$ 224.1070, found 224.1074.

HPLC (ChiralPak OD-H column), hexane/*i*-PrOH = 80:20, flow rate = 1.0 mL/min, λ = 254 nm, t_R = 13.025 min (major), t_R = 9.995 min (minor).

(*R,E*)-3-(4-methoxyphenyl)-1-phenylprop-2-en-1-ol (64)

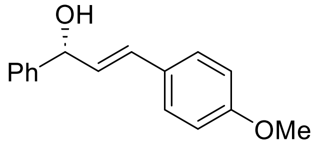

64

Colorless oil, R_f = 0.2 (petroleum ether/ethyl acetate = 5/1), 46.9 mg (98% yield), 99.6:0.4 e.r.

$[\alpha]_D^{20}$ = + 19.1 (c = 0.89, EtOH).

^{1}H NMR (400 MHz, $CDCl_3$) δ 7.55 – 7.30 (m, 7H), 6.88 (d, *J* = 8.8 Hz, 2H), 6.65 (d, *J* = 15.8 Hz, 1H), 6.28 (dd, *J* = 15.8, 6.7 Hz, 1H), 5.38 (d, *J* = 6.7 Hz, 1H), 3.83 (s, 3H), 2.34 (s, 1H).

^{13}C NMR (101 MHz, $CDCl_3$) δ 159.4, 143.1, 130.2, 129.5, 129.3, 128.6, 127.9, 127.7, 126.4, 114.0, 75.3, 55.3.

HRMS (ESI) *m/z*: $[M - OH]^+$ calcd. for $C_{16}H_{15}O$ 223.1117, found 223.1116.

HPLC (ChiralPak OD-H column), hexane/*i*-PrOH = 90:10, flow rate = 1.0 mL/min, λ = 254 nm, t_R = 17.860 min (major), t_R = 20.644 min (minor).

(*R,E*)-1-phenyl-3-(m-tolyl)prop-2-en-1-ol (65)

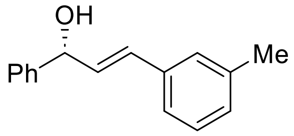

65

Colorless oil, R_f = 0.3 (petroleum ether/ethyl acetate = 5/1), 44.4 mg (99% yield), 99.6:0.4 e.r.

$[\alpha]_D^{20}$ = + 22.2 (c = 0.87, EtOH).

^{1}H NMR (400 MHz, $CDCl_3$) δ 7.53 – 7.38 (m, 4H), 7.35 (t, *J* = 7.1 Hz, 1H), 7.25 (d, *J* = 6.3 Hz, 3H), 7.11 (d, *J* = 7.9 Hz, 1H), 6.70 (d, *J* = 15.8 Hz, 1H), 6.42 (dd, *J* = 15.8, 6.6 Hz, 1H), 5.41 (d, *J* = 6.5 Hz, 1H), 2.38 (s, 3H), 2.32 (s, 1H).

^{13}C NMR (101 MHz, $CDCl_3$) δ 142.9, 138.2, 136.5, 131.4, 130.7, 128.7, 128.5, 127.8, 127.4, 126.4, 123.9, 75.2, 21.4.

HRMS (ESI) *m/z*: $[M - OH]^+$ calcd. for $C_{16}H_{15}$ 207.1168, found 207.1167.

HPLC (ChiralPak OD-H column), hexane/*i*-PrOH = 90:10, flow rate = 1.0 mL/min, λ = 254 nm, t_R = 15.877 min (major), t_R = 12.420 min (minor).

(*R,E*)-1-phenyl-3-(*o*-tolyl)prop-2-en-1-ol (66)

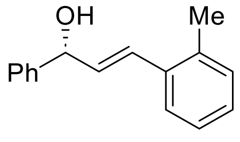

66

Colorless oil, R_f = 0.4 (petroleum ether/ethyl acetate = 5/1), 32.7 mg (73% yield), 99:1 e.r.

[α]$_D$^{20} = + 15.4 (c = 0.63, EtOH).

^{1}H NMR (400 MHz, CDCl$_3$) δ 7.56 – 7.38 (m, 5H), 7.35 (d, *J* = 7.1 Hz, 1H), 7.19 (d, *J* = 3.2 Hz, 3H), 6.96 (d, *J* = 15.6 Hz, 1H), 6.32 (dd, *J* = 15.7, 6.5 Hz, 1H), 5.44 (d, *J* = 6.5 Hz, 1H), 2.41 (s, 3H), 2.23 (s, 1H).

^{13}C NMR (101 MHz, CDCl$_3$) δ 142.9, 135.7, 135.7, 132.9, 130.4, 128.7, 128.4, 127.8, 127.7, 126.4, 126.1, 125.8, 75.4, 19.9.

HRMS (ESI) *m/z*: [M - OH]$^+$ calcd. for $C_{16}H_{15}$ 207.1168, found 207.1169.

HPLC (ChiralPak OD-H column), hexane/*i*-PrOH = 90:10, flow rate = 1.0 mL/min, λ = 254 nm, t_R = 20.326 min (major), t_R = 16.400 min (minor).

(*R,E*)-3-(4-fluorophenyl)-1-phenylprop-2-en-1-ol (67)

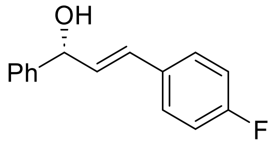

67

Colorless oil, R_f = 0.3 (petroleum ether/ethyl acetate = 5/1), 43.6 mg (96% yield), 99.9:0.1 e.r.

[α]$_D$^{20} = + 19.7 (c = 0.83, EtOH).

^{1}H NMR (400 MHz, CDCl$_3$) δ 7.54 – 7.30 (m, 7H), 7.03 (t, *J* = 8.6 Hz, 2H), 6.67 (d, *J* = 15.8 Hz, 1H), 6.33 (dd, *J* = 15.9, 6.4 Hz, 1H), 5.39 (d, *J* = 6.4 Hz, 1H), 2.33 (s, 1H).

^{13}C NMR (101 MHz, CDCl$_3$) δ 162.4 (d, *J* = 247.4 Hz), 142.8, 132.7 (d, *J* = 4.0 Hz), 131.3 (d, *J* =2.0 Hz), 129.4, 128.7, 128. 2, 128.1, 127.9, 126.4, 115.5 (d, *J* = 22.2 Hz), 75.1.

^{19}F NMR (376 MHz, CDCl$_3$) δ -114.1 (s, 1F).

HRMS (ESI) *m/z*: [M - OH]$^+$ calcd. for $C_{15}H_{12}F$ 211.0918, found 211.0918.

HPLC (ChiralPak OD-H column), hexane/*i*-PrOH = 90:10, flow rate = 1.0 mL/min, λ = 254 nm, t_R = 10.630 min (major), t_R = 13.786 min (minor).

(*R,E*)-3-(4-chlorophenyl)-1-phenylprop-2-en-1-ol (68)

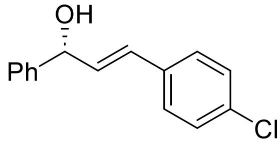

68

Colorless oil, R_f = 0.4 (petroleum ether/ethyl acetate = 5/1), 34.5 mg (71% yield), 99.7:0.3 e.r.

[α]$_D$^{20} = + 26.1 (c = 0.66, EtOH).

^{1}H NMR (400 MHz, CDCl$_3$) δ 7.48 – 7.28 (m, 9H), 6.66 (d, *J* = 15.8 Hz, 1H), 6.38 (dd, *J* = 15.7, 6.2 Hz, 1H), 5.40 (d, *J* = 6.3 Hz, 1H), 2.27 (s, 1H).

^{13}C NMR (101 MHz, CDCl$_3$) δ 142.6, 135.1, 133.4, 132.2, 129.2, 128.8, 128.7, 128.0, 127.8, 126.4,

75.0.

HRMS (ESI) *m/z*: $[M - OH]^+$ calcd. for $C_{15}H_{12}Cl$ 227.0622, found 227.0621.

HPLC (ChiralPak OD-H column), hexane/*i*-PrOH = 90:10, flow rate = 1.0 mL/min, λ = 254 nm, t_R = 11.124 min (major), t_R = 14.391 min (minor).

(*R*,*E*)-3-(4-bromophenyl)-1-phenylprop-2-en-1-ol (69)

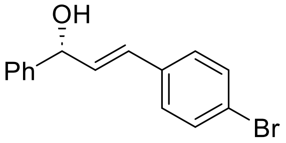

69

White soild, R_f = 0.5 (petroleum ether/ethyl acetate = 4/1), 37.6 mg (65% yield), 99.6:0.4 e.r.

$[\alpha]_D^{20}$ = + 24.2 (c = 0.71, EtOH).

^{1}H NMR (400 MHz, Chloroform-*d*) δ 7.49 – 7.24 (m, 9H), 6.65 (d, *J* = 16.0 Hz, 1H), 6.40 (dd, *J* = 15.8, 6.3 Hz, 1H), 5.39 (d, *J* = 6.3 Hz, 1H), 2.23 (s, 1H).

^{13}C NMR (101 MHz, $CDCl_3$) δ 142.6, 135.5, 132.3, 131.7, 129.2, 128.7, 128.2, 128.0, 126.4, 121.6, 75.1.

HRMS (ESI) *m/z*: $[M - OH]^+$ calcd. for $C_{15}H_{12}Br$ 271.0117, found 271.0120.

HPLC (ChiralPak OD-H column), hexane/*i*-PrOH = 90:10, flow rate = 1.0 mL/min, λ = 254 nm, t_R = 11.967 min (major), t_R = 16.508 min (minor).

(*R*,*E*)-3-(benzo[*d*][1,3]dioxol-5-yl)-1-phenylprop-2-en-1-ol (70)

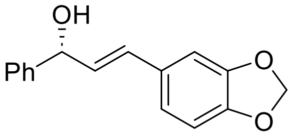

70

Yellow oil, R_f = 0.3 (petroleum ether/ethyl acetate = 5/1), 42.0 mg (83% yield), 99.8:0.2 e.r.

$[\alpha]_D^{20}$ = + 26.8 (c = 0.82, EtOH).

^{1}H NMR (400 MHz, $CDCl_3$) δ 7.49 – 7.36 (m, 4H), 7.33 (t, *J* = 7.2 Hz, 1H), 6.94 (s, 1H), 6.84 (d, *J* = 7.9 Hz, 1H), 6.77 (d, *J* = 8.1 Hz, 1H), 6.61 (d, *J* = 15.7 Hz, 1H), 6.24 (dd, *J* = 15.8, 6.6 Hz, 1H), 5.96 (s, 2H), 5.37 (d, *J* = 6.7 Hz, 1H), 2.26 (s, 1H).

^{13}C NMR (101 MHz, $CDCl_3$) δ 148.0, 147.4, 142.9, 131.0, 130.3, 129.8, 128.6, 127.8, 126.3, 121.4, 108.3, 105.9, 101.1, 75.2.

HRMS (ESI) *m/z*: $[M - OH]^+$ calcd. for $C_{16}H_{13}O_2$ 237.0910, found 237.0911.

HPLC (ChiralPak IC-H column), hexane/*i*-PrOH = 95:5, flow rate = 1.0 mL/min, λ = 254 nm, t_R = 28.276 min (major), t_R = 23.577 min (minor).

(*R*,*E*)-3-(naphthalen-2-yl)-1-phenylprop-2-en-1-ol (71)

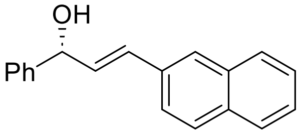

71

White soild, R_f = 0.5 (petroleum ether/ethyl acetate = 5/1), 41.8 mg (80% yield), 99:1 e.r.
[α]$_D^{20}$ = + 4.9 (c = 0.81, EtOH).
^{1}H NMR (400 MHz, CDCl$_3$) δ 7.88 – 7.73 (m, 4H), 7.62 (d, *J* = 8.5 Hz, 1H), 7.57 – 7.32 (m, 7H), 6.88 (d, *J* = 15.8 Hz, 1H), 6.55 (dd, *J* = 15.9, 6.5 Hz, 1H), 5.47 (d, *J* = 6.4 Hz, 1H), 2.36 (s, 1H).
^{13}C NMR (101 MHz, CDCl$_3$) δ 142.8, 134.0, 133.6, 133.1, 132.0, 130.7, 128.7, 128.3, 128.1, 127.9, 127.7, 126.8, 126.4, 126.4, 126.0, 123.7, 75.3.
HRMS (ESI) *m/z*: [M - OH]$^+$ calcd. for $C_{19}H_{15}$ 243.1168, found 243.1168.
HPLC (ChiralPak OD-H column), hexane/*i*-PrOH = 80:20, flow rate = 1.0 mL/min, λ = 254 nm, t_R = 11.029 min (major), t_R = 9.071 min (minor).

(*R, E*)-1-phenyl-3-(4-(trifluoromethyl)phenyl)prop-2-en-1-ol (72)

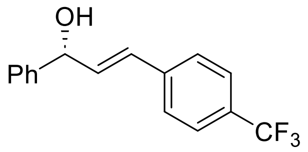

72

White soild, R_f = 0.4 (petroleum ether/ethyl acetate = 5/1), 40.5 mg (73% yield), 99.9:0.1 e.r.
[α]$_D^{20}$ = + 23.3 (c = 0.75, EtOH).
^{1}H NMR (400 MHz, CDCl$_3$) δ 7.59 (d, *J* = 8.1 Hz, 2H), 7.54 – 7.31 (m, 7H), 6.76 (d, *J* = 15.8 Hz, 1H), 6.51 (dd, *J* = 15.9, 6.0 Hz, 1H), 5.43 (d, *J* = 6.1 Hz, 1H), 2.32 (s, 1H).
^{13}C NMR (101 MHz, CDCl$_3$) δ 142.4, 140.1, 134.1, 129.5 (q, *J* = 32.3 Hz), 128.8, 128.8, 128.1, 126.8, 126.4, 125.5 (q, *J* = 3.0 Hz), 124.2 (q, *J* = 272.7 Hz), 74.9.
^{19}F NMR (376 MHz, CDCl$_3$) δ -62.5 (s, 3F).
HRMS (ESI) *m/z*: [M - OH]$^+$ calcd. for $C_{16}H_{12}F_3$ 261.0886, found 261.0885.
HPLC (ChiralPak OD-H column), hexane/*i*-PrOH = 90:10, flow rate = 1.0 mL/min, λ = 254 nm, t_R = 9.332 min (major), t_R = 10.589 min (minor).

(*R, E*)-4-(3-hydroxy-3-phenylprop-1-en-1-yl)benzonitrile (73)

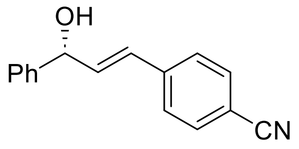

73

Colorless oil, R_f = 0.4 (petroleum ether/ethyl acetate = 5/1), 26.1 mg (56% yield), 99:1 e.r.
[α]$_D^{20}$ = + 34.2 (c = 0.46, EtOH).
^{1}H NMR (400 MHz, CDCl$_3$) δ 7.59 (d, *J* = 8.3 Hz, 2H), 7.51 – 7.38 (m, 6H), 7.38 – 7.31 (m, 1H), 6.74 (d, *J* = 15.9 Hz, 1H), 6.52 (dd, *J* = 15.9, 5.8 Hz, 1H), 5.43 (d, *J* = 5.9 Hz, 1H), 2.38 (s, 1H).
^{13}C NMR (101 MHz, CDCl$_3$) δ 142.2, 141.2, 135.5, 132.4, 128.8, 128.3, 128.2, 127.1, 126.4, 118.9, 110.9, 74.7.
HRMS (ESI) *m/z*: [M - OH]$^+$ calcd. for $C_{16}H_{12}N$ 218.0964, found 218.0964.
HPLC (ChiralPak OD-H column), hexane/*i*-PrOH = 80:20, flow rate = 1.0 mL/min, λ = 254 nm, t_R = 11.835 min (major), t_R = 13.026 min (minor).

(3*S*,8*S*,9*S*,10*R*,13*R*,14*S*,17*R*)-10,13-dimethyl-17-((*R*)-6-methylheptan-2-yl)-2,3,4,7,8,9,10,11,12,

13,14,15,16,17-tetradecahydro-1*H*-cyclopenta[a]phenanthren-3-yl 4-((*R*,*E*)-1-hydroxy-3-phenylallyl)benzoate (74)

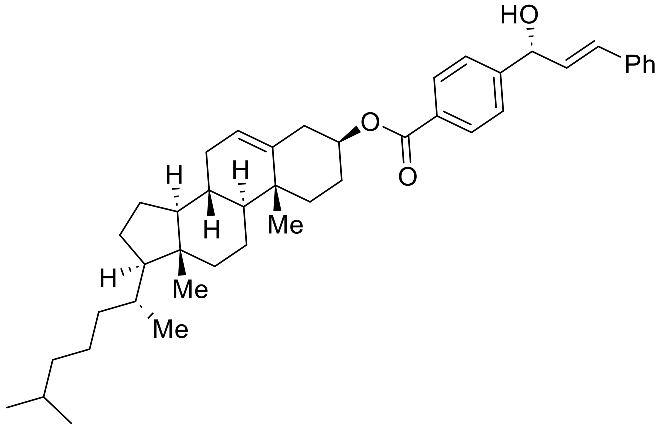

74

Prepared by the general procedure B with aldehyde (0.2 mmol, 1.0 equiv), vinyl chloride **2** (1.5 equiv), $NiBr_2$•glyme (10 mol%), **L11** (5 mol %), NaI (2.0 equiv), $ZnBr_2$ (20 mol%), Zn (3.0 equiv), Et_3N (2.0 equiv) in 2-Me-THF (1.0 mL) under nitrogen at 25 °C for 48 h.

White soild, R_f = 0.5 (petroleum ether/ethyl acetate = 4/1), 77.2 mg (62% yield), >99:1 d.r.

^{1}H NMR (400 MHz, CDCl$_3$) δ 8.06 (d, *J* = 7.9 Hz, 2H), 7.52 (d, *J* = 8.0 Hz, 2H), 7.40 (d, *J* = 7.6 Hz, 2H), 7.33 (t, *J* = 7.4 Hz, 2H), 7.26 (d, *J* = 7.1 Hz, 1H), 6.70 (d, *J* = 15.8 Hz, 1H), 6.36 (dd, *J* = 15.8, 6.7 Hz, 1H), 5.46 (d, *J* = 6.5 Hz, 2H), 4.92 – 4.84 (m, 1H), 2.49 (d, *J* = 8.2 Hz, 2H), 2.38 (s, 1H), 2.03 (t, *J* = 14.6 Hz, 3H), 1.97 – 1.67 (m, 4H), 1.66 – 1.47 (m, 6H), 1.37 (d, *J* = 8.2 Hz, 3H), 1.26 – 1.00 (m, 13H), 0.95 (d, *J* = 6.4 Hz, 3H), 0.90 (d, *J* = 6.6 Hz, 6H), 0.72 (s, 3H).

^{13}C NMR (101 MHz, CDCl$_3$) δ 165.9, 147.6, 139.7, 136.3, 131.3, 130.9, 130.2, 129.9, 128.6, 128.0, 126.7, 126.1, 122.8, 74.8, 74.7, 56.7, 56.2, 50.0, 42.3, 39.8, 39.6, 38.2, 37.0, 36.7, 36.2, 35.8, 32.0, 31.9, 28.3, 28.0, 27.9, 24.3, 23.9, 22.9, 22.6, 21.1, 19.4, 18.8, 11.9.

HRMS (ESI) *m/z*: $[M + H]^+$ calcd. for $C_{43}H_{59}O_3$ 623.4459, found 623.4459.

HPLC (ChiralPak AD-H column), hexane/*i*-PrOH = 95:5, flow rate = 1.0 mL/min, λ = 254 nm, t_R = 11.796 min (major), t_R = 14.448 min (minor).

4-((*R*,*E*)-1-hydroxy-3-phenylallyl)phenyl (1*R*,4a*R*,4b*R*,10a*R*)-7-isopropyl-1,4a-dimethyl-1,2,3,4,4a,4b,5,6,10,10a-decahydrophenanthrene-1-carboxylate (75)

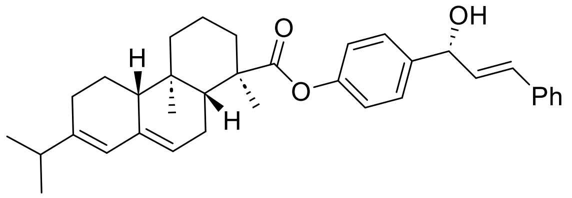

75

Prepared by the general procedure B with aldehyde (0.2 mmol, 1.0 equiv), vinyl chloride **2** (1.5 equiv), $NiBr_2$•glyme (10 mol%), **L11** (5 mol %), NaI (2.0 equiv), $ZnBr_2$ (20 mol%), Zn (3.0 equiv), Et_3N (2.0 equiv) in 2-Me-THF (1.0 mL) under nitrogen at 25 °C for 48 h.

White soild, R_f = 0.5 (petroleum ether/ethyl acetate = 4/1), 50.0 mg (49% yield), 99:1 d.r.

^{1}H NMR (400 MHz, CDCl$_3$) δ 7.42 (dd, *J* = 13.6, 7.7 Hz, 4H), 7.34 (t, *J* = 7.4 Hz, 2H), 7.28 – 7.25 (m, 1H), 7.03 (d, *J* = 8.5 Hz, 2H), 6.69 (d, *J* = 15.9 Hz, 1H), 6.37 (dd, *J* = 15.9, 6.5 Hz, 1H),

5.83 (s, 1H), 5.49 – 5.42 (m, 1H), 5.39 (d, J = 6.5 Hz, 1H), 2.34 – 2.09 (m, 6H), 2.06 – 1.93 (m, 4H), 1.89 – 1.80 (m, 2H), 1.72 – 1.65 (m, 2H), 1.41 (s, 3H), 1.28 – 1.20 (m, 2H), 1.05 (dd, J = 6.8, 3.8 Hz, 6H), 0.91 (s, 3H).

^{13}C NMR (101 MHz, CDCl$_3$) δ 177.2, 150.7, 145.6, 140.1, 136.4, 135.6, 131.3, 130.7, 128.6, 127.9, 127.4, 126.7, 122.4, 121.7, 120.5, 74.6, 50.9, 46.9, 45.1, 38.3, 37.1, 34.9, 34.6, 27.5, 25.8, 22.5, 21.4, 20.9, 18.2, 17.2, 14.1.

HRMS (ESI) *m/z*: [M - OH]$^+$ calcd. for $C_{35}H_{41}O_2$ 493.3101, found 493.3105.

HPLC (ChiralPak AD-H column), hexane/*i*-PrOH = 90:10, flow rate = 1.0 mL/min, λ = 254 nm, t_R = 18.309 min (major), t_R = 20.214 min (minor).

4-((*R*,*E*)-1-hydroxy-3-phenylallyl)phenyl (*R*)-4-((3*R*,5*R*,8*R*,9*S*,10*S*,13*R*,14*S*,17*R*)-3-acetoxy-10,13-dimethylhexadecahydro-1*H*-cyclopenta[*a*]phenanthren-17-yl)pentanoate (76)

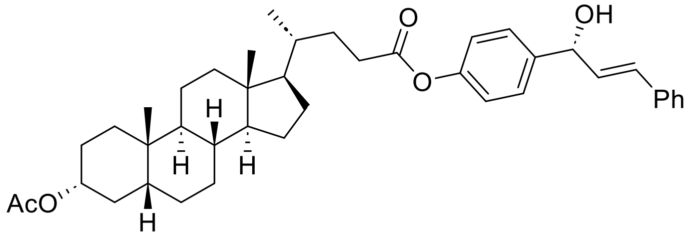

76

Prepared by the general procedure B with aldehyde (0.2 mmol, 1.0 equiv), vinyl chloride **2** (1.5 equiv), NiBr$_2$•glyme (10 mol%), **L11** (5 mol %), NaI (2.0 equiv), ZnBr$_2$ (20 mol%), Zn (3.0 equiv), Et$_3$N (2.0 equiv) in 2-Me-THF (1.0 mL) under nitrogen at 25 °C for 48 h.

White soild, R_f = 0.5 (petroleum ether/ethyl acetate = 4/1), 85.9 mg (69% yield), 99:1 d.r.

^{1}H NMR (400 MHz, CDCl$_3$) δ 7.46 (d, J = 8.6 Hz, 2H), 7.40 (d, J = 7.2 Hz, 2H), 7.33 (t, J = 7.5 Hz, 2H), 7.28 –7.24 (m, 1H), 7.09 (d, J = 8.5 Hz, 2H), 6.70 (d, J = 15.8 Hz, 1H), 6.37 (dd, J = 15.8, 6.5 Hz, 1H), 5.40 (d, J = 6.5 Hz, 1H), 4.78 – 4.70 (m, 1H), 2.66 – 2.59 (m, 1H), 2.53 – 2.45 (m, 1H), 2.31 (s, 1H), 2.08 – 1.80 (m, 9H), 1.70 (d, J = 6.7 Hz, 1H), 1.65 – 1.31 (m, 12H), 1.27 – 1.03 (m, 7H), 1.00 (d, J = 6.2 Hz, 3H), 0.95 (s, 3H), 0.69 (s, 3H).

^{13}C NMR (101 MHz, CDCl$_3$) δ 172.8, 170.8, 150.2, 140.3, 136.4, 131.3, 130.7, 128.6, 127.9, 127.5, 126.6, 121.7, 74.6, 74.4, 56.5, 56.0, 42.8, 41.9, 40.4, 40.2, 35.8, 35.4, 35.0, 34.6, 32.3, 31.4, 31.0, 28.3, 27.0, 26.6, 26.3, 24.2, 23.4, 21.5, 20.9, 18.4, 12.1.

HRMS (ESI) *m/z*: [M - OH]$^+$ calcd. for $C_{41}H_{53}O_4$ 609.3938, found 609.3941.

HPLC (ChiralPak OD-H column), hexane/*i*-PrOH = 70:30, flow rate = 1.0 mL/min, λ = 254 nm, t_R = 15.316 min (major), t_R = 11.180 min (minor).

(1*R*,2*S*,5*R*)-2-isopropyl-5-methylcyclohexyl 4-((*R*,*E*)-1-hydroxy-3-phenylallyl)benzoate (77)

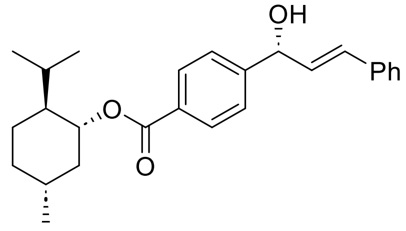

77

Prepared by the general procedure B with aldehyde (0.2 mmol, 1.0 equiv), vinyl chloride **2** (1.5 equiv), $NiBr_2$•glyme (10 mol%), **L11** (5 mol %), NaI (2.0 equiv), $ZnBr_2$ (20 mol%), Zn (3.0 equiv), Et_3N (2.0 equiv) in 2-Me-THF (1.0 mL) under nitrogen at 25 °C for 24 h.
Colorless oil, R_f = 0.5 (petroleum ether/ethyl acetate = 4/1), 55.6 mg (71% yield), >99:1 d.r.
^{1}H NMR (400 MHz, $CDCl_3$) δ 8.07 (d, J = 8.0 Hz, 2H), 7.53 (d, J = 8.1 Hz, 2H), 7.40 (d, J = 7.6 Hz, 2H), 7.33 (t, J = 7.5 Hz, 2H), 7.26 (d, J = 7.1 Hz, 1H), 6.71 (d, J = 15.8 Hz, 1H), 6.36 (dd, J = 15.8, 6.8 Hz, 1H), 5.46 (d, J = 6.8 Hz, 1H), 4.95 (td, J = 10.8, 4.4 Hz, 1H), 2.38 (s, 1H), 2.15 (d, J = 12.2 Hz, 1H), 2.01 – 1.94 (m, 1H), 1.74 (t, J = 12.9 Hz, 2H), 1.64 – 1.53 (m, 2H), 1.20 – 1.18 (m, 2H), 1.00 – 0.91 (m, 7H), 0.81 (d, J = 7.0 Hz, 3H).
^{13}C NMR (101 MHz, $CDCl_3$) δ 166.0, 147.6, 136.3, 131.3, 130.9, 130.2, 129.9, 128.6, 128.0, 126.7, 126.1, 74.9, 74.8, 47.3, 41.0, 34.3, 31.5, 26.6, 23.7, 22.1, 20.8, 16.6.
HRMS (ESI) *m/z*: $[M - OH]^+$ calcd. for $C_{26}H_{31}O_2$ 375.2319, found 375.2323.
HPLC (ChiralPak AD-H column), hexane/*i*-PrOH = 85:15, flow rate = 1.0 mL/min, λ = 254 nm, t_R = 13.385 min (major), t_R = 28.091 min (minor).

8. Transformations of Chiral Products

(*S*,*E*)-1,3-diphenylprop-2-en-1-amine (78)[10,11]

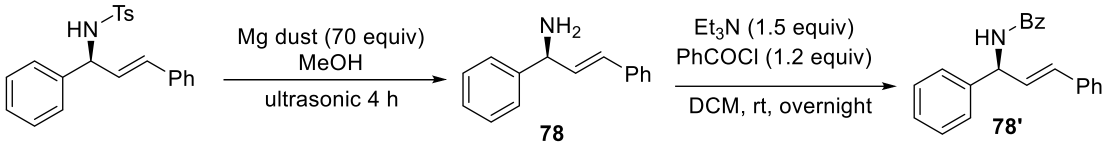

Magnesium dust (336 mg, 14.0 mmol) was added in one portion to a suspension of **3** (72.6 mg, 0.2 mmol) in MeOH (6.0 mL). The reaction mixture was stirred under sonication for 4 h. The reaction mixture was diluted with ether and carefully quenched with saturated NH_4Cl solution. The product was extracted with EtOAc (3 x 10 mL), and the combined organic extracts were washed with brine (20 mL), dried over anhydrous $MgSO_4$, and concentrated under reduced pressure. The crude product was purified on silica gel PTLC (DCM/EA = 2/1) to give the allylic amine.
Colorless oil, R_f = 0.2 (dichloromethane /ethyl acetate = 2/1), 26.3 mg (63% yield), 97:3 e.r. The e.r. was determined by converting it to compound **78'**. The spectral data of the product are reported in the literature.[12]
$[\alpha]_D^{20}$ = -20.0 (c = 0.32, CH_2Cl_2).
^{1}H NMR (400 MHz, $CDCl_3$) δ 7.49 – 7.23 (m, 10H), 6.62 (d, J = 15.8 Hz, 1H), 6.41 (dd, J = 15.8, 6.6 Hz, 1H), 4.77 (d, J = 6.6 Hz, 1H), 3.50 (br s, 2H).
^{13}C NMR (101 MHz, $CDCl_3$) δ 136.7, 132.3, 130.1, 128.8, 128.6, 127.6, 127.5, 126.9, 126.5, 57.8.
HPLC (ChiralPak OD-H column), hexane/*i*-PrOH = 80:20, flow rate = 1.0 mL/min, λ = 254 nm, t_R = 12.440 min (major), t_R = 14.287 min (minor).
HRMS (ESI) *m/z*: $[M + H]^+$ calcd. for $C_{15}H_{16}N$ 210.1277, found 210.1275.

tert-butyl (*S*,*E*)-(1,3-diphenylallyl)carbamate (79)[12]

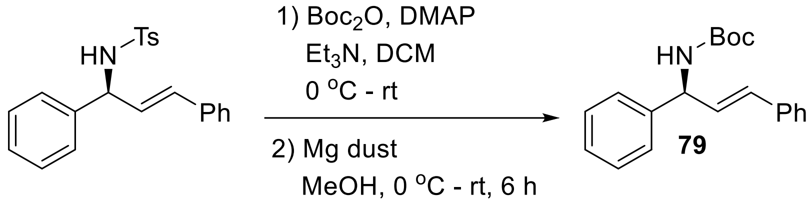

Step 1: A 4 ml vial was charged with sulfonyl amine **3** (0.1 mmol, 36.3 mg, 1.0 equiv.), DMAP (0.06 mmol, 7.3 mg, 0.6 equiv.), and anhydrous DCM (1.5 mL). Boc_2O (0.2 mmol, 43.6 mg, 2.0 equiv.) and Et_3N (0.3 mmol, 42 μL, 3.0 equiv.) were added at 0 °C. The reaction mixture was stirred at room temperature overnight, and then concentrated under reduced pressure. Purification by column chromatography afforded N-Boc product.

Step 2: The above compound was dissolved in MeOH (6 mL). Mg powder (5 mmol, 120 mg, 50 equiv.) was added at 0 °C. The reaction mixture was stirred vigorously until the starting material was consumed completely. The reaction mixture was quenched with saturated NH_4Cl solution, extracted with DCM 3 times. The combined organic layer was dried over $NaSO_4$, and concentrated. Purification by column chromatography afforded the desired product (26.3 mg, 85% overall yield).

White solid, R_f = 0.5 (petroleum ether/ ethyl acetate = 10/1), 26.3 mg (85% yield), 95:5 e.r.

[α]$_D$^{20} = -3.4 (*c* = 0.52, CH_2Cl_2).

^{1}H NMR (400 MHz, $CDCl_3$) δ 7.46 – 7.22 (m, 10H), 6.57 (d, *J* = 15.9 Hz, 1H), 6.35 (dd, *J* = 15.9, 6.0 Hz, 1H), 5.48 (br s, 1H), 4.99 (br s, 1H), 1.48 (s, 9H).

^{13}C NMR (101 MHz, $CDCl_3$) δ 155.1, 141.4, 136.6, 131.0, 129.6, 128.8, 128.6, 127.7, 127.6, 127.0, 126.6, 79.8, 56.3, 28.4.

HPLC (ChiralPak OD-H column), hexane/*i*-PrOH = 90:10, flow rate = 1.0 mL/min, λ = 254 nm, t_R = 5.939 min (minor), t_R = 6.773 min (major).

HRMS (ESI) *m/z*: $[M + H]^+$ calcd. for $C_{20}H_{24}NO_2$ 310.1802, found 310.1794.

(*S*)-*N*-(1,3-diphenylpropyl)-4-methylbenzenesulfonamide (80)[13]

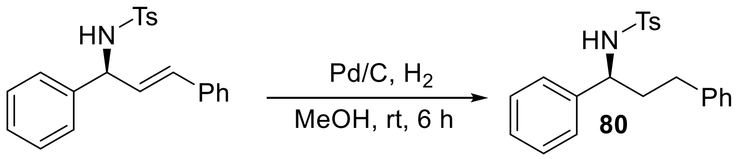

Add 10% palladium carbon (7.0 mg) and (E)-N-(1,3-diphenylallyl)-4-methylbenzenesulfonamide (**3**) (36.3 mg, 0.1 mmol, 1.0 equiv) into a round-bottomed flask, replace the gas with nitrogen three times, then replace the gas with hydrogen three times, then insert a hydrogen balloon, inject methanol as a solvent, the reaction mixture was stirred at room temperature for 6 h. After the reaction, suction filtration under reduced pressure and residue was purified by column chromatography (petroleum ether/EtOAc = 6:1) to provide the title compound as a white solid in 91% yield.

White solid, R_f = 0.3 (petroleum ether/ ethyl acetate = 6/1), 33.2 mg (91% yield), 97:3 e.r.

[α]$_D$^{20} = -18.3 (*c* = 0.66, CH_2Cl_2).

^{1}H NMR (400 MHz, $CDCl_3$) δ 7.55 (d, *J* = 8.1 Hz, 2H), 7.32 – 7.00 (m, 12H), 5.41 (d, *J* = 7.7 Hz, 1H), 4.31 (q, *J* = 7.4 Hz, 1H), 2.63 – 2.47 (m, 2H), 2.38 (s, 3H), 2.20 – 1.97 (m, 2H).

^{13}C NMR (101 MHz, $CDCl_3$) δ 143.0, 140.9, 140.7, 137.6, 129.3, 128.5, 128.4, 127.4, 127.1, 126.6, 126.0, 57.9, 39.0, 32.1, 21.5.

HPLC (ChiralPak OD-H column), hexane/*i*-PrOH = 90:10, flow rate = 1.0 mL/min, λ = 254 nm, t_R = 13.185 min (minor), t_R = 22.959 min (major).
HRMS (ESI) *m/z*: $[M + Na]^+$ calcd. for $C_{22}H_{23}NNaO_2S$ 388.1342, found 388.1339.

4-methyl-N-((1*R*)-phenyl(3-phenyloxiran-2-yl)methyl)benzenesulfonamide (81)[13]

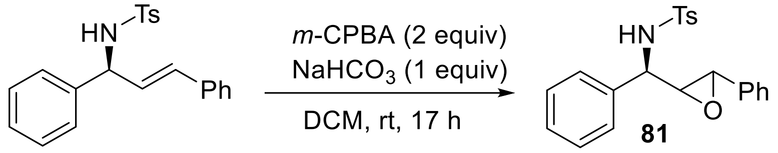

To a solution of (E)-N-(1,3-diphenylallyl)-4-methylbenzenesulfonamide (**3**) (36.3 mg, 0.10 mmol, 1.0 equiv) in CH_2Cl_2 (1.0 mL) was added $NaHCO_3$ (16.8 mg, 0.20 mmol, 2.0 equiv) and m-CPBA (34.4 mg, 0.20 mmol, 2.0 equiv) and the reaction was then stirred at rt for 17 h. The reaction mixture was washed with sat. aq. Na_2SO_3 solution (2 x 15 mL) and then sat. aq. Na_2CO_3 solution (2 x 15 mL). The organic layer was dried ($MgSO_4$) and solvent evaporated in vacuo. The residue was purified by column chromatography (petroleum ether/EtOAc = 6:1) to provide the title compound as a white solid in 89% yield.
White solid, R_f = 0.3 (petroleum ether/ ethyl acetate = 6/1), 34.0 mg (89% yield), 97:3 e.r. 2:1 d.r.
[α]D20 = -15.1 (*c* = 0.68, CH_2Cl_2).
^{1}H NMR (400 MHz, $CDCl_3$) δ 7.69 – 7.60 (m, 3H), 7.35 – 7.14 (m, 18H), 5.48 (d, *J* = 6.5 Hz, 1H, major), 5.44 (d, *J* = 8.6 Hz, 0.5H, minor), 4.77 (dd, *J* = 8.5, 2.4 Hz, 0.5H, minor), 4.47 (t, *J* = 6.0 Hz, 1H, major), 3.92 (d, *J* = 2.0 Hz, 0.5H, minor), 3.71 (d, *J* = 2.0 Hz, 1H, major), 3.28 (dd, *J* = 5.5, 2.0 Hz, 1H, major), 3.25 (t, *J* = 2.2 Hz, 0.5H, minor), 2.40 (s, 1.5H, minor), 2.39 (s, 3H, major).
^{13}C NMR (101 MHz, $CDCl_3$) δ 143.5, 143.3, 138.2, 137.7, 137.3, 136.5, 136.0, 135.9, 129.6, 129.5, 128.8, 128.8, 128.5, 128.5, 128.4, 128.4, 128.4, 128.1, 127.4, 127.1, 127.0, 127.0, 125.8, 125.8, 64.3, 63.7, 58.3, 57.7, 56.4, 55.8, 21.5.
HPLC (ChiralPak AD-H column), hexane/*i*-PrOH = 70:30, flow rate = 1.0 mL/min, λ = 220 nm, t_R = 9.724 min (minor-1), t_R = 11.653 min (major-1), t_R = 14.605 min (minor-2), t_R = 16.131 min (major-2).
HRMS (ESI) *m/z*: $[M + H]^+$ calcd. for $C_{22}H_{22}NO_3S$ 380.1315, found 380.1320.

(2*S*,3*S*,4*R*)-3-bromo-2-phenyl-4-(*p*-tolyl)-1-tosylazetidine (82)[13]

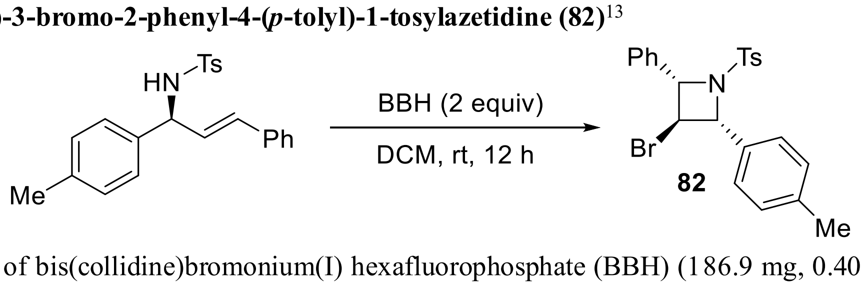

A solution of bis(collidine)bromonium(I) hexafluorophosphate (BBH) (186.9 mg, 0.40 mmol, 2.0 equiv) in dichloromethane (5 mL) was added at room temp. over 6 h to a solution of sulfonamide (**9**) (75.5 mg, 0.20 mmol, 1.0 equiv) in dichloromethane (5.0 mL). After complete addition, the mixture was stirred for 12 h. Silica gel was added and the solvent removed under reduced pressure. The residue was purified by column chromatography (petroleum ether/EtOAc = 15:1) to provide the title compound as a colorless oil in 66% yield.
Colorless oil, R_f = 0.5 (petroleum ether/ ethyl acetate = 10/1), 60.7 mg (66% yield), 98:2 e.r.
[α]D20 = -2.7 (*c* = 1.20, CH_2Cl_2).

^{1}H NMR (400 MHz, CDCl$_3$) δ 7.65 – 7.60 (m, 2H), 7.55 – 7.50 (m, 2H), 7.44 – 7.37 (m, 5H), 7.32 – 7.27 (m, 2H), 7.23 (d, J = 7.9 Hz, 2H), 5.06 (t, J = 7.2 Hz, 2H), 3.97 (t, J = 6.9 Hz, 1H), 2.45 (s, 3H), 2.40 (s, 3H).
^{13}C NMR (101 MHz, CDCl$_3$) δ 144.5, 138.9, 137.5, 134.5, 132.6, 129.7, 129.6, 128.9, 128.9, 128.4, 126.6, 126.5, 72.1, 72.1, 47.9, 21.7, 21.3.
HPLC (ChiralPak OD-H column), hexane/*i*-PrOH = 90:10, flow rate = 1.0 mL/min, λ = 220 nm, t_R = 6.056 min (major), t_R = 6.479 min (minor).
HRMS (ESI) *m/z*: [M + K]$^+$ calcd. for $C_{23}H_{22}BrKNO_2S$ 494.0186, found 494.1187.

9. Mechanistic study

A) Reaction with performed dinickel complex[6]

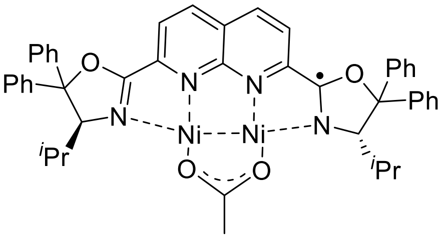

L1·Ni$_2$(OAc): In an N_2-filled glovebox, **L1** (131.4 mg, 0.20 mmol, 1.00 equiv), $Ni(OAc)_2$ (17.7 mg, 0.10 mmol, 0.50 equiv) and $Ni(cod)_2$ (82.5 mg, 0.31 mmol, 1.50 equiv) were added to a 10 mL Schlenk flask as solids. A magnetic stir bar and dry THF (2.5 mL) were added. The flask was sealed and removed from the glovebox. The reaction mixture was stirred at 75 °C. After 120 h, the flask was cooled to room temperature and pumped back into the glovebox. Under N_2 atmosphere, the reaction mixture was filtered through a glass fiber pad and concentrated under vacuum. The resulting residue was washed with pentane (2 × 1 mL) and concentrated. The resulting powder was redissolved in C_6H_6 and filtered through a glass fiber pad. The filtrate was lyophilized under vacuum, giving the product as a bright black solid. Yield = 110 mg (65%).

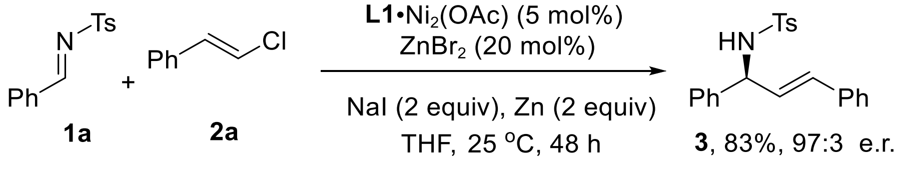

The **L1**·$Ni_2(OAc)$ (5 mol %), $ZnBr_2$ (9.0 mg, 0.04 mmol, 20 mol %), NaI (60.0 mg, 0.4 mmol, 2.0 equiv) and Zn powder (26.0 mg, 0.4 mmol, 2.0 equiv) were introduced into a flame-dried Schlenk tube in an N_2-filled glovebox. After taking out from the glove box, reaction tube was connected to Schlenk line under N_2. 1 mL dry THF was injected into the Schlenk tube. The resulting mixture was stirred at 40 °C for 2 h, at which point the suspension was allowed to cool to rt. The vinyl chloride **2a** (41.4 mg, 0.3 mmol, 1.5 equiv) and imine **1a** (51.8mg, 0.2 mmol, 1.0 equiv) were sequentially added under nitrogen. The resulting mixture was stirred at 25 °C for 48 hours. The reaction mixture was quenched with saturated NH_4Cl (aq.) solution and extracted with EtOAc (3x). The organic phase was washed with brine, dried over anhydrous Na_2SO_4, filtered, and concentrated under reduced pressure. The crude material was purified using column chromatography to give the pure

product. 60.2 mg (83% yield), 97:3 e.r.

B) Radical probe

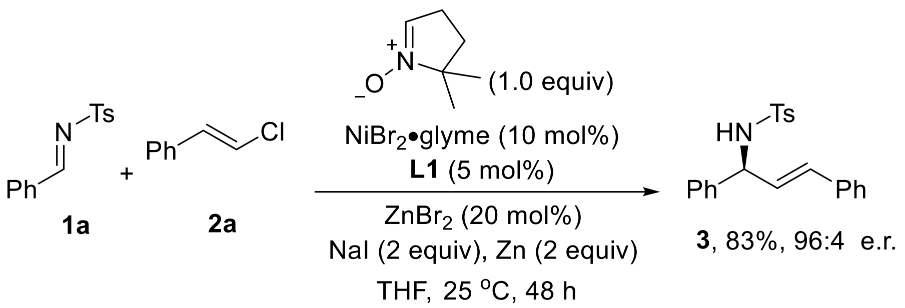

In a N_2-filled glove-box, an oven-dried Schlenk tube equipped with a magnetic stir bar was charged with $NiBr_2$•glyme (6.1 mg, 0.02 mmol, 10 mol %), **L1** (0.01 mmol, 5 mol %), $ZnBr_2$ (9.0 mg, 0.04 mmol, 20 mol %), NaI (60.0 mg, 0.4 mmol, 2.0 equiv) and Zn powder (26.0 mg, 0.4 mmol, 2.0 equiv). Dry THF (1.0 mL) was added, then the mixture was allowed to stir at room temperature for 30 min. **2a** (41.4 mg, 0.3 mmol, 1.5 equiv), **1a** (51.8mg, 0.2 mmol, 1.0 equiv), DMPO (22.6 mg, 0.2 mmol, 1.0 equiv) were added to the solution. The flask was sealed and removed from the glovebox. The resulting mixture was stirred at 25 °C for 48 hours. The reaction mixture was quenched with saturated NH_4Cl (aq.) solution and extracted with EtOAc (3x). The organic phase was washed with brine, dried over anhydrous Na_2SO_4, filtered, and concentrated under reduced pressure. The crude material was purified using column chromatography to give the pure product. 60.3 mg (83% yield), 96:4 e.r.

C) Probe of α-amino radical

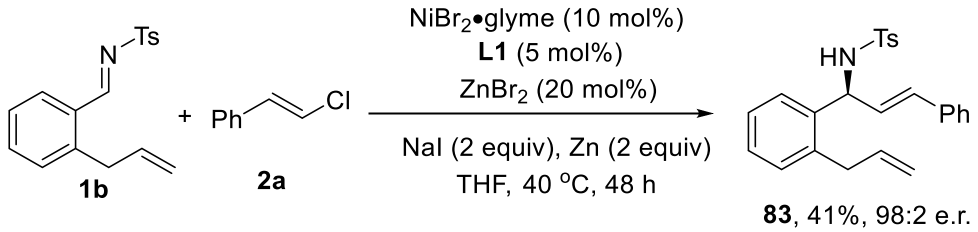

The $NiBr_2$•glyme (6.1 mg, 0.02 mmol, 10 mol %), **L1** (6.6 mg, 0.01 mmol, 5 mol %), $ZnBr_2$ (9.0 mg, 0.04 mmol, 20 mol %), NaI (60.0 mg, 0.4 mmol, 2.0 equiv) and Zn powder (26.0 mg, 0.4 mmol, 2.0 equiv) were introduced into a flame-dried Schlenk tube in an N_2-filled glove box. After taking out from the glove box, Schlenk tube was connected to Schlenk line under N_2. 1 mL dry THF was injected into the Schlenk tube, the mixture was stirred at r.t. for 30 min. The vinyl chloride **2a** (41.4 mg, 0.3 mmol, 1.5 equiv) and imine **1b** (60 mg, 0.2 mmol, 1.0 equiv) were sequentially added under nitrogen. The resulting mixture was stirred at 40 °C for 48 hours. The reaction mixture was cooled to room temperature, quenched with saturated NH_4Cl (aq.) solution and extracted with EtOAc (3x). The organic phase was washed with brine, dried over anhydrous Na_2SO_4, filtered, and concentrated under reduced pressure. The crude material was purified using column chromatography to give the pure product.

(*S*,*E*)-*N*-(1-(2-allylphenyl)-3-phenylallyl)-4-methylbenzenesulfonamide (83)

White solid, R_f = 0.3 (petroleum ether/ ethyl acetate = 6/1), 33.3 mg (41% yield), 98:2 e.r.

$[\alpha]_D^{20}$ = +1.9 (c = 0.54, CH_2Cl_2).

^{1}H NMR (400 MHz, $CDCl_3$) δ 7.67 (d, J = 8.3 Hz, 2H), 7.30 – 7.13 (m, 11H), 6.29 (d, J = 15.9 Hz, 1H), 6.13 (dd, J = 15.9, 6.1 Hz, 1H), 6.02 – 5.87 (m, 1H), 5.40 (t, J = 7.2 Hz, 1H), 5.14 – 5.06 (m, 1H), 5.04 – 4.92 (m, 2H), 3.47 (dd, J = 16.1, 6.5 Hz, 1H), 3.36 (dd, J = 16.1, 5.9 Hz, 1H), 2.36 (s, 3H).

^{13}C NMR (101 MHz, $CDCl_3$) δ 143.3, 137.8, 137.7, 137.2, 136.8, 136.1, 131.9, 130.4, 129.4, 128.5, 128.4, 128.1, 127.9, 127.3, 126.9, 126.5, 116.4, 55.8, 36.8, 21.4.

HPLC (ChiralPak OD-H column), hexane/i-PrOH = 90:10, flow rate = 1.0 mL/min, λ = 254 nm, t_R = 9.745 min (minor), t_R = 17.160 min (major).

HRMS (ESI) m/z: $[M + Na]^+$ calcd. for $C_{25}H_{25}NNaO_2S$ 426.1498, found 426.1498.

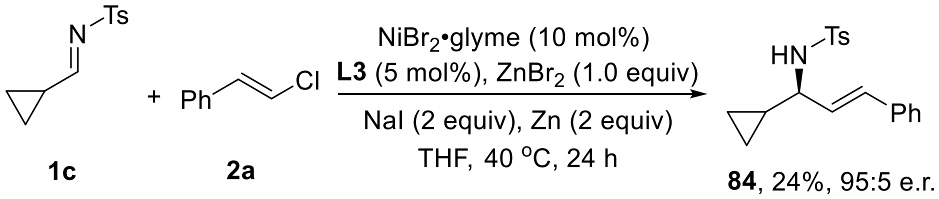

The $NiBr_2$•glyme (6.1 mg, 0.02 mmol, 10 mol %), **L3** (8.5 mg, 0.01 mmol, 5 mol %), $ZnBr_2$ (45.0 mg, 0.2 mmol, 1.0 equiv), NaI (60.0 mg, 0.4 mmol, 2.0 equiv) and Zn powder (26.0 mg, 0.4 mmol, 2.0 equiv) were introduced into a flame-dried Schlenk tube in an N_2-filled glove box. After taking out from the glove box, Schlenk tube was connected to Schlenk line under N_2. 1 mL dry THF was injected into the Schlenk tube, the mixture was stirred at r.t. for 30 min. The vinyl chloride **2a** (41.4 mg, 0.3 mmol, 1.5 equiv) and imine **1c** (44.6 mg, 0.2 mmol, 1.0 equiv) were sequentially added under nitrogen. The resulting mixture was stirred at 40 °C for 24 hours. The reaction mixture was cooled to room temperature, quenched with saturated NH_4Cl (aq.) solution and extracted with EtOAc (3x). The organic phase was washed with brine, dried over anhydrous Na_2SO_4, filtered, and concentrated under reduced pressure. The crude material was purified using column chromatography to give the pure product.

(*R*,*E*)-*N*-(1-cyclopropyl-3-phenylallyl)-4-methylbenzenesulfonamide (84)

White solid, R_f = 0.3 (petroleum ether/ ethyl acetate = 5/1), 15.4 mg (24% yield), 95:5 e.r.

$[\alpha]_D^{20}$ = +72.5 (c = 0.48, $CHCl_3$).

^{1}H NMR (400 MHz, $CDCl_3$) δ 7.77 (d, J = 8.3 Hz, 2H), 7.34 – 7.13 (m, 7H), 6.33 (d, J = 15.9 Hz, 1H), 5.86 (dd, J = 15.9, 7.1 Hz, 1H), 5.04 (d, J = 6.6 Hz, 1H), 3.39 (q, J = 7.1 Hz, 1H), 2.35 (s, 3H), 1.05 – 0.94 (m, 1H), 0.61 – 0.44 (m, 2H), 0.34 – 0.20 (m, 2H).

^{13}C NMR (101 MHz, $CDCl_3$) δ 143.2, 138.2, 136.4, 131.4, 129.5, 128.4, 127.9, 127.7, 127.4, 126.4, 60.4, 21.4, 16.5, 3.7, 3.3.

HPLC (ChiralPak OD-H column), hexane/i-PrOH = 90:10, flow rate = 1.0 mL/min, λ = 254 nm, t_R = 12.037 min (minor), t_R = 13.747 min (major).

HRMS (ESI) m/z: $[M + Na]^+$ calcd. for $C_{19}H_{21}NO_2S$ 350.1185, found 350.1177.

D) Competition between imine and aldehyde

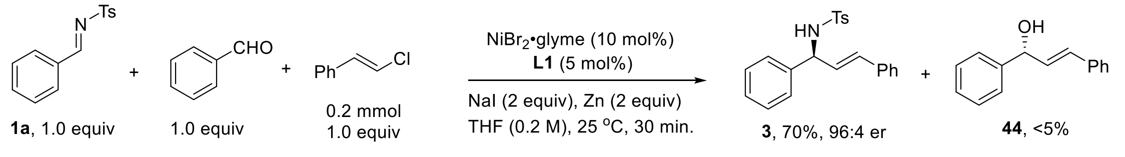

The $NiBr_2$•glyme (6.1 mg, 0.02 mmol, 10 mol %), **L1** (6.6 mg, 0.01 mmol, 5 mol %), NaI (60.0 mg, 0.4 mmol, 2.0 equiv) and Zn powder (26.0 mg, 0.4 mmol, 2.0 equiv) were introduced into a flame-dried Schlenk tube in an N_2-filled glove box. After taking out from the glove box, Schlenk tube was connected to Schlenk line under N_2. 0.5 mL dry THF was injected into the Schlenk tube, the mixture was stirred at r.t. for 30 min. The vinyl chloride **2a** (41.4 mg, 0.3 mmol, 1.5 equiv) and a solution (imine **1a** 51.9 mg, 0.2 mmol, 1.0 equiv, benzaldehyde 21.2 mg, 0.2 mmol, 1.0 equiv, 0.5 mL THF) were sequentially added under nitrogen. The resulting mixture was stirred at 25 °C for 30 min. The reaction mixture quenched with saturated NH_4Cl (aq.) solution and extracted with EtOAc (3x). The organic phase was washed with brine, dried over anhydrous Na_2SO_4, filtered, and concentrated under reduced pressure. The crude material was purified using column chromatography to give the pure product.

10. Determination of the Absolute Configuration

The absolute configuration of compound **3** has been established by comparison with literature data (for *S*-**3** in ref 14: ($[\alpha]_D^{21}$ = +21.9 (c 1.0, CH_2Cl_2), 94% ee for *S*; our experiment result of **3**: $[\alpha]_D^{20}$ = +51.7 (c 1.03, CH_2Cl_2) (96% ee)). The absolute configuration of compound **26** has been established by comparison with literature data (for *R*-**26** in ref 15: ($[\alpha]_D^{25}$ = +96.9 (c 0.32, $CHCl_3$), 99% ee for *R*; our experiment result of **26**: $[\alpha]_D^{20}$ = +94.1 (c 0.94, $CHCl_3$) (96% ee)). The absolute configuration of compound **44** has been established by comparison with literature data (for *R*-**44** in ref 16: ($[\alpha]_D^{25}$ = +26.4 (c 1.27, $CHCl_3$), 99% ee for *R*; our experiment result of **44**: $[\alpha]_D^{20}$ = +25.5 (c 0.78, $CHCl_3$) (98% ee)). All the other configurations are uncertain and based on the assumption that the configuration follows that of **3**, **26** or **44**.

11. Density functional theory calculations

All density functional theory (DFT) calculations were performed with the Gaussian 09[17] program. Geometry optimizations were performed in the gas phase using M06L[18] functional and a mixed basis set, in which the def2-SVP[19] basis set was adopted for C H O S Cl atoms, the def2-TZVP[20] basis set for N and Ni atom and lanl2dz[21] basis set for I atom (BS1). Single-point energies were further calculated using dispersion (D3)[22] corrected M06L functional and the def2-TZVP basis set for all atom under the SMD solvation model in TetraHydroFuran (THF) solvent (BS2). All optimized species were verified by the presence of zero imaginary vibrational frequency. Free energies were evaluated at 298 K using harmonic vibrational frequencies. The calculated structures were displayed with the CYL view software[23] and Pymol. IGMH analysis were processed with the Multiwfn 3.8[24] and VMD 1.9.3 program.[25] In addition, the calculation formula for free energy follows Eq-S1, G_{corr} and E_{sol} are the thermal correction for free energy base on the BS1 and the single point energy base on the BS2, respectively. Specific data as seen in Supplementary Table S8.

$$G_{sol} = E_{sol}\,(\text{BS2}) + G_{corr}\,(\text{BS1}) \qquad \text{Eq-S1}$$

Considering the impact of excessive NaI on the energy, energy correction[26] had been applied according to Eq-S2

$$\Delta G = \Delta G^{\theta} + \Delta G' \qquad \text{Eq-S2}$$

$\Delta G'$ is related to the amount of reactant, which can be expressed as $\Delta G' = RT\ln K$.

Supplementary Table S8. Benchmark of different density functionals for the relative energies of enantioselective transition states

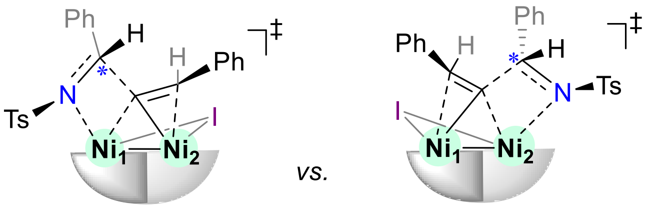

^{3}ts2-S
Favored

^{3}ts2'-R
Unfavored

	M06L-D3	TPSSh-D3	wB97XD	B3LYP-D3
$\Delta\Delta G^{\ddagger}$(kcal/mol)	1.7	2.4	1.6	1.3

Based on previous studies[27] and benchmark of DFT methods (M06L-D3, TPSSh-D3, wB97XD, and B3LYP-D3), the M06L-D3 functional was identified as an appropriate functional for dinickel complexes to evaluatie relative energies of enantioselective energy that matched experimental data. Therefore, we use M06L-D3 functional at the single point energy level in the main text and supporting information.

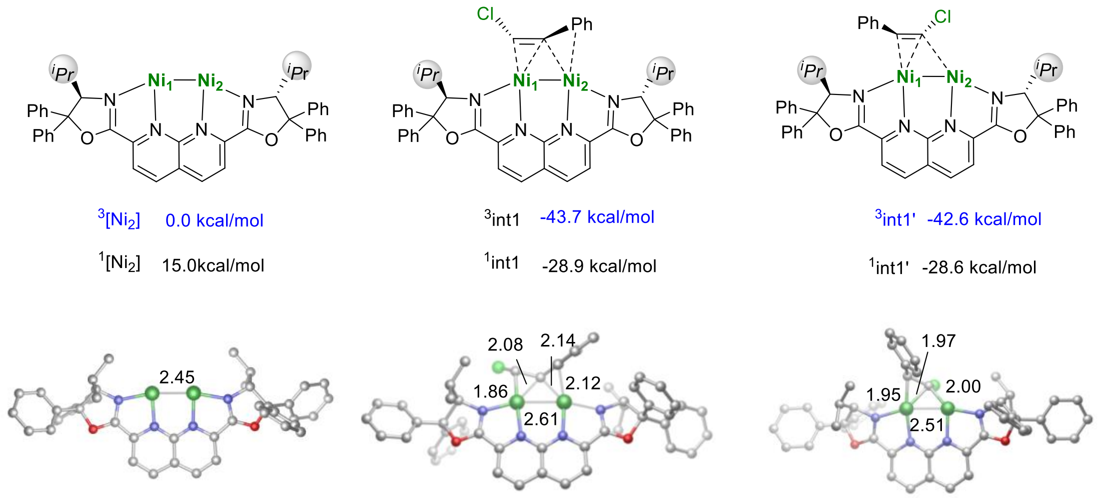

Supplementary Figure S7. Structures and energies of dinuclear (napbox)Ni2 (**[Ni]2**) and π-complex (Take the energy of **³[Ni₂]** as the energy zero point)**.**

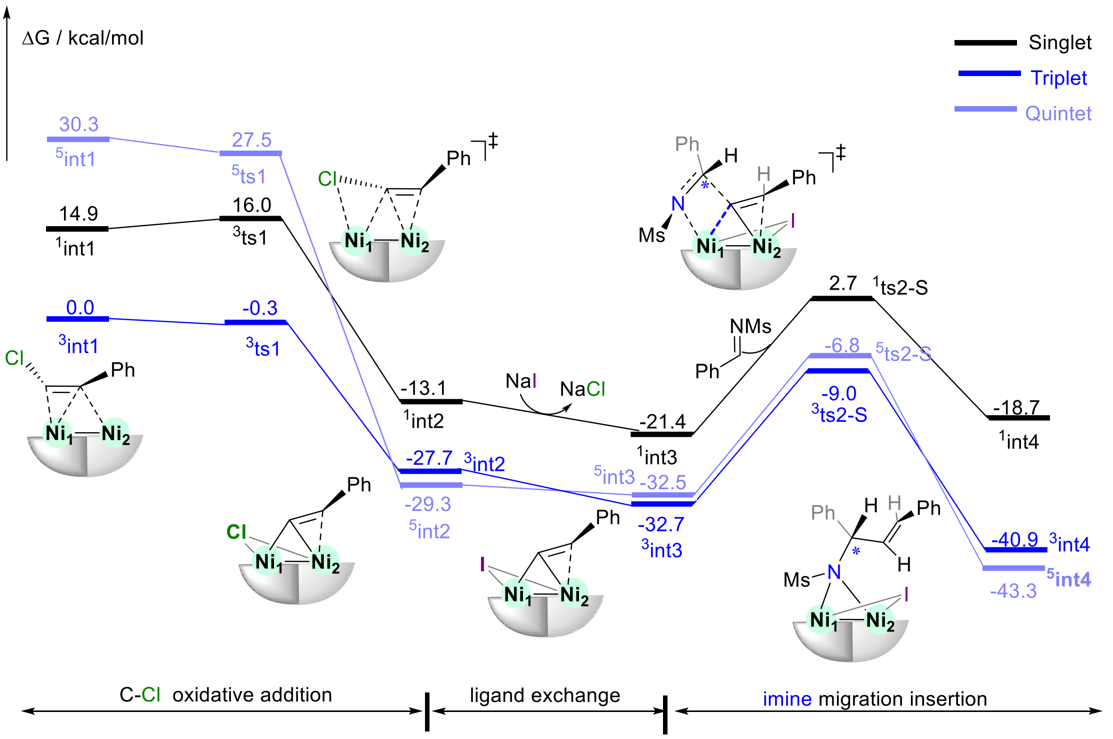

Supplementary Figure S8. Free energy profile in different multiple states.

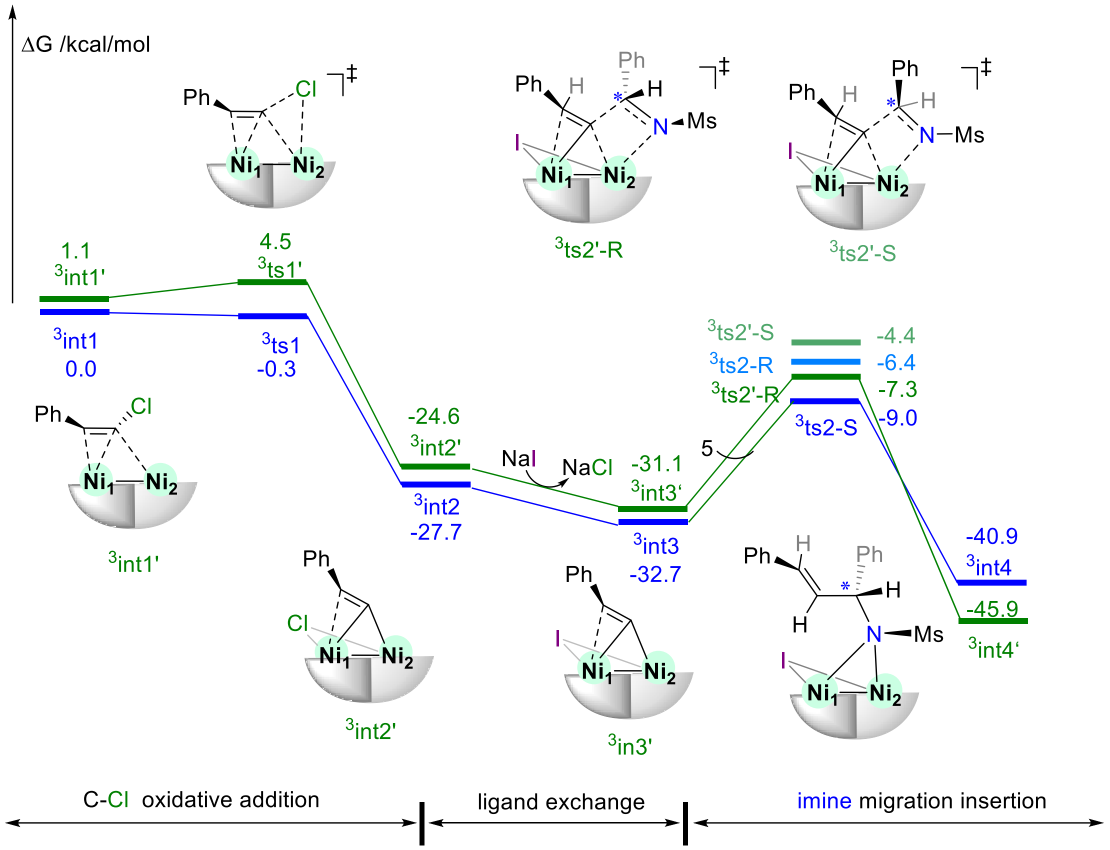

Supplementary Figure S9. Free energy profiles dominated by the Ni1 reaction site (blue line) and the Ni2 reaction site (green line) in triplet state.

Supplementary Table S9. Thermodynamic data for intermediates and transition states

	E (a.u.)	E_{sol} (a.u.)	H-corr (a.u.)	G-corr (a.u.)
**^{1}[Ni$_2$]**	-5085.380328	-5087.37494	0.787112	0.661455
**^{3}[Ni$_2$]**	-5085.410337	-5087.3996	0.786264	0.66215
**^{3}int1**	-5854.357649	-5856.82382	0.922166	0.780346
**^{3}int1’**	-5854.352271	-5856.82074	0.921504	0.779084
**^{3}ts1**	-5854.354359	-5856.82247	0.920637	0.77849
**^{3}ts1’**	-5854.346586	-5856.81472	0.92074	0.778411
**^{3}int2**	-5854.396007	-5856.86598	0.921154	0.77831
**^{3}int2’**	-5854.390477	-5856.85941	0.921913	0.776671

**^{3}int3**	-5405.709213	-5694.53463	0.920185	0.774416
**^{3}int3’**	-5405.705384	-5694.53069	0.920394	0.77308
**^{3}ts2-*R***	-6318.79321	-6608.31422	1.095506	0.925847
**^{3}ts2-*S***	-6318.796052	-6608.32058	1.095364	0.927971
**^{3}ts2′-*R***	-6318.79142	-6608.31371	1.094783	0.923904
**^{3}ts2’-*S***	-6318.790767	-6608.31146	1.095177	0.926268
**^{3}int4**	-6318.852552	-6608.37203	1.097704	0.928711
**^{3}int4’**	-6318.861491	-6608.3827	1.099391	0.931351
**^{1}int1**	-5854.336334	-5856.80259	0.922221	0.782811
**^{1}int1’**	-5854.325088	-5856.79703	0.921912	0.777598
**^{1}ts1**	-5854.339954	-5856.798569	0.920887	0.78063
**^{1}int2**	-5854.371558	-5856.8443	0.922165	0.779908
**^{1}int3**	-5405.691784	-5694.52046	0.922759	0.778226
**^{1}ts2-*S***	-6318.782222	-6608.30321	1.095892	0.929264
**^{1}int4**	-6318.823827	-6608.3426	1.098661	0.935151
**^{5}int1**	-5854.307444	-5856.77644	0.922841	0.781184
**^{5}ts1**	-5854.308927	-5856.7778	0.918592	0.778136
**^{5}int2**	-5854.401451	-5856.86903	0.922188	0.778852
**^{5}int3**	-5405.712786	-5694.53548	0.9204	0.775508
**^{5}ts2-*S***	-6318.791788	-6608.31589	1.094878	0.926923

**^{5}int4**	-6318.85985	-6608.37782	1.098514	0.93058
**NaCl**	-622.358522	-622.597456	0.0045	-0.0215
**NaI**	-173.674821	-460.262449	0.004352	-0.024

12. NMR Spectra

^{1}H NMR of Compound **S3** ($CDCl_3$, 400 MHz, 20 °C):

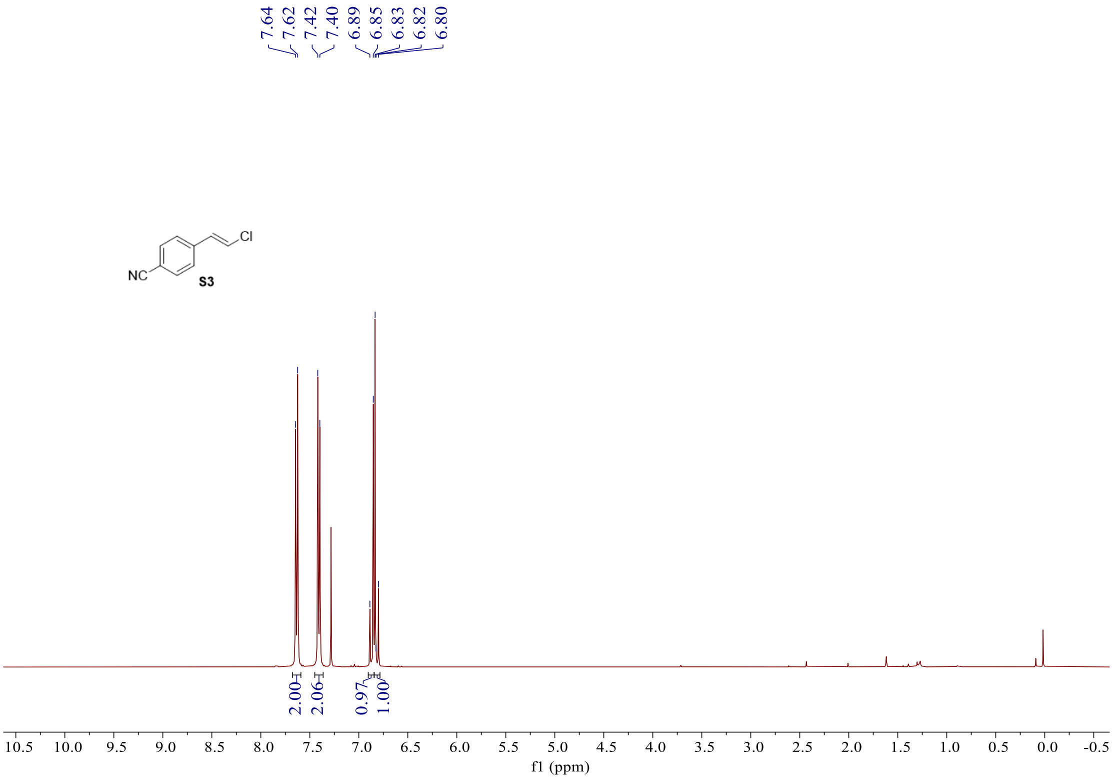

^{13}C NMR of Compound **S3** ($CDCl_3$, 101 MHz, 20 °C):

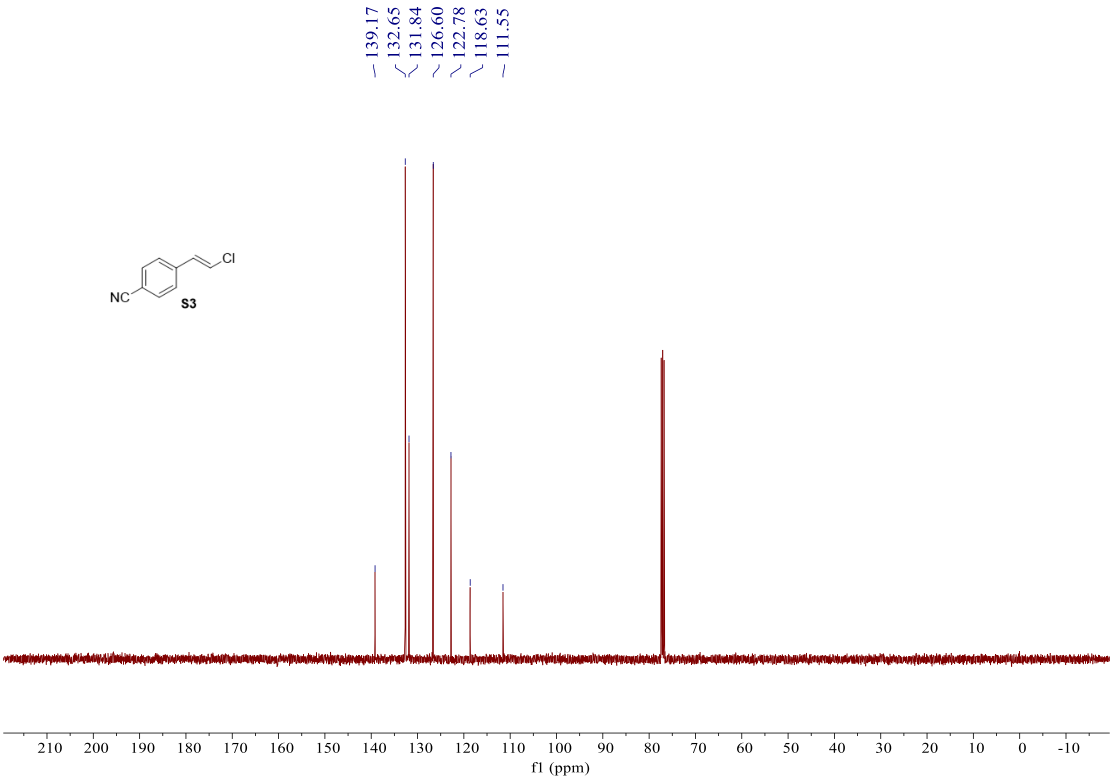

^{1}H NMR of Compound **L1** ($CDCl_3$, 400 MHz, 20 °C):

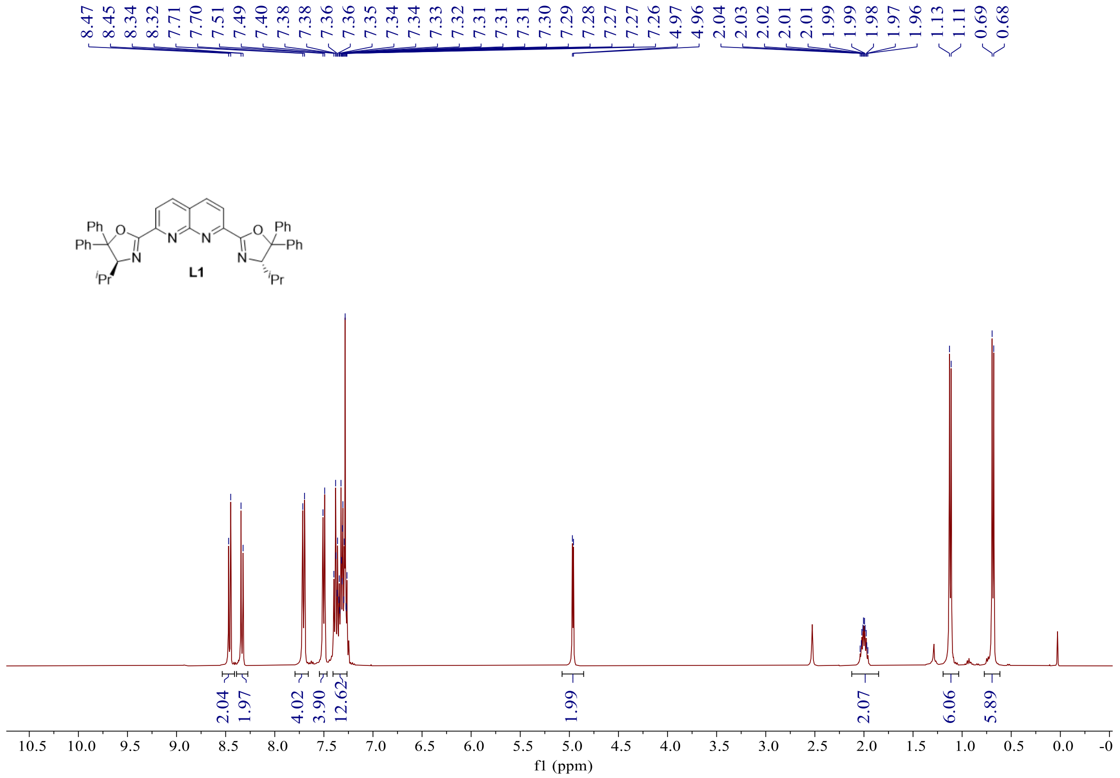

^{13}C NMR of Compound **L1** ($CDCl_3$, 101MHz, 20 °C):

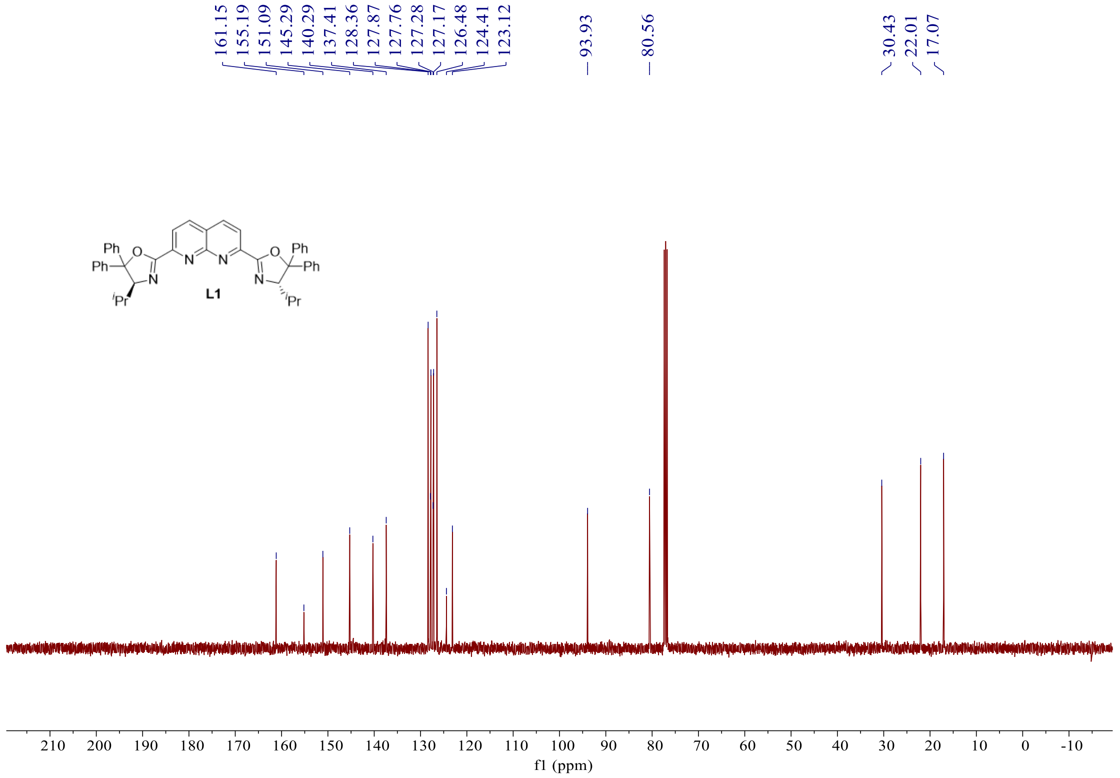

^{1}H NMR of Compound **L2** ($CDCl_3$, 400 MHz, 20 °C):

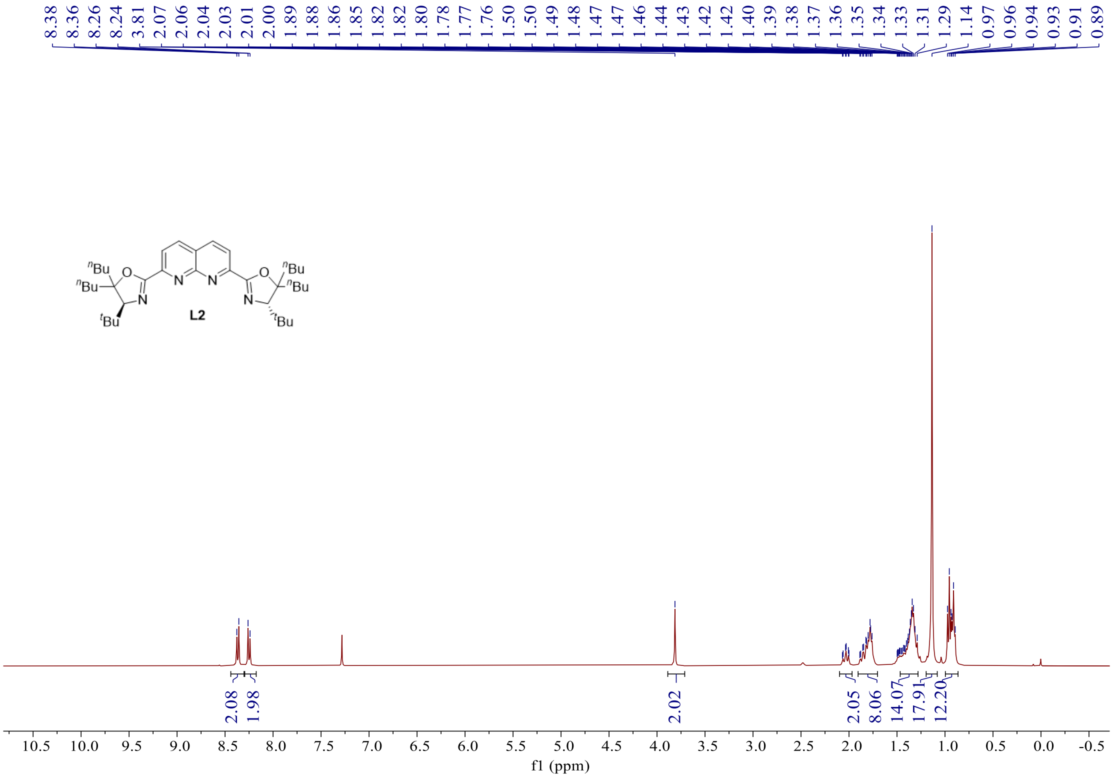

^{13}C NMR of Compound **L2** ($CDCl_3$, 101MHz, 20 °C):

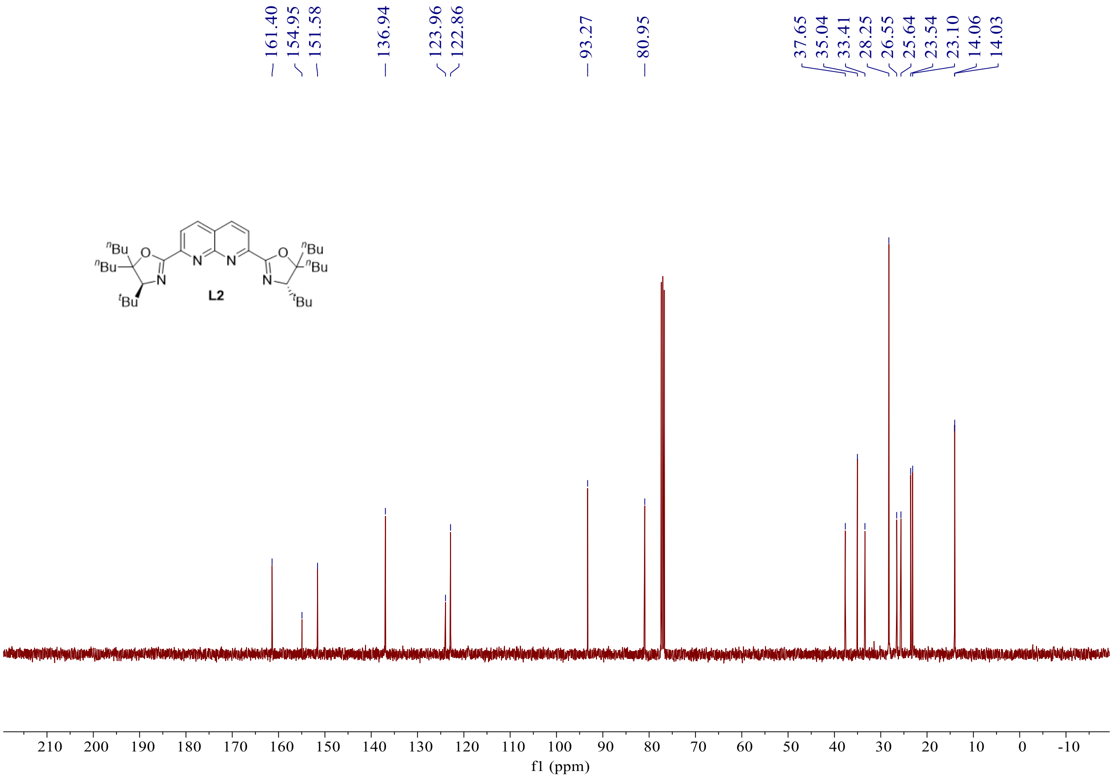

^{1}H NMR of Compound **L7** ($CDCl_3$, 400 MHz, 20 °C):

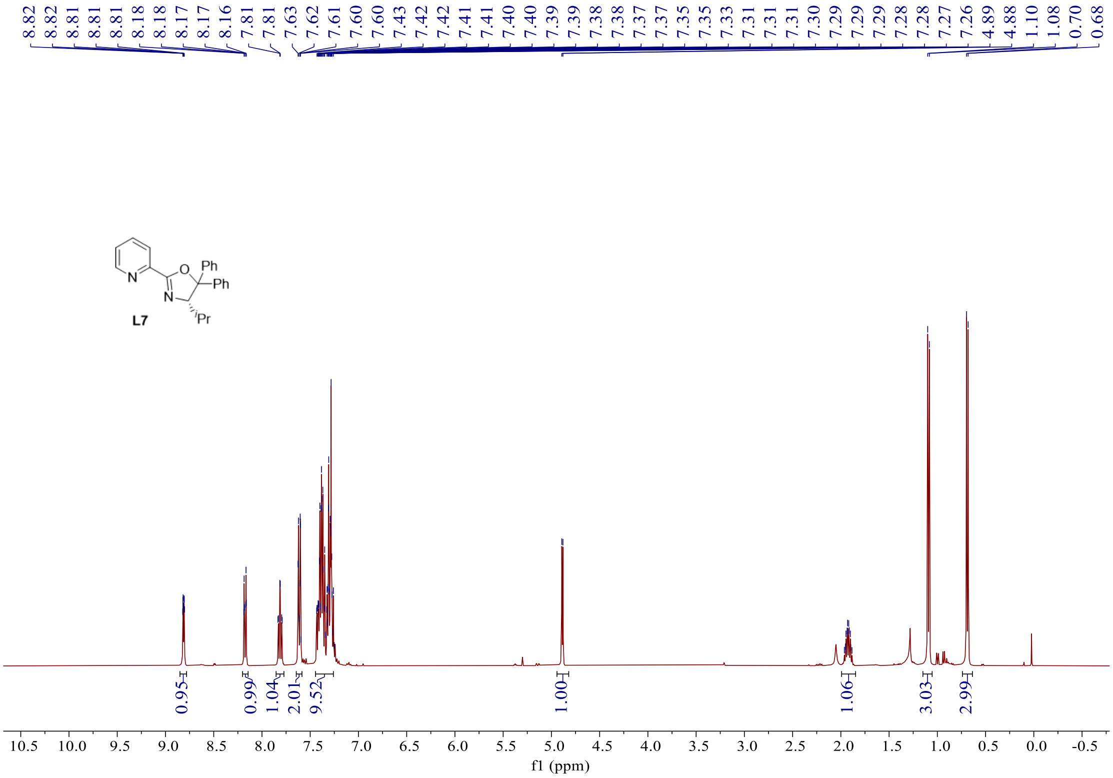

^{13}C NMR of Compound **L7** ($CDCl_3$, 101MHz, 20 °C):

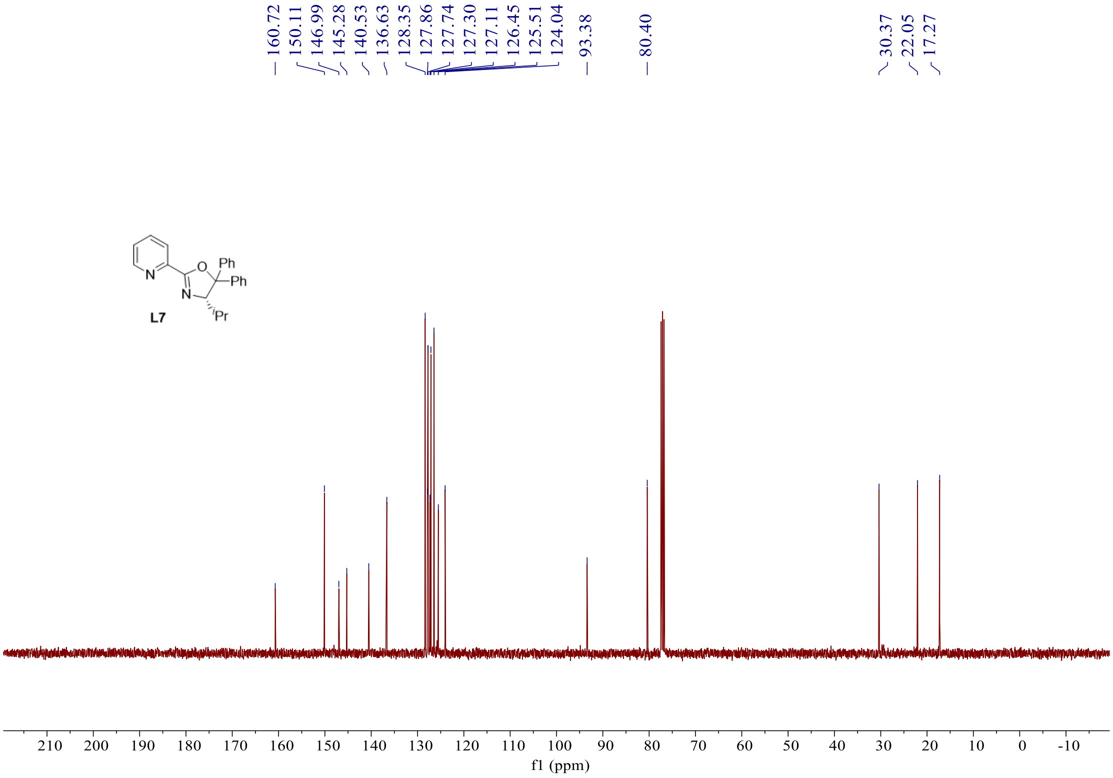

^{1}H NMR of Compound **L8** ($CDCl_3$, 400 MHz, 20 °C):

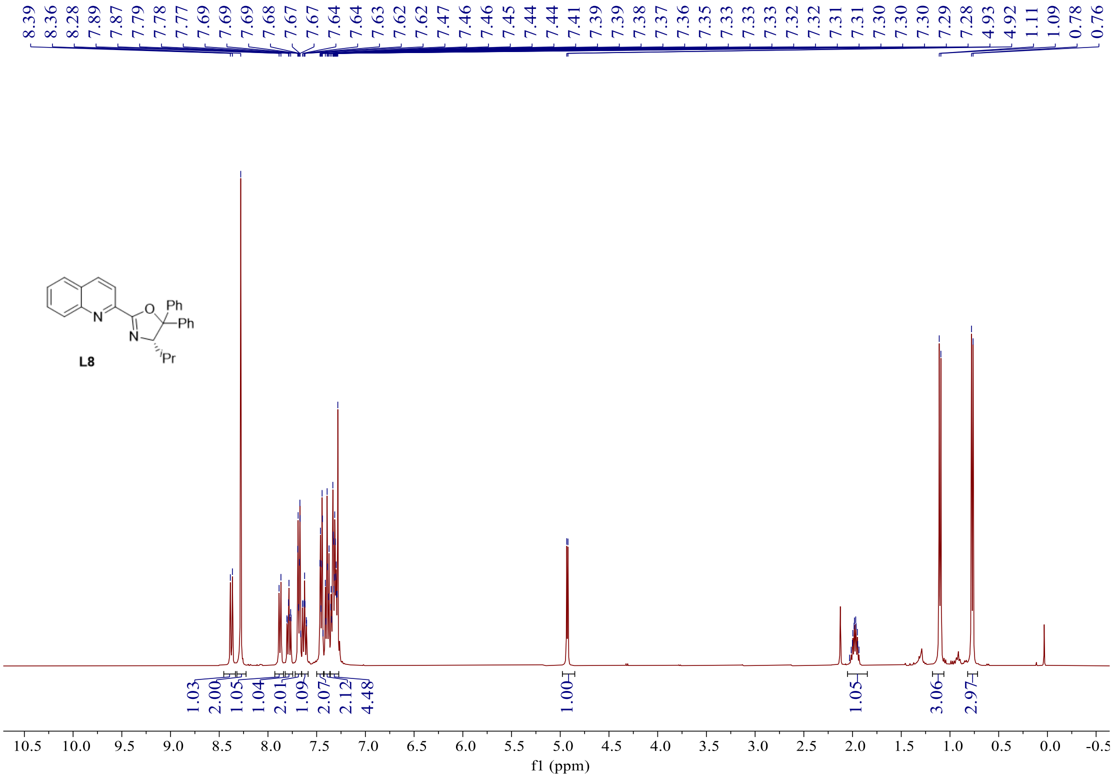

^{13}C NMR of Compound **L8** ($CDCl_3$, 101MHz, 20 °C):

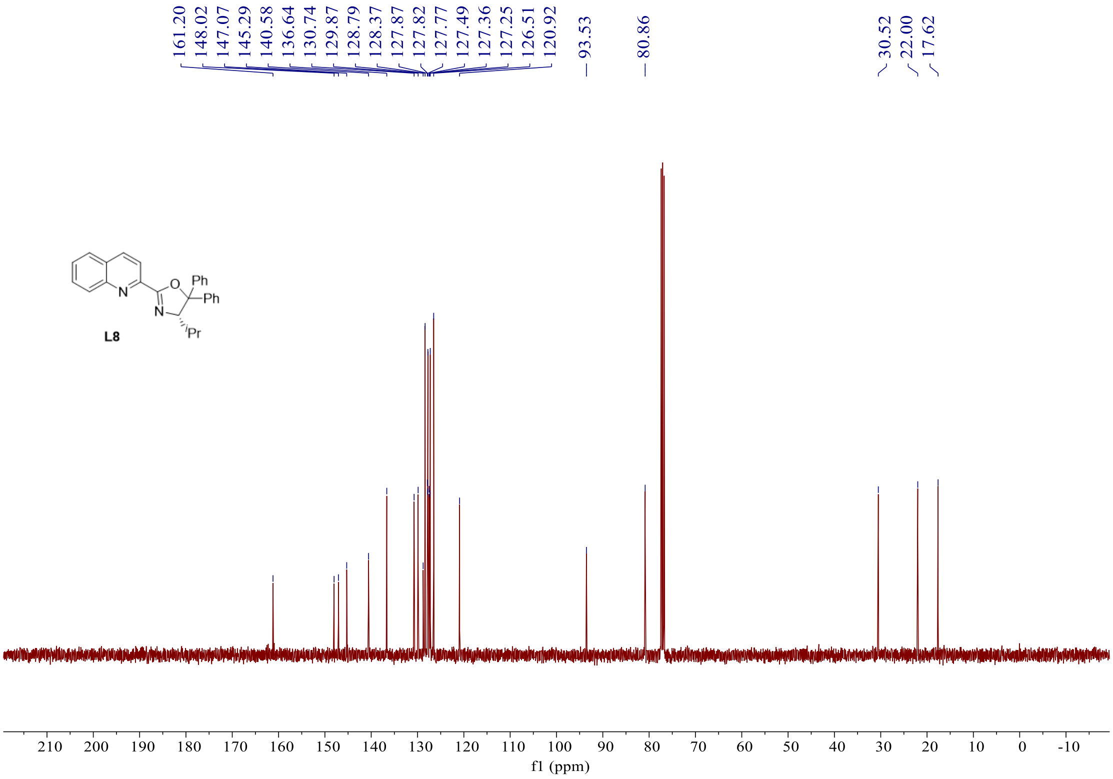

^{1}H NMR of Compound **L9** ($CDCl_3$, 400 MHz, 20 °C):

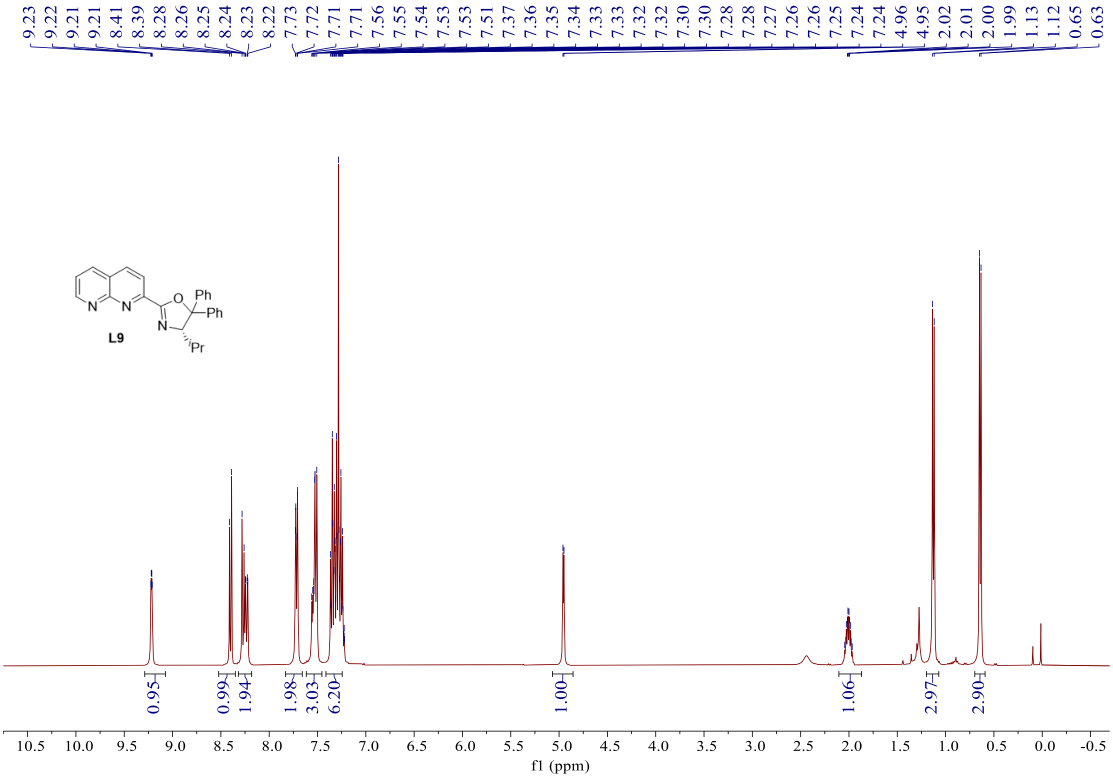

^{13}C NMR of Compound **L9** ($CDCl_3$, 101MHz, 20 °C):

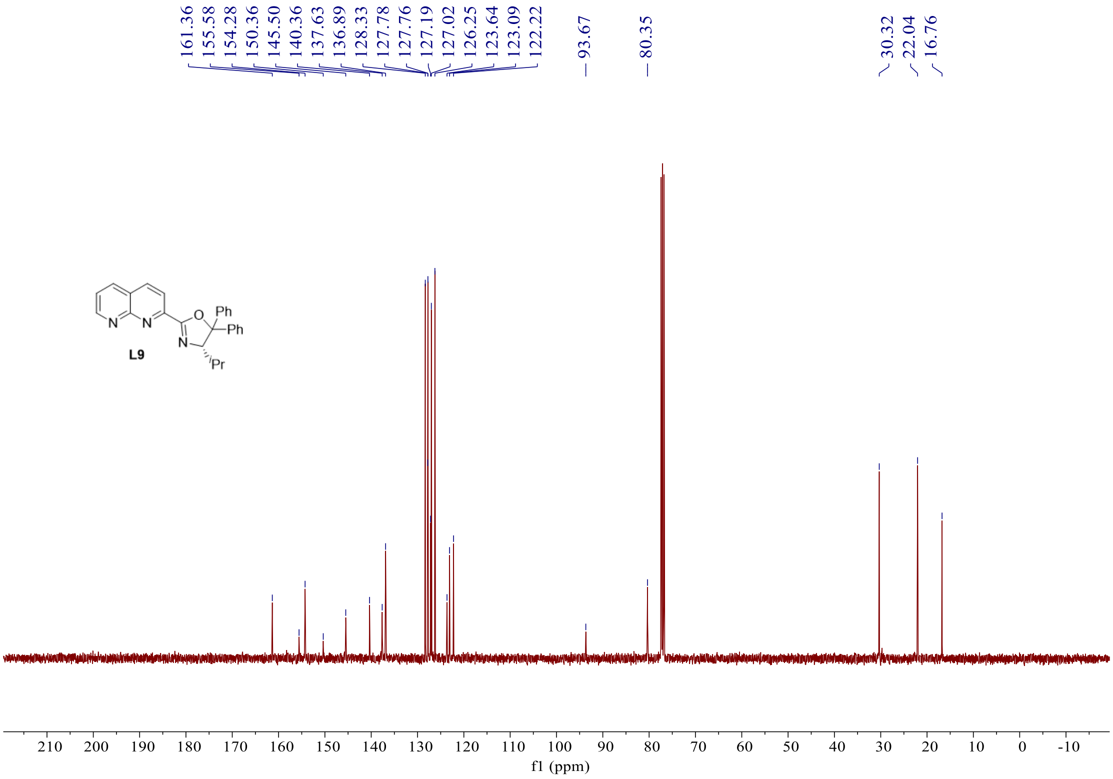

^{1}H NMR of Compound **L11** ($CDCl_3$, 400 MHz, 20 °C):

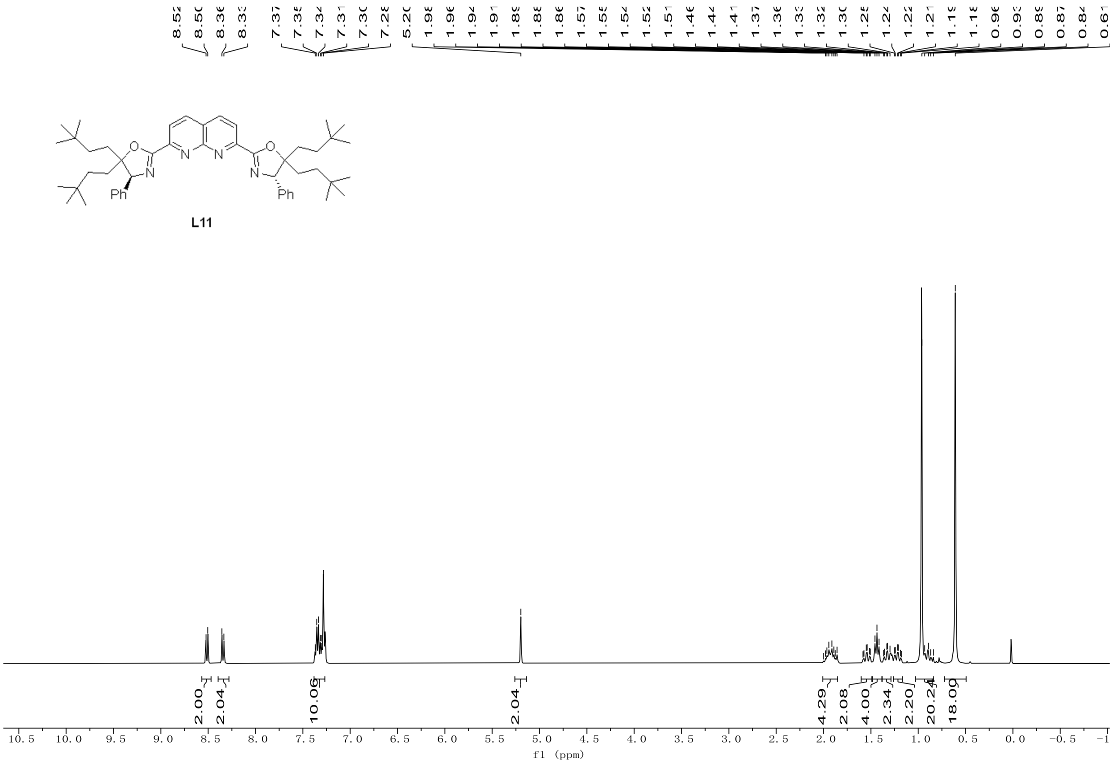

^{13}C NMR of Compound **L11** ($CDCl_3$, 101MHz, 20 °C):

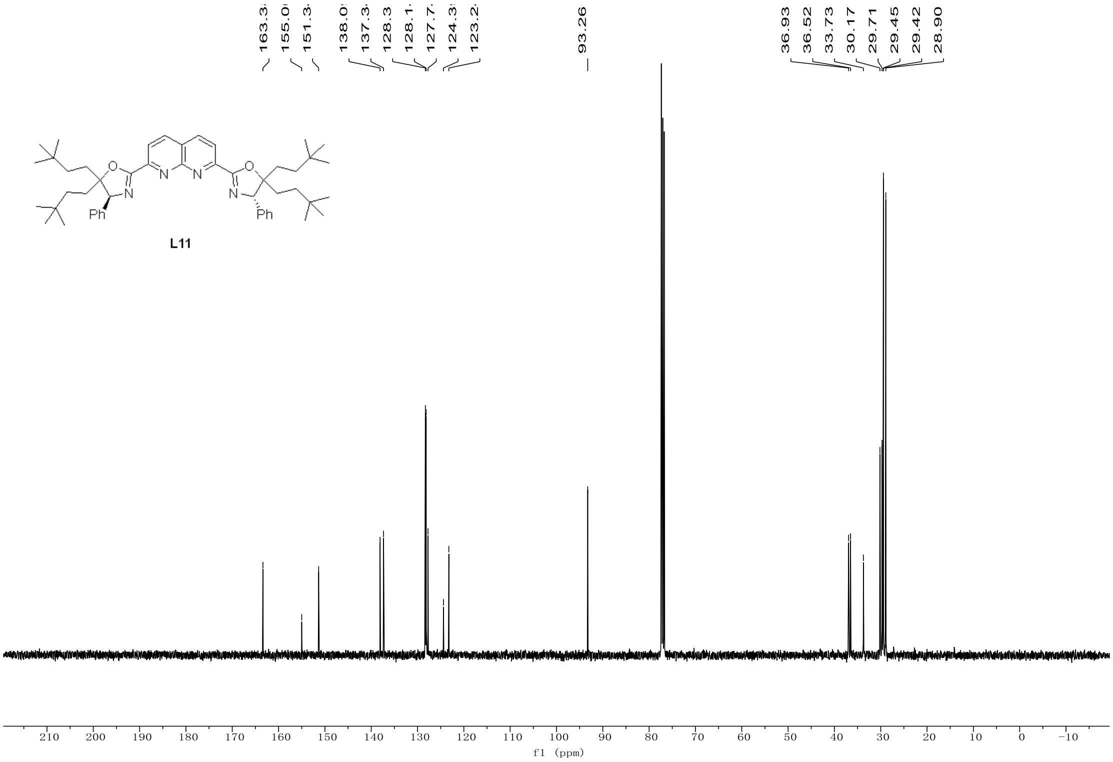

^{1}H NMR of Compound **SL13** ($CDCl_3$, 400 MHz, 20 °C):

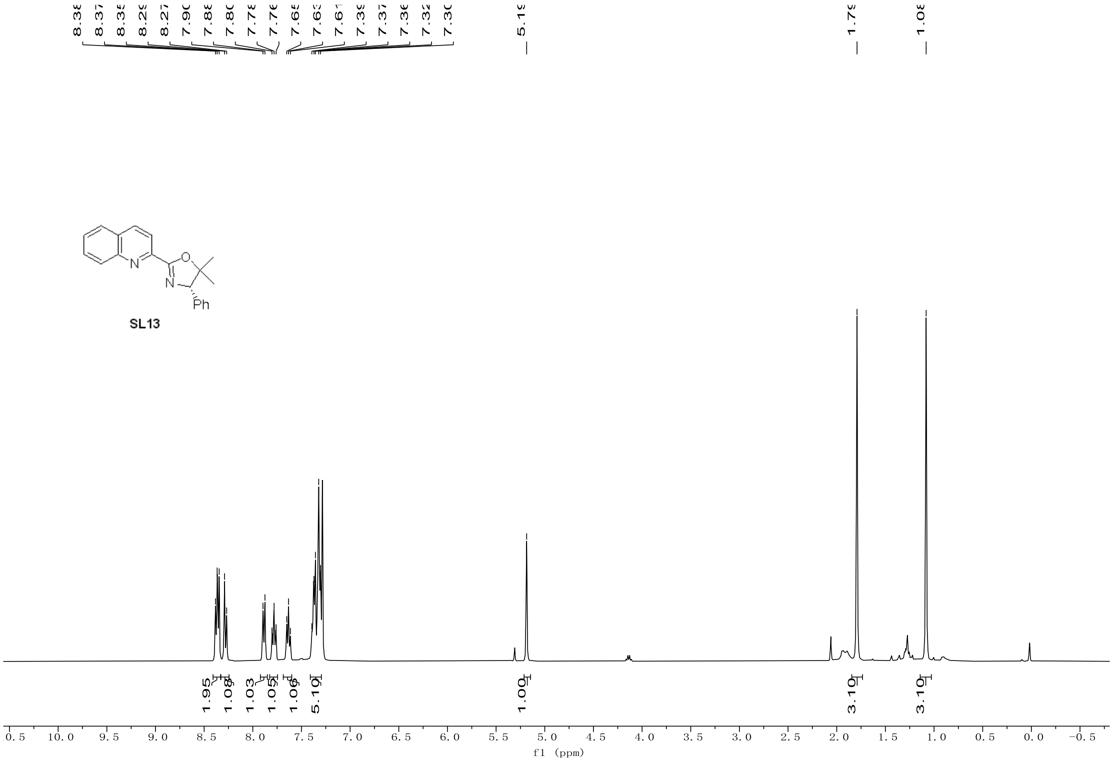

^{13}C NMR of Compound **SL13** ($CDCl_3$, 101MHz, 20 °C):

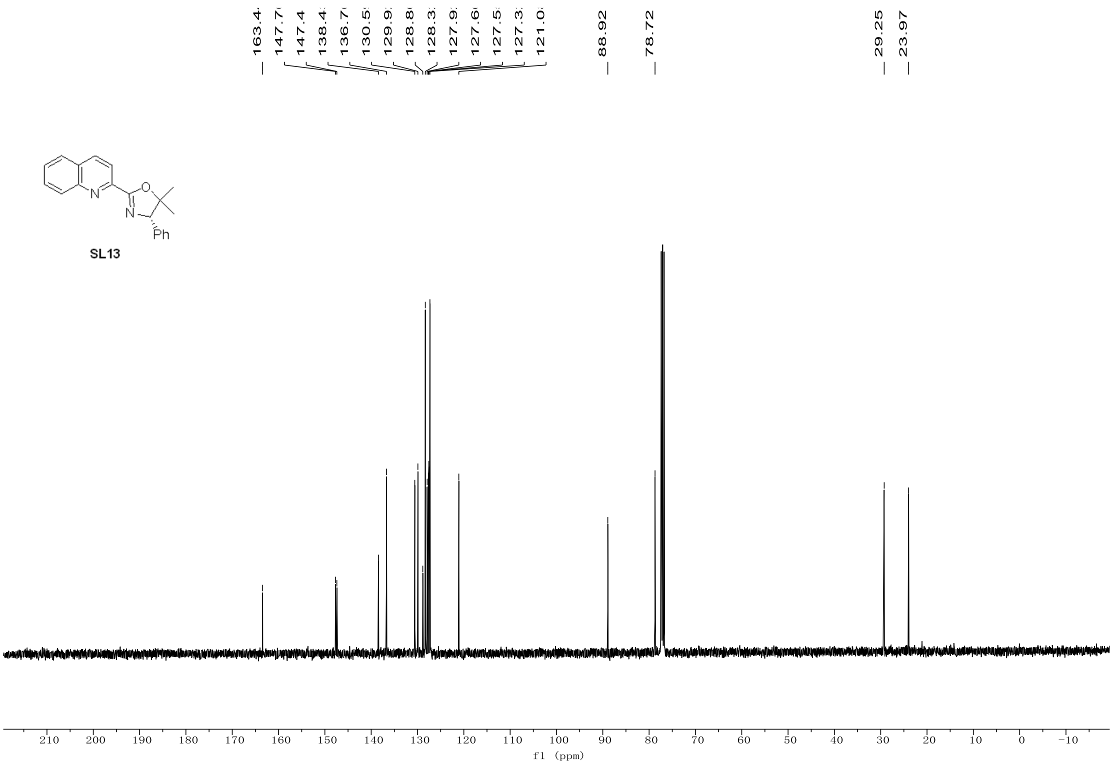

^{1}H NMR of Compound **SL14** ($CDCl_3$, 400 MHz, 20 °C):

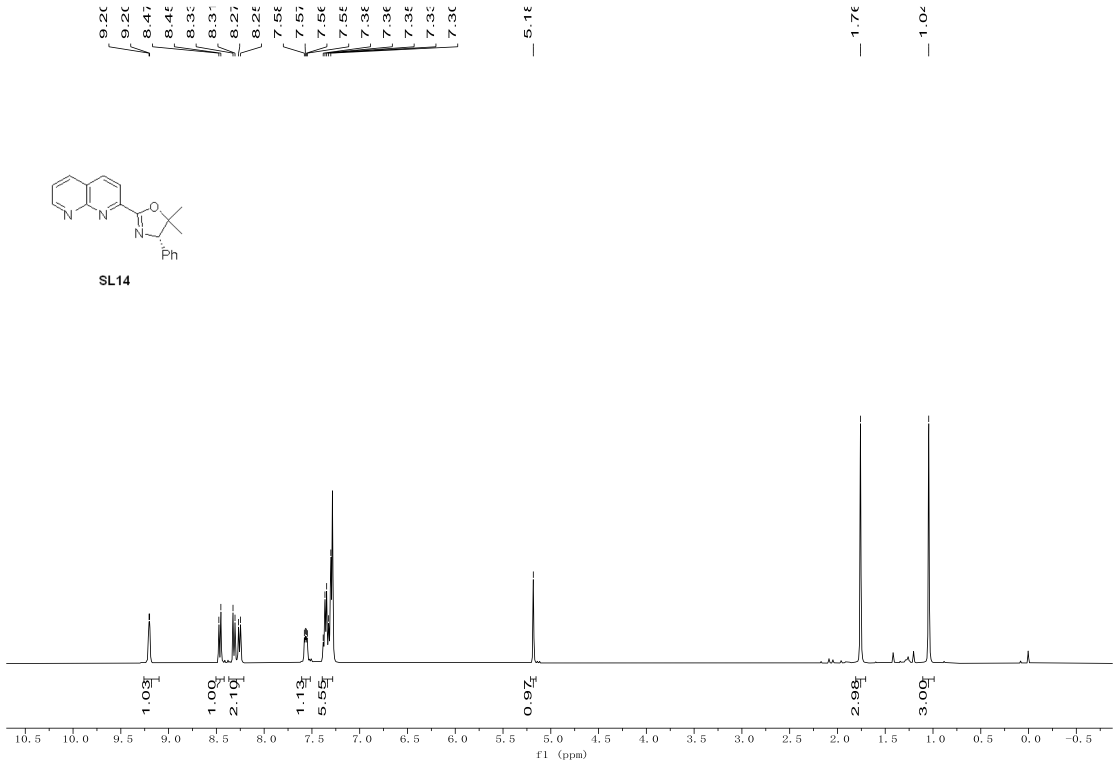

^{13}C NMR of Compound **SL14** ($CDCl_3$, 101MHz, 20 °C):

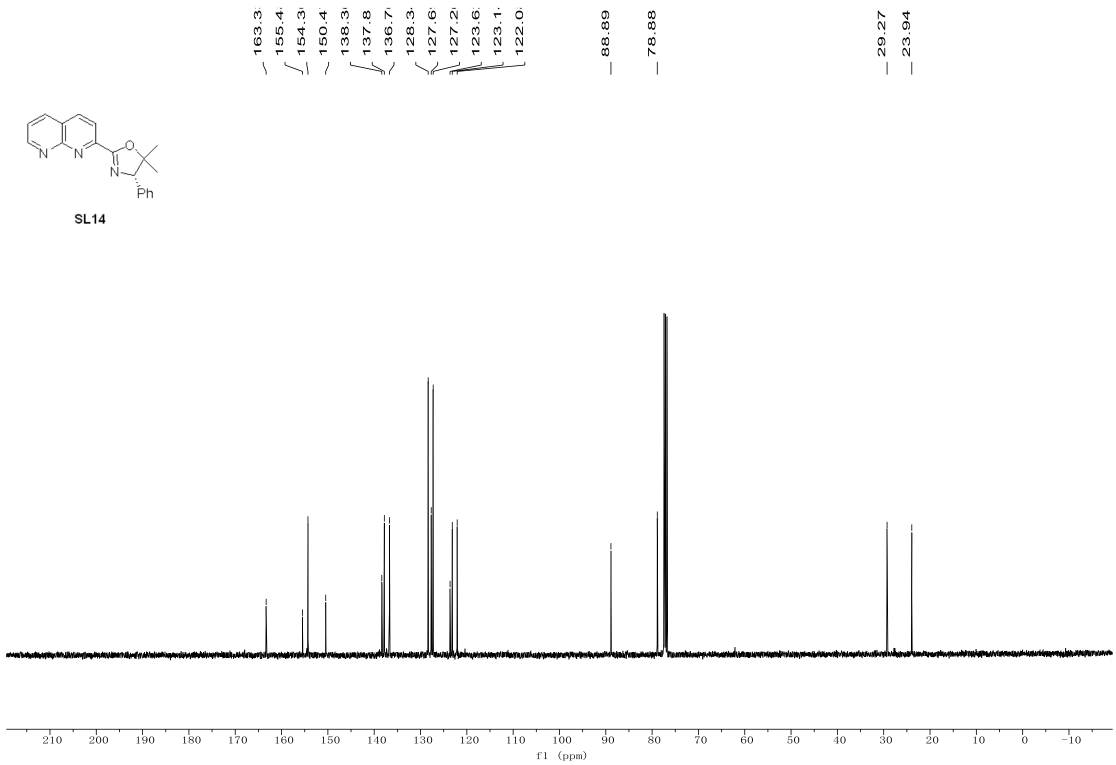

^{1}H NMR of Compound **SL15** ($CDCl_3$, 400 MHz, 20 °C):

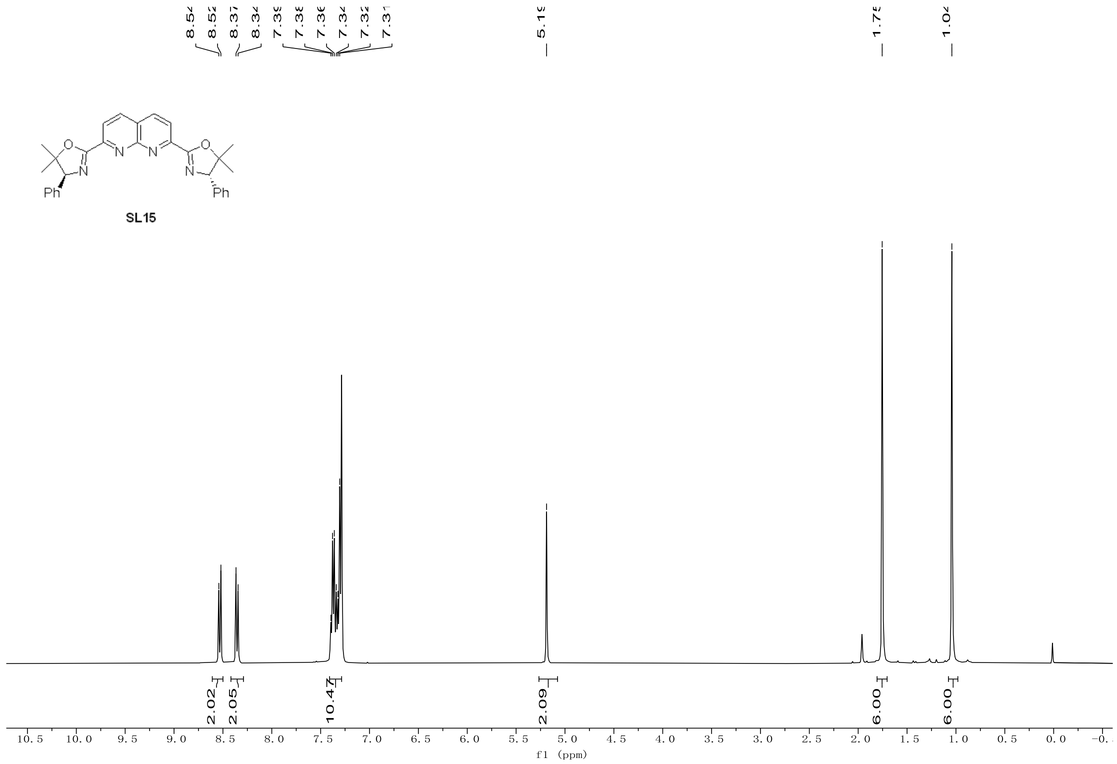

^{13}C NMR of Compound **SL15** ($CDCl_3$, 101MHz, 20 °C):

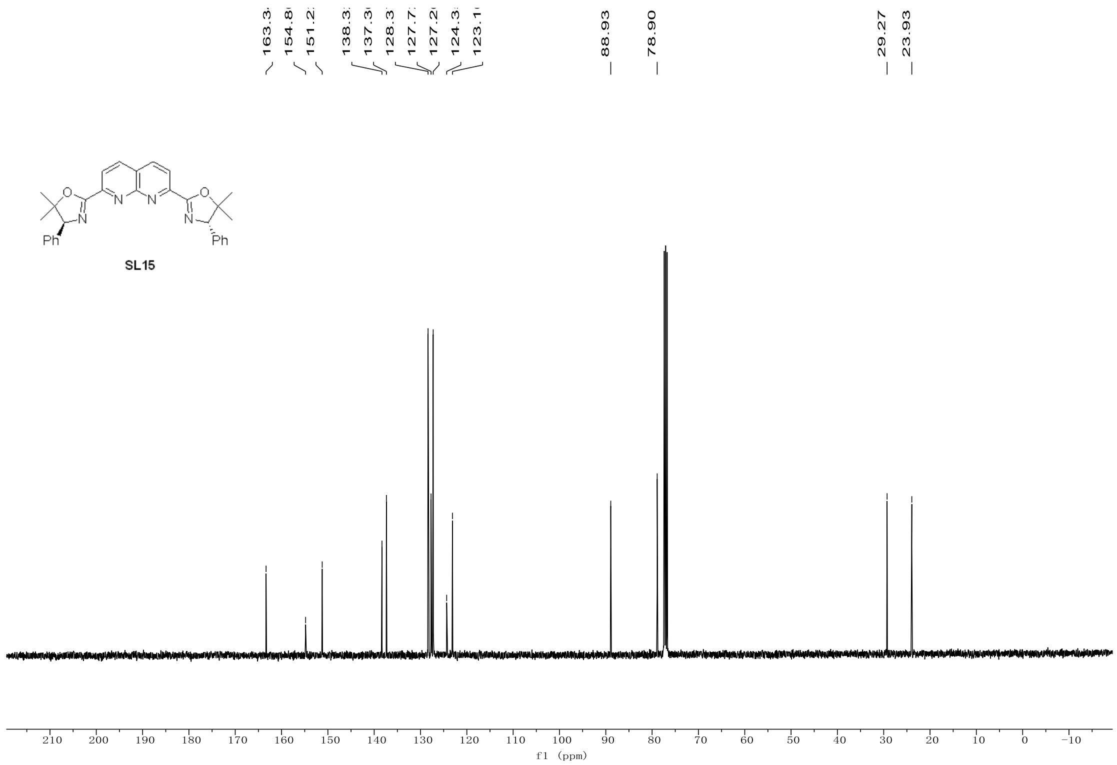

^{1}H NMR of Compound **3** ($CDCl_3$, 400 MHz, 20 °C):

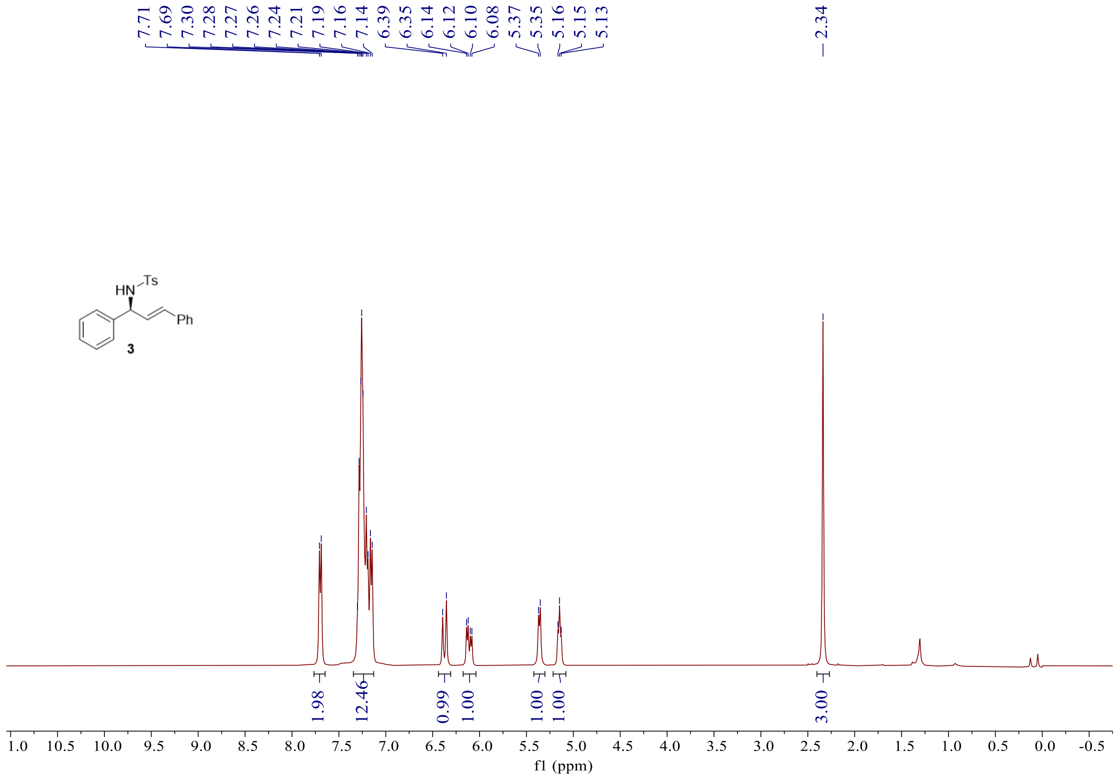

^{13}C NMR of Compound **3** ($CDCl_3$, 101MHz, 20 °C):

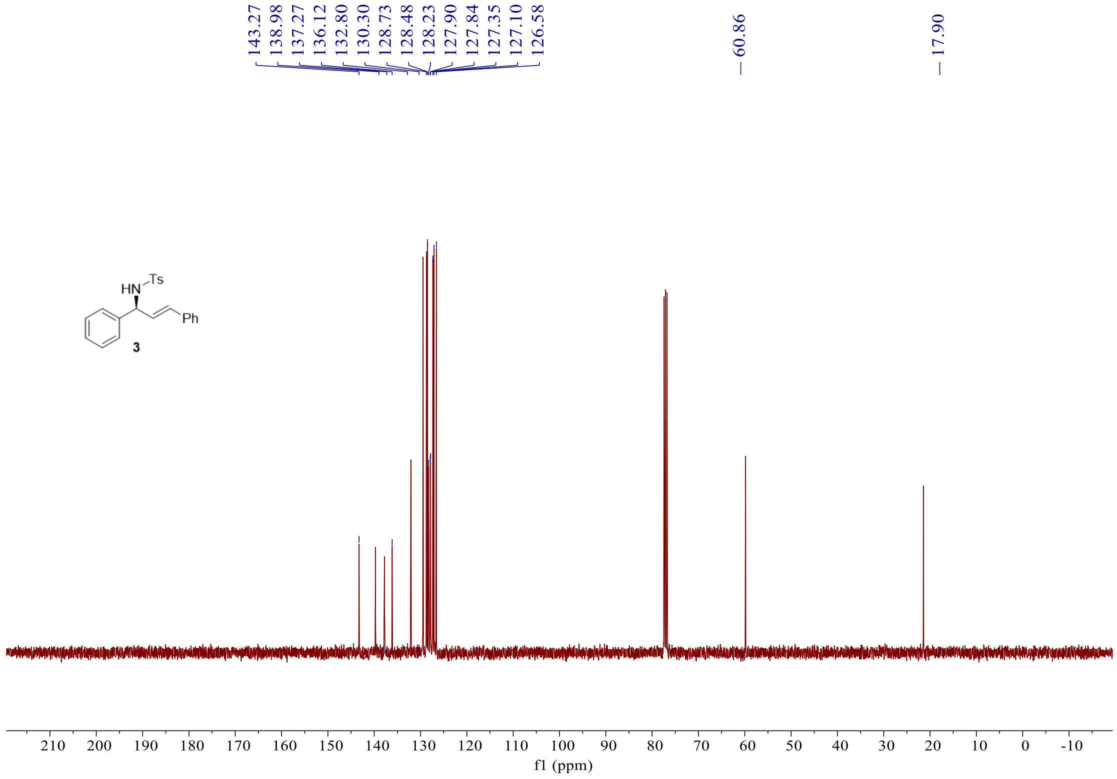

^{1}H NMR of Compound **4** ($CDCl_3$, 400 MHz, 20 °C):

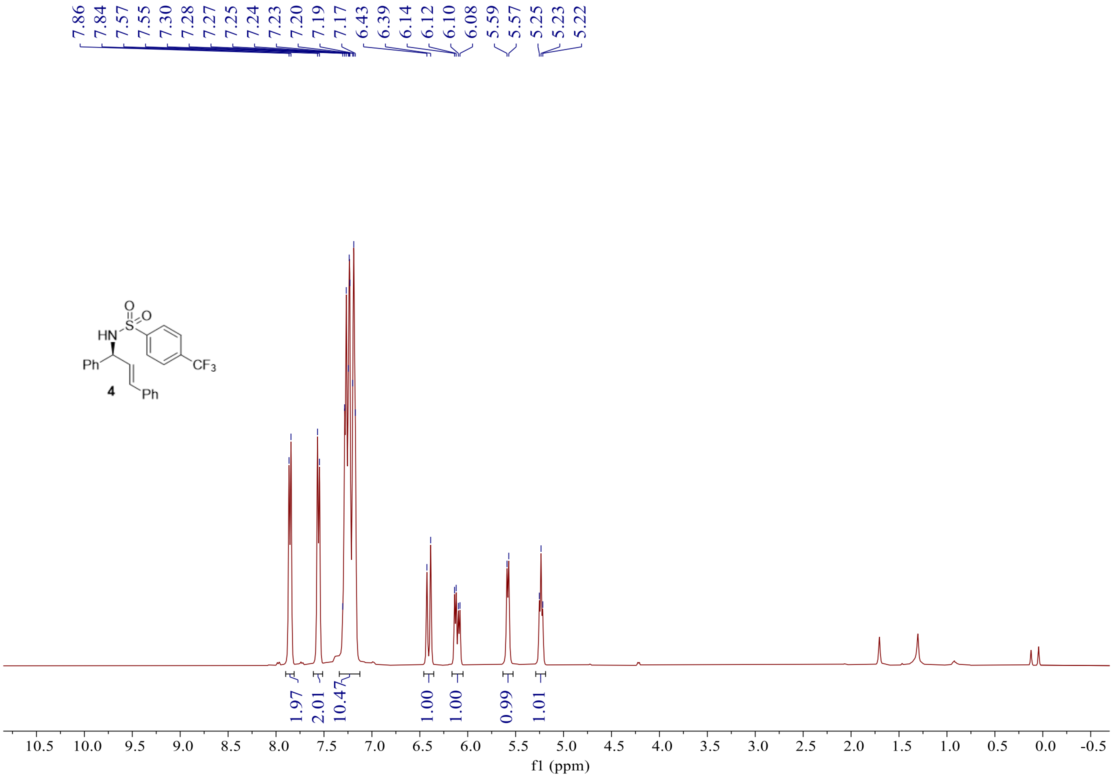

^{13}C NMR of Compound **4** ($CDCl_3$, 101MHz, 20 °C):

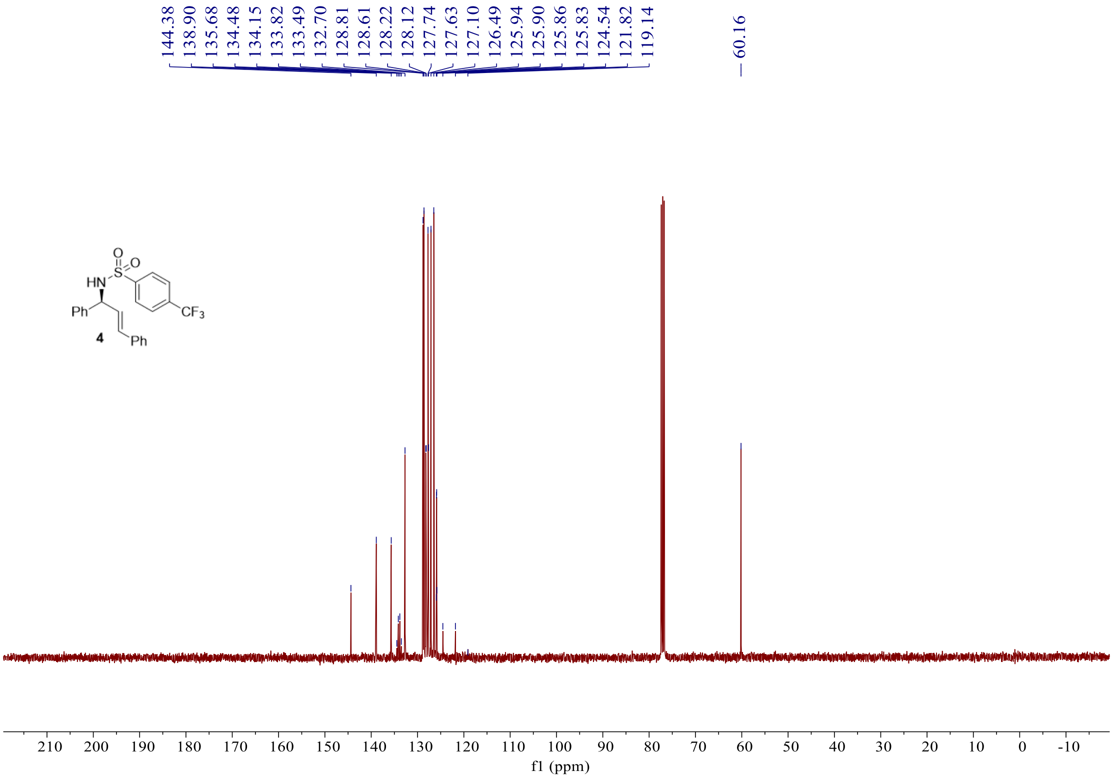

^{19}F NMR of Compound **4** ($CDCl_3$, 376 MHz, 20 °C):

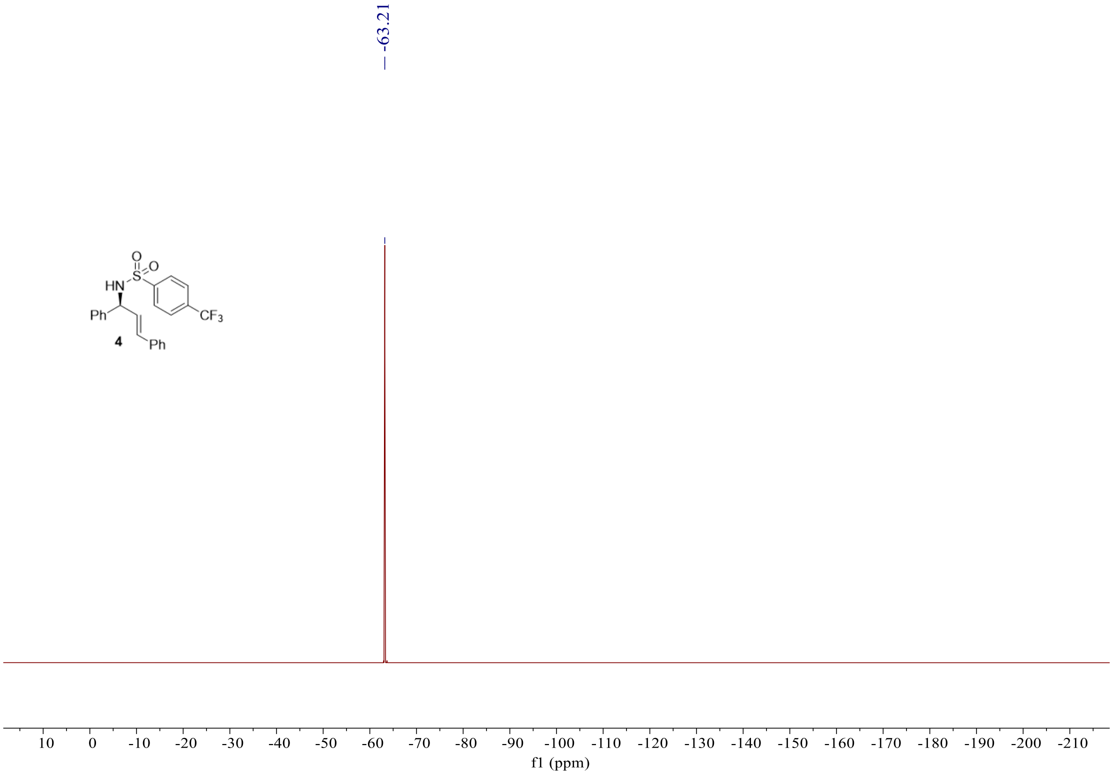

^{1}H NMR of Compound **5** ($CDCl_3$, 400 MHz, 20 °C):

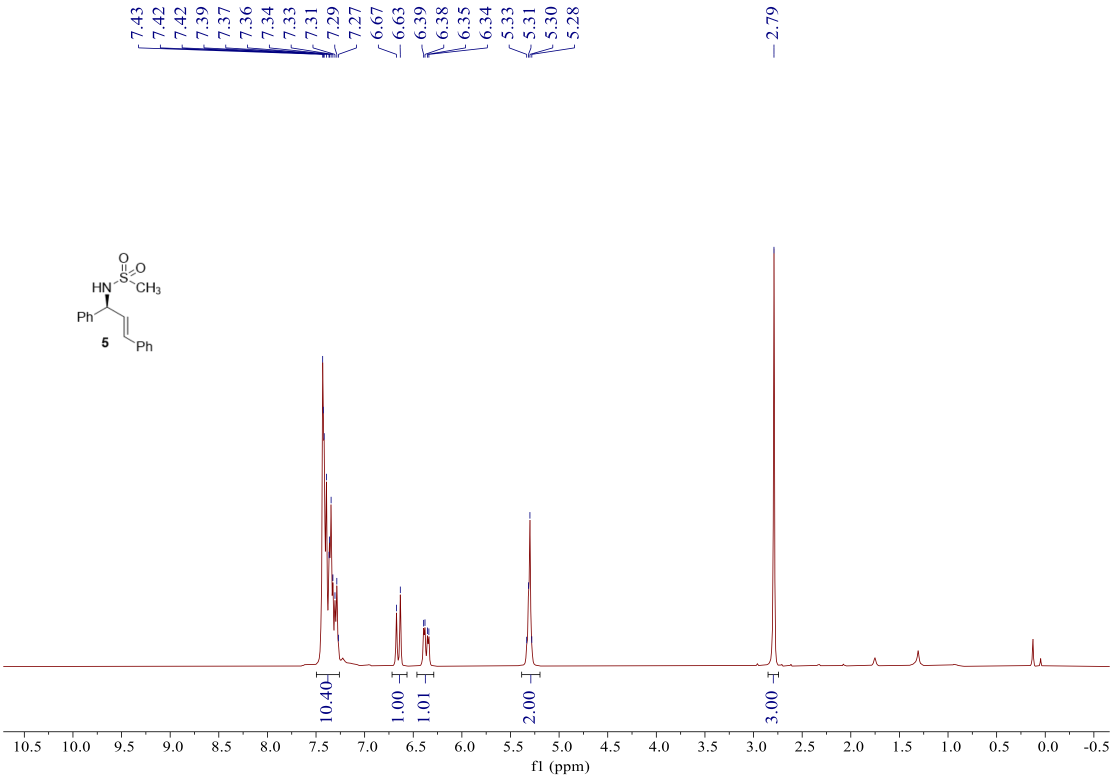

^{13}C NMR of Compound **5** ($CDCl_3$, 101 MHz, 20 °C):

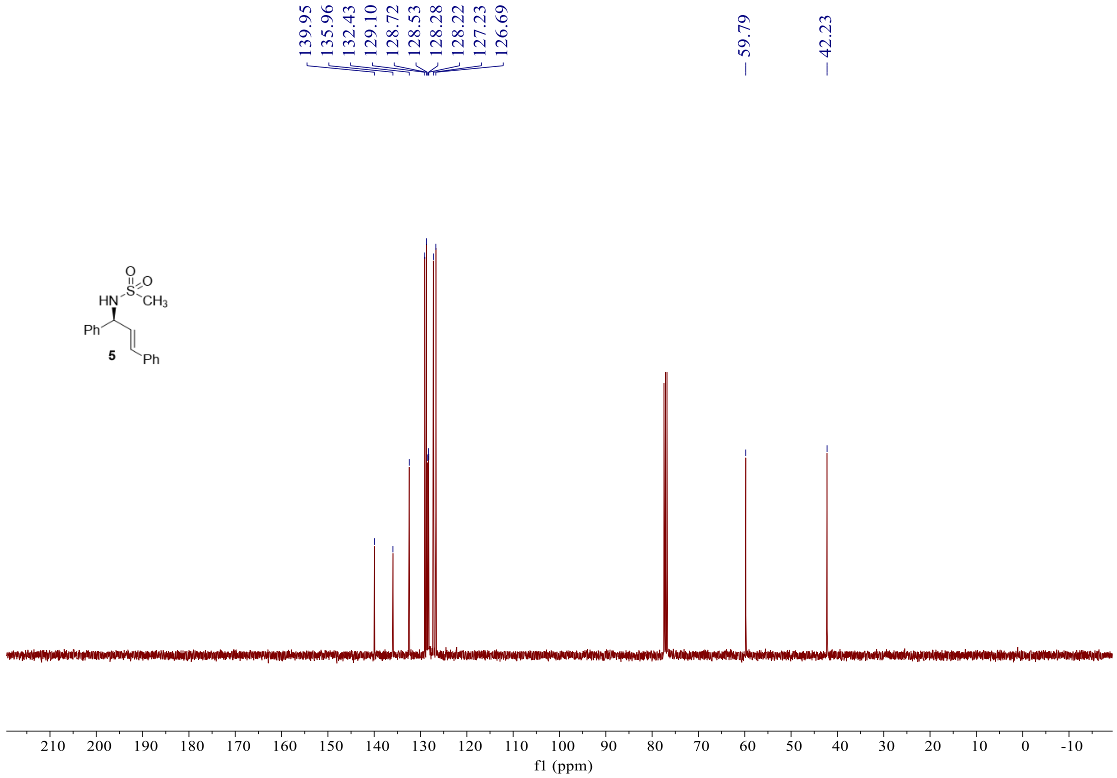

^{1}H NMR of Compound **6** ($CDCl_3$, 400 MHz, 20 °C):

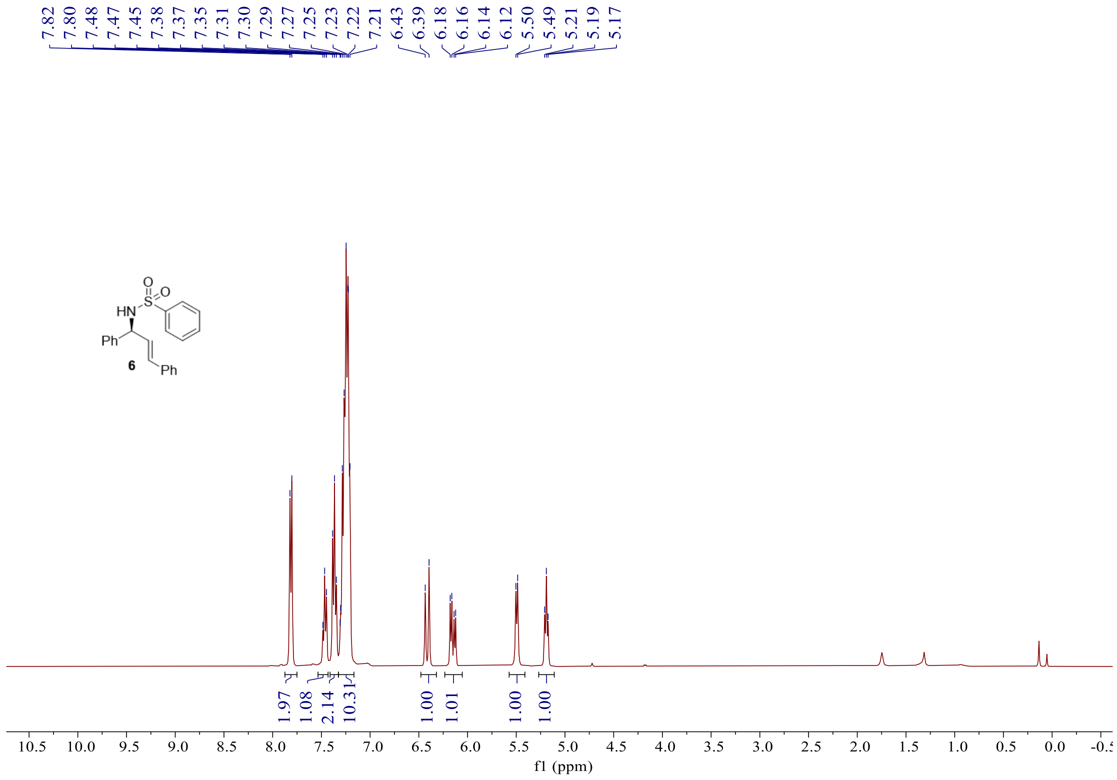

^{13}C NMR of Compound **6** ($CDCl_3$, 101 MHz, 20 °C):

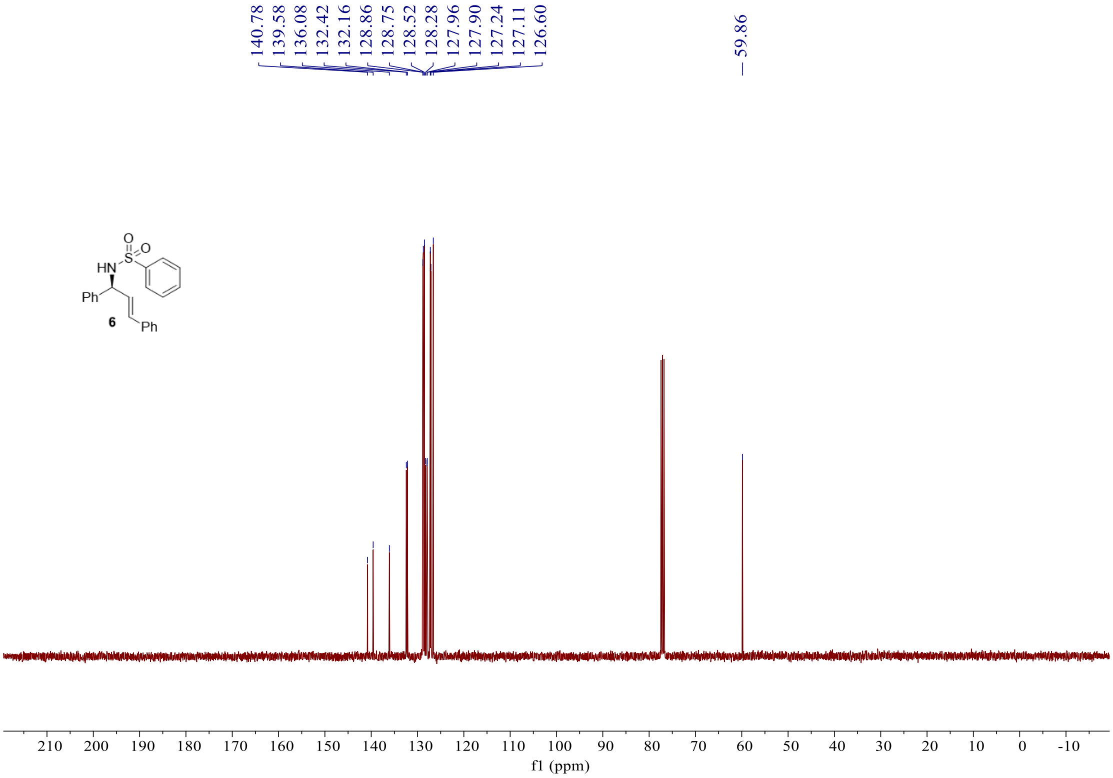

^{1}H NMR of Compound **7** ($CDCl_3$, 400 MHz, 20 °C):

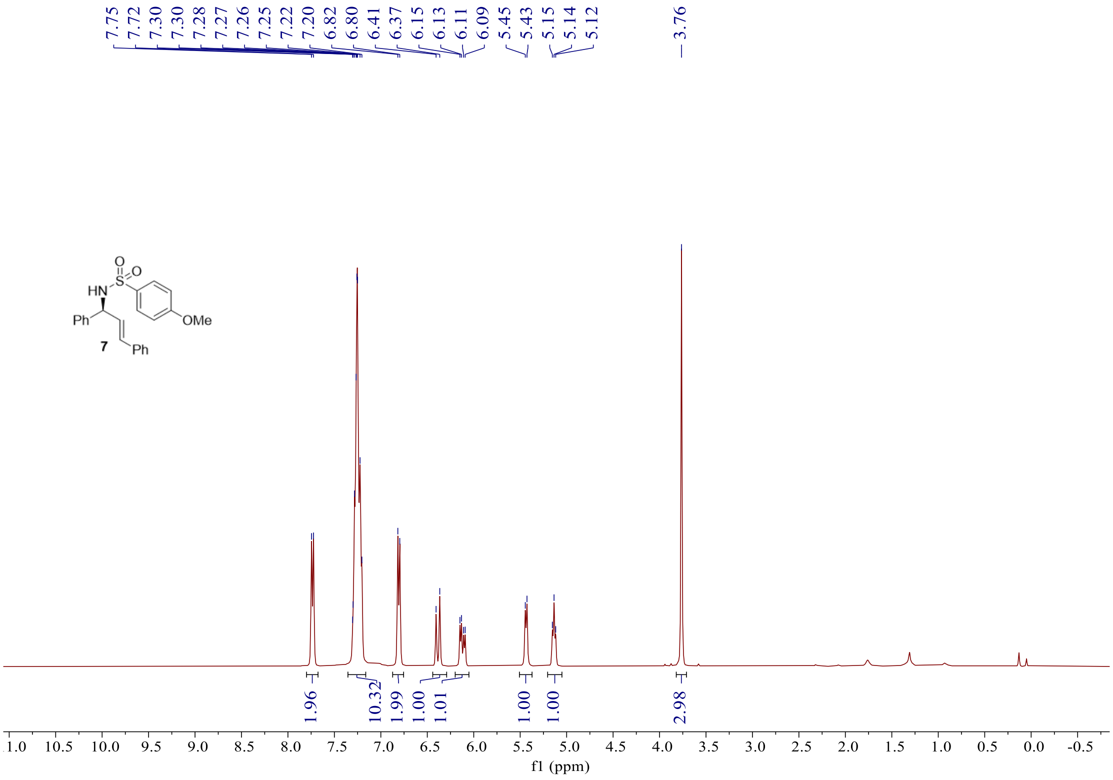

^{13}C NMR of Compound **7** ($CDCl_3$, 101 MHz, 20 °C):

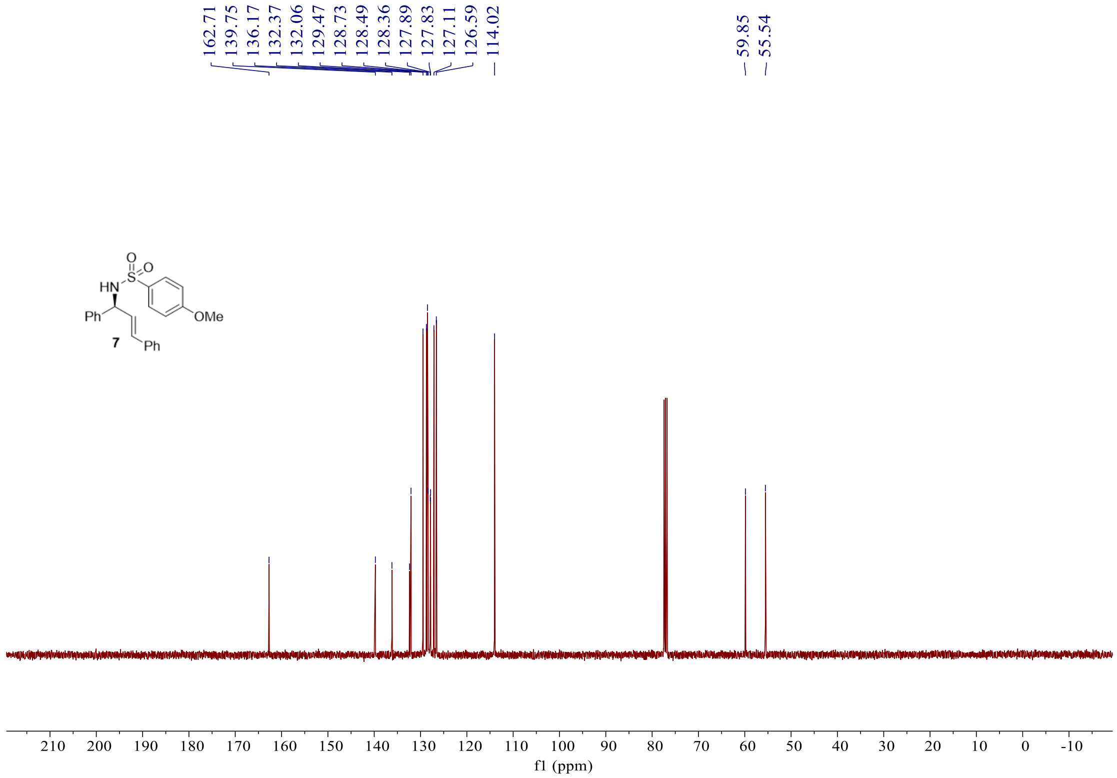

^{1}H NMR of Compound **8** ($CDCl_3$, 400 MHz, 20 °C):

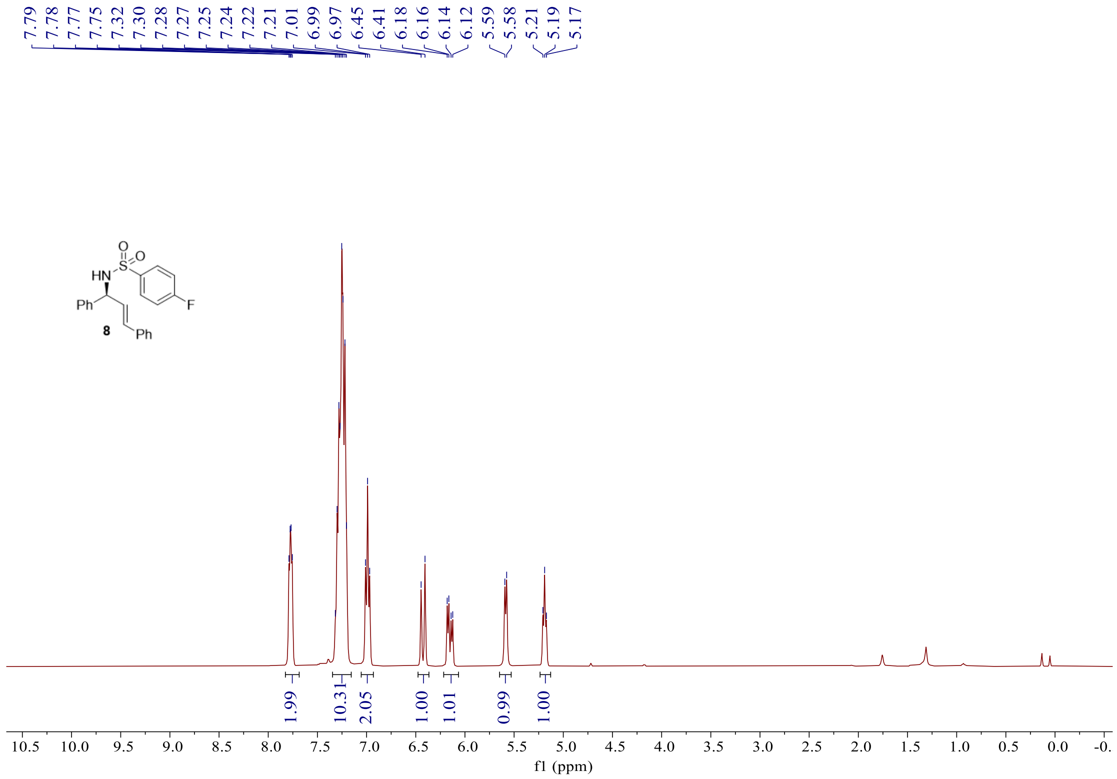

^{13}C NMR of Compound **8** ($CDCl_3$, 101 MHz, 20 °C):

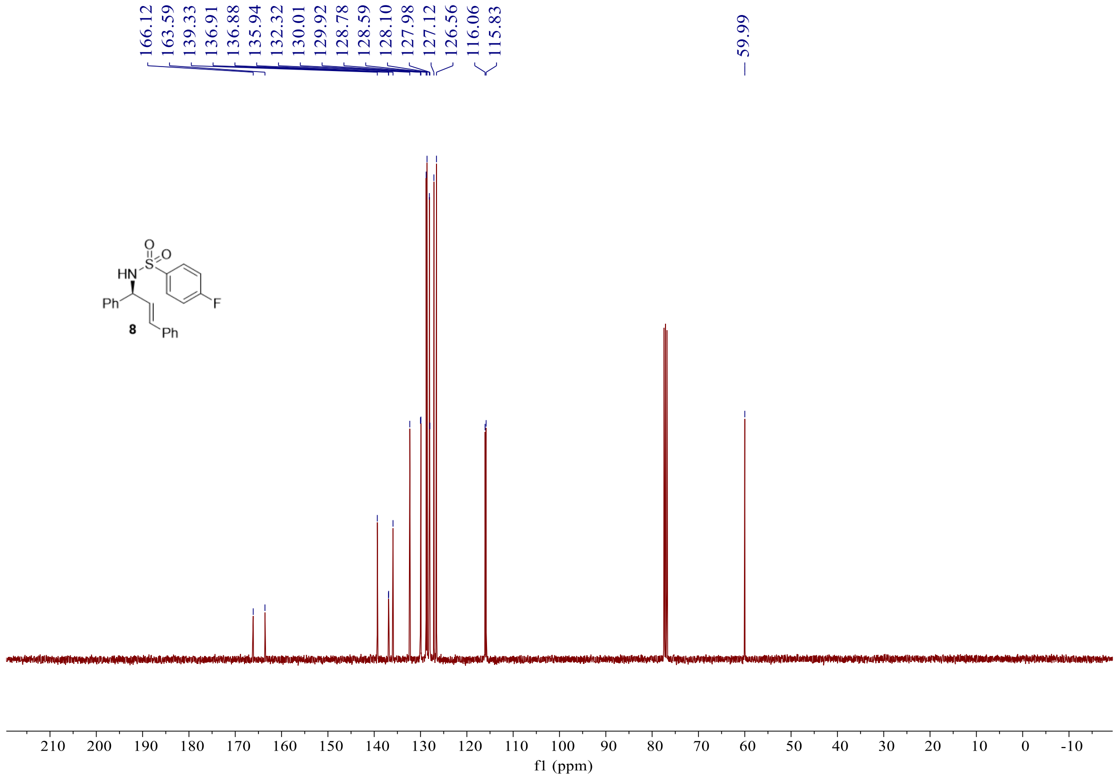

^{19}F NMR of Compound **8** ($CDCl_3$, 376 MHz, 20 °C):

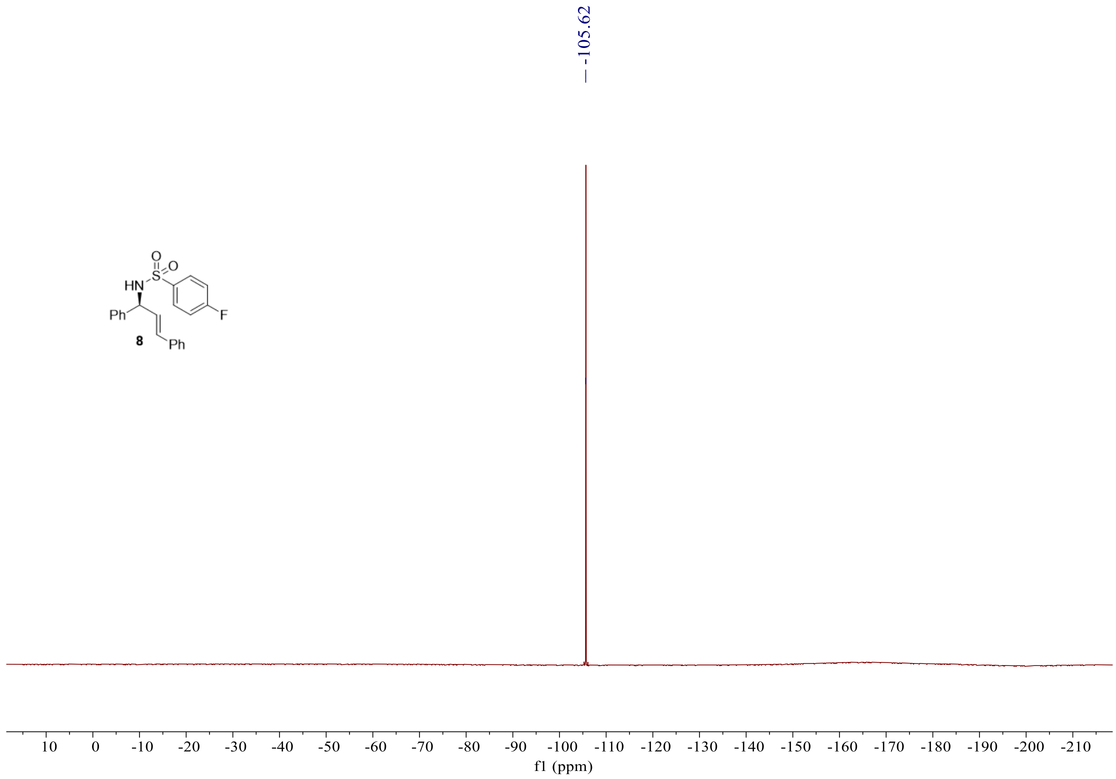

^1H NMR of Compound **9** ($CDCl_3$, 400 MHz, 20 °C):

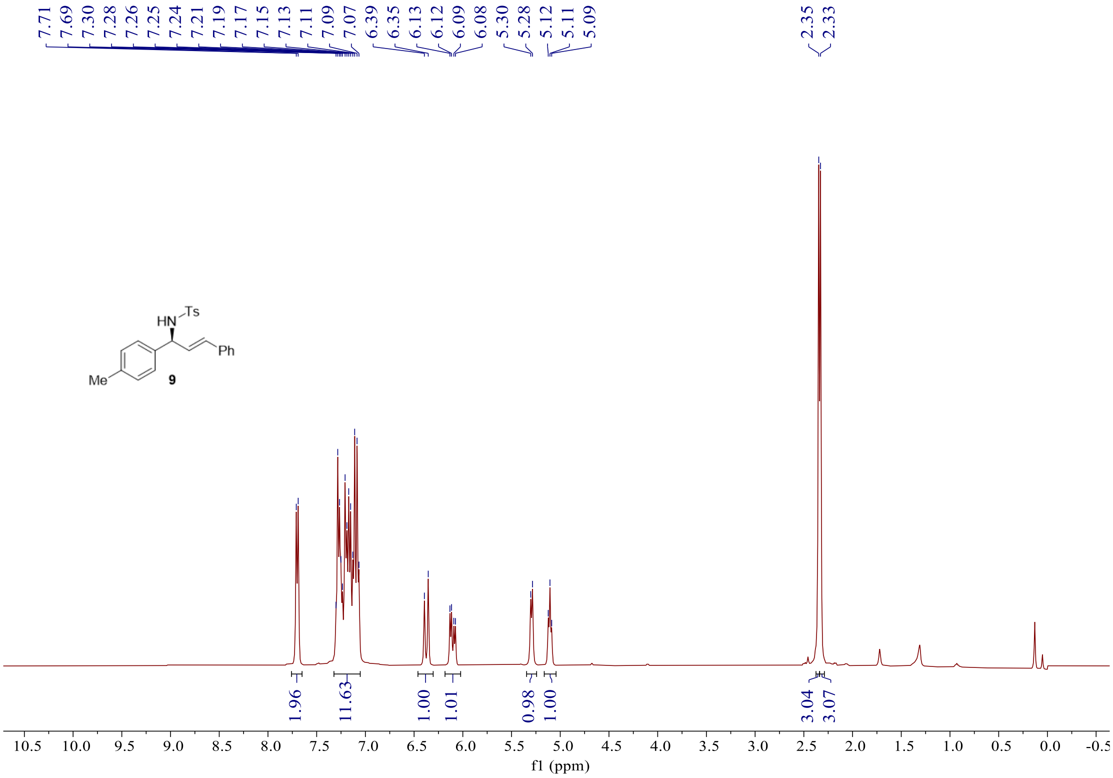

^{13}C NMR of Compound **9** ($CDCl_3$, 101 MHz, 20 °C):

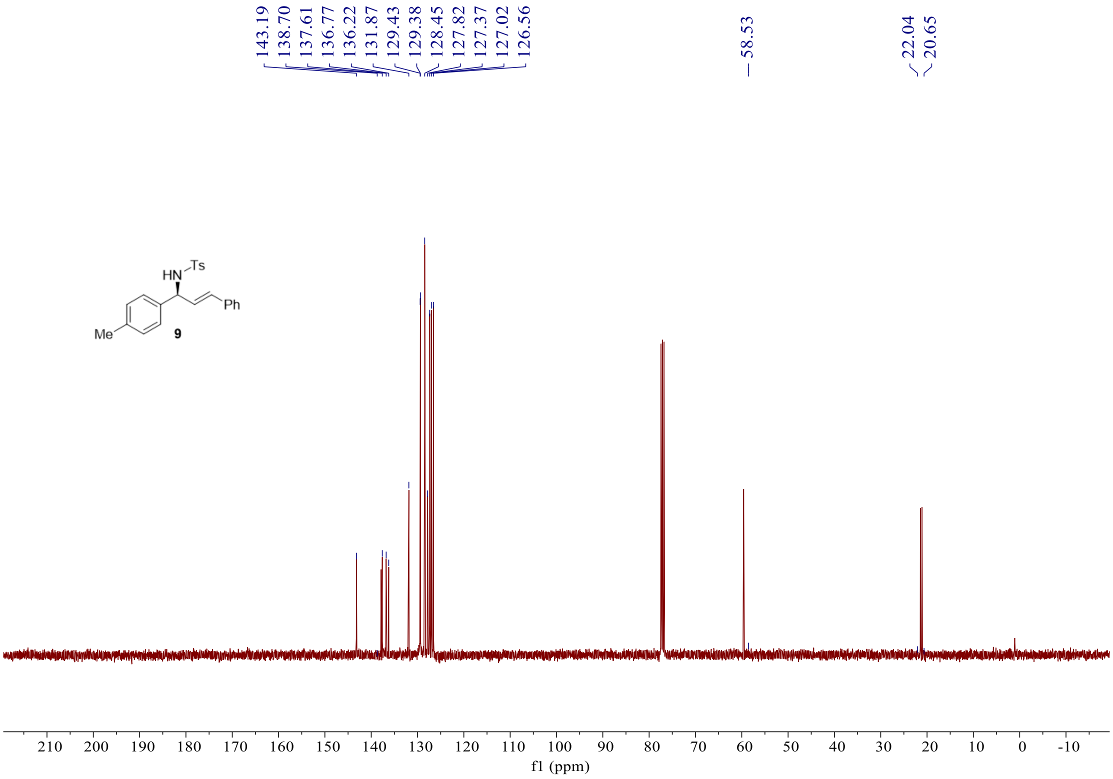

^{1}H NMR of Compound **10** ($CDCl_3$, 400 MHz, 20 °C):

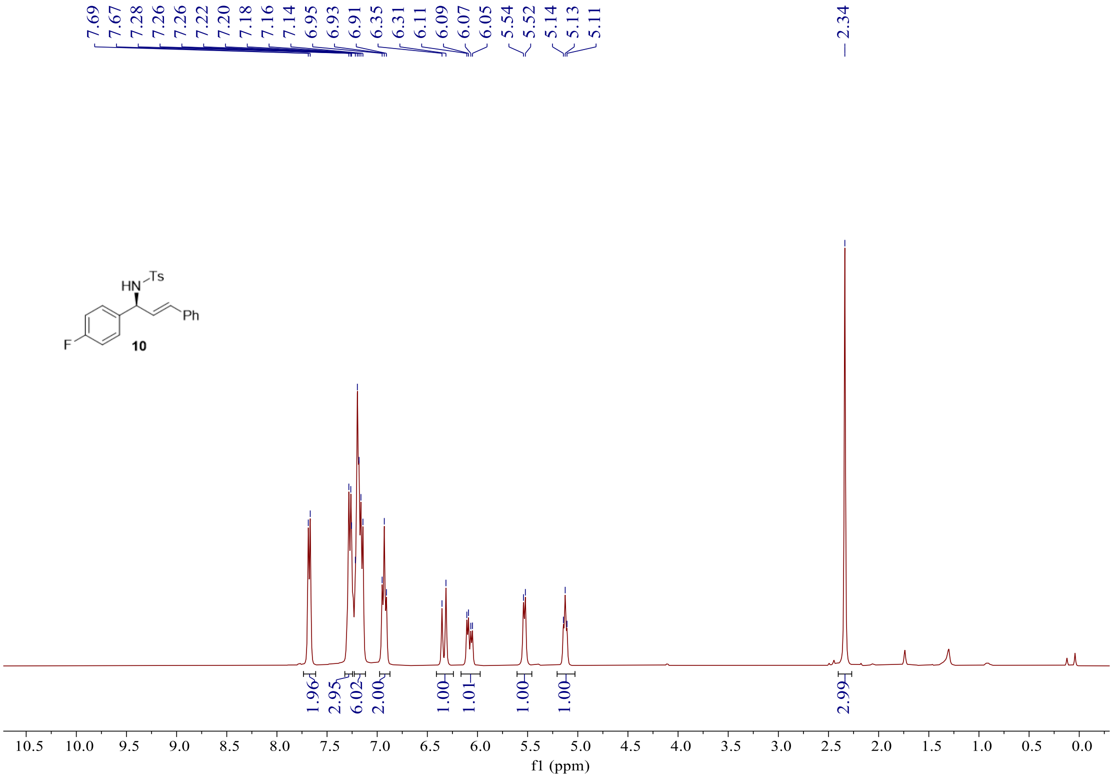

^{13}C NMR of Compound **10** ($CDCl_3$, 101 MHz, 20 °C):

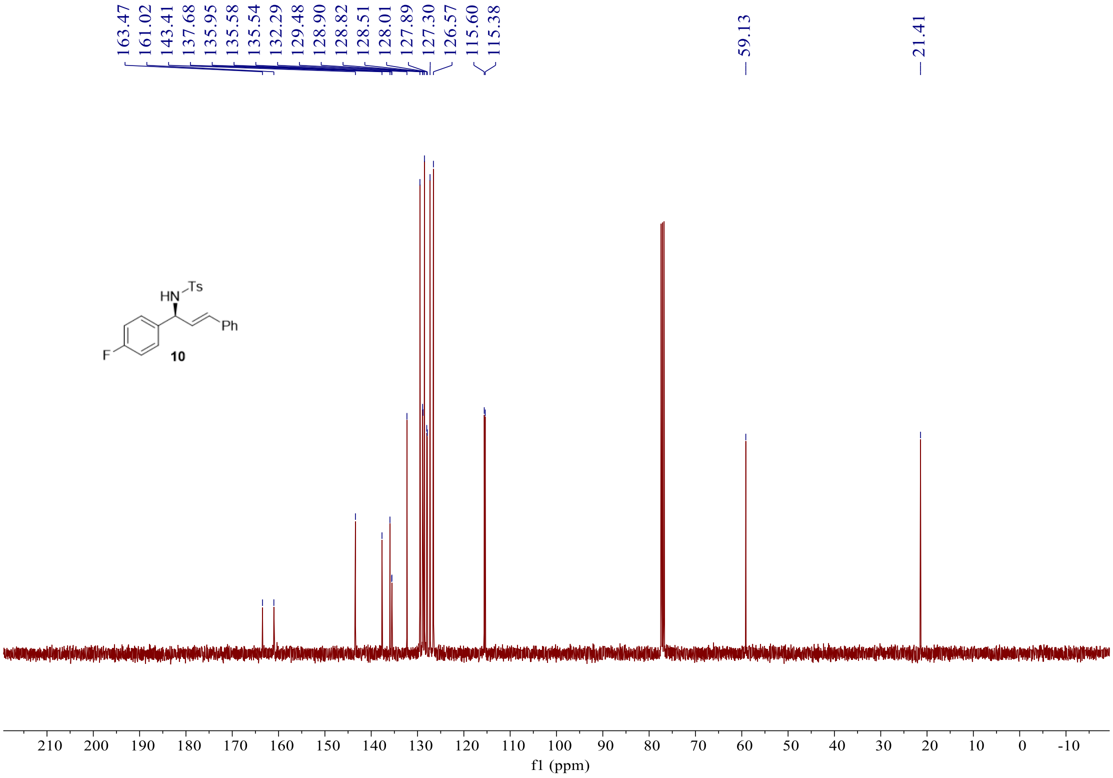

^{19}F NMR of Compound **10** ($CDCl_3$, 376MHz, 20 °C):

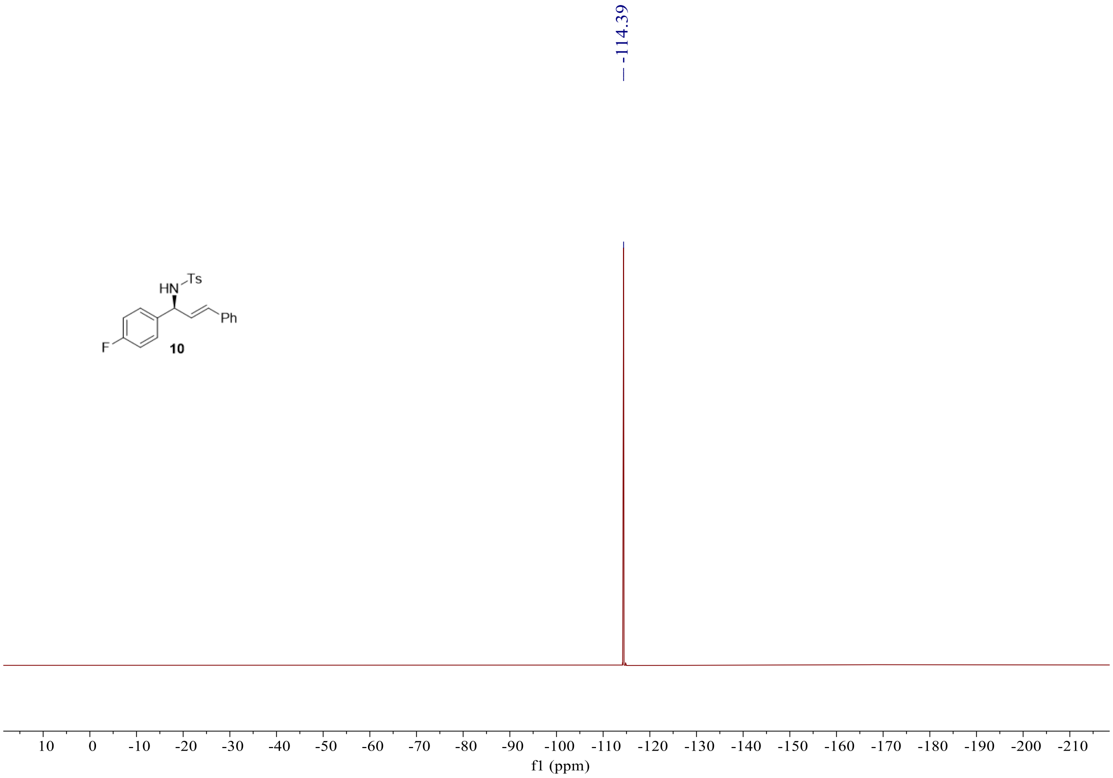

^{1}H NMR of Compound **11** ($CDCl_3$, 400 MHz, 20 °C):

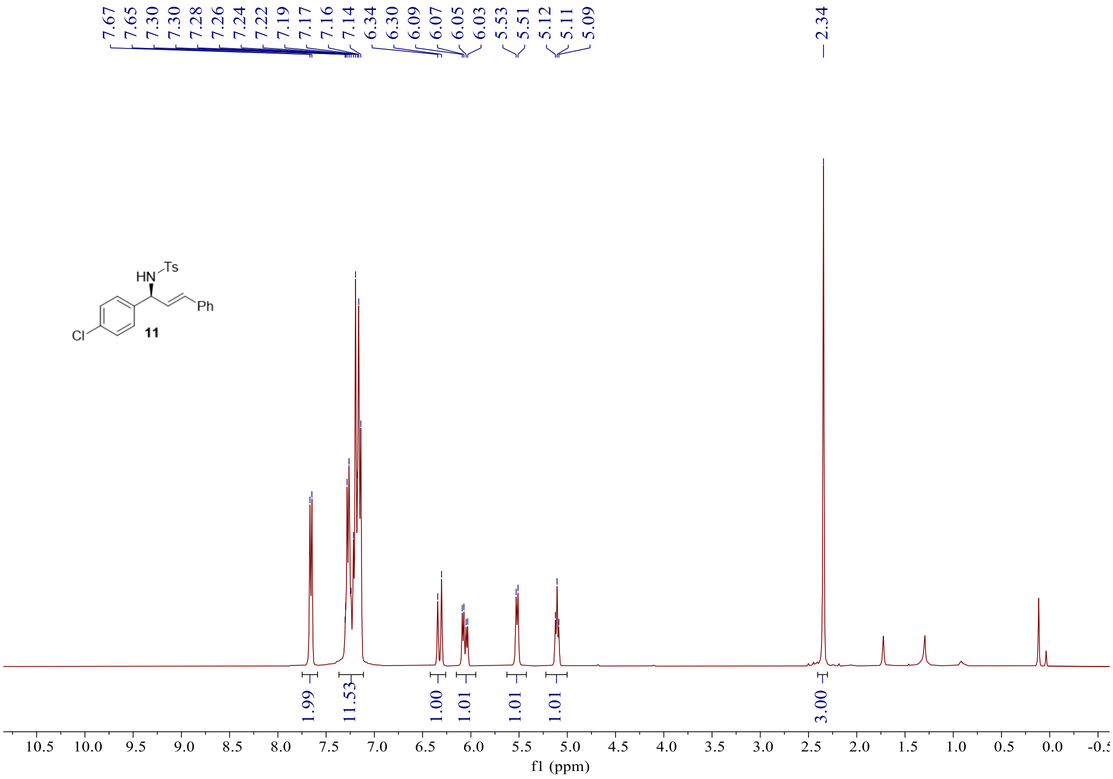

^{13}C NMR of Compound **11** ($CDCl_3$, 101MHz, 20 °C):

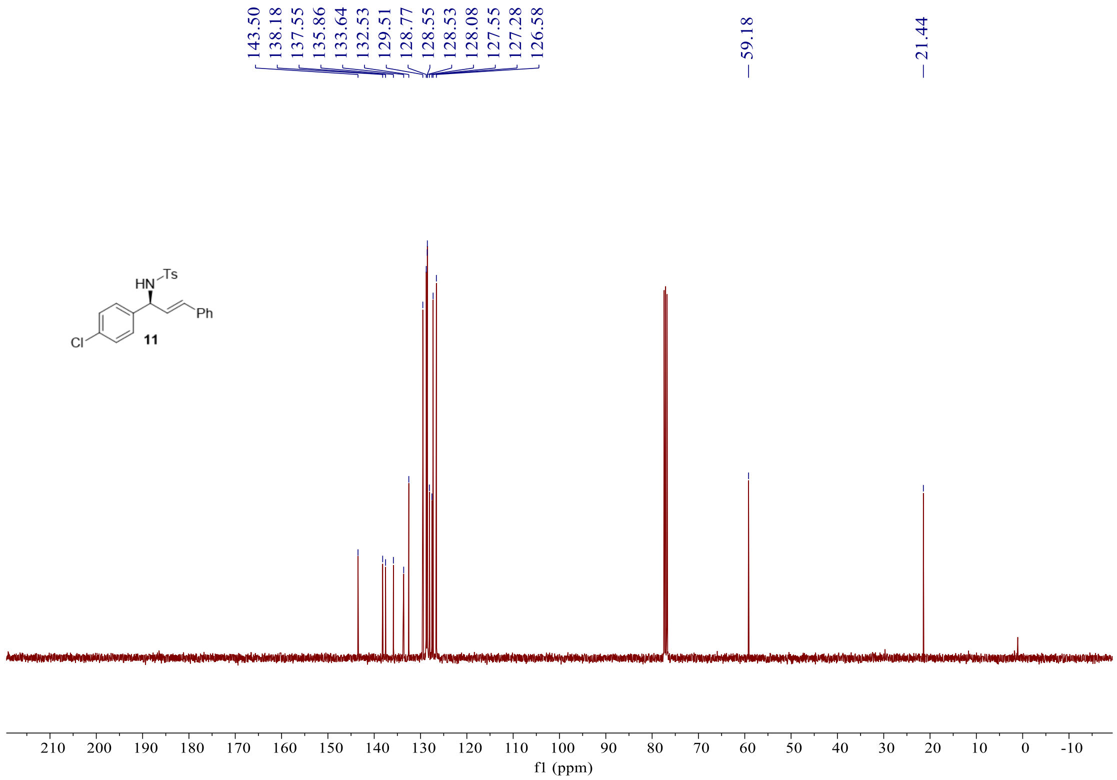

^{1}H NMR of Compound **12** ($CDCl_3$, 400 MHz, 20 °C):

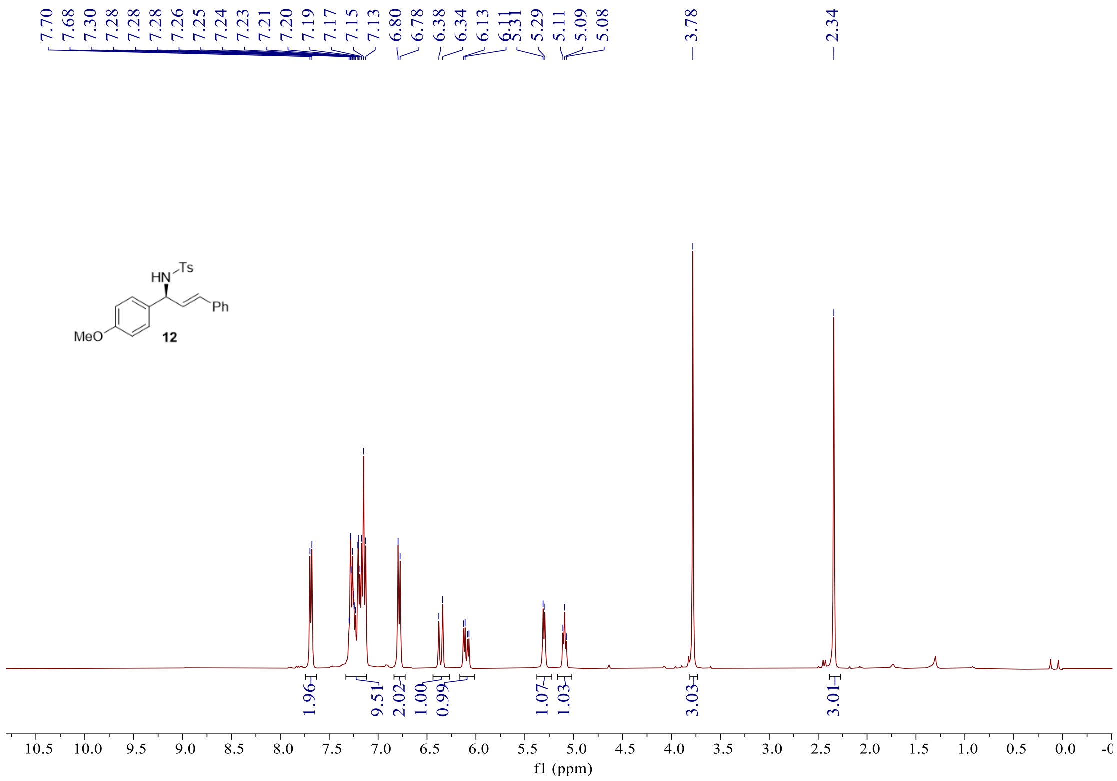

^{13}C NMR of Compound **12** ($CDCl_3$, 101MHz, 20 °C):

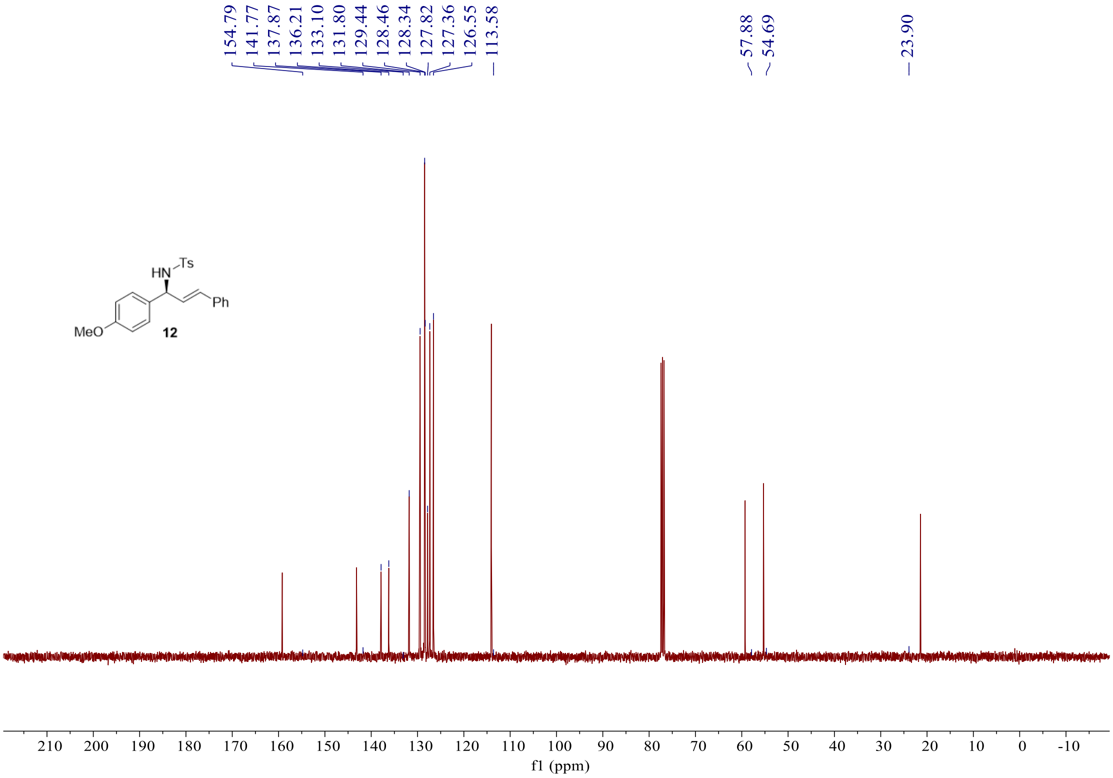

^{1}H NMR of Compound **13** ($CDCl_3$, 400 MHz, 20 °C):

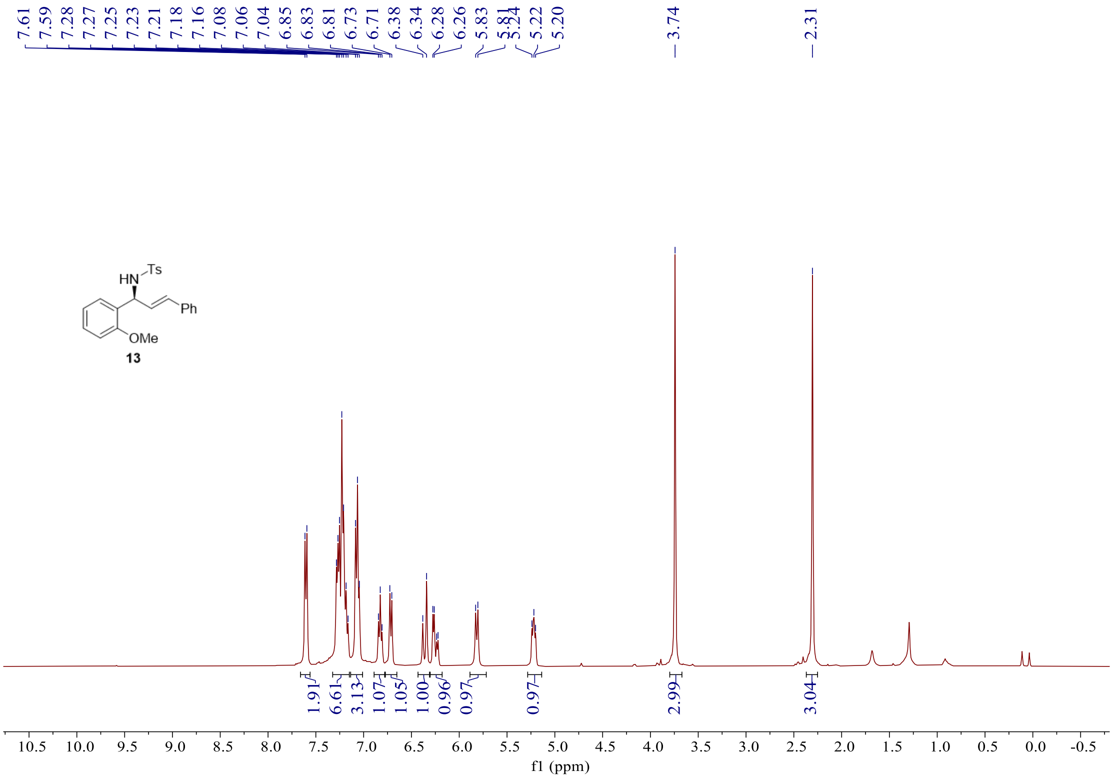

^{13}C NMR of Compound **13** ($CDCl_3$, 101MHz, 20 °C):

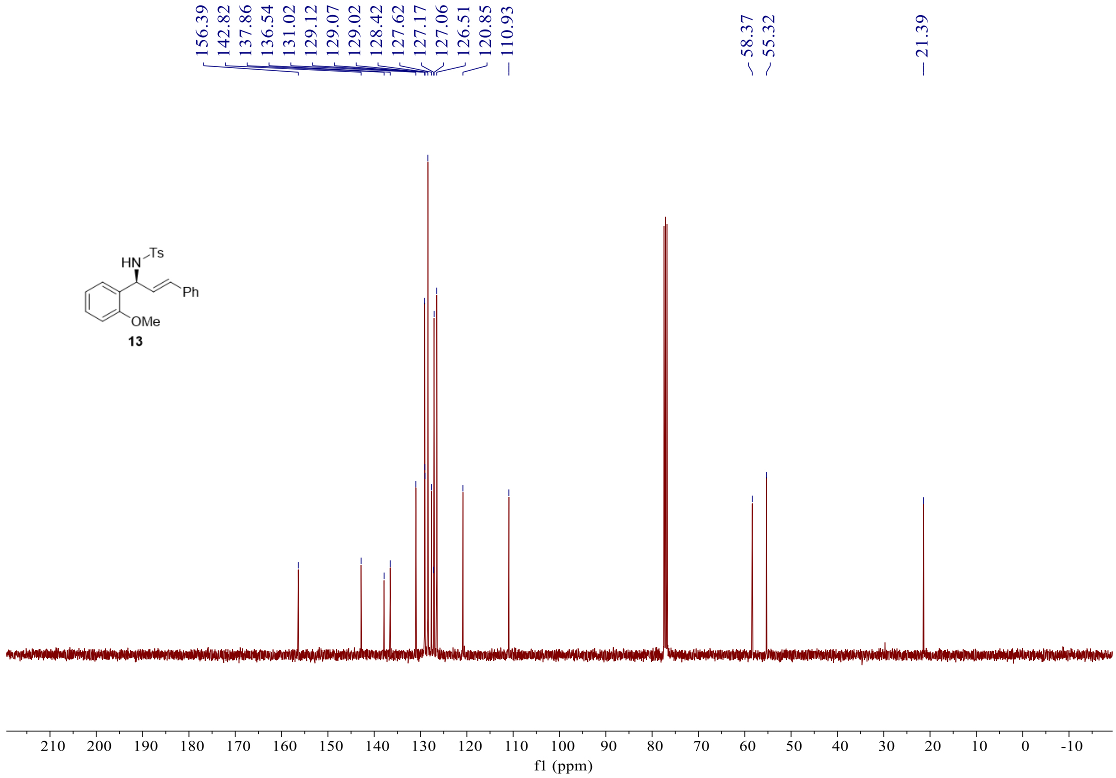

^{1}H NMR of Compound **14** ($CDCl_3$, 400 MHz, 20 °C):

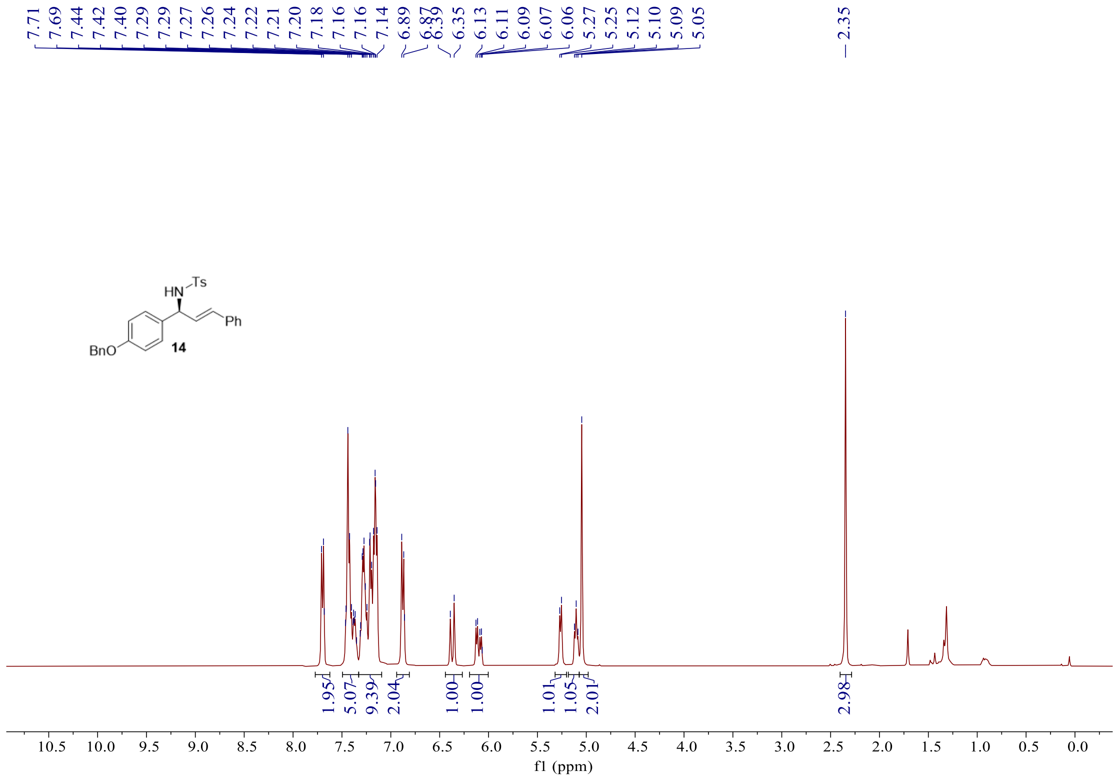

^{13}C NMR of Compound **14** ($CDCl_3$, 101MHz, 20 °C):

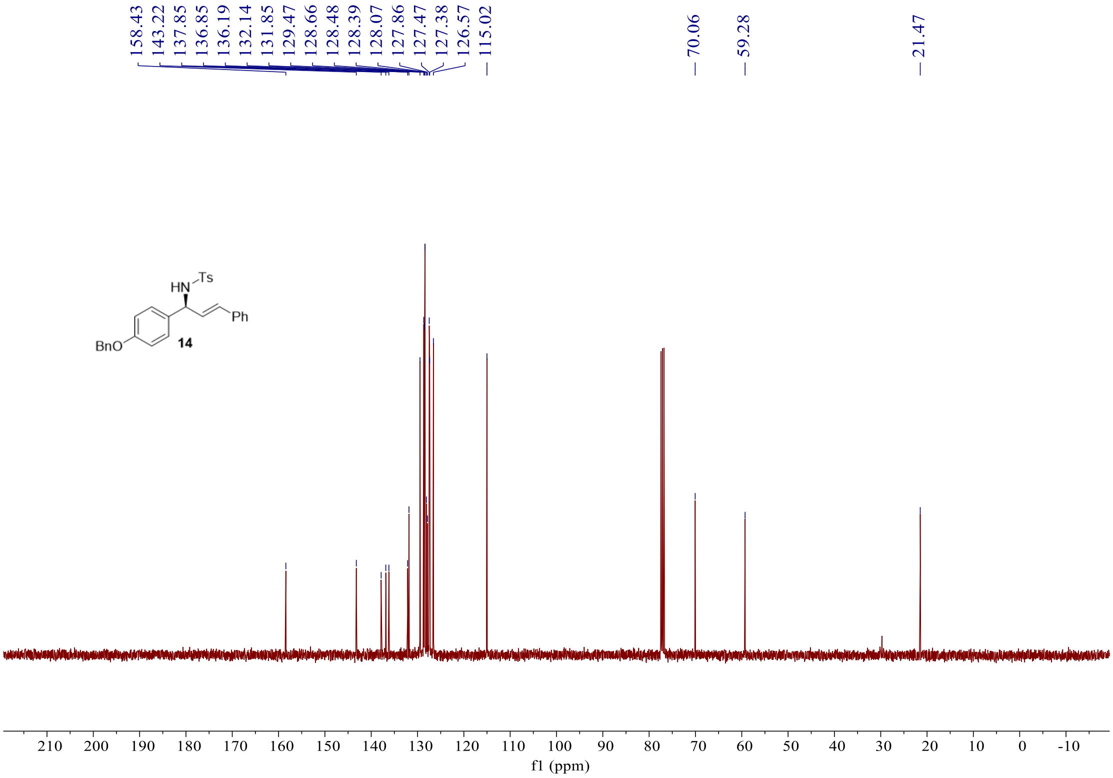

^{1}H NMR of Compound **15** ($CDCl_3$, 400 MHz, 20 °C):

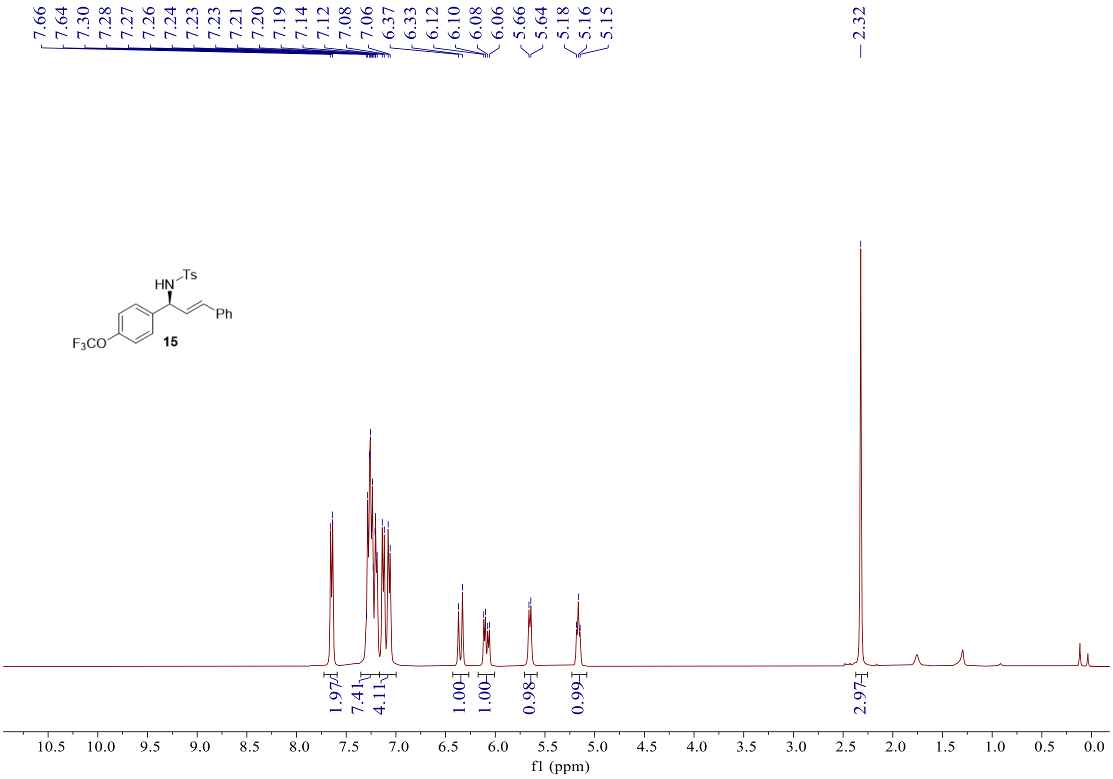

^{13}C NMR of Compound **15** ($CDCl_3$, 101MHz, 20 °C):

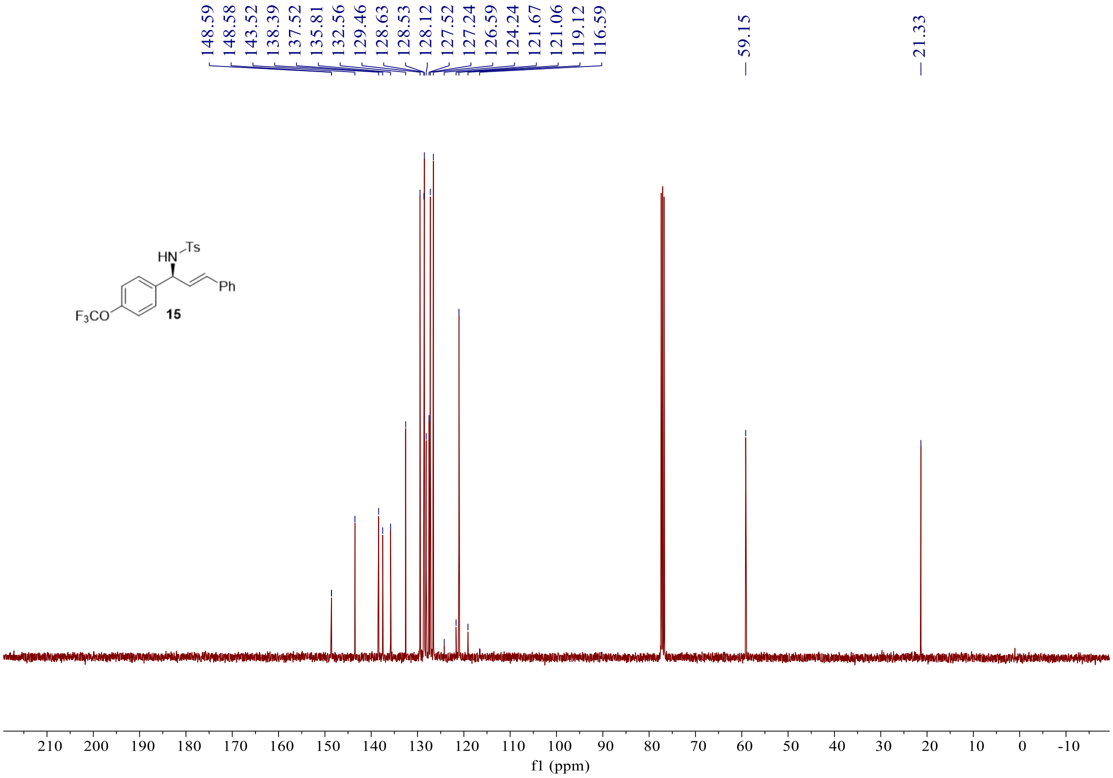

^{19}F NMR of Compound **15** ($CDCl_3$, 376MHz, 20 °C):

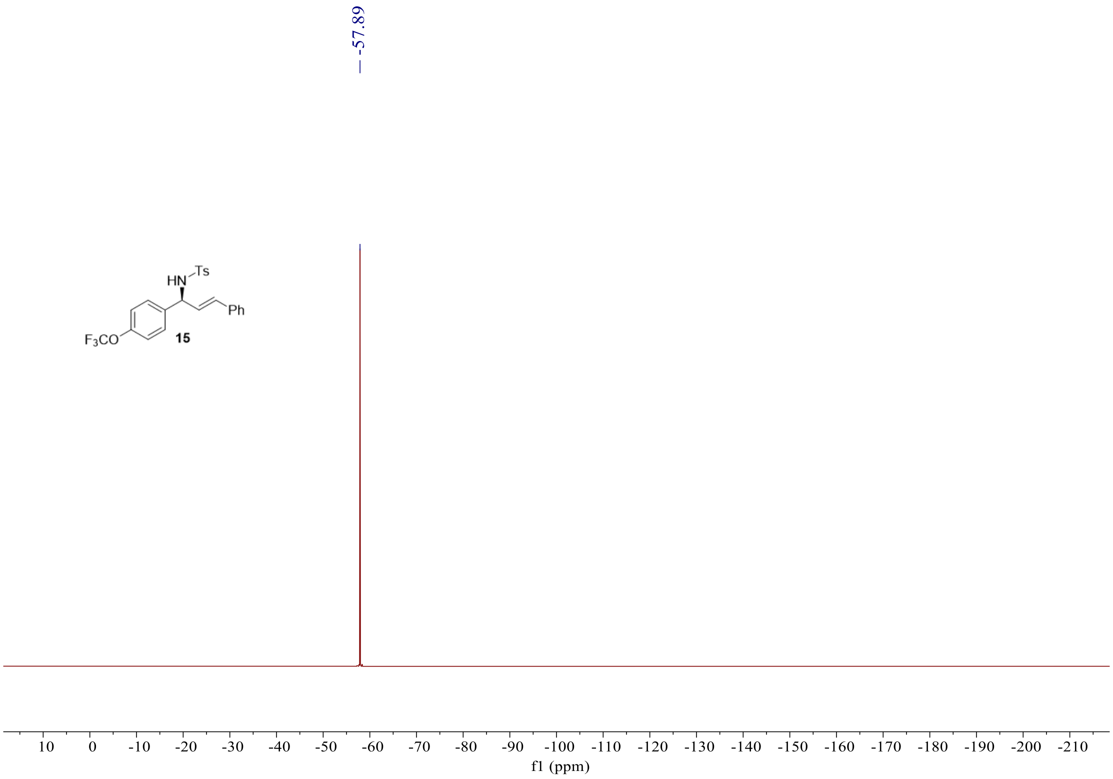

^{1}H NMR of Compound **16** ($CDCl_3$, 400 MHz, 20 °C):

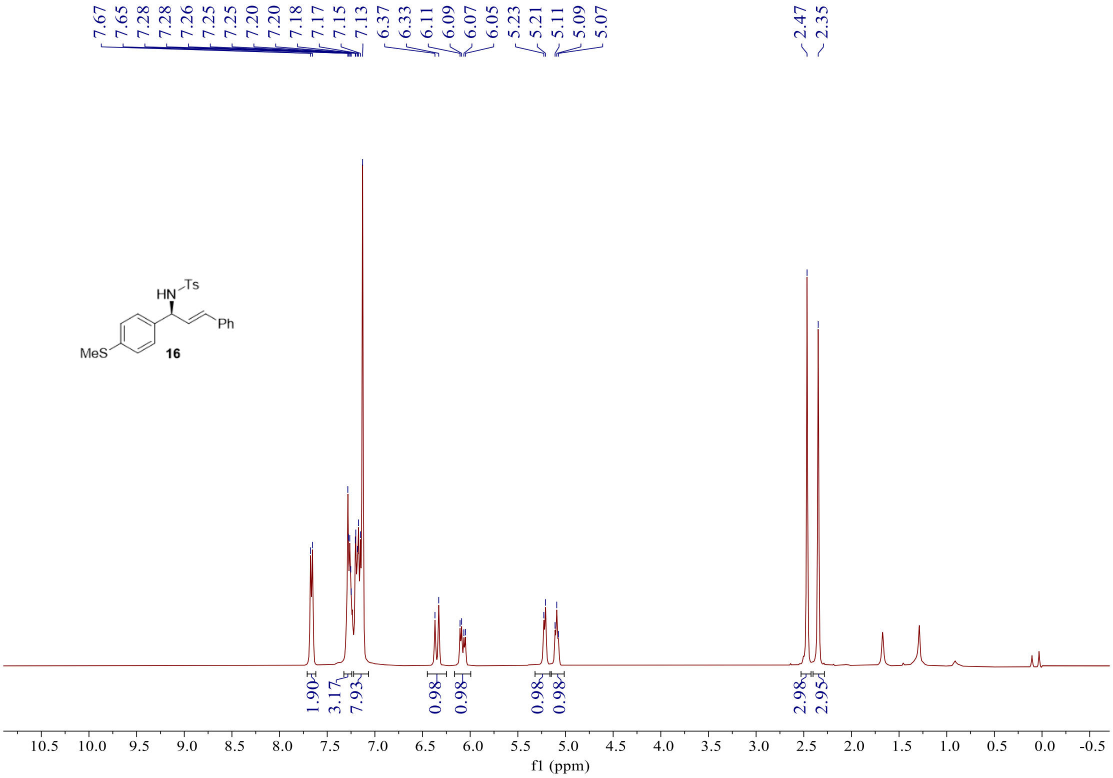

^{13}C NMR of Compound **16** ($CDCl_3$, 101MHz, 20 °C):

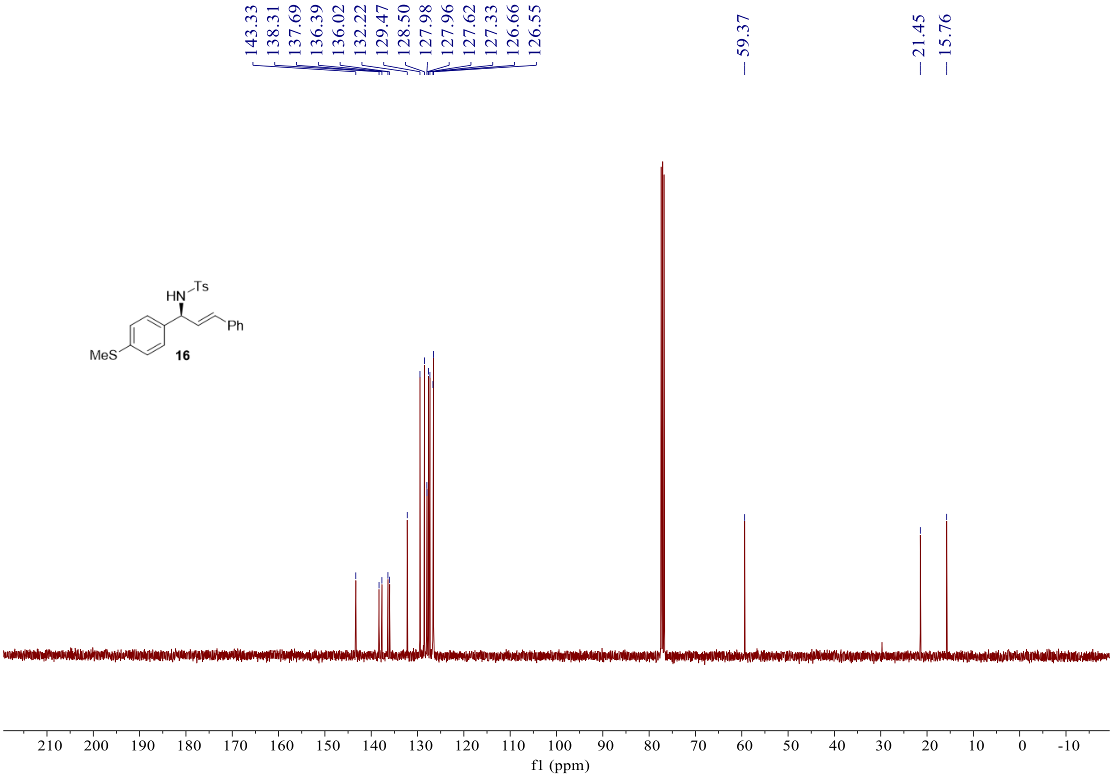

^{1}H NMR of Compound **17** ($CDCl_3$, 400 MHz, 20 °C):

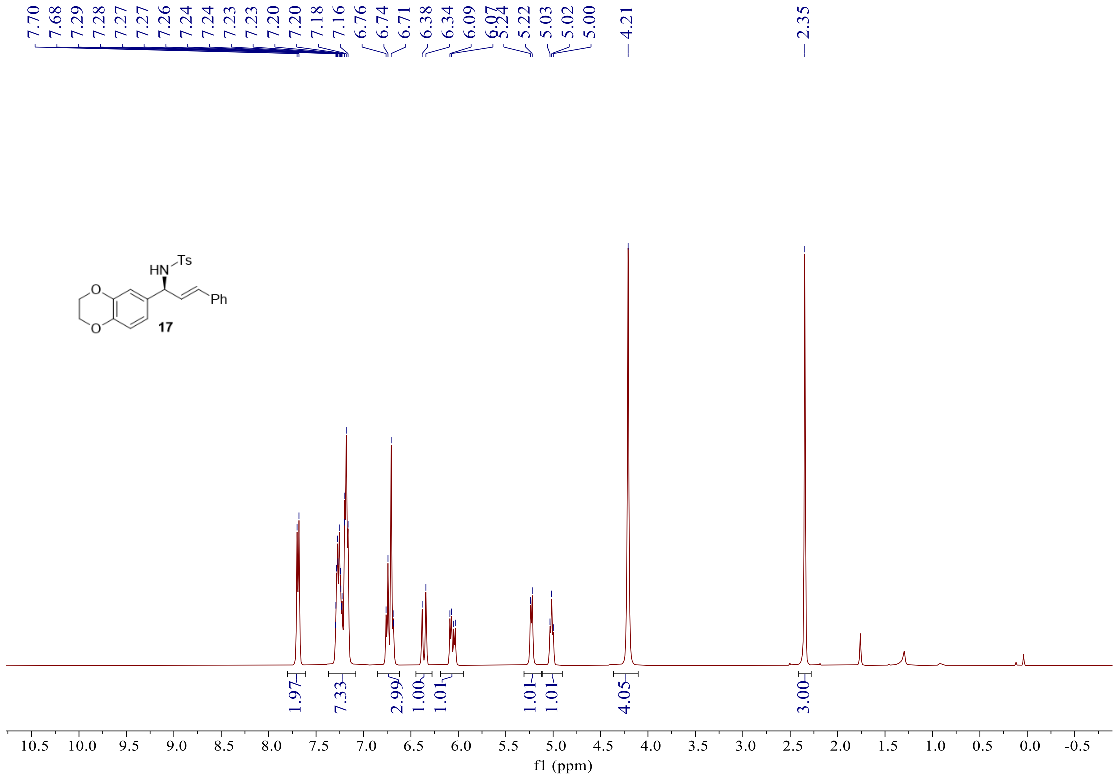

^{13}C NMR of Compound **17** ($CDCl_3$, 101MHz, 20 °C):

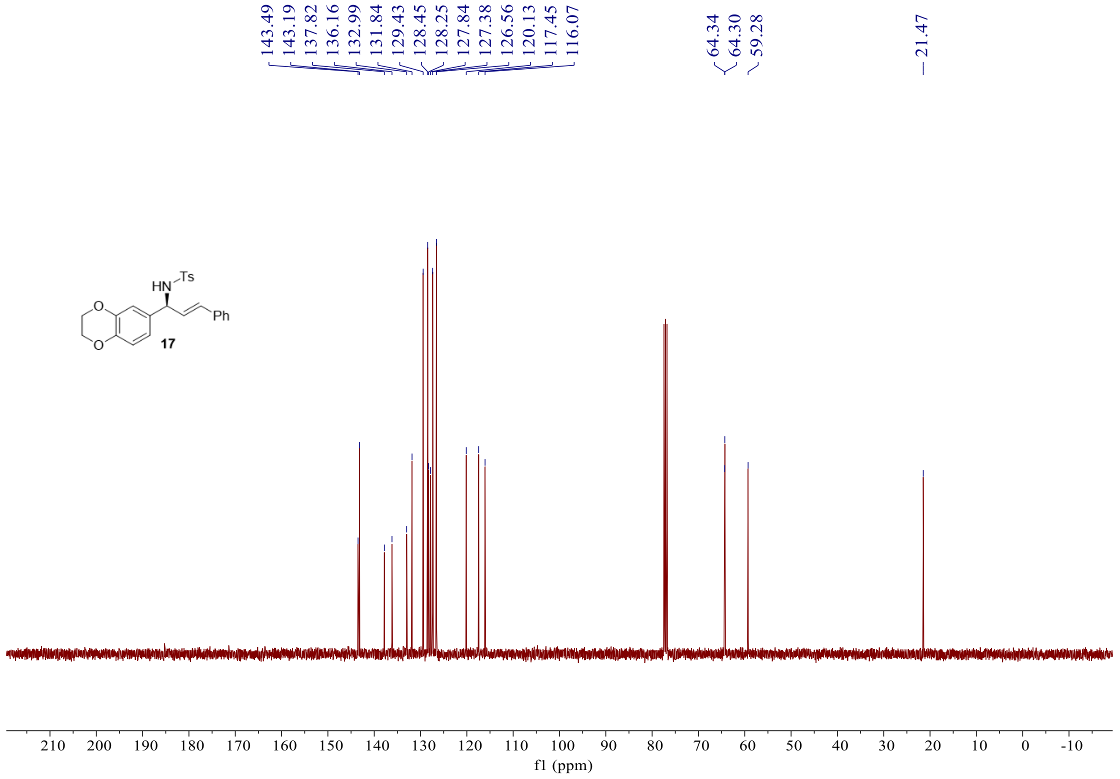

^{1}H NMR of Compound **18** (DMSO-*d6*, 400 MHz, 20 °C):

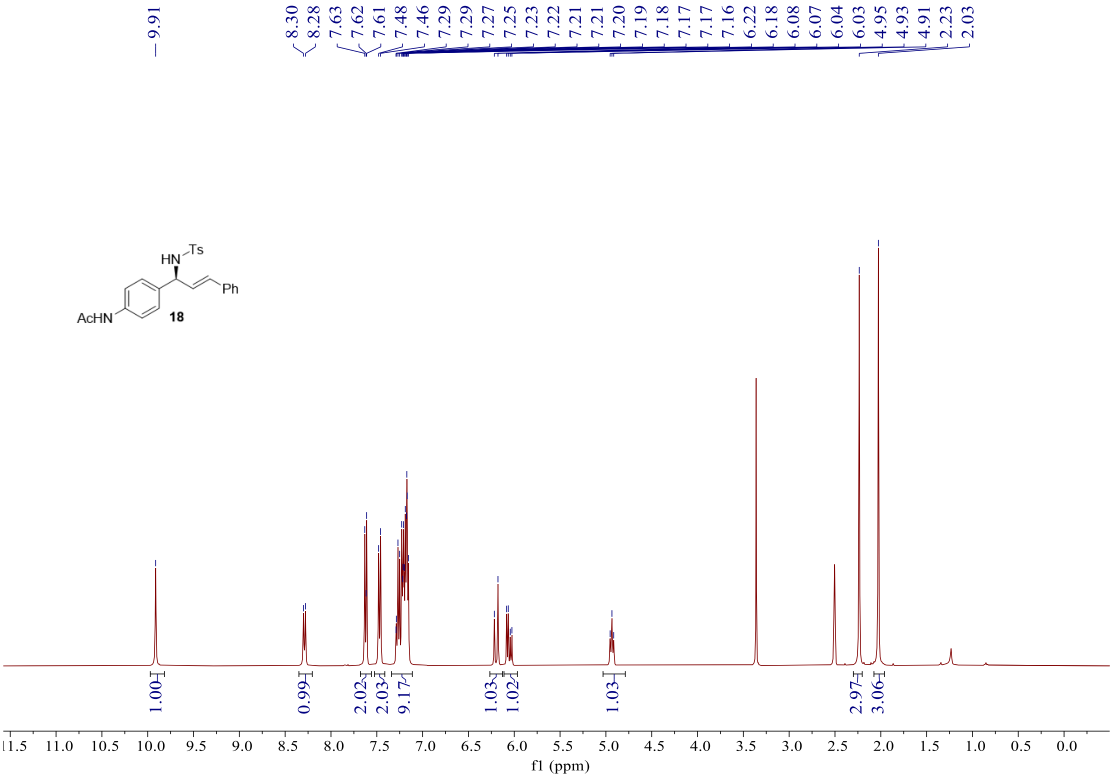

^{13}C NMR of Compound **18** (DMSO-*d6*, 101 MHz, 20 °C):

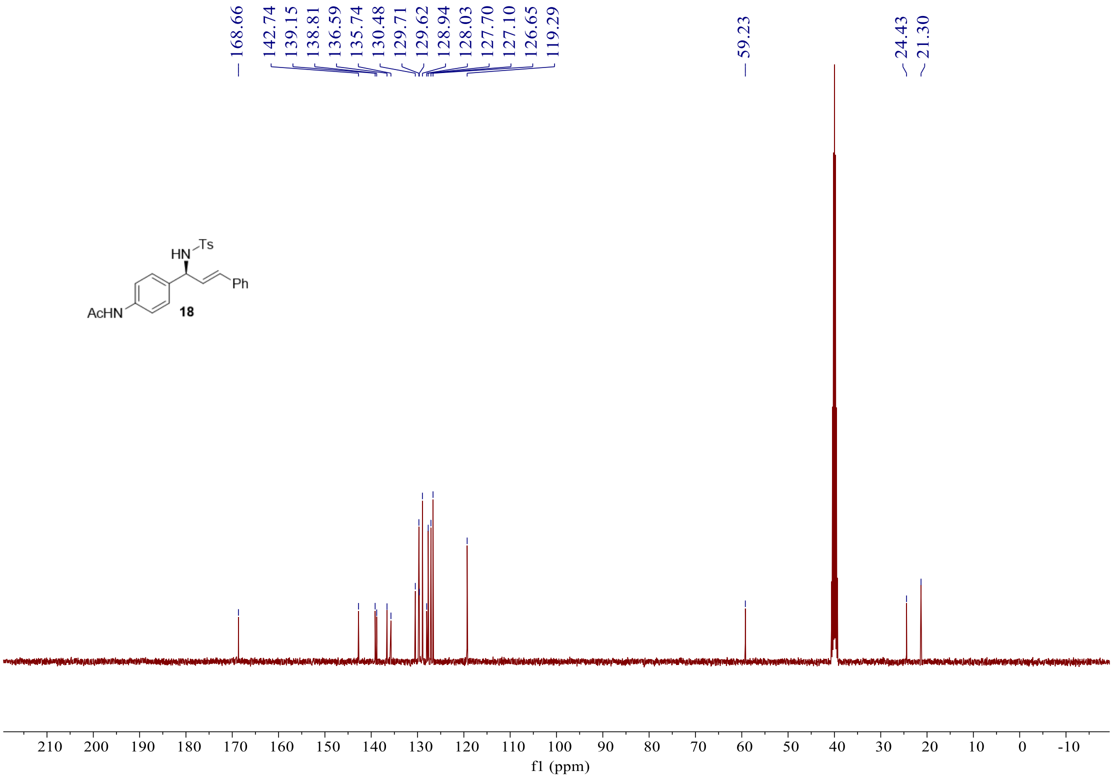

^{1}H NMR of Compound **19** ($CDCl_3$, 400 MHz, 20 °C):

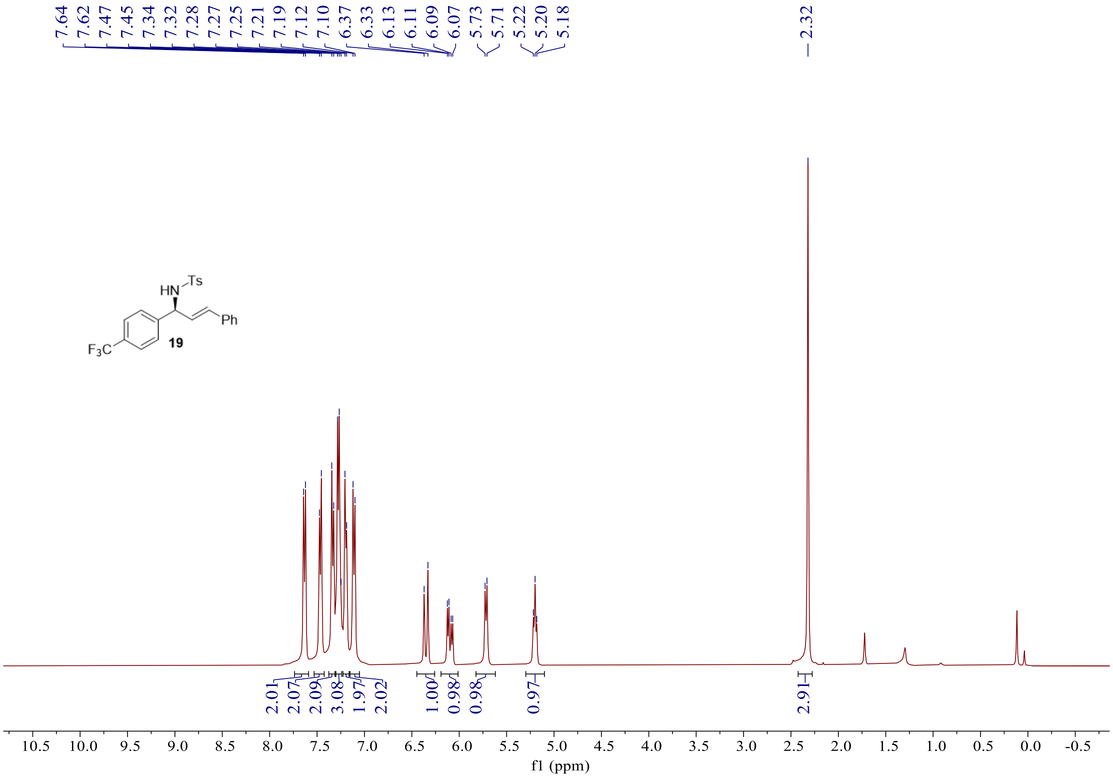

^{13}C NMR of Compound **19** ($CDCl_3$, 101MHz, 20 °C):

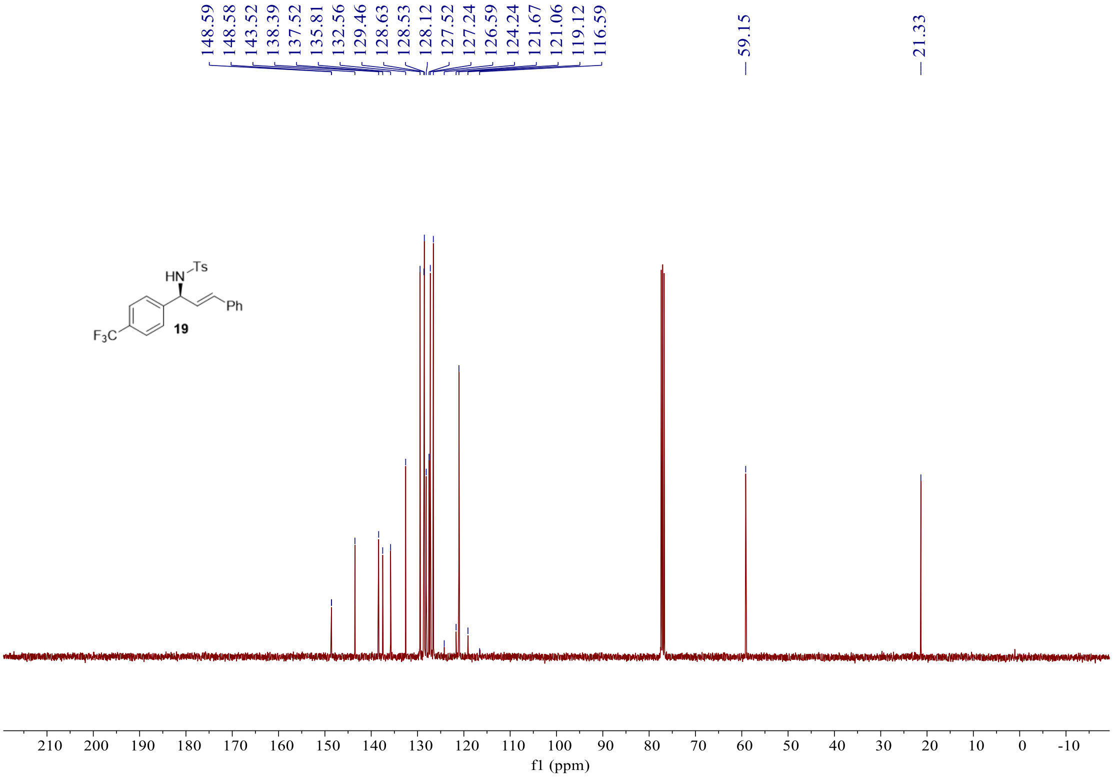

^{19}F NMR of Compound **19** ($CDCl_3$, 376MHz, 20 °C):

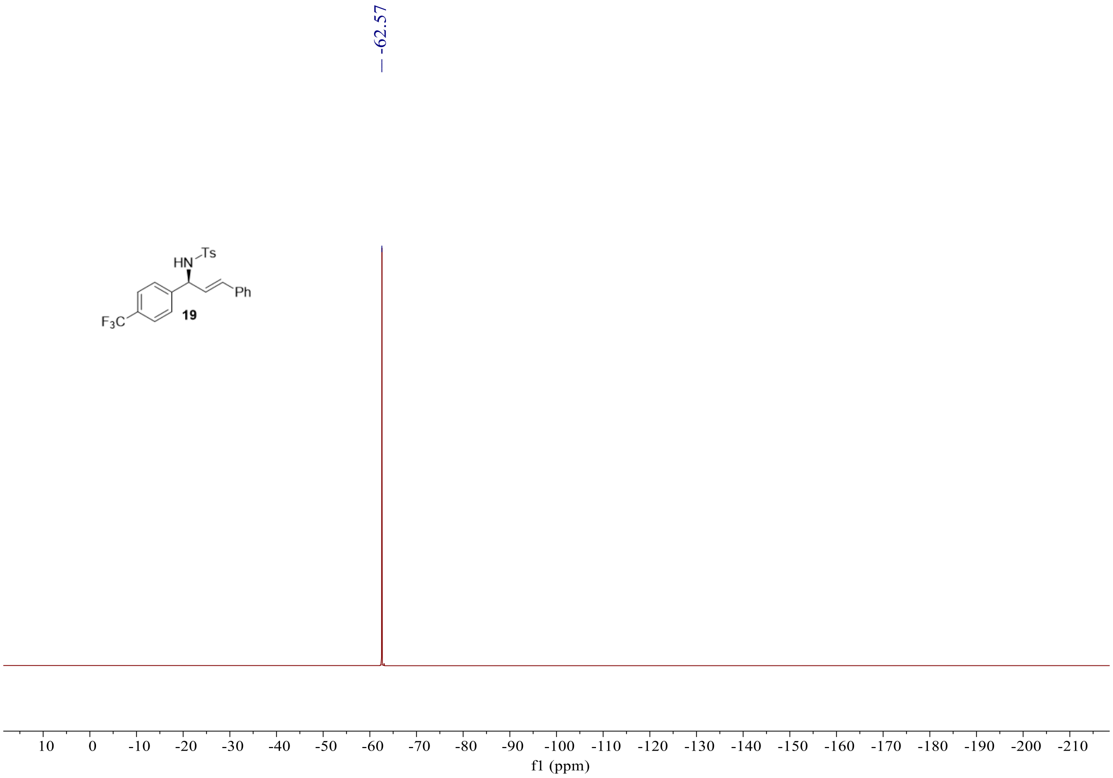

^{1}H NMR of Compound **20** ($CDCl_3$, 400 MHz, 20 °C):

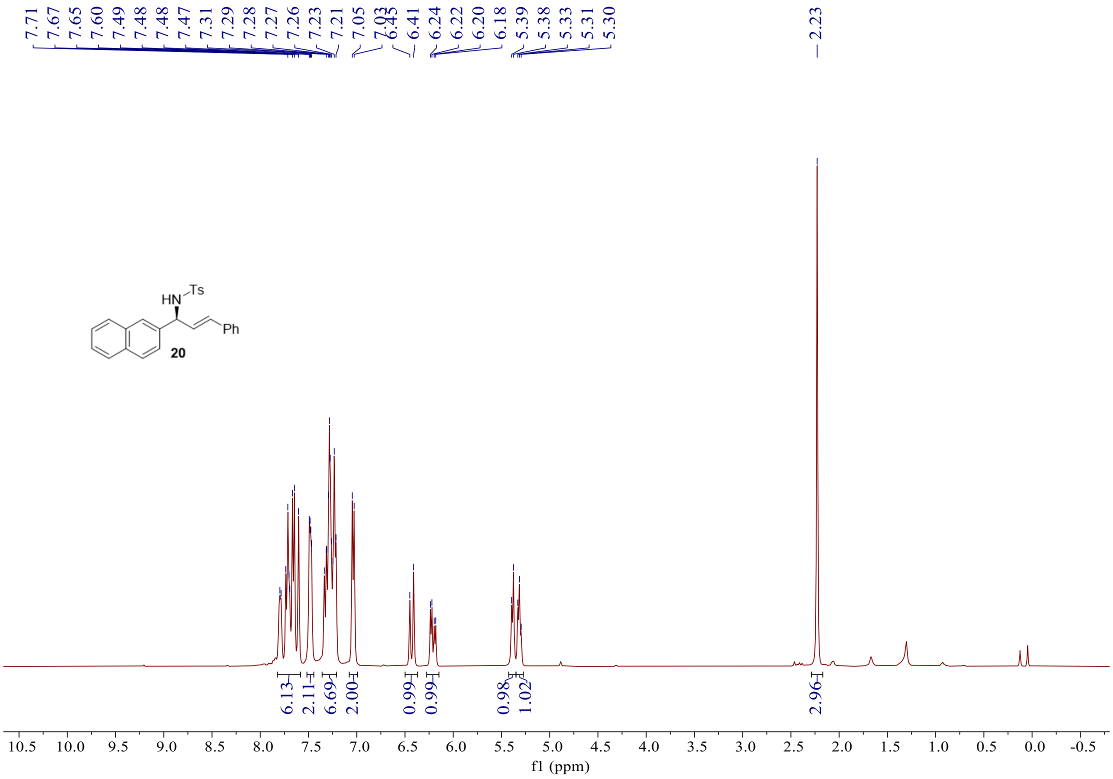

^{13}C NMR of Compound **20** ($CDCl_3$, 101MHz, 20 °C):

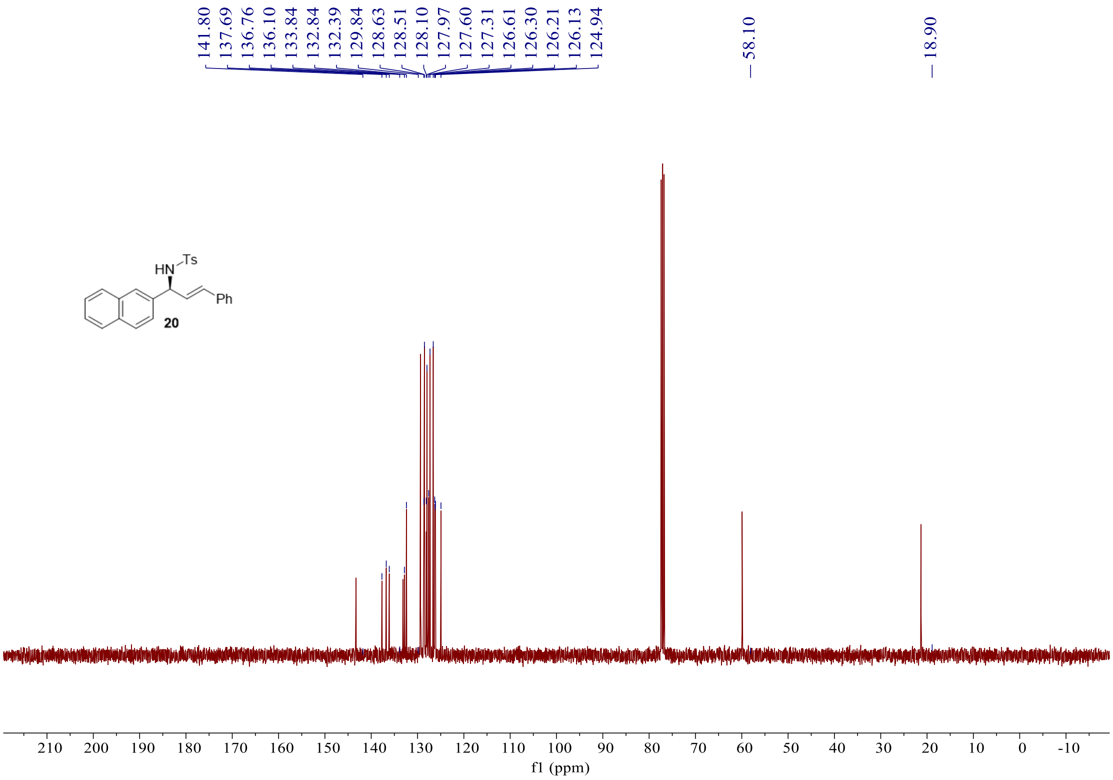

^{1}H NMR of Compound **21** ($CDCl_3$, 400 MHz, 20 °C):

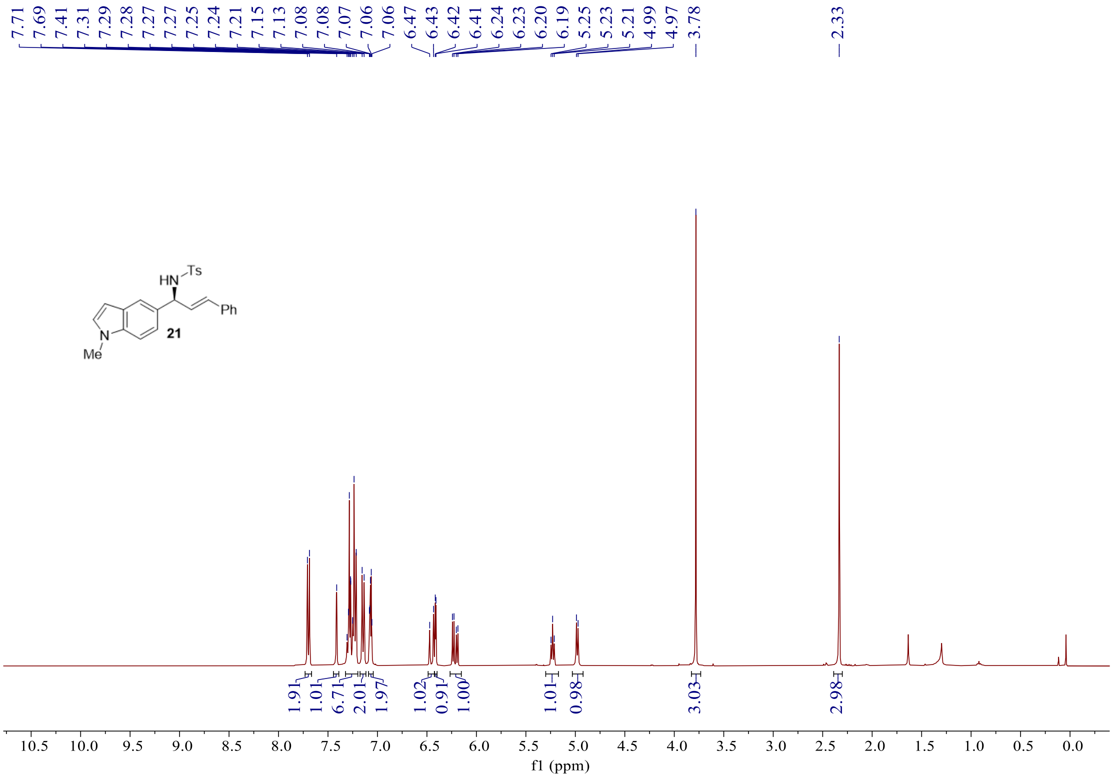

^{13}C NMR of Compound **21** ($CDCl_3$, 101MHz, 20 °C):

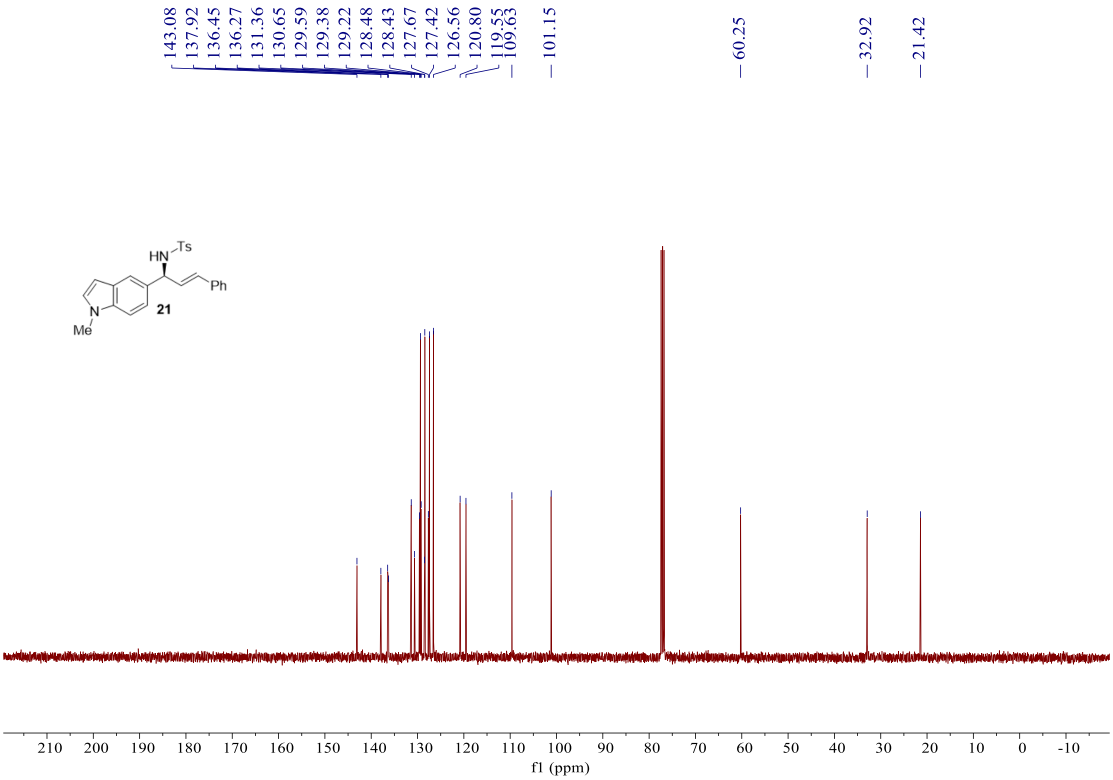

^{1}H NMR of Compound **22** ($CDCl_3$, 400 MHz, 20 °C):

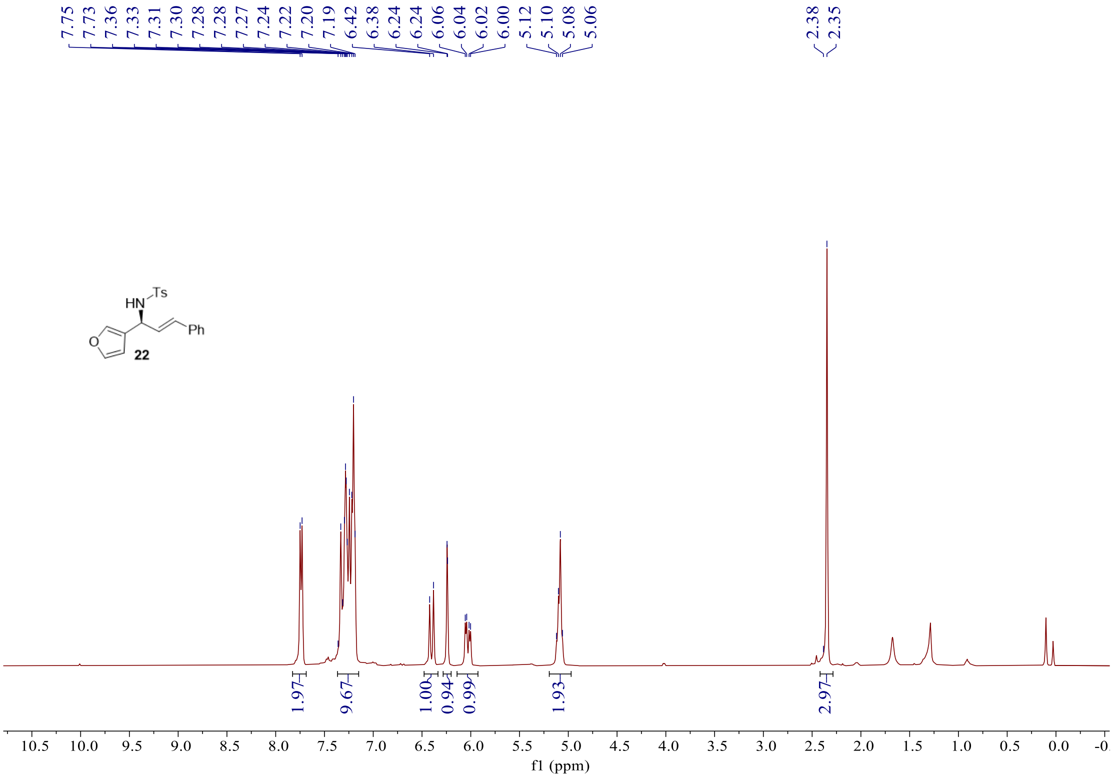

^{13}C NMR of Compound **22** ($CDCl_3$, 101MHz, 20 °C):

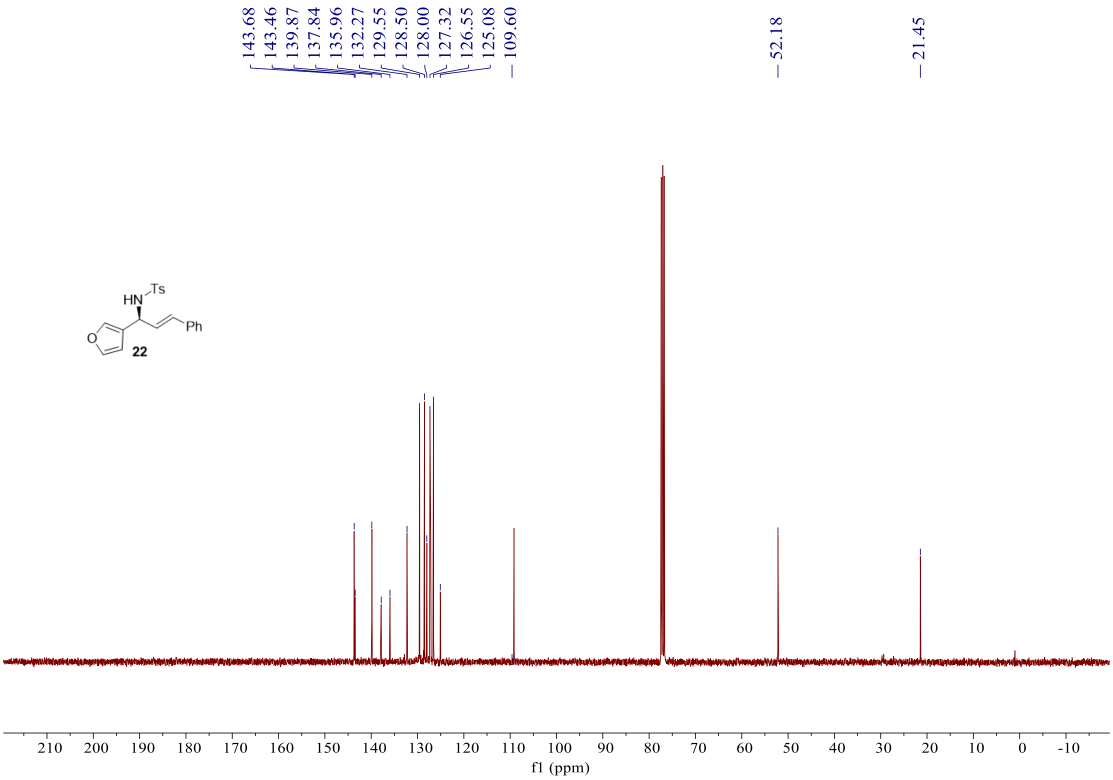

^{1}H NMR of Compound **23** ($CDCl_3$, 400 MHz, 20 °C):

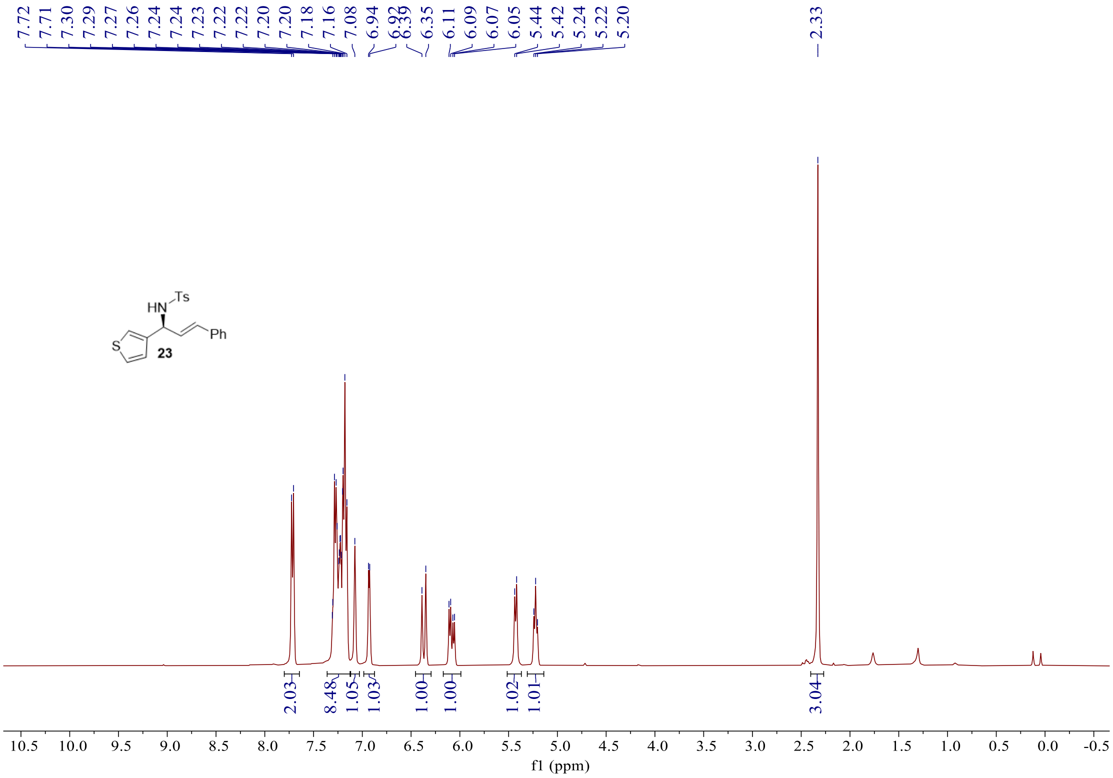

^{13}C NMR of Compound **23** ($CDCl_3$, 101MHz, 20 °C):

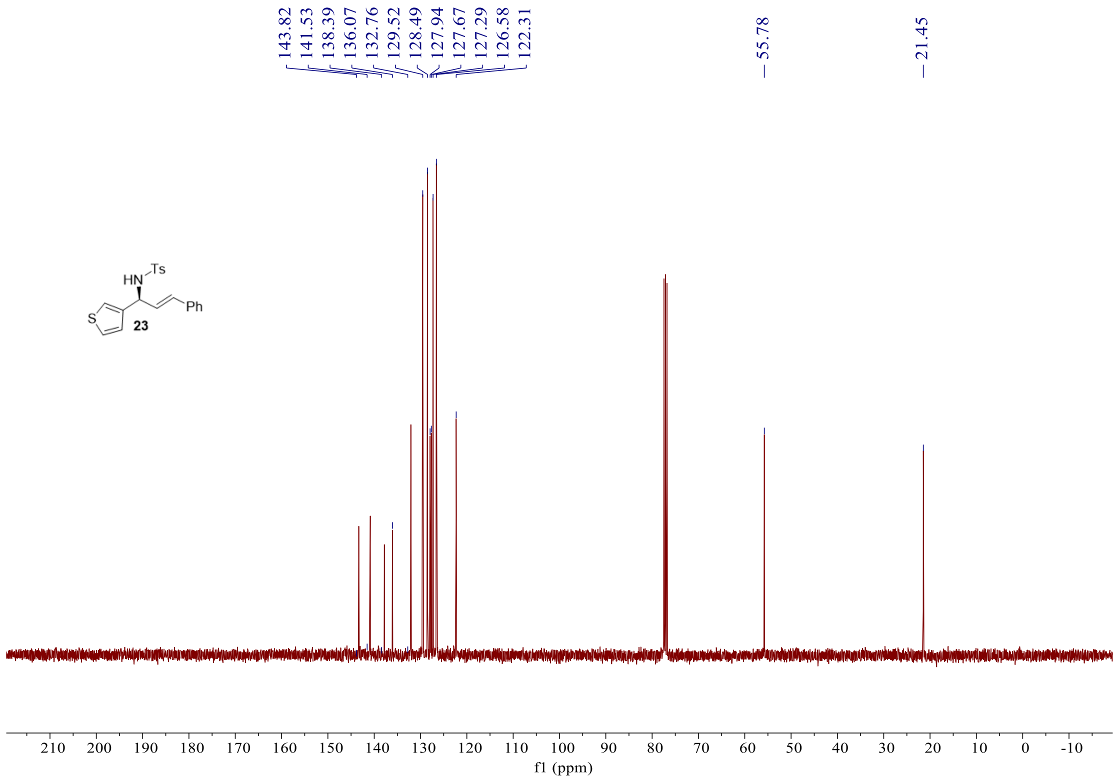

^{1}H NMR of Compound **24** ($CDCl_3$, 400 MHz, 20 °C):

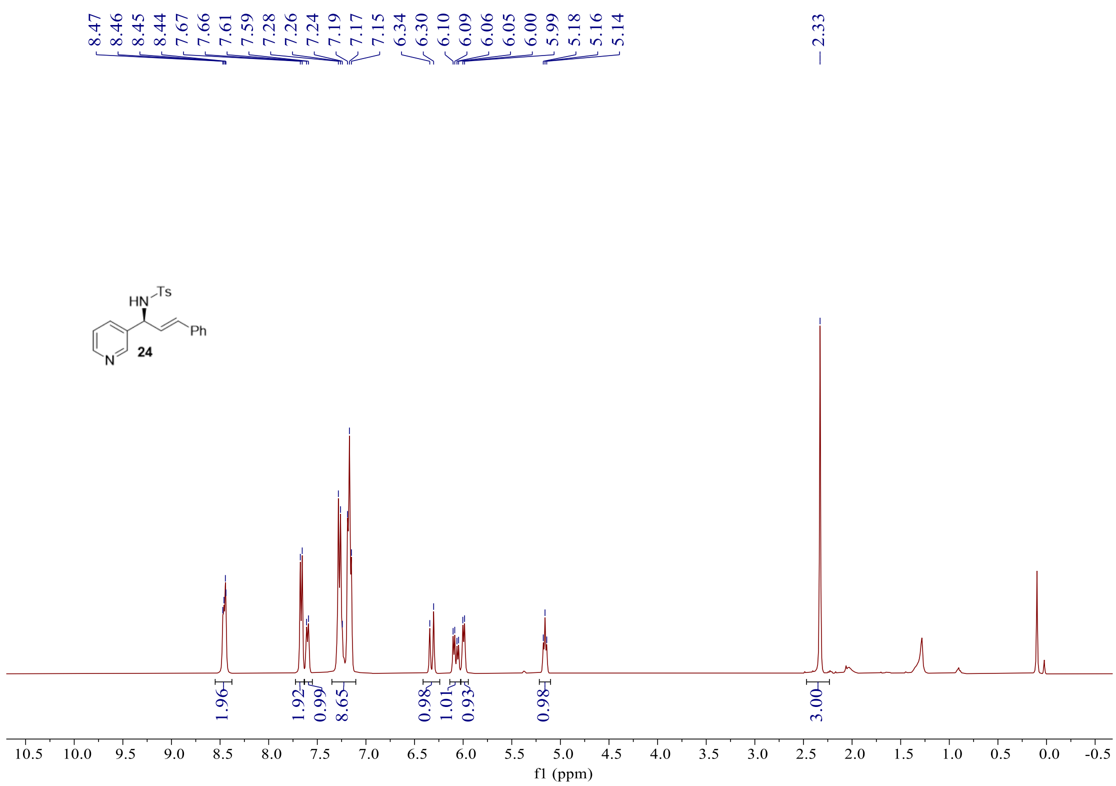

^{13}C NMR of Compound **24** ($CDCl_3$, 101MHz, 20 °C):

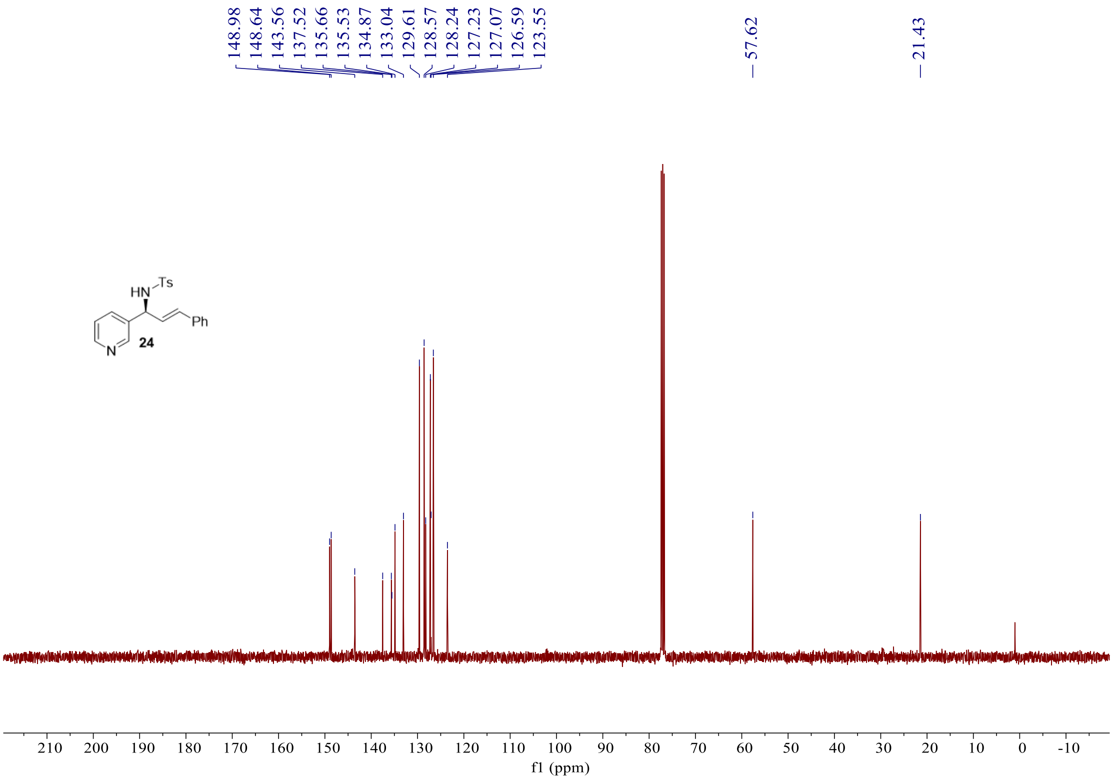

^{1}H NMR of Compound **25** ($CDCl_3$, 400 MHz, 20 °C):

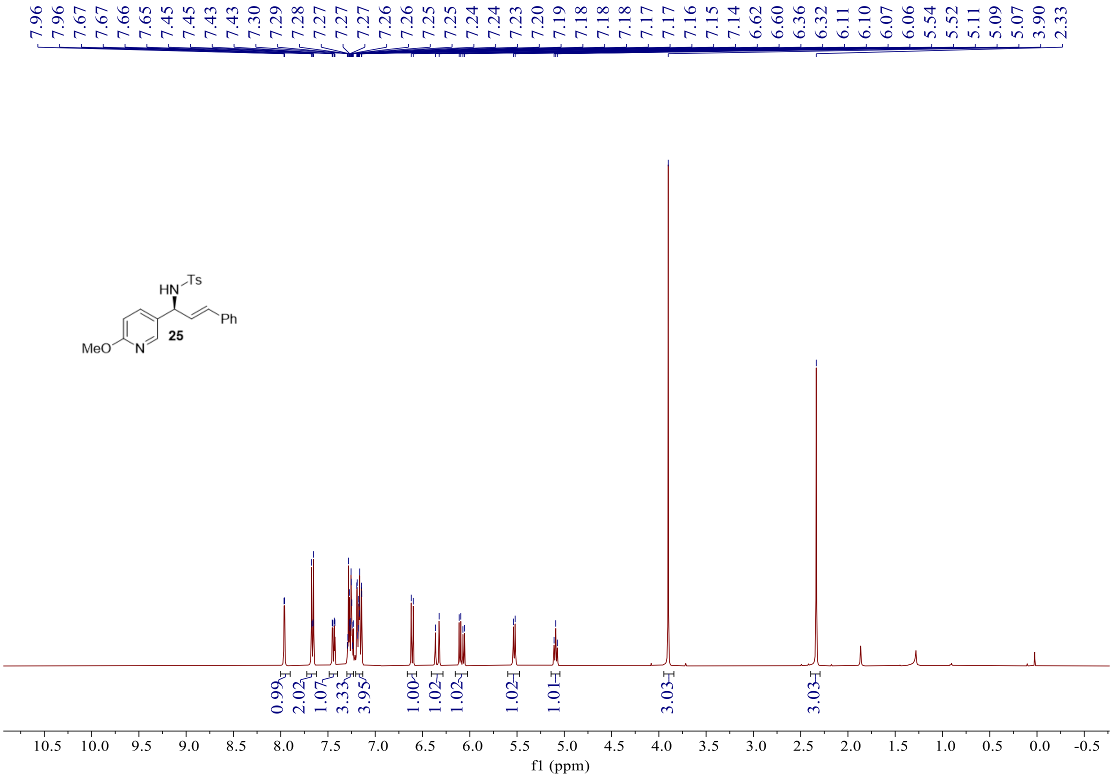

^{13}C NMR of Compound **25** ($CDCl_3$, 101MHz, 20 °C):

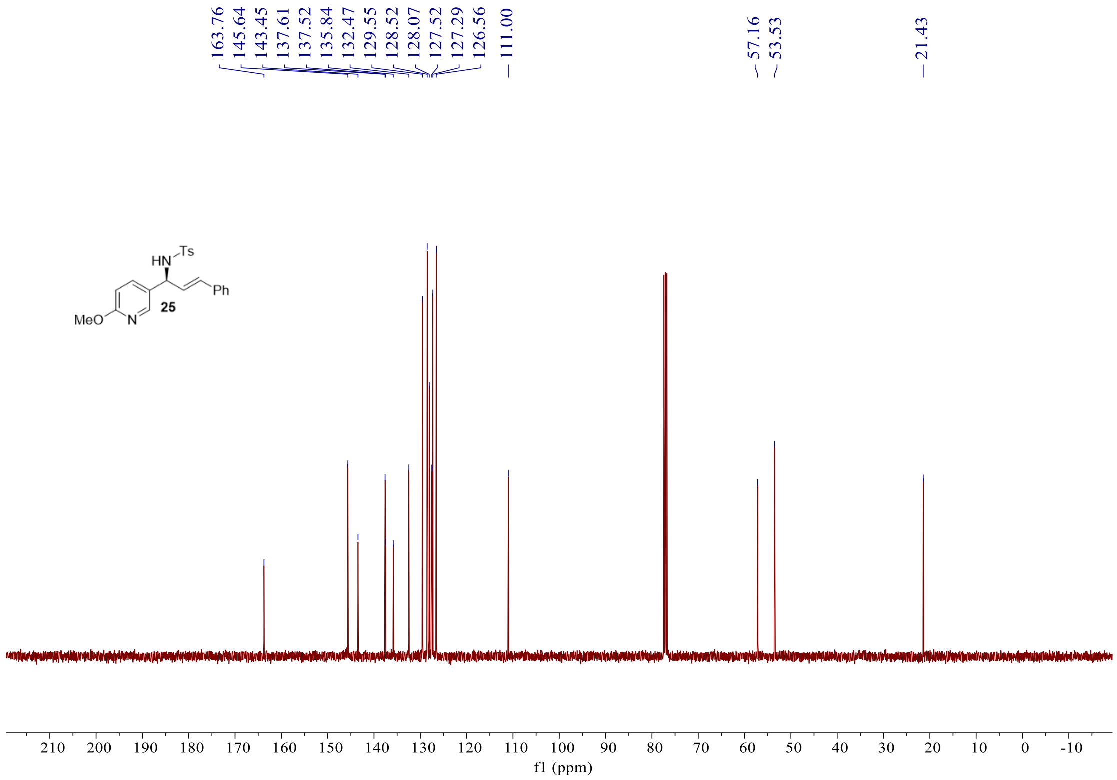

^{1}H NMR of Compound **26** ($CDCl_3$, 400 MHz, 20 °C):

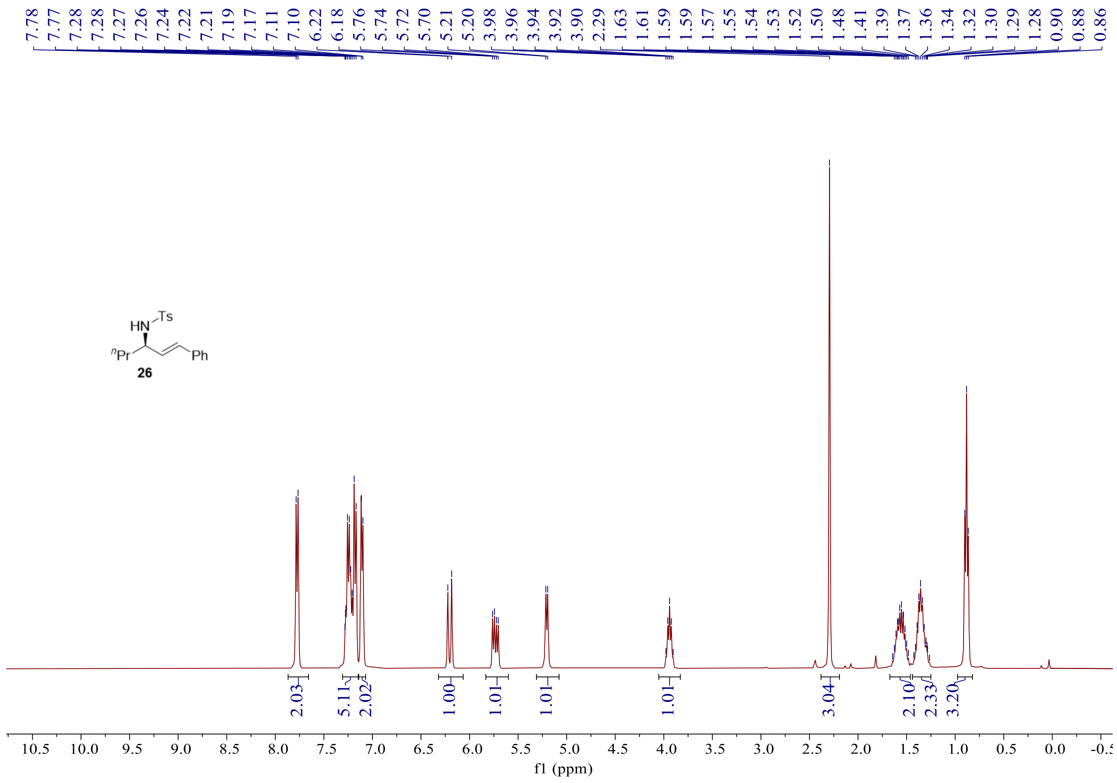

^{13}C NMR of Compound **26** ($CDCl_3$, 101MHz, 20 °C):

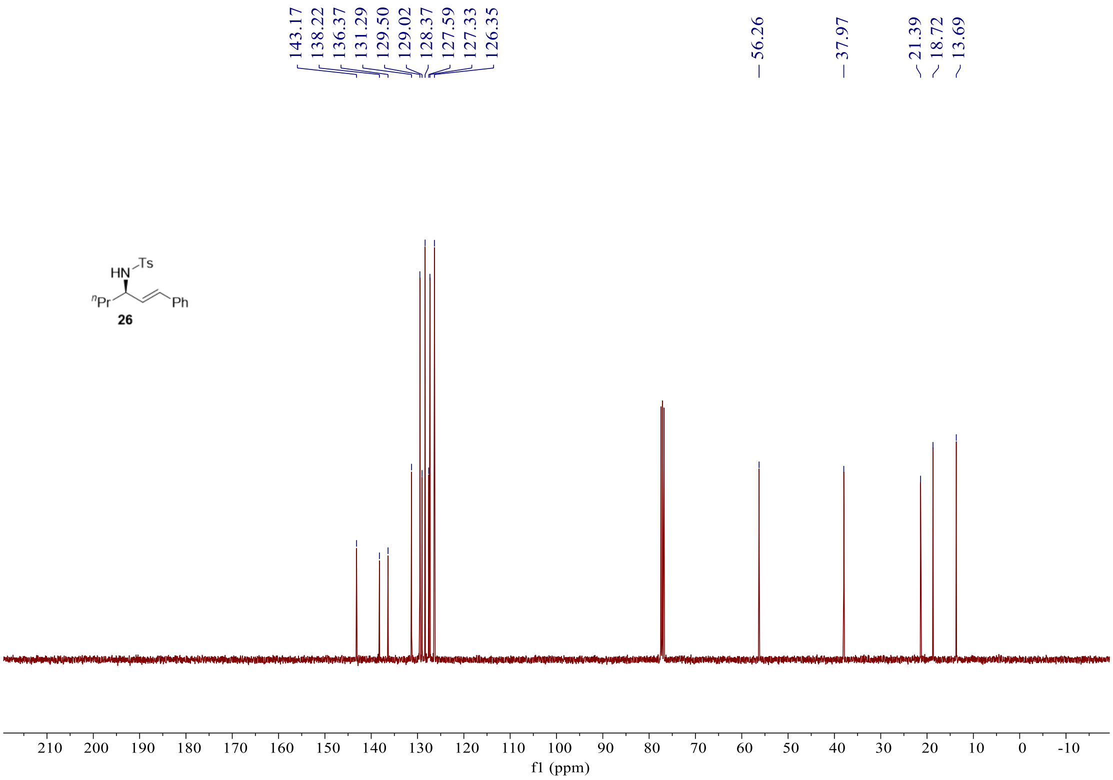

^{1}H NMR of Compound **27** ($CDCl_3$, 400 MHz, 20 °C):

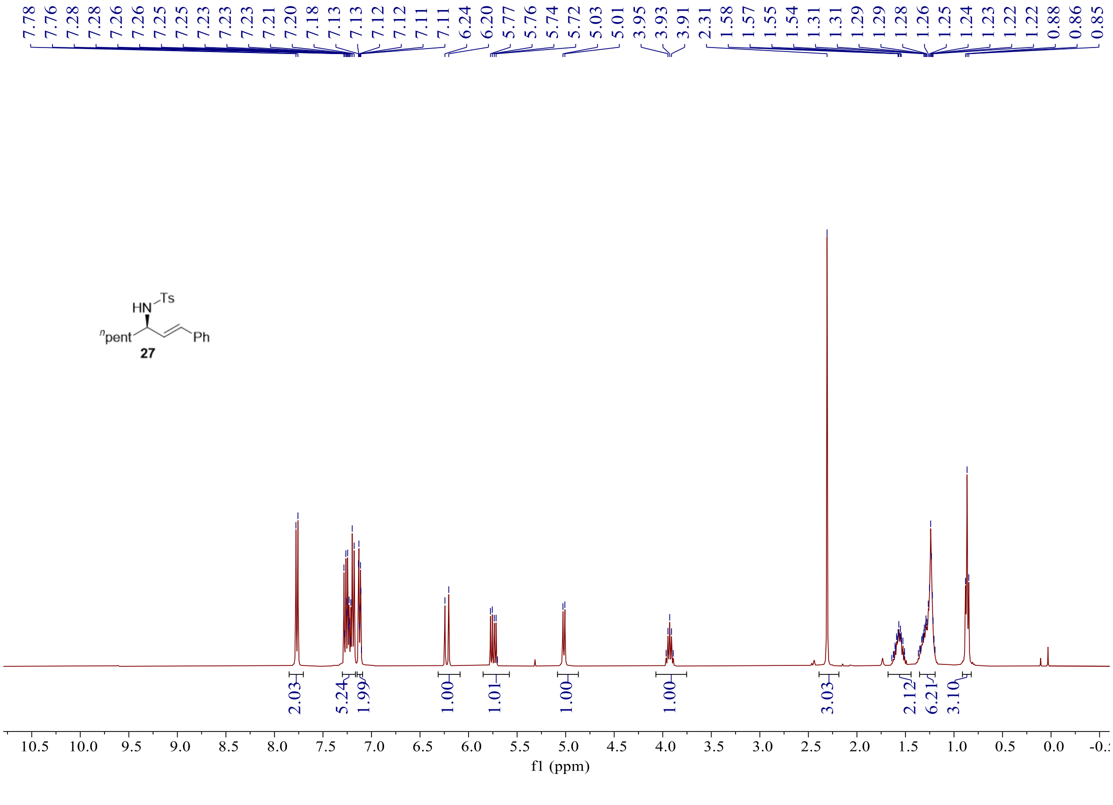

^{13}C NMR of Compound **27** ($CDCl_3$, 101MHz, 20 °C):

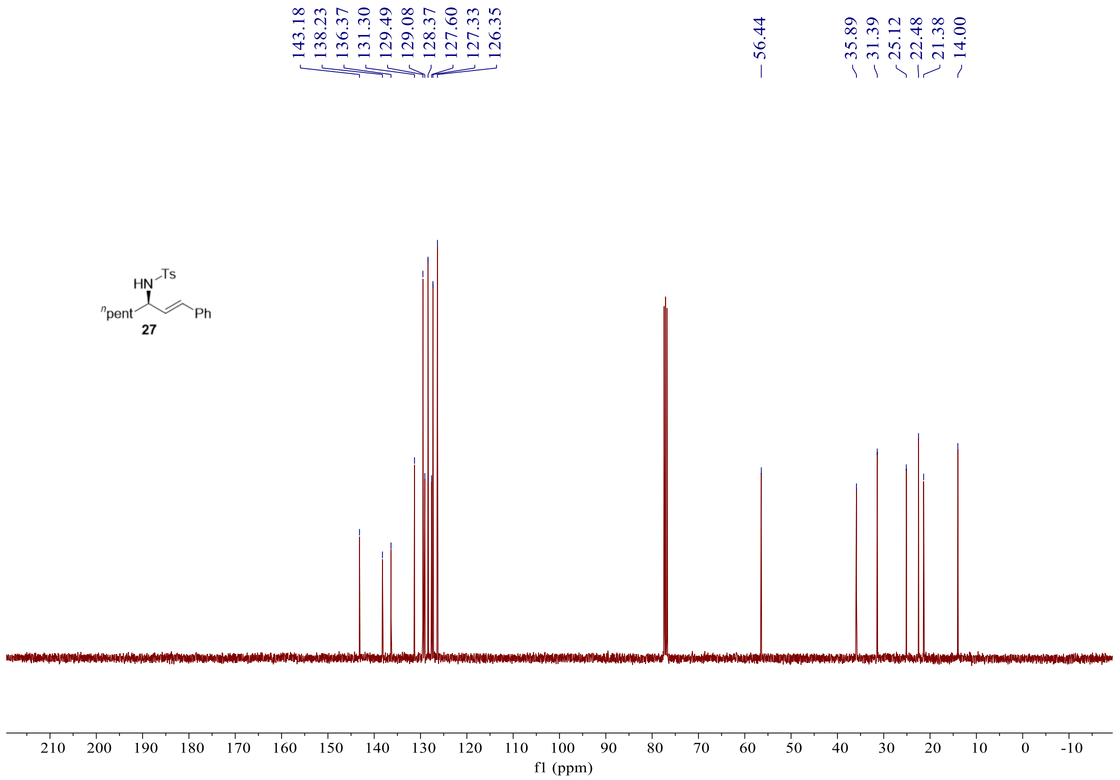

^{1}H NMR of Compound **28** ($CDCl_3$, 400 MHz, 20 °C):

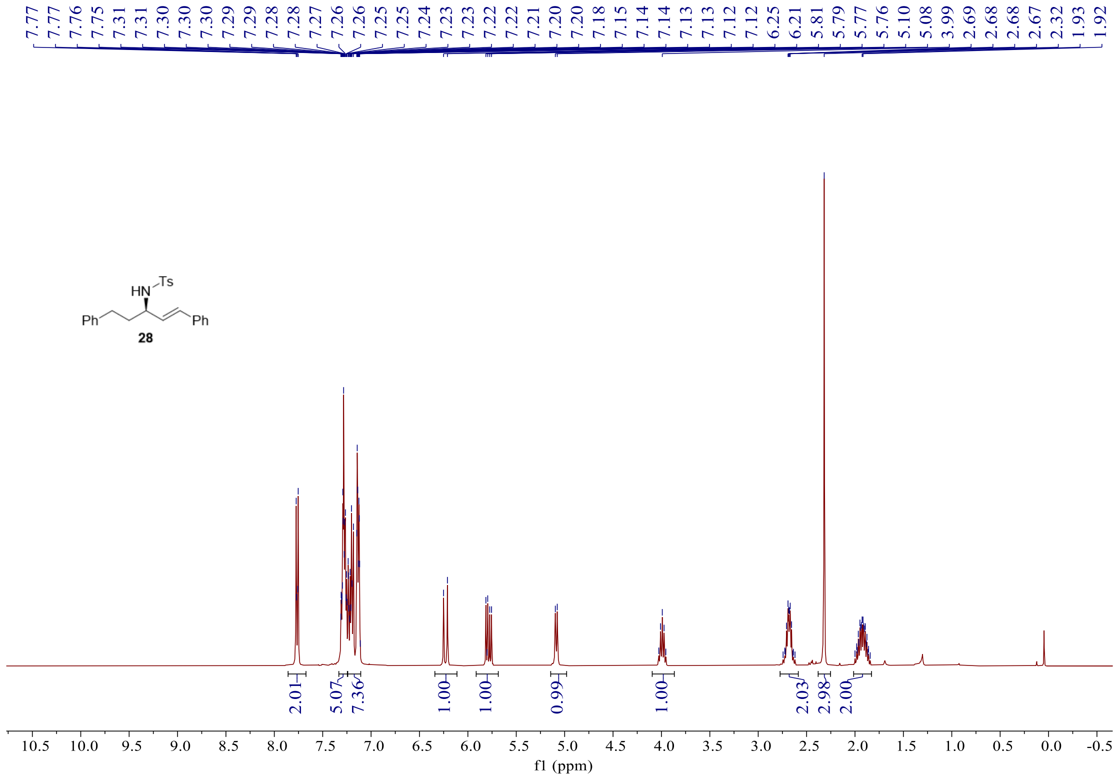

^{13}C NMR of Compound **28** ($CDCl_3$, 101MHz, 20 °C):

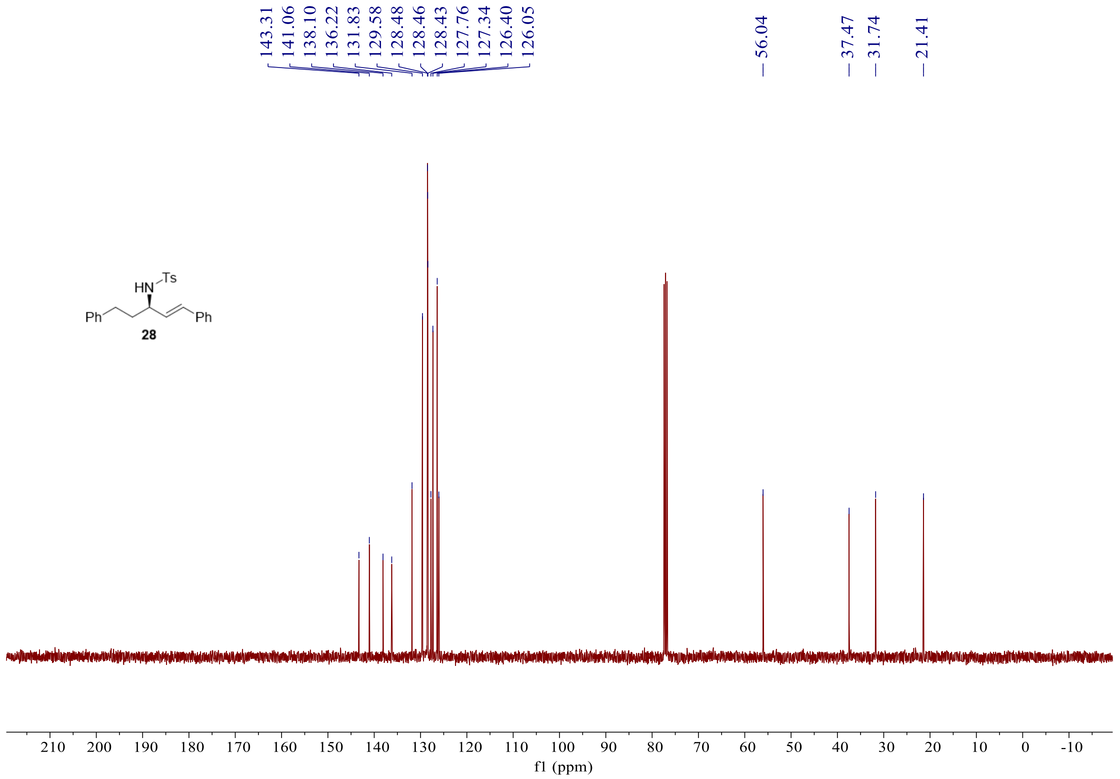

^{1}H NMR of Compound **29** ($CDCl_3$, 400 MHz, 20 °C):

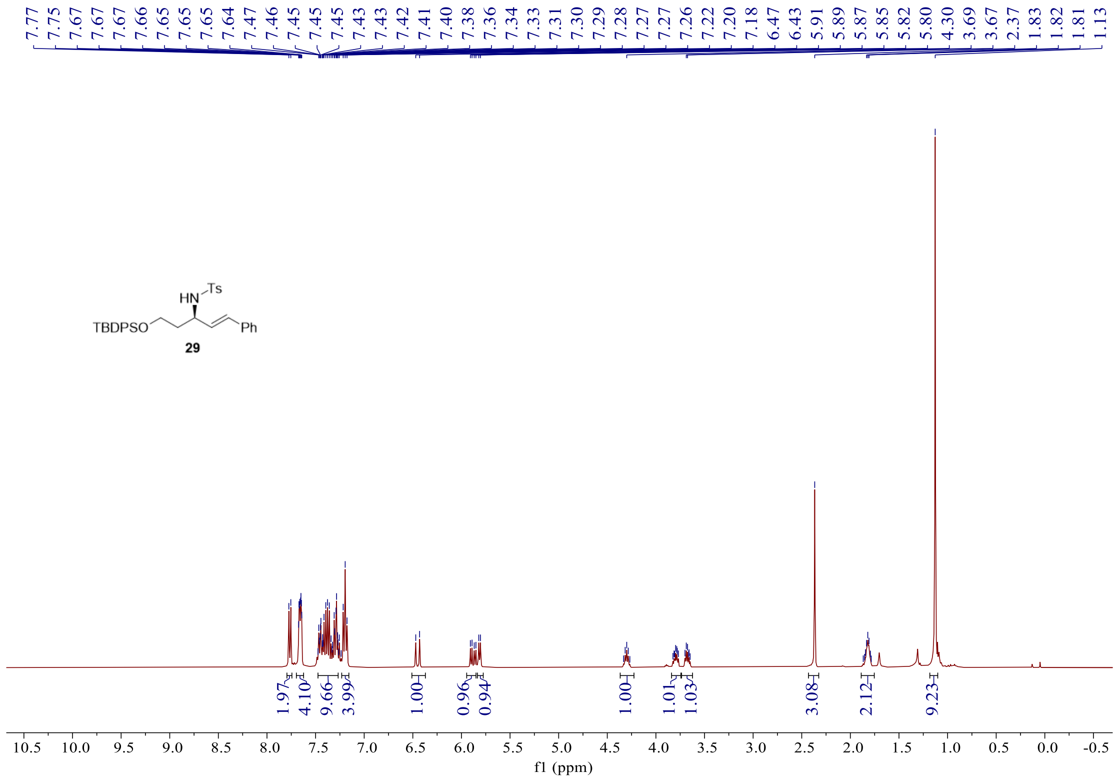

^{13}C NMR of Compound **29** ($CDCl_3$, 101MHz, 20 °C):

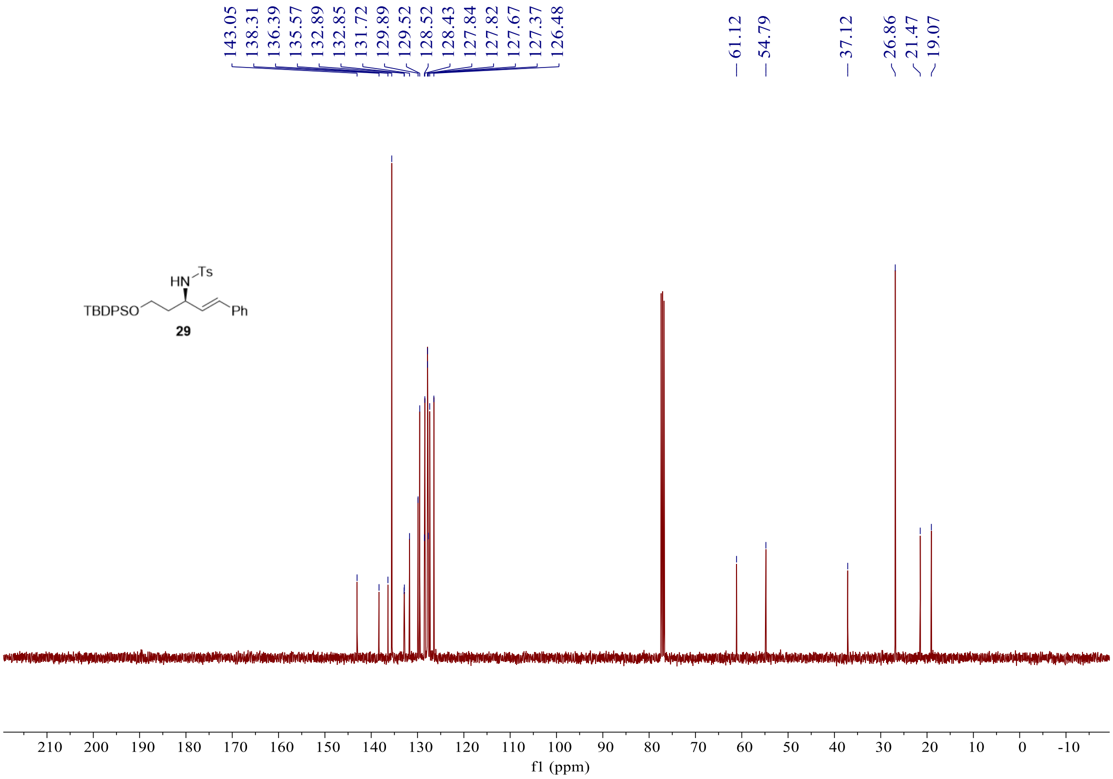

^{1}H NMR of Compound **30** ($CDCl_3$, 400 MHz, 20 °C):

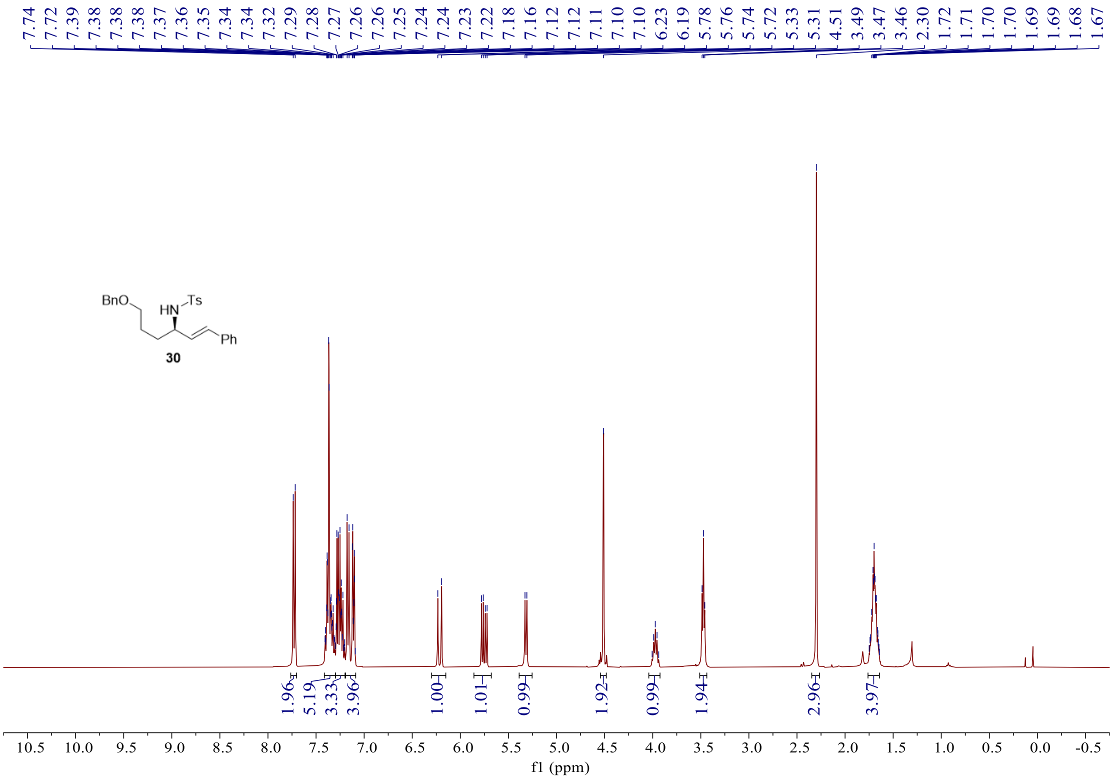

^{13}C NMR of Compound **30** ($CDCl_3$, 101MHz, 20 °C):

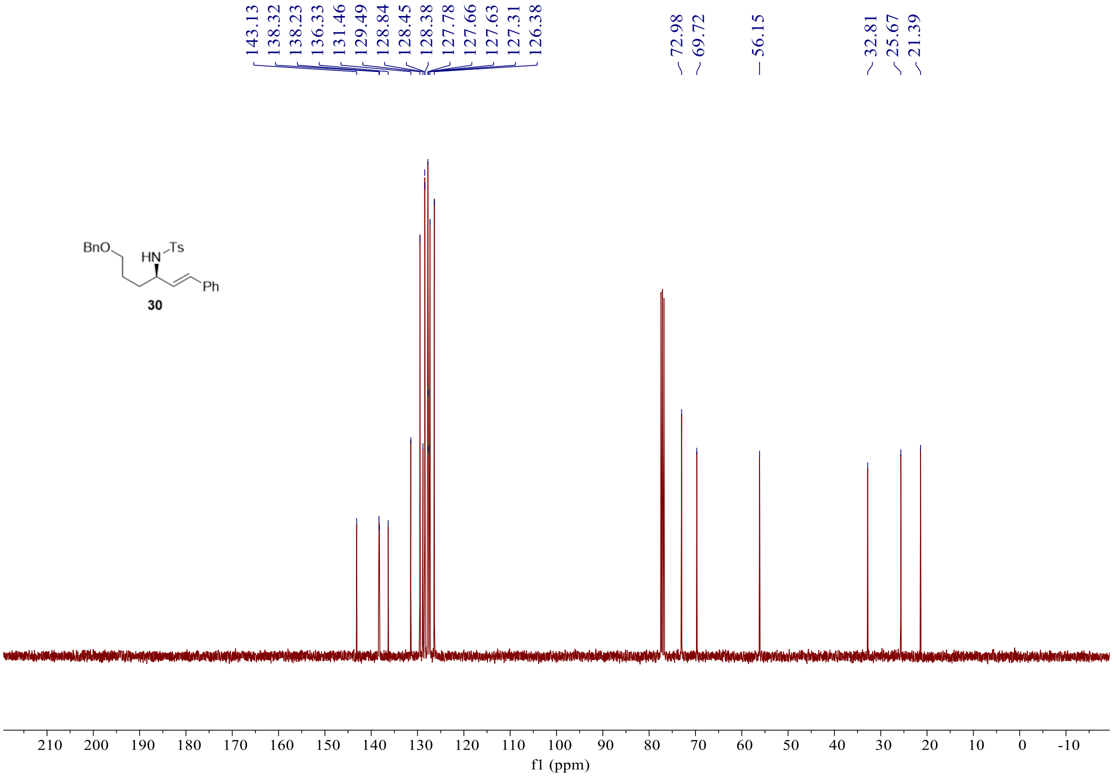

^{1}H NMR of Compound **31** ($CDCl_3$, 400 MHz, 20 °C):

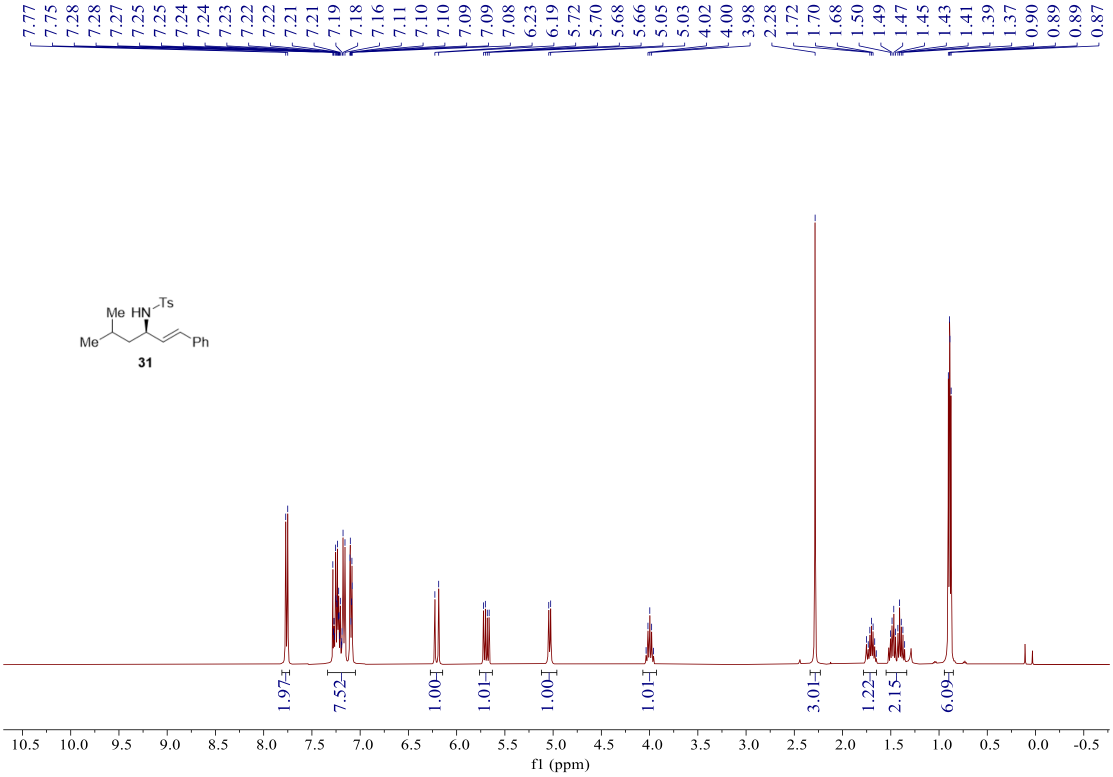

^{13}C NMR of Compound **31** ($CDCl_3$, 101MHz, 20 °C):

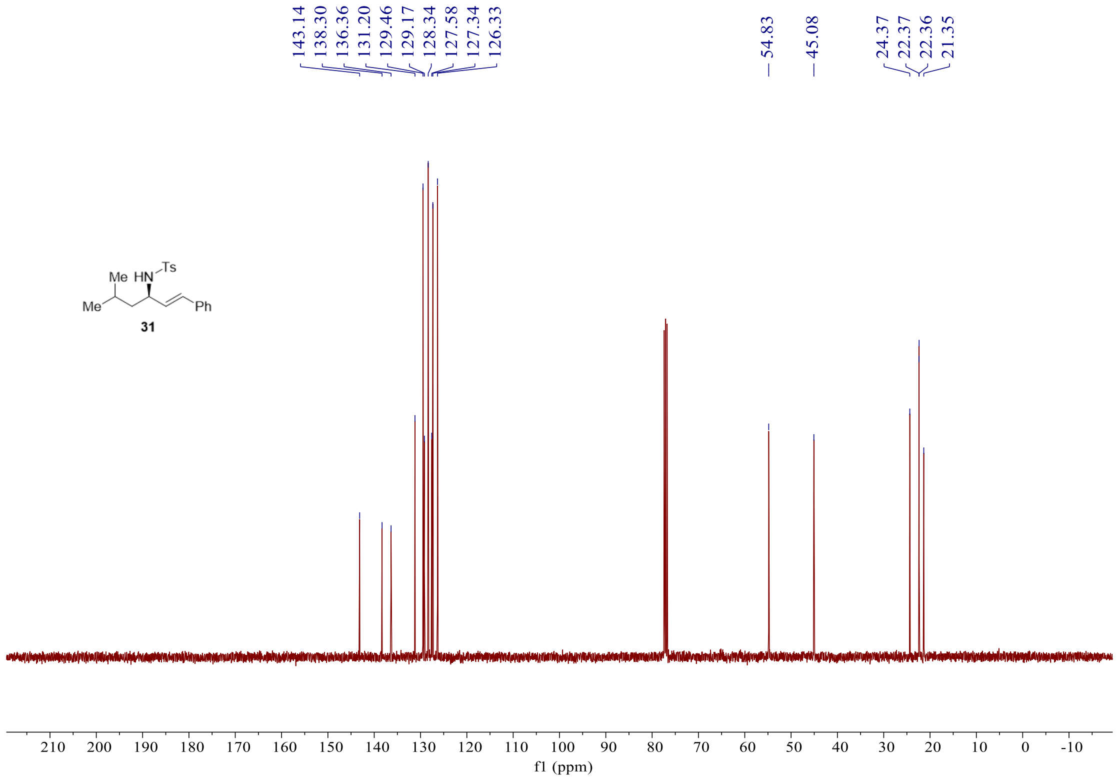

^{1}H NMR of Compound **32** ($CDCl_3$, 400 MHz, 20 °C):

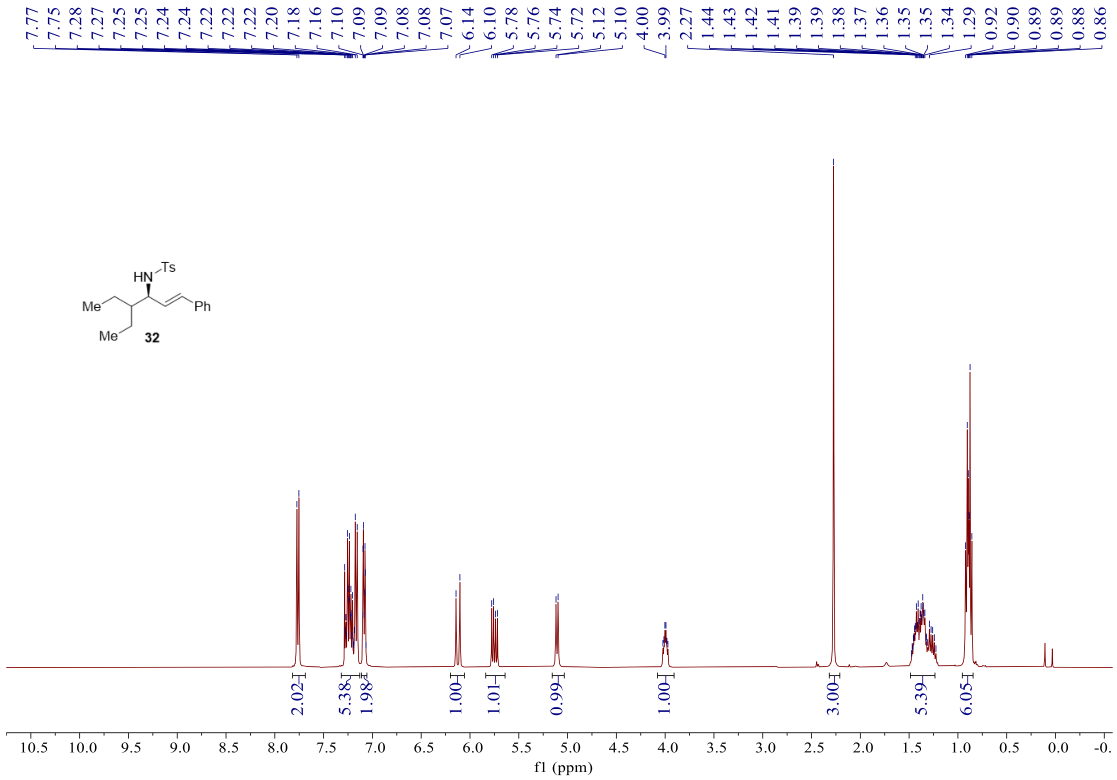

[13] C NMR of Compound **32** ($CDCl_3$, 101MHz, 20 °C):

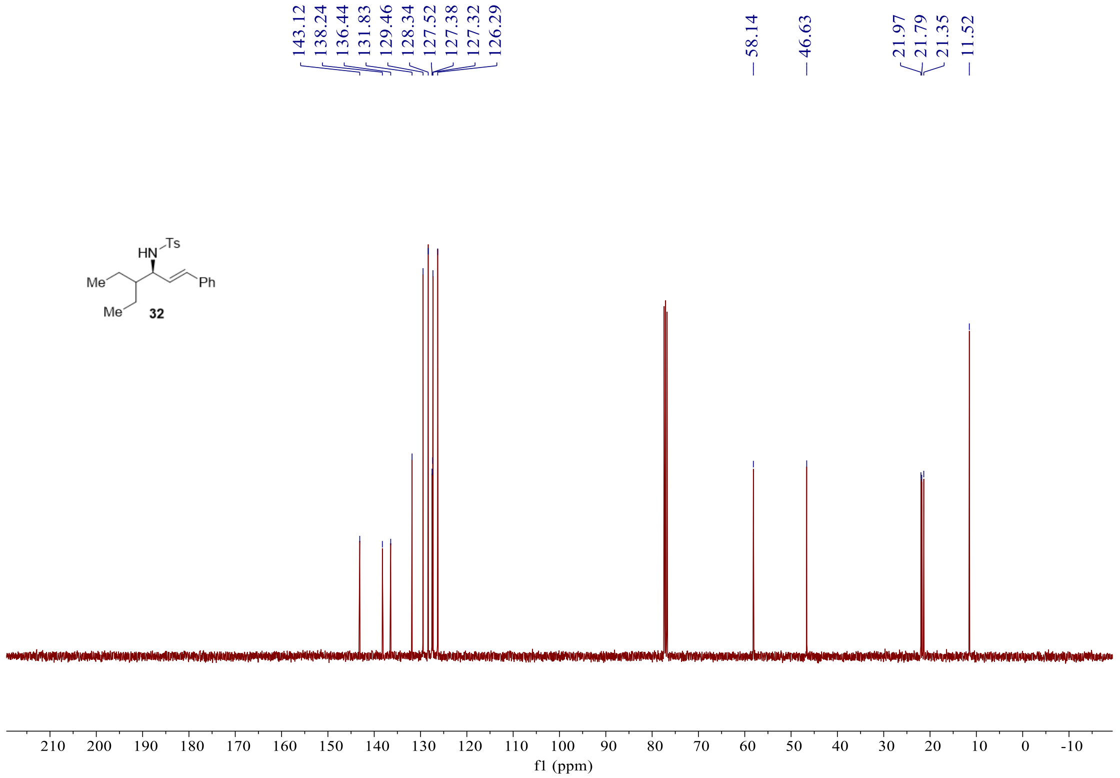

[1] H NMR of Compound **33** ($CDCl_3$, 400 MHz, 20 °C):

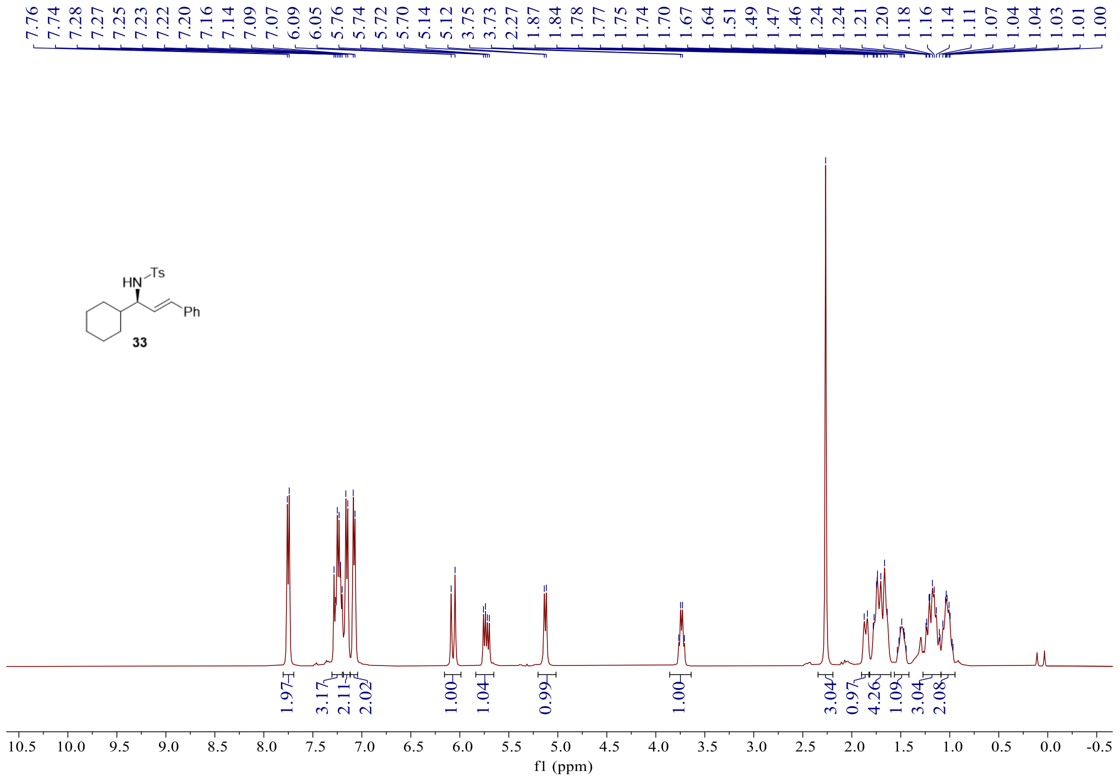

^{13}C NMR of Compound **33** ($CDCl_3$, 101MHz, 20 °C):

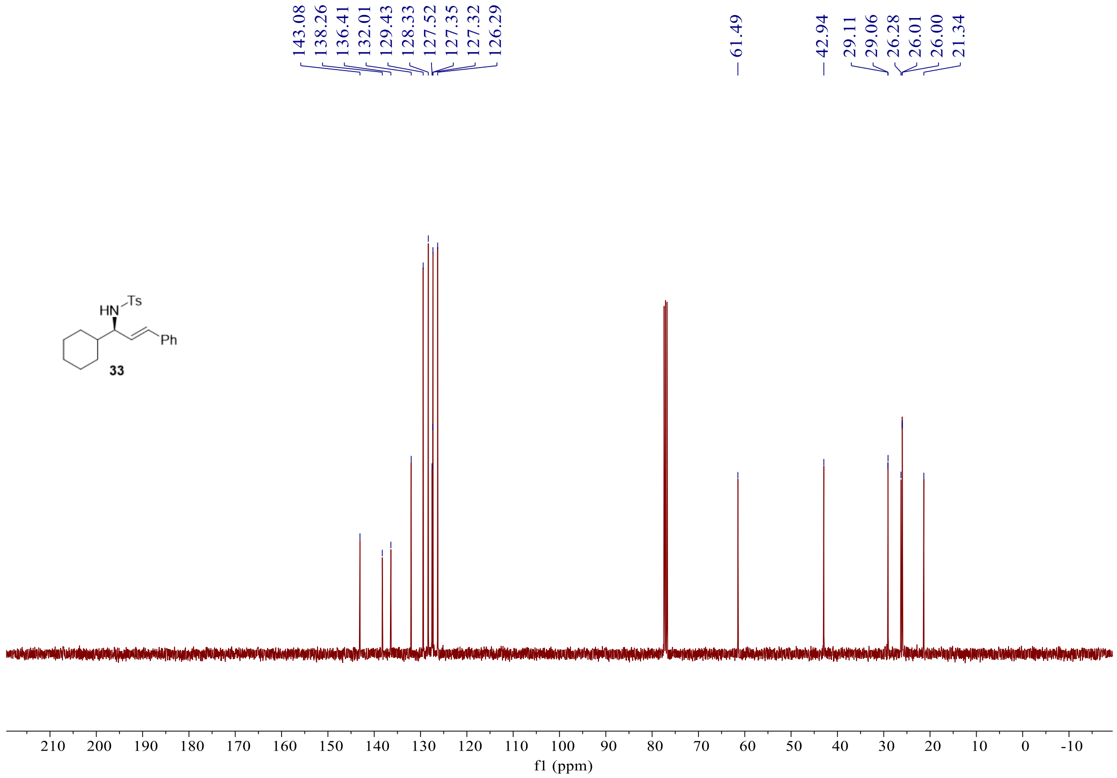

^{1}H NMR of Compound **34** ($CDCl_3$, 400 MHz, 20 °C):

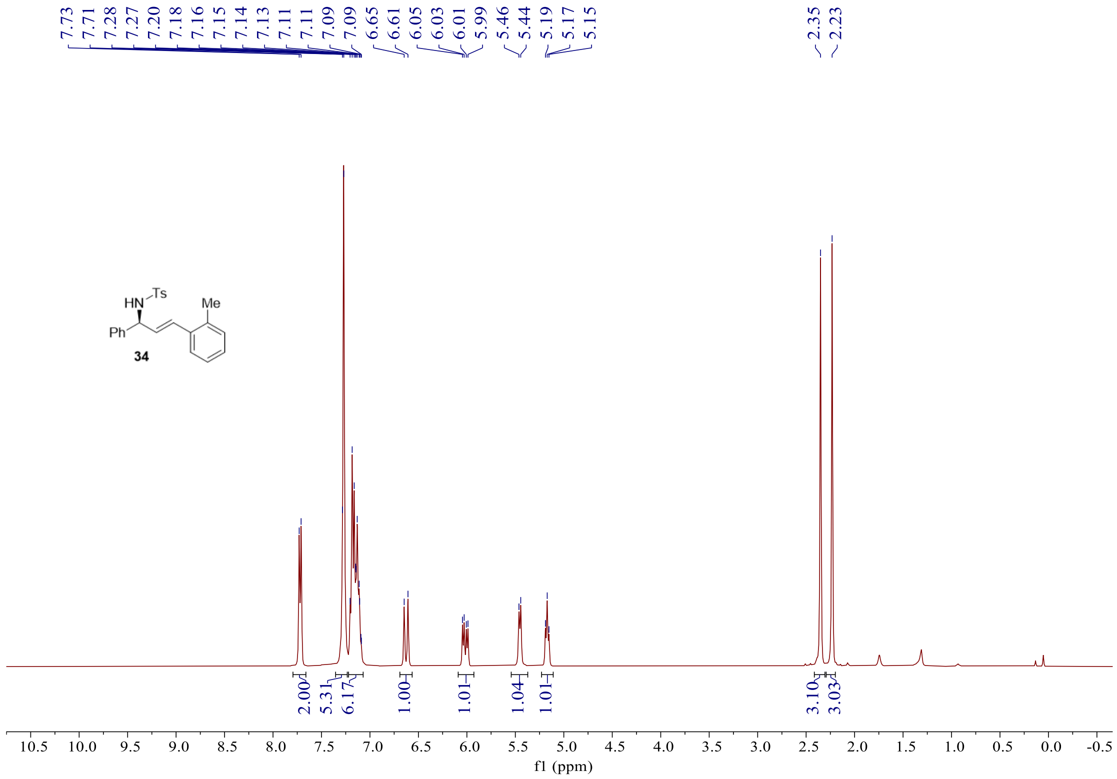

^{13}C NMR of Compound **34** ($CDCl_3$, 101MHz, 20 °C):

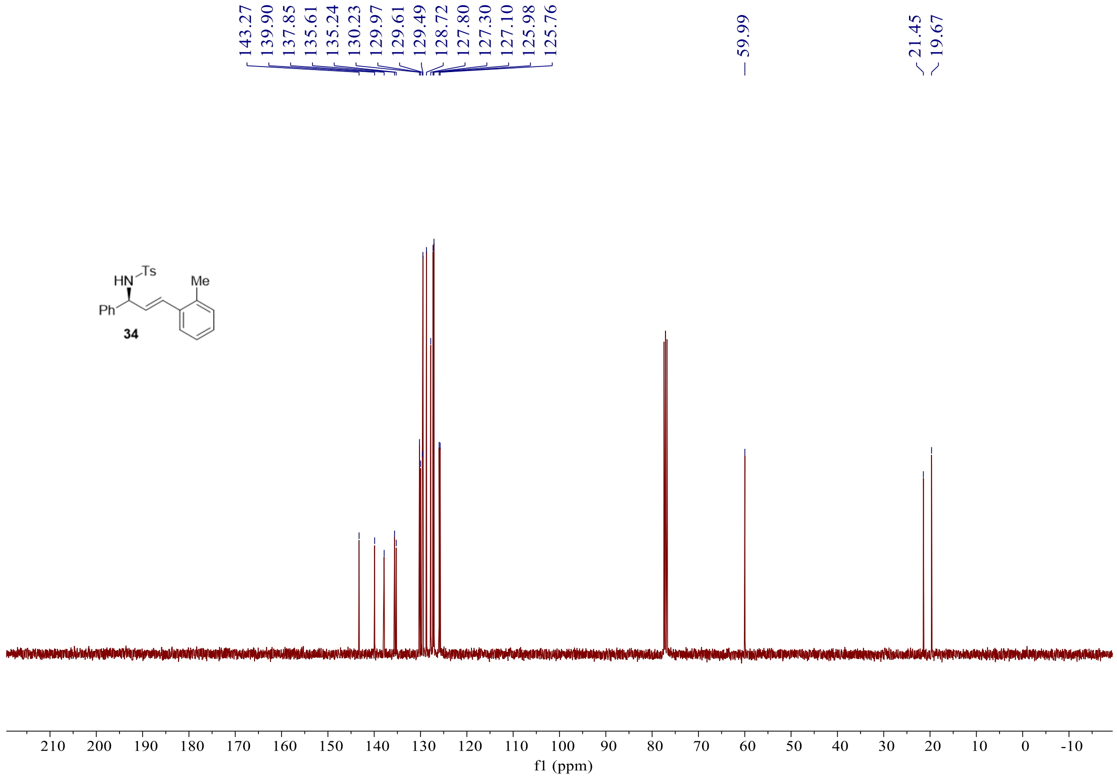

^{1}H NMR of Compound **35** ($CDCl_3$, 400 MHz, 20 °C):

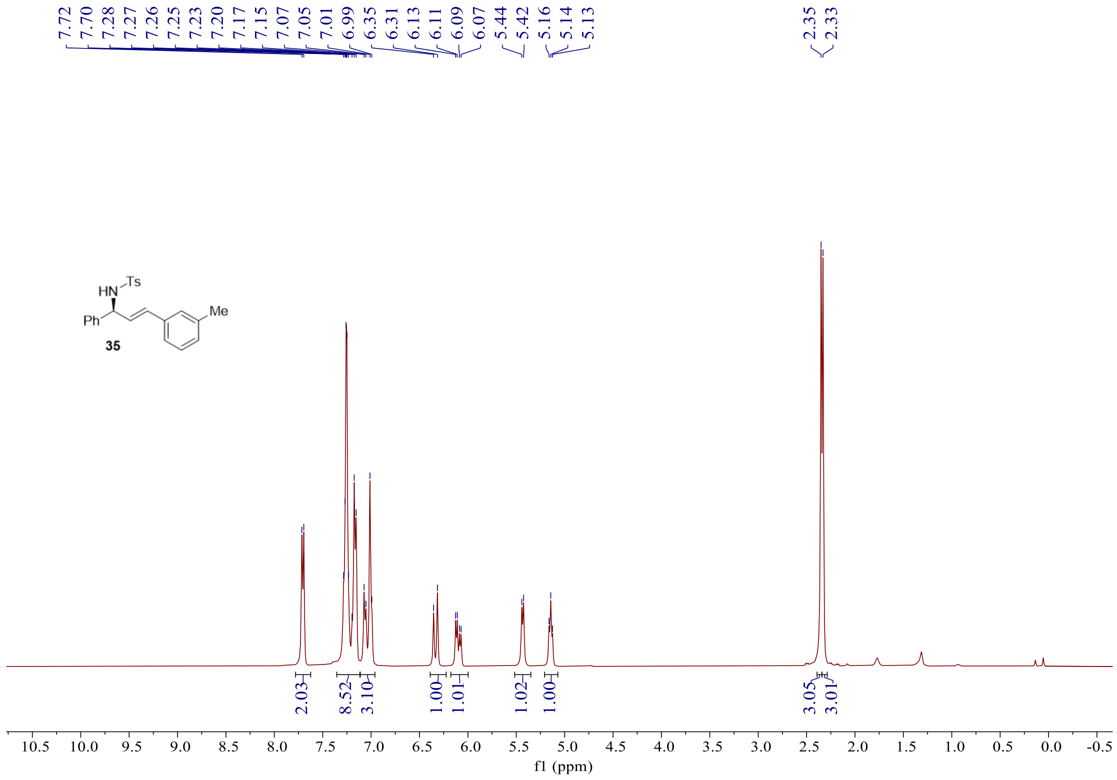

^{13}C NMR of Compound **35** ($CDCl_3$, 101MHz, 20 °C):

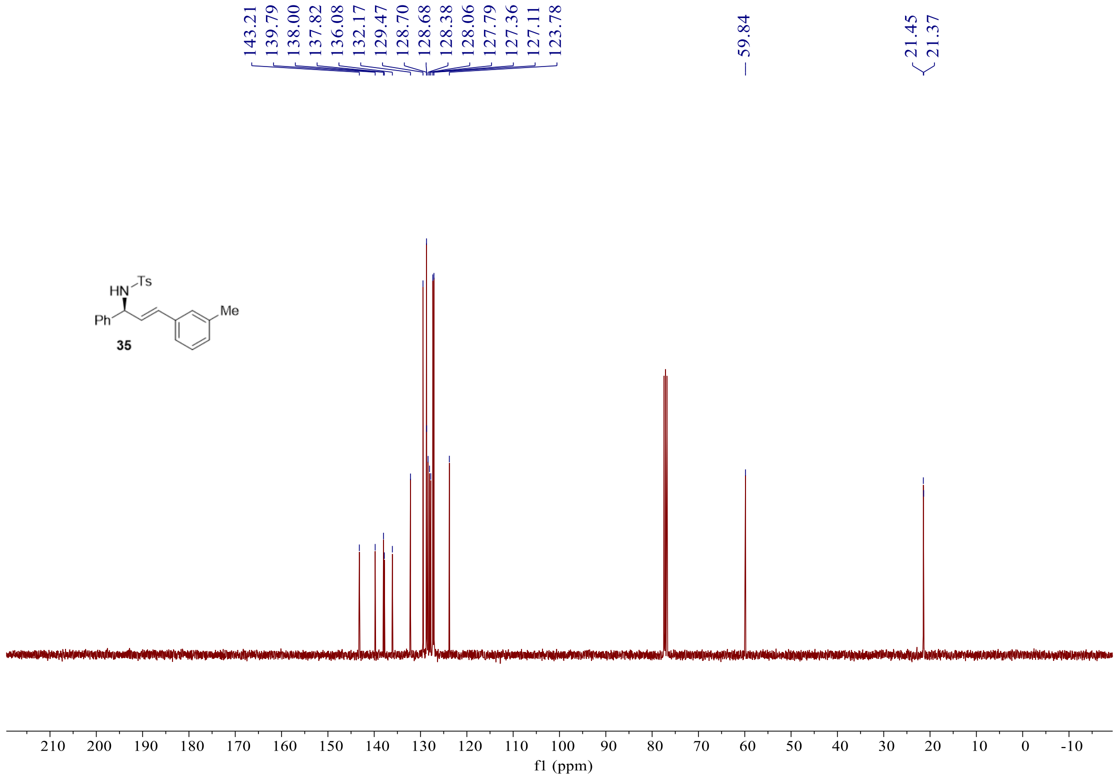

^{1}H NMR of Compound **36** ($CDCl_3$, 400 MHz, 20 °C):

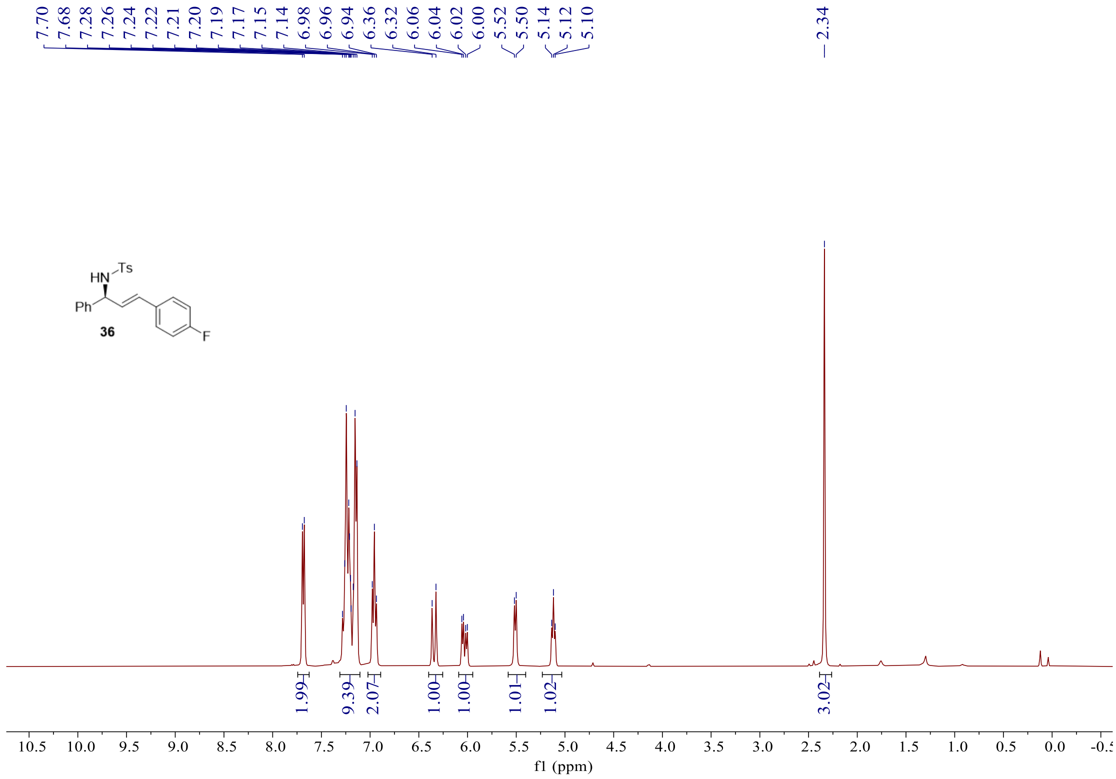

^{13}C NMR of Compound **36** ($CDCl_3$, 101MHz, 20 °C):

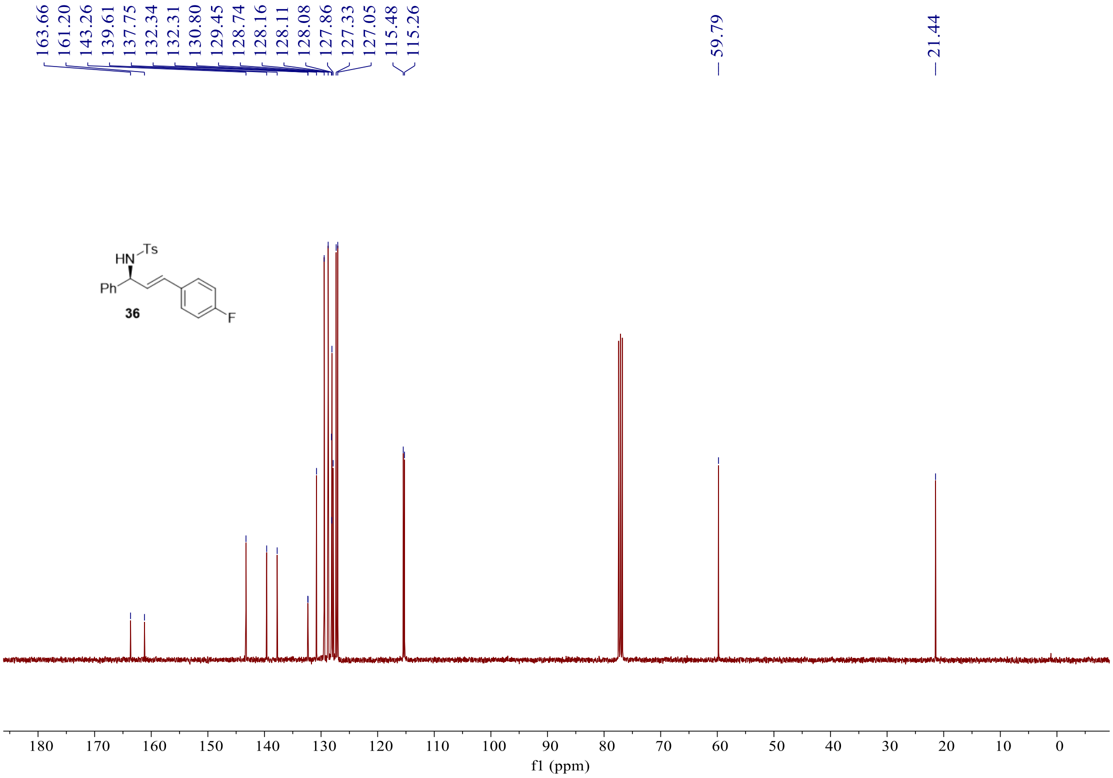

^{19}F NMR of Compound **36** ($CDCl_3$, 376MHz, 20 °C):

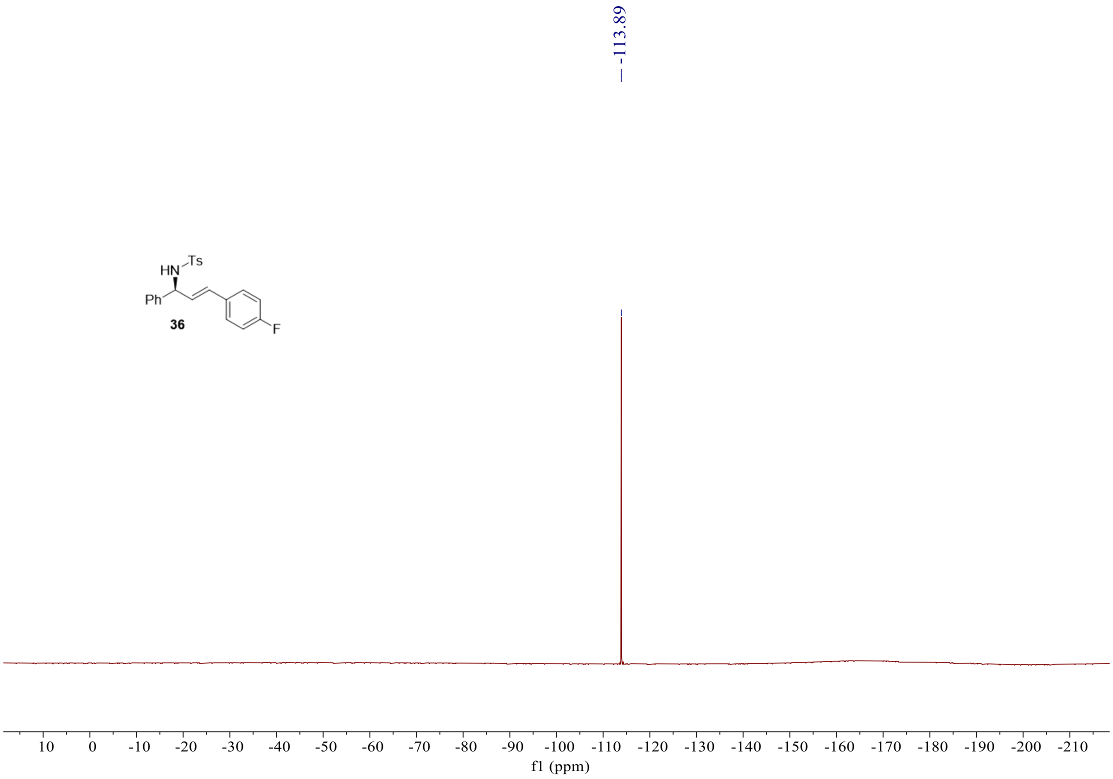

^{1}H NMR of Compound **37** ($CDCl_3$, 400 MHz, 20 °C):

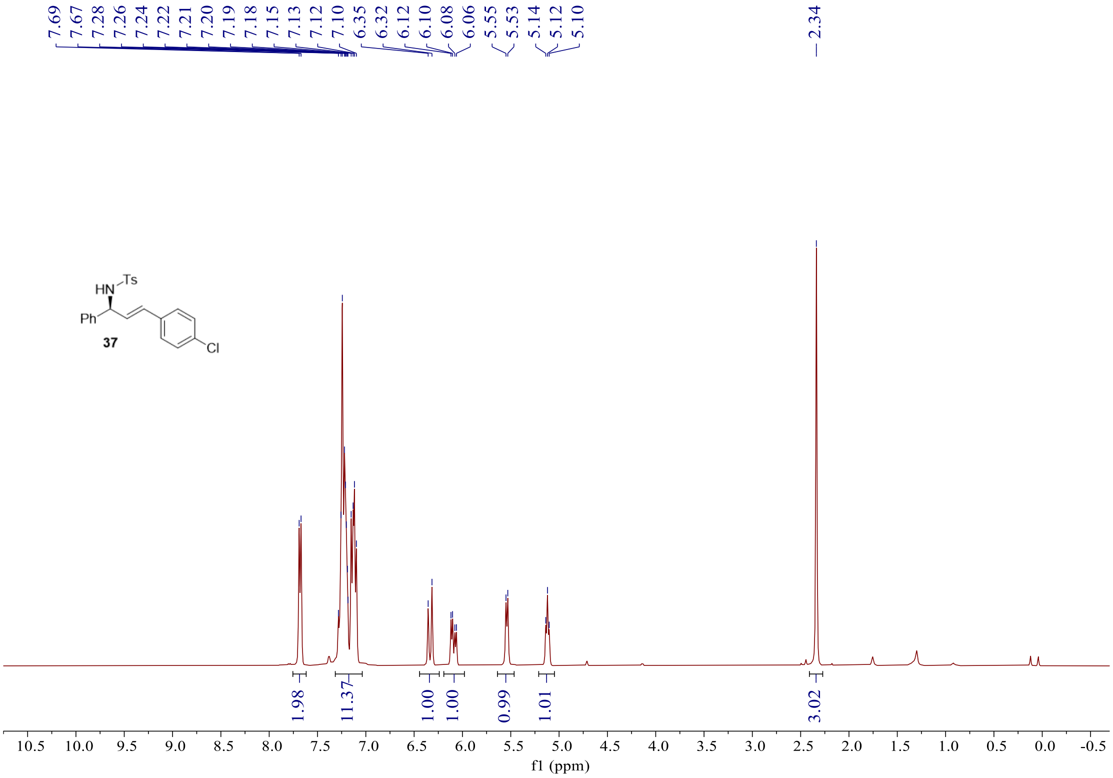

^{13}C NMR of Compound **37** ($CDCl_3$, 101MHz, 20 °C):

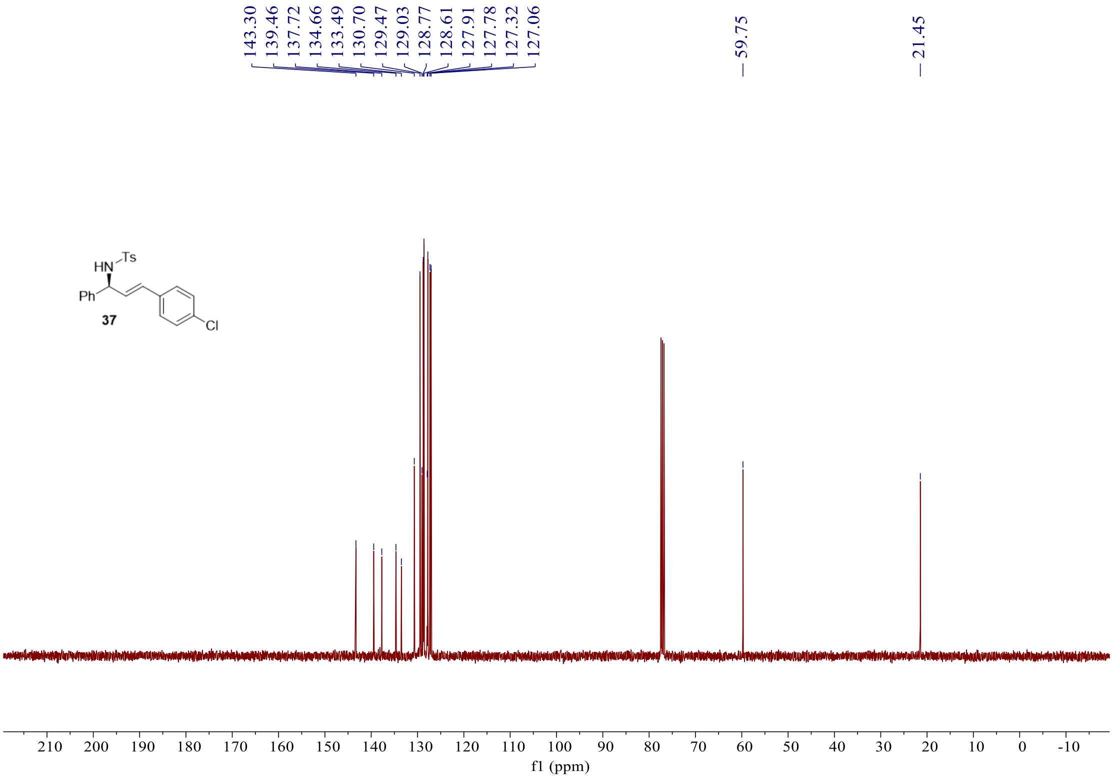

^{1}H NMR of Compound **38** ($CDCl_3$, 400 MHz, 20 °C):

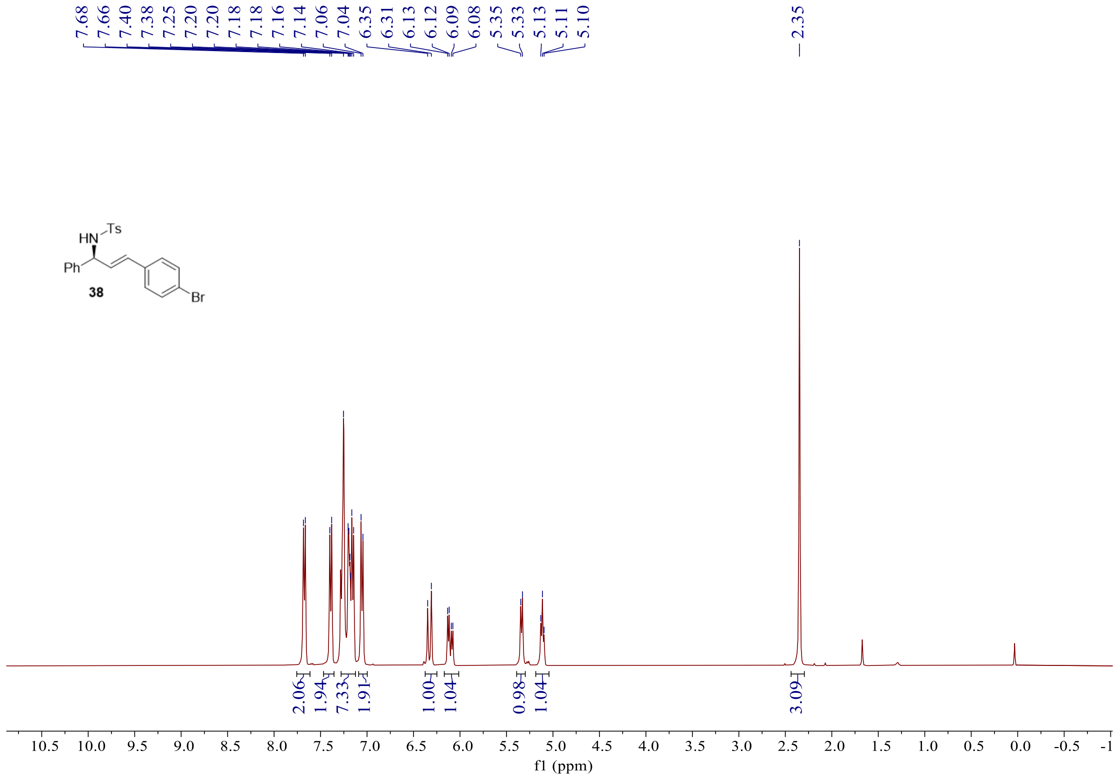

^{13}C NMR of Compound **38** ($CDCl_3$, 101MHz, 20 °C):

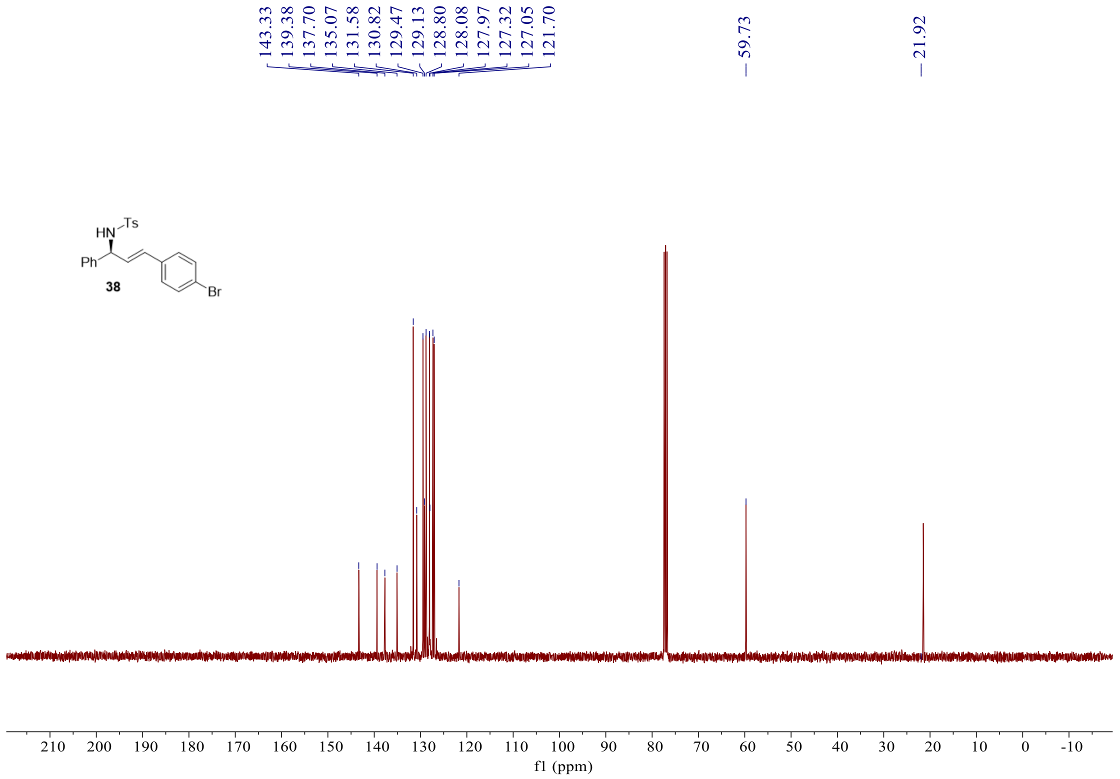

^{1}H NMR of Compound **39** ($CDCl_3$, 400 MHz, 20 °C):

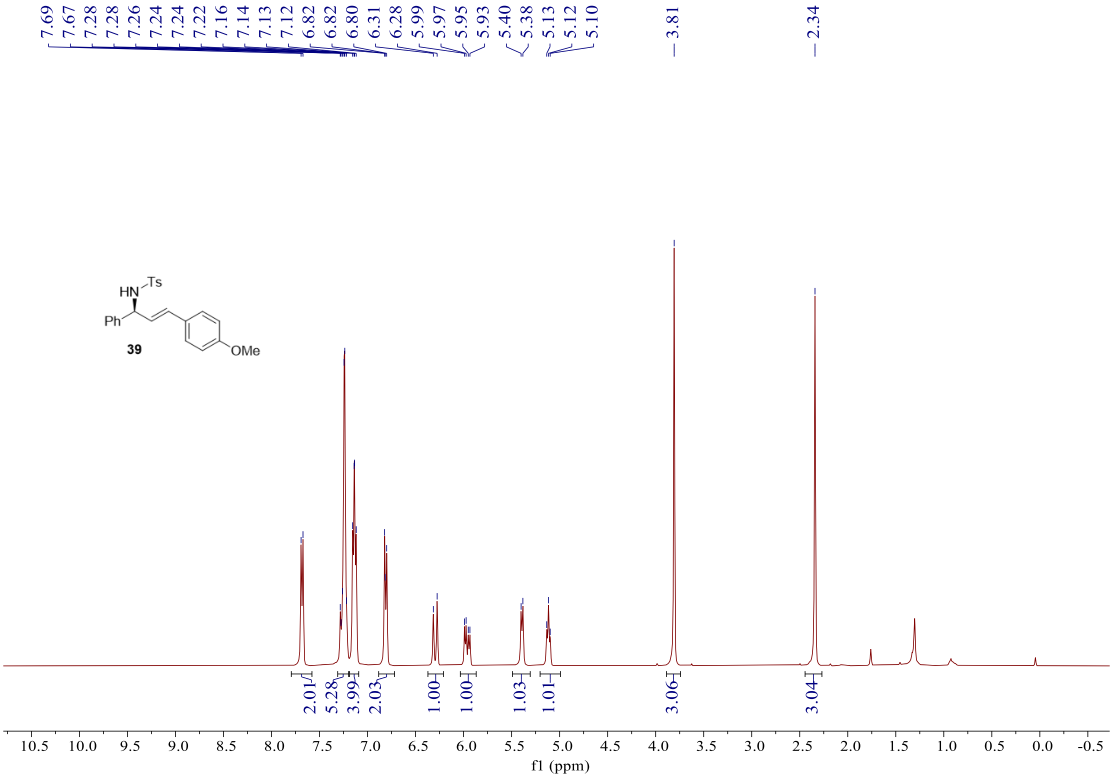

^{13}C NMR of Compound **39** ($CDCl_3$, 101MHz, 20 °C):

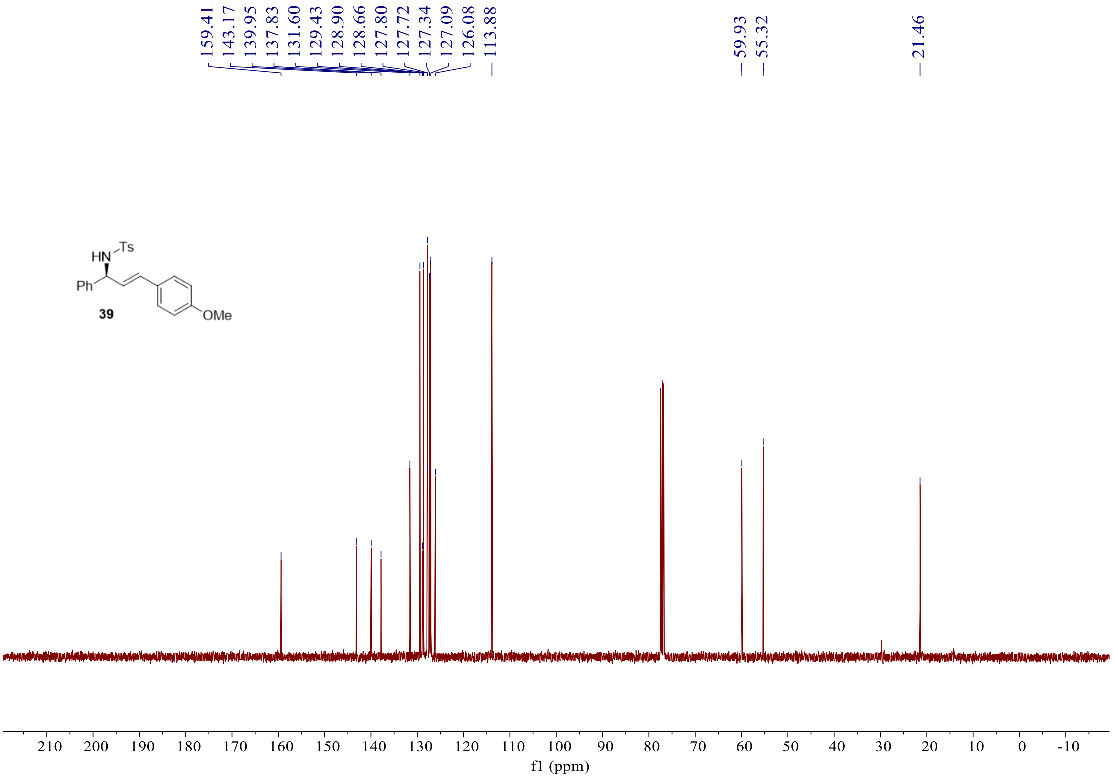

^{1}H NMR of Compound **40** ($CDCl_3$, 400 MHz, 20 °C):

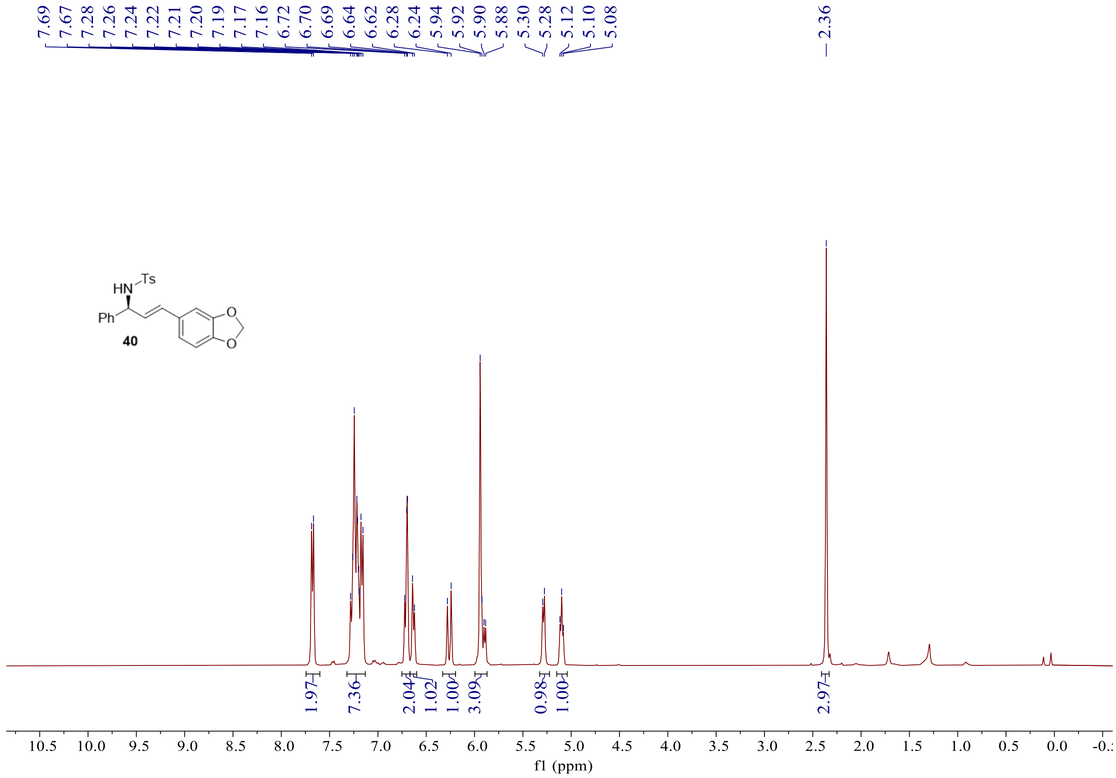

^{13}C NMR of Compound **40** ($CDCl_3$, 101MHz, 20 °C):

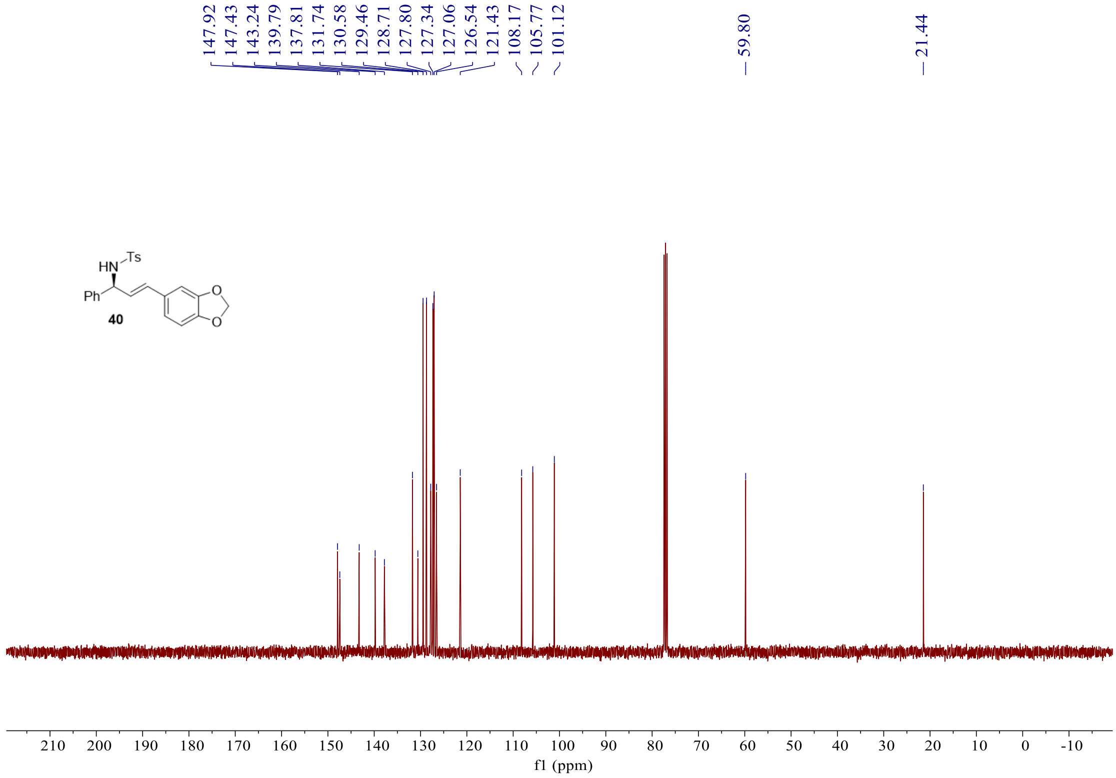

^{1}H NMR of Compound **41** ($CDCl_3$, 400 MHz, 20 °C):

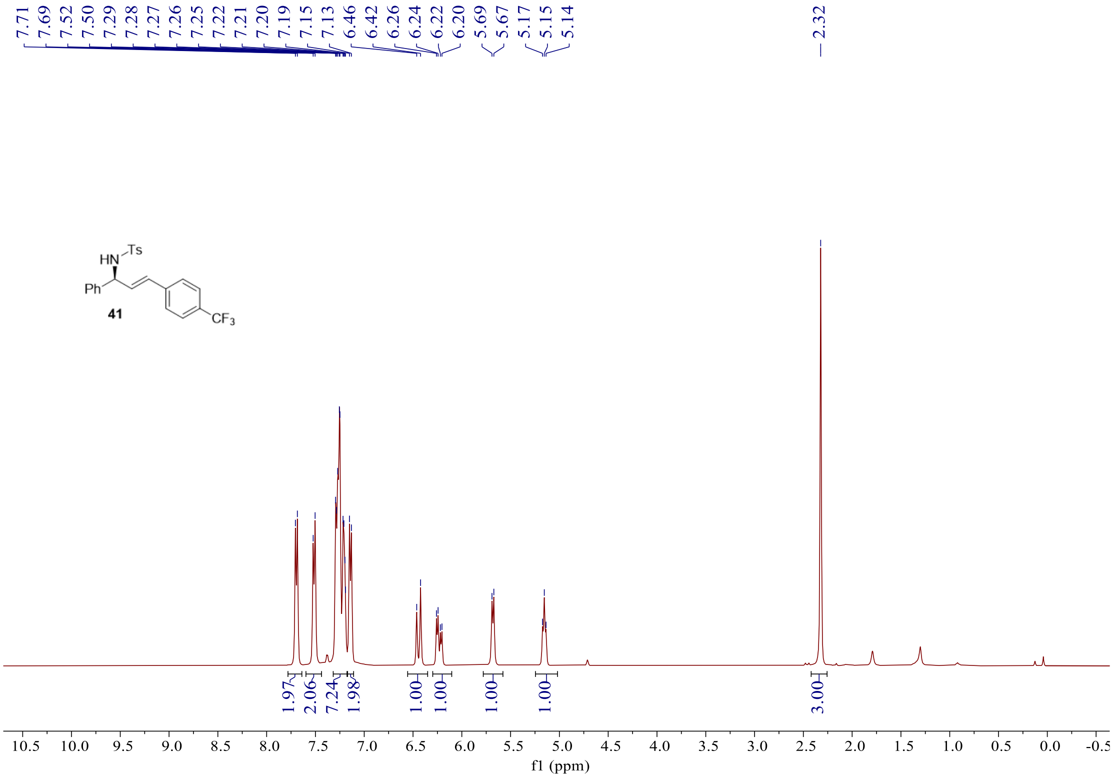

^{13}C NMR of Compound **41** ($CDCl_3$, 101MHz, 20 °C):

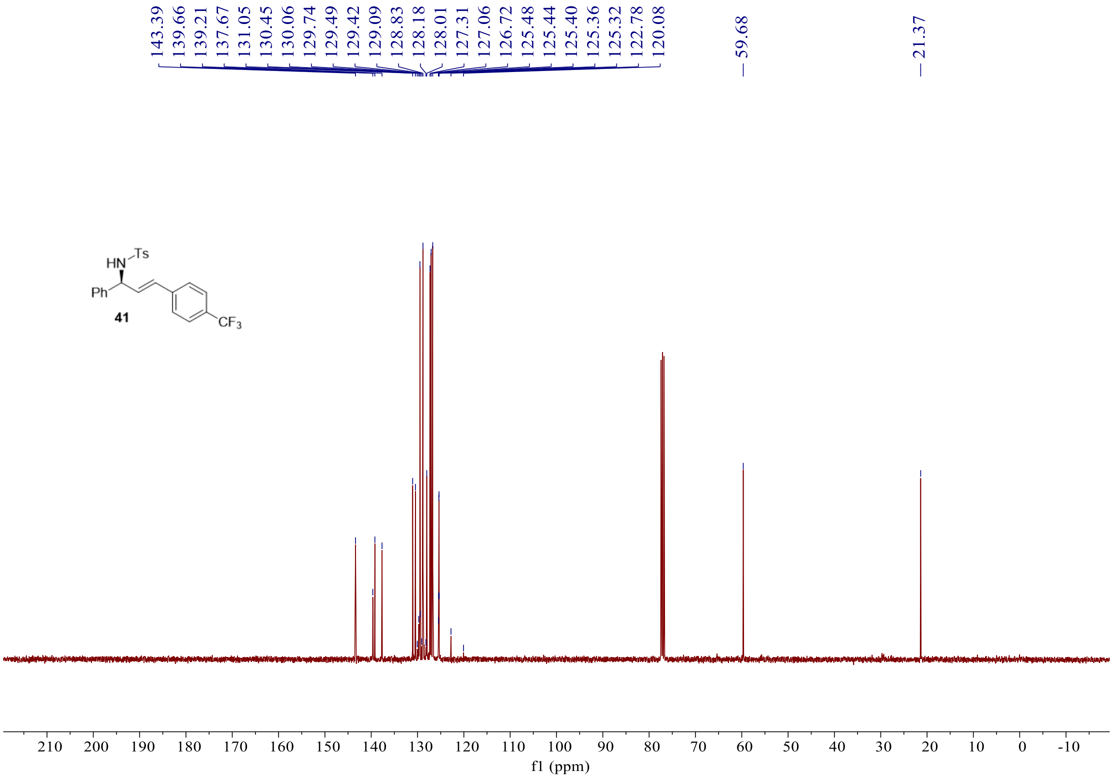

^{19}F NMR of Compound **41** ($CDCl_3$, 376MHz, 20 °C):

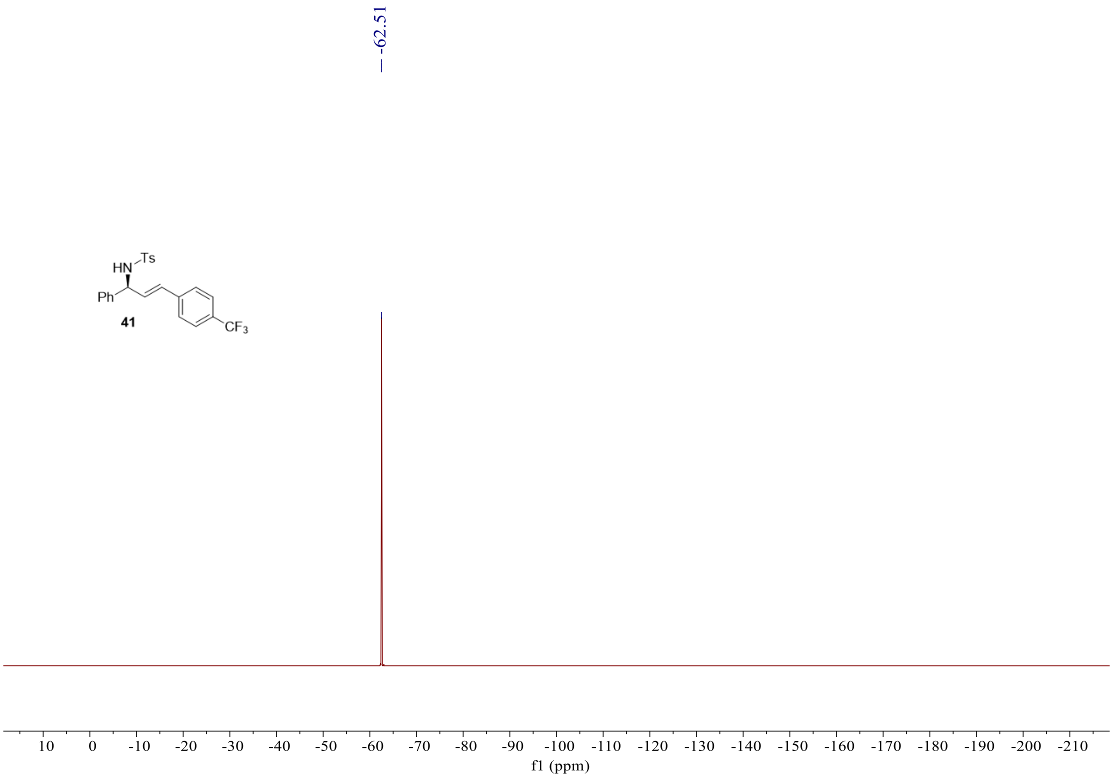

^{1}H NMR of Compound **42** ($CDCl_3$, 400 MHz, 20 °C):

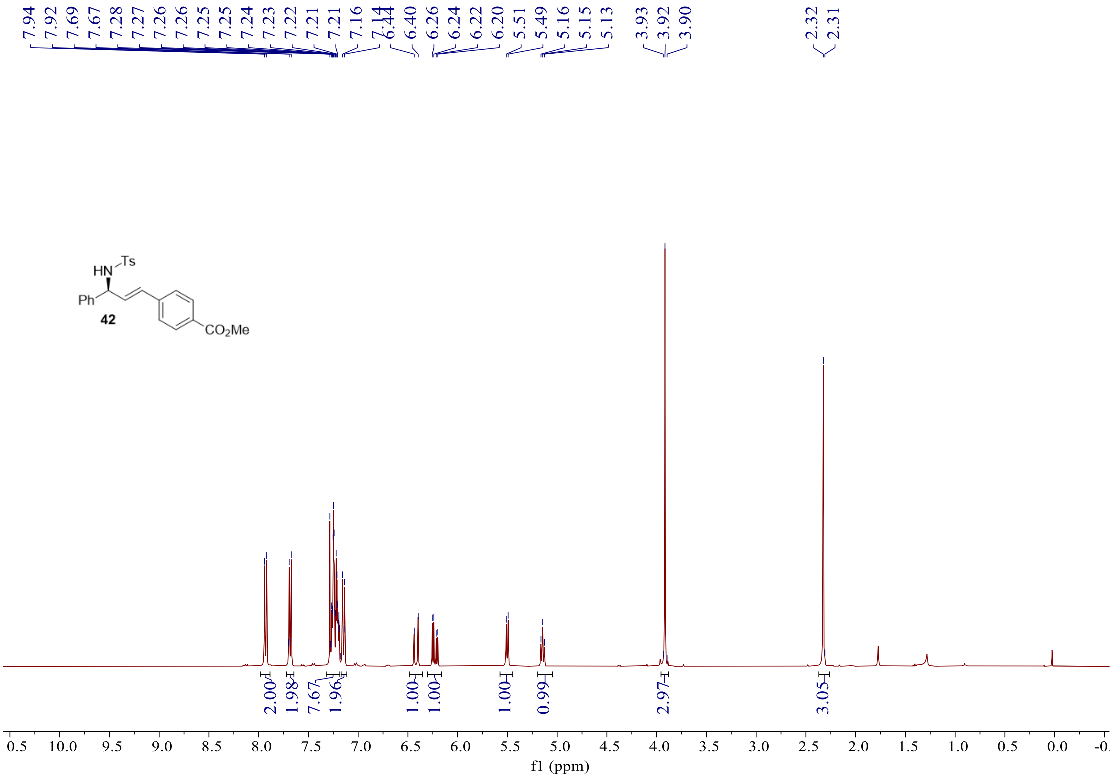

^{13}C NMR of Compound **42** ($CDCl_3$, 101MHz, 20 °C):

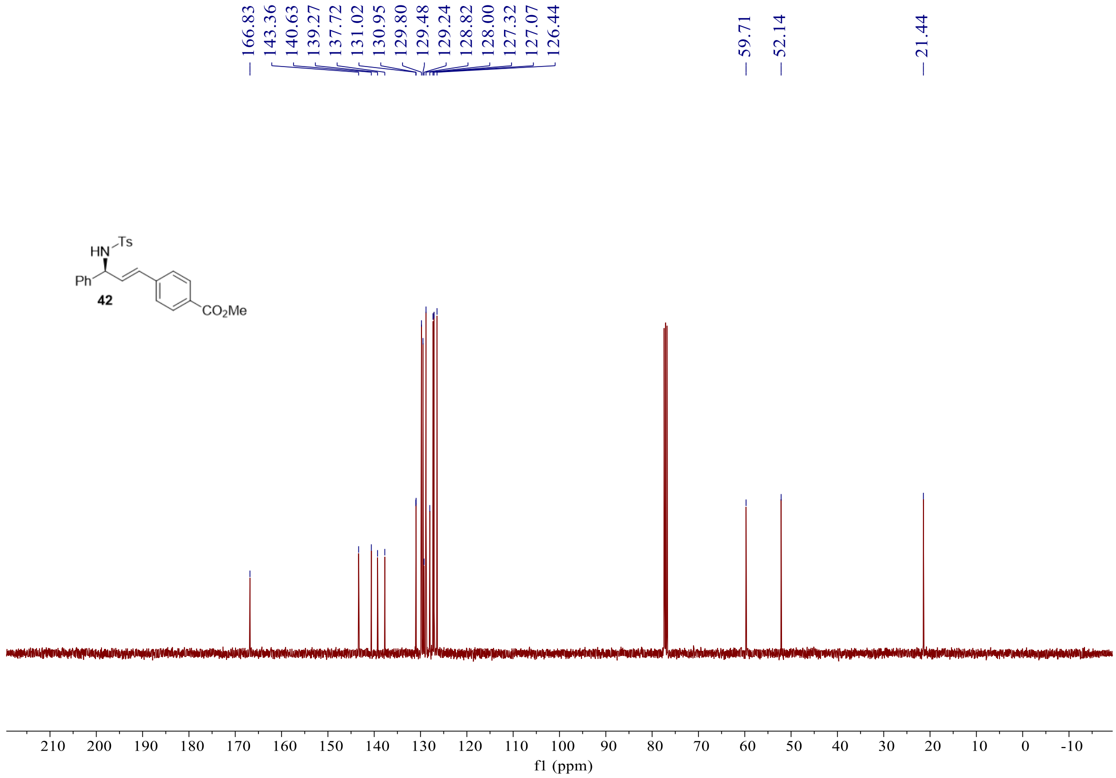

^{1}H NMR of Compound **43** ($CDCl_3$, 400 MHz, 20 °C):

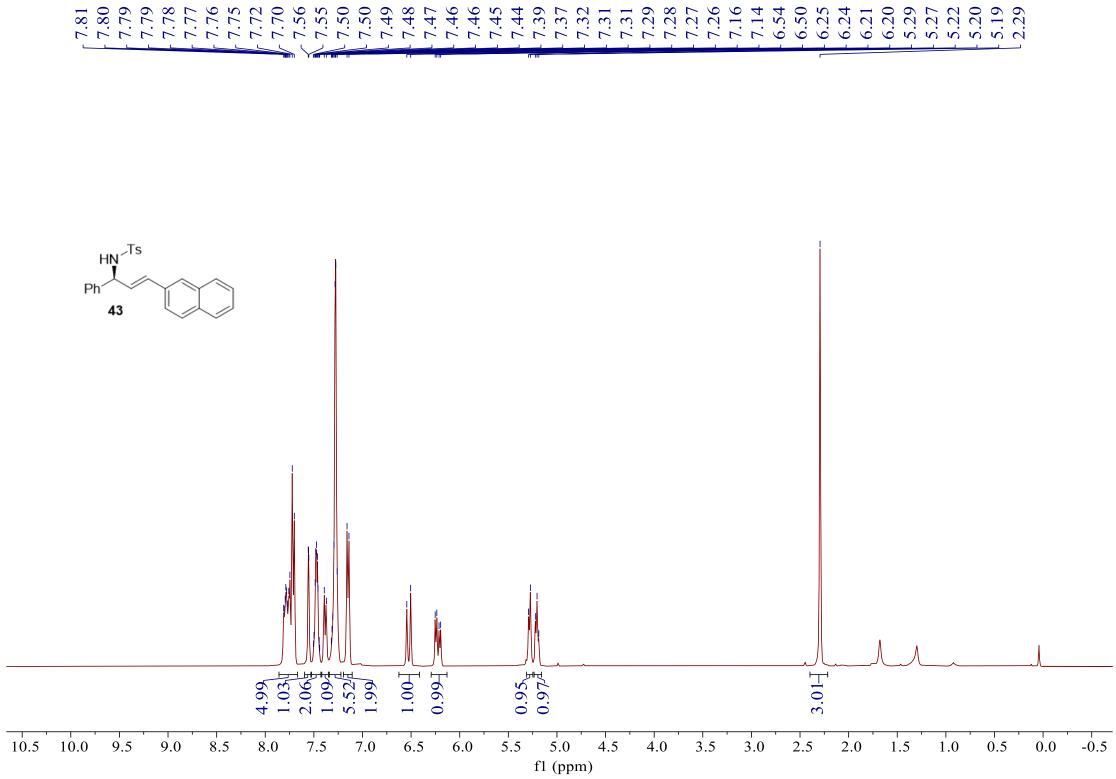

^{13}C NMR of Compound **43** ($CDCl_3$, 101MHz, 20 °C):

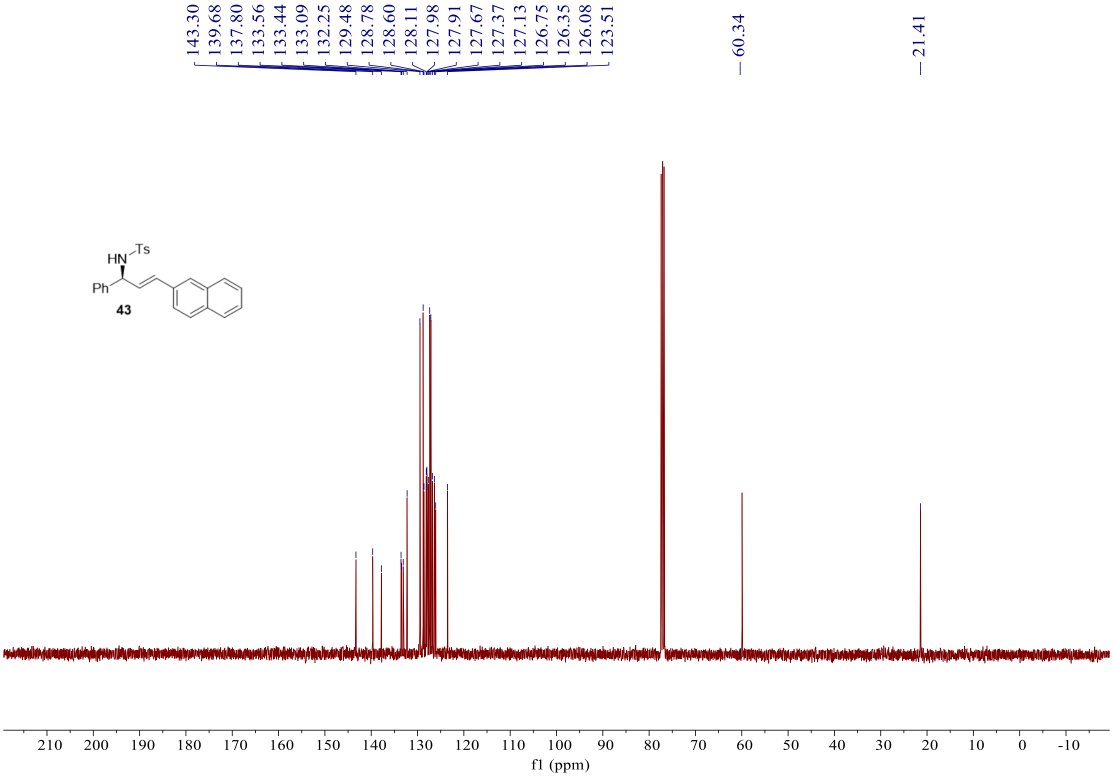

^{1}H NMR of Compound **44** ($CDCl_3$, 400 MHz, 20 °C):

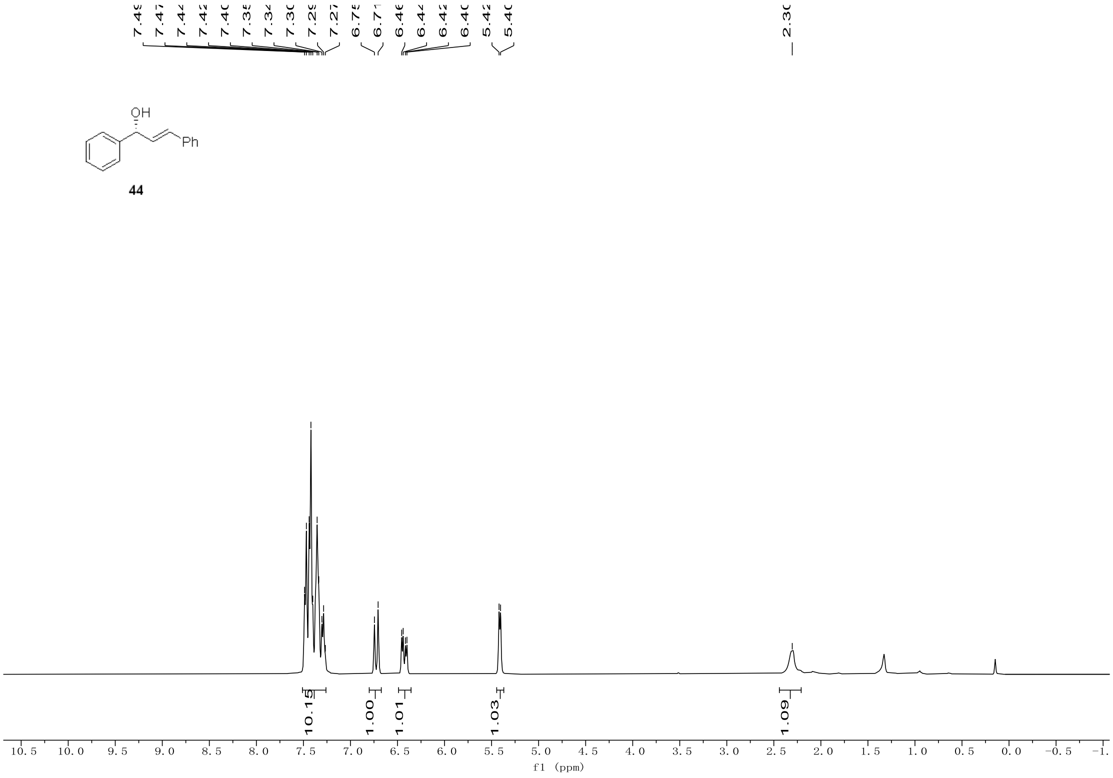

^{13}C NMR of Compound **44** ($CDCl_3$, 101MHz, 20 °C):

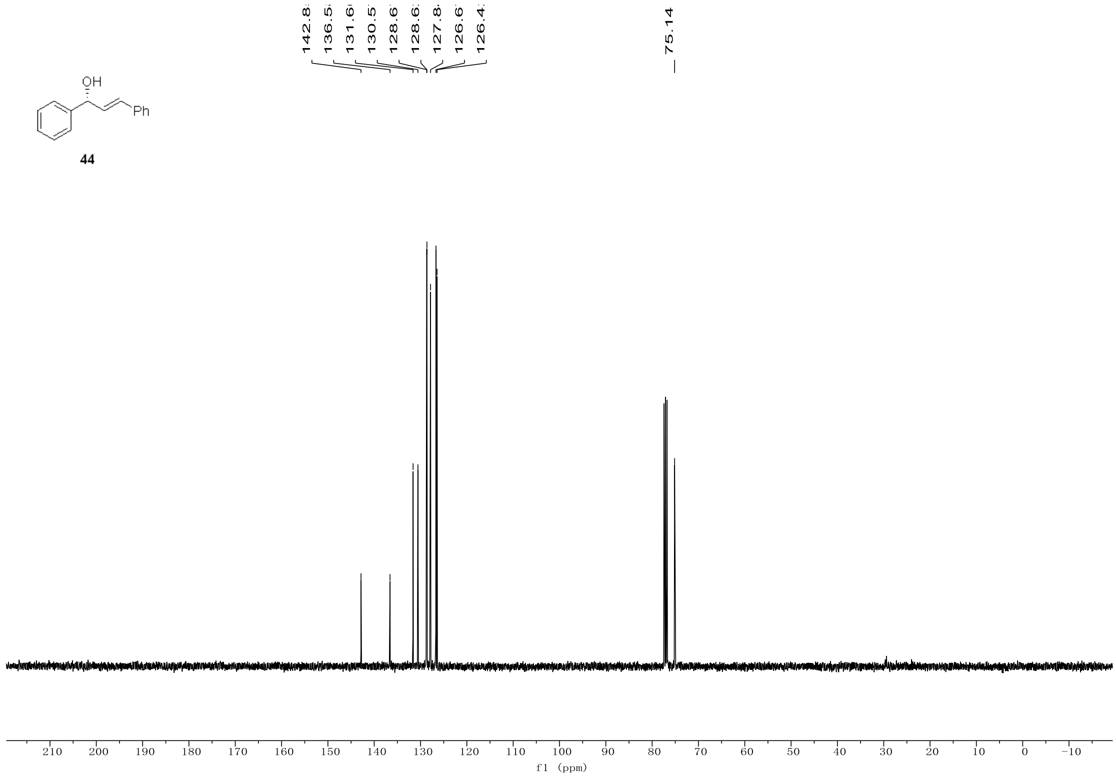

^{1}H NMR of Compound **45** ($CDCl_3$, 400 MHz, 20 °C):

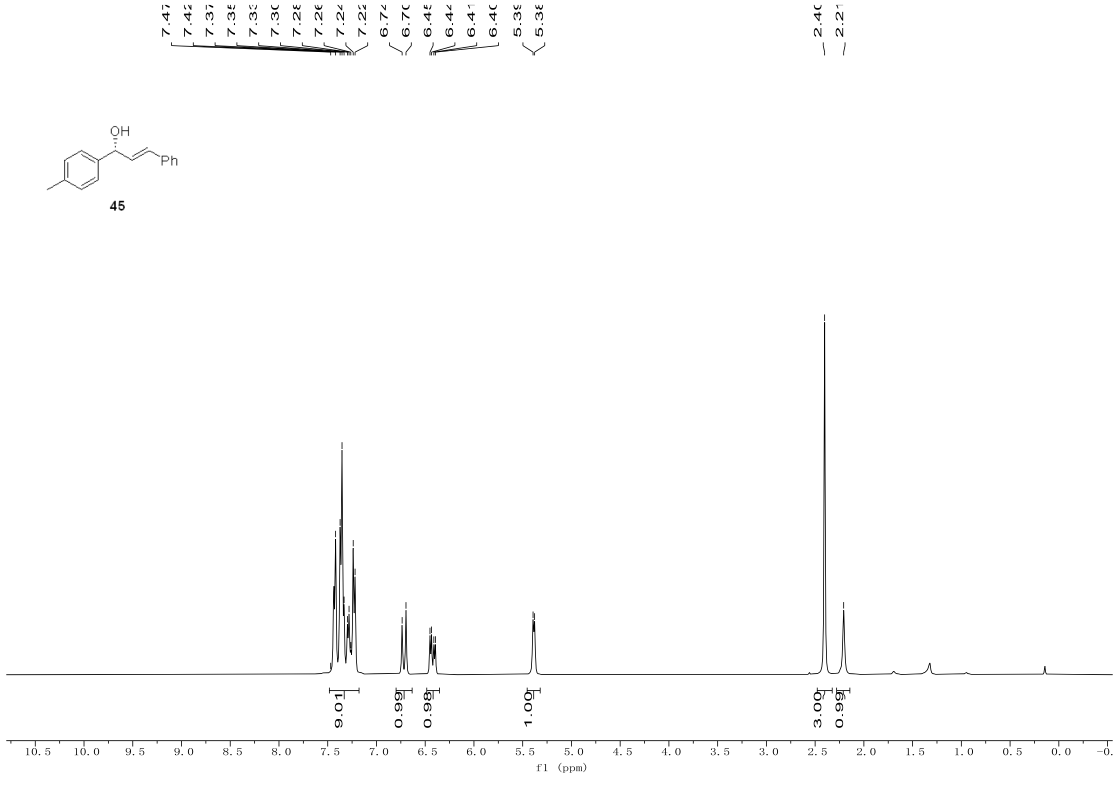

^{13}C NMR of Compound **45** ($CDCl_3$, 101MHz, 20 °C):

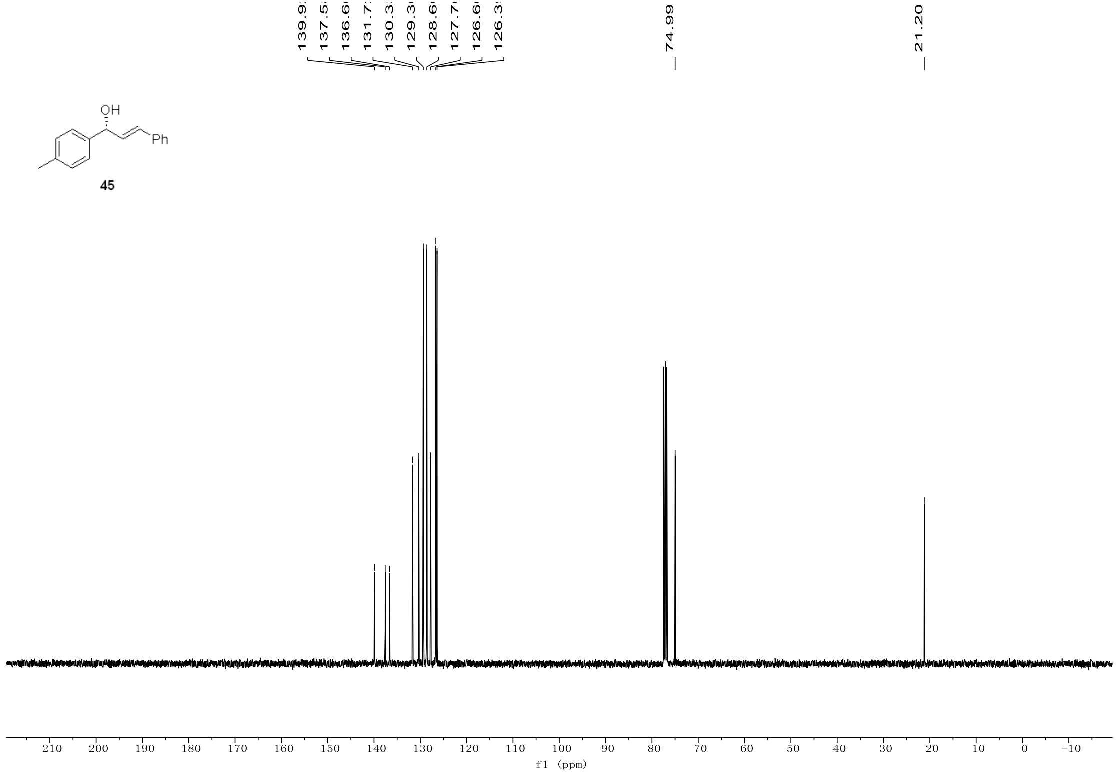

^{1}H NMR of Compound **46** ($CDCl_3$, 400 MHz, 20 °C):

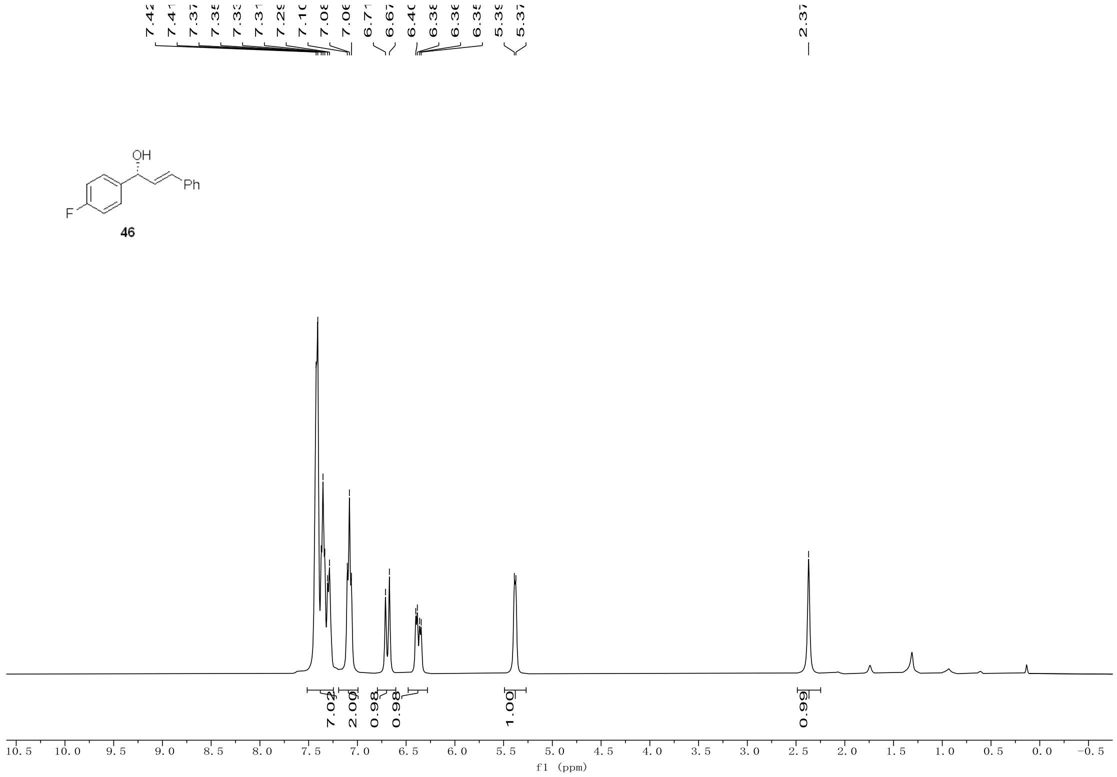

^{13}C NMR of Compound **46** ($CDCl_3$, 101MHz, 20 °C):

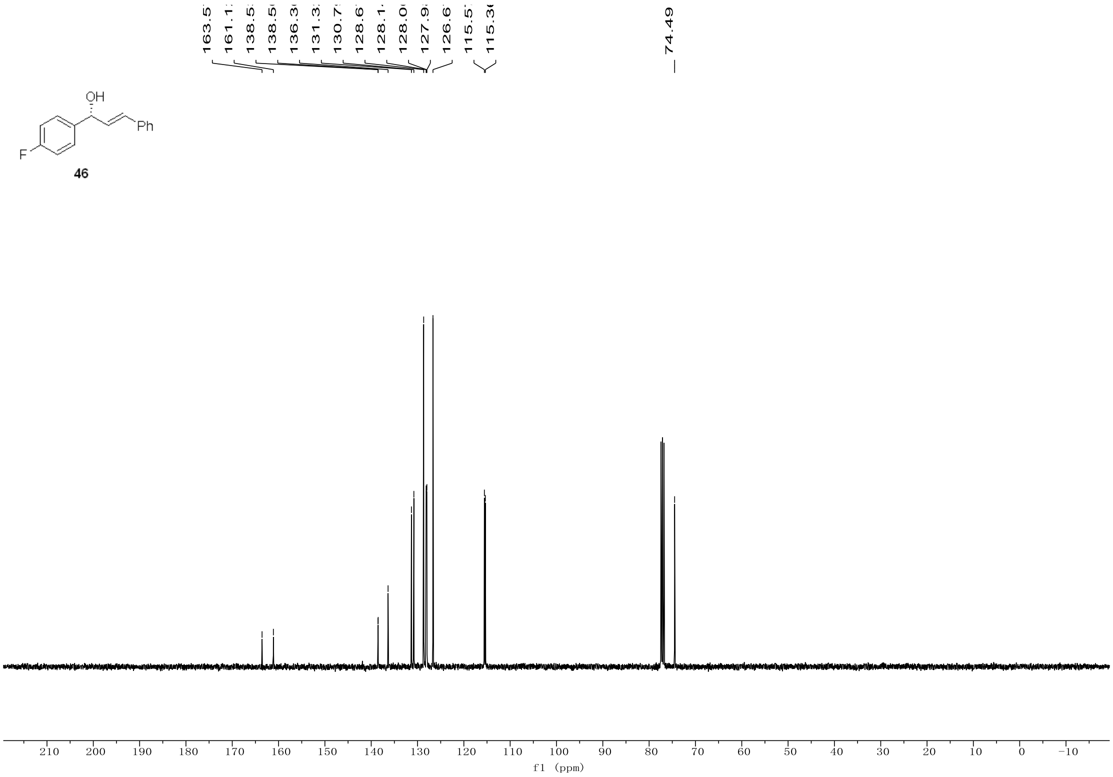

^{19}F NMR of Compound **46** ($CDCl_3$, 376MHz, 20 °C):

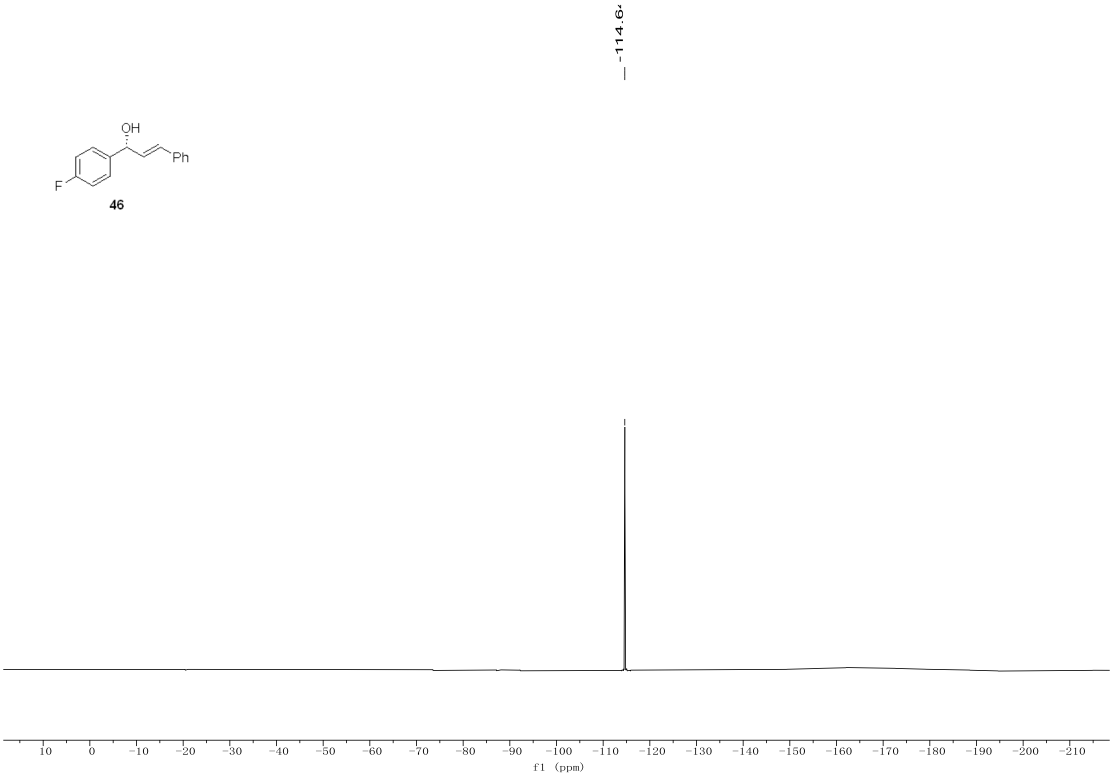

^{1}H NMR of Compound **47** ($CDCl_3$, 400 MHz, 20 °C):

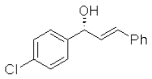

47

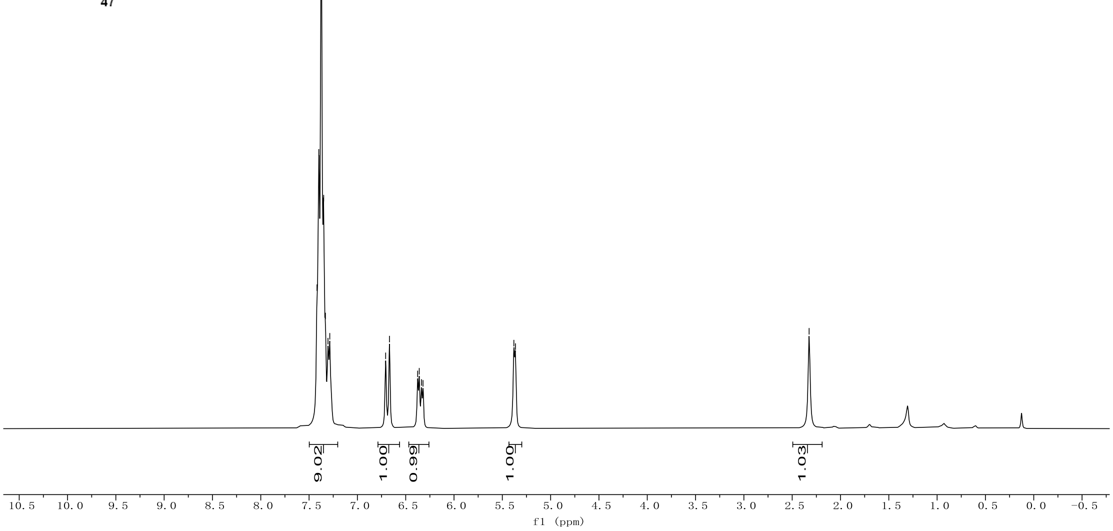

^{13}C NMR of Compound **47** ($CDCl_3$, 101MHz, 20 °C):

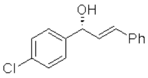

47

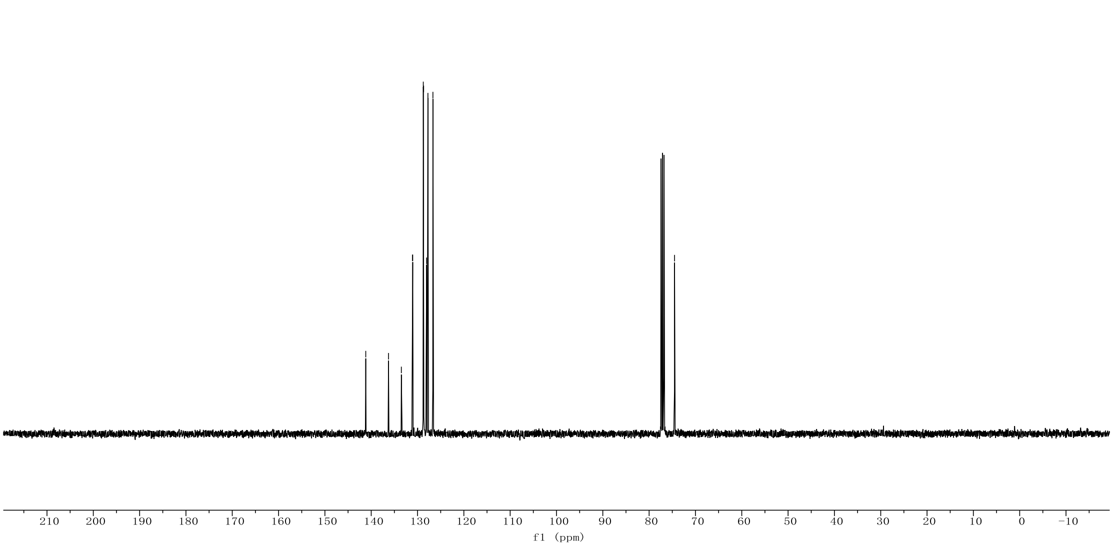

^{1}H NMR of Compound **48** ($CDCl_3$, 400 MHz, 20 °C):

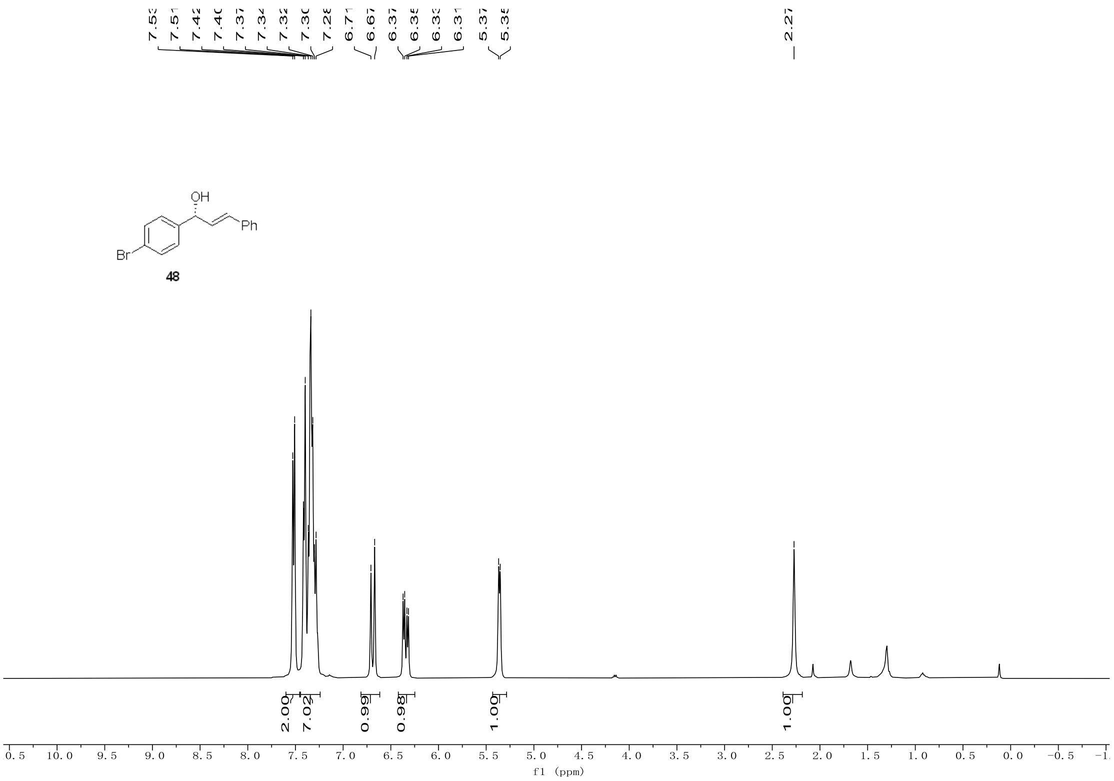

^{13}C NMR of Compound **48** ($CDCl_3$, 101MHz, 20 °C):

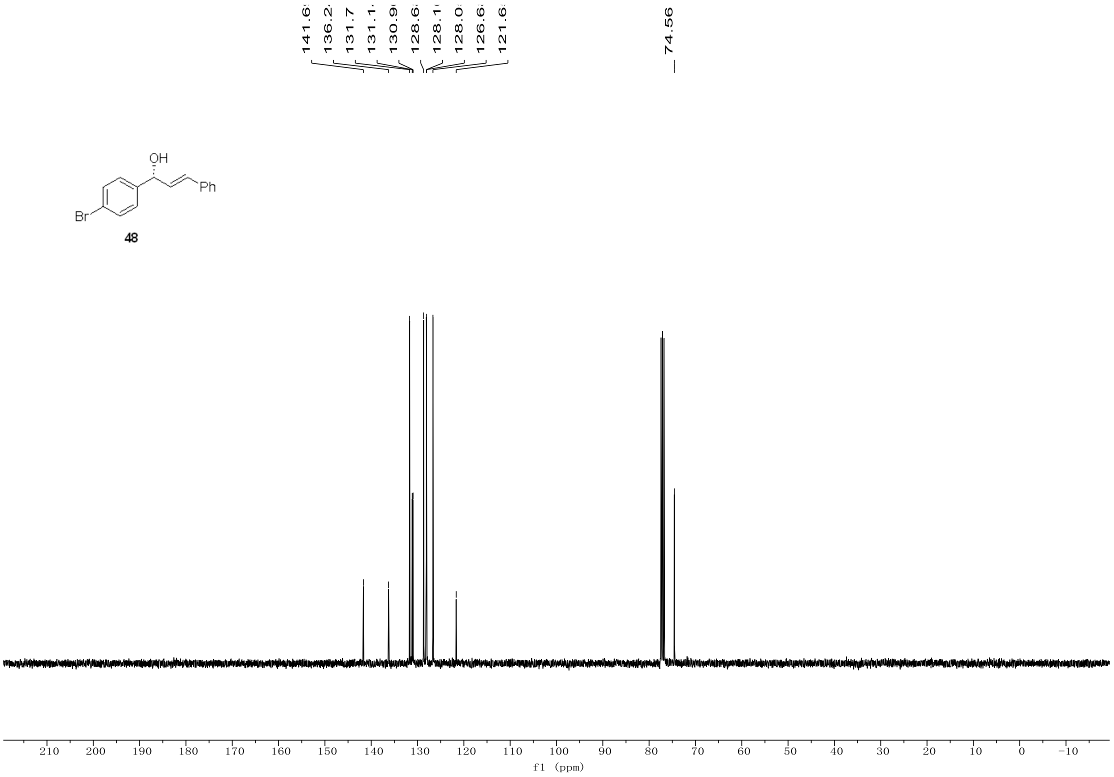

^{1}H NMR of Compound **49** ($CDCl_3$, 400 MHz, 20 °C):

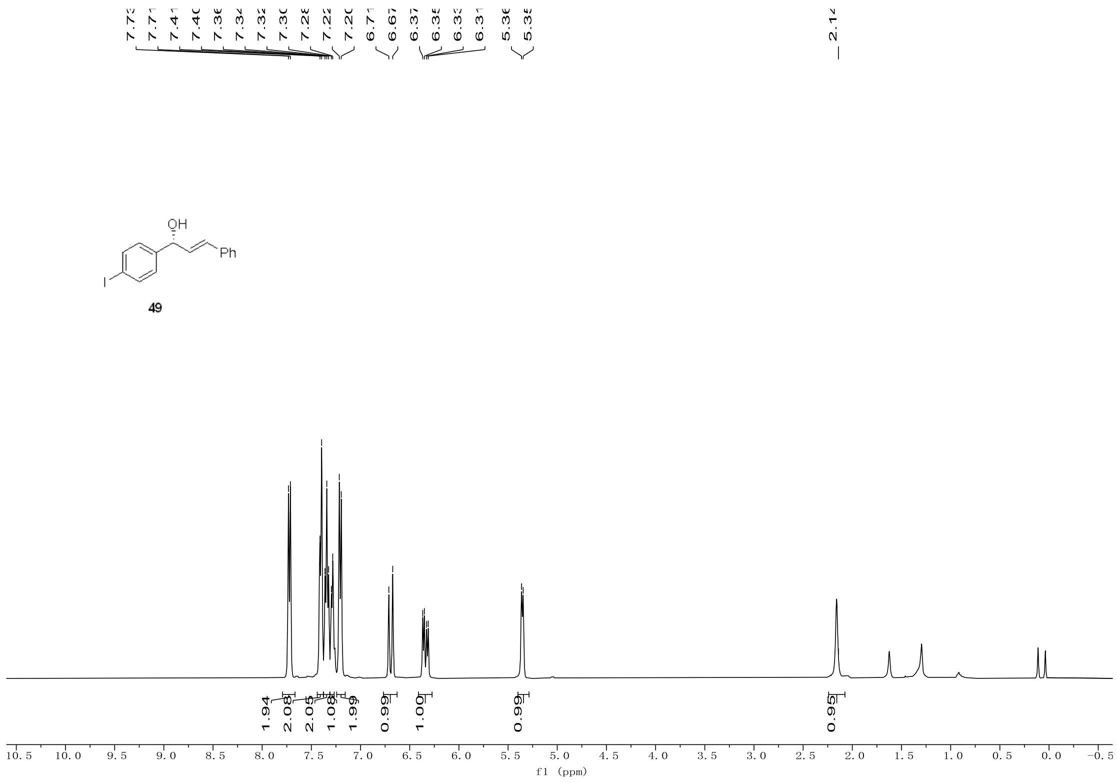

^{13}C NMR of Compound **49** ($CDCl_3$, 101MHz, 20 °C):

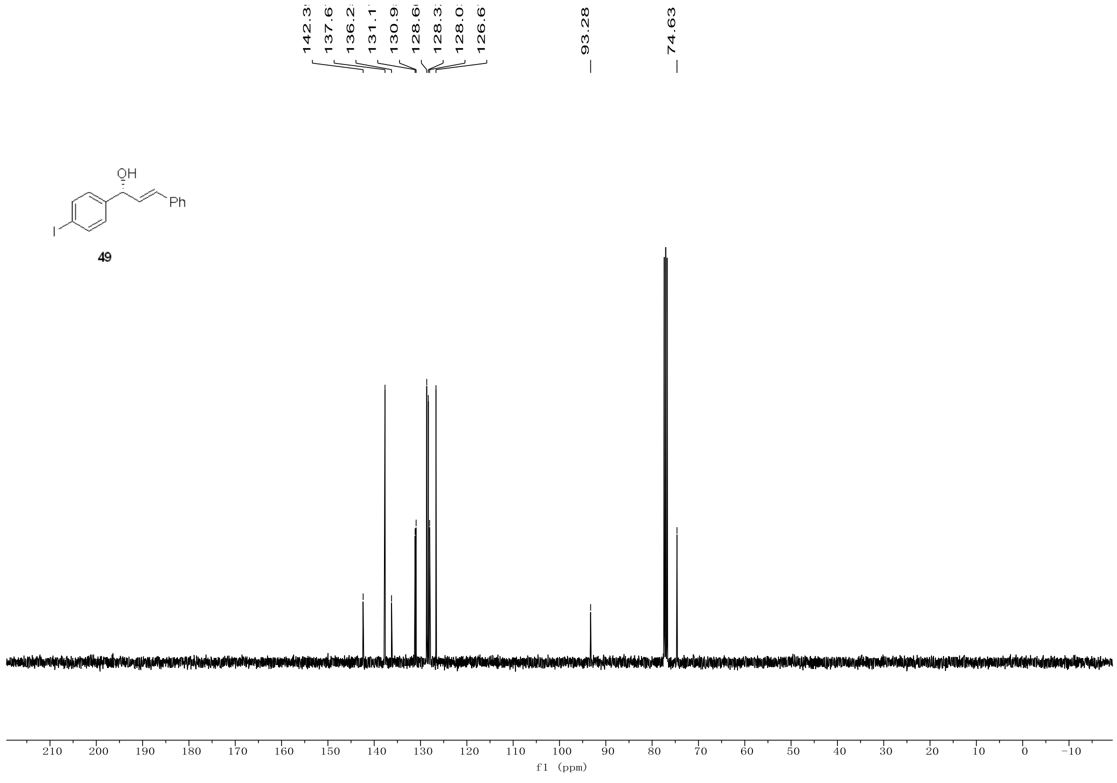

^{1}H NMR of Compound **50** ($CDCl_3$, 400 MHz, 20 °C):

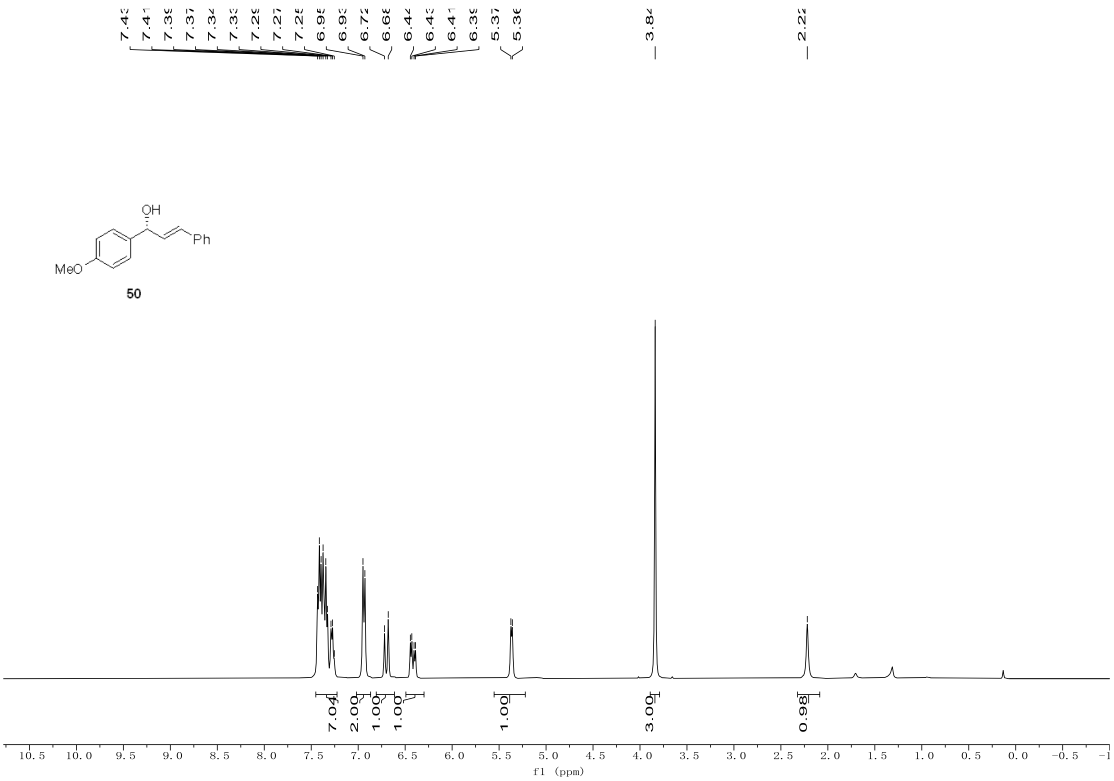

^{13}C NMR of Compound **50** ($CDCl_3$, 101MHz, 20 °C):

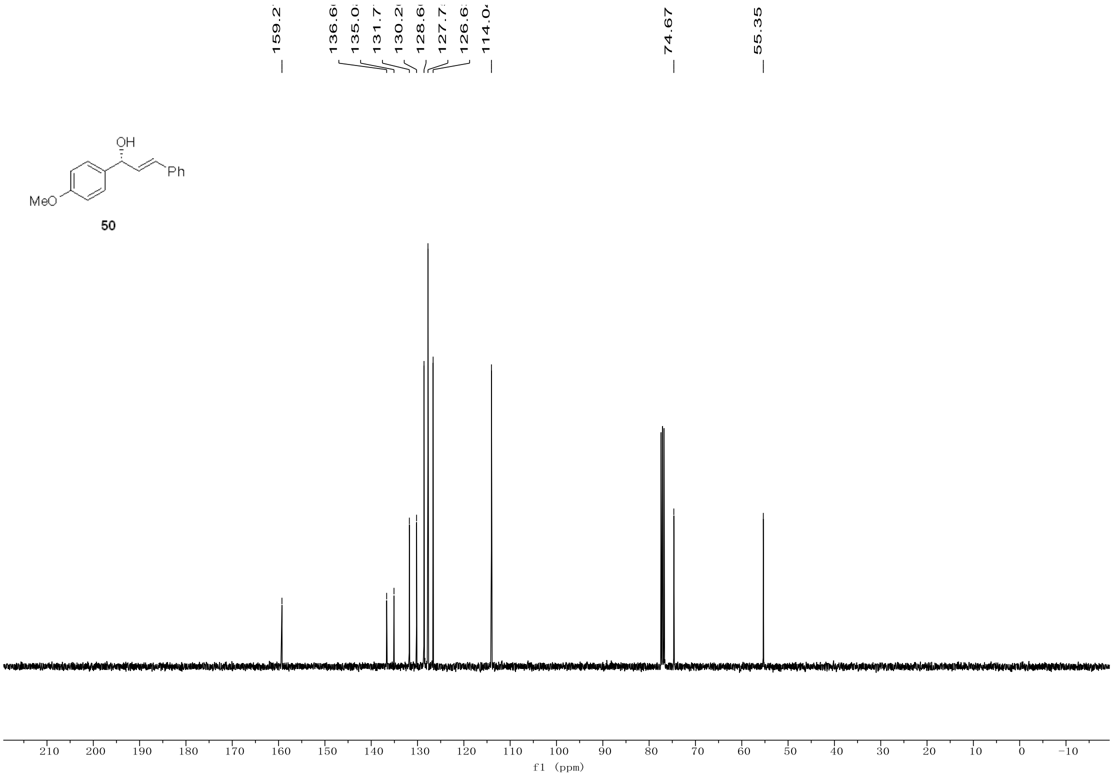

^{1}H NMR of Compound **51** ($CDCl_3$, 400 MHz, 20 °C):

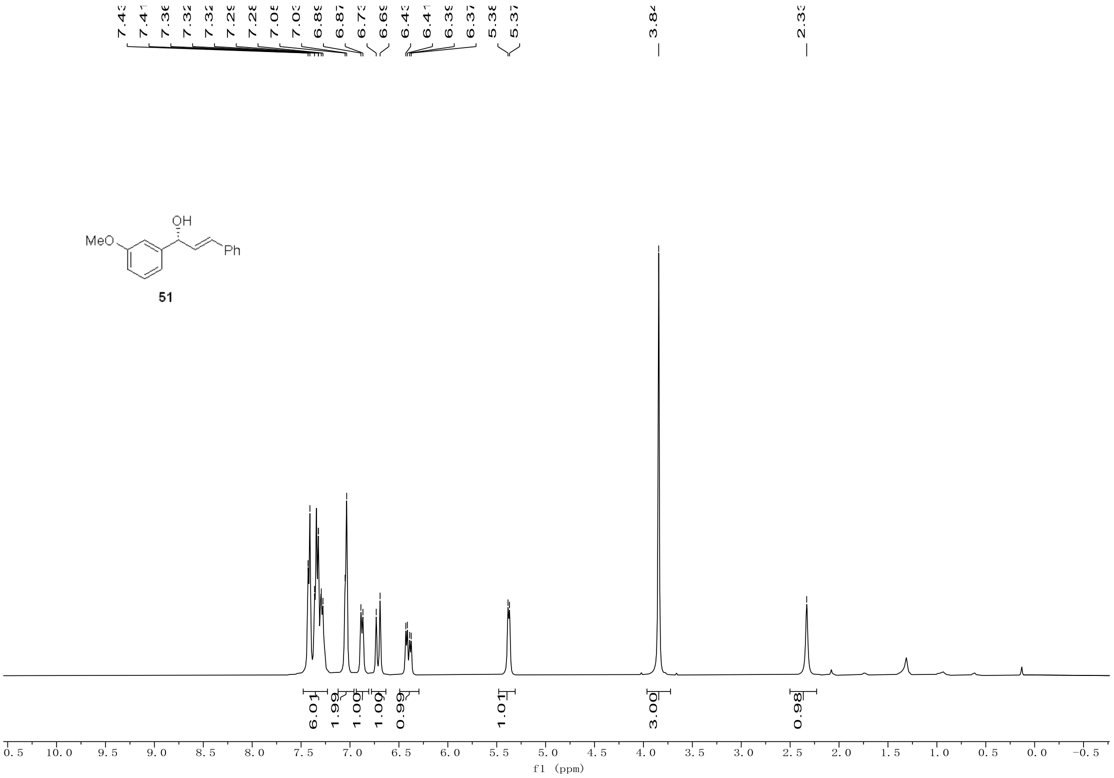

^{13}C NMR of Compound **51** ($CDCl_3$, 101MHz, 20 °C):

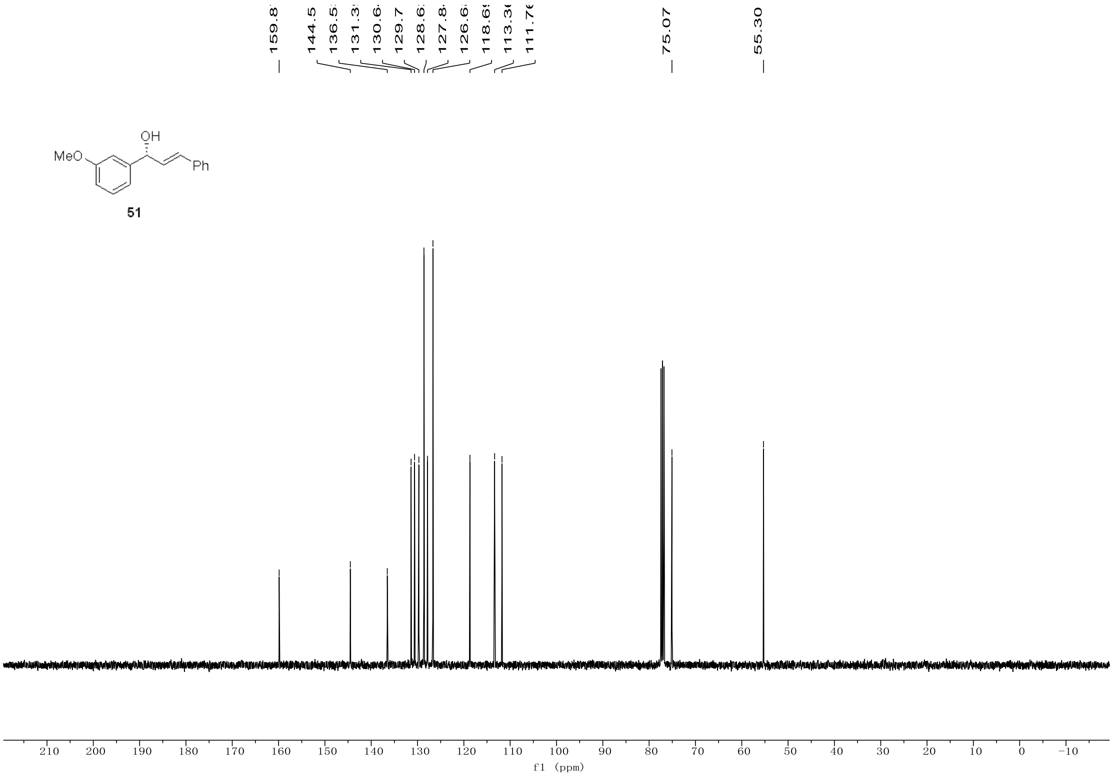

^{1}H NMR of Compound **52** ($CDCl_3$, 400 MHz, 20 °C):

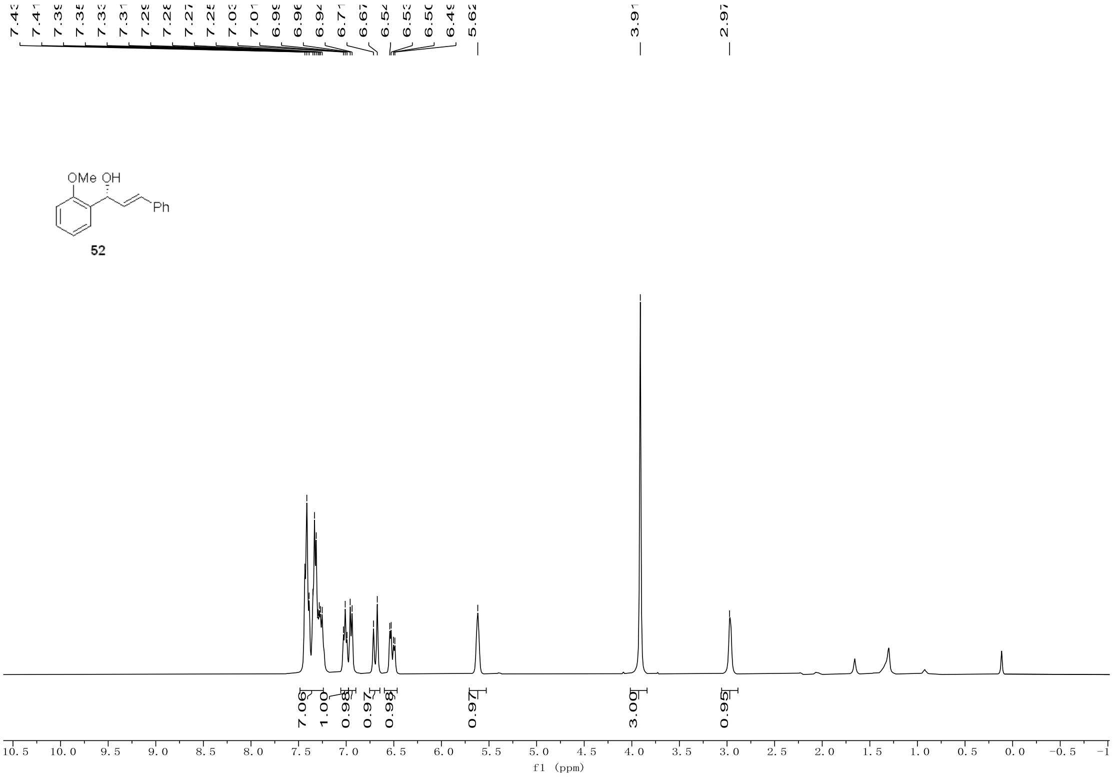

^{13}C NMR of Compound **52** ($CDCl_3$, 101MHz, 20 °C):

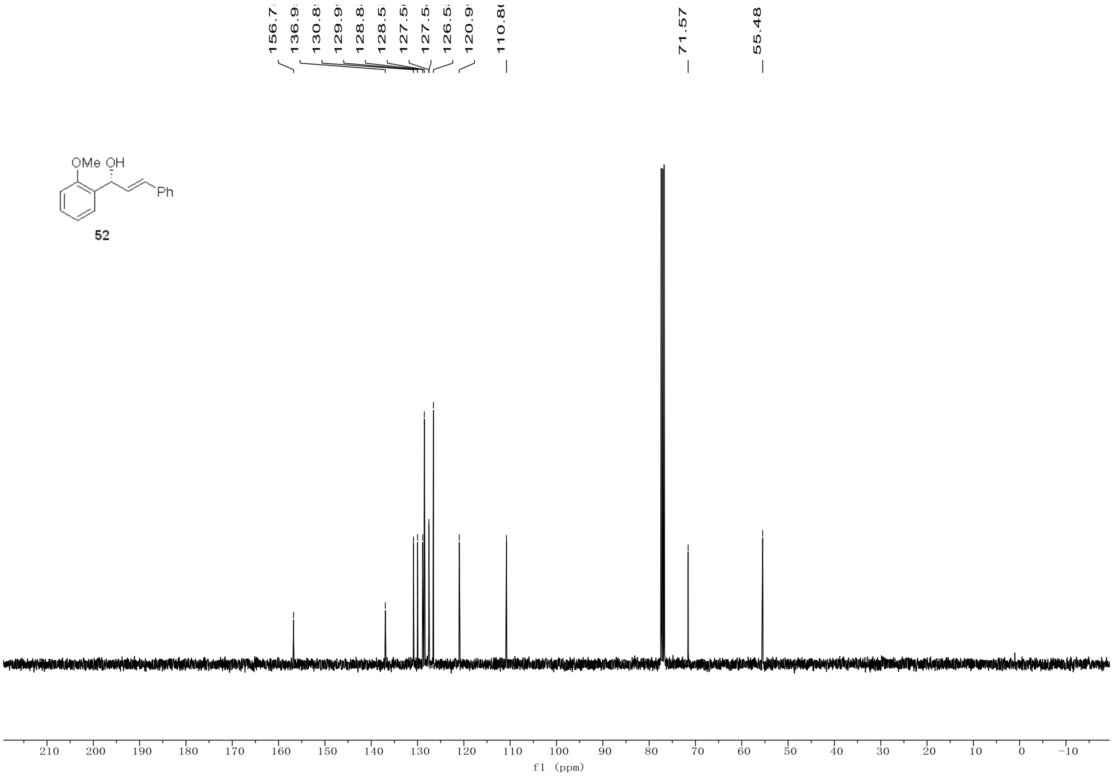

^{1}H NMR of Compound **53** ($CDCl_3$, 400 MHz, 20 °C):

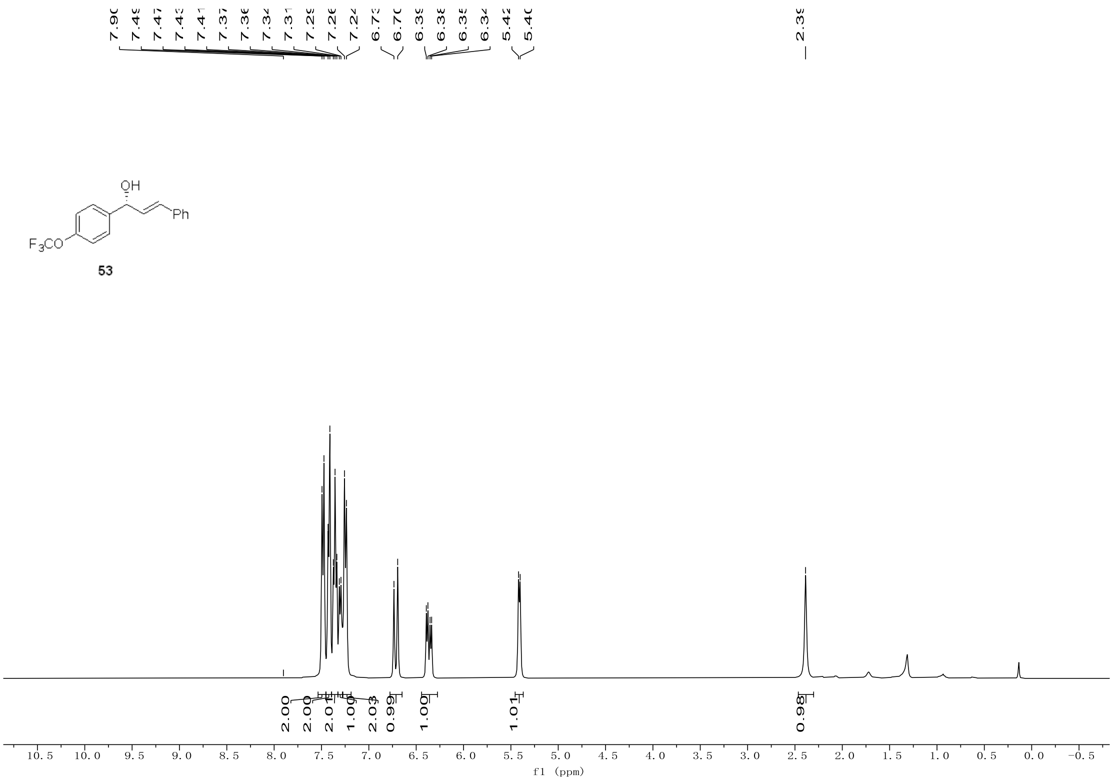

^{13}C NMR of Compound **53** ($CDCl_3$, 101MHz, 20 °C):

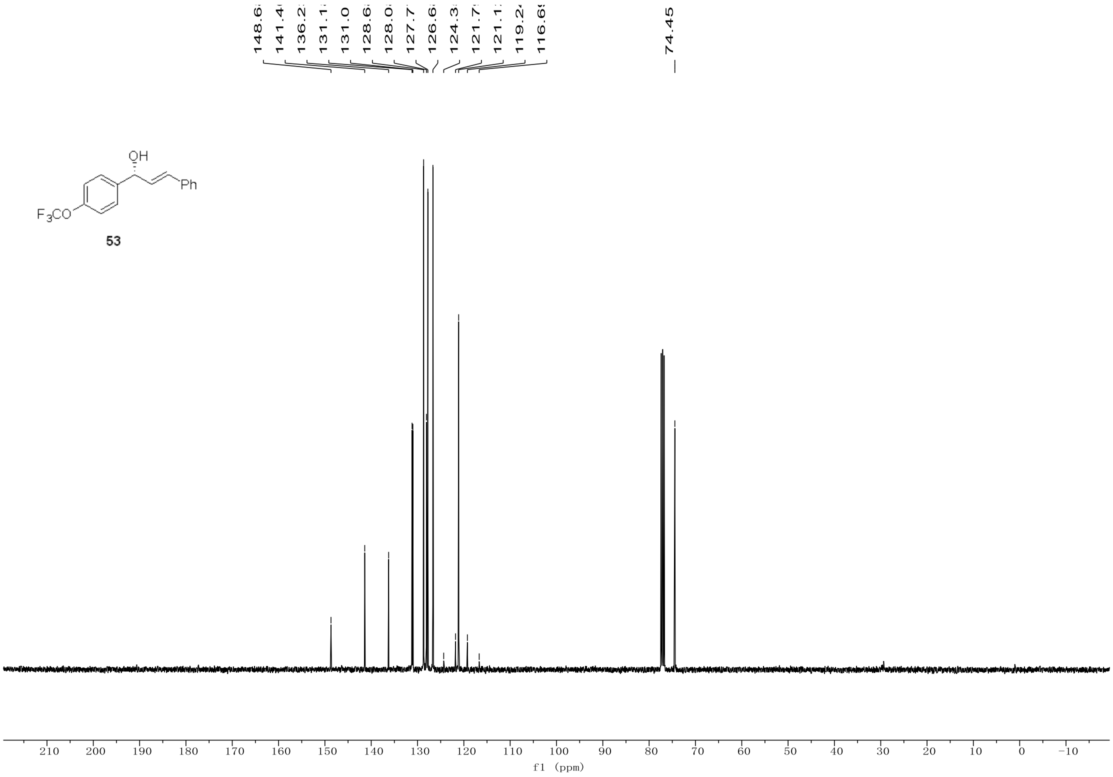

^{19}F NMR of Compound **53** ($CDCl_3$, 376MHz, 20 °C):

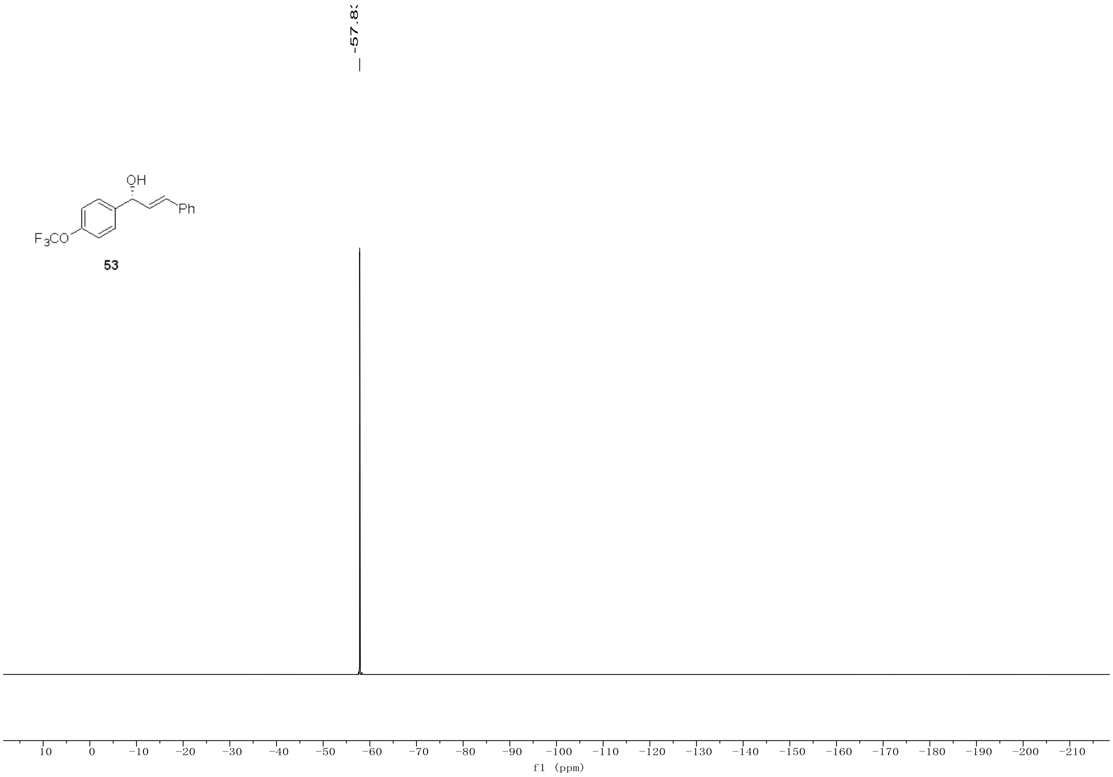

^{1}H NMR of Compound **54** ($CDCl_3$, 400 MHz, 20 °C):

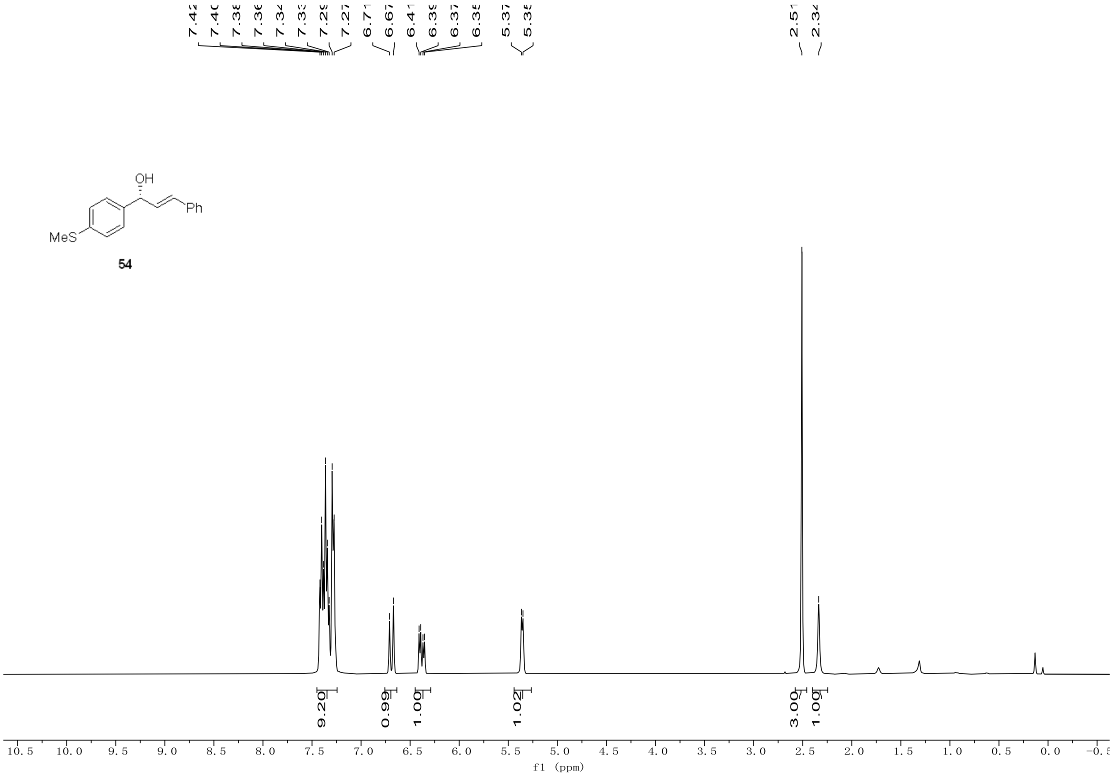

^{13}C NMR of Compound **54** ($CDCl_3$, 101MHz, 20 °C):

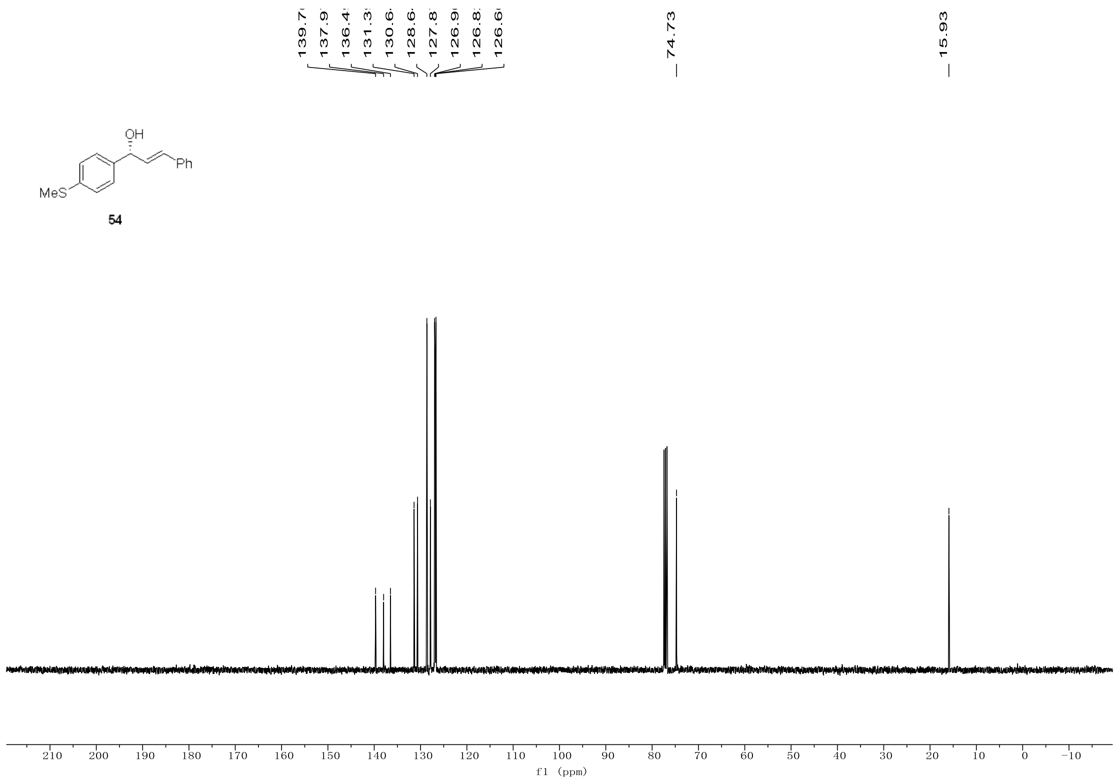

54

^{1}H NMR of Compound **55** ($CDCl_3$, 400 MHz, 20 °C):

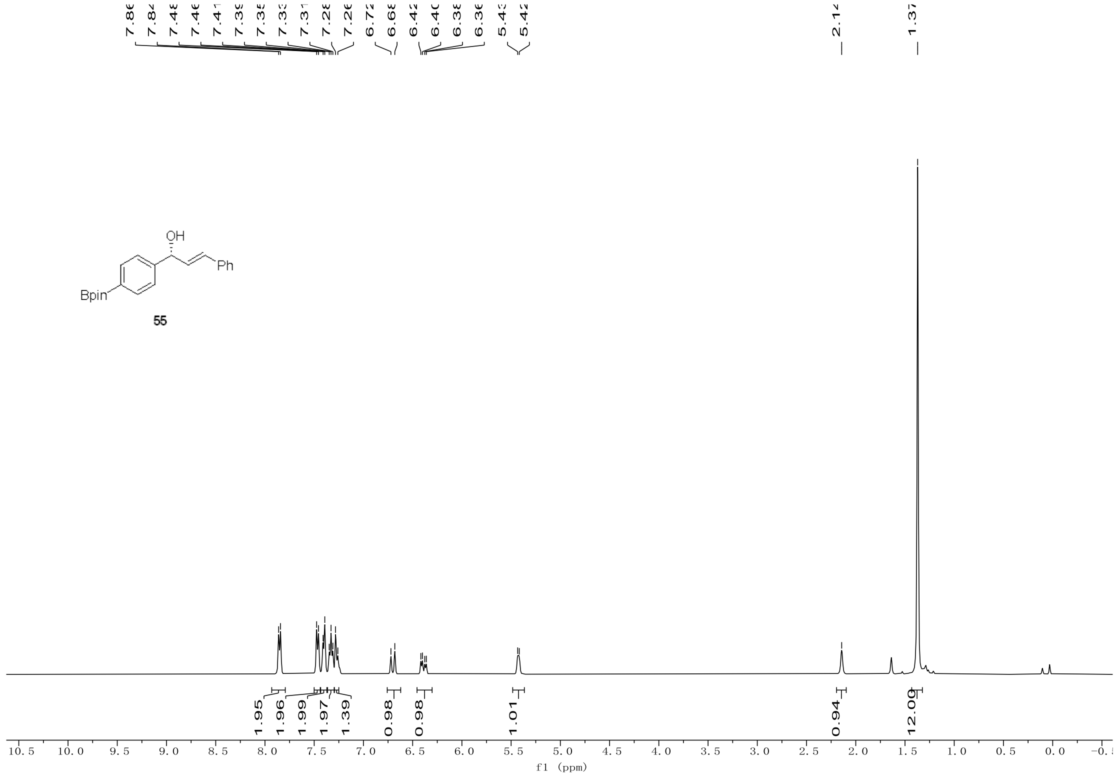

55

^{13}C NMR of Compound **55** ($CDCl_3$, 101MHz, 20 °C):

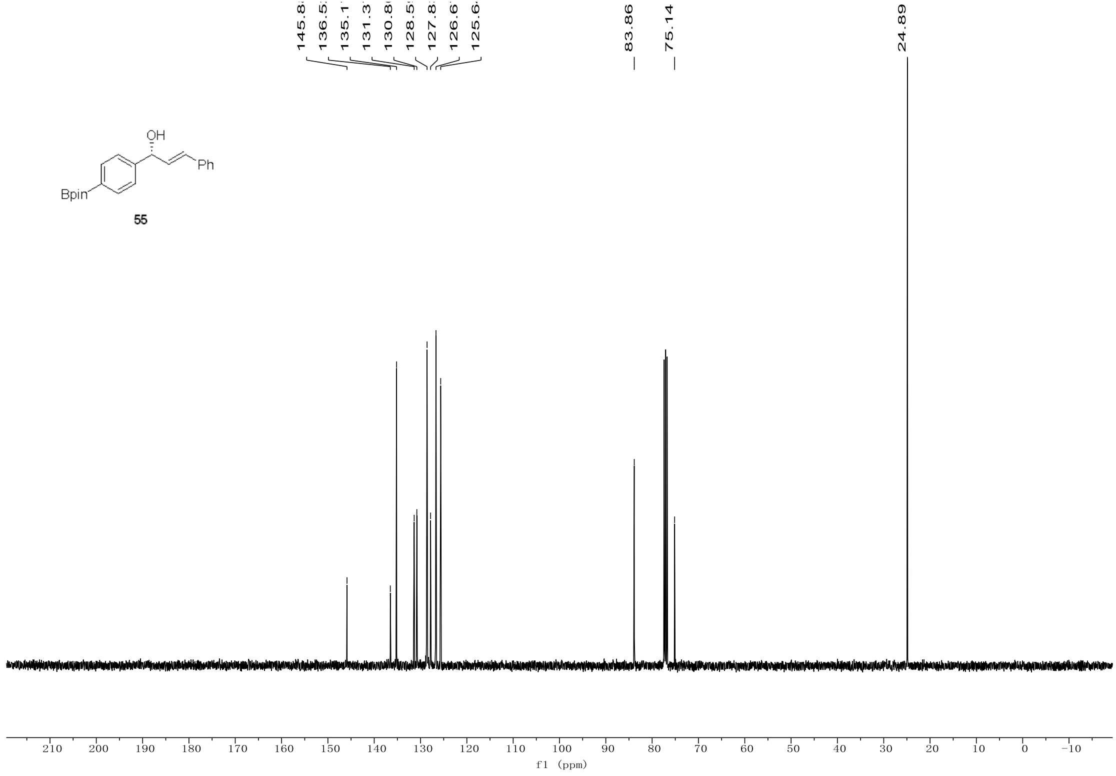

^{1}H NMR of Compound **56** ($CDCl_3$, 400 MHz, 20 °C):

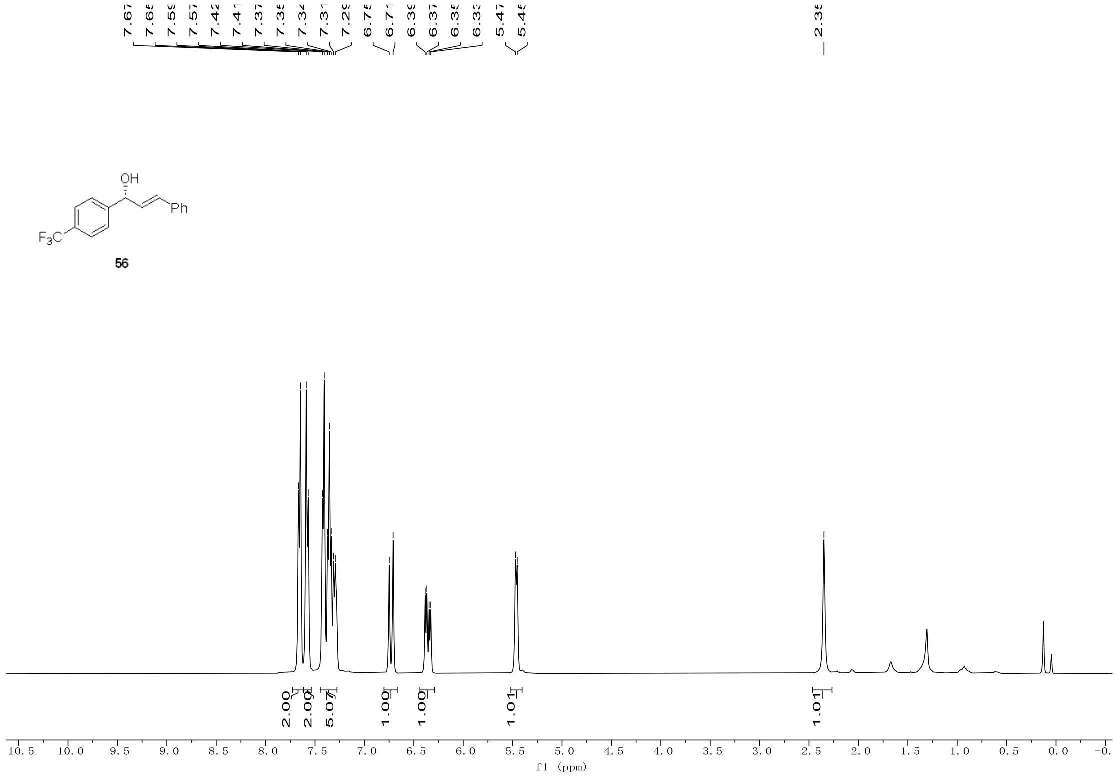

^{13}C NMR of Compound **56** ($CDCl_3$, 101MHz, 20 °C):

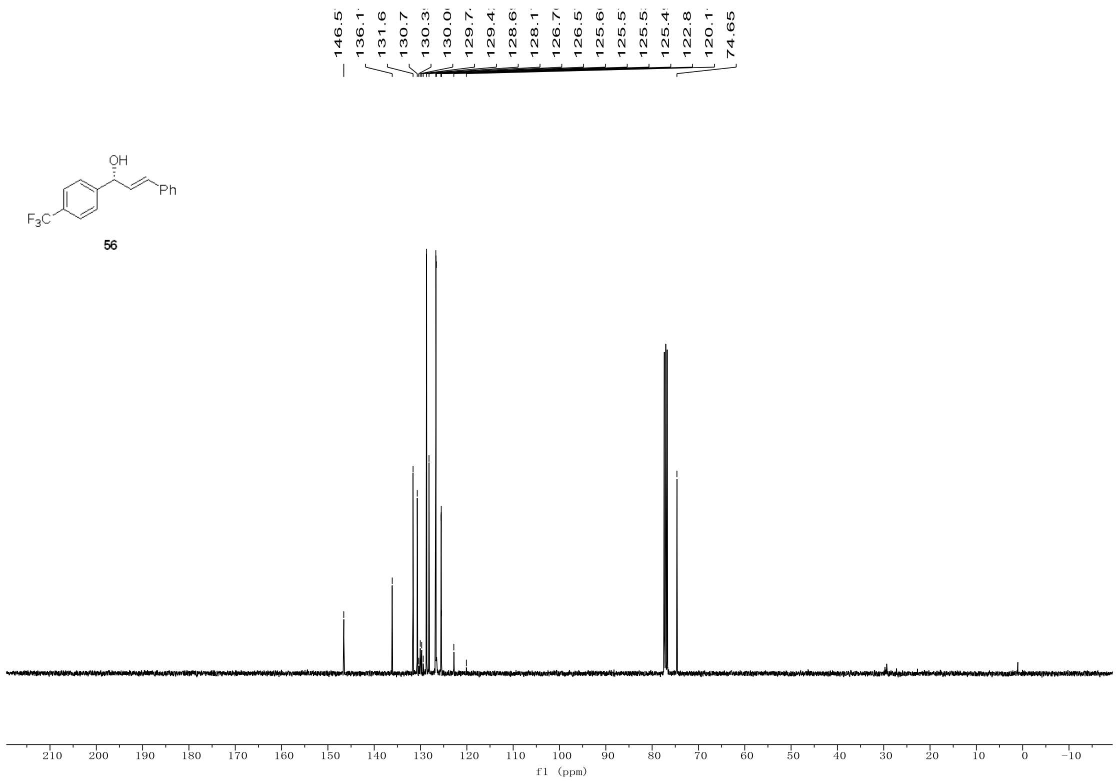

^{19}F NMR of Compound **56** ($CDCl_3$, 376MHz, 20 °C):

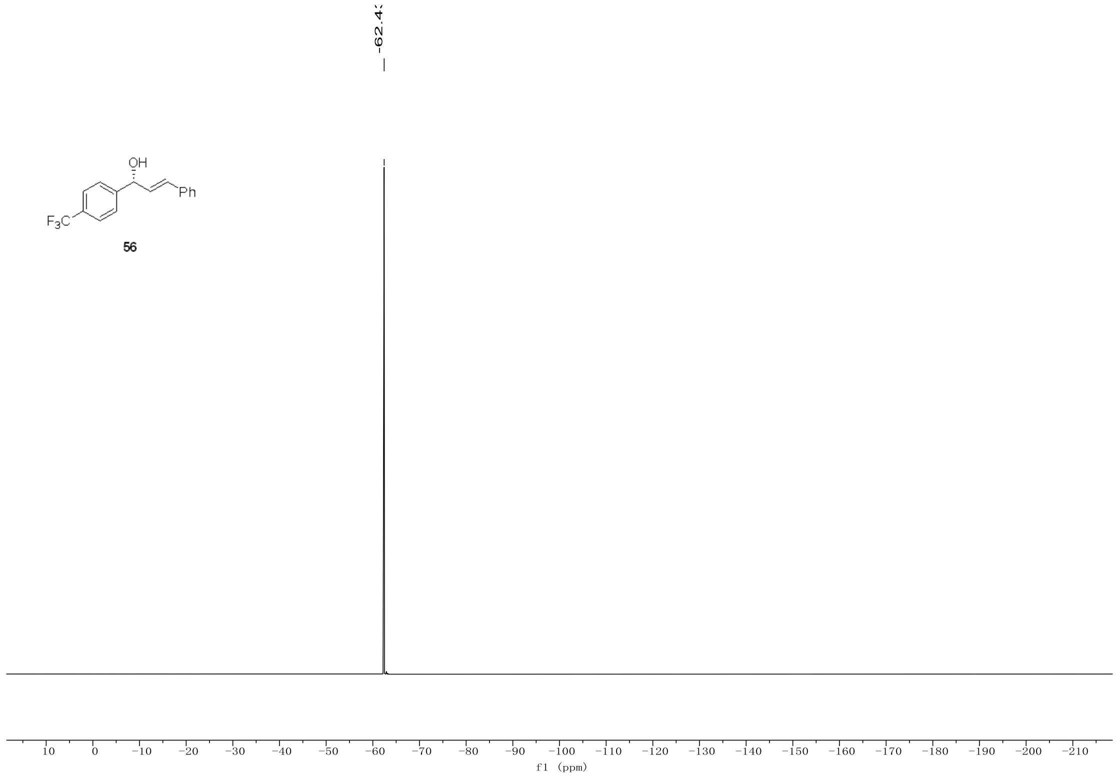

^{1}H NMR of Compound **57** ($CDCl_3$, 400 MHz, 20 °C):

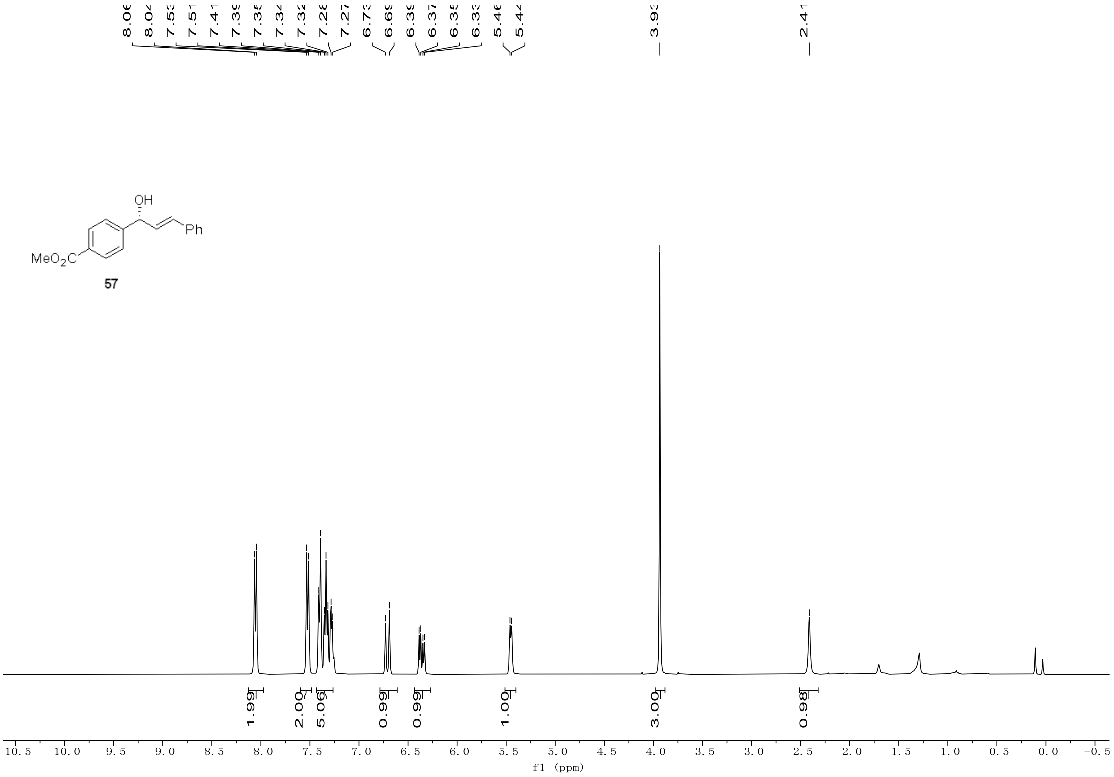

^{13}C NMR of Compound **57** ($CDCl_3$, 101MHz, 20 °C):

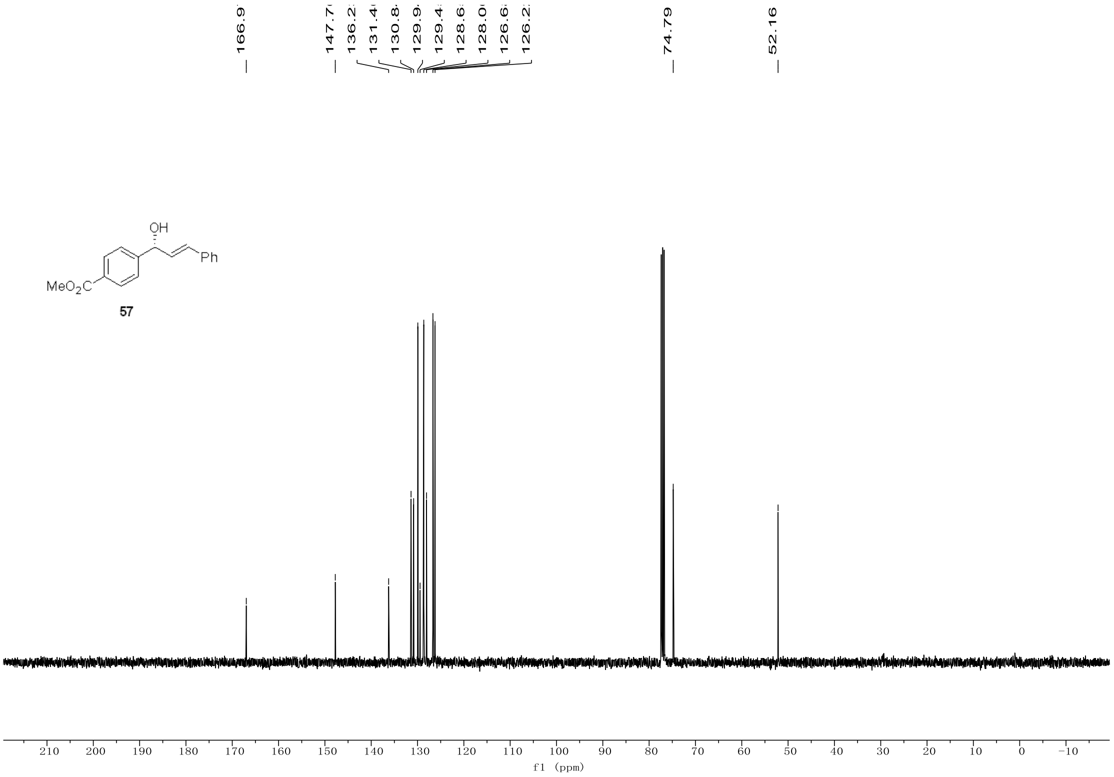

^{1}H NMR of Compound **58** ($CDCl_3$, 400 MHz, 20 °C):

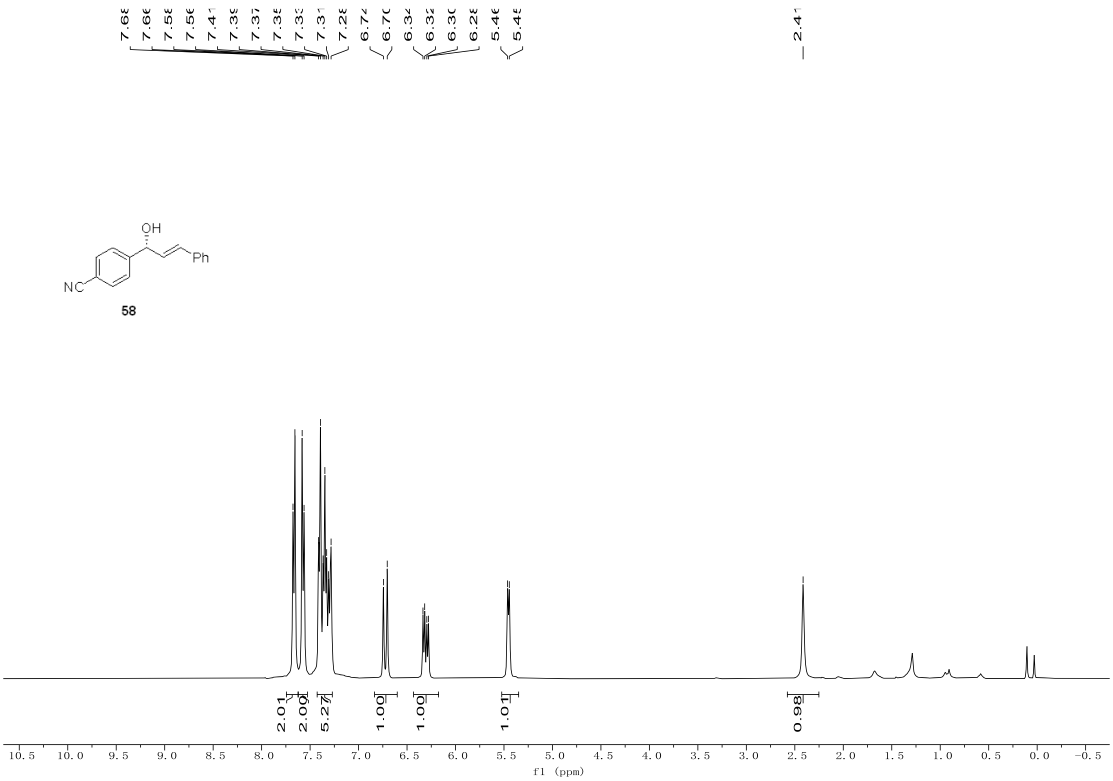

^{13}C NMR of Compound **58** ($CDCl_3$, 101MHz, 20 °C):

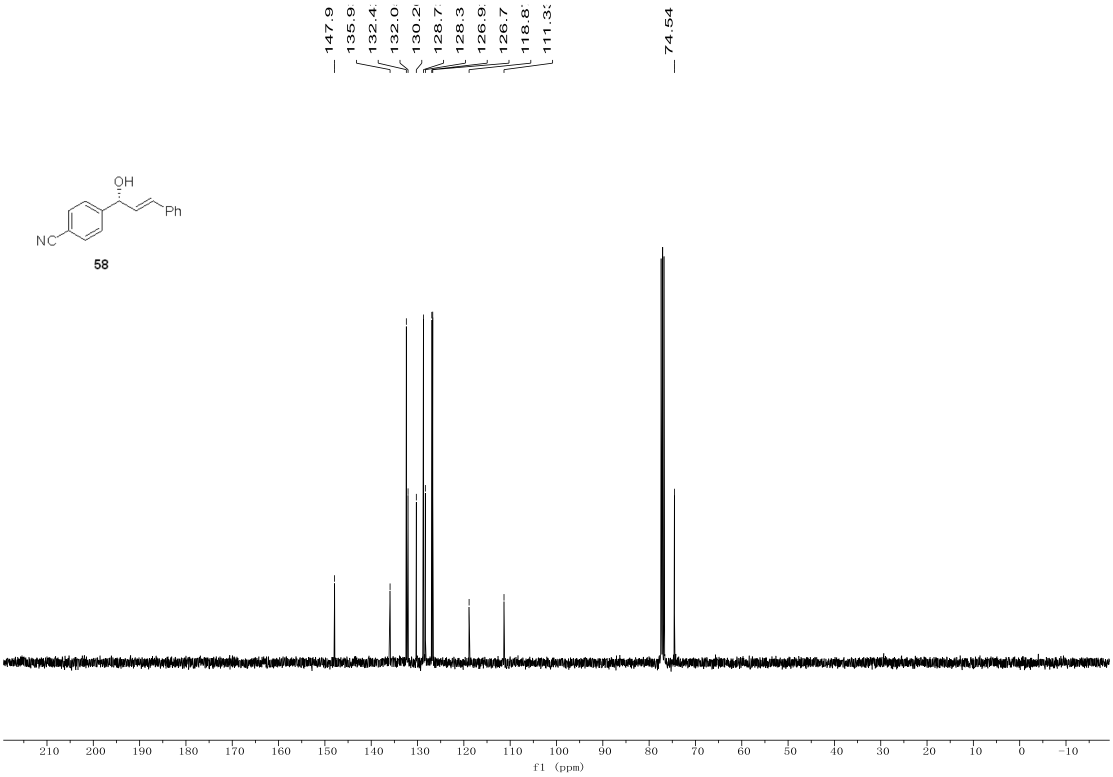

^{1}H NMR of Compound **59** ($CDCl_3$, 400 MHz, 20 °C):

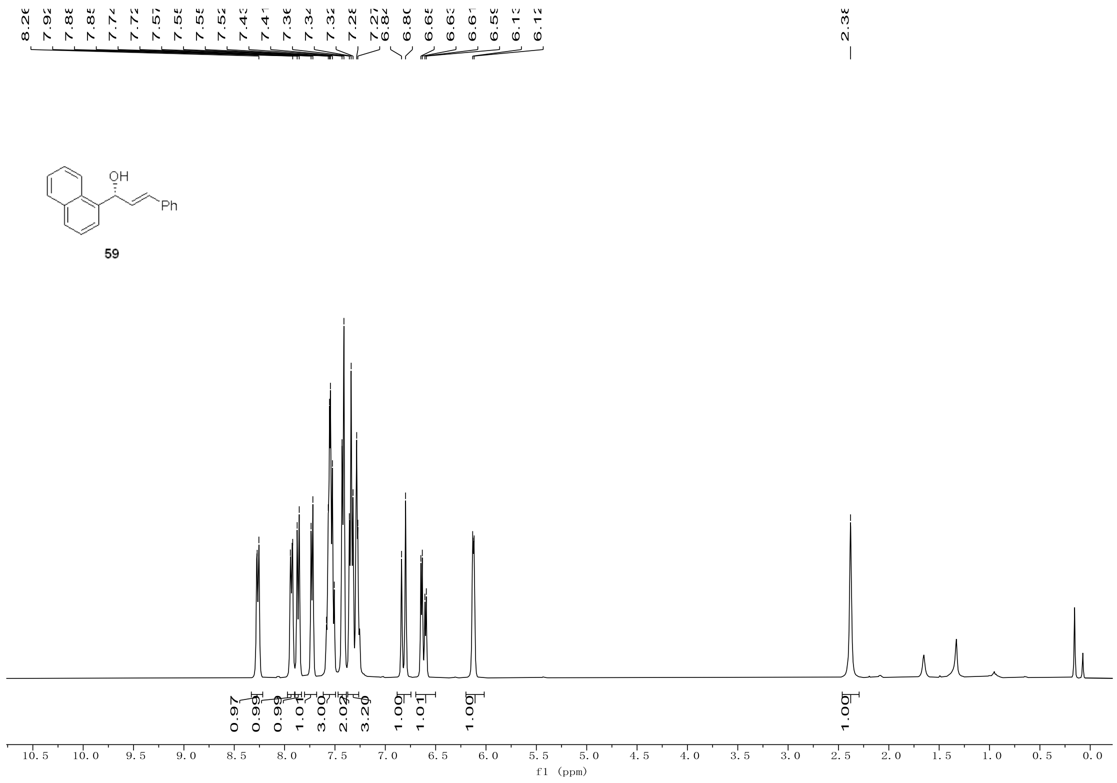

^{13}C NMR of Compound **59** ($CDCl_3$, 101MHz, 20 °C):

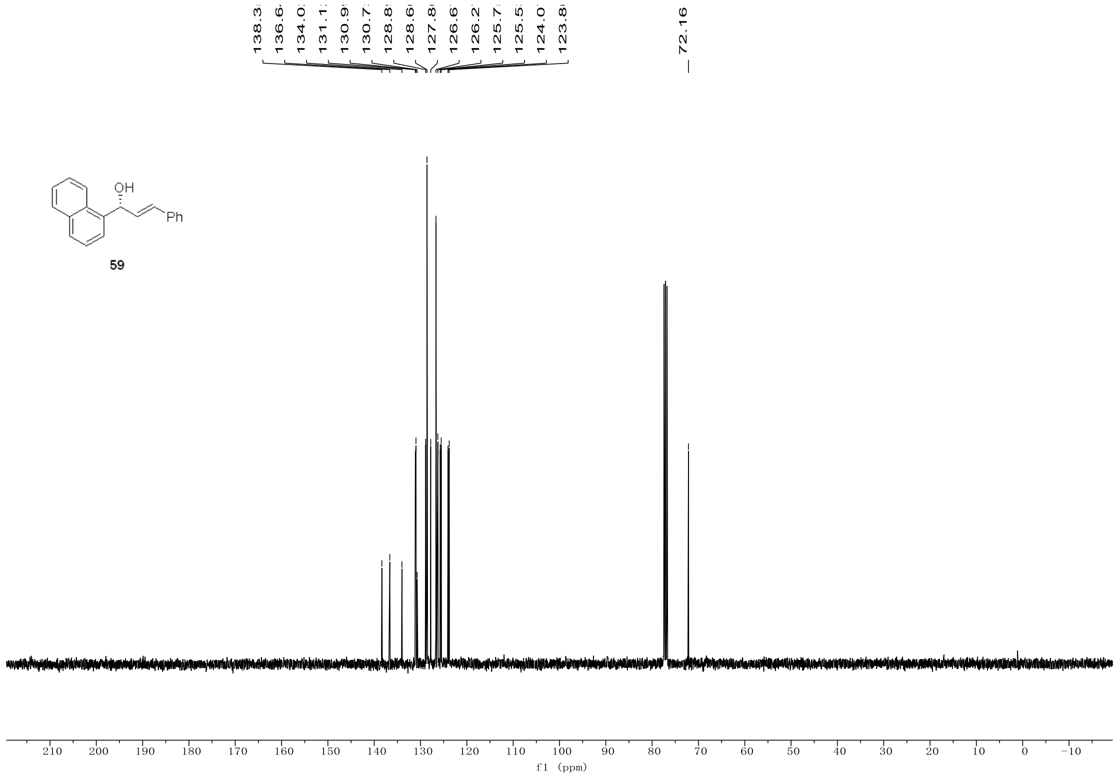

^{1}H NMR of Compound **60** ($CDCl_3$, 400 MHz, 20 °C):

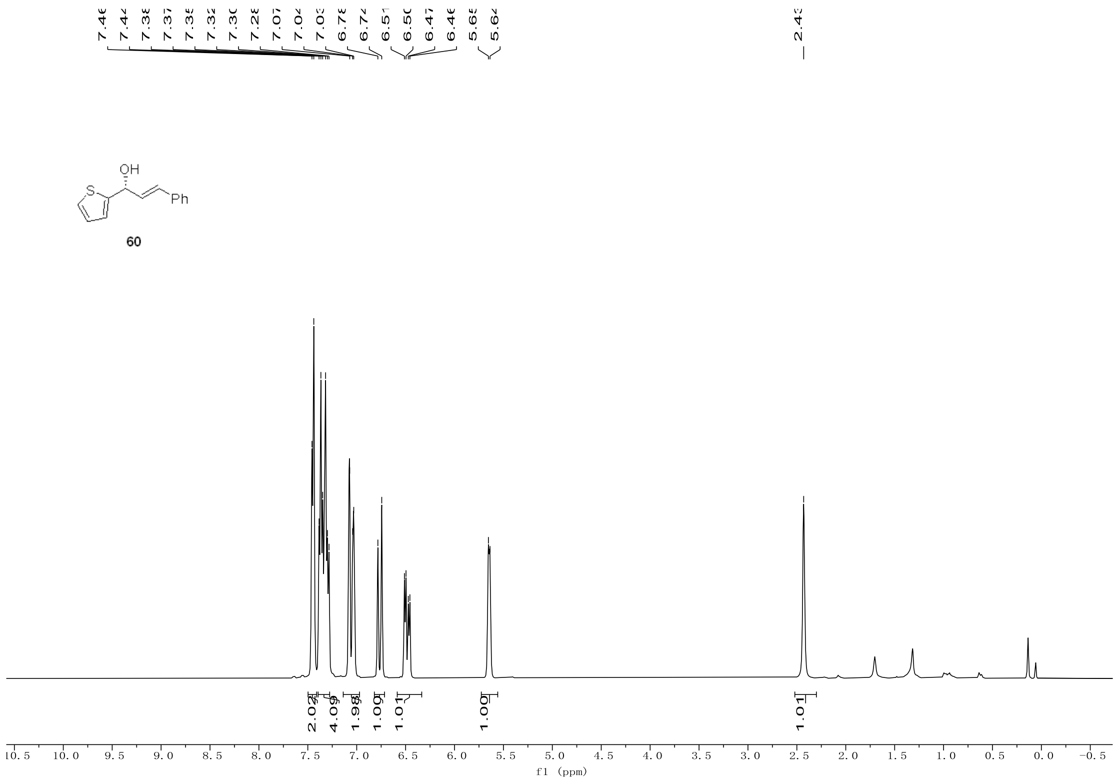

^{13}C NMR of Compound **60** ($CDCl_3$, 101MHz, 20 °C):

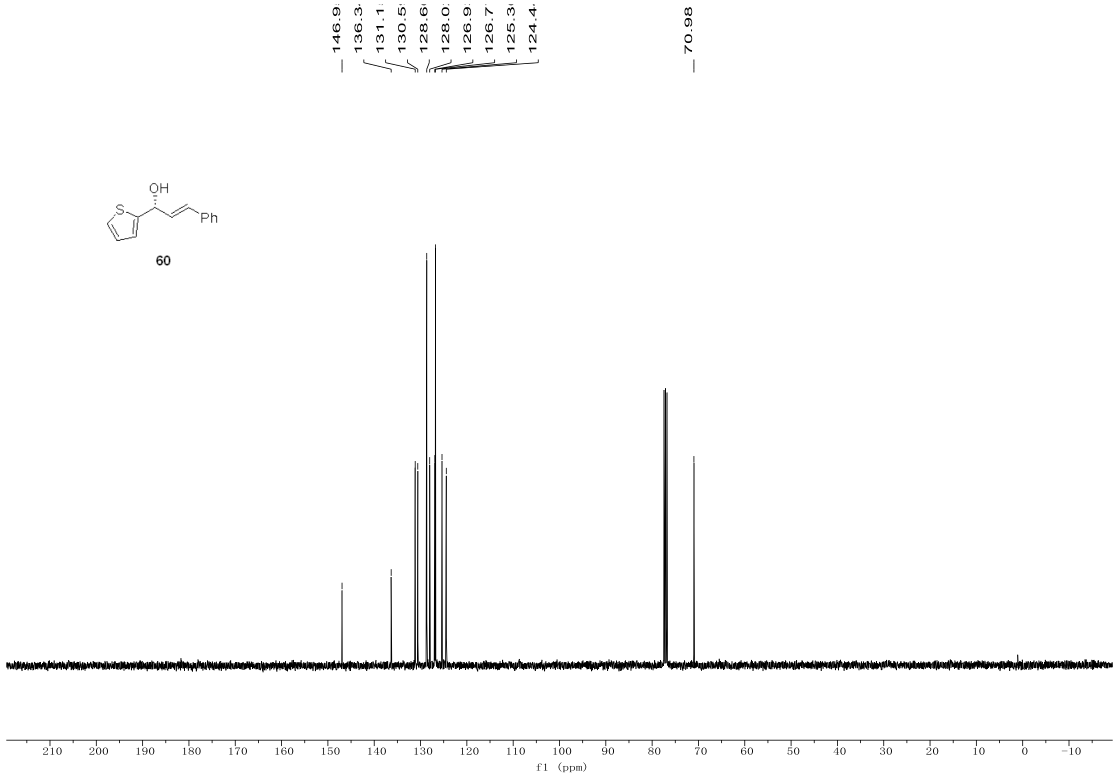

^{1}H NMR of Compound **61** ($CDCl_3$, 400 MHz, 20 °C):

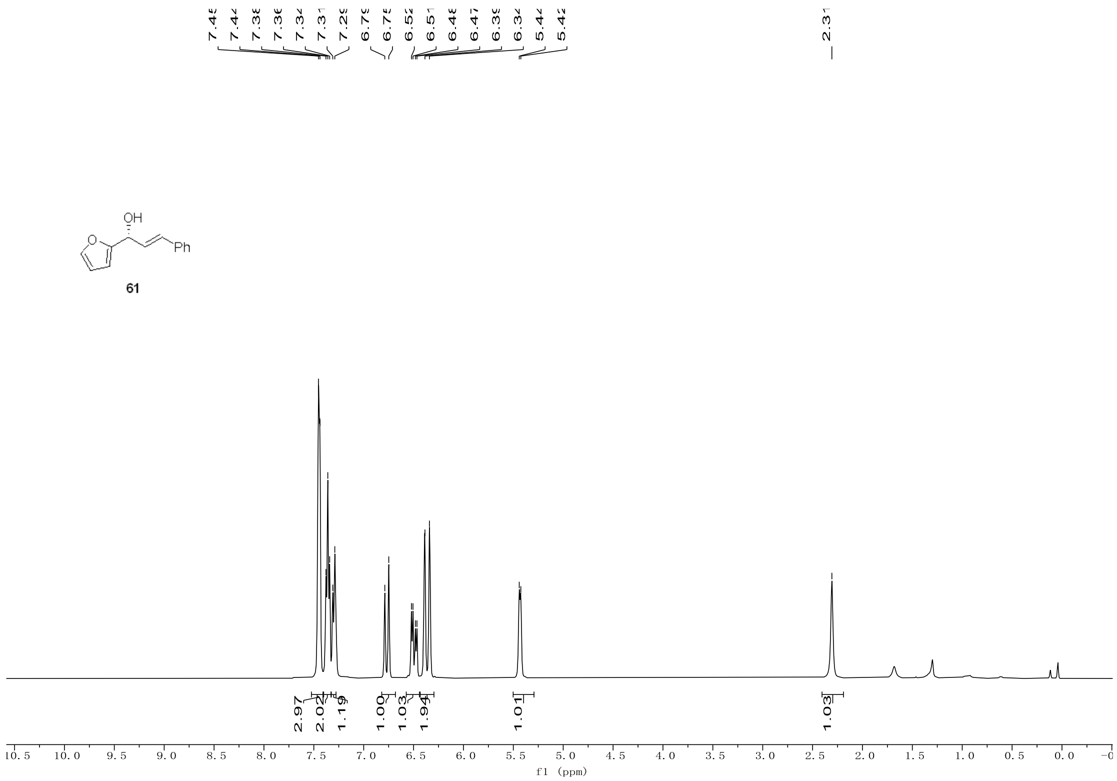

^{13}C NMR of Compound **61** ($CDCl_3$, 101MHz, 20 °C):

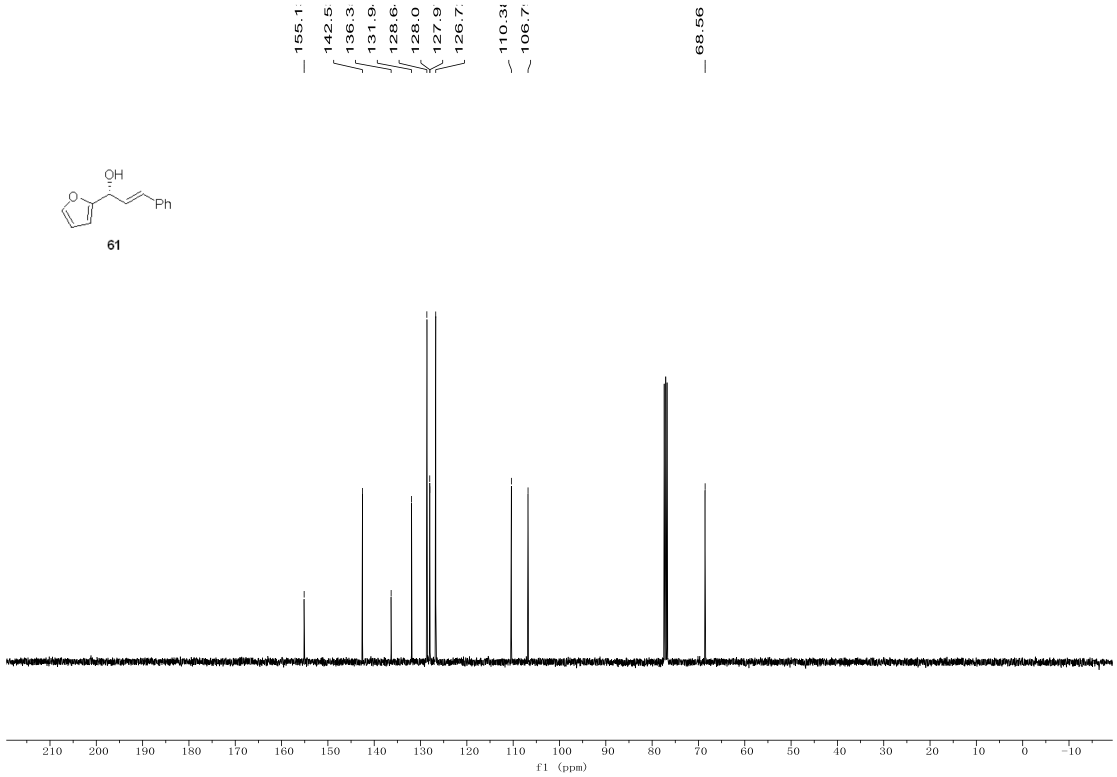

^{1}H NMR of Compound **62** ($CDCl_3$, 400 MHz, 20 °C):

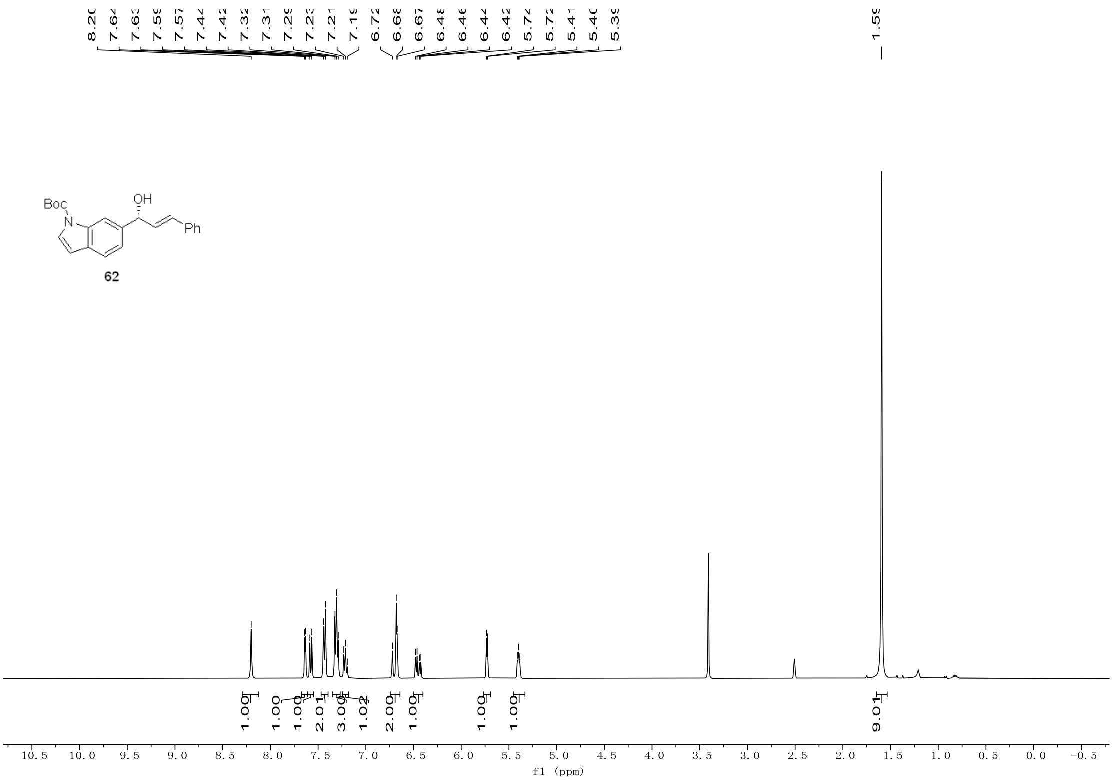

^{13}C NMR of Compound **62** ($CDCl_3$, 101MHz, 20 °C):

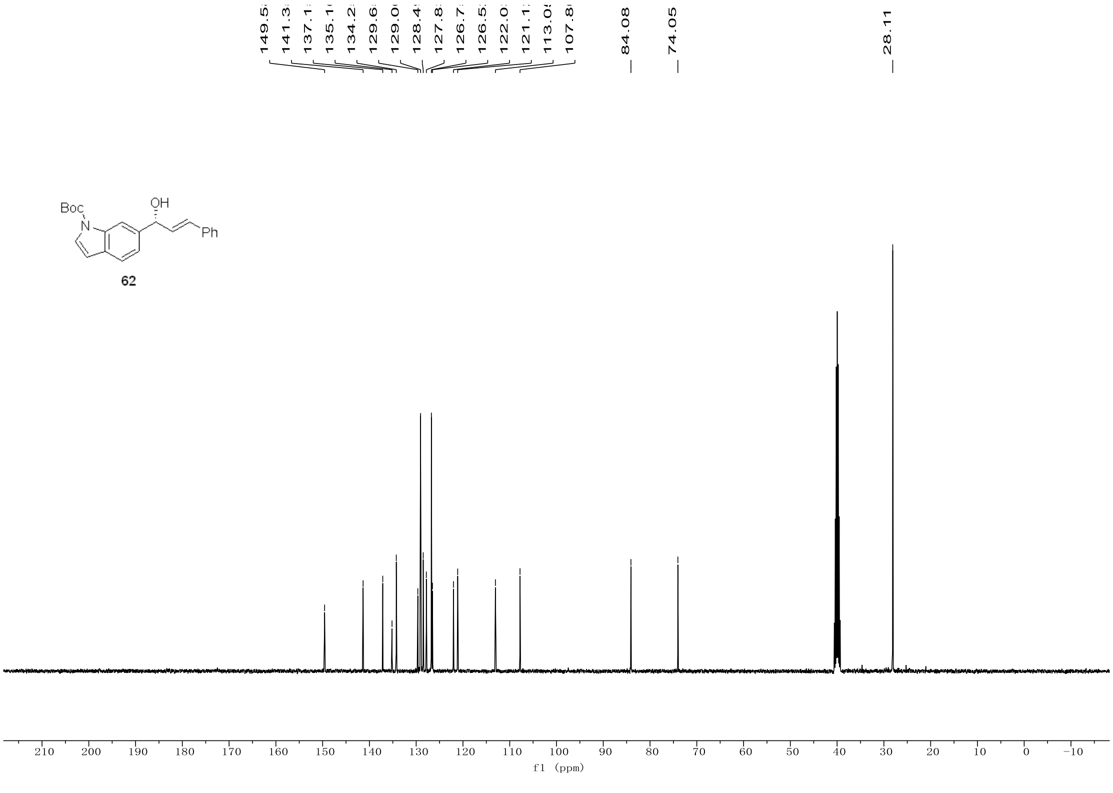

^{1}H NMR of Compound **63** ($CDCl_3$, 400 MHz, 20 °C):

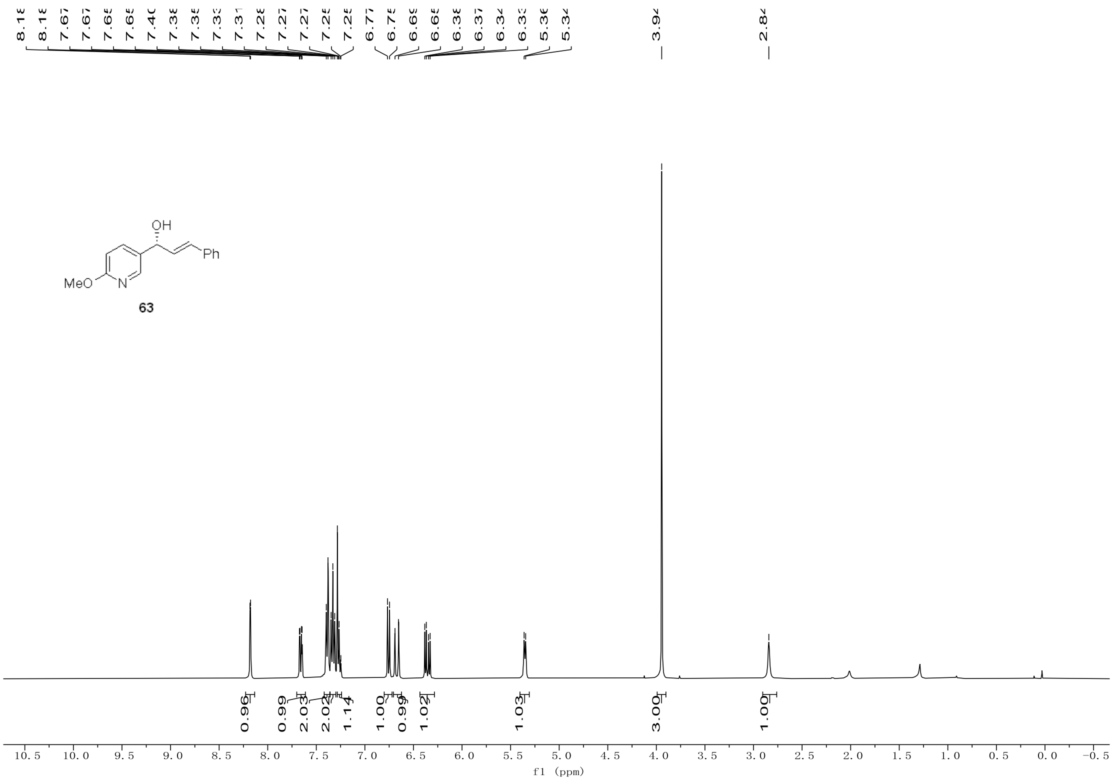

^{13}C NMR of Compound **63** ($CDCl_3$, 101MHz, 20 °C):

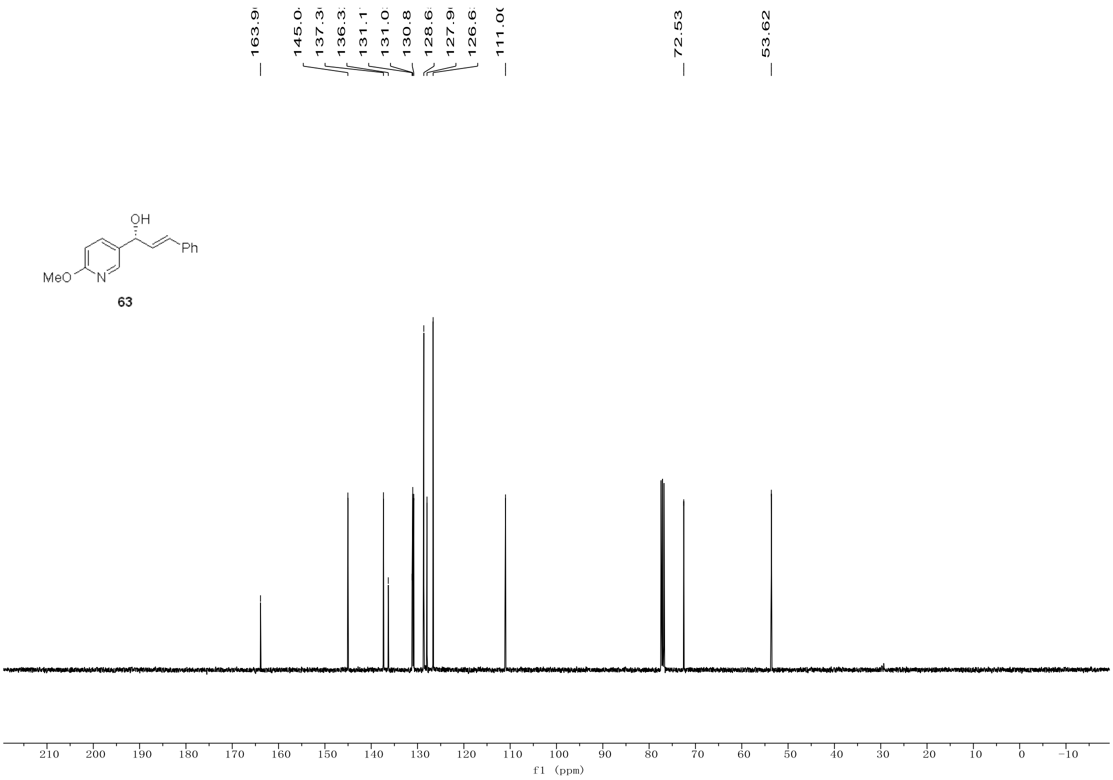

^{1}H NMR of Compound **64** ($CDCl_3$, 400 MHz, 20 °C):

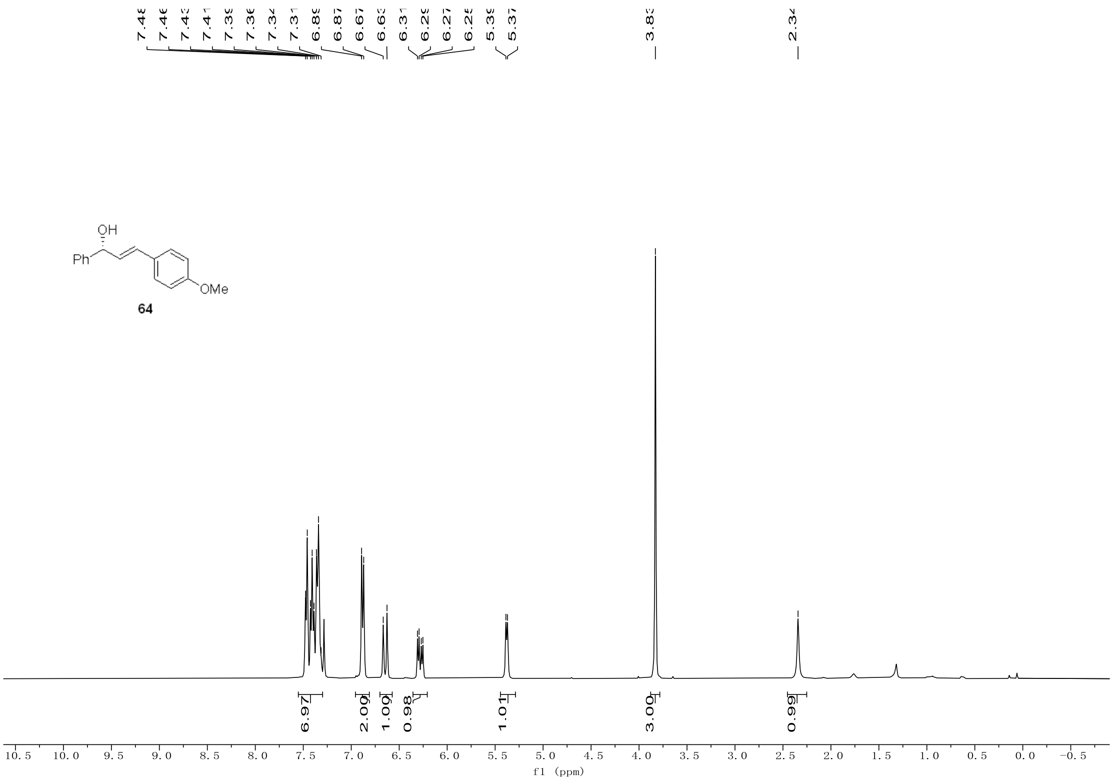

^{13}C NMR of Compound **64** ($CDCl_3$, 101MHz, 20 °C):

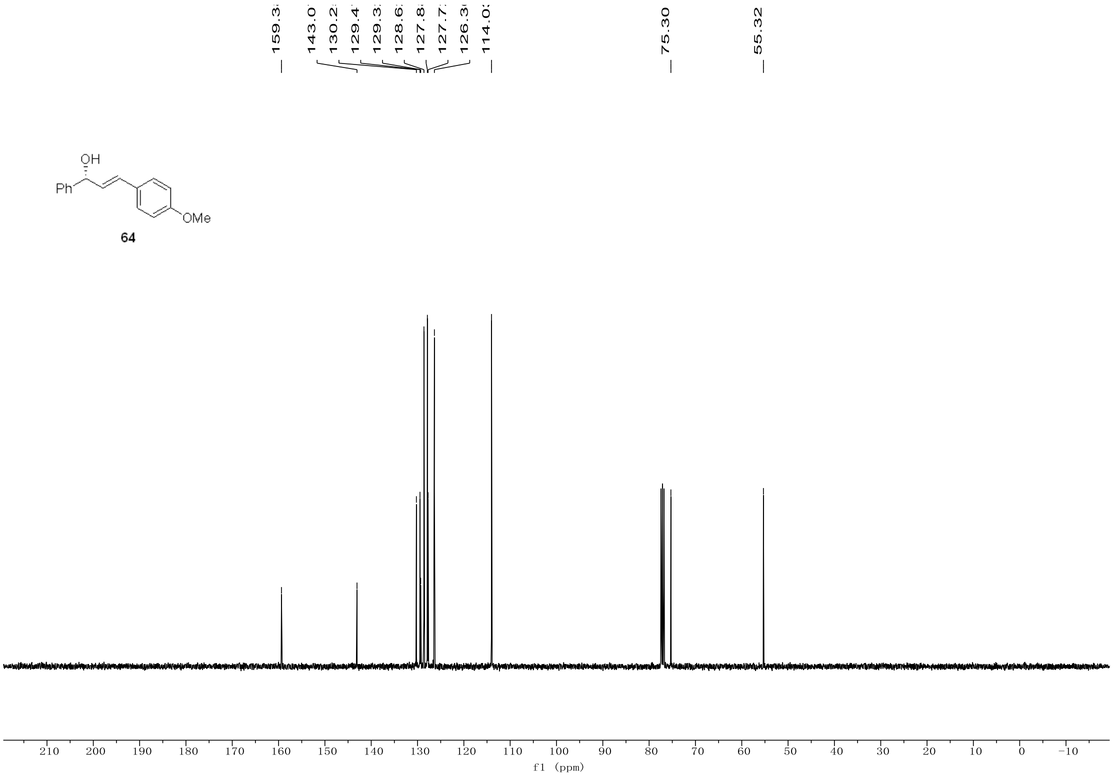

^{1}H NMR of Compound **65** ($CDCl_3$, 400 MHz, 20 °C):

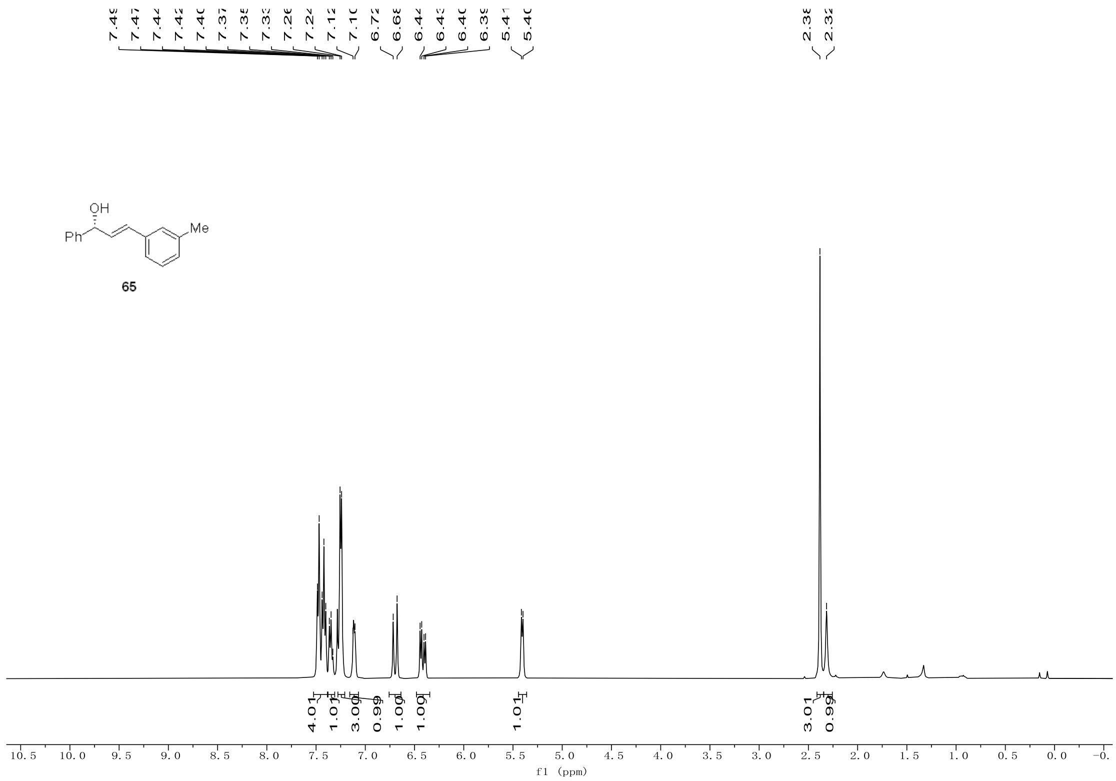

^{13}C NMR of Compound **65** ($CDCl_3$, 101MHz, 20 °C):

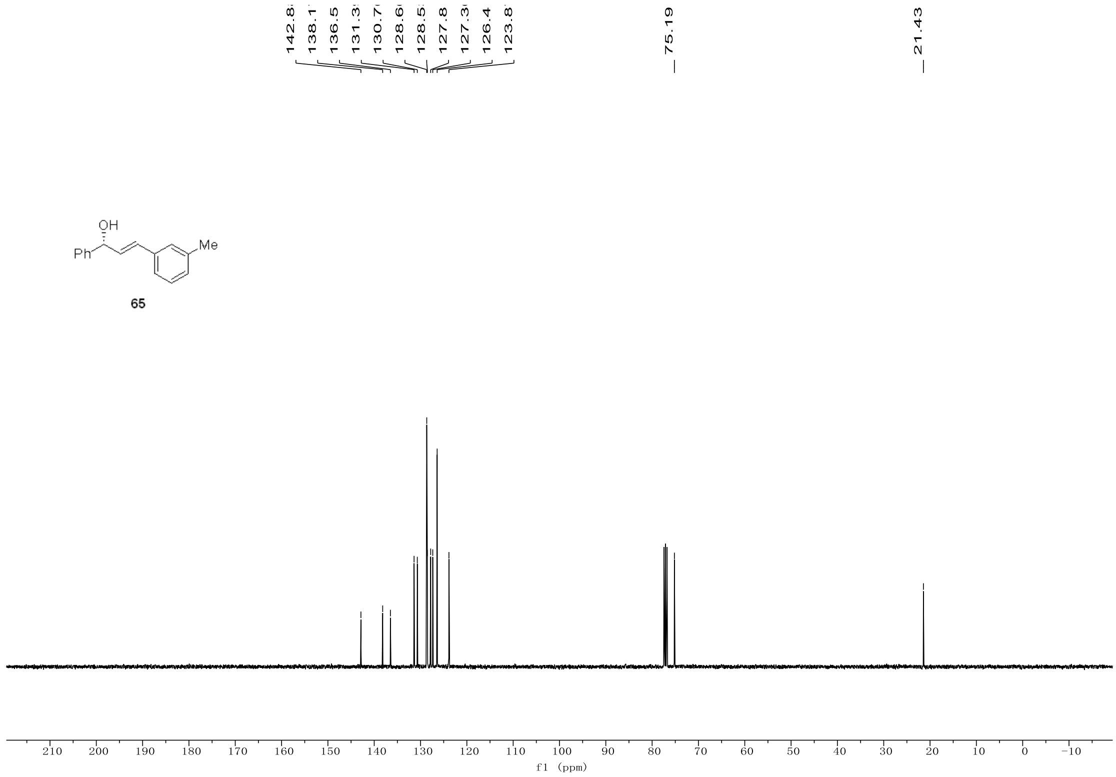

^{1}H NMR of Compound **66** ($CDCl_3$, 400 MHz, 20 °C):

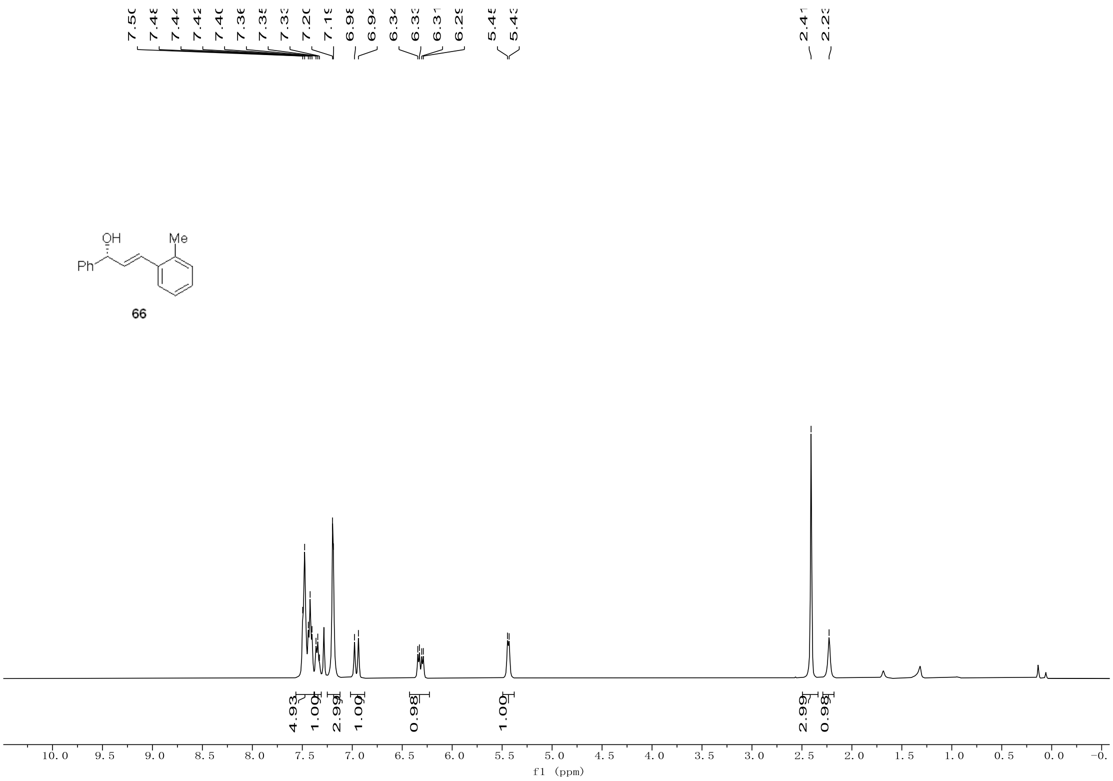

^{13}C NMR of Compound **66** ($CDCl_3$, 101MHz, 20 °C):

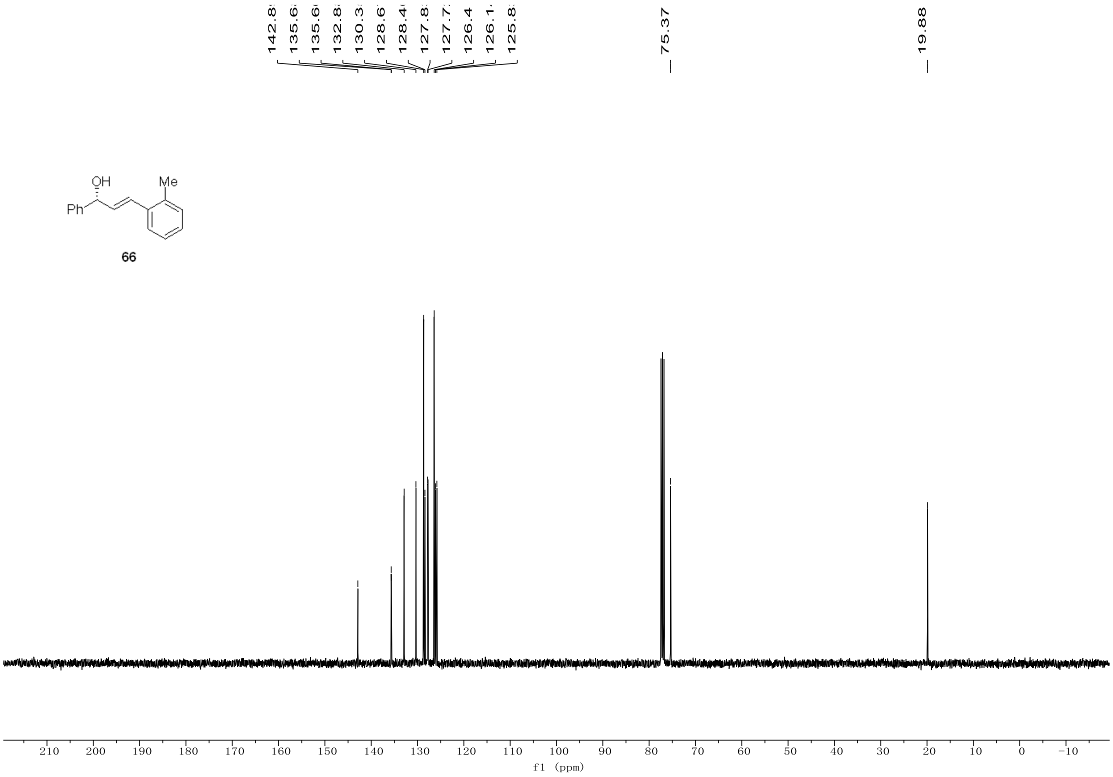

^{1}H NMR of Compound **67** ($CDCl_3$, 400 MHz, 20 °C):

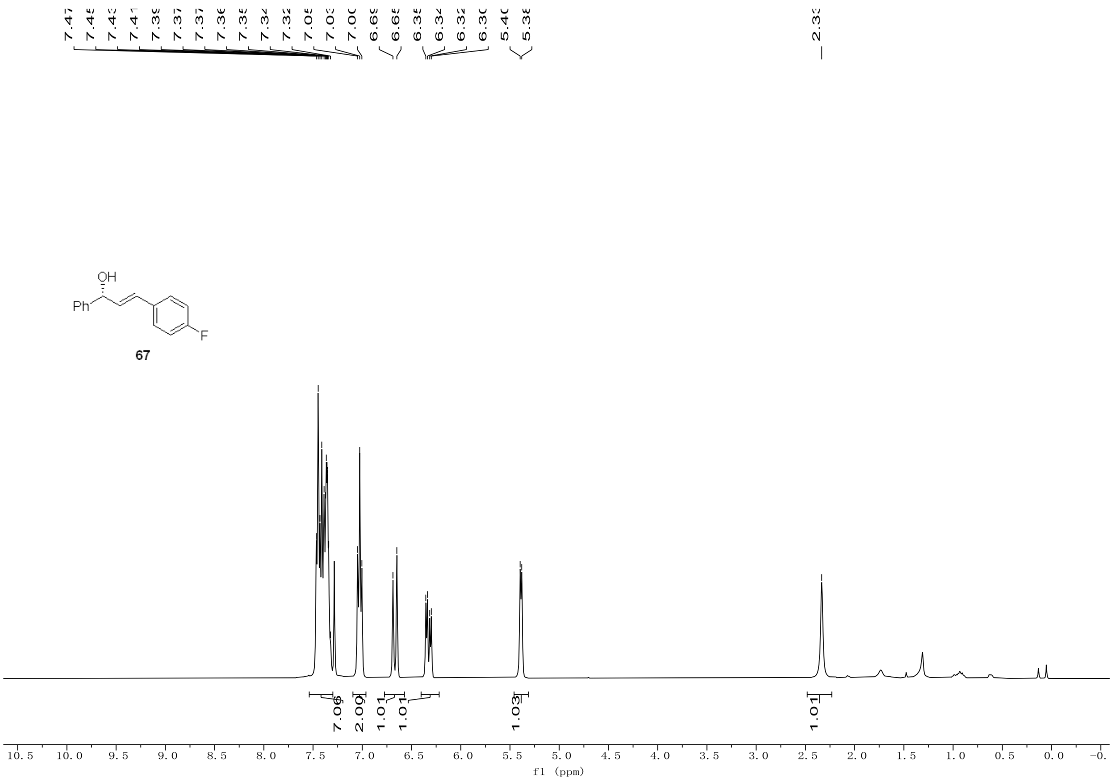

^{13}C NMR of Compound **67** ($CDCl_3$, 101MHz, 20 °C):

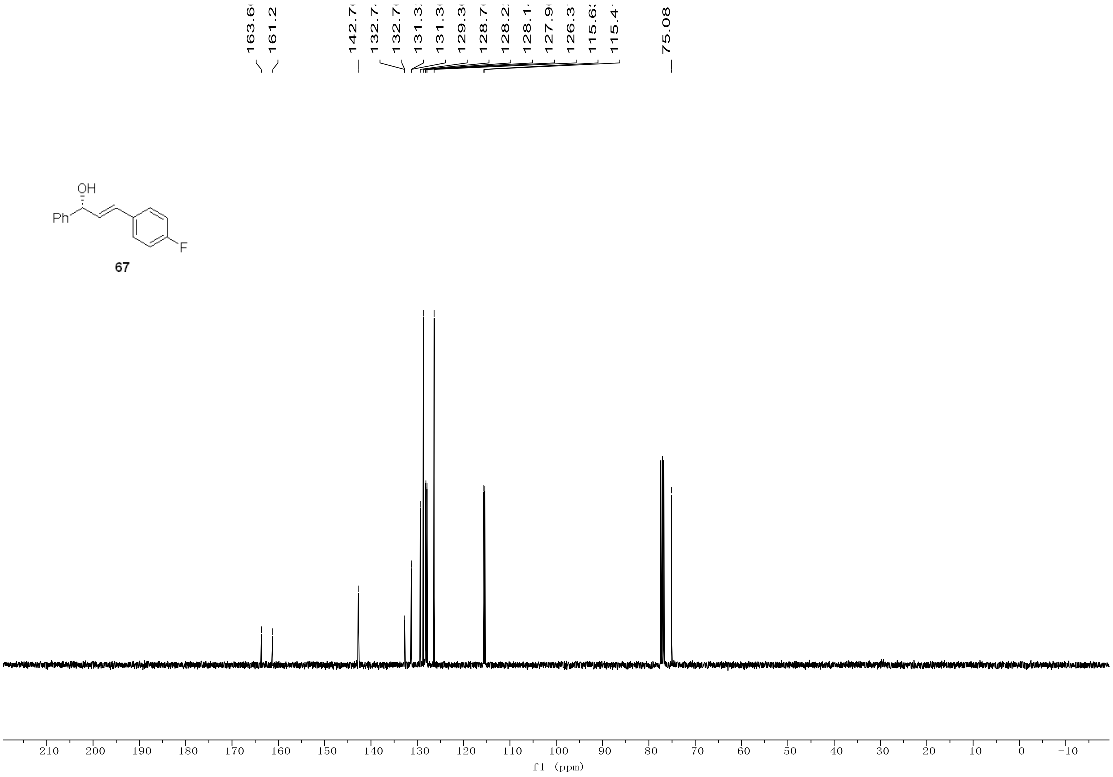

^{19}F NMR of Compound **67** ($CDCl_3$, 376MHz, 20 °C):

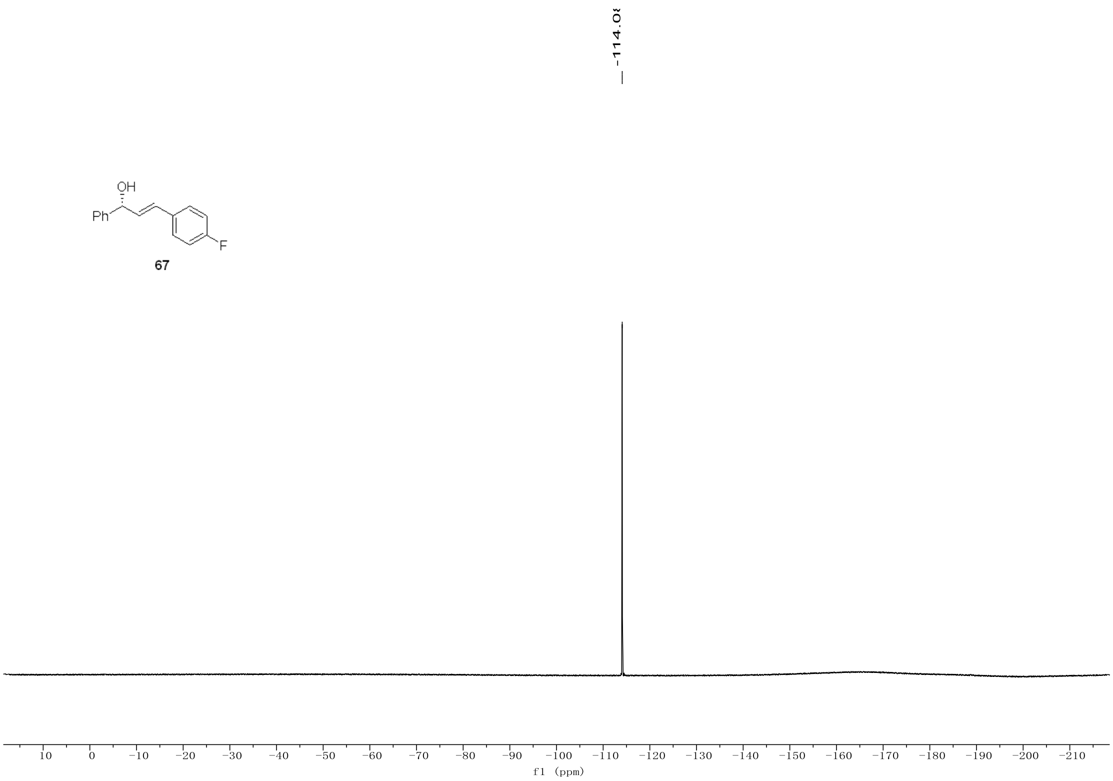

^{1}H NMR of Compound **68** ($CDCl_3$, 400 MHz, 20 °C):

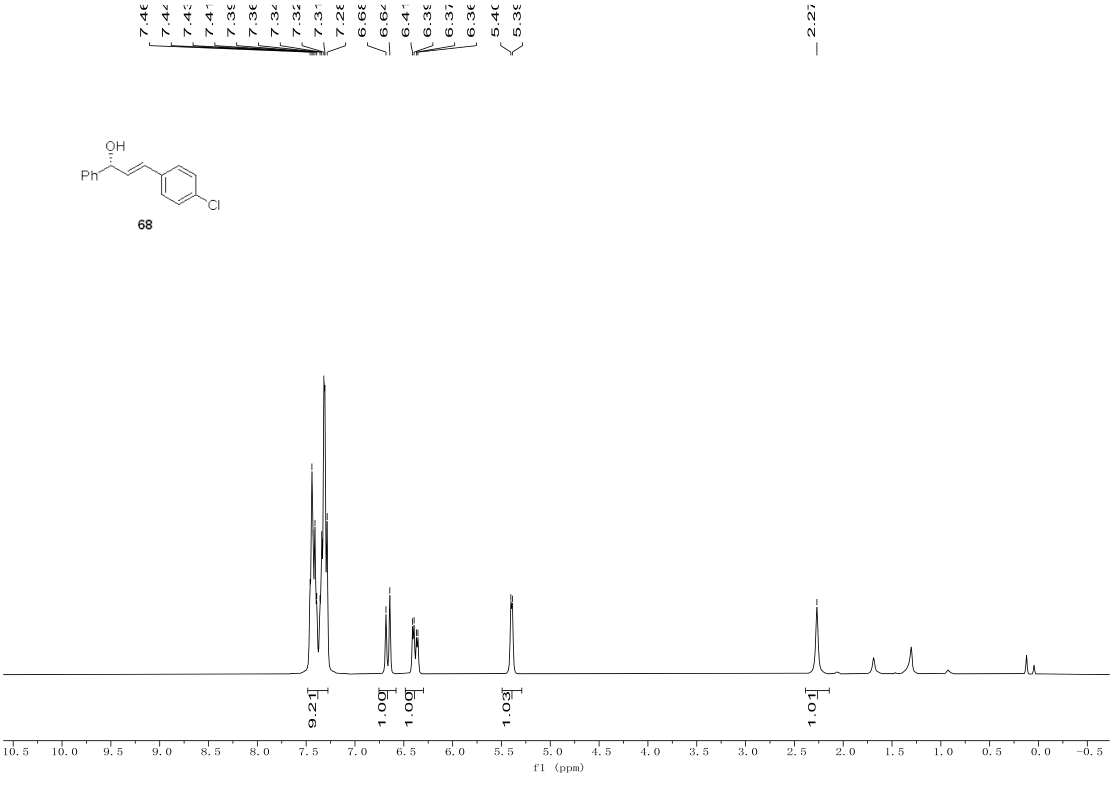

^{13}C NMR of Compound **68** ($CDCl_3$, 101MHz, 20 °C):

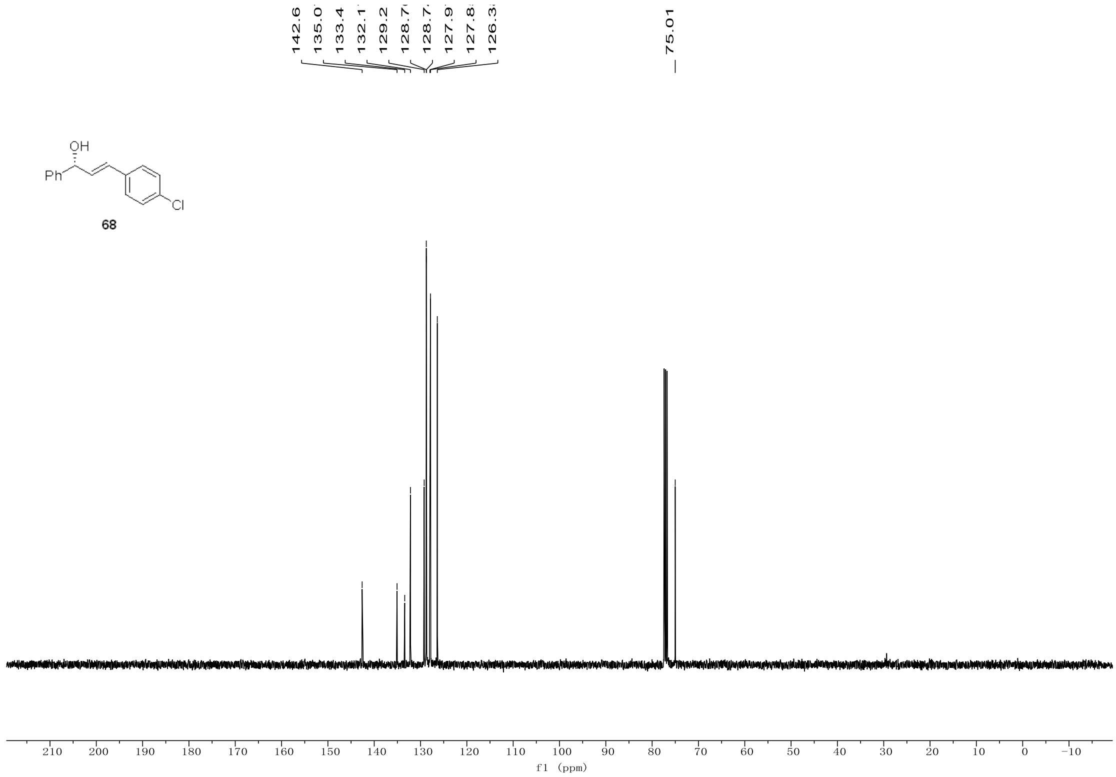

^{1}H NMR of Compound **69** ($CDCl_3$, 400 MHz, 20 °C):

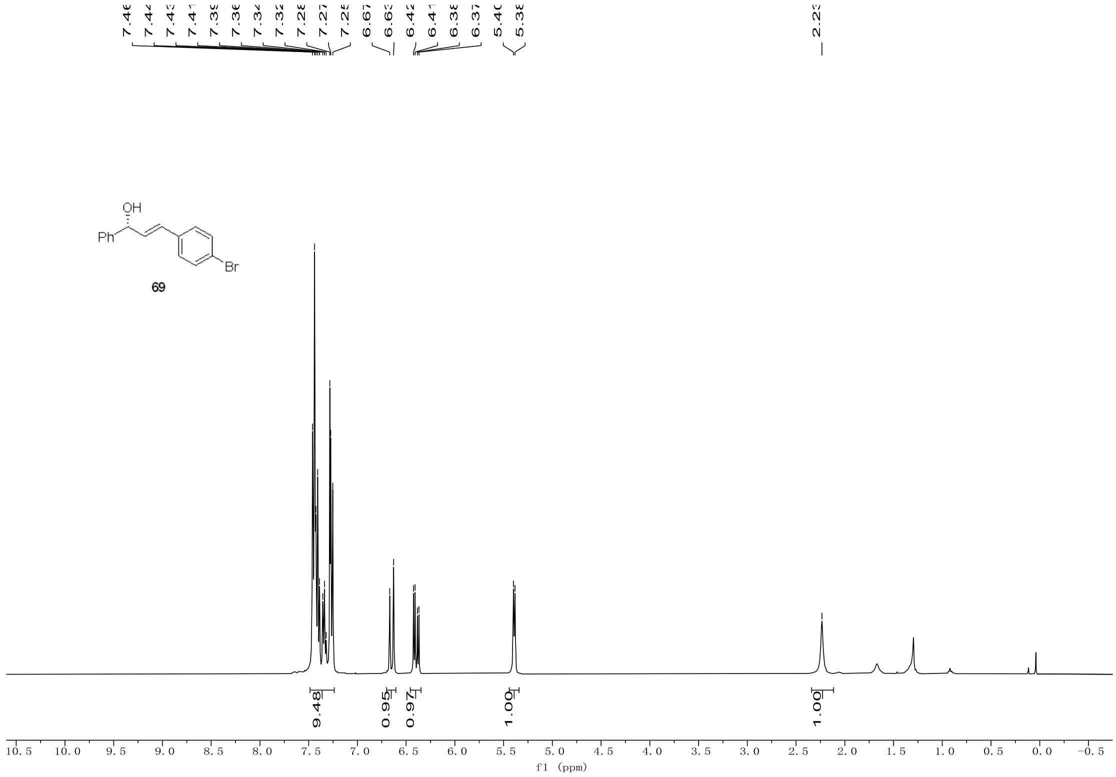

^{13}C NMR of Compound **69** ($CDCl_3$, 101MHz, 20 °C):

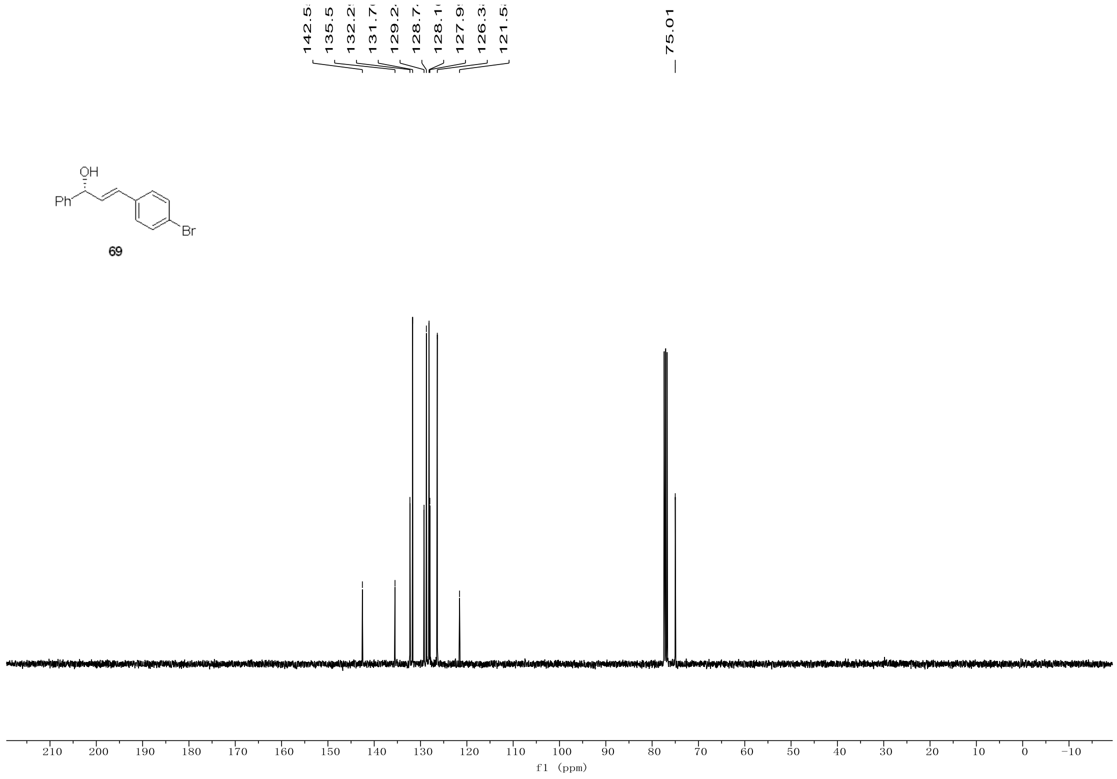

^{1}H NMR of Compound **70** ($CDCl_3$, 400 MHz, 20 °C):

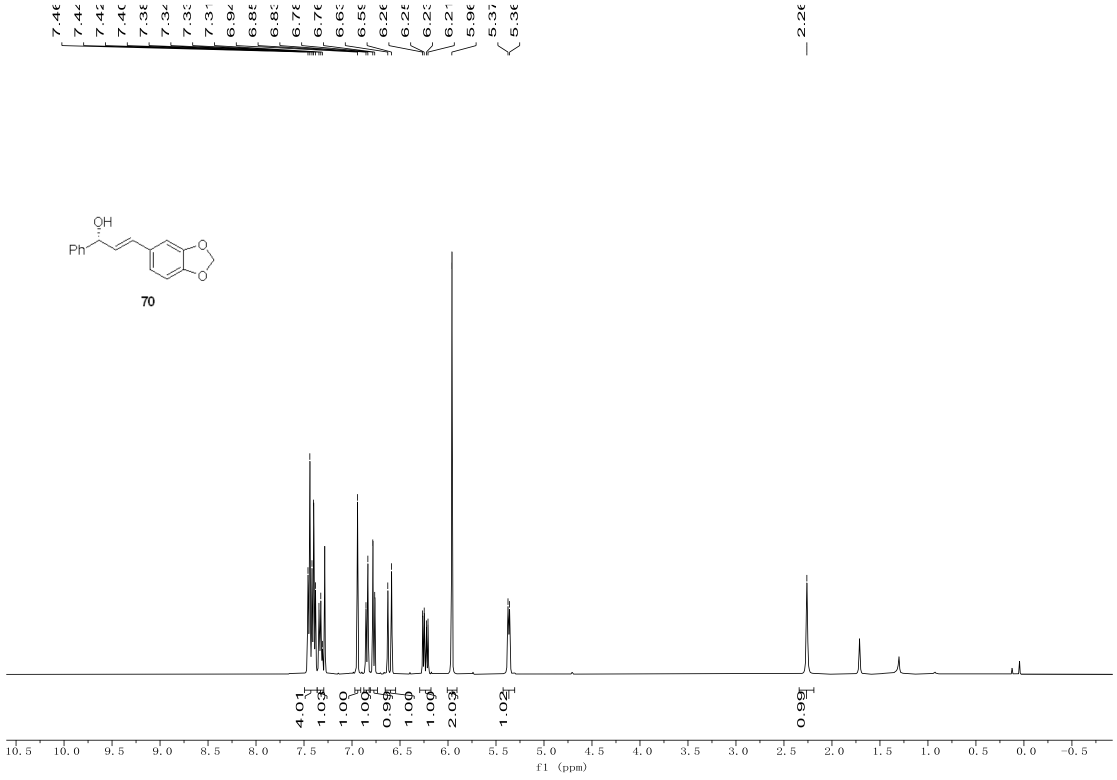

^{13}C NMR of Compound **70** ($CDCl_3$, 101MHz, 20 °C):

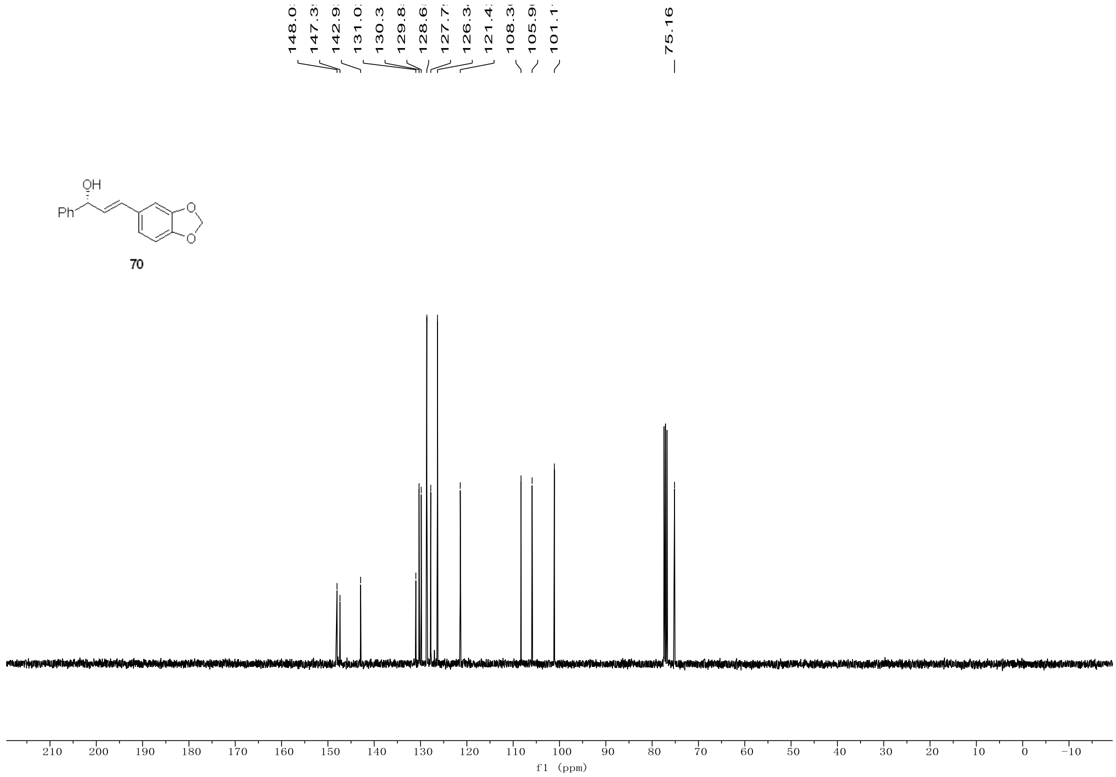

^{1}H NMR of Compound **71** ($CDCl_3$, 400 MHz, 20 °C):

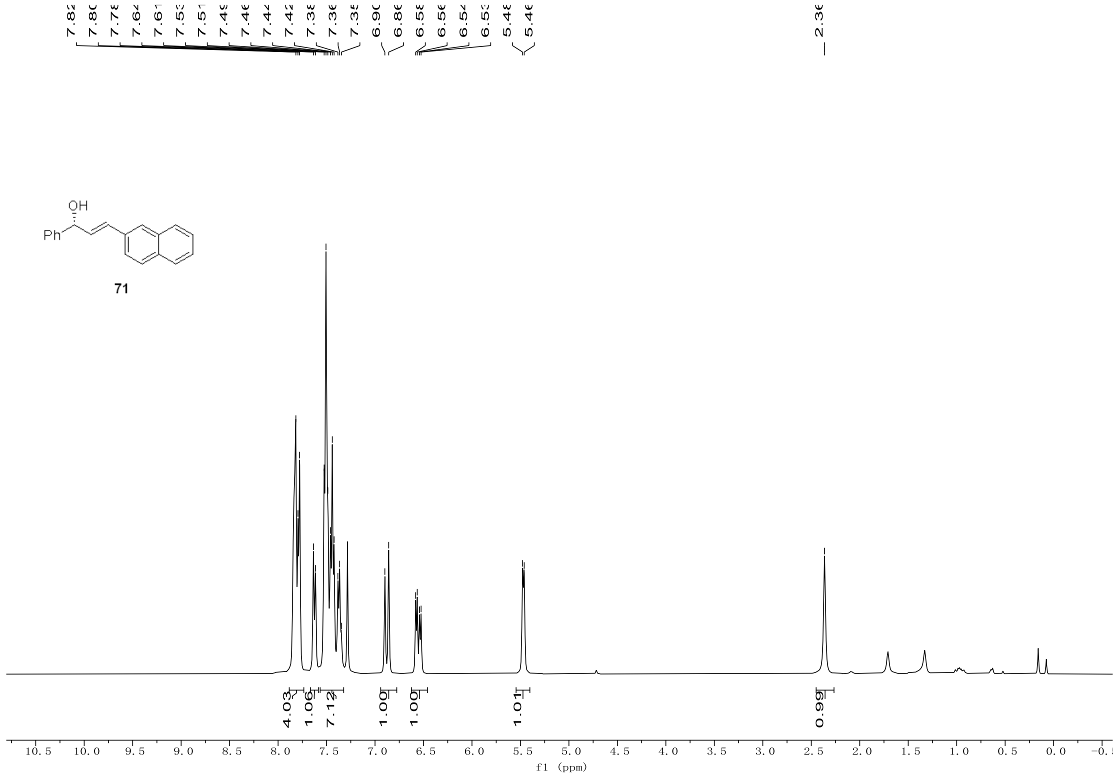

^{13}C NMR of Compound **71** ($CDCl_3$, 101MHz, 20 °C):

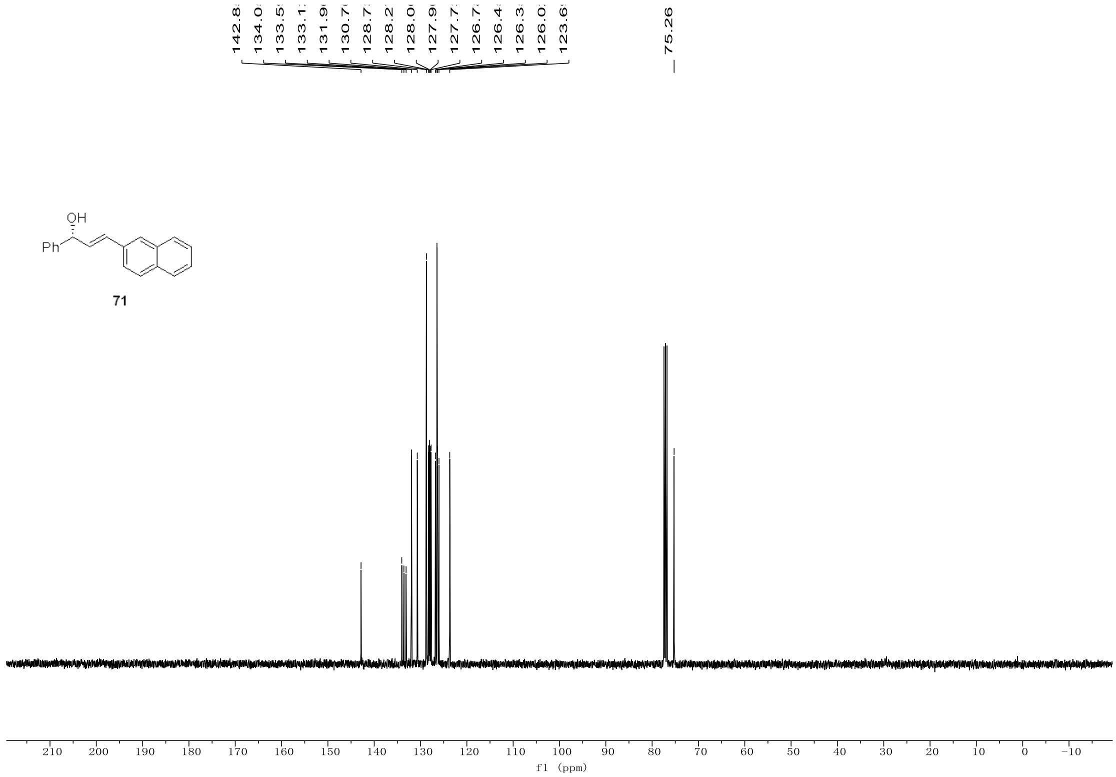

^{1}H NMR of Compound **72** ($CDCl_3$, 400 MHz, 20 °C):

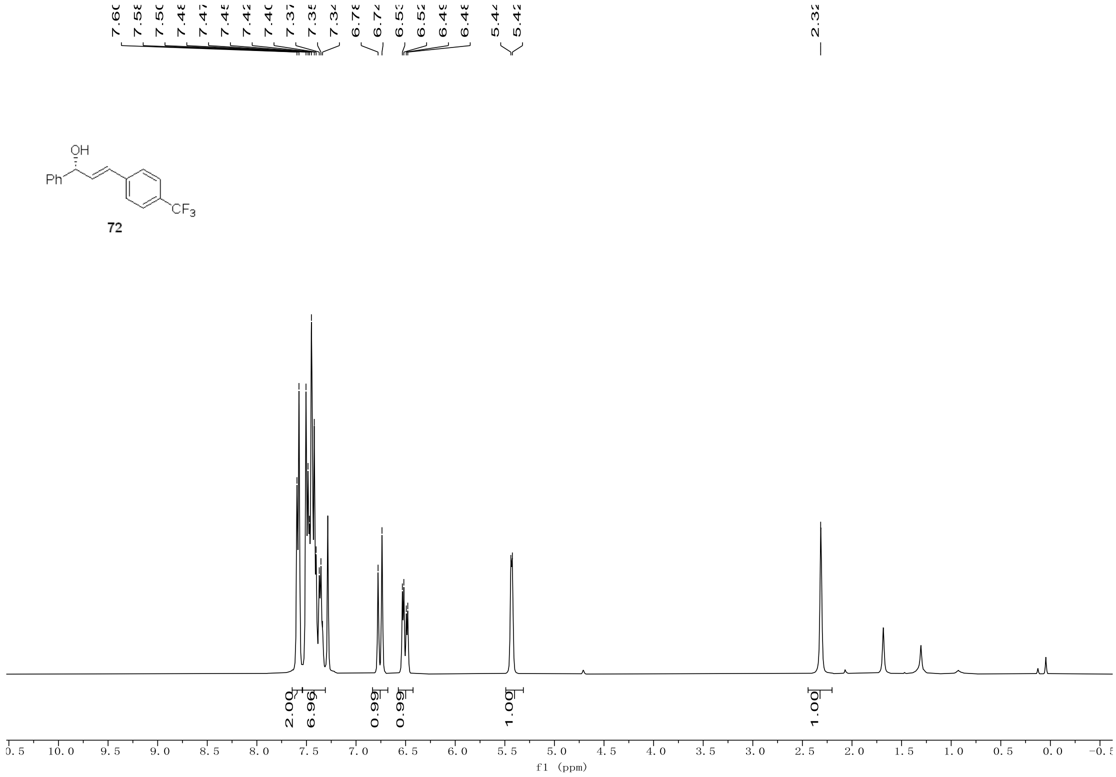

^{13}C NMR of Compound **72** ($CDCl_3$, 101MHz, 20 °C):

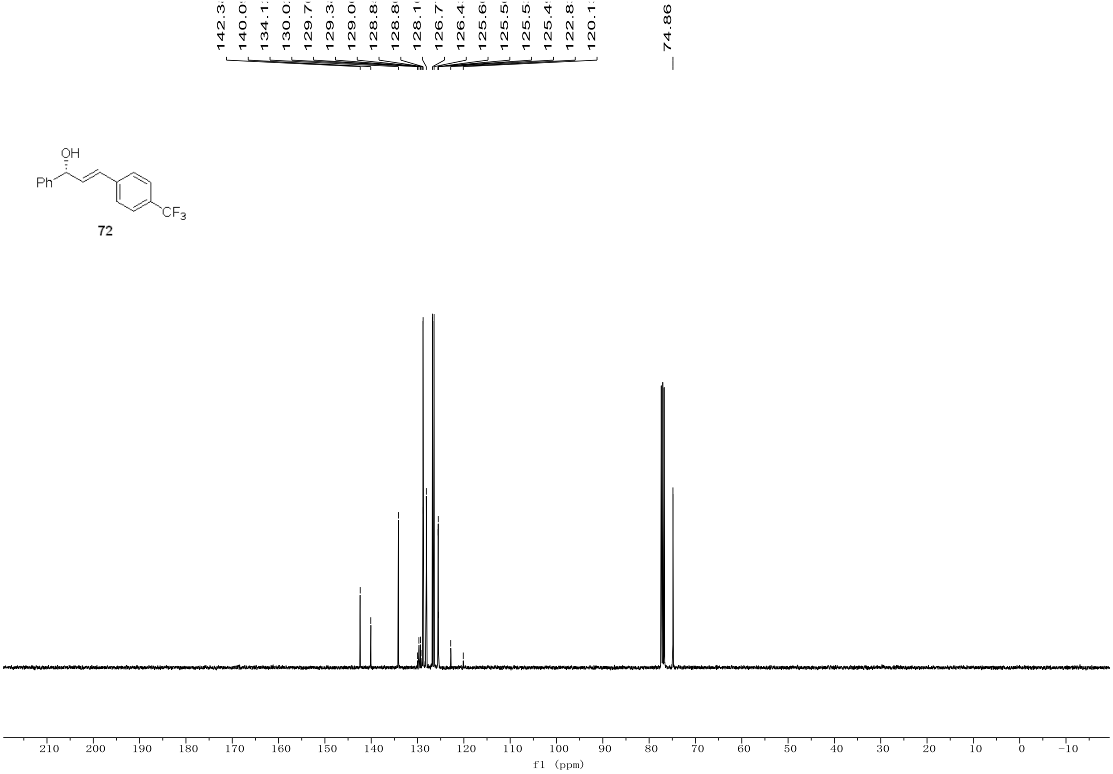

^{19}F NMR of Compound **72** ($CDCl_3$, 376MHz, 20 °C):

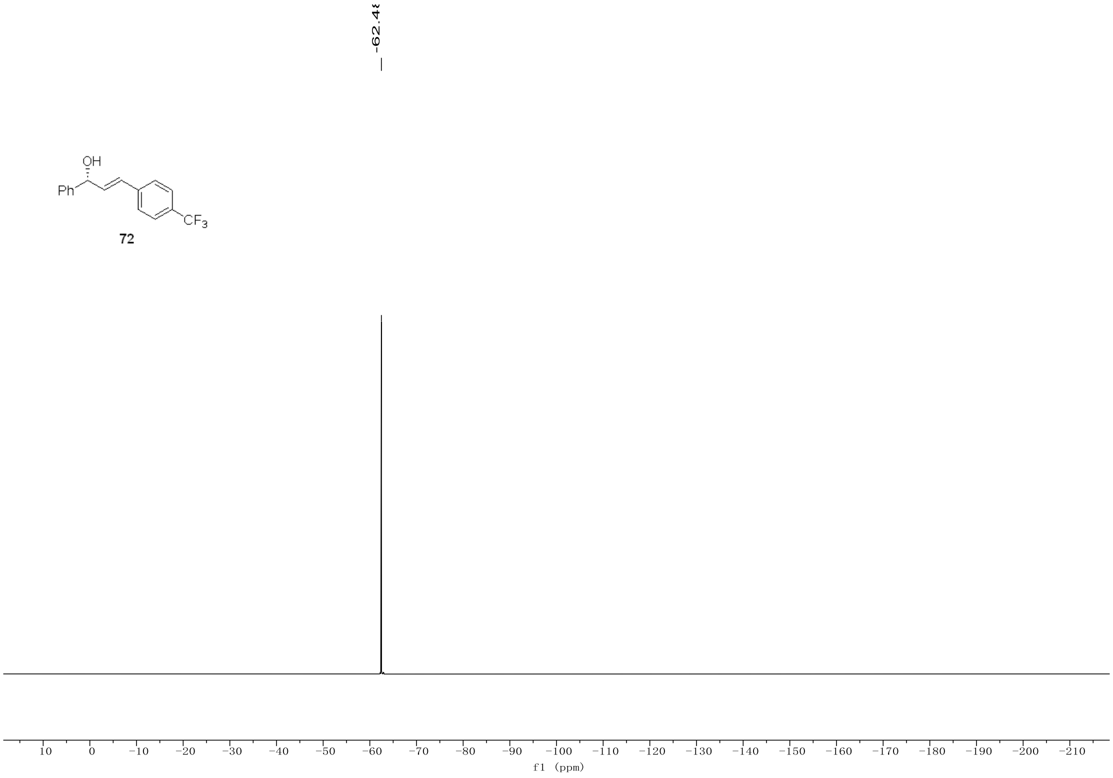

^{1}H NMR of Compound **73** ($CDCl_3$, 400 MHz, 20 °C):

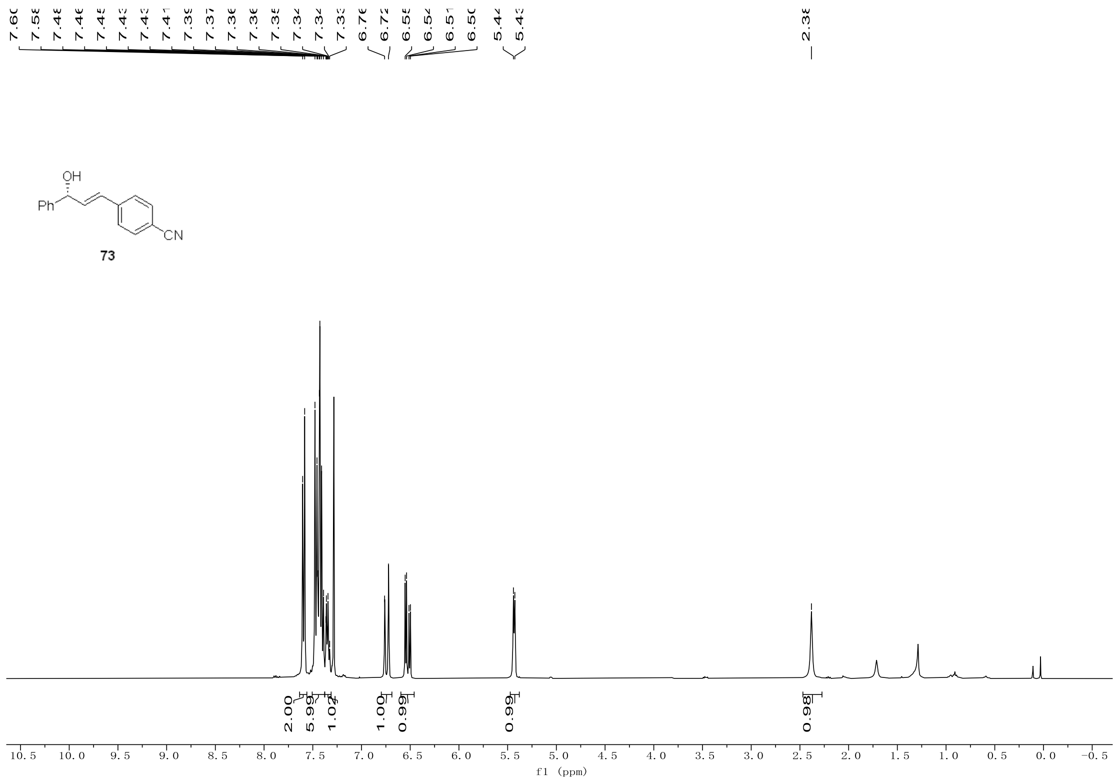

^{13}C NMR of Compound **73** ($CDCl_3$, 101MHz, 20 °C):

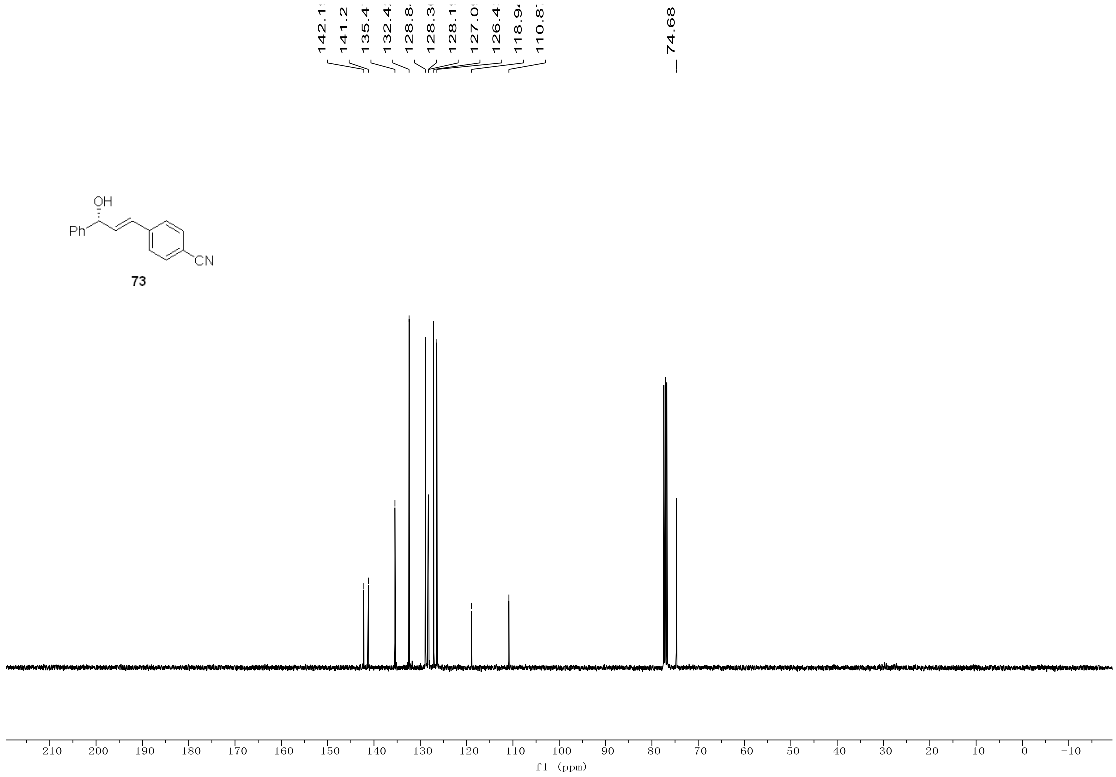

^{1}H NMR of Compound **74** ($CDCl_3$, 400 MHz, 20 °C):

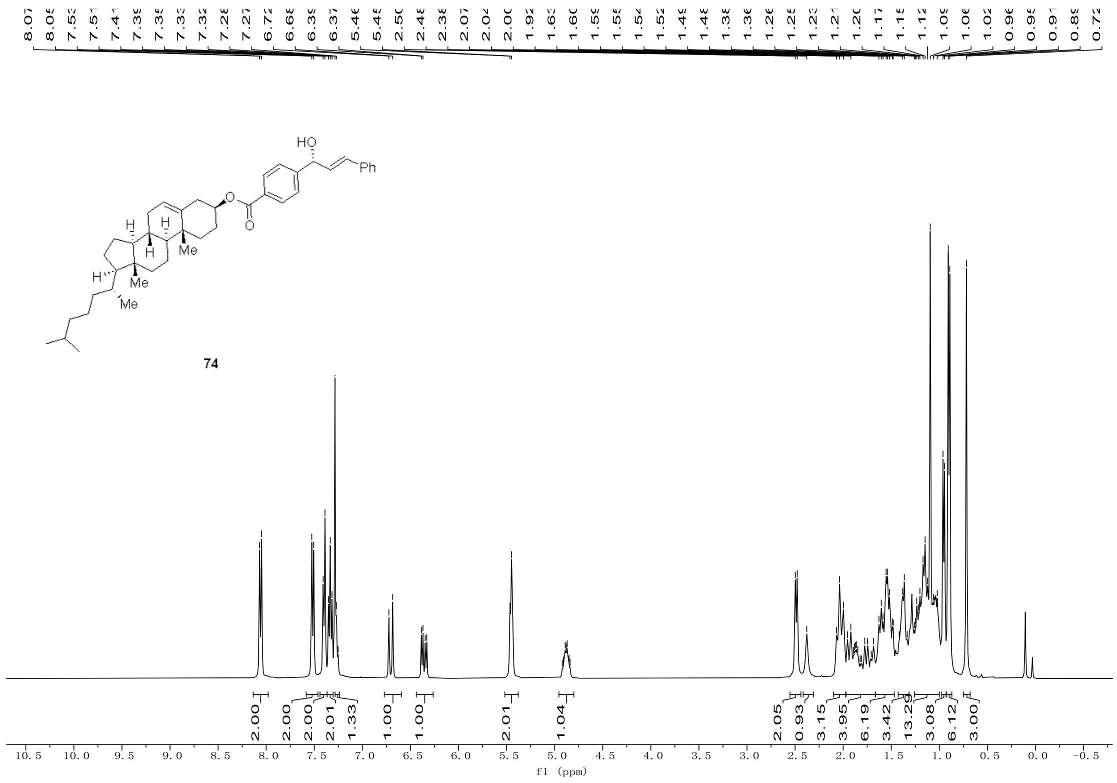

^{13}C NMR of Compound **74** ($CDCl_3$, 101MHz, 20 °C):

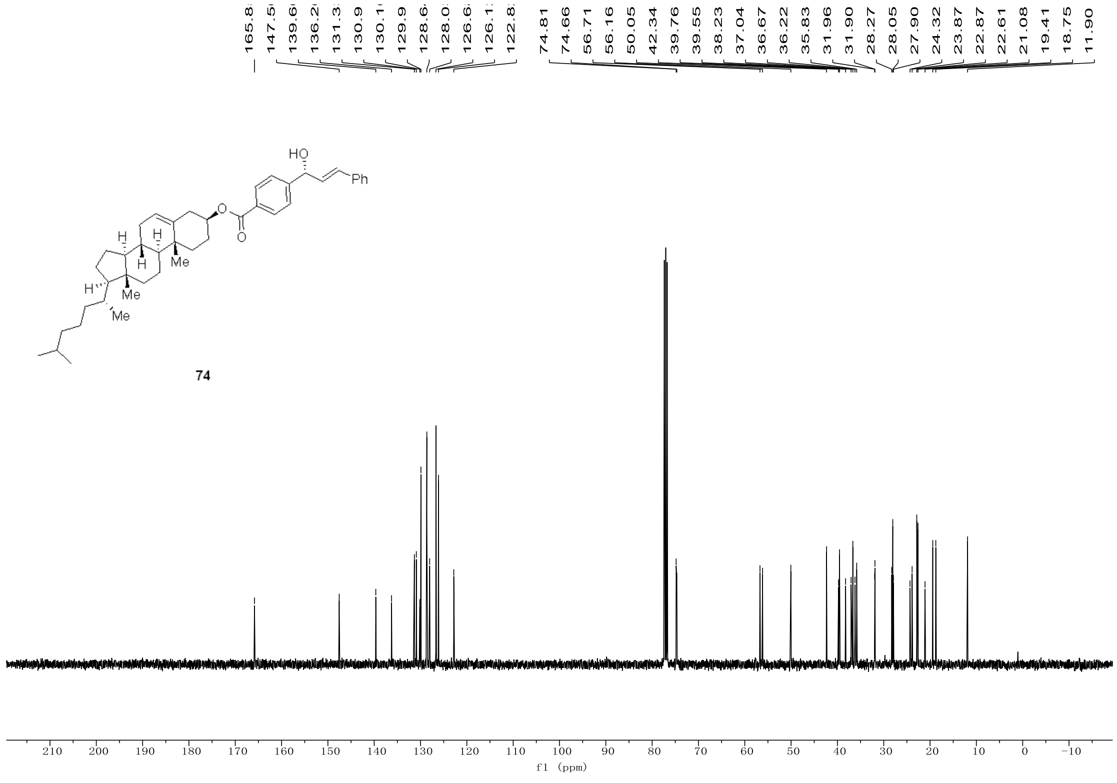

^{1}H NMR of Compound **75** ($CDCl_3$, 400 MHz, 20 °C):

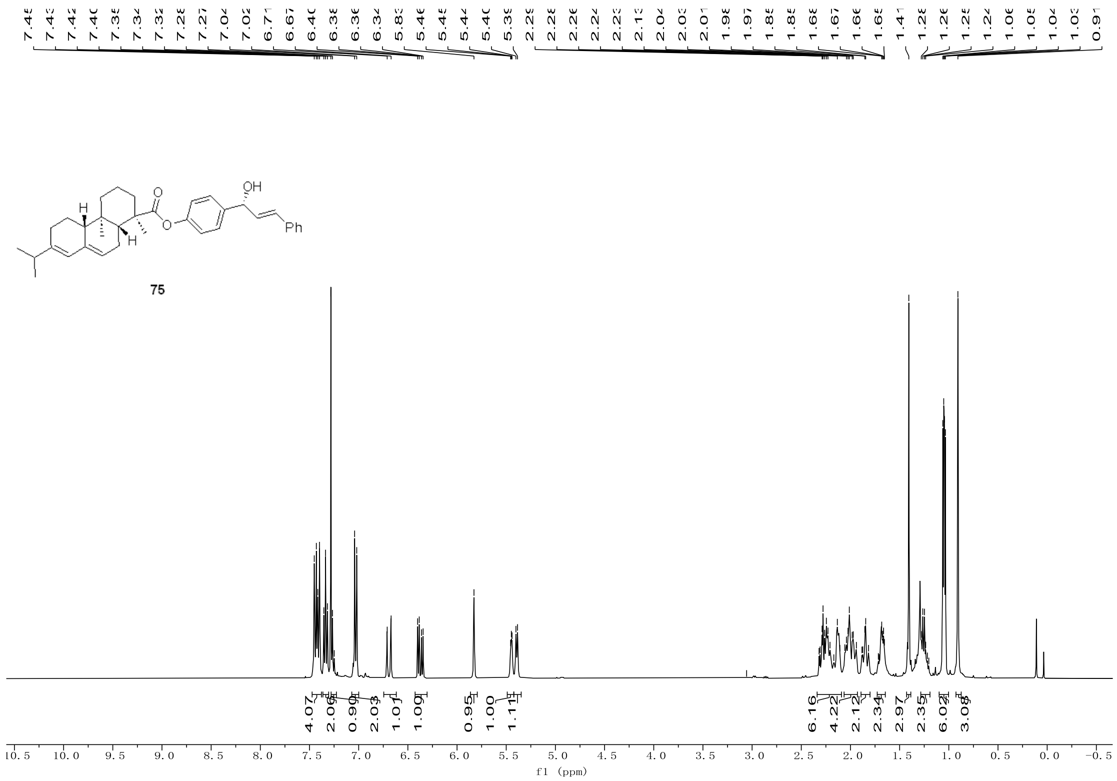

^{13}C NMR of Compound **75** ($CDCl_3$, 101MHz, 20 °C):

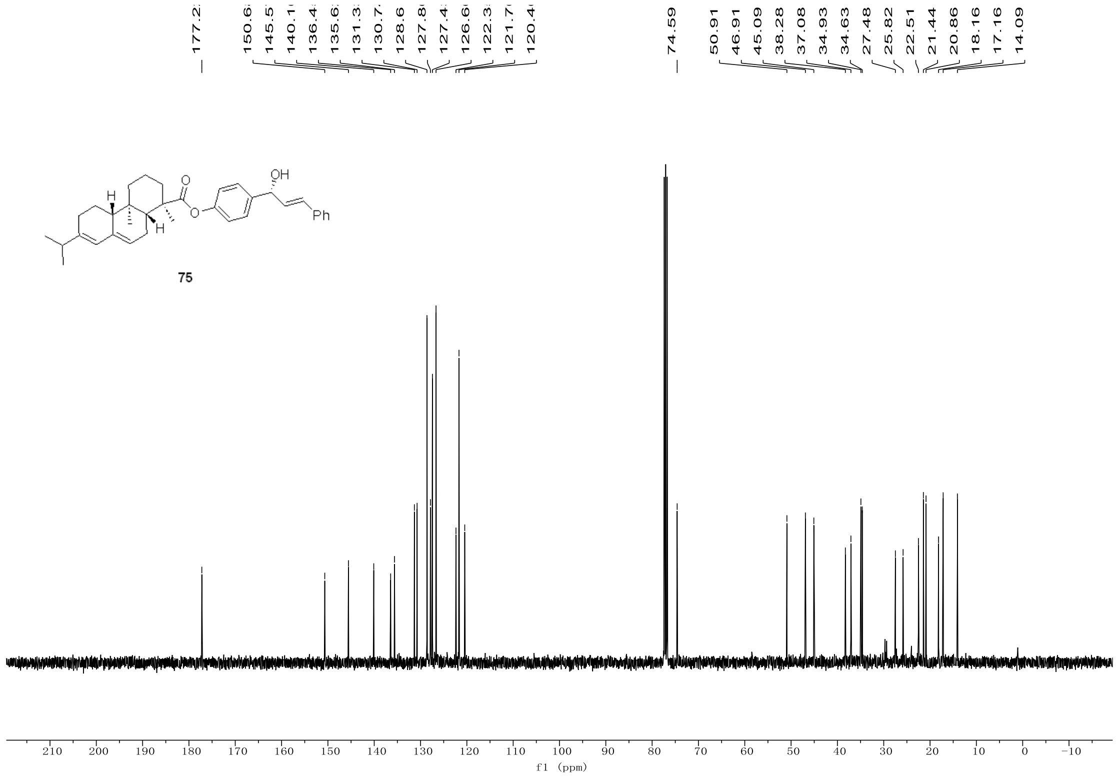

^{1}H NMR of Compound **76** ($CDCl_3$, 400 MHz, 20 °C):

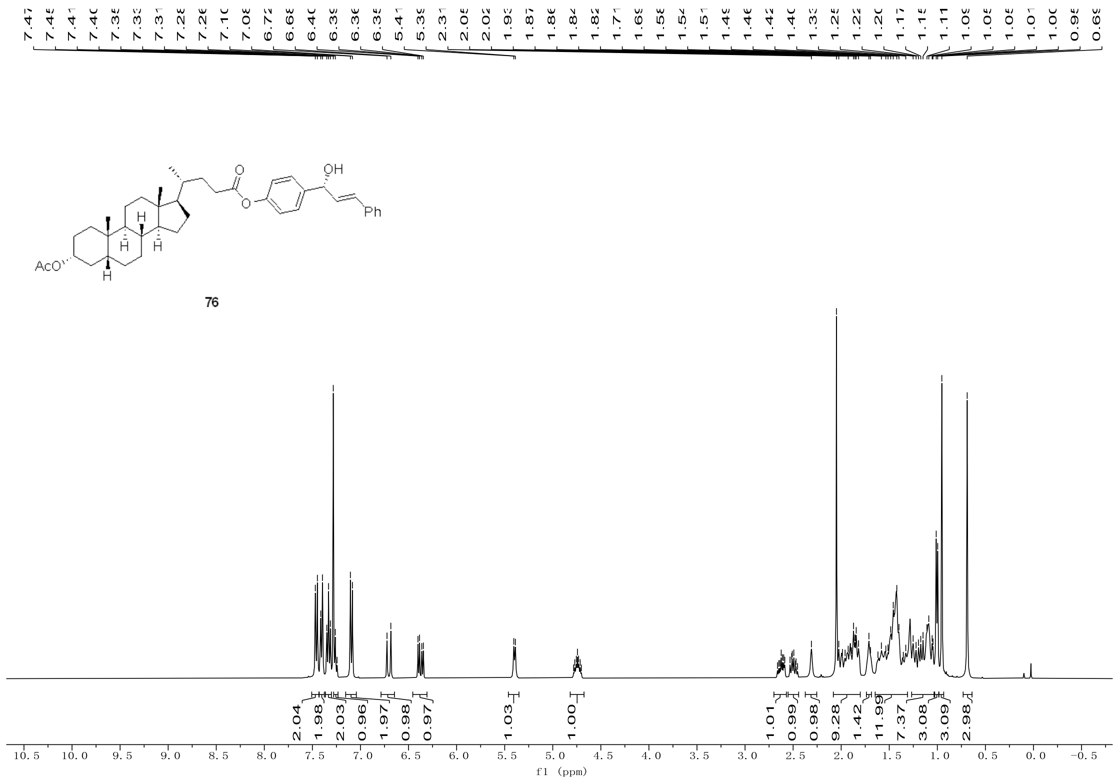

^{13}C NMR of Compound **76** ($CDCl_3$, 101MHz, 20 °C):

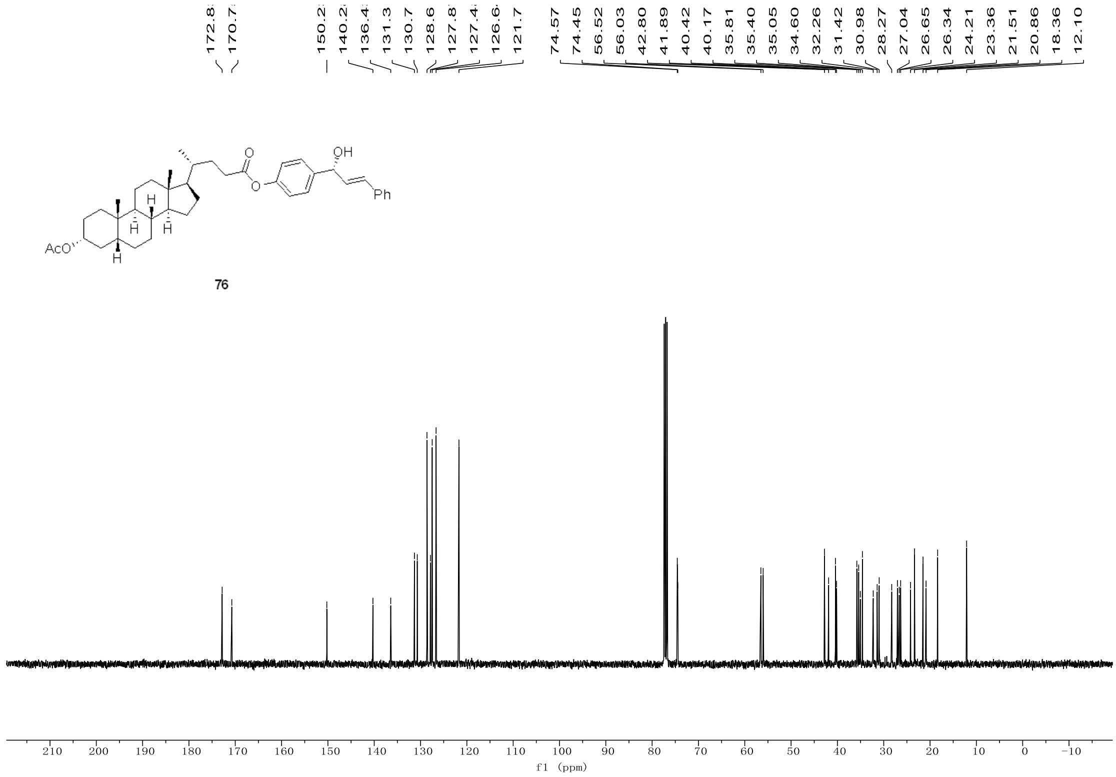

^{1}H NMR of Compound **77** ($CDCl_3$, 400 MHz, 20 °C):

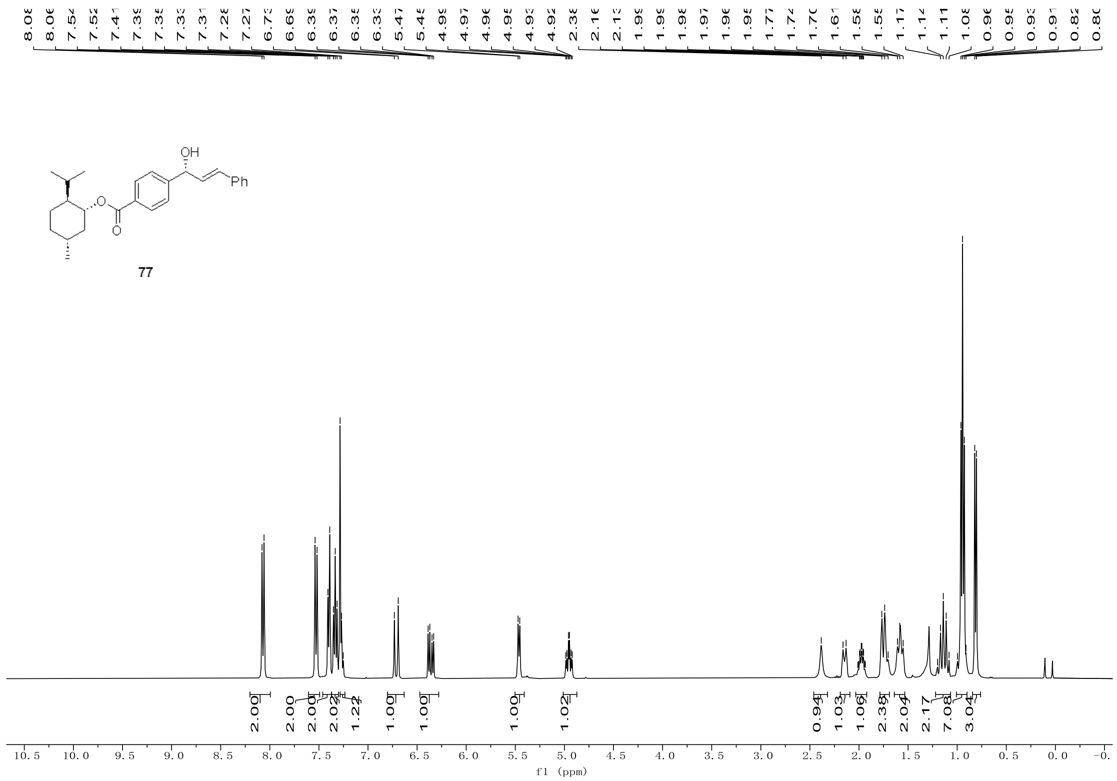

^{13}C NMR of Compound **77** ($CDCl_3$, 101MHz, 20 °C):

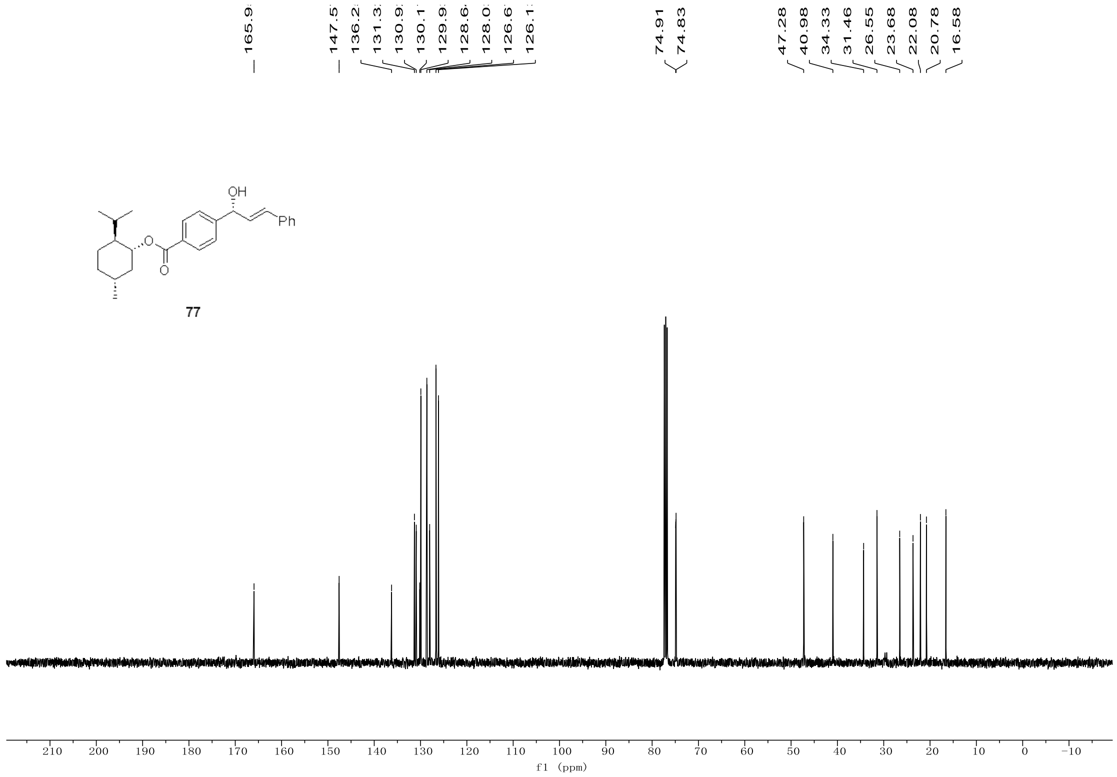

^{1}H NMR of Compound **79** ($CDCl_3$, 400 MHz, 20 °C):

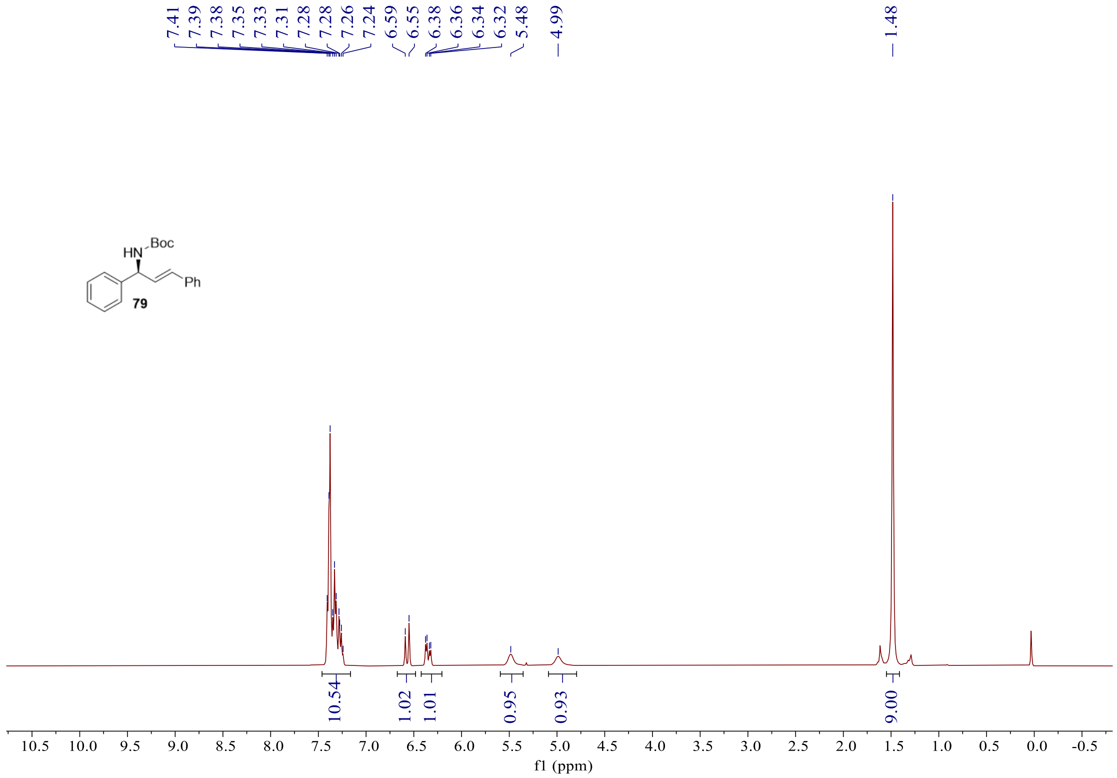

^{13}C NMR of Compound **79** ($CDCl_3$, 101MHz, 20 °C):

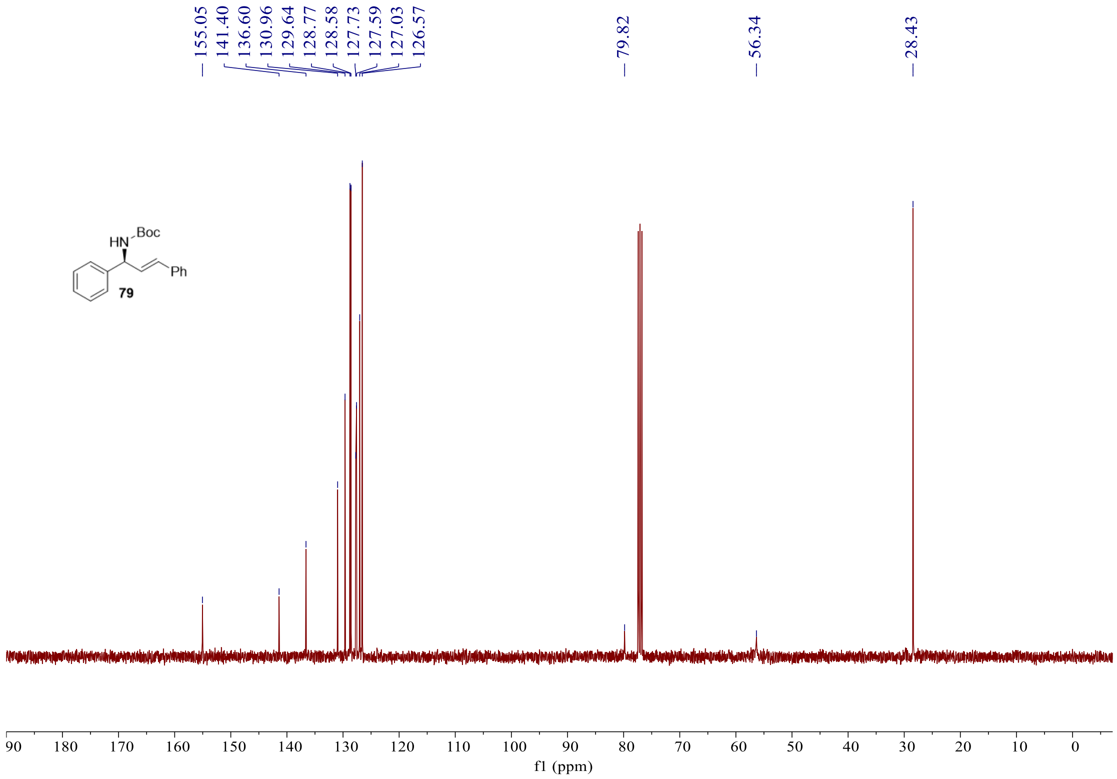

^{1}H NMR of Compound **80** ($CDCl_3$, 400 MHz, 20 °C):

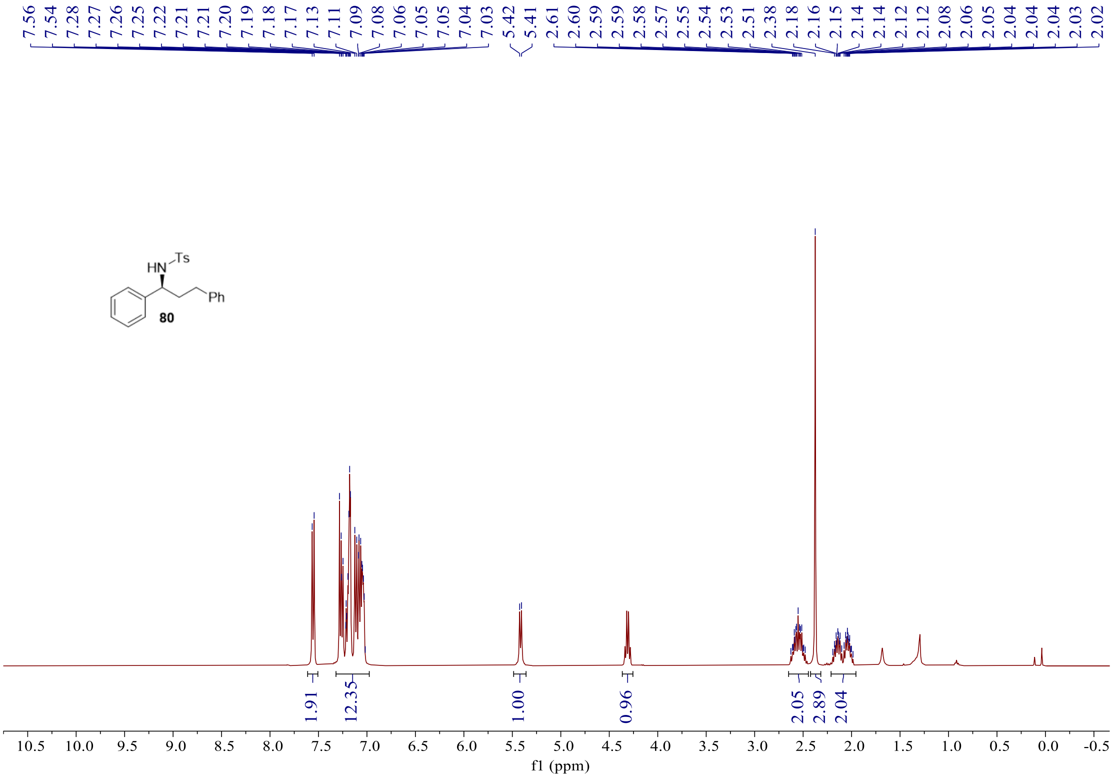

^{13}C NMR of Compound **80** ($CDCl_3$, 101MHz, 20 °C):

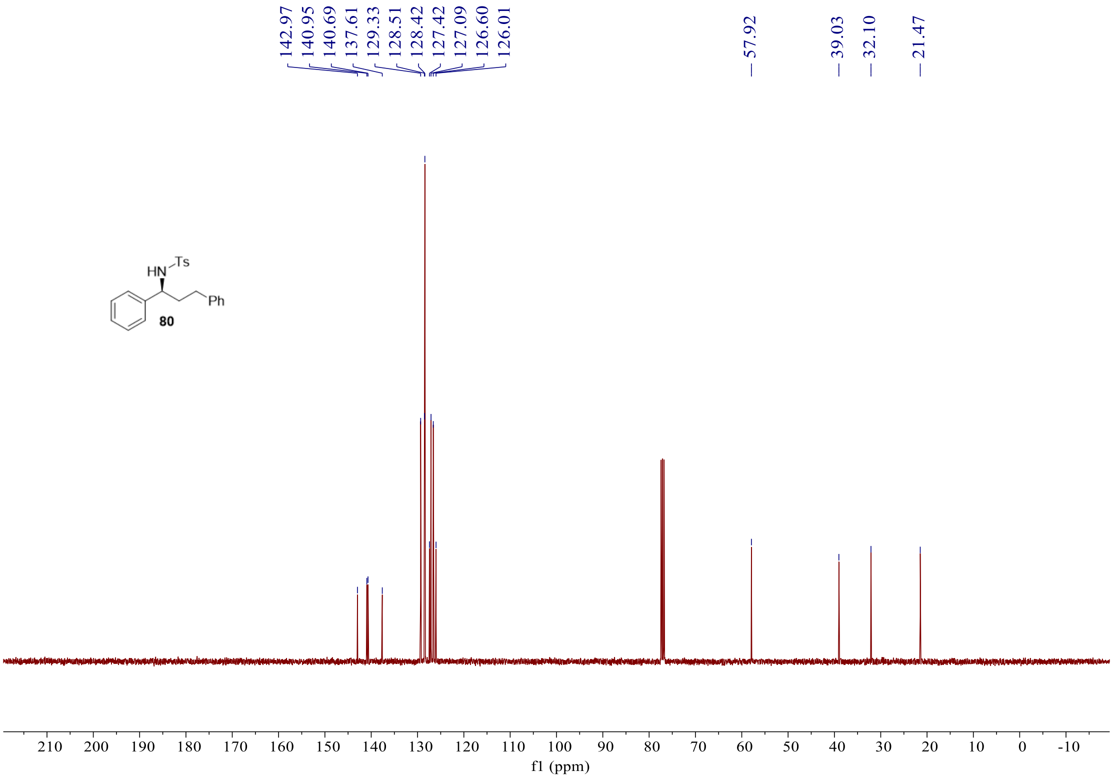

^{1}H NMR of Compound **81** ($CDCl_3$, 400 MHz, 20 °C):

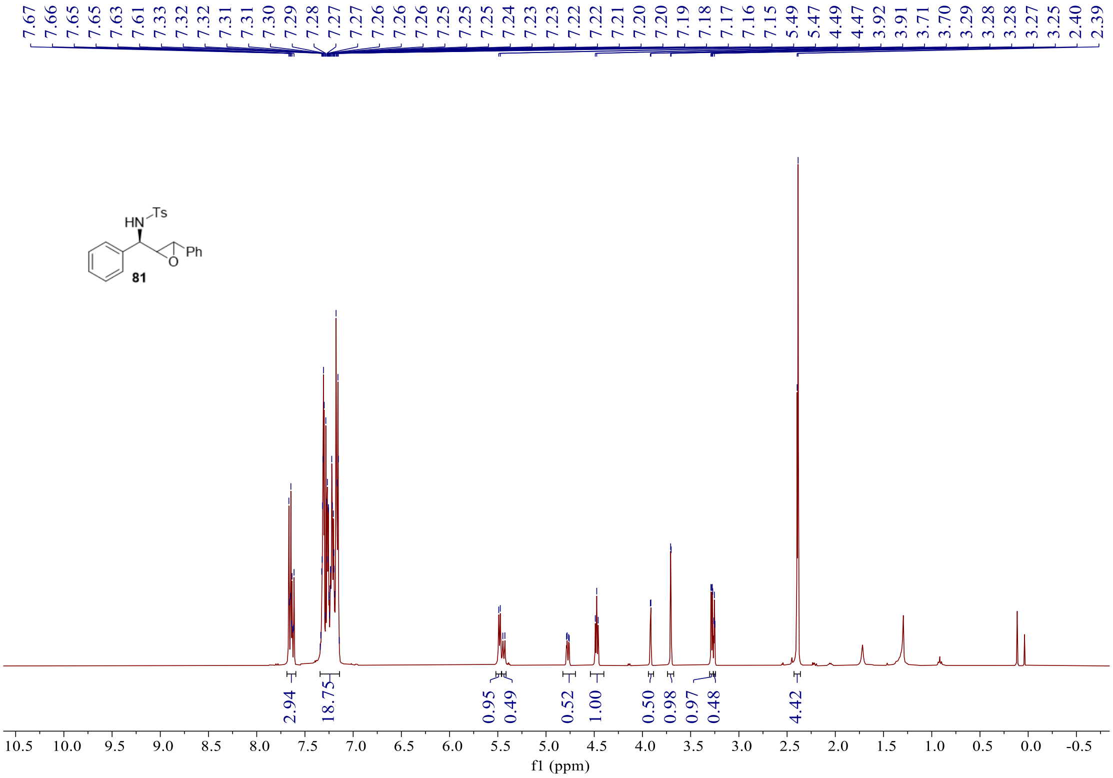

^{13}C NMR of Compound **81** ($CDCl_3$, 101MHz, 20 °C):

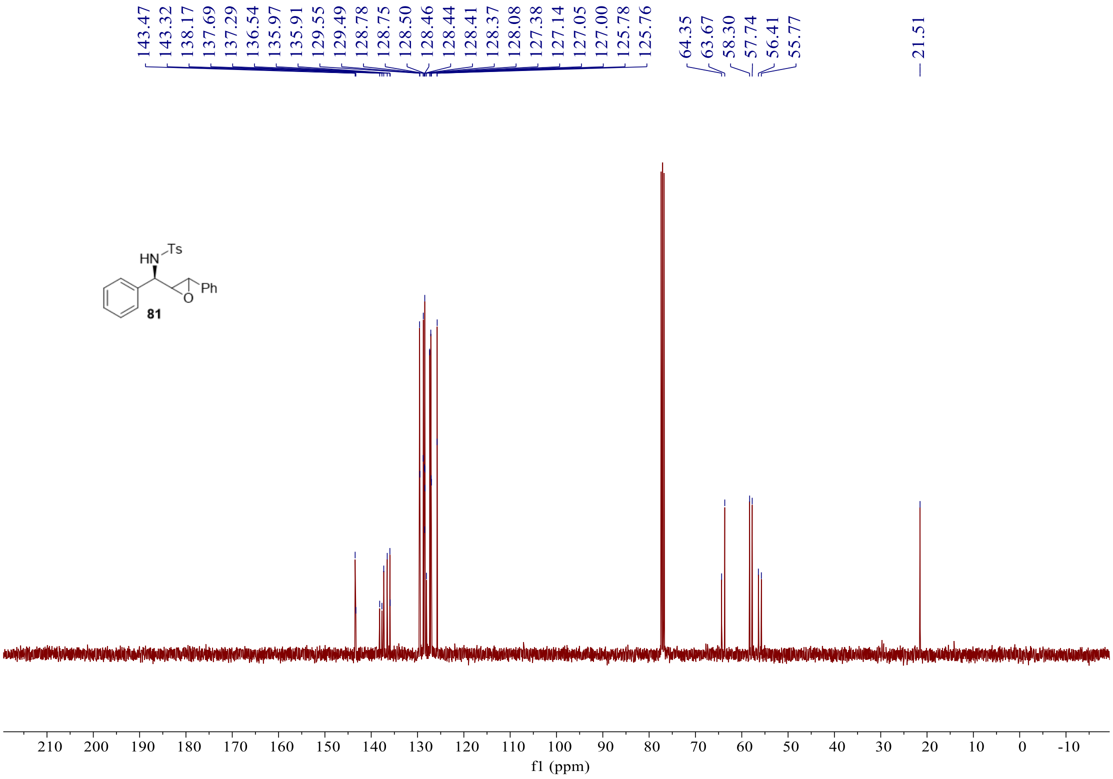

^{1}H NMR of Compound **82** ($CDCl_3$, 400 MHz, 20 °C):

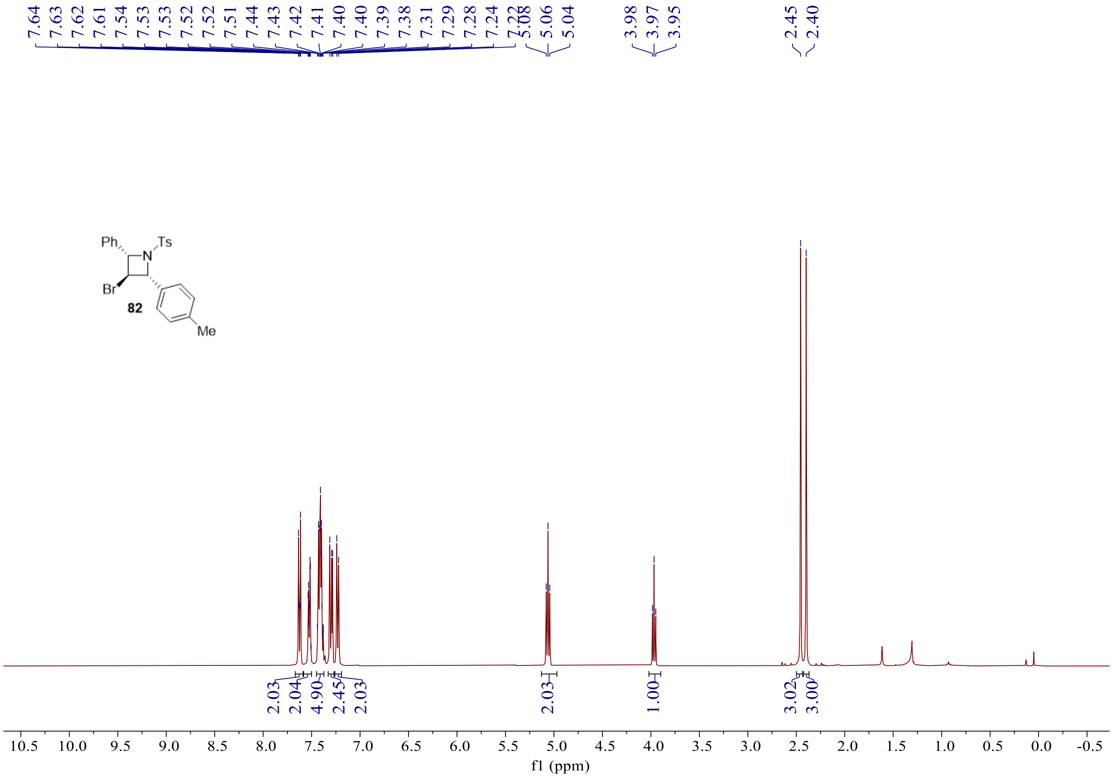

^{13}C NMR of Compound **82** ($CDCl_3$, 101MHz, 20 °C):

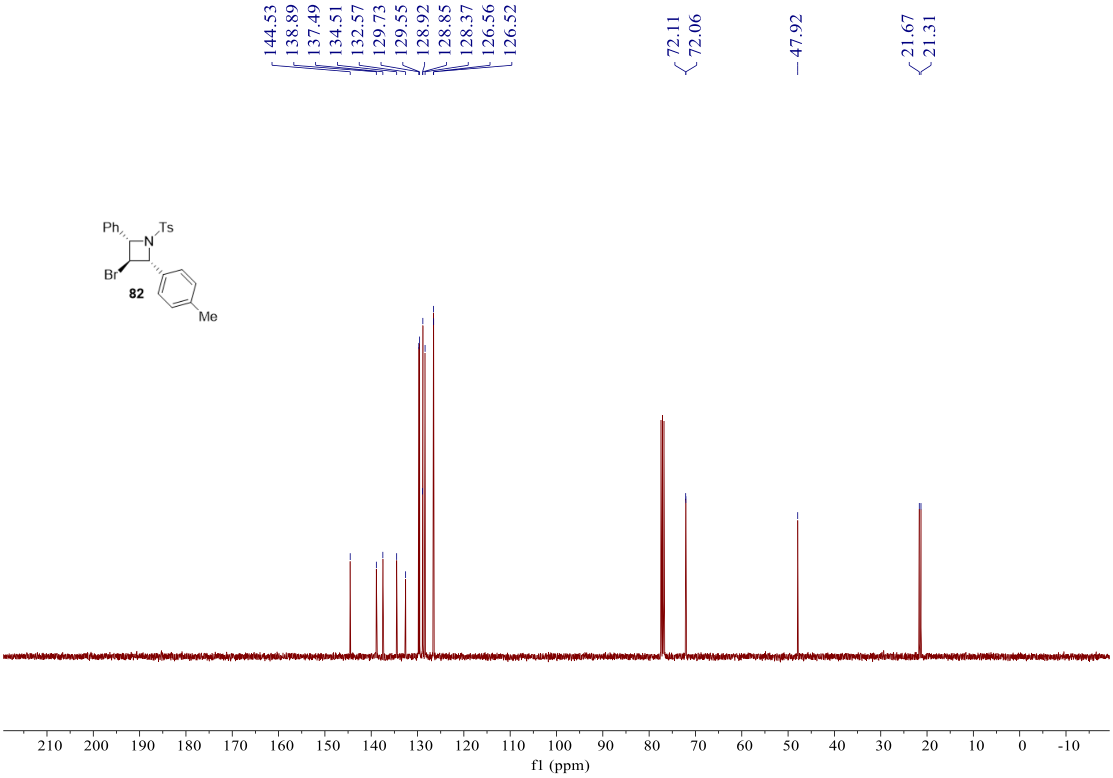

^{1}H NMR of Compound **83** ($CDCl_3$, 400 MHz, 20 °C):

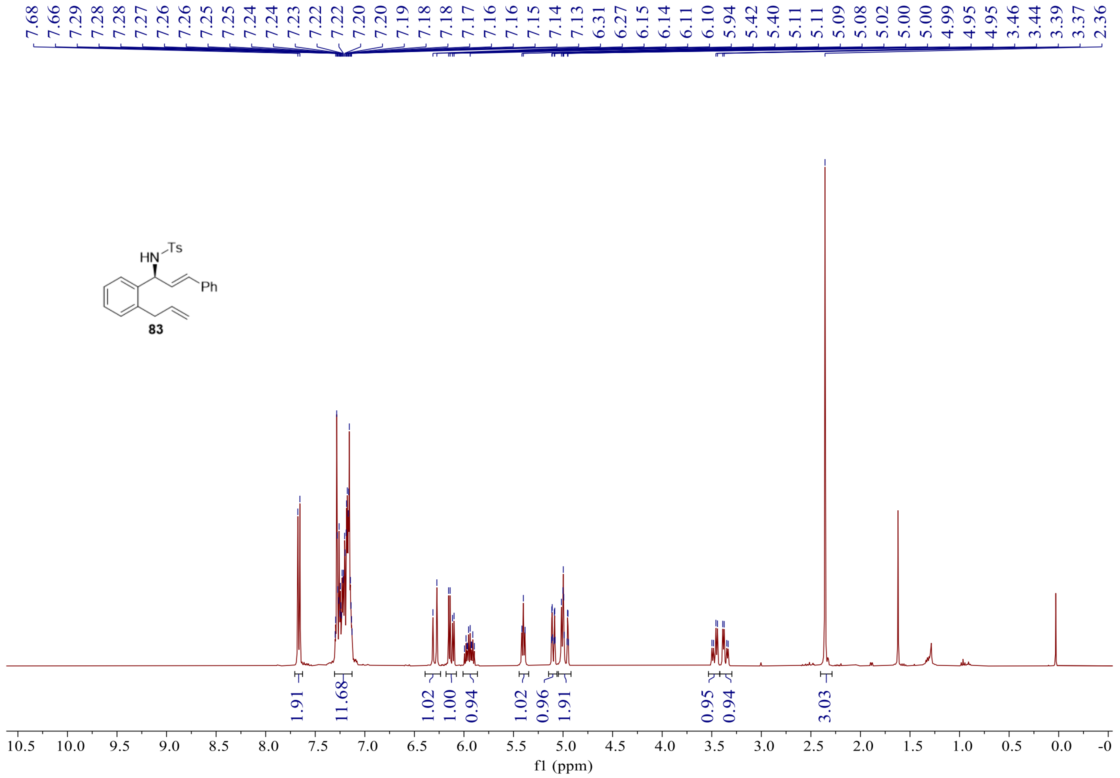

^{13}C NMR of Compound **83** ($CDCl_3$, 101MHz, 20 °C):

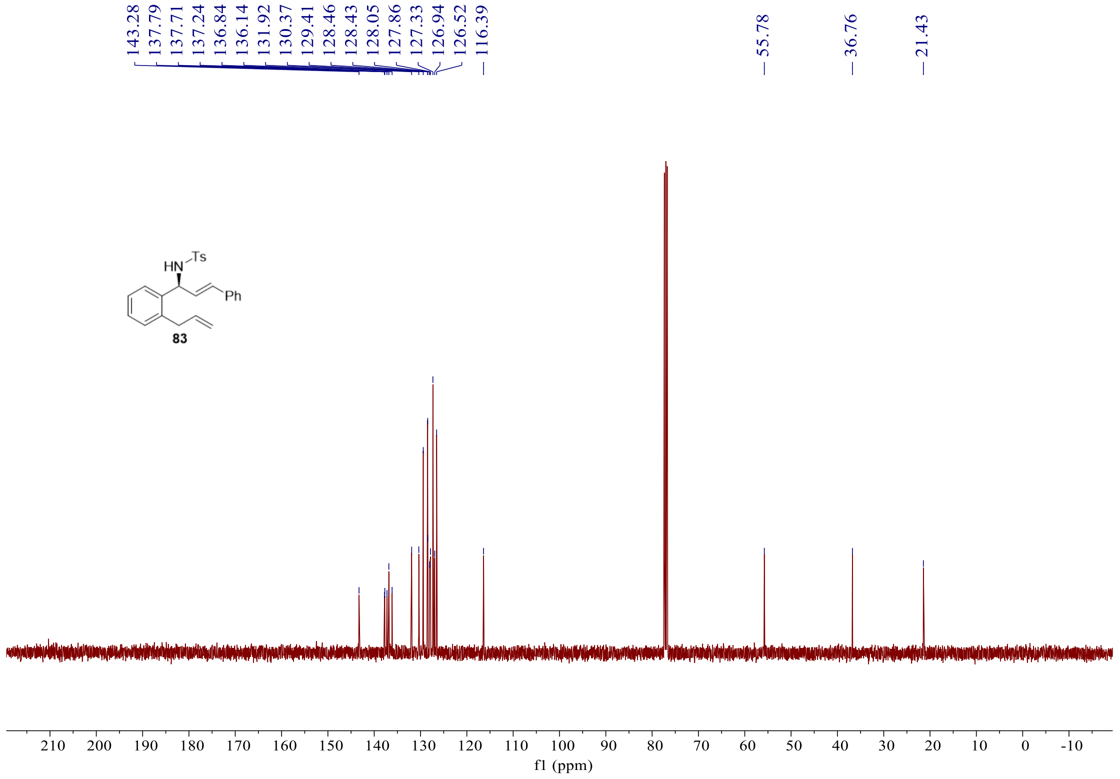

^{1}H NMR of Compound **84** ($CDCl_3$, 400 MHz, 20 °C):

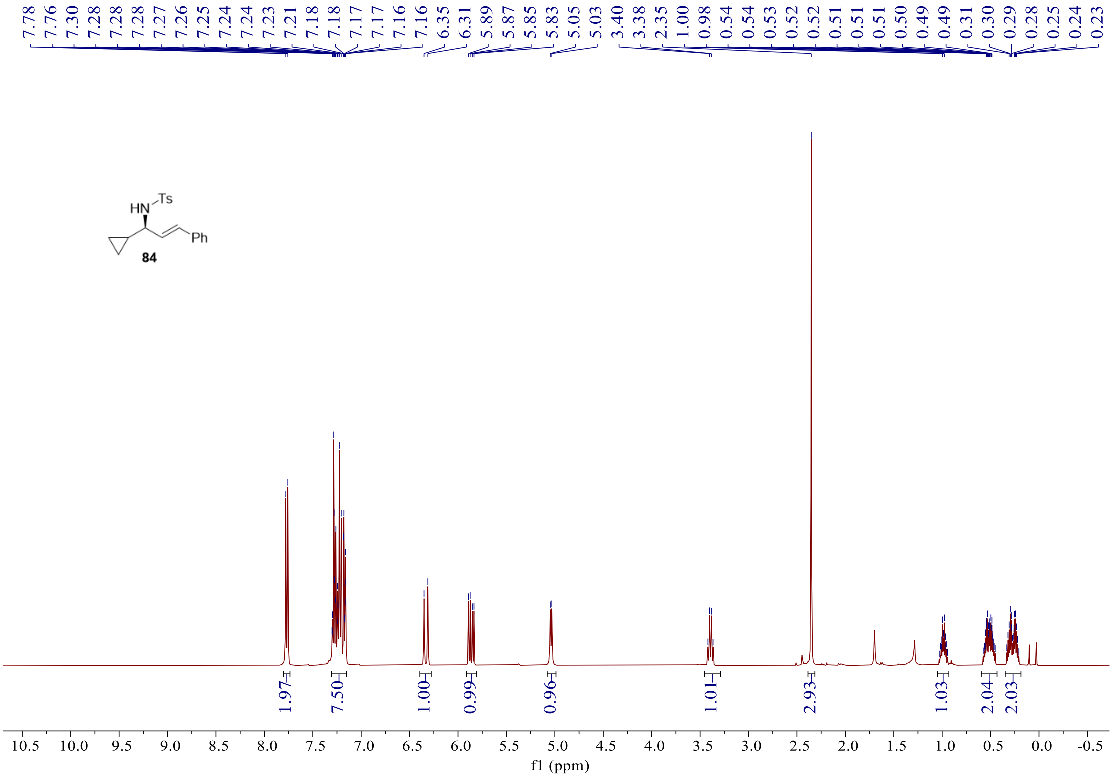

^{13}C NMR of Compound **84** ($CDCl_3$, 101MHz, 20 °C):

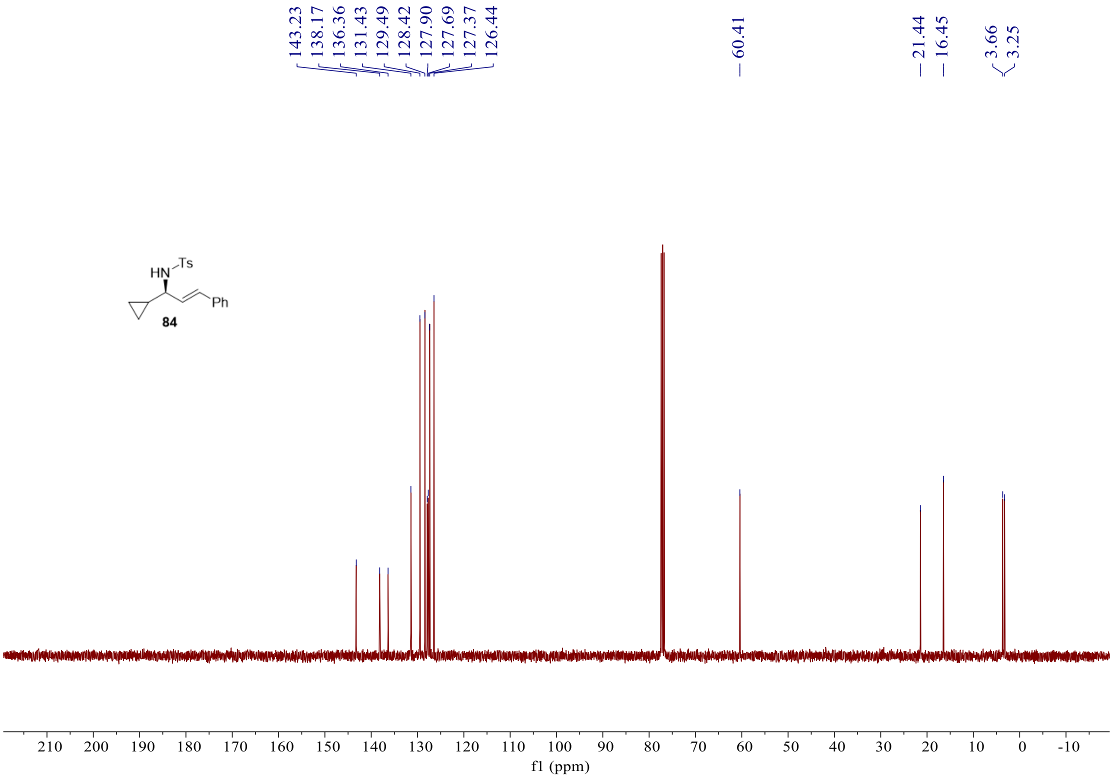

13. HPLC Spectra

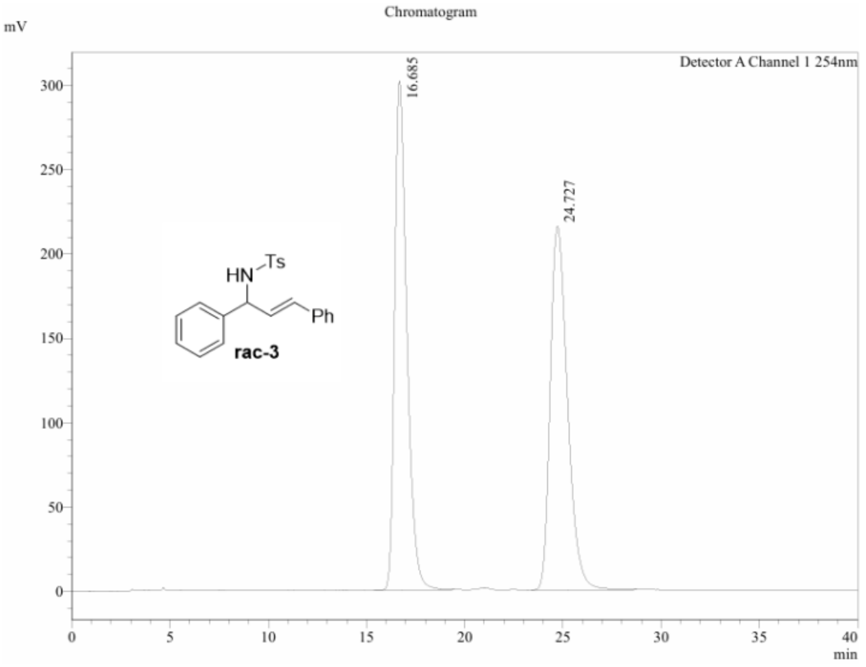

Peak Table

Detector A Channel 1 254nm

Peak#	Ret. Time	Area	Height	Area%	Height%
1	16.685	13142479	301765	50.019	58.329
2	24.727	13132725	215587	49.981	41.671
Total		26275204	517352	100.000	100.000

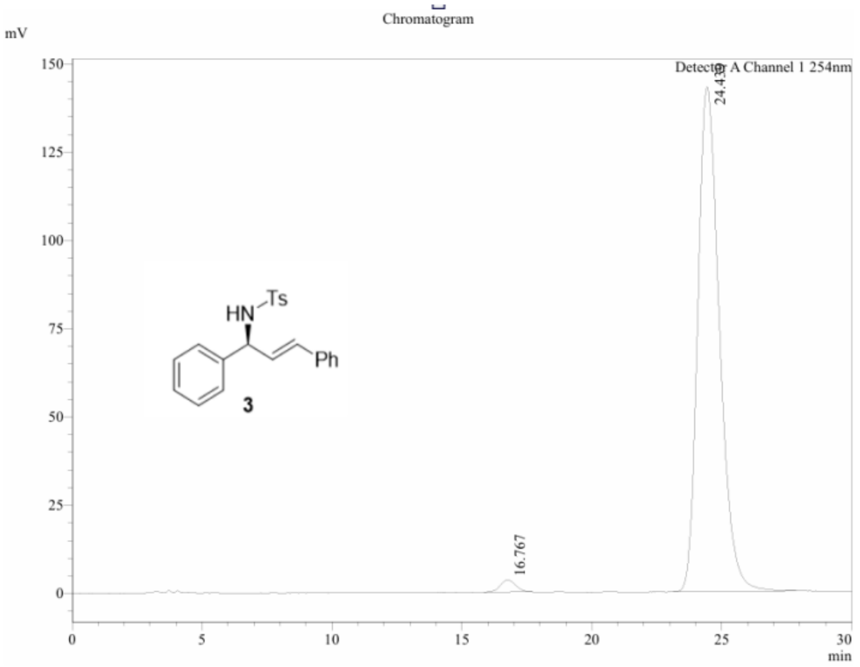

Peak Table

Detector A Channel 1 254nm

Peak#	Ret. Time	Area	Height	Area%	Height%
1	16.767	139838	3412	1.660	2.333
2	24.439	8282180	142859	98.340	97.667
Total		8422018	146271	100.000	100.000

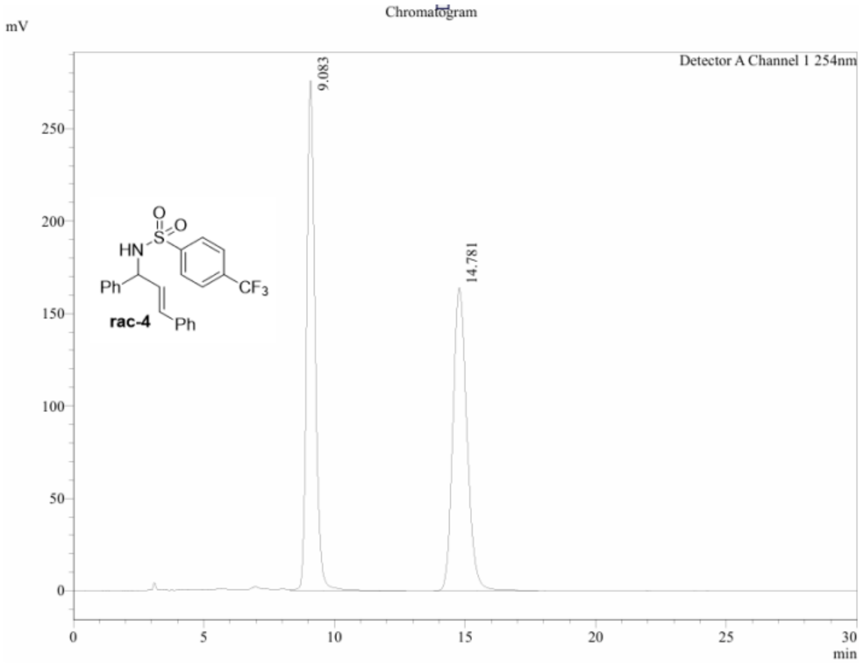

Peak Table

Detector A Channel 1 254nm

Peak#	Ret. Time	Area	Height	Area%	Height%
1	9.083	6191274	275829	50.309	62.750
2	14.781	6115244	163739	49.691	37.250
Total		12306518	439569	100.000	100.000

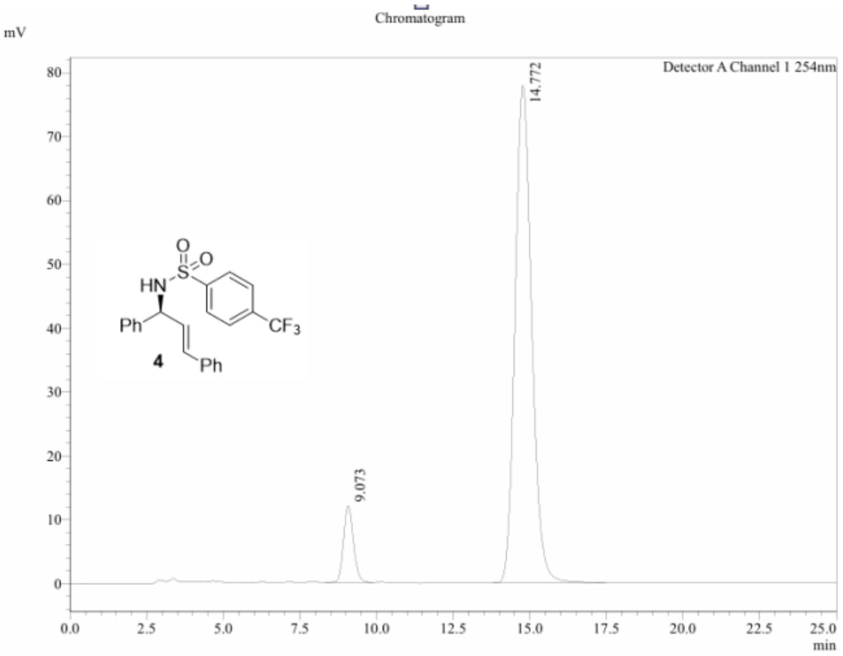

Peak Table

Detector A Channel 1 254nm

Peak#	Ret. Time	Area	Height	Area%	Height%
1	9.073	274201	12062	8.510	13.402
2	14.772	2947976	77940	91.490	86.598
Total		3222178	90002	100.000	100.000

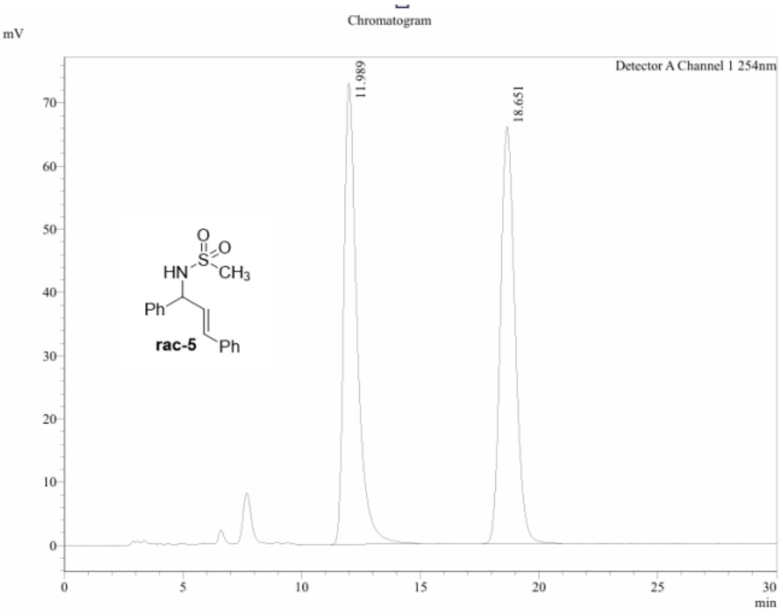

Peak Table

Detector A Channel 1 254nm

Peak#	Ret. Time	Area	Height	Area%	Height%
1	11.989	2813241	72934	49.842	52.502
2	18.651	2831116	65983	50.158	47.498
Total		5644356	138917	100.000	100.000

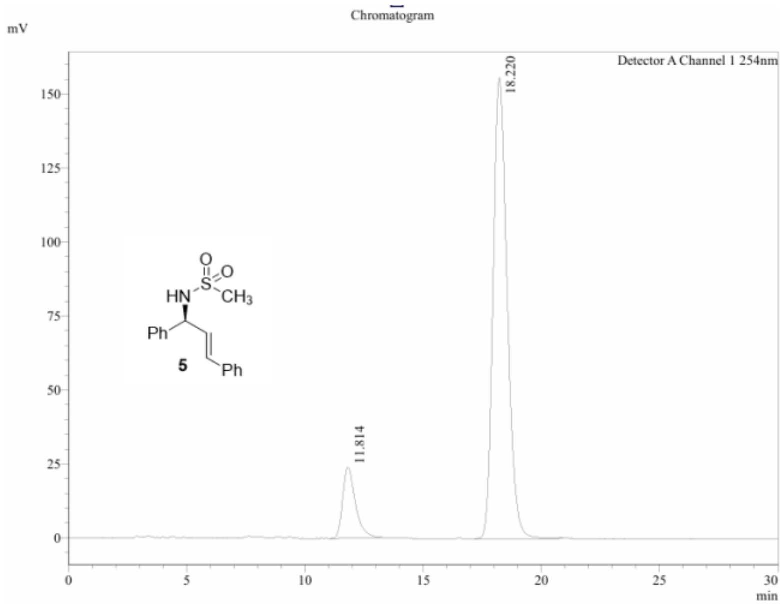

Peak Table

Detector A Channel 1 254nm

Peak#	Ret. Time	Area	Height	Area%	Height%
1	11.814	896226	23968	12.171	13.333
2	18.220	6467208	155794	87.829	86.667
Total		7363434	179762	100.000	100.000

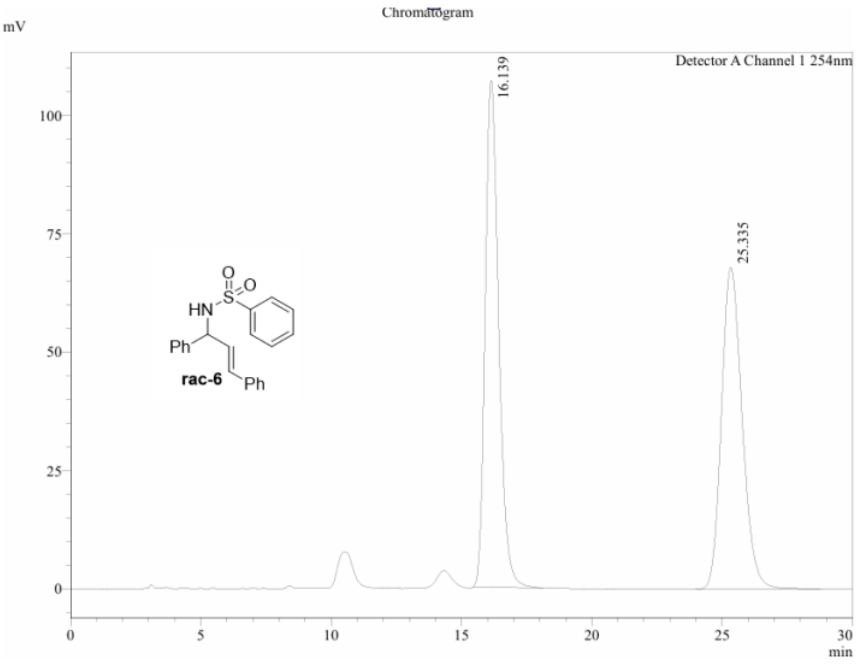

Peak Table

Detector A Channel 1 254nm

Peak#	Ret. Time	Area	Height	Area%	Height%
1	16.139	3855907	106991	50.683	61.172
2	25.335	3751975	67912	49.317	38.828
Total		7607882	174902	100.000	100.000

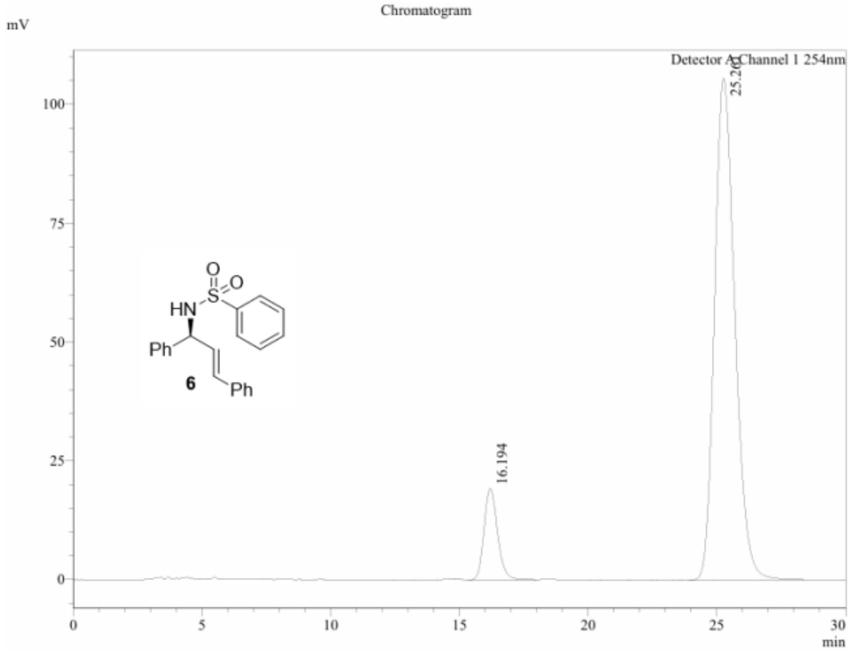

Peak Table

Detector A Channel 1 254nm

Peak#	Ret. Time	Area	Height	Area%	Height%
1	16.194	697999	19250	10.735	15.425
2	25.261	5804066	105546	89.265	84.575
Total		6502065	124796	100.000	100.000

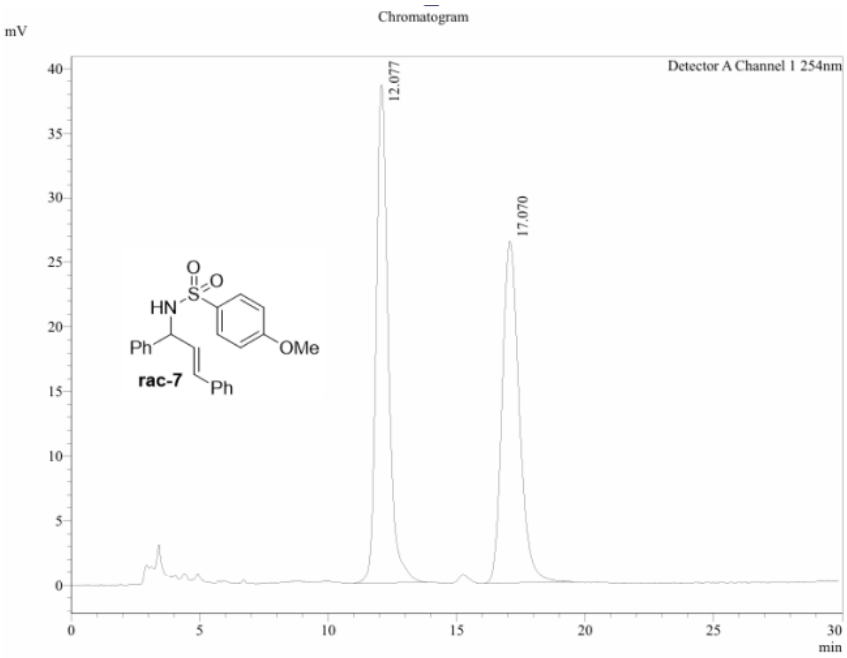

Peak Table

Detector A Channel 1 254nm

Peak#	Ret. Time	Area	Height	Area%	Height%
1	12.077	1256973	38573	50.781	59.314
2	17.070	1218327	26459	49.219	40.686
Total		2475300	65032	100.000	100.000

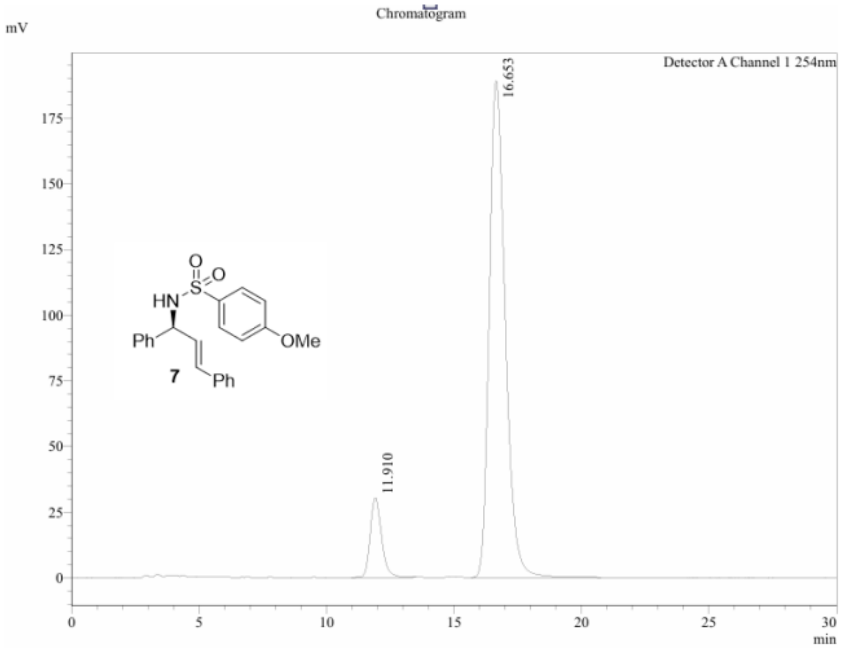

Peak Table

Detector A Channel 1 254nm

Peak#	Ret. Time	Area	Height	Area%	Height%
1	11.910	933710	30340	10.173	13.826
2	16.653	8244466	189095	89.827	86.174
Total		9178176	219434	100.000	100.000

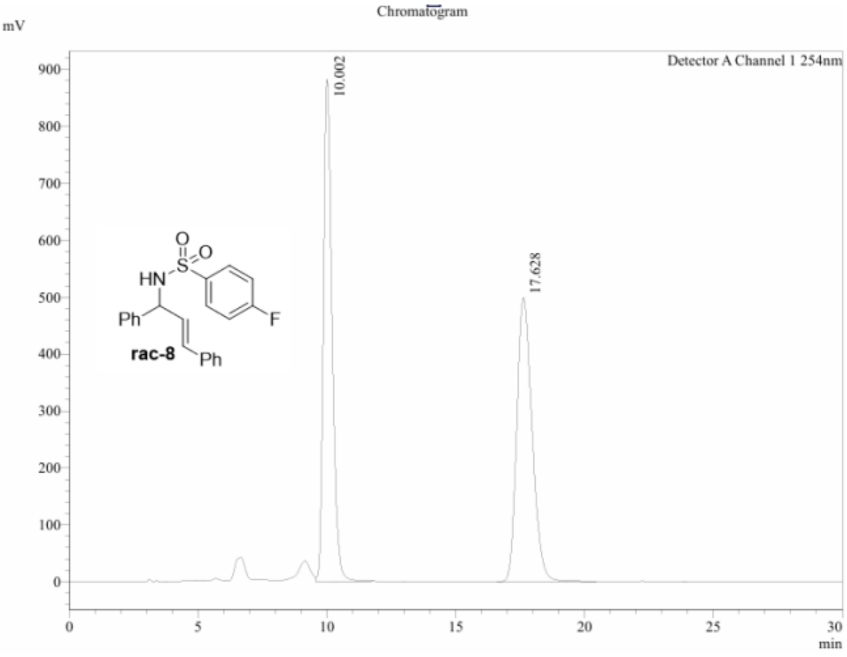

Peak Table

Detector A Channel 1 254nm

Peak#	Ret. Time	Area	Height	Area%	Height%
1	10.002	20620026	882094	50.039	63.888
2	17.628	20587586	498603	49.961	36.112
Total		41207613	1380697	100.000	100.000

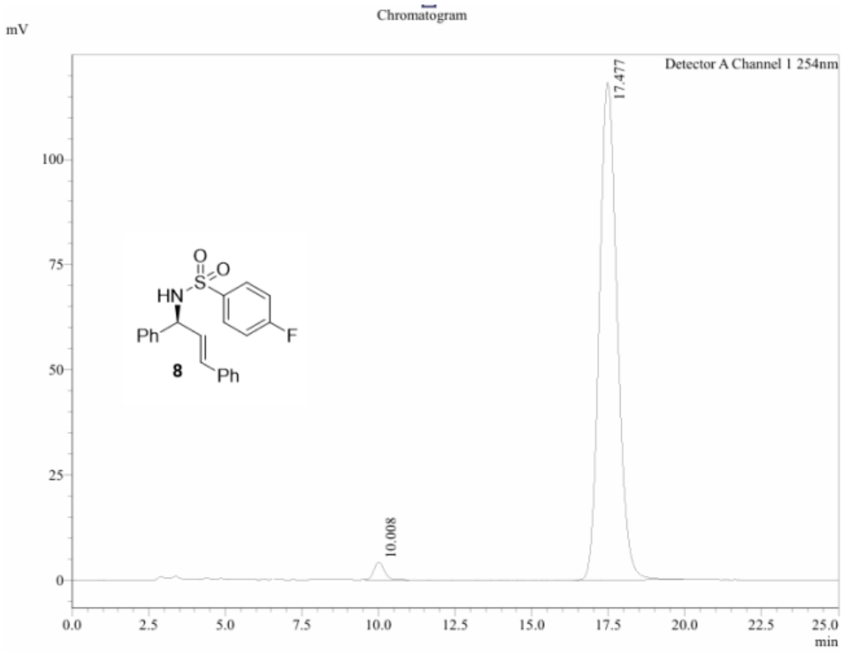

Peak Table

Detector A Channel 1 254nm

Peak#	Ret. Time	Area	Height	Area%	Height%
1	10.008	98684	4210	2.014	3.439
2	17.477	4800474	118232	97.986	96.561
Total		4899158	122443	100.000	100.000

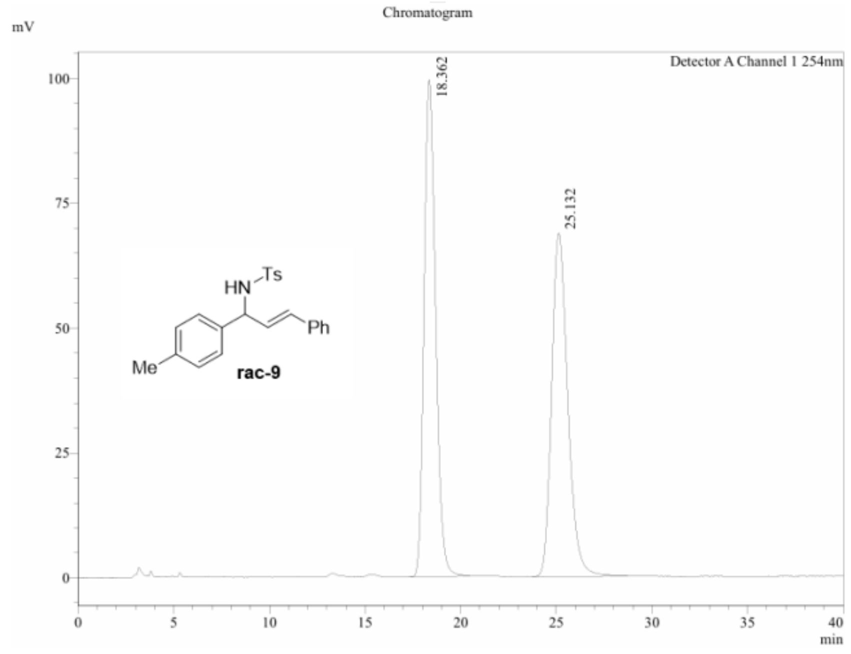

Peak Table

Detector A Channel 1 254nm

Peak#	Ret. Time	Area	Height	Area%	Height%
1	18.362	4157446	99470	50.757	59.120
2	25.132	4033496	68781	49.243	40.880
Total		8190942	168251	100.000	100.000

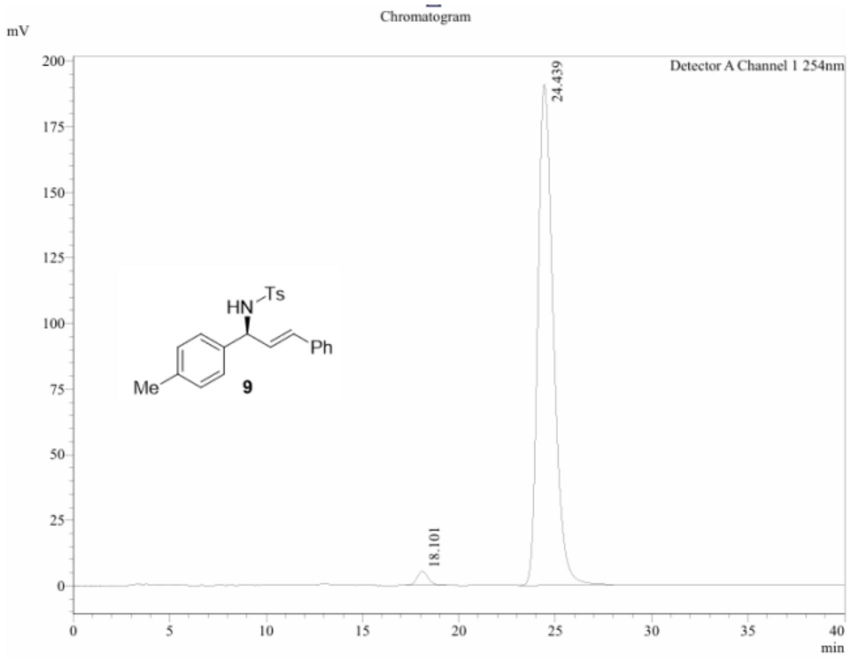

Peak Table

Detector A Channel 1 254nm

Peak#	Ret. Time	Area	Height	Area%	Height%
1	18.101	214322	5307	1.973	2.706
2	24.439	10650054	190812	98.027	97.294
Total		10864376	196119	100.000	100.000

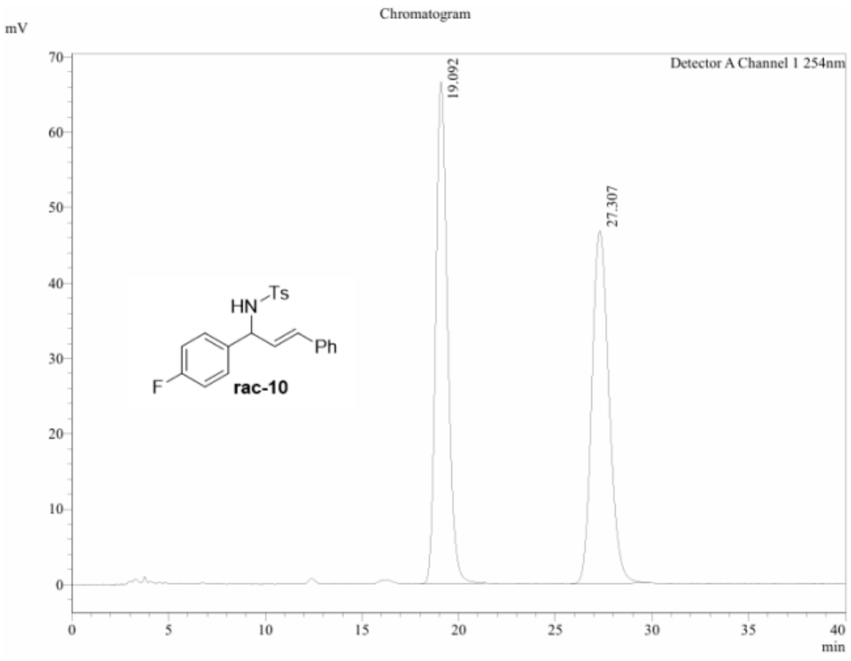

Peak Table

Detector A Channel 1 254nm

Peak#	Ret. Time	Area	Height	Area%	Height%
1	19.092	2830519	66560	49.512	58.708
2	27.307	2886322	46816	50.488	41.292
Total		5716841	113376	100.000	100.000

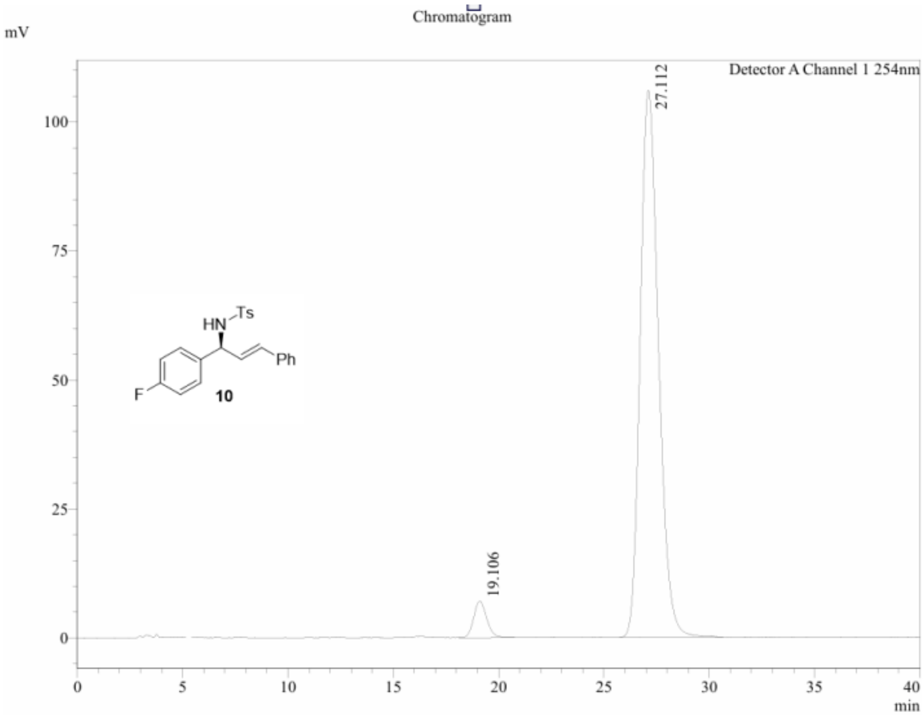

Peak Table

Detector A Channel 1 254nm

Peak#	Ret. Time	Area	Height	Area%	Height%
1	19.106	304443	7048	4.540	6.236
2	27.112	6402079	105969	95.460	93.764
Total		6706522	113017	100.000	100.000

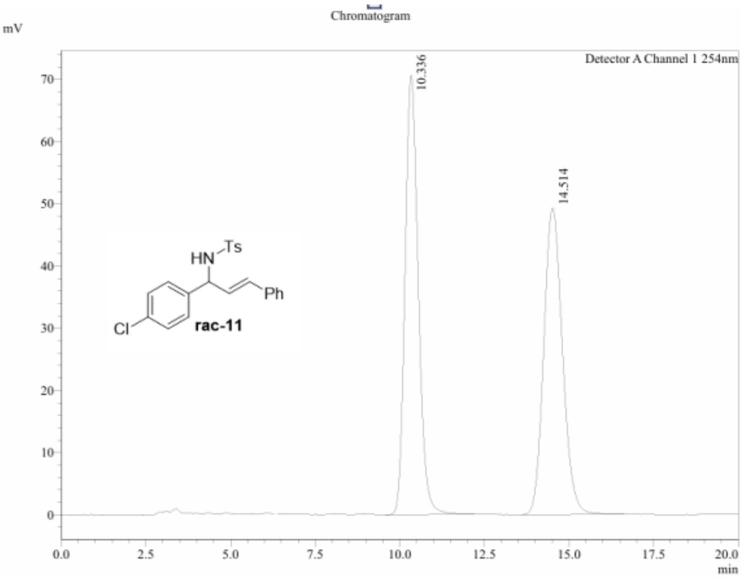

Peak Table

Detector A Channel 1 254nm

Peak#	Ret. Time	Area	Height	Area%	Height%
1	10.336	1920548	70654	50.355	58.902
2	14.514	1893483	49298	49.645	41.098
Total		3814031	119953	100.000	100.000

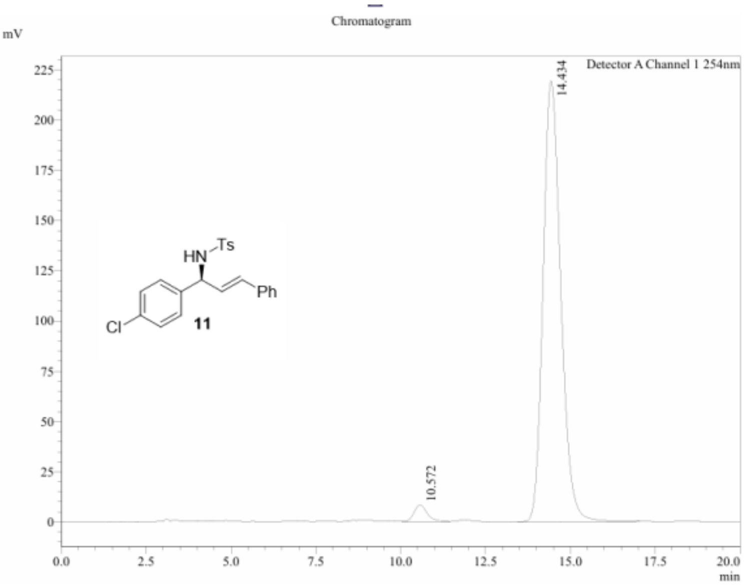

Peak Table

Detector A Channel 1 254nm

Peak#	Ret. Time	Area	Height	Area%	Height%
1	10.572	233291	8428	2.847	3.700
2	14.434	7960444	219342	97.153	96.300
Total		8193736	227770	100.000	100.000

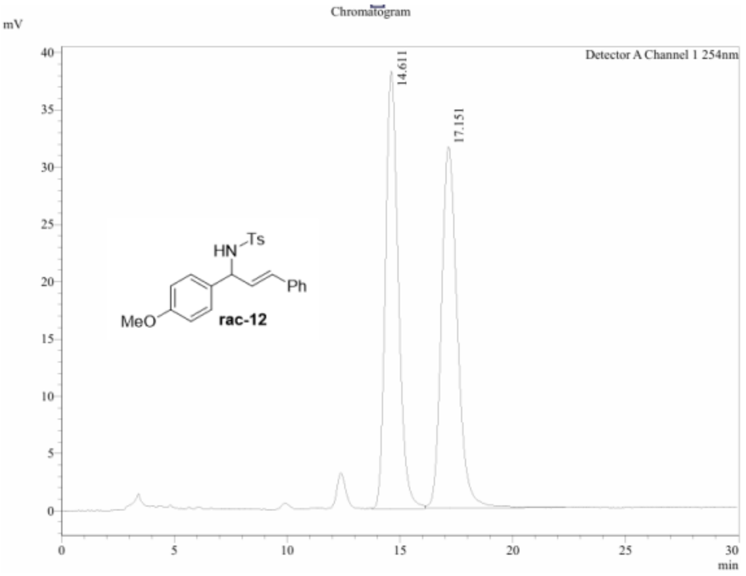

Peak Table

Detector A Channel 1 254nm

Peak#	Ret. Time	Area	Height	Area%	Height%
1	14.611	1512999	38196	49.433	54.758
2	17.151	1547708	31559	50.567	45.242
Total		3060706	69755	100.000	100.000

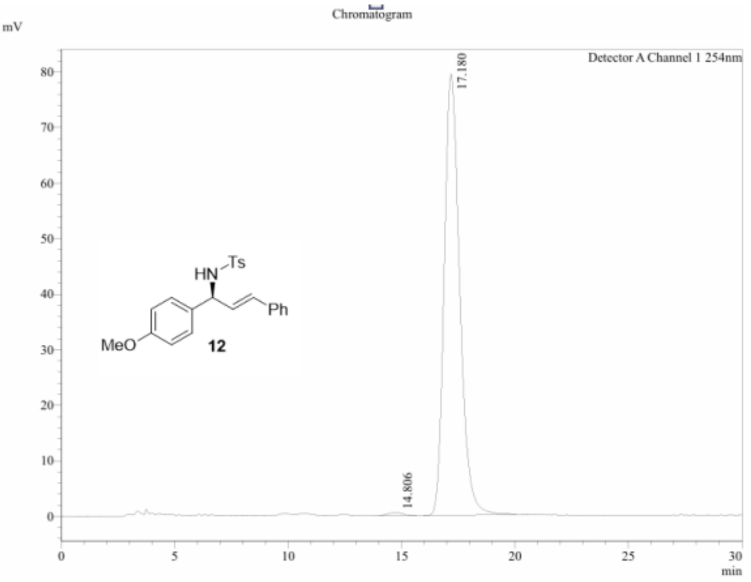

Peak Table

Detector A Channel 1 254nm

Peak#	Ret. Time	Area	Height	Area%	Height%
1	14.806	23203	557	0.611	0.697
2	17.180	3774164	79424	99.389	99.303
Total		3797367	79981	100.000	100.000

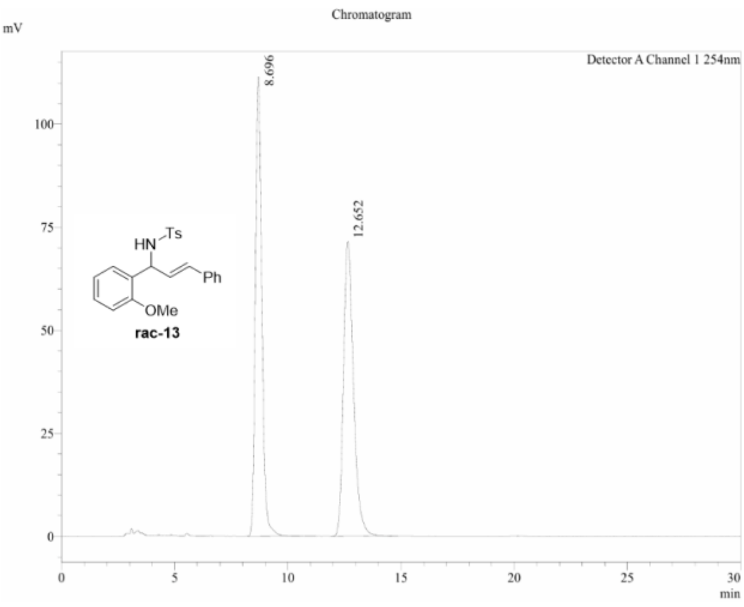

Peak Table

Detector A Channel 1 254nm

Peak#	Ret. Time	Area	Height	Area%	Height%
1	8.696	2212873	111448	50.102	60.921
2	12.652	2203882	71490	49.898	39.079
Total		4416755	182938	100.000	100.000

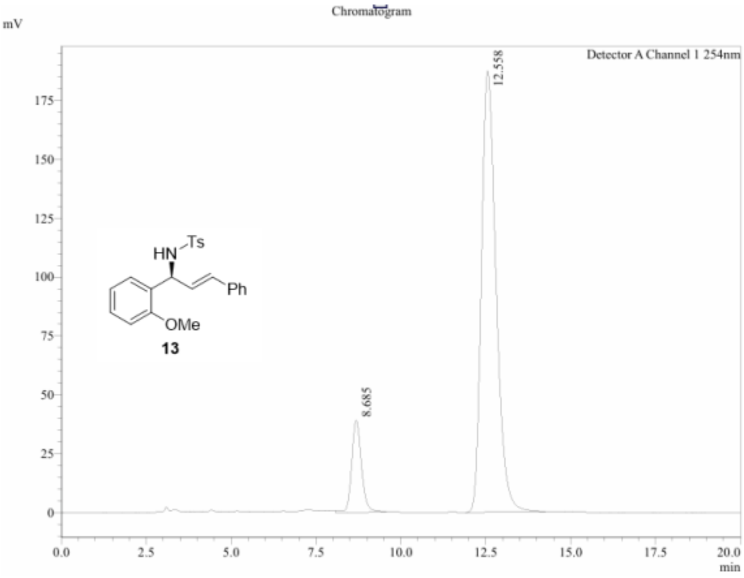

Peak Table

Detector A Channel 1 254nm

Peak#	Ret. Time	Area	Height	Area%	Height%
1	8.685	778998	39052	12.196	17.243
2	12.558	5608126	187424	87.804	82.757
Total		6387124	226476	100.000	100.000

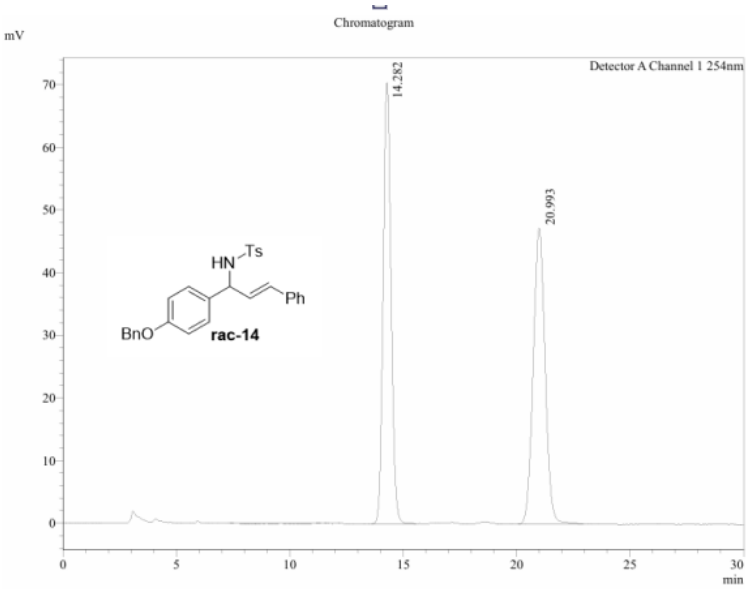

Peak Table

Detector A Channel 1 254nm

Peak#	Ret. Time	Area	Height	Area%	Height%
1	14.282	1705663	70432	49.742	59.862
2	20.993	1723346	47226	50.258	40.138
Total		3429009	117658	100.000	100.000

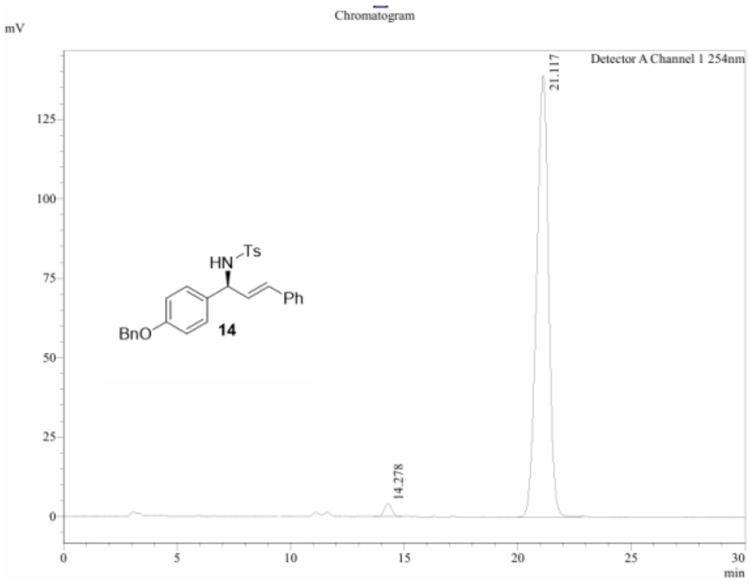

Peak Table

Detector A Channel 1 254nm

Peak#	Ret. Time	Area	Height	Area%	Height%
1	14.278	100683	4154	1.940	2.902
2	21.117	5090253	138985	98.060	97.098
Total		5190936	143139	100.000	100.000

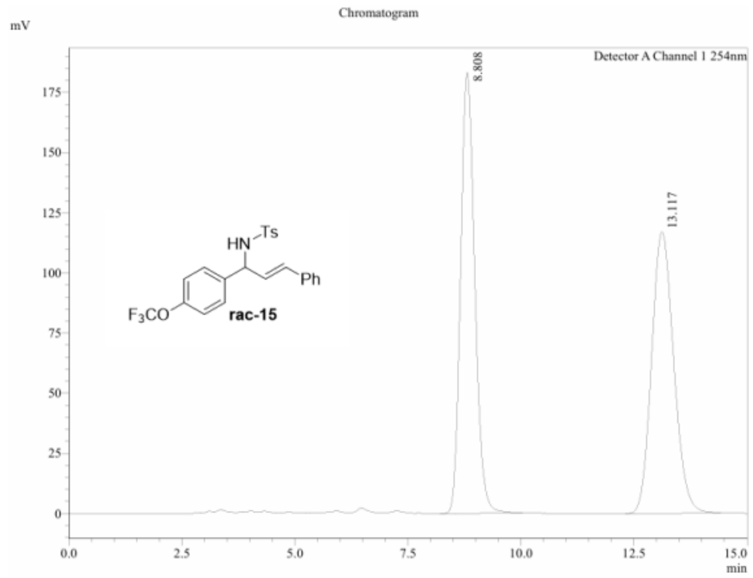

Peak Table

Detector A Channel 1 254nm

Peak#	Ret. Time	Area	Height	Area%	Height%
1	8.808	3994693	183229	50.078	61.015
2	13.117	3982195	117070	49.922	38.985
Total		7976889	300299	100.000	100.000

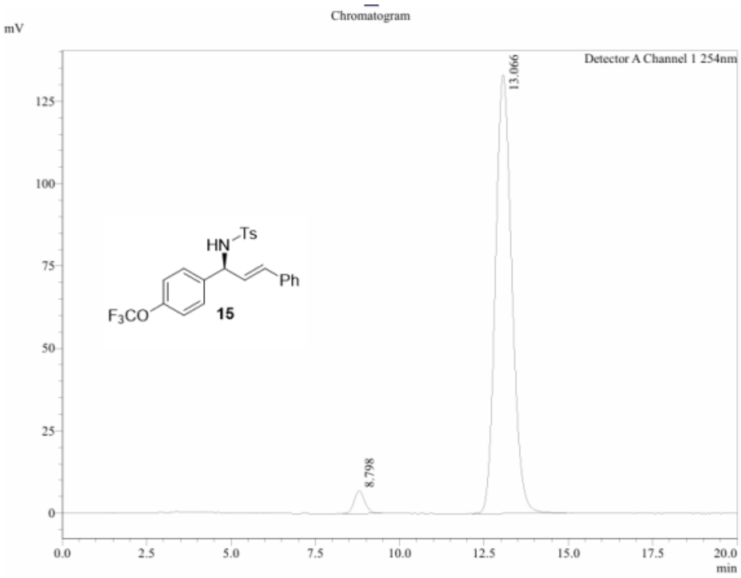

Peak Table

Detector A Channel 1 254nm

Peak#	Ret. Time	Area	Height	Area%	Height%
1	8.798	151929	6884	3.257	4.918
2	13.066	4512481	133072	96.743	95.082
Total		4664409	139955	100.000	100.000

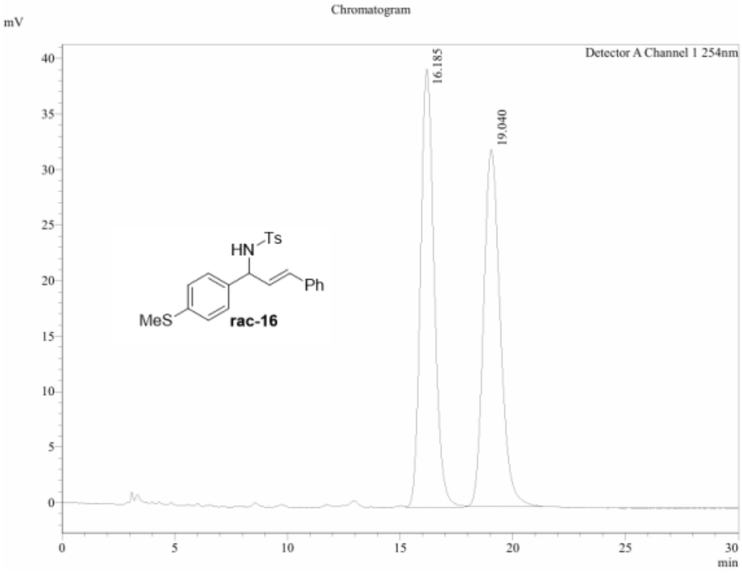

Peak Table

Detector A Channel 1 254nm

Peak#	Ret. Time	Area	Height	Area%	Height%
1	16.185	1650924	39453	49.997	55.076
2	19.040	1651148	32180	50.003	44.924
Total		3302072	71633	100.000	100.000

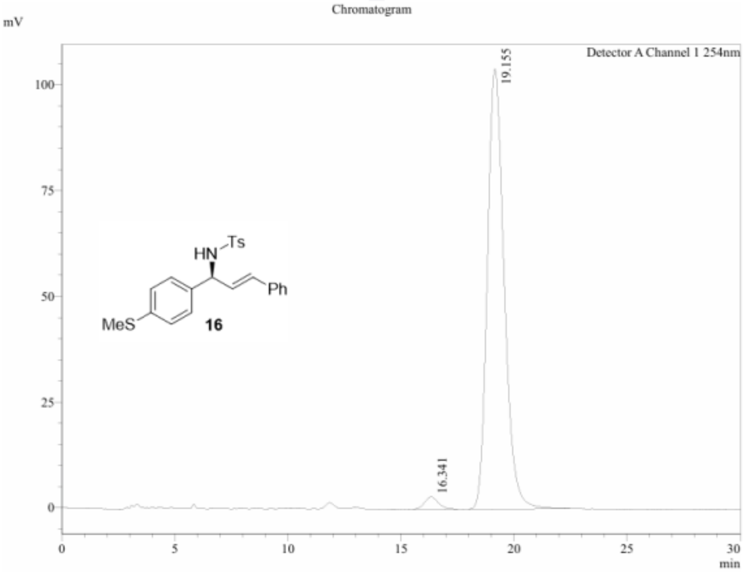

Peak Table

Detector A Channel 1 254nm

Peak#	Ret. Time	Area	Height	Area%	Height%
1	16.341	123893	2901	2.258	2.714
2	19.155	5362895	104006	97.742	97.286
Total		5486788	106907	100.000	100.000

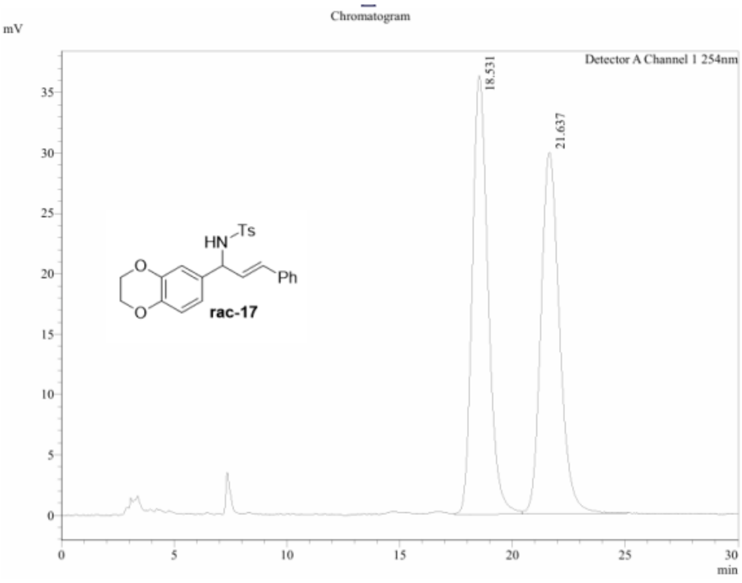

Peak Table

Detector A Channel 1 254nm

Peak#	Ret. Time	Area	Height	Area%	Height%
1	18.531	1772269	36320	50.874	54.827
2	21.637	1711354	29924	49.126	45.173
Total		3483623	66244	100.000	100.000

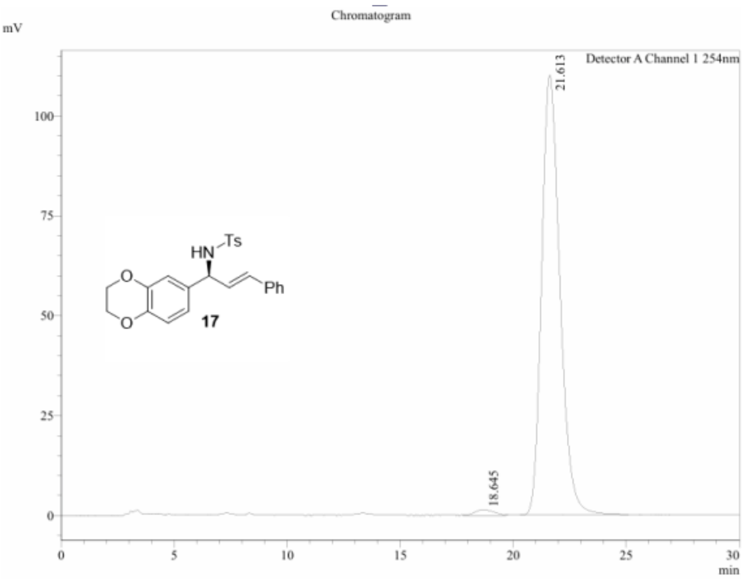

Peak Table

Detector A Channel 1 254nm

Peak#	Ret. Time	Area	Height	Area%	Height%
1	18.645	79573	1368	1.269	1.228
2	21.613	6192058	110035	98.731	98.772
Total		6271632	111402	100.000	100.000

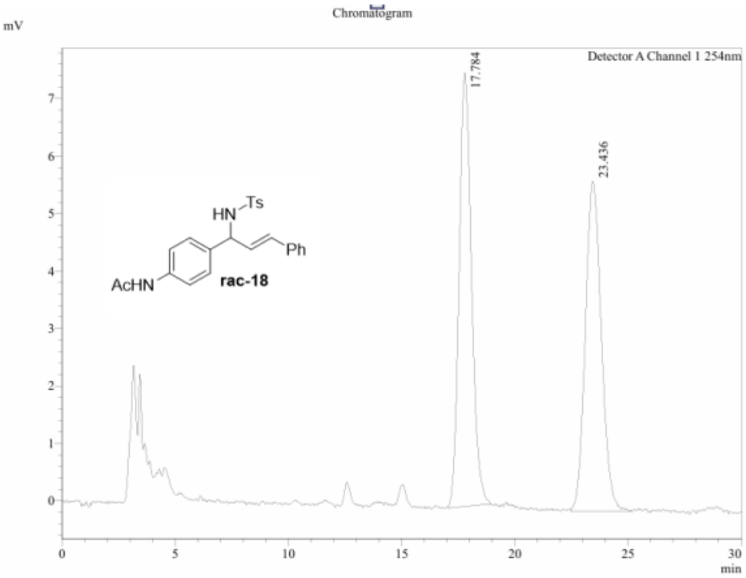

Peak Table

Detector A Channel 1 254nm

Peak#	Ret. Time	Area	Height	Area%	Height%
1	17.784	286189	7542	50.361	56.762
2	23.436	282083	5745	49.639	43.238
Total		568272	13288	100.000	100.000

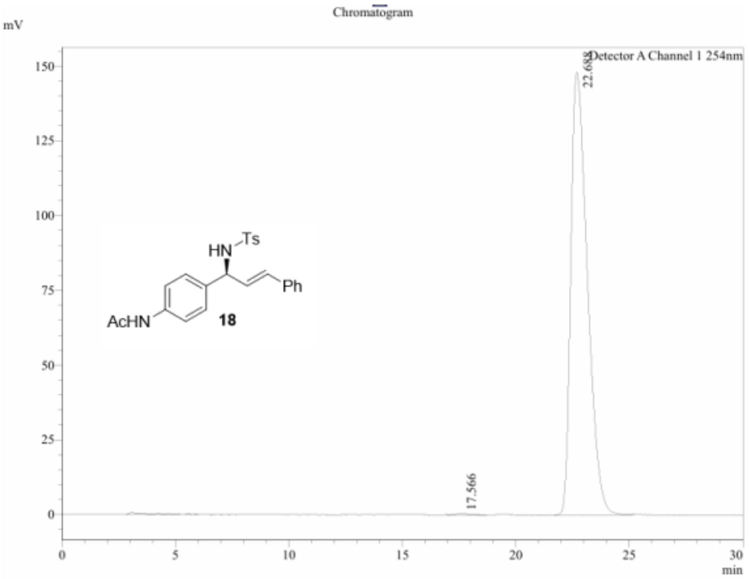

Peak Table

Detector A Channel 1 254nm

Peak#	Ret. Time	Area	Height	Area%	Height%
1	17.566	19816	476	0.263	0.321
2	22.688	7526854	147987	99.737	99.679
Total		7546671	148463	100.000	100.000

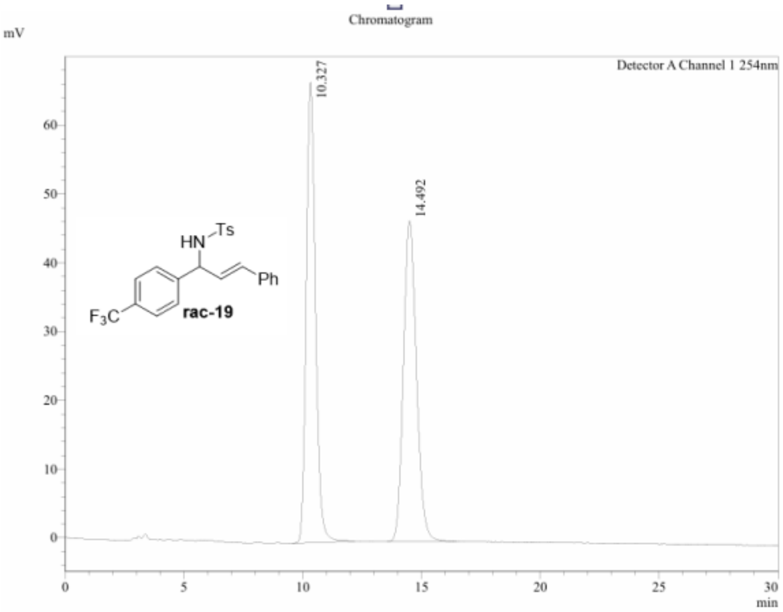

Peak Table

Detector A Channel 1 254nm

Peak#	Ret. Time	Area	Height	Area%	Height%
1	10.327	1807679	66932	50.332	58.937
2	14.492	1783855	46633	49.668	41.063
Total		3591534	113565	100.000	100.000

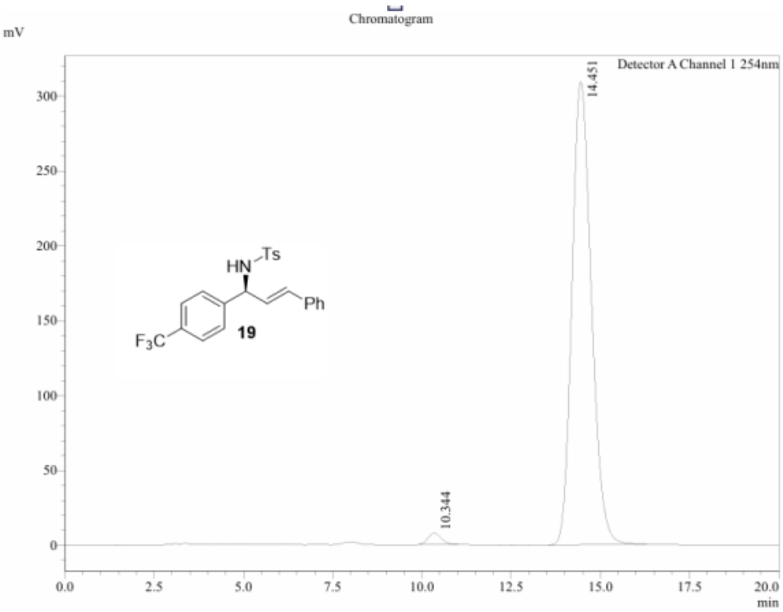

Peak Table

Detector A Channel 1 254nm

Peak#	Ret. Time	Area	Height	Area%	Height%
1	10.344	197372	7700	1.657	2.429
2	14.451	11714603	309341	98.343	97.571
Total		11911975	317040	100.000	100.000

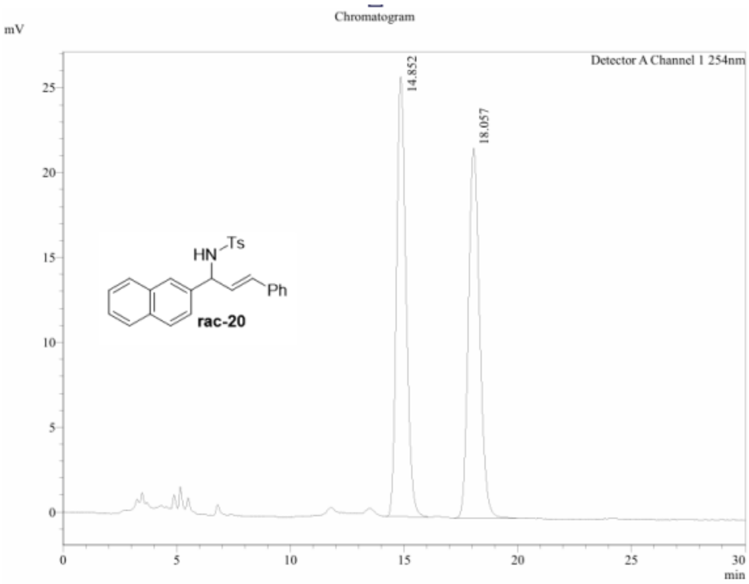

Peak Table

Detector A Channel 1 254nm

Peak#	Ret. Time	Area	Height	Area%	Height%
1	14.852	753869	25935	50.087	54.310
2	18.057	751241	21818	49.913	45.690
Total		1505110	47753	100.000	100.000

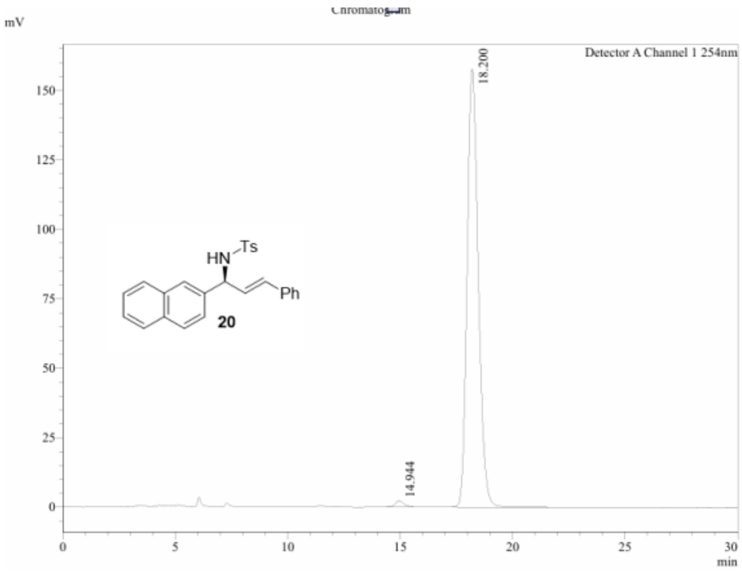

Peak Table

Detector A Channel 1 254nm

Peak#	Ret. Time	Area	Height	Area%	Height%
1	14.944	66151	2319	1.201	1.450
2	18.200	5441220	157663	98.799	98.550
Total		5507371	159982	100.000	100.000

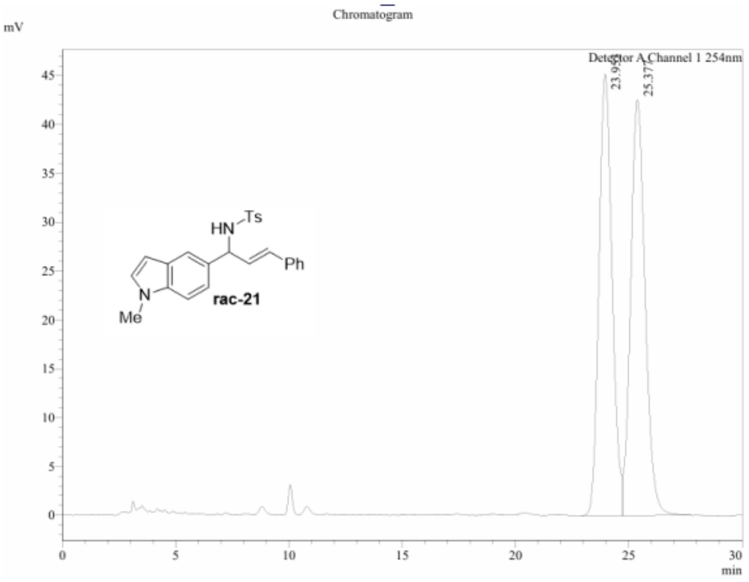

Peak Table

Detector A Channel 1 254nm

Peak#	Ret. Time	Area	Height	Area%	Height%
1	23.953	1821965	45173	49.015	51.501
2	25.377	1895225	42540	50.985	48.499
Total		3717190	87713	100.000	100.000

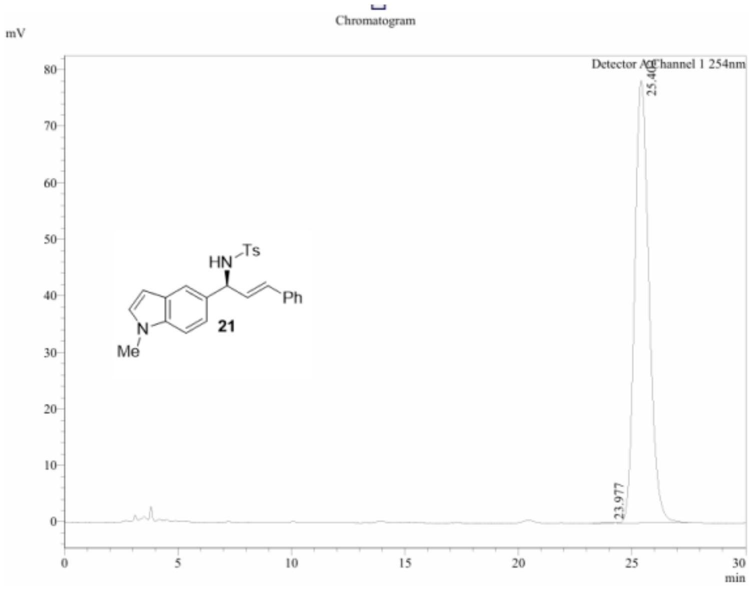

Peak Table

Detector A Channel 1 254nm

Peak#	Ret. Time	Area	Height	Area%	Height%
1	23.977	1193	54	0.035	0.069
2	25.402	3448412	78234	99.965	99.931
Total		3449606	78289	100.000	100.000

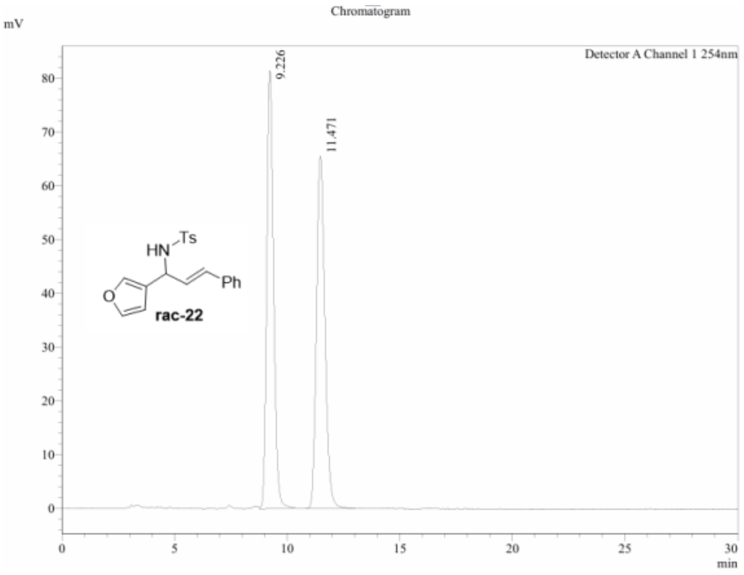

Peak Table

Detector A Channel 1 254nm

Peak#	Ret. Time	Area	Height	Area%	Height%
1	9.226	1716339	81487	50.188	55.425
2	11.471	1703464	65534	49.812	44.575
Total		3419803	147020	100.000	100.000

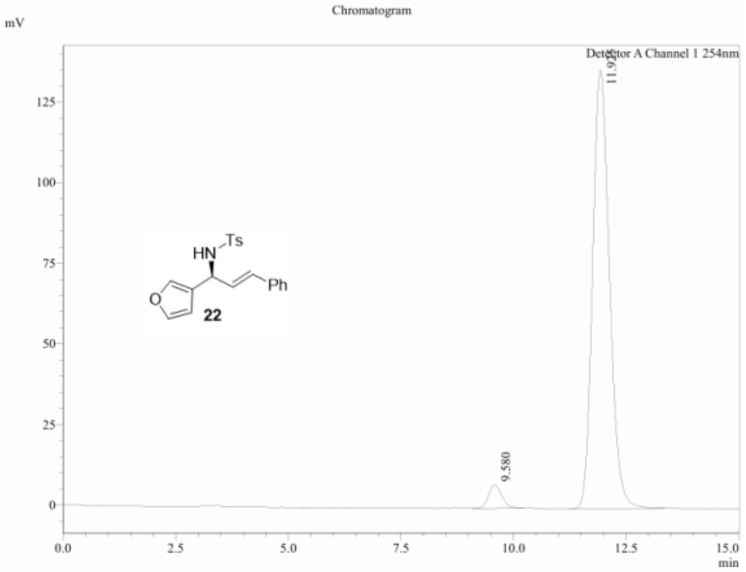

Peak Table

Detector A Channel 1 254nm

Peak#	Ret. Time	Area	Height	Area%	Height%
1	9.580	147145	7211	3.998	5.031
2	11.925	3533620	136109	96.002	94.969
Total		3680764	143319	100.000	100.000

Chromatogram

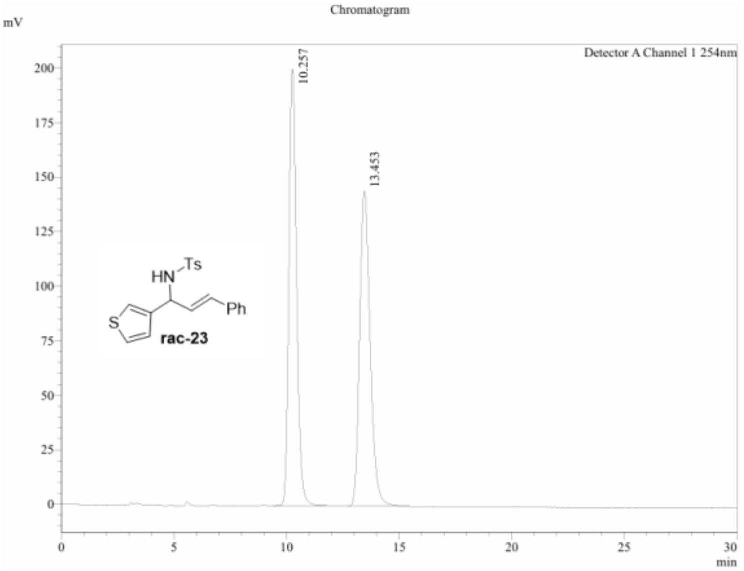

Peak Table

Detector A Channel 1 254nm

Peak#	Ret. Time	Area	Height	Area%	Height%
1	10.257	4748887	200259	50.214	58.060
2	13.453	4708438	144658	49.786	41.940
Total		9457325	344917	100.000	100.000

Chromatogram

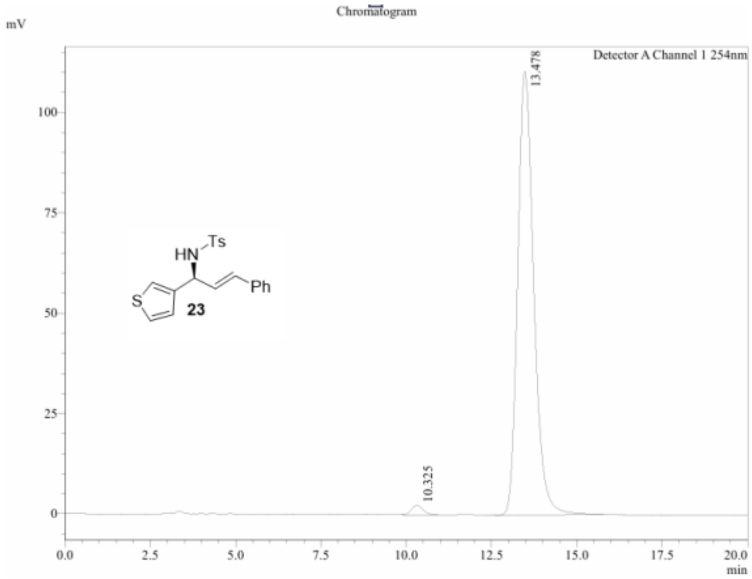

Peak Table

Detector A Channel 1 254nm

Peak#	Ret. Time	Area	Height	Area%	Height%
1	10.325	54766	2294	1.489	2.034
2	13.478	3622592	110529	98.511	97.966
Total		3677358	112823	100.000	100.000

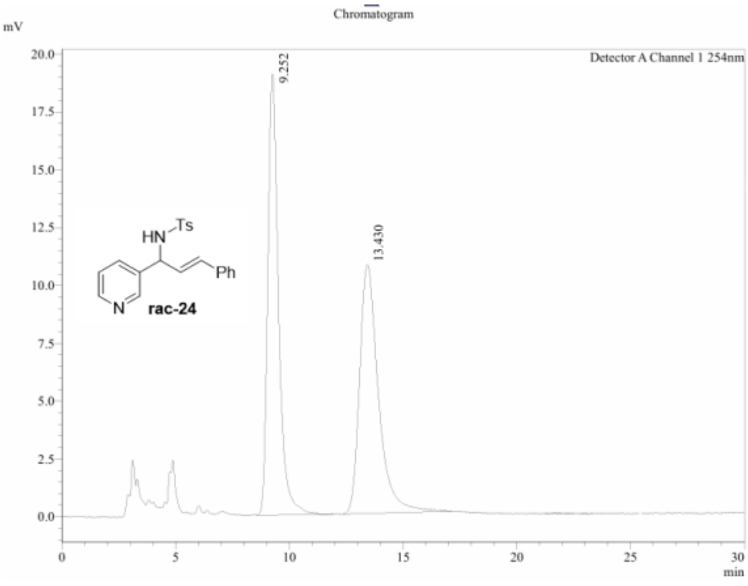

Peak Table

Detector A Channel 1 254nm

Peak#	Ret. Time	Area	Height	Area%	Height%
1	9.252	624817	19069	50.282	63.939
2	13.430	617821	10755	49.718	36.061
Total		1242638	29824	100.000	100.000

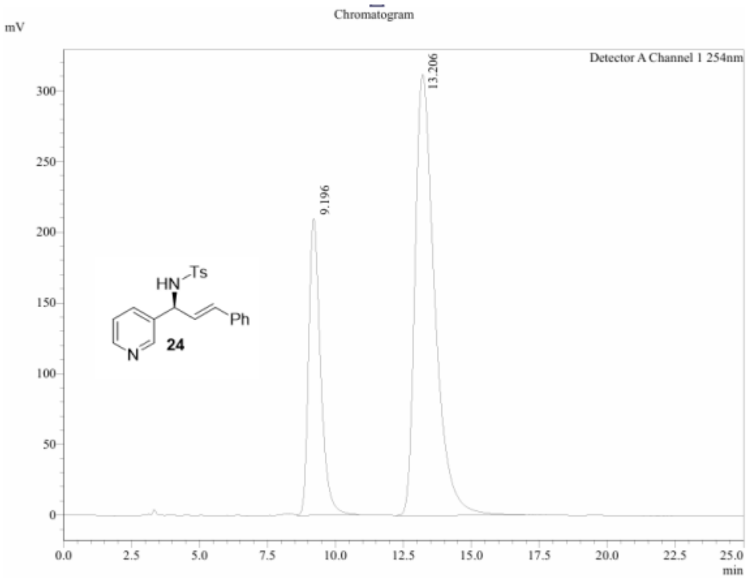

Peak Table

Detector A Channel 1 254nm

Peak#	Ret. Time	Area	Height	Area%	Height%
1	9.196	6404985	209874	28.982	40.232
2	13.206	15694663	311782	71.018	59.768
Total		22099648	521656	100.000	100.000

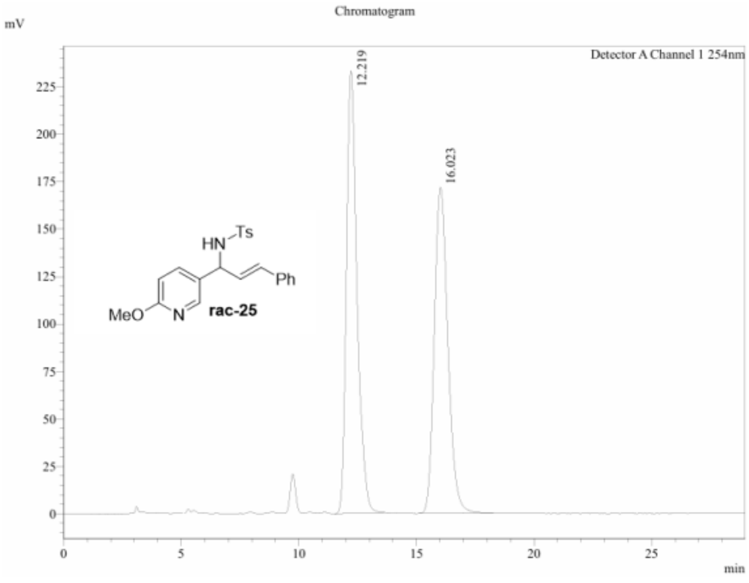

Peak Table

Detector A Channel 1 254nm

Peak#	Ret. Time	Area	Height	Area%	Height%
1	12.219	7244419	232993	51.150	57.571
2	16.023	6918571	171713	48.850	42.429
Total		14162990	404707	100.000	100.000

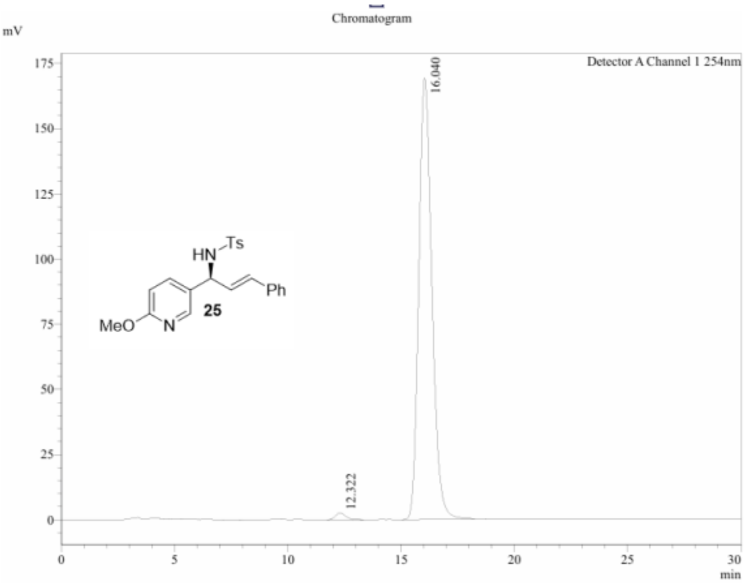

Peak Table

Detector A Channel 1 254nm

Peak#	Ret. Time	Area	Height	Area%	Height%
1	12.322	83383	2629	1.207	1.529
2	16.040	6826259	169308	98.793	98.471
Total		6909642	171937	100.000	100.000

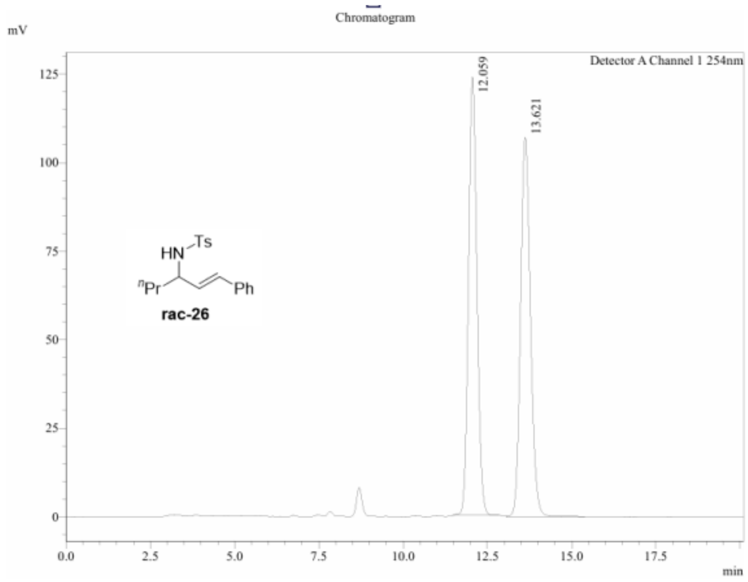

Peak Table

Detector A Channel 1 254nm

Peak#	Ret. Time	Area	Height	Area%	Height%
1	12.059	2167013	123787	49.818	53.611
2	13.621	2182838	107112	50.182	46.389
Total		4349851	230900	100.000	100.000

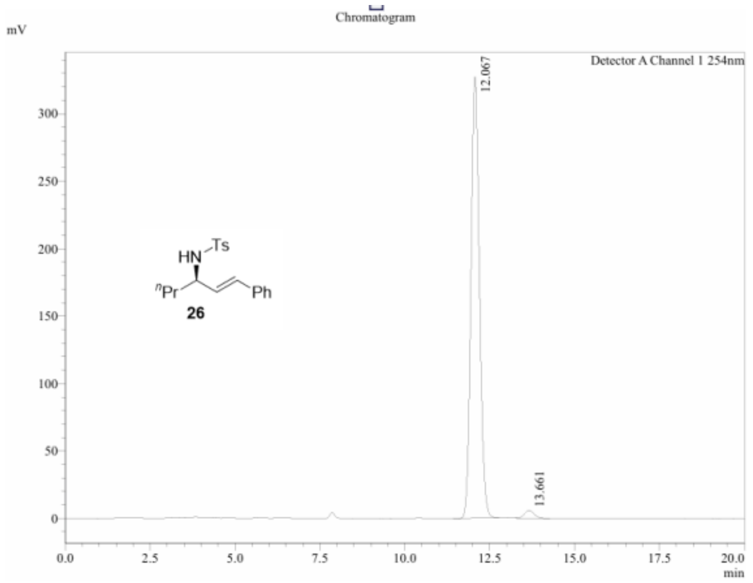

Peak Table

Detector A Channel 1 254nm

Peak#	Ret. Time	Area	Height	Area%	Height%
1	12.067	5703721	326945	98.042	98.264
2	13.661	113932	5777	1.958	1.736
Total		5817652	332722	100.000	100.000

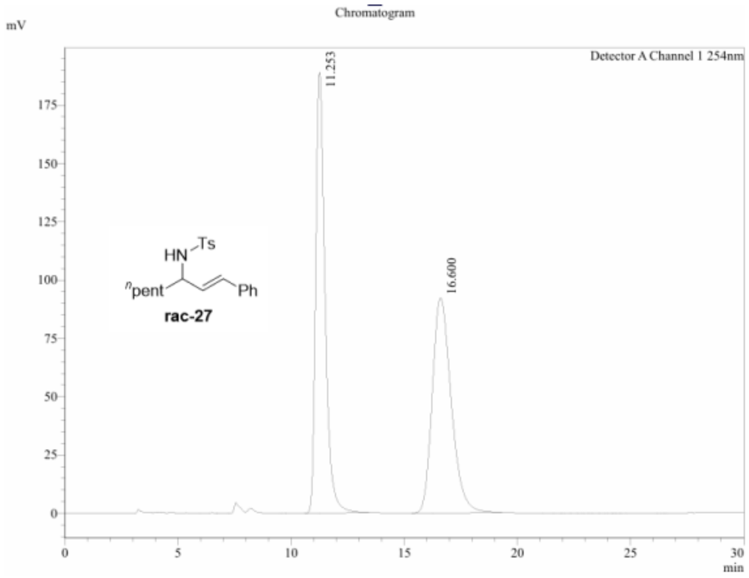

Peak Table

Detector A Channel 1 254nm

Peak#	Ret. Time	Area	Height	Area%	Height%
1	11.253	5403980	189019	50.093	67.184
2	16.600	5383852	92324	49.907	32.816
Total		10787832	281343	100.000	100.000

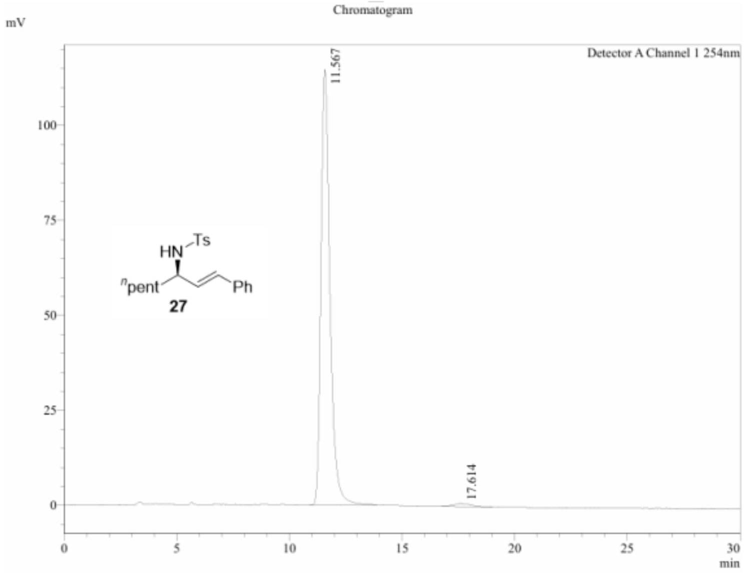

Peak Table

Detector A Channel 1 254nm

Peak#	Ret. Time	Area	Height	Area%	Height%
1	11.567	3193429	114687	98.688	99.309
2	17.614	42453	798	1.312	0.691
Total		3235881	115486	100.000	100.000

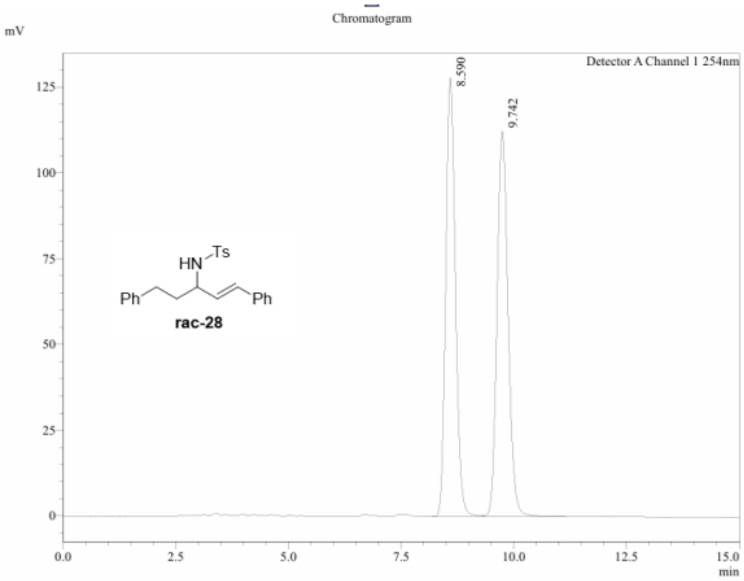

Peak Table

Detector A Channel 1 254nm

Peak#	Ret. Time	Area	Height	Area%	Height%
1	8.590	1906148	127869	49.856	53.234
2	9.742	1917161	112333	50.144	46.766
Total		3823310	240202	100.000	100.000

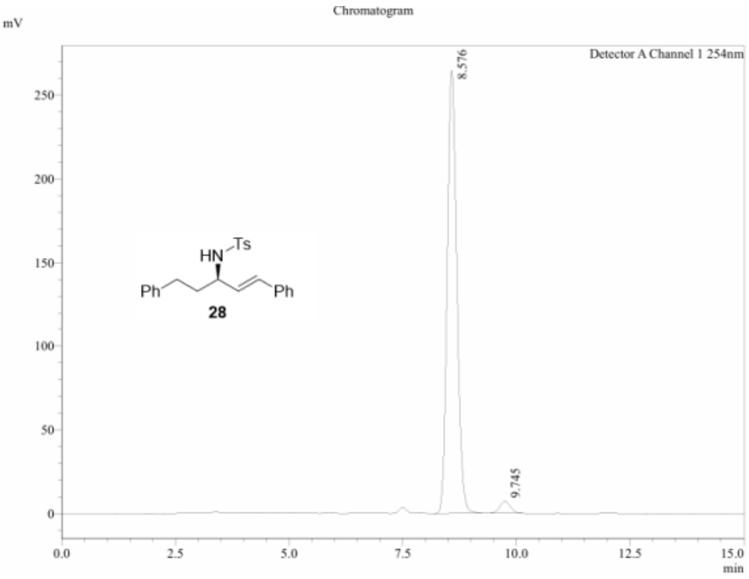

Peak Table

Detector A Channel 1 254nm

Peak#	Ret. Time	Area	Height	Area%	Height%
1	8.576	3881973	264659	97.107	97.410
2	9.745	115640	7038	2.893	2.590
Total		3997614	271697	100.000	100.000

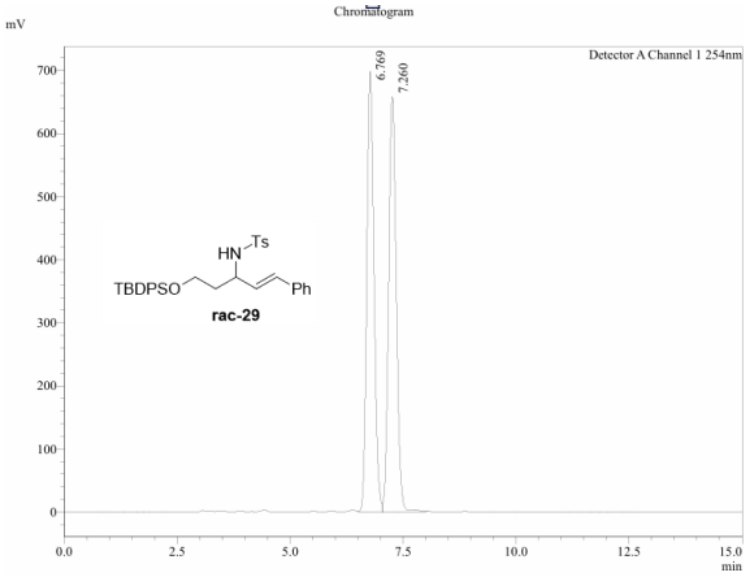

Peak Table

Detector A Channel 1 254nm

Peak#	Ret. Time	Area	Height	Area%	Height%
1	6.769	7723920	698527	49.851	51.473
2	7.260	7770187	658548	50.149	48.527
Total		15494107	1357075	100.000	100.000

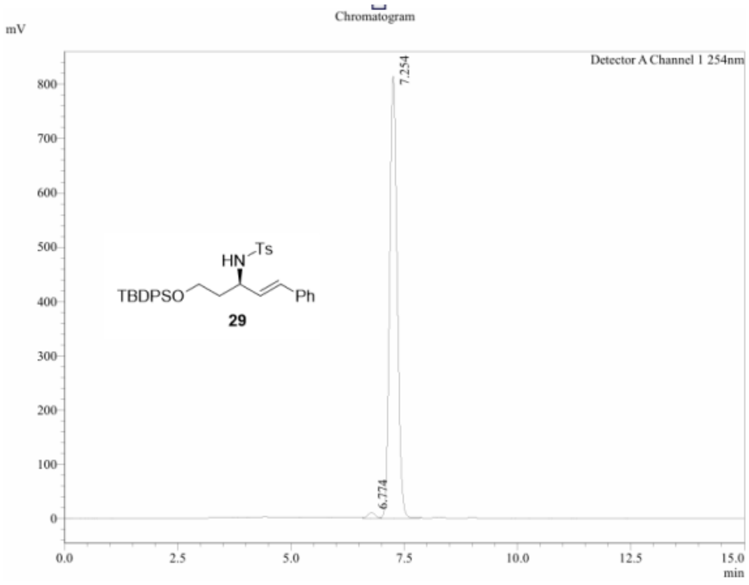

Peak Table

Detector A Channel 1 254nm

Peak#	Ret. Time	Area	Height	Area%	Height%
1	6.774	123588	10552	1.282	1.279
2	7.254	9516429	814262	98.718	98.721
Total		9640017	824814	100.000	100.000

Chromatogram

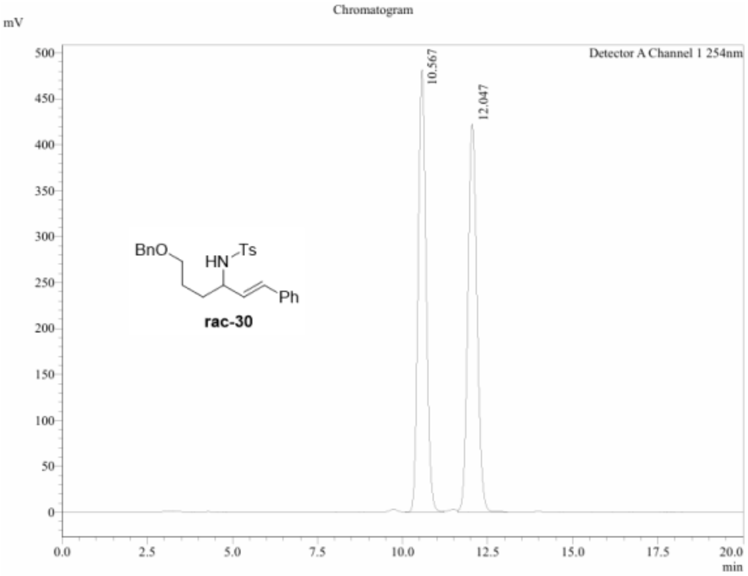

Peak Table

Detector A Channel 1 254nm

Peak#	Ret. Time	Area	Height	Area%	Height%
1	10.567	7898056	481694	49.949	53.278
2	12.047	7914158	422420	50.051	46.722
Total		15812214	904114	100.000	100.000

Chromatogram

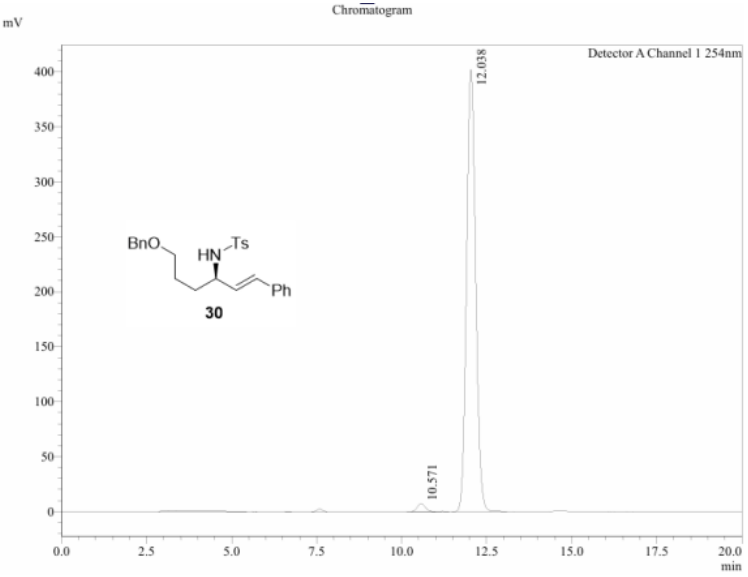

Peak Table

Detector A Channel 1 254nm

Peak#	Ret. Time	Area	Height	Area%	Height%
1	10.571	120124	7234	1.580	1.769
2	12.038	7484622	401746	98.420	98.231
Total		7604745	408980	100.000	100.000

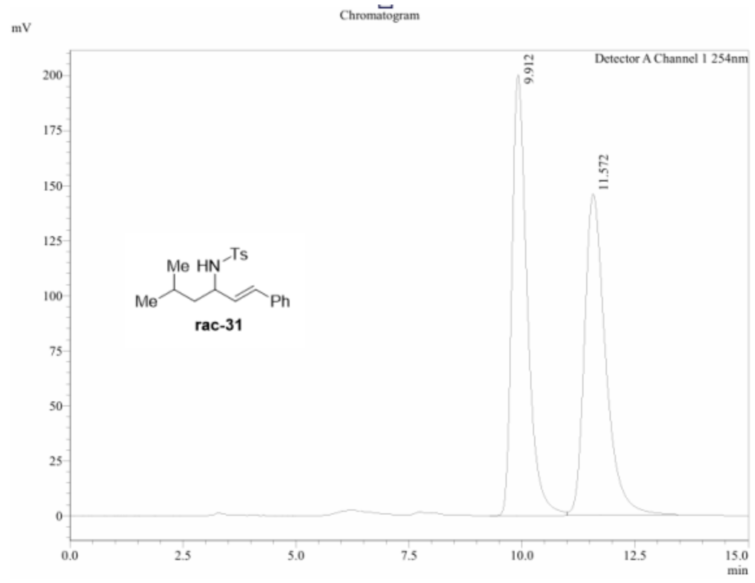

Peak Table

Detector A Channel 1 254nm

Peak#	Ret. Time	Area	Height	Area%	Height%
1	9.912	4708795	200057	49.902	57.843
2	11.572	4727324	145806	50.098	42.157
Total		9436120	345863	100.000	100.000

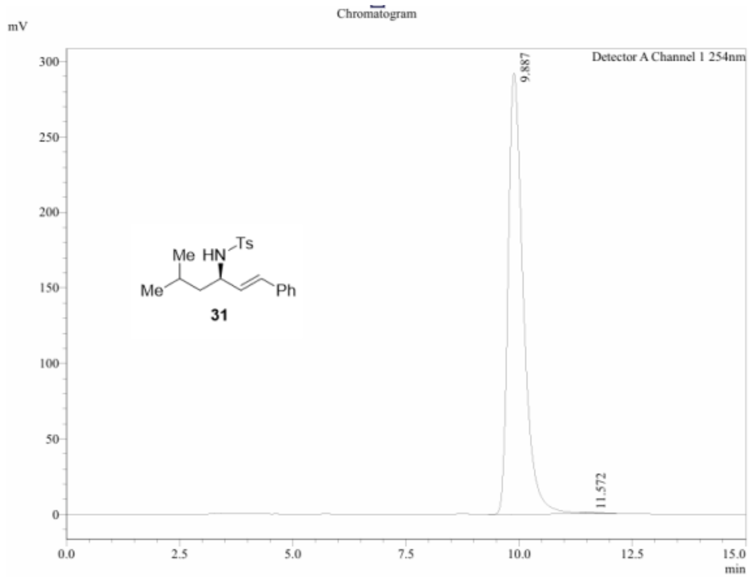

Peak Table

Detector A Channel 1 254nm

Peak#	Ret. Time	Area	Height	Area%	Height%
1	9.887	6844133	292000	99.875	99.883
2	11.572	8579	341	0.125	0.117
Total		6852713	292341	100.000	100.000

Chromatogram

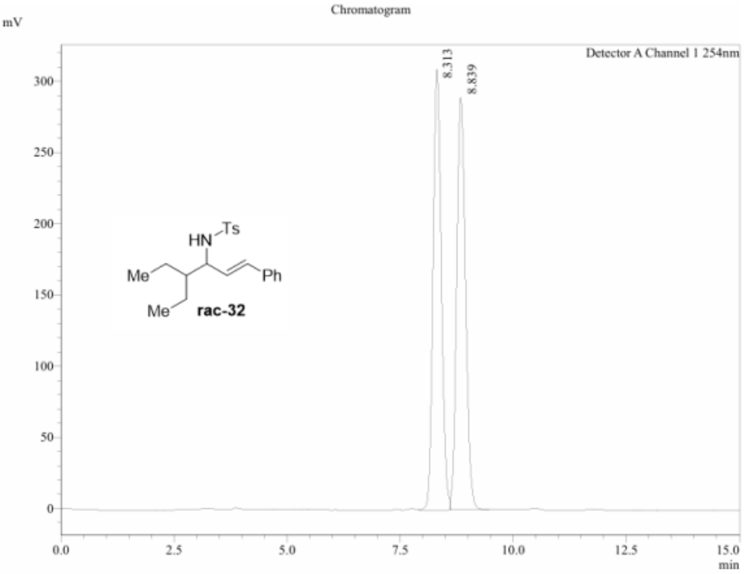

Peak Table

Detector A Channel 1 254nm

Peak#	Ret. Time	Area	Height	Area%	Height%
1	8.313	3946293	309303	50.008	51.645
2	8.839	3944991	289604	49.992	48.355
Total		7891284	598907	100.000	100.000

Chromatogram

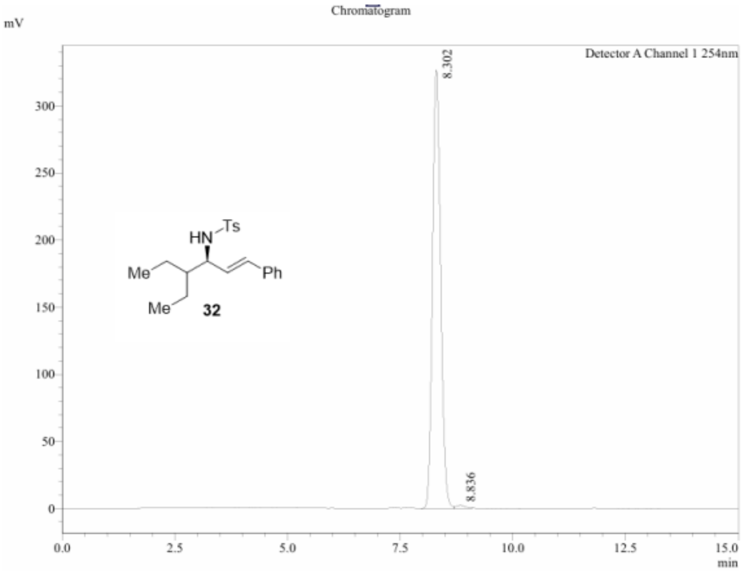

Peak Table

Detector A Channel 1 254nm

Peak#	Ret. Time	Area	Height	Area%	Height%
1	8.302	4128134	326630	99.250	99.313
2	8.836	31185	2259	0.750	0.687
Total		4159319	328888	100.000	100.000

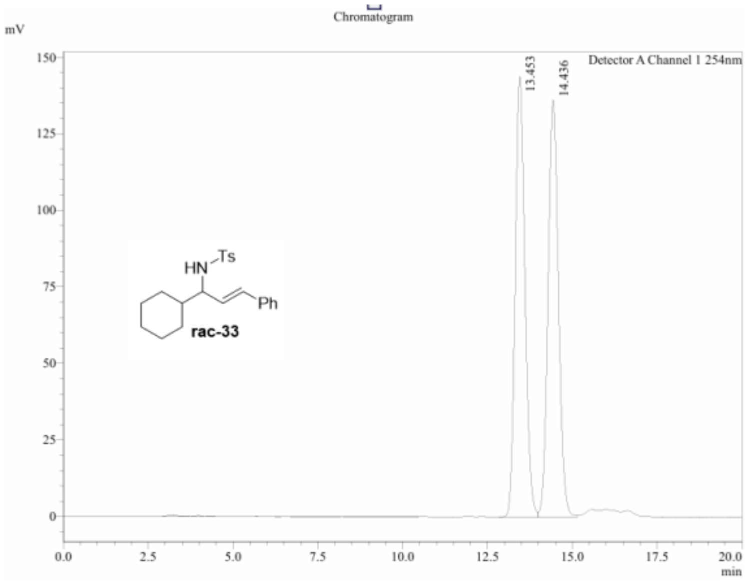

Peak Table

Detector A Channel 1 254nm

Peak#	Ret. Time	Area	Height	Area%	Height%
1	13.453	2886171	143904	49.792	51.371
2	14.436	2910302	136221	50.208	48.629
Total		5796473	280125	100.000	100.000

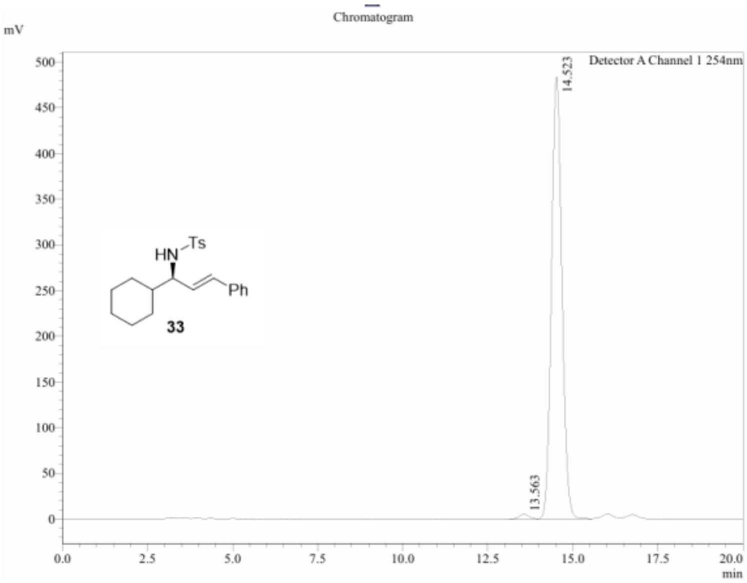

Peak Table

Detector A Channel 1 254nm

Peak#	Ret. Time	Area	Height	Area%	Height%
1	13.563	97318	5030	0.931	1.029
2	14.523	10359743	483679	99.069	98.971
Total		10457062	488709	100.000	100.000

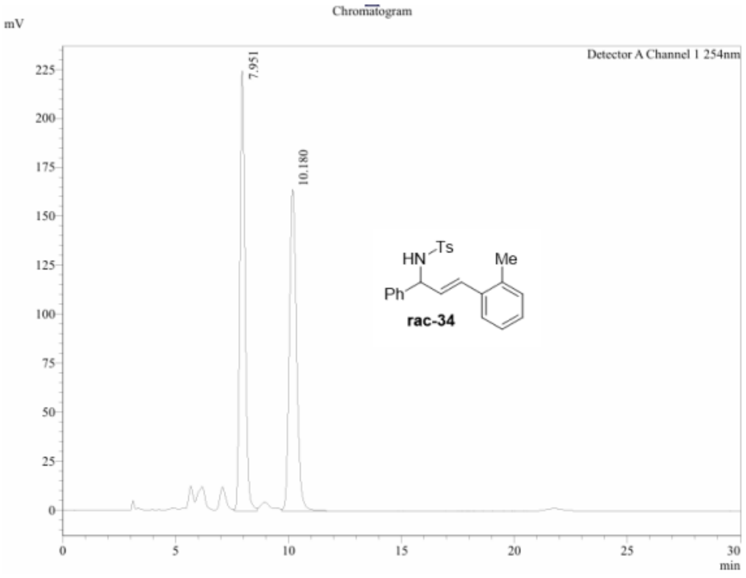

Peak Table

Detector A Channel 1 254nm

Peak#	Ret. Time	Area	Height	Area%	Height%
1	7.951	3637225	224694	50.185	57.797
2	10.180	3610467	164070	49.815	42.203
Total		7247691	388764	100.000	100.000

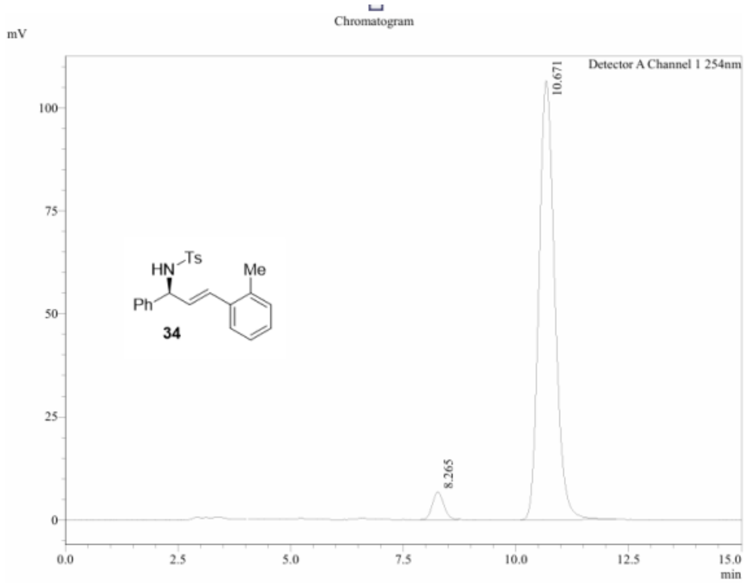

Peak Table

Detector A Channel 1 254nm

Peak#	Ret. Time	Area	Height	Area%	Height%
1	8.265	118297	6686	4.351	5.903
2	10.671	2600508	106570	95.649	94.097
Total		2718805	113256	100.000	100.000

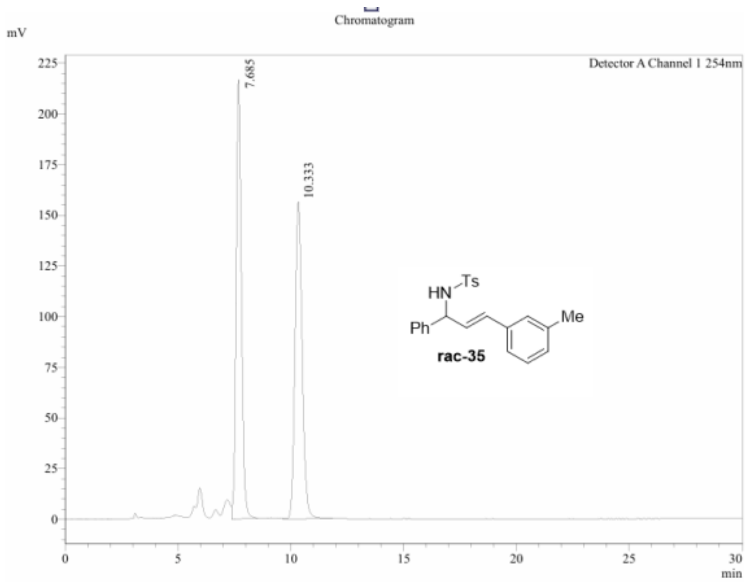

Peak Table

Detector A Channel 1 254nm

Peak#	Ret. Time	Area	Height	Area%	Height%
1	7.685	3539856	216666	50.358	58.088
2	10.333	3489530	156327	49.642	41.912
Total		7029386	372993	100.000	100.000

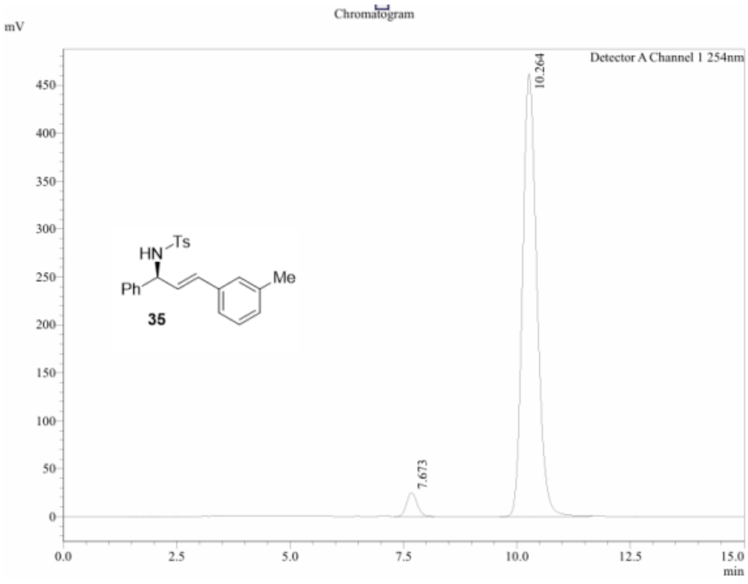

Peak Table

Detector A Channel 1 254nm

Peak#	Ret. Time	Area	Height	Area%	Height%
1	7.673	399729	24803	3.797	5.100
2	10.264	10126535	461501	96.203	94.900
Total		10526263	486304	100.000	100.000

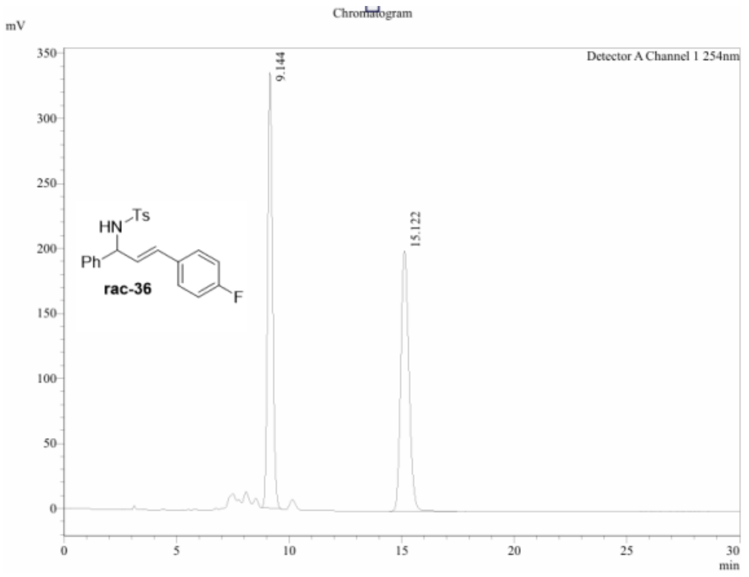

Peak Table

Detector A Channel 1 254nm

Peak#	Ret. Time	Area	Height	Area%	Height%
1	9.144	5049153	334736	50.172	62.610
2	15.122	5014619	199900	49.828	37.390
Total		10063772	534636	100.000	100.000

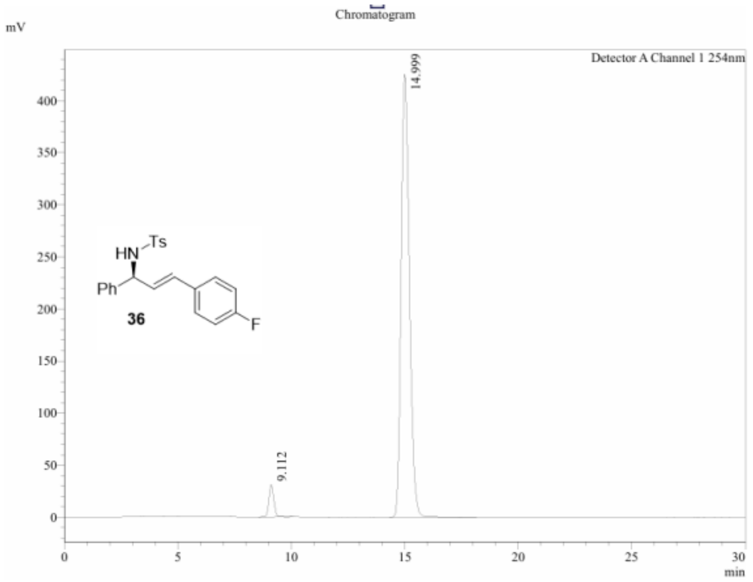

Peak Table

Detector A Channel 1 254nm

Peak#	Ret. Time	Area	Height	Area%	Height%
1	9.112	454791	30945	4.052	6.784
2	14.999	10769602	425225	95.948	93.216
Total		11224393	456170	100.000	100.000

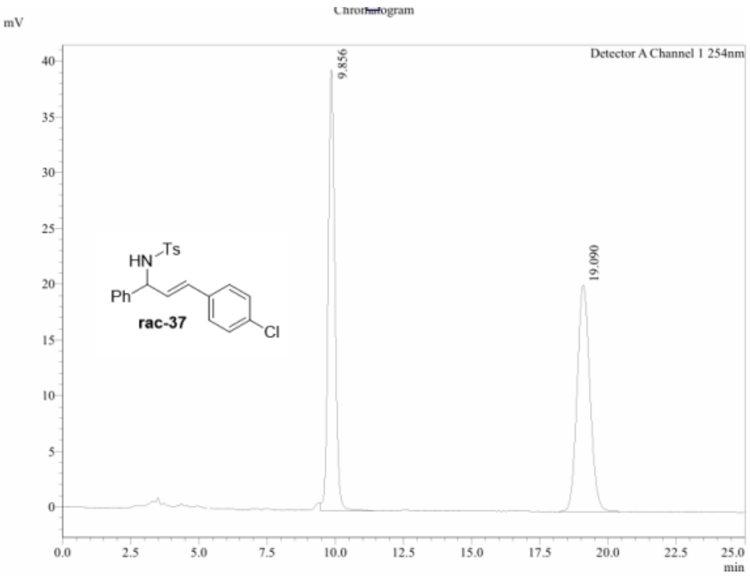

Peak Table

Detector A Channel 1 254nm

Peak#	Ret. Time	Area	Height	Area%	Height%
1	9.856	672000	39553	50.360	66.030
2	19.090	662404	20349	49.640	33.970
Total		1334404	59901	100.000	100.000

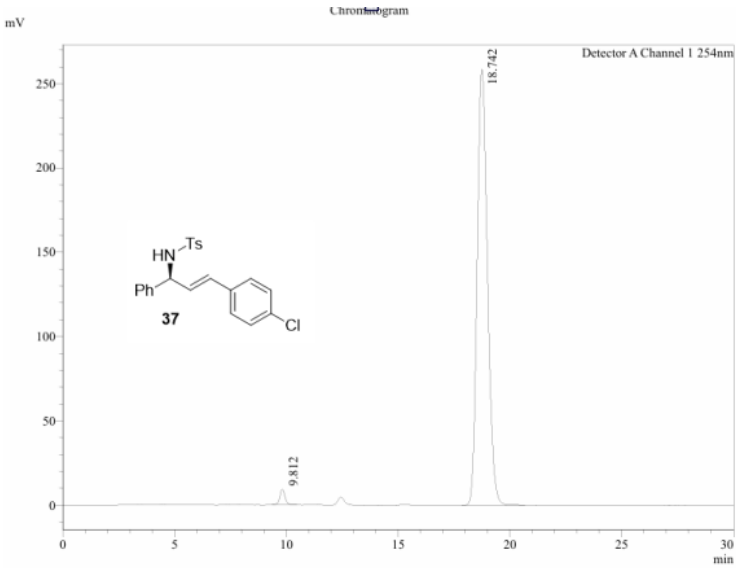

Peak Table

Detector A Channel 1 254nm

Peak#	Ret. Time	Area	Height	Area%	Height%
1	9.812	142839	8927	1.688	3.338
2	18.742	8320842	258517	98.312	96.662
Total		8463682	267444	100.000	100.000

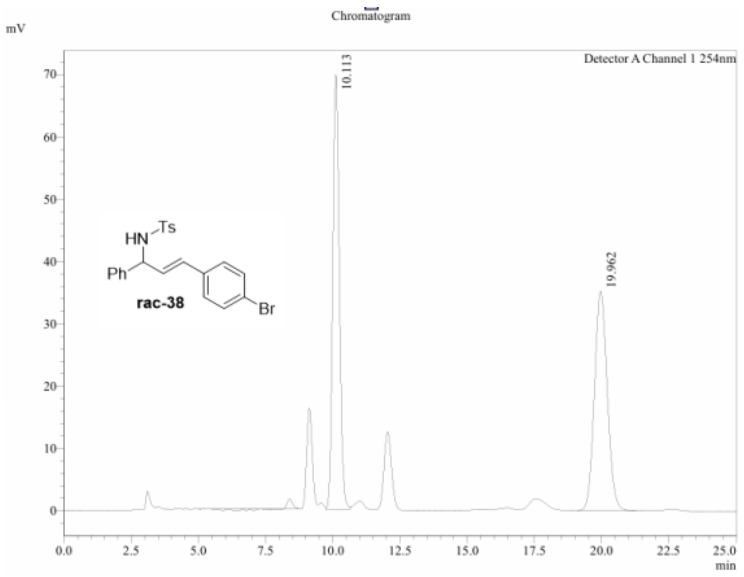

Peak Table

Detector A Channel 1 254nm

Peak#	Ret. Time	Area	Height	Area%	Height%
1	10.113	1188443	69694	50.292	66.434
2	19.962	1174649	35214	49.708	33.566
Total		2363092	104908	100.000	100.000

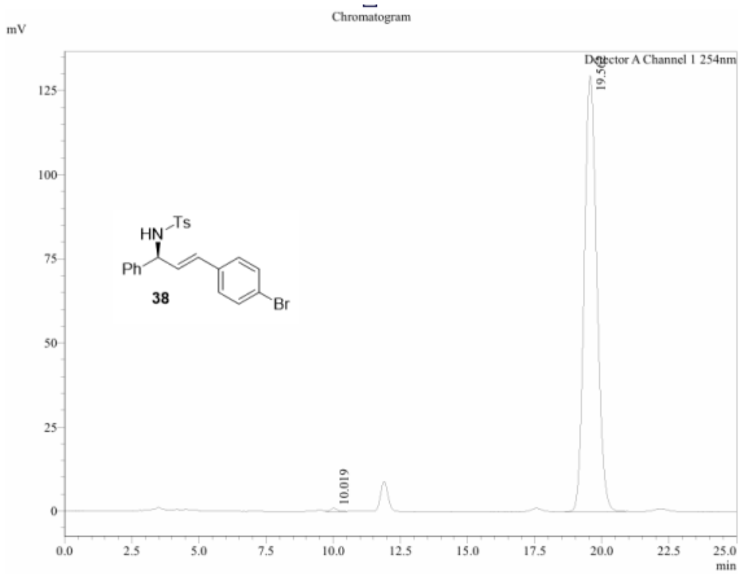

Peak Table

Detector A Channel 1 254nm

Peak#	Ret. Time	Area	Height	Area%	Height%
1	10.019	18231	1002	0.430	0.767
2	19.562	4217185	129574	99.570	99.233
Total		4235416	130575	100.000	100.000

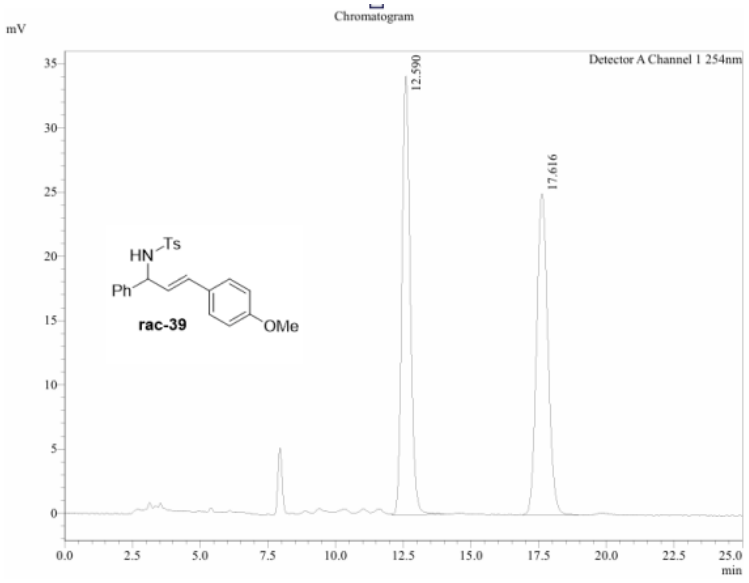

Peak Table

Detector A Channel 1 254nm

Peak#	Ret. Time	Area	Height	Area%	Height%
1	12.590	730474	34179	50.139	57.743
2	17.616	726412	25012	49.861	42.257
Total		1456886	59191	100.000	100.000

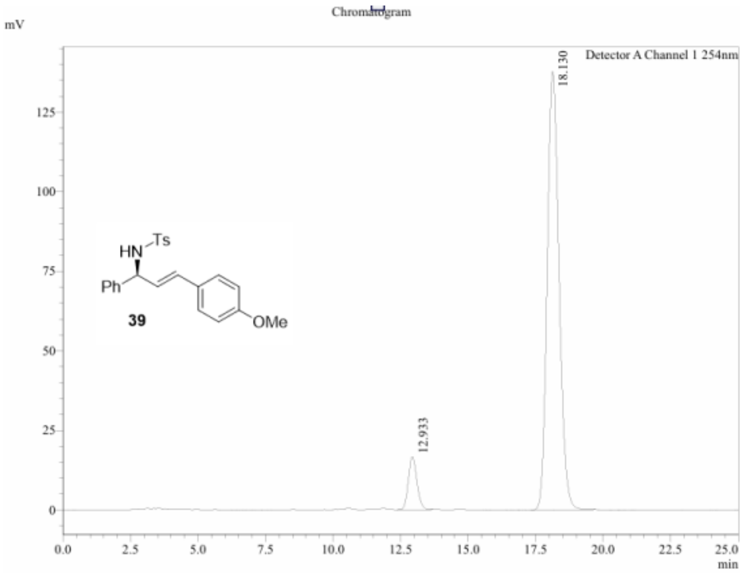

Peak Table

Detector A Channel 1 254nm

Peak#	Ret. Time	Area	Height	Area%	Height%
1	12.933	366317	16625	8.035	10.768
2	18.130	4192641	137760	91.965	89.232
Total		4558957	154384	100.000	100.000

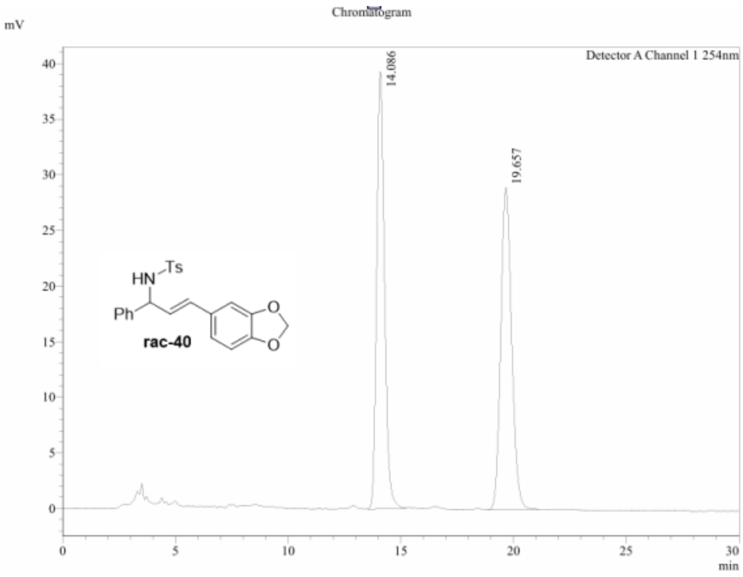

Peak Table

Detector A Channel 1 254nm

Peak#	Ret. Time	Area	Height	Area%	Height%
1	14.086	946574	39292	49.340	57.563
2	19.657	971895	28967	50.660	42.437
Total		1918469	68259	100.000	100.000

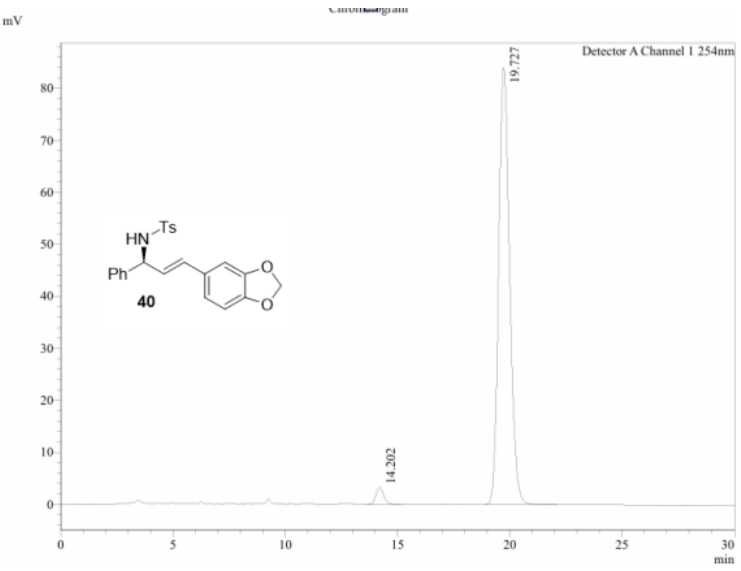

Peak Table

Detector A Channel 1 254nm

Peak#	Ret. Time	Area	Height	Area%	Height%
1	14.202	82373	3298	2.794	3.772
2	19.727	2865656	84118	97.206	96.228
Total		2948029	87415	100.000	100.000

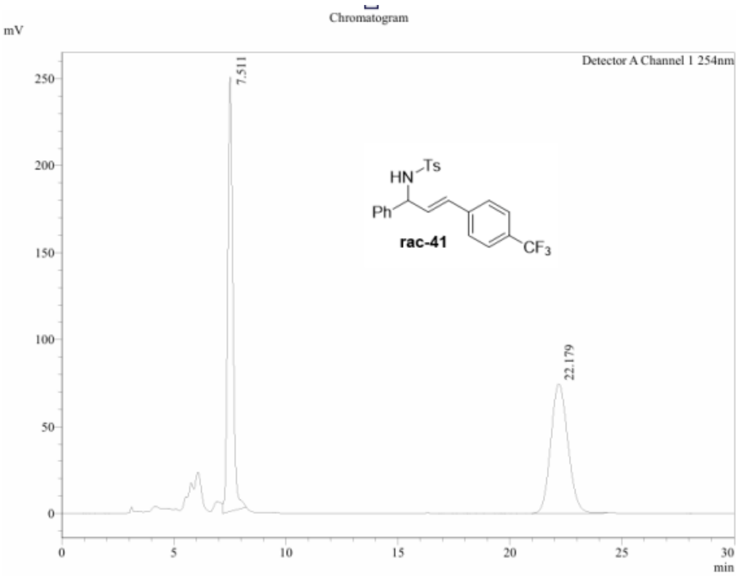

Peak Table

Detector A Channel 1 254nm

Peak#	Ret. Time	Area	Height	Area%	Height%
1	7.511	3987803	249839	50.216	77.049
2	22.179	3953422	74420	49.784	22.951
Total		7941225	324259	100.000	100.000

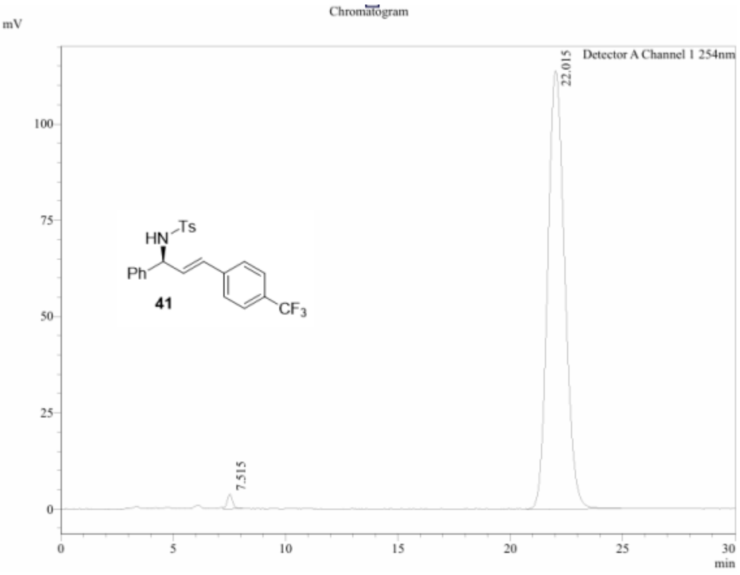

Peak Table

Detector A Channel 1 254nm

Peak#	Ret. Time	Area	Height	Area%	Height%
1	7.515	63747	3855	1.052	3.277
2	22.015	5997886	113798	98.948	96.723
Total		6061633	117653	100.000	100.000

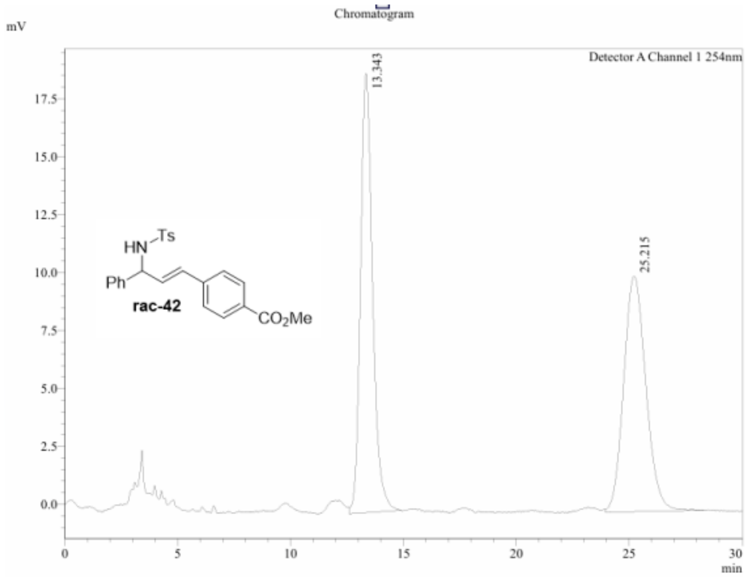

Peak Table

Detector A Channel 1 254nm

Peak#	Ret. Time	Area	Height	Area%	Height%
1	13.343	705998	18947	50.424	65.103
2	25.215	694128	10156	49.576	34.897
Total		1400126	29103	100.000	100.000

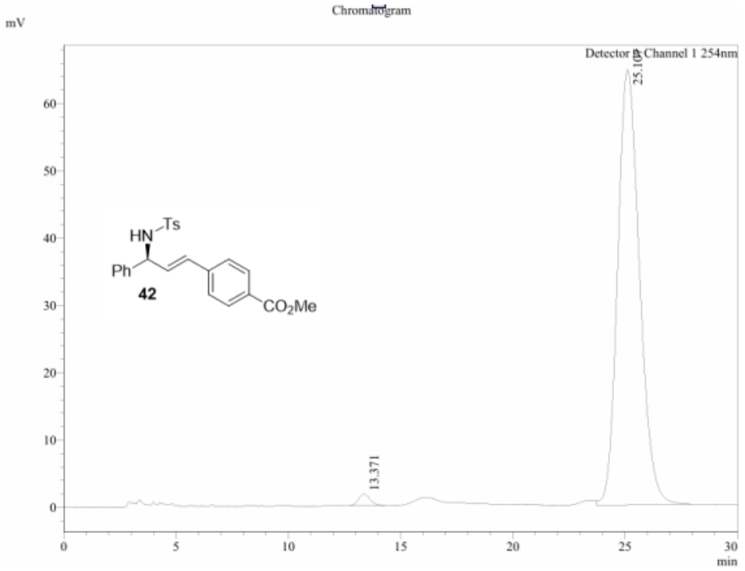

Peak Table

Detector A Channel 1 254nm

Peak#	Ret. Time	Area	Height	Area%	Height%
1	13.371	61000	1701	1.374	2.561
2	25.107	4379067	64704	98.626	97.439
Total		4440066	66405	100.000	100.000

Chromatogram

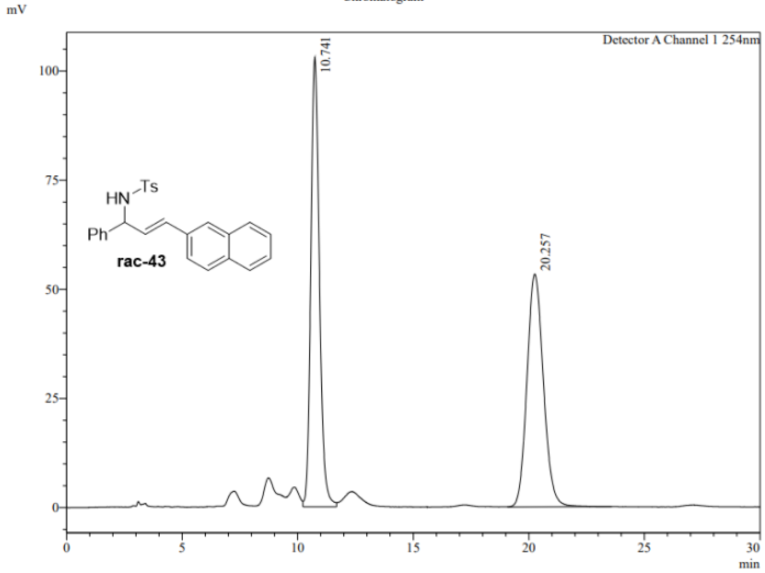

Peak Table

Detector A Channel 1 254nm

Peak#	Ret. Time	Area	Height	Area%	Height%
1	10.741	2648102	102942	50.087	65.865
2	20.257	2638946	53350	49.913	34.135
Total		5287048	156292	100.000	100.000

Chromatogram

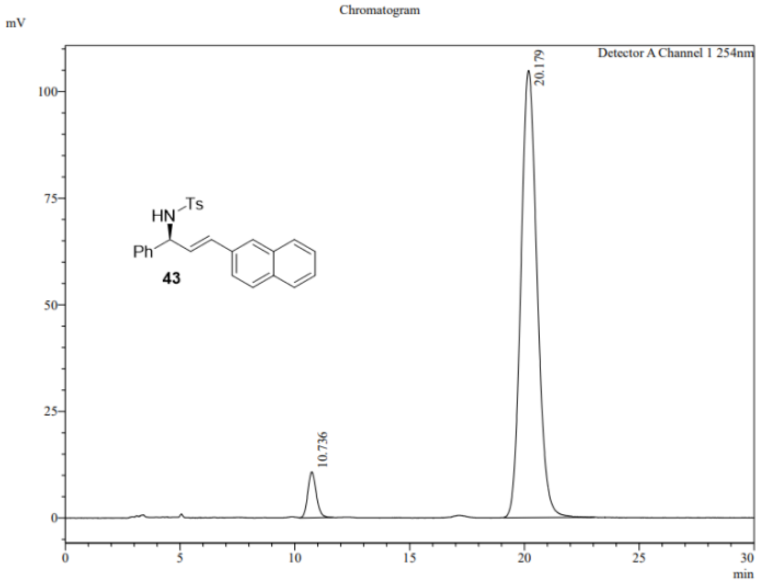

Peak Table

Detector A Channel 1 254nm

Peak#	Ret. Time	Area	Height	Area%	Height%
1	10.736	275135	10743	5.087	9.293
2	20.179	5133228	104855	94.913	90.707
Total		5408363	115598	100.000	100.000

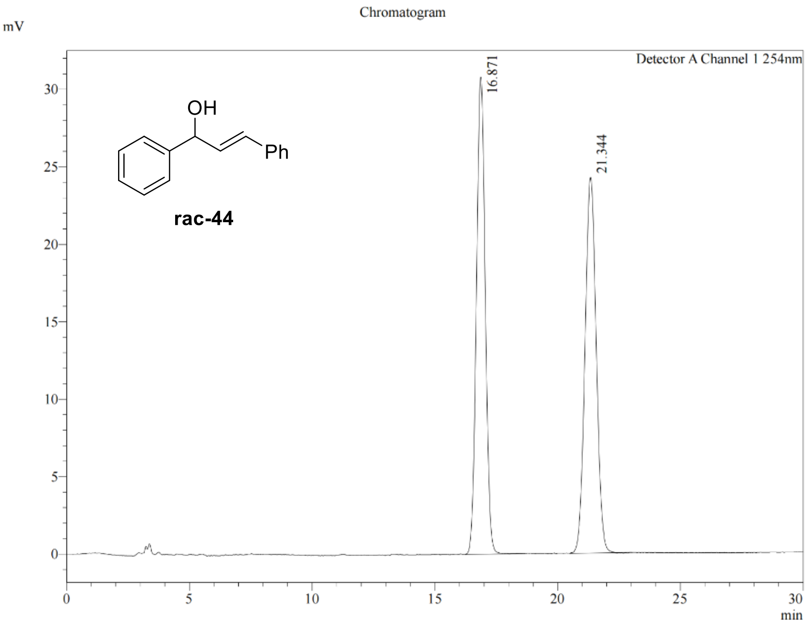

Peak Table

Detector A Channel 1 254nm

Peak#	Ret. Time	Area	Height	Area%	Height%
1	16.871	769440	30794	50.005	55.927
2	21.344	769301	24268	49.995	44.073
Total		1538742	55062	100.000	100.000

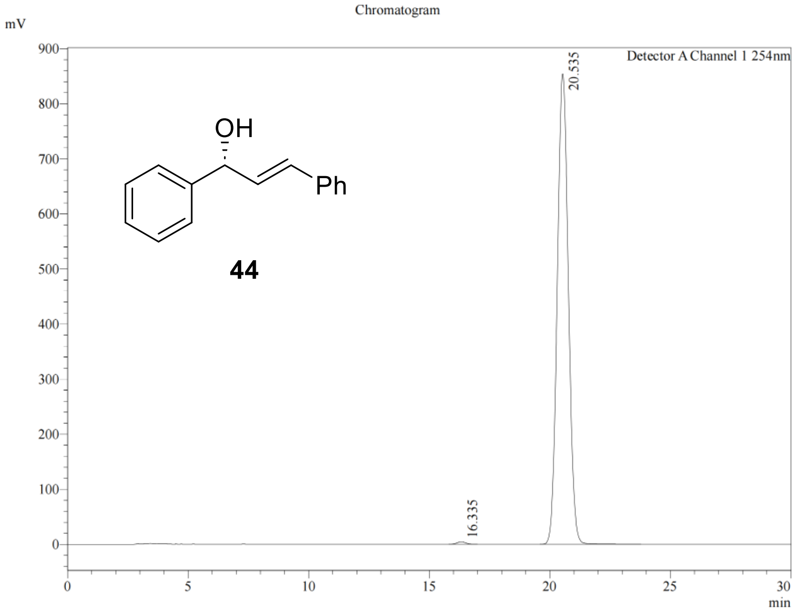

Peak Table

Detector A Channel 1 254nm

Peak#	Ret. Time	Area	Height	Area%	Height%
1	16.335	114876	4558	0.419	0.531
2	20.535	27320193	854147	99.581	99.469
Total		27435069	858705	100.000	100.000

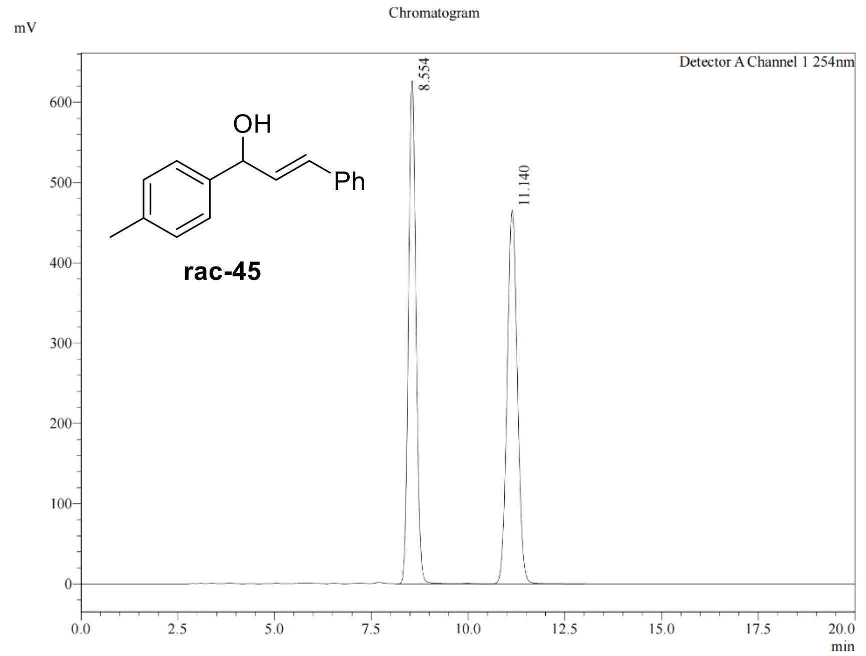

Peak Table

Detector A Channel 1 254nm

Peak#	Ret. Time	Area	Height	Area%	Height%
1	8.554	8515821	626356	49.857	57.372
2	11.140	8564543	465387	50.143	42.628
Total		17080364	1091743	100.000	100.000

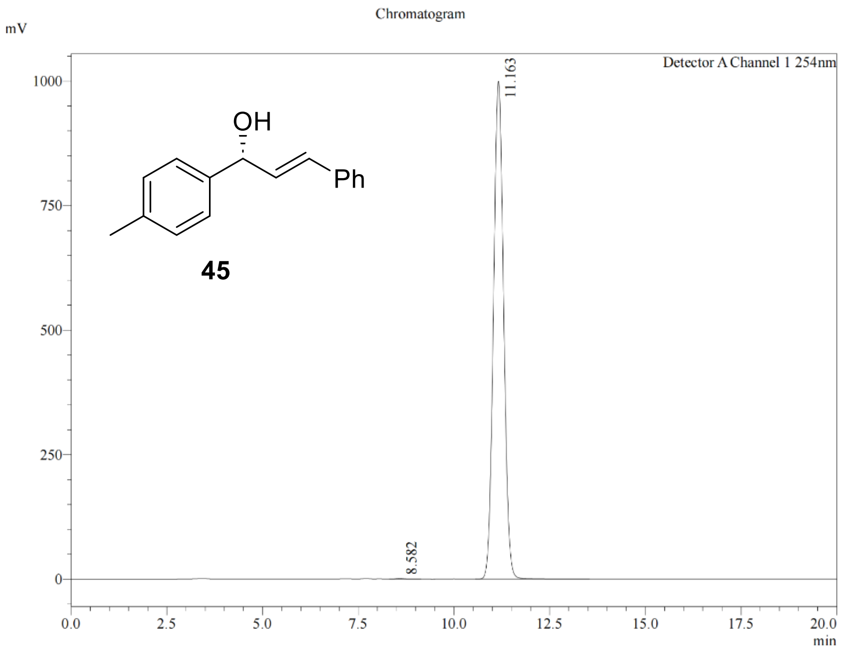

Peak Table

Detector A Channel 1 254nm

Peak#	Ret. Time	Area	Height	Area%	Height%
1	8.582	24478	1601	0.133	0.160
2	11.163	18432062	999214	99.867	99.840
Total		18456540	1000815	100.000	100.000

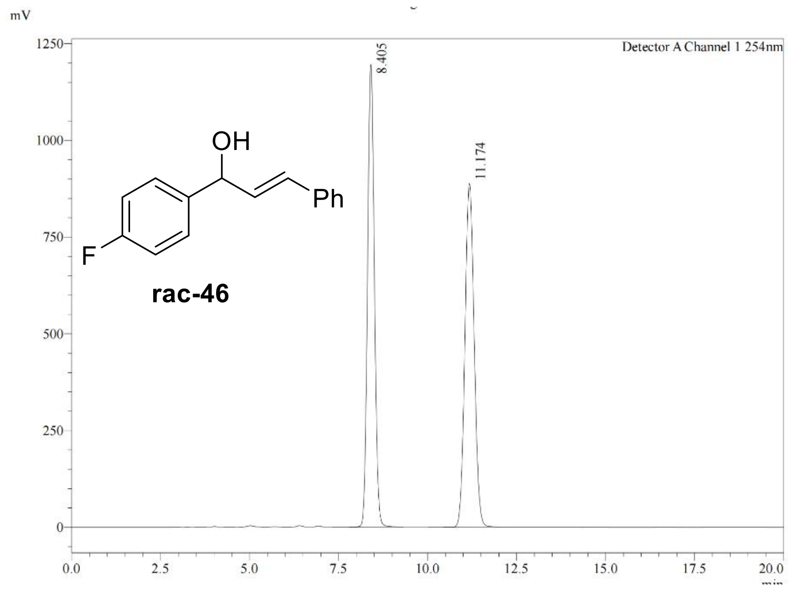

Peak Table

Detector A Channel 1 254nm

Peak#	Ret. Time	Area	Height	Area%	Height%
1	8.405	16020711	1195469	50.006	57.384
2	11.174	16016657	887822	49.994	42.616
Total		32037369	2083291	100.000	100.000

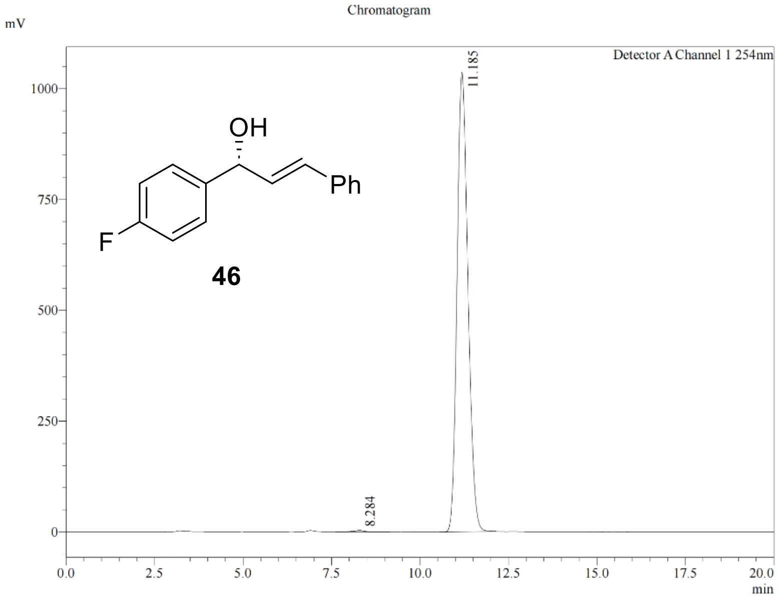

Peak Table

Detector A Channel 1 254nm

Peak#	Ret. Time	Area	Height	Area%	Height%
1	8.284	94122	4004	0.423	0.385
2	11.185	22166274	1035894	99.577	99.615
Total		22260396	1039898	100.000	100.000

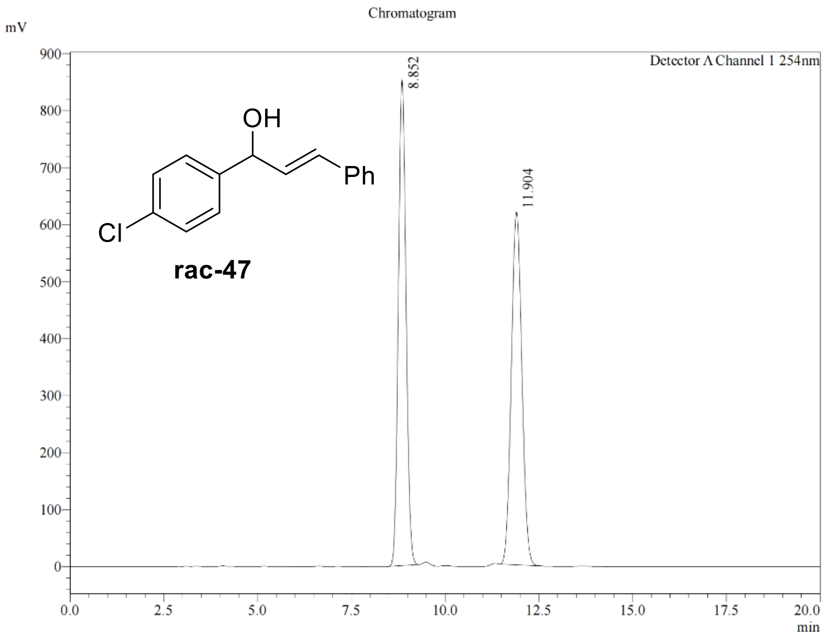

Peak Table

Detector A Channel 1 254nm

Peak#	Ret. Time	Area	Height	Area%	Height%
1	8.852	12155446	853317	50.107	57.960
2	11.904	12103355	618947	49.893	42.040
Total		24258800	1472264	100.000	100.000

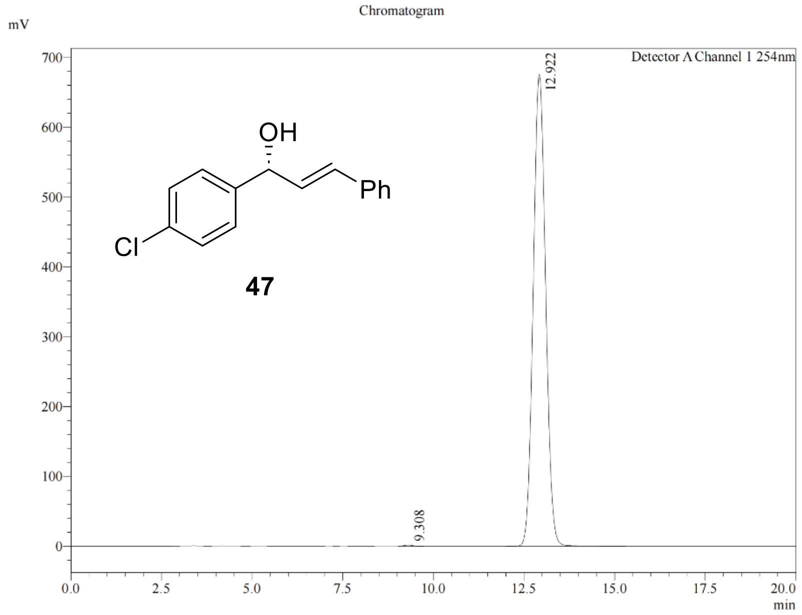

Peak Table

Detector A Channel 1 254nm

Peak#	Ret. Time	Area	Height	Area%	Height%
1	9.308	30811	1779	0.192	0.263
2	12.922	16036765	675280	99.808	99.737
Total		16067577	677058	100.000	100.000

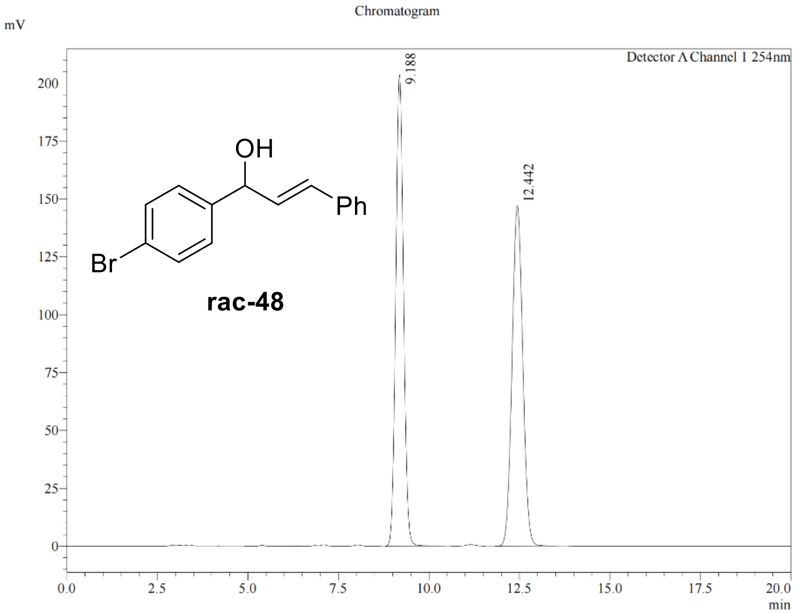

Peak Table

Detector A Channel 1 254nm

Peak#	Ret. Time	Area	Height	Area%	Height%
1	9.188	3034768	203570	50.111	58.084
2	12.442	3021358	146904	49.889	41.916
Total		6056126	350474	100.000	100.000

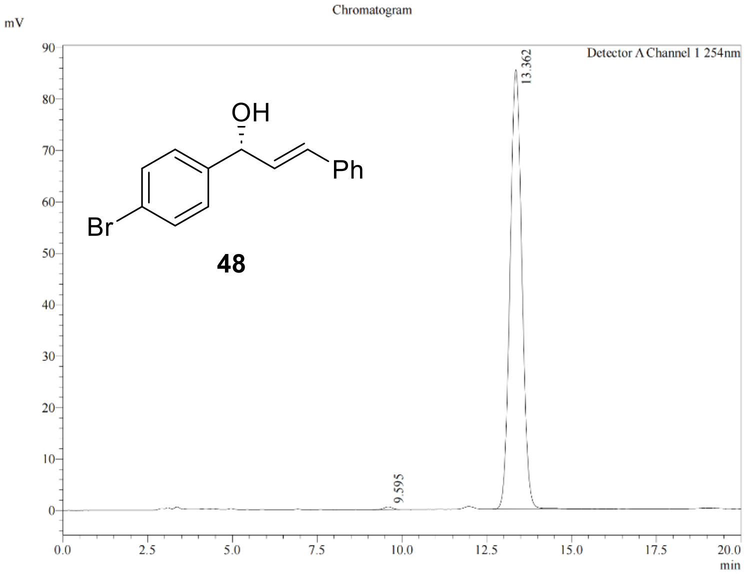

Peak Table

Detector A Channel 1 254nm

Peak#	Ret. Time	Area	Height	Area%	Height%
1	9.595	9365	532	0.448	0.619
2	13.362	2081478	85456	99.552	99.381
Total		2090843	85989	100.000	100.000

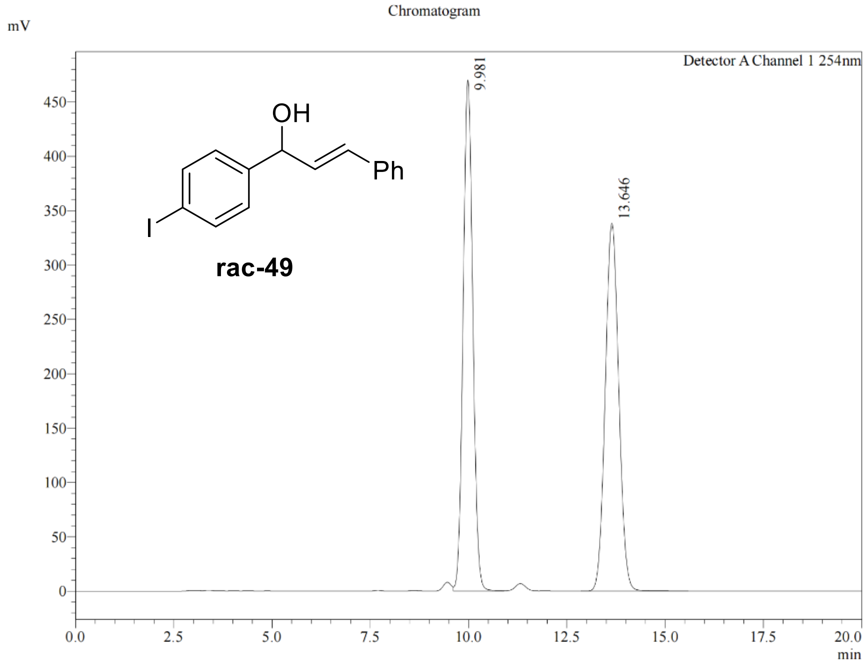

Peak Table

Detector A Channel 1 254nm

Peak#	Ret. Time	Area	Height	Area%	Height%
1	9.981	8075649	470073	49.957	58.145
2	13.646	8089470	338373	50.043	41.855
Total		16165119	808447	100.000	100.000

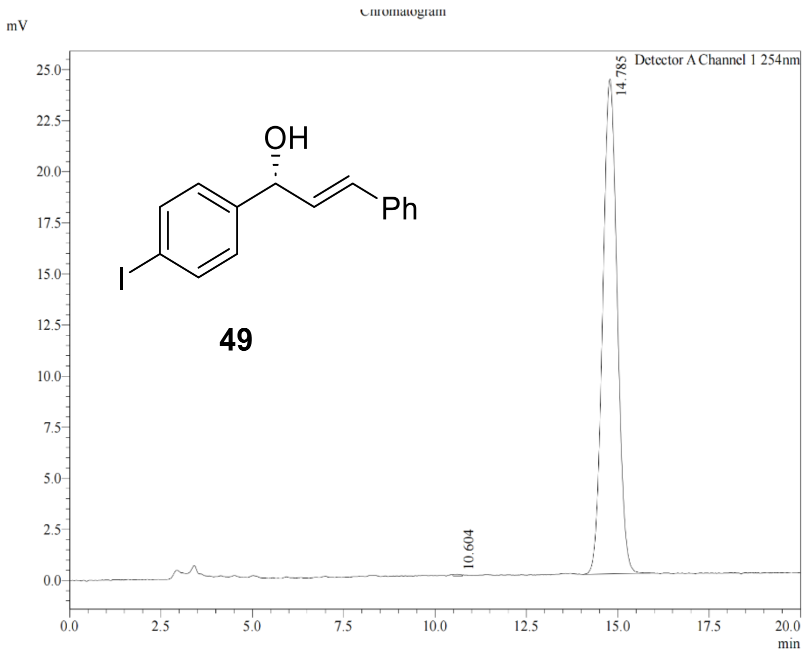

Peak Table

Detector A Channel 1 254nm

Peak#	Ret. Time	Area	Height	Area%	Height%
1	10.604	1070	82	0.159	0.336
2	14.785	669989	24217	99.841	99.664
Total		671059	24299	100.000	100.000

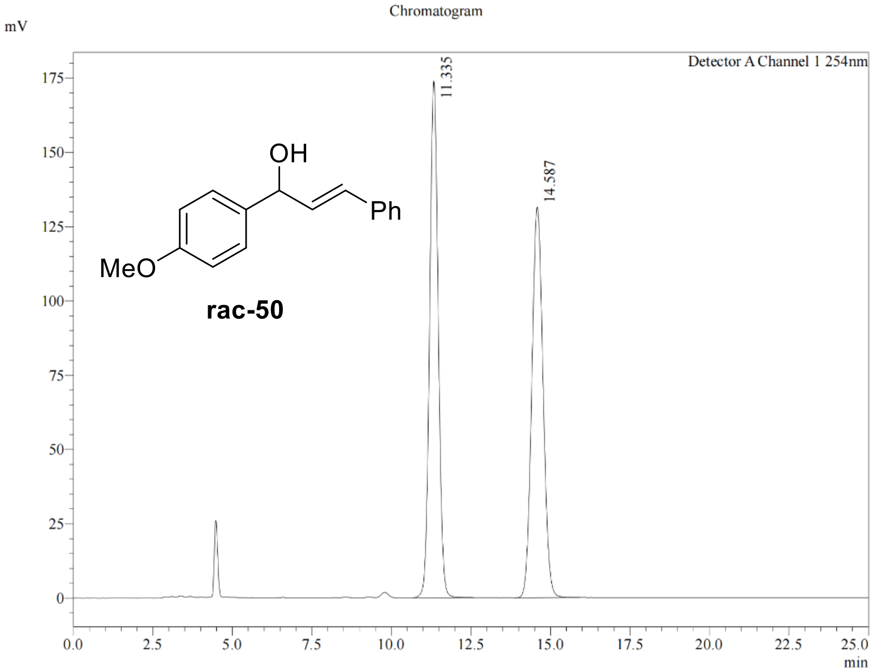

Peak Table

Detector A Channel 1 254nm

Peak#	Ret. Time	Area	Height	Area%	Height%
1	11.335	3269436	173810	50.225	56.932
2	14.587	3240164	131485	49.775	43.068
Total		6509599	305296	100.000	100.000

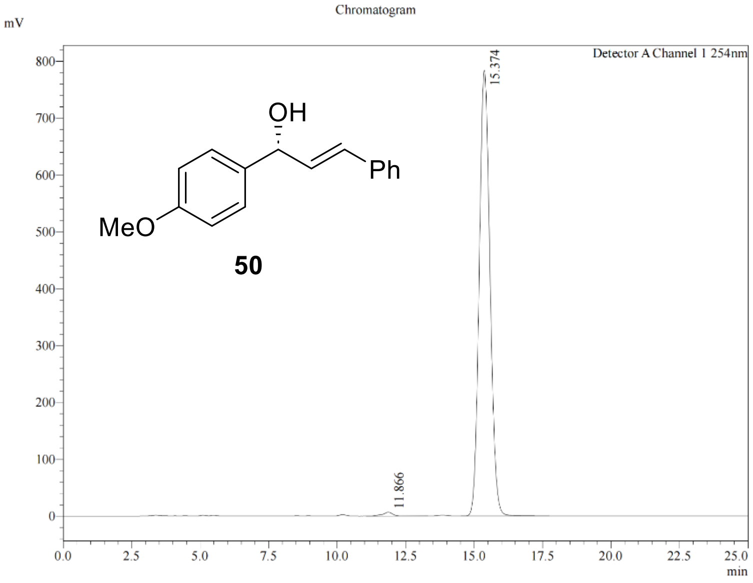

Peak Table

Detector A Channel 1 254nm

Peak#	Ret. Time	Area	Height	Area%	Height%
1	11.866	174453	6749	0.807	0.854
2	15.374	21454789	783900	99.193	99.146
Total		21629242	790648	100.000	100.000

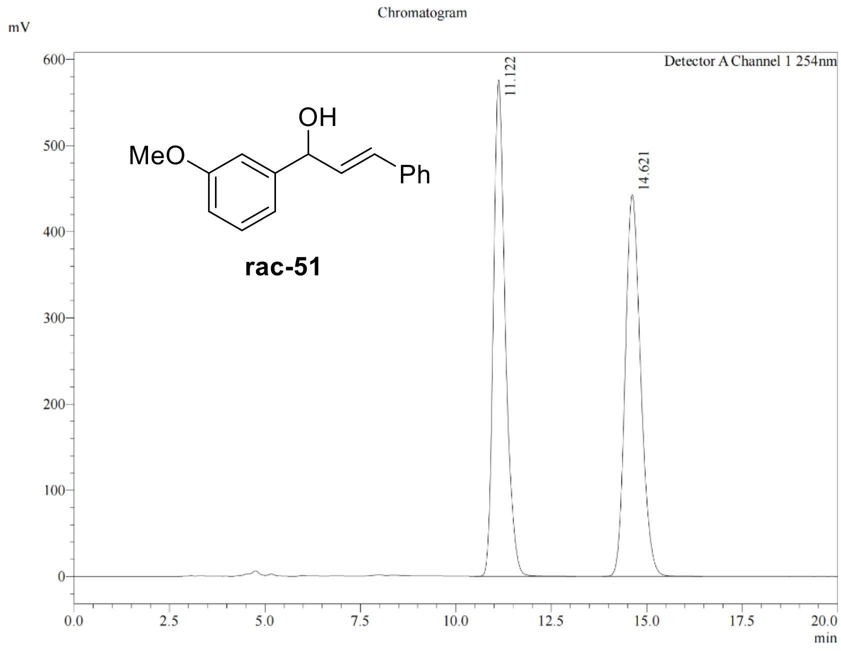

Peak Table

Detector A Channel 1 254nm

Peak#	Ret. Time	Area	Height	Area%	Height%
1	11.122	12513943	575942	50.010	56.536
2	14.621	12508904	442769	49.990	43.464
Total		25022848	1018711	100.000	100.000

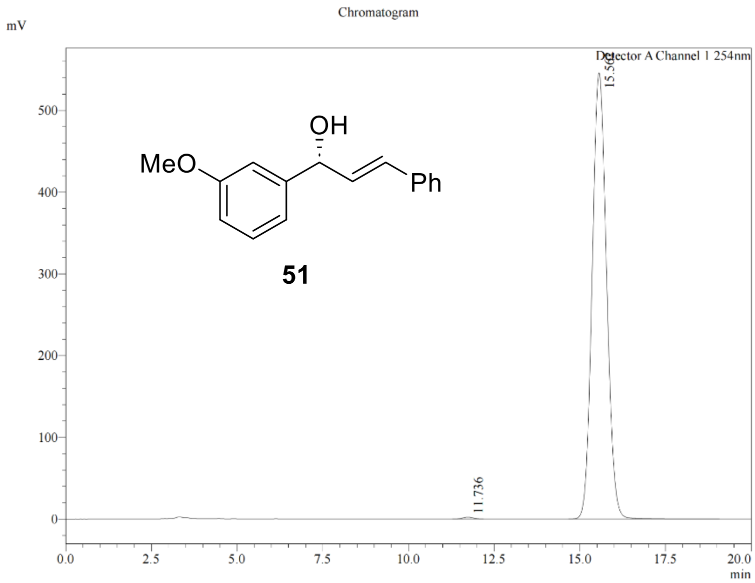

Peak Table

Detector A Channel 1 254nm

Peak#	Ret. Time	Area	Height	Area%	Height%
1	11.736	50578	2394	0.316	0.437
2	15.562	15937762	545852	99.684	99.563
Total		15988339	548246	100.000	100.000

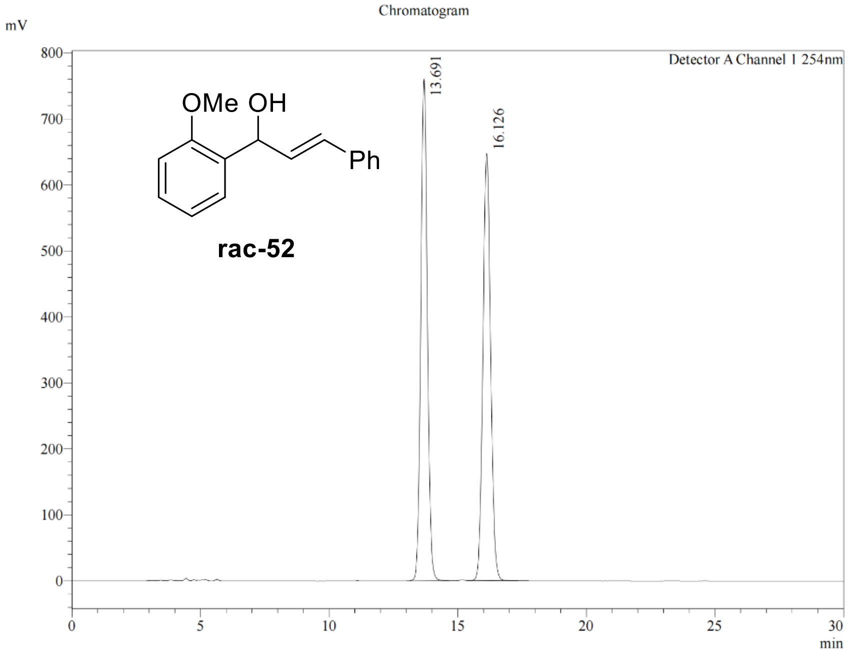

Peak Table

Detector A Channel 1 254nm

Peak#	Ret. Time	Area	Height	Area%	Height%
1	13.691	13527192	760728	50.087	54.046
2	16.126	13479962	646830	49.913	45.954
Total		27007155	1407558	100.000	100.000

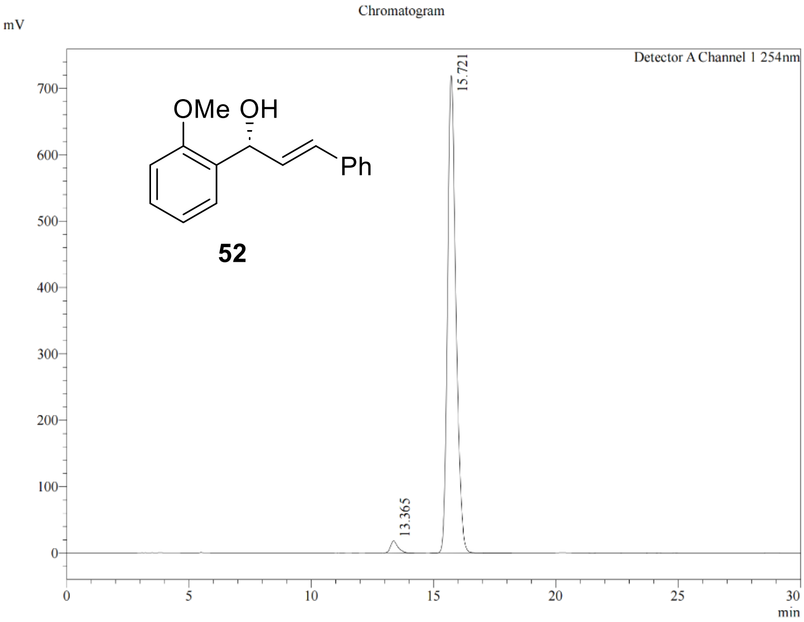

Peak Table

Detector A Channel 1 254nm

Peak#	Ret. Time	Area	Height	Area%	Height%
1	13.365	383399	18118	2.187	2.457
2	15.721	17148870	719208	97.813	97.543
Total		17532269	737327	100.000	100.000

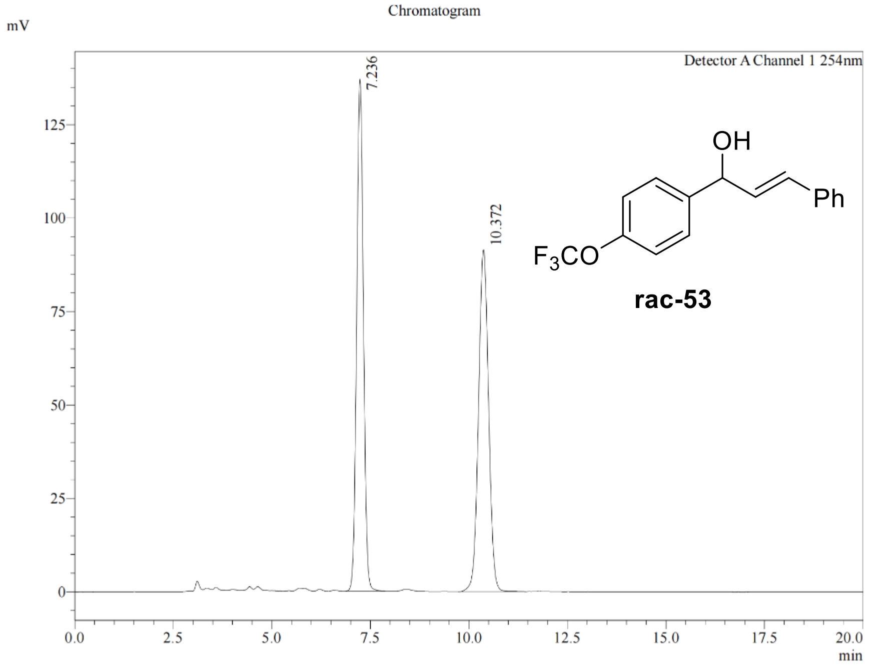

Peak Table

Detector A Channel 1 254nm

Peak#	Ret. Time	Area	Height	Area%	Height%
1	7.236	1631487	136729	50.017	59.922
2	10.372	1630361	91450	49.983	40.078
Total		3261847	228179	100.000	100.000

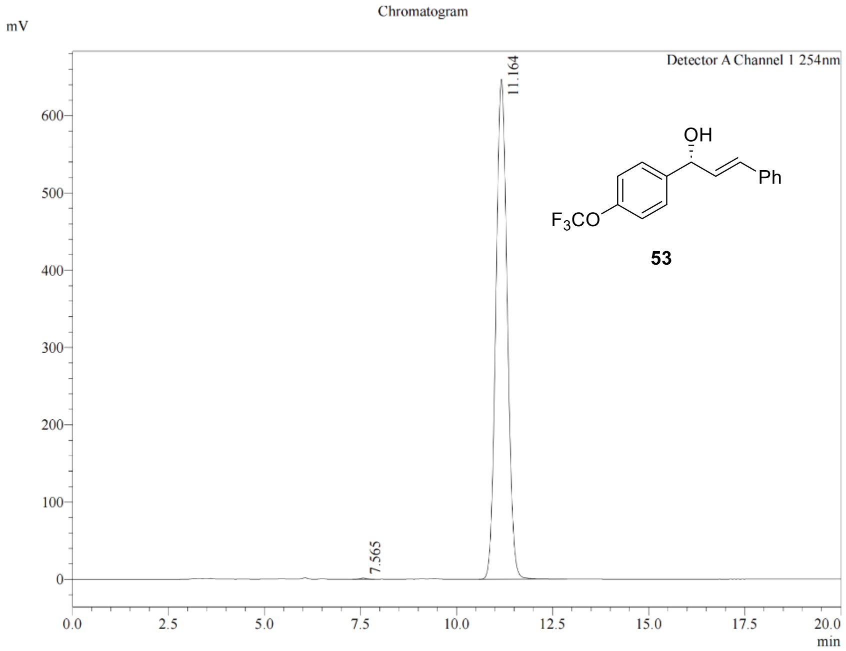

Peak Table

Detector A Channel 1 254nm

Peak#	Ret. Time	Area	Height	Area%	Height%
1	7.565	20491	1455	0.154	0.224
2	11.164	13301246	646969	99.846	99.776
Total		13321737	648424	100.000	100.000

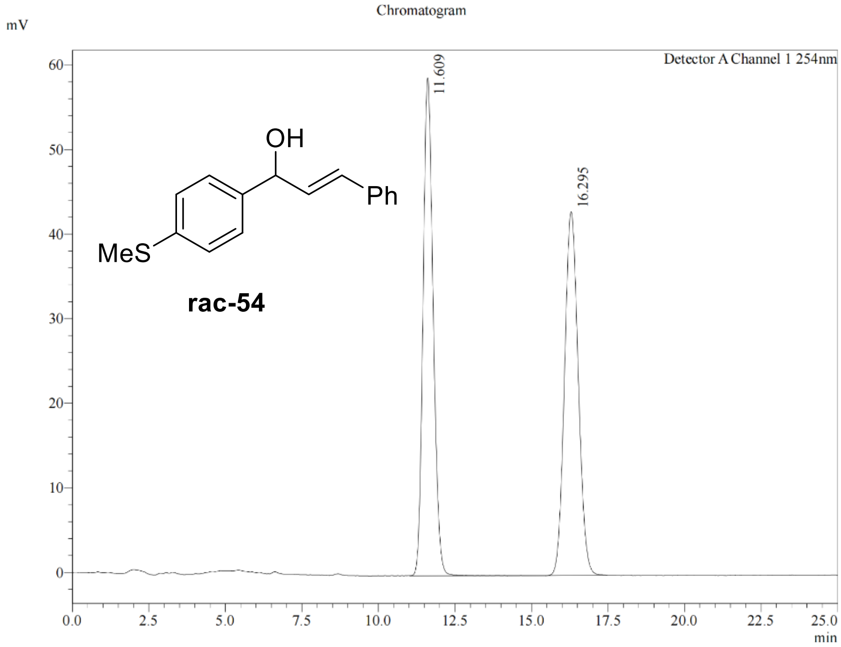

Peak Table

Detector A Channel 1 254nm

Peak#	Ret. Time	Area	Height	Area%	Height%
1	11.609	1357913	58863	50.291	57.788
2	16.295	1342208	42997	49.709	42.212
Total		2700121	101860	100.000	100.000

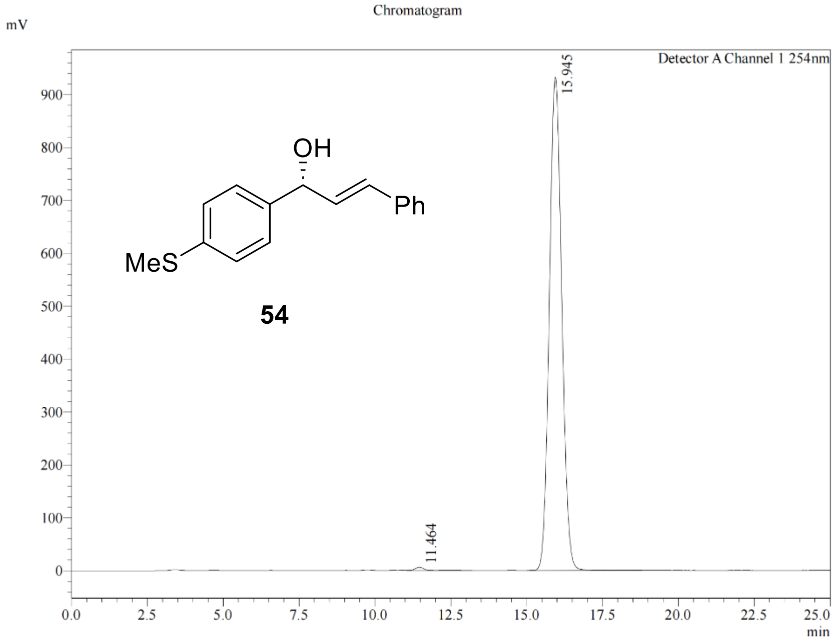

Peak Table

Detector A Channel 1 254nm

Peak#	Ret. Time	Area	Height	Area%	Height%
1	11.464	134122	6166	0.503	0.657
2	15.945	26535361	932629	99.497	99.343
Total		26669484	938795	100.000	100.000

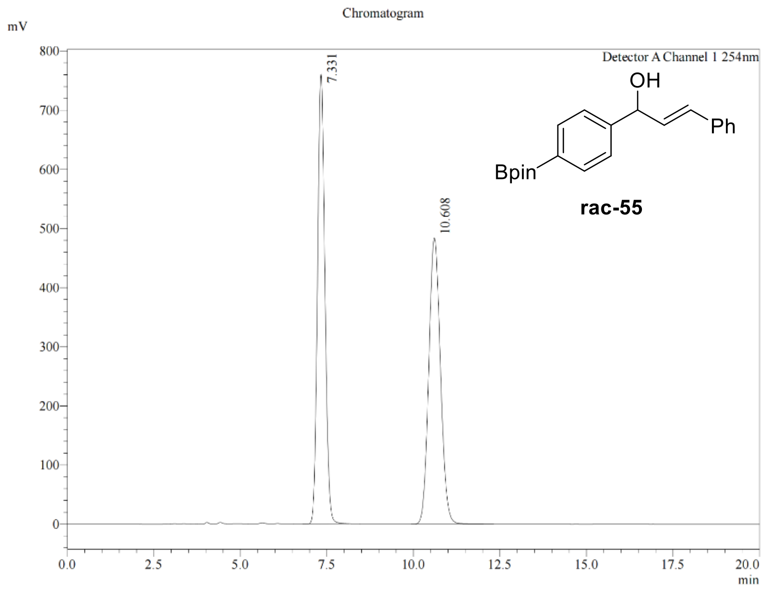

Peak Table

Detector A Channel 1 254nm

Peak#	Ret. Time	Area	Height	Area%	Height%
1	7.331	11328423	760586	49.911	61.120
2	10.608	11368599	483822	50.089	38.880
Total		22697022	1244409	100.000	100.000

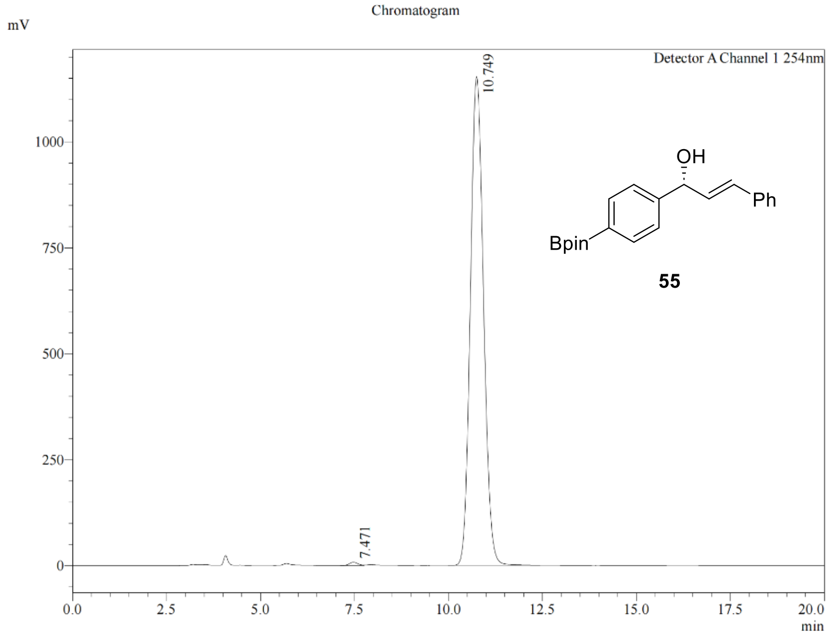

Peak Table

Detector A Channel 1 254nm

Peak#	Ret. Time	Area	Height	Area%	Height%
1	7.471	131826	8008	0.468	0.690
2	10.749	28029593	1152872	99.532	99.310
Total		28161419	1160881	100.000	100.000

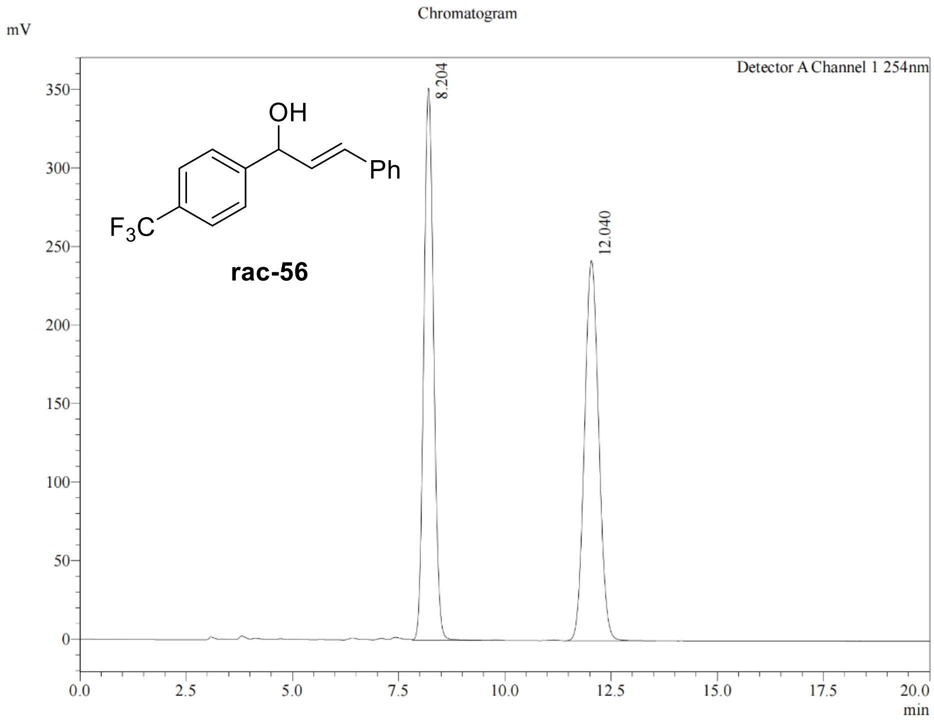

Peak Table

Detector A Channel 1 254nm

Peak#	Ret. Time	Area	Height	Area%	Height%
1	8.204	5642203	351345	49.964	59.210
2	12.040	5650352	242047	50.036	40.790
Total		11292554	593392	100.000	100.000

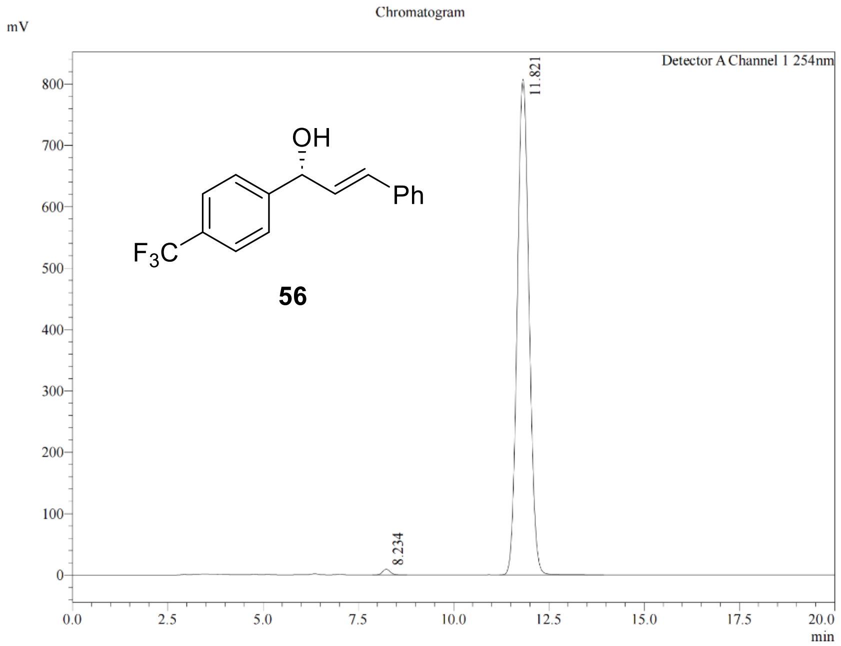

Peak Table

Detector A Channel 1 254nm

Peak#	Ret. Time	Area	Height	Area%	Height%
1	8.234	137472	9423	0.784	1.154
2	11.821	17405819	807123	99.216	98.846
Total		17543290	816547	100.000	100.000

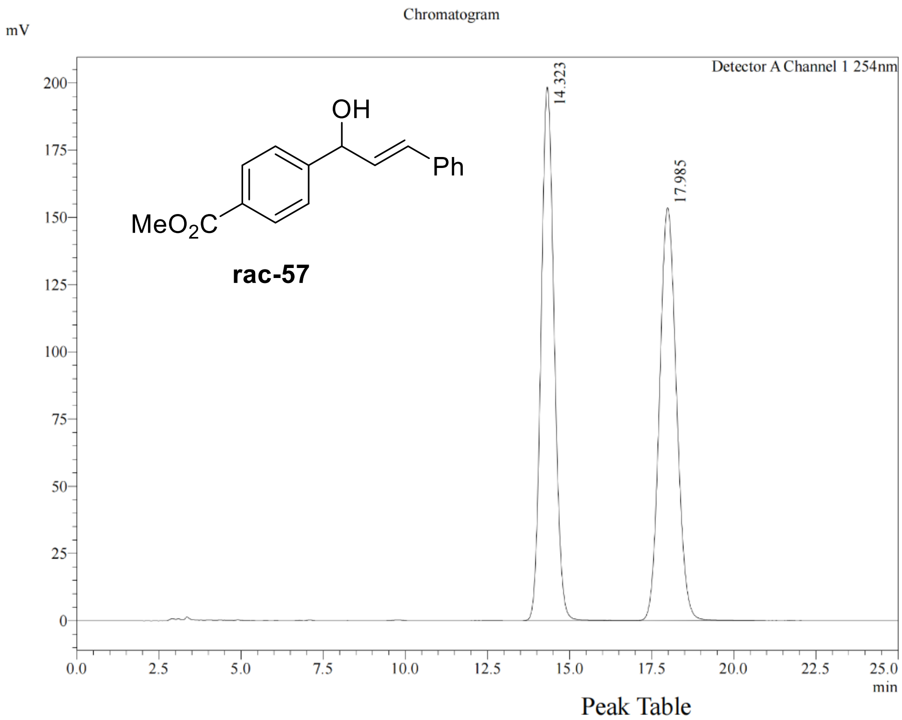

Peak Table

Detector A Channel 1 254nm

Peak#	Ret. Time	Area	Height	Area%	Height%
1	14.323	5527150	198526	49.999	56.385
2	17.985	5527299	153562	50.001	43.615
Total		11054449	352088	100.000	100.000

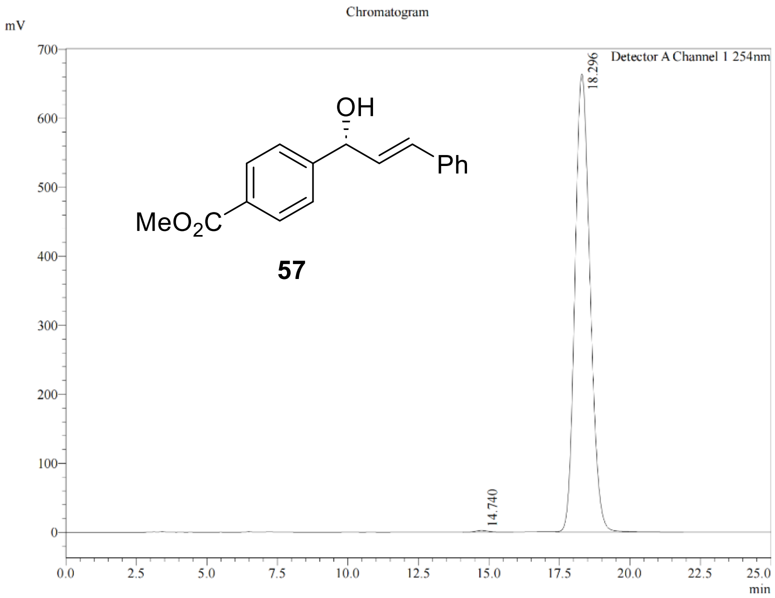

Peak Table

Detector A Channel 1 254nm

Peak#	Ret. Time	Area	Height	Area%	Height%
1	14.740	74576	2407	0.300	0.361
2	18.296	24775316	663536	99.700	99.639
Total		24849893	665944	100.000	100.000

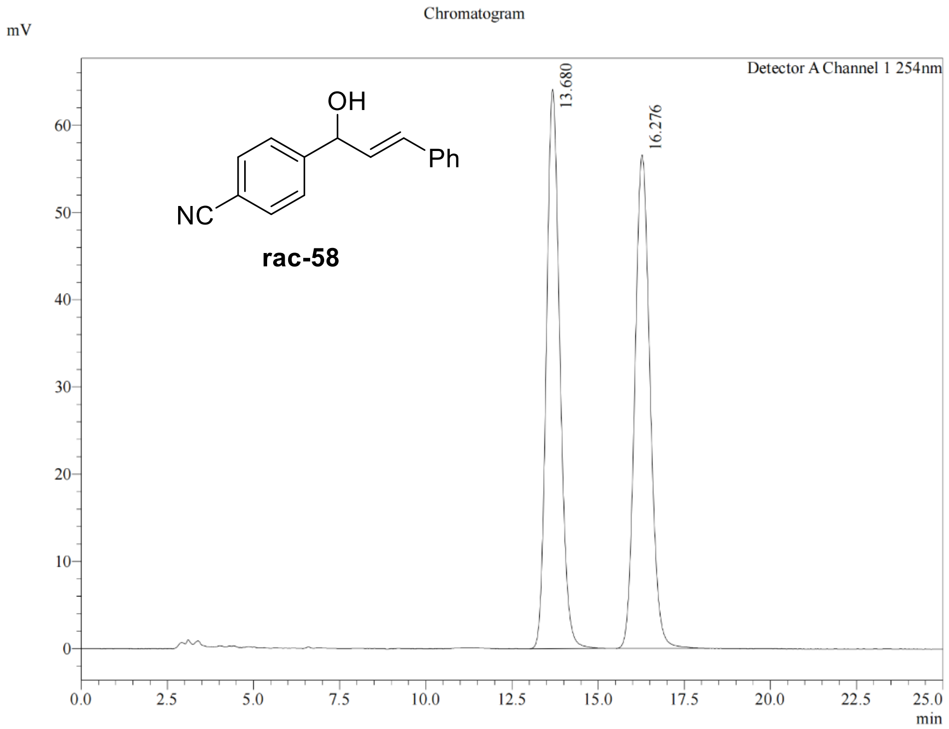

Peak Table

Detector A Channel 1 254nm

Peak#	Ret. Time	Area	Height	Area%	Height%
1	13.680	1743269	64117	50.085	53.126
2	16.276	1737351	56570	49.915	46.874
Total		3480619	120687	100.000	100.000

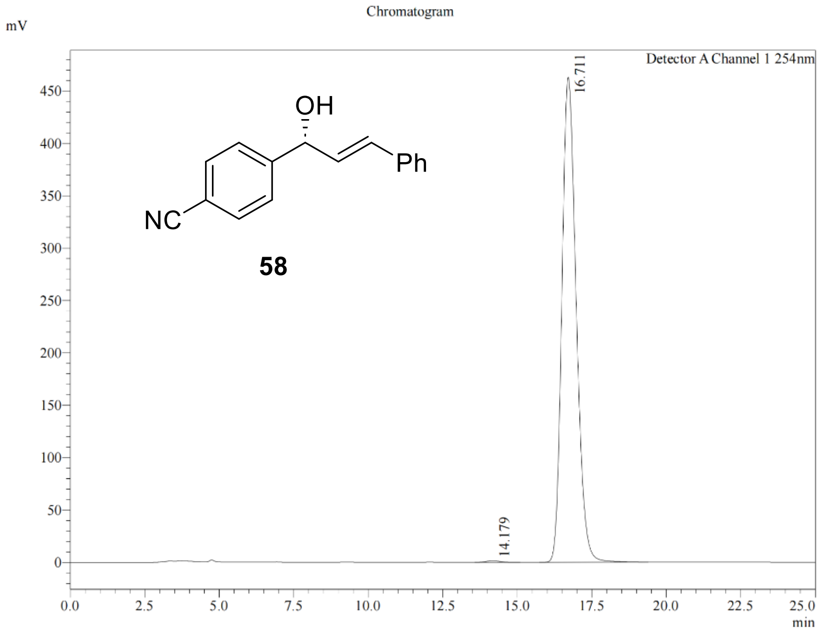

Peak Table

Detector A Channel 1 254nm

Peak#	Ret. Time	Area	Height	Area%	Height%
1	14.179	47366	1487	0.306	0.320
2	16.711	15437304	462958	99.694	99.680
Total		15484670	464445	100.000	100.000

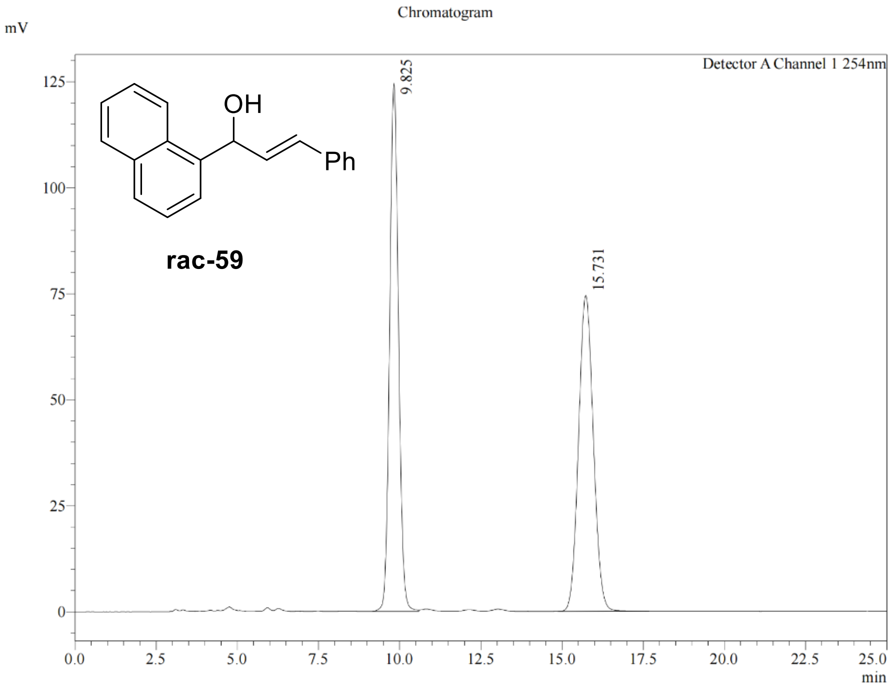

Peak Table

Detector A Channel 1 254nm

Peak#	Ret. Time	Area	Height	Area%	Height%
1	9.825	2370174	124401	50.097	62.539
2	15.731	2360965	74518	49.903	37.461
Total		4731140	198918	100.000	100.000

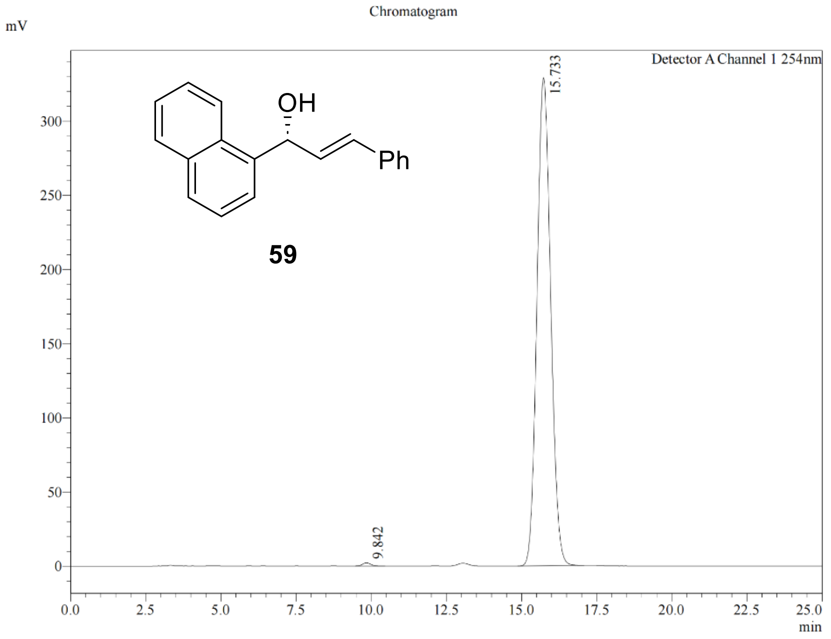

Peak Table

Detector A Channel 1 254nm

Peak#	Ret. Time	Area	Height	Area%	Height%
1	9.842	42958	2163	0.410	0.653
2	15.733	10423305	329158	99.590	99.347
Total		10466263	331321	100.000	100.000

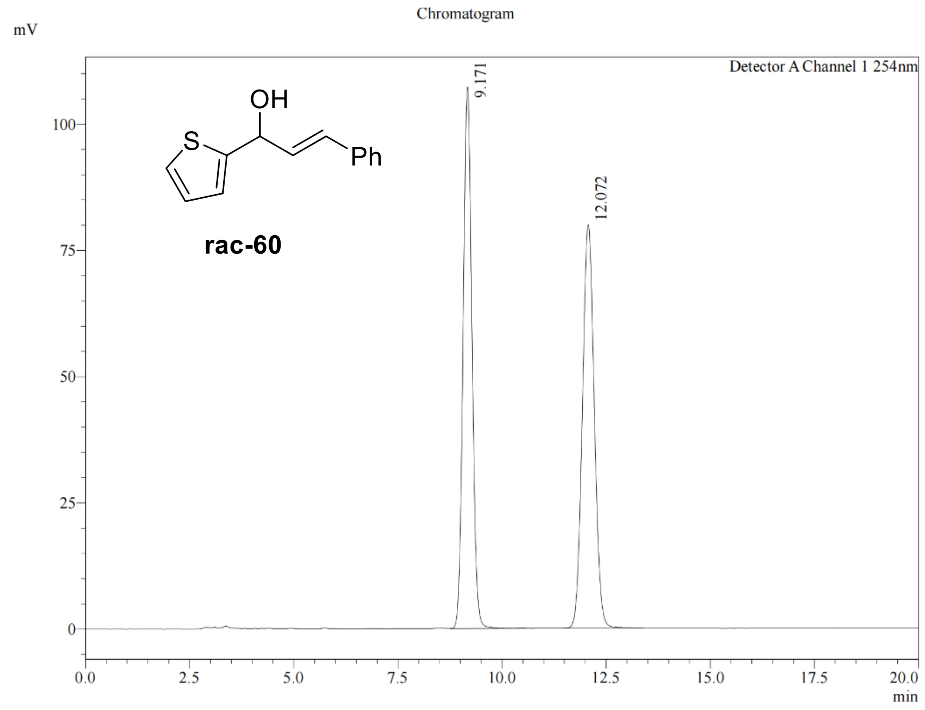

Peak Table

Detector A Channel 1 254nm

Peak#	Ret. Time	Area	Height	Area%	Height%
1	9.171	1588811	107222	50.061	57.311
2	12.072	1584910	79866	49.939	42.689
Total		3173721	187088	100.000	100.000

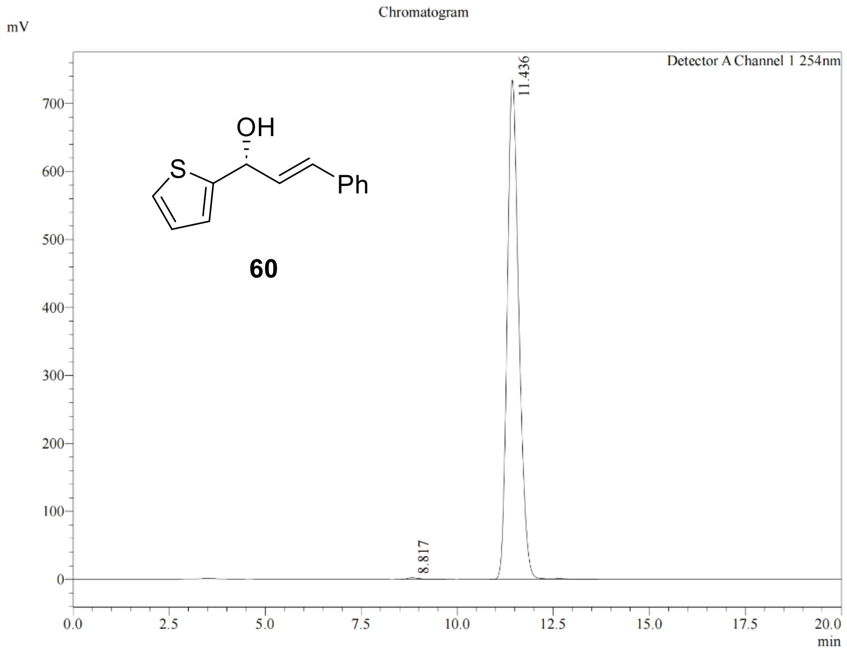

Peak Table

Detector A Channel 1 254nm

Peak#	Ret. Time	Area	Height	Area%	Height%
1	8.817	50925	2578	0.327	0.350
2	11.436	15521634	734635	99.673	99.650
Total		15572560	737213	100.000	100.000

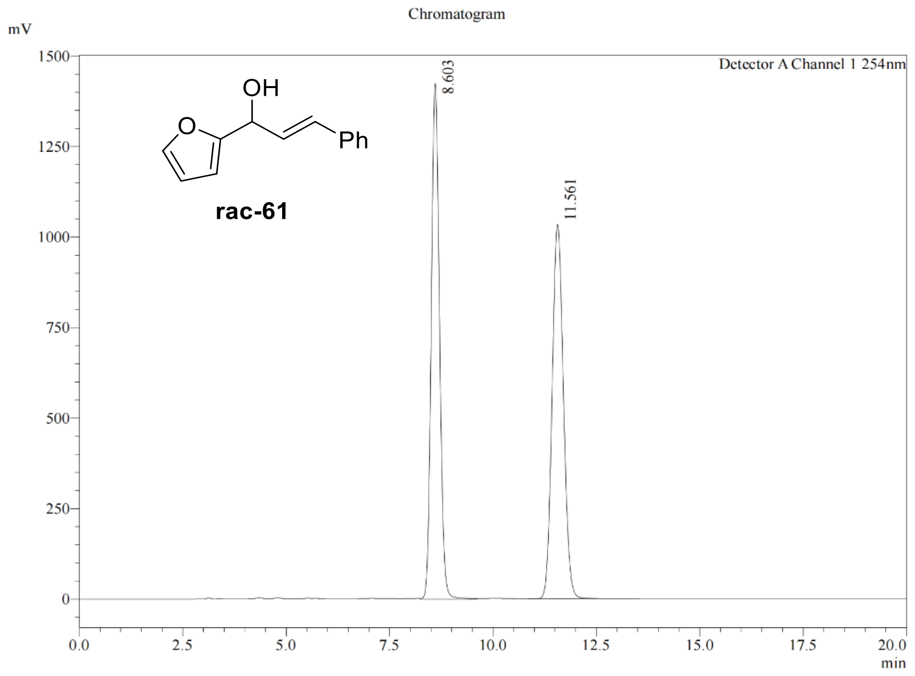

Peak Table

Detector A Channel 1 254nm

Peak#	Ret. Time	Area	Height	Area%	Height%
1	8.603	19735548	1423174	50.089	57.929
2	11.561	19665684	1033575	49.911	42.071
Total		39401232	2456749	100.000	100.000

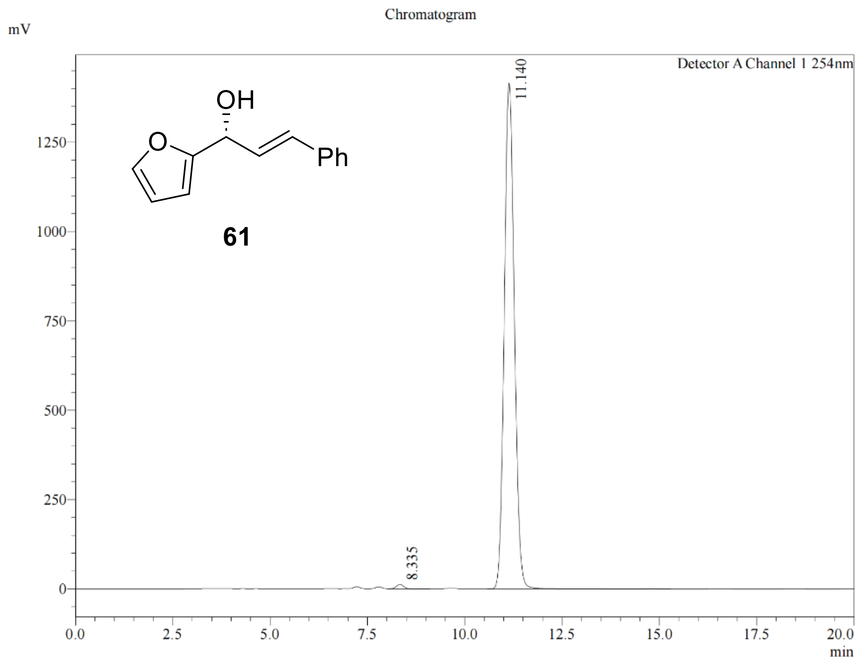

Peak Table

Detector A Channel 1 254nm

Peak#	Ret. Time	Area	Height	Area%	Height%
1	8.335	183351	12835	0.715	0.899
2	11.140	25467457	1415618	99.285	99.101
Total		25650808	1428453	100.000	100.000

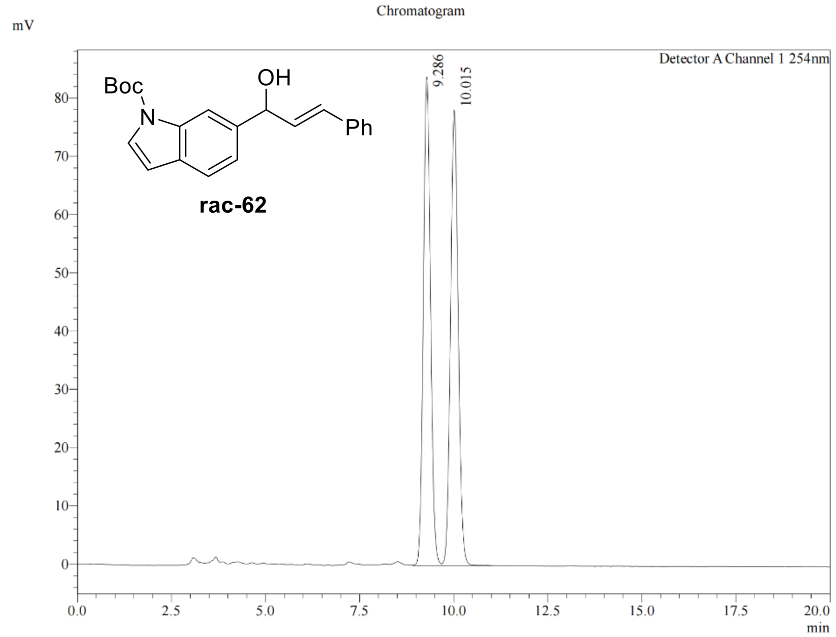

Peak Table

Detector A Channel 1 254nm

Peak#	Ret. Time	Area	Height	Area%	Height%
1	9.286	1112237	83833	49.908	51.753
2	10.015	1116334	78154	50.092	48.247
Total		2228571	161988	100.000	100.000

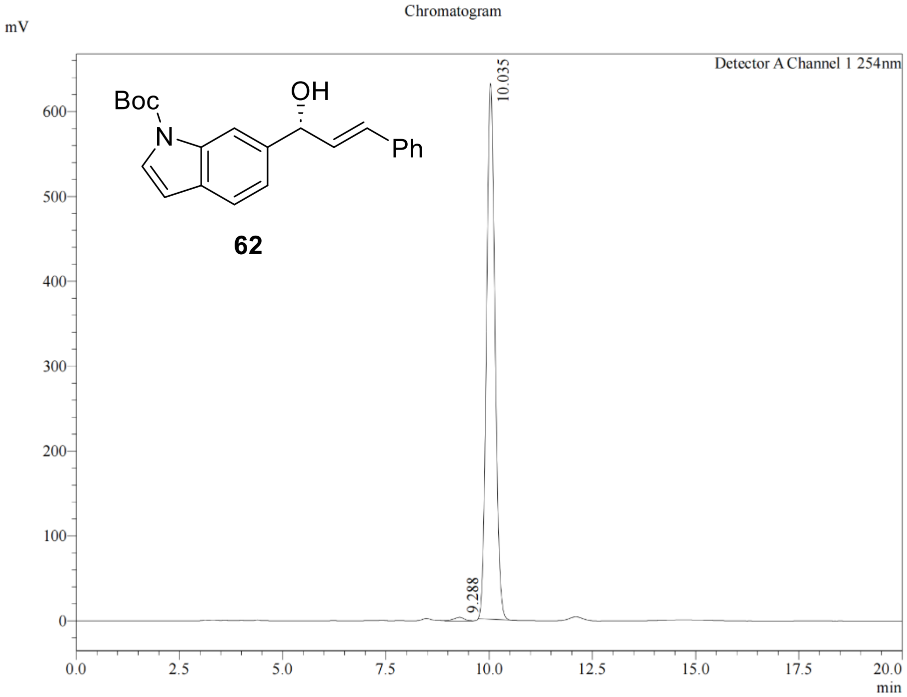

Peak Table

Detector A Channel 1 254nm

Peak#	Ret. Time	Area	Height	Area%	Height%
1	9.288	79502	4308	0.872	0.678
2	10.035	9040329	630730	99.128	99.322
Total		9119831	635038	100.000	100.000

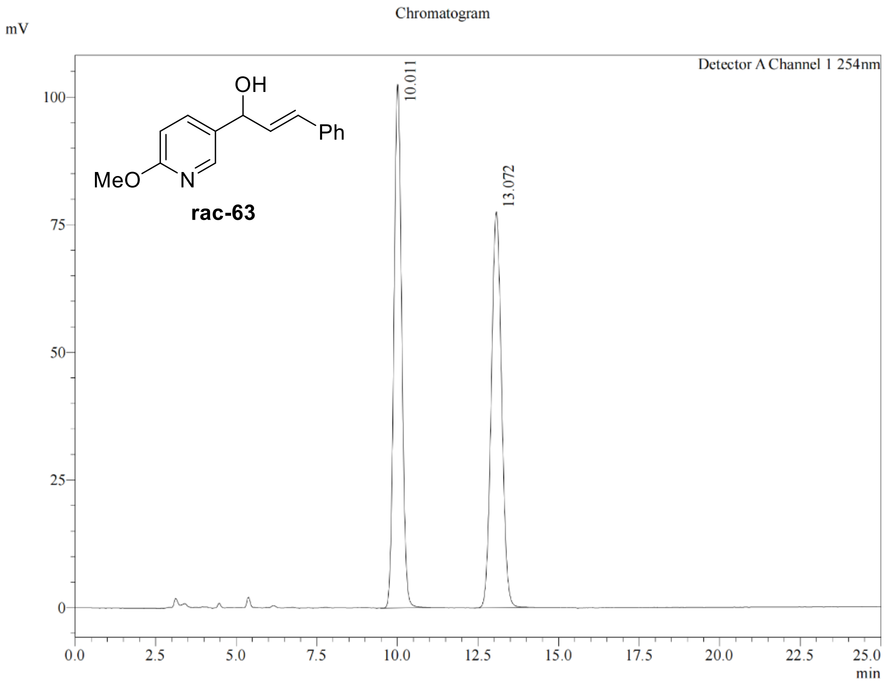

Peak Table

Detector A Channel 1 254nm

Peak#	Ret. Time	Area	Height	Area%	Height%
1	10.011	1729856	102536	49.987	56.934
2	13.072	1730753	77560	50.013	43.066
Total		3460610	180097	100.000	100.000

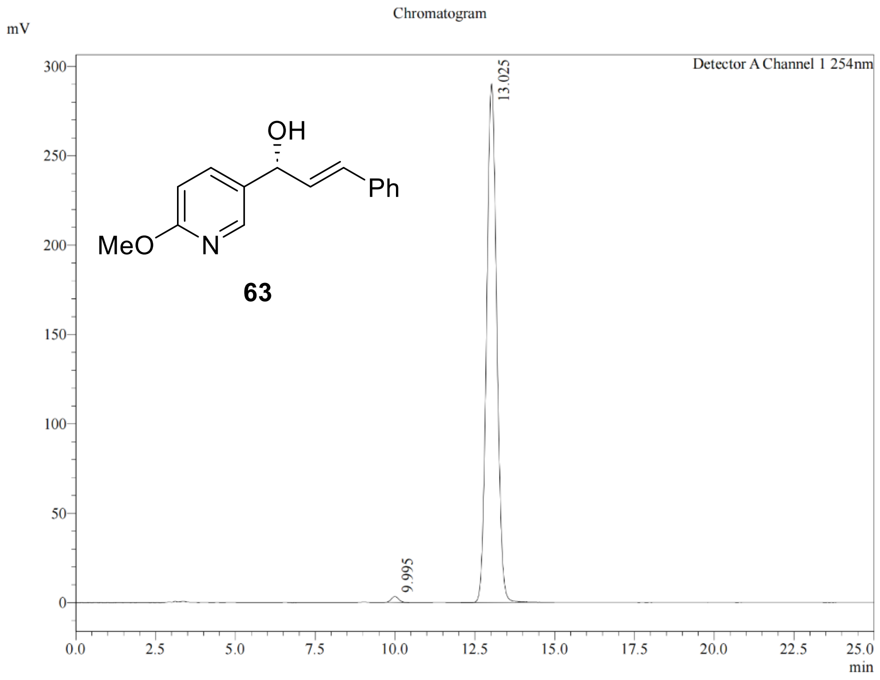

Peak Table

Detector A Channel 1 254nm

Peak#	Ret. Time	Area	Height	Area%	Height%
1	9.995	53330	3280	0.826	1.118
2	13.025	6406926	290187	99.174	98.882
Total		6460256	293467	100.000	100.000

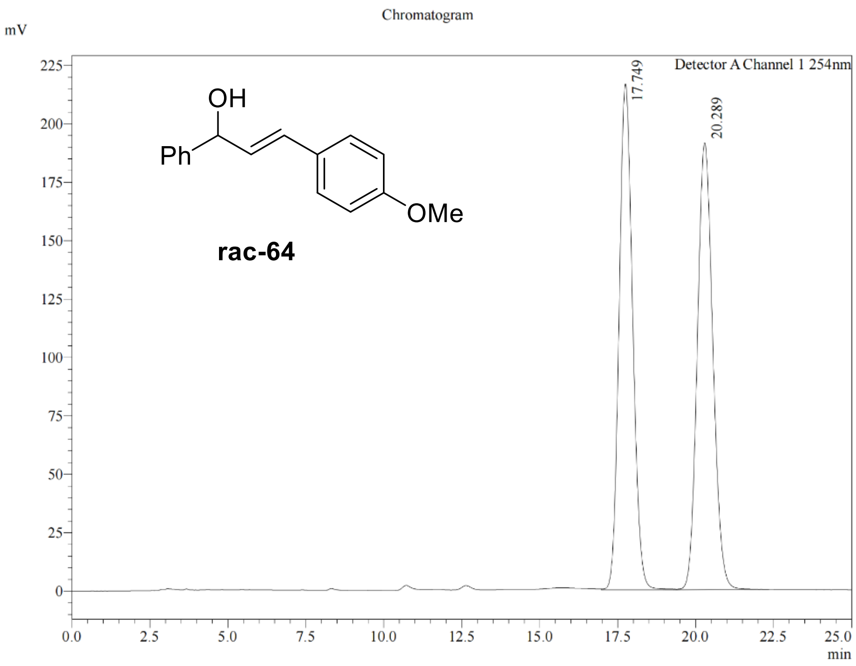

Peak Table

Detector A Channel 1 254nm

Peak#	Ret. Time	Area	Height	Area%	Height%
1	17.749	6556721	216595	49.659	53.099
2	20.289	6646687	191312	50.341	46.901
Total		13203409	407907	100.000	100.000

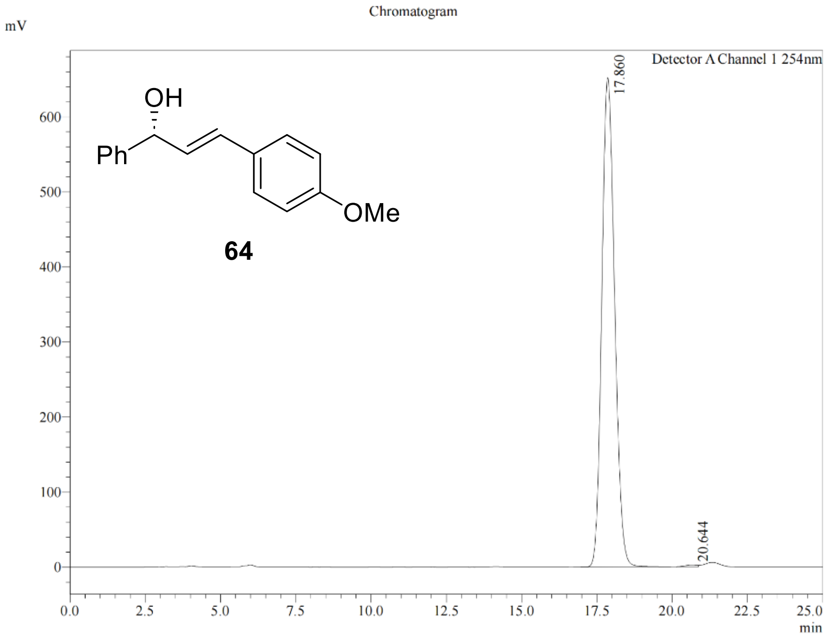

Peak Table

Detector A Channel 1 254nm

Peak#	Ret. Time	Area	Height	Area%	Height%
1	17.860	19307831	651841	99.663	99.645
2	20.644	65305	2319	0.337	0.355
Total		19373136	654160	100.000	100.000

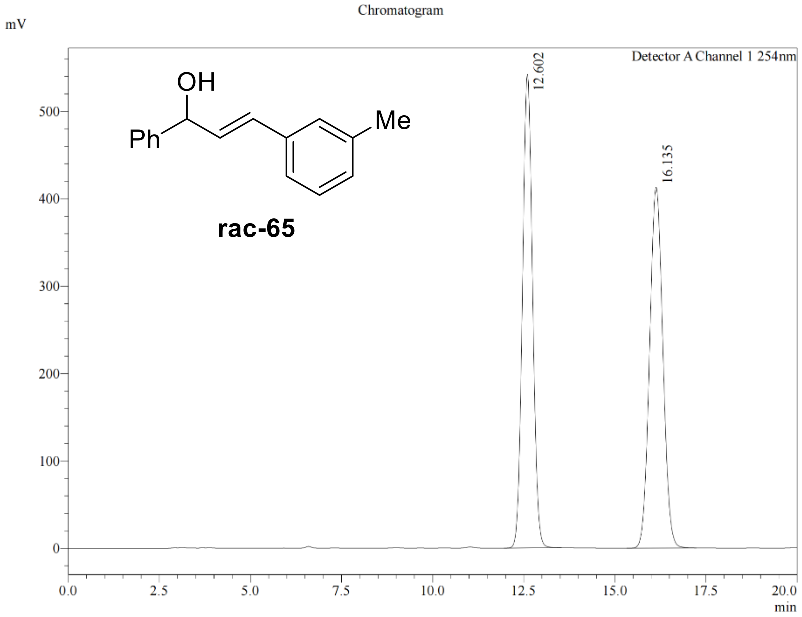

Peak Table

Detector A Channel 1 254nm

Peak#	Ret. Time	Area	Height	Area%	Height%
1	12.602	10430376	541809	49.876	56.767
2	16.135	10482075	412638	50.124	43.233
Total		20912451	954447	100.000	100.000

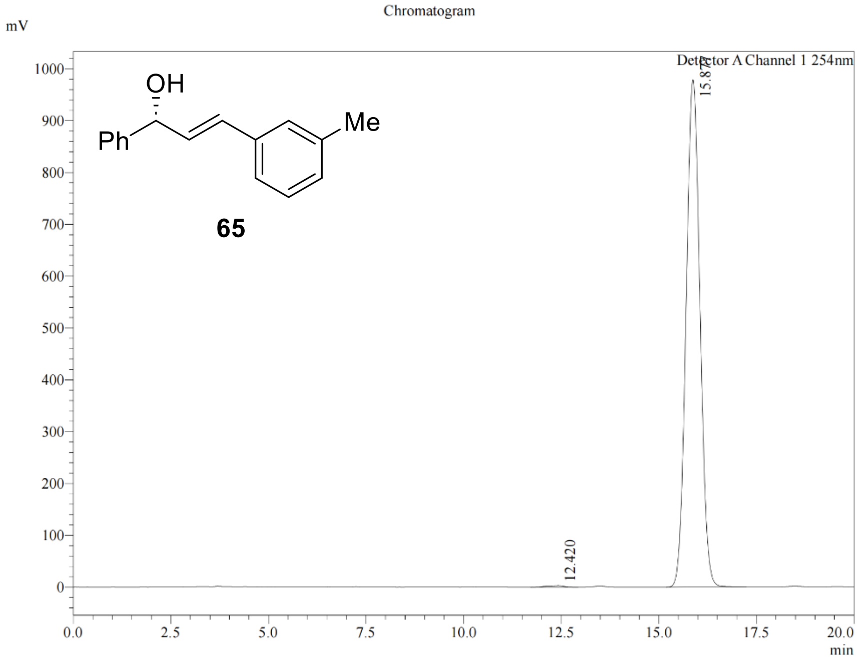

Peak Table

Detector A Channel 1 254nm

Peak#	Ret. Time	Area	Height	Area%	Height%
1	12.420	89741	3154	0.369	0.321
2	15.877	24219409	978922	99.631	99.679
Total		24309151	982076	100.000	100.000

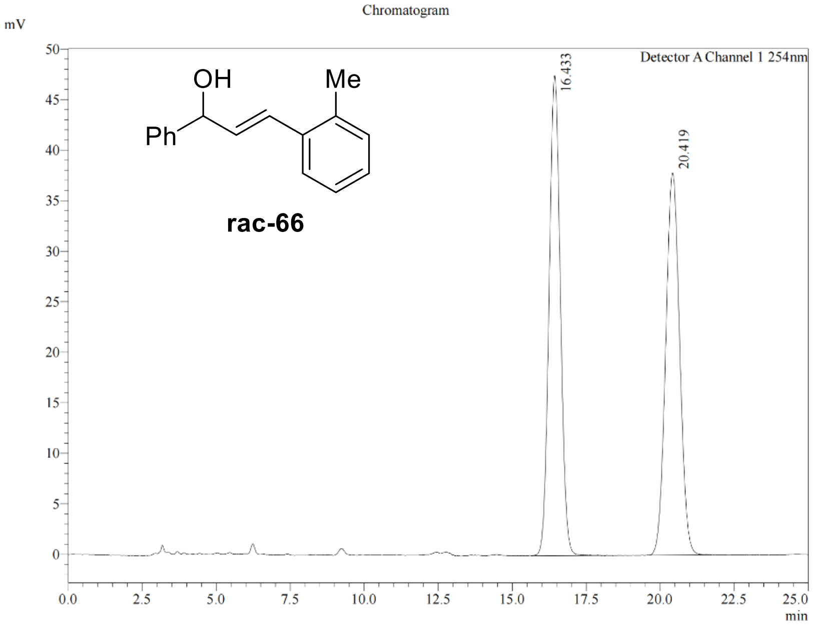

Peak Table

Detector A Channel 1 254nm

Peak#	Ret. Time	Area	Height	Area%	Height%
1	16.433	1254574	47520	50.289	55.689
2	20.419	1240163	37810	49.711	44.311
Total		2494737	85331	100.000	100.000

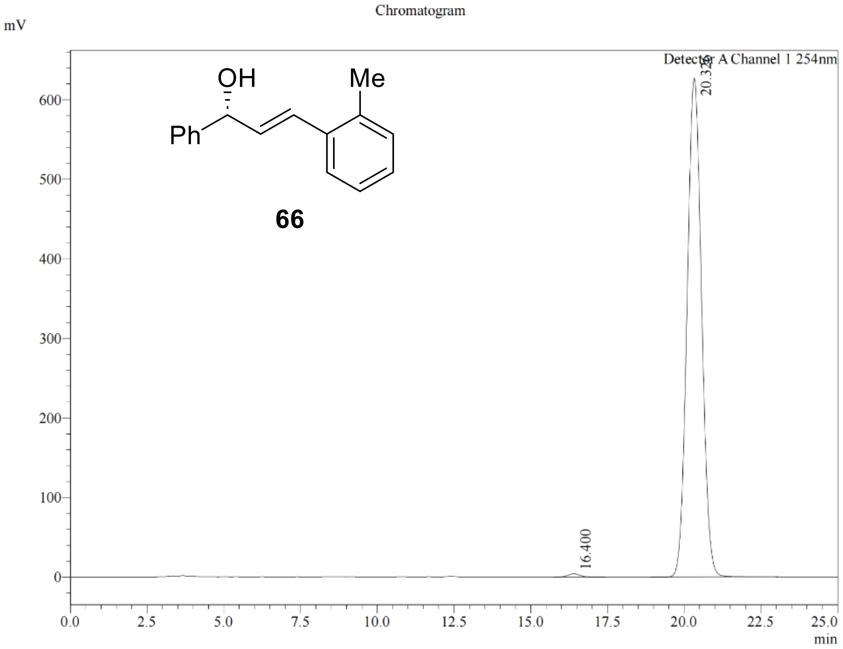

Peak Table

Detector A Channel 1 254nm

Peak#	Ret. Time	Area	Height	Area%	Height%
1	16.400	107957	3972	0.521	0.630
2	20.326	20620736	626962	99.479	99.370
Total		20728693	630934	100.000	100.000

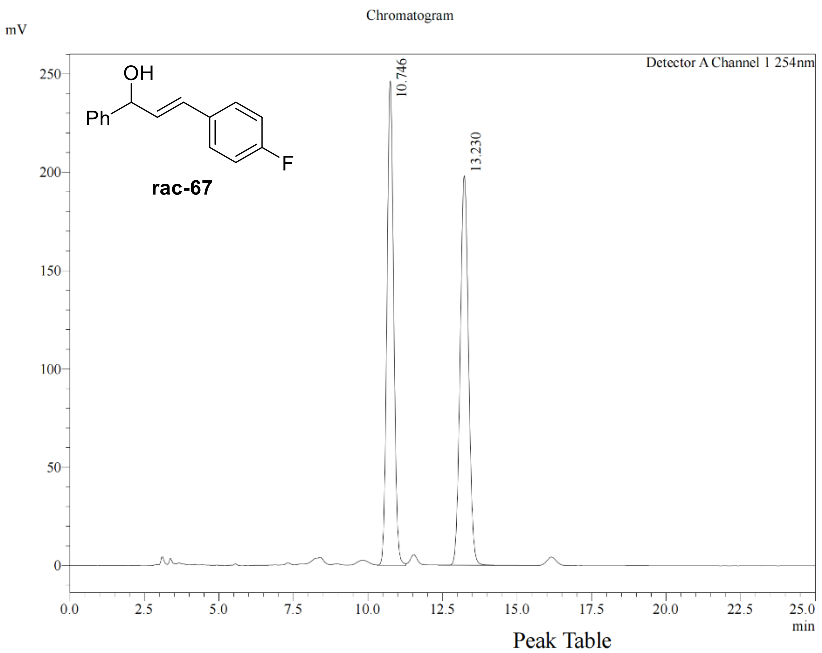

Peak Table

Detector A Channel 1 254nm

Peak#	Ret. Time	Area	Height	Area%	Height%
1	10.746	3878119	246416	50.162	55.457
2	13.230	3853108	197921	49.838	44.543
Total		7731227	444337	100.000	100.000

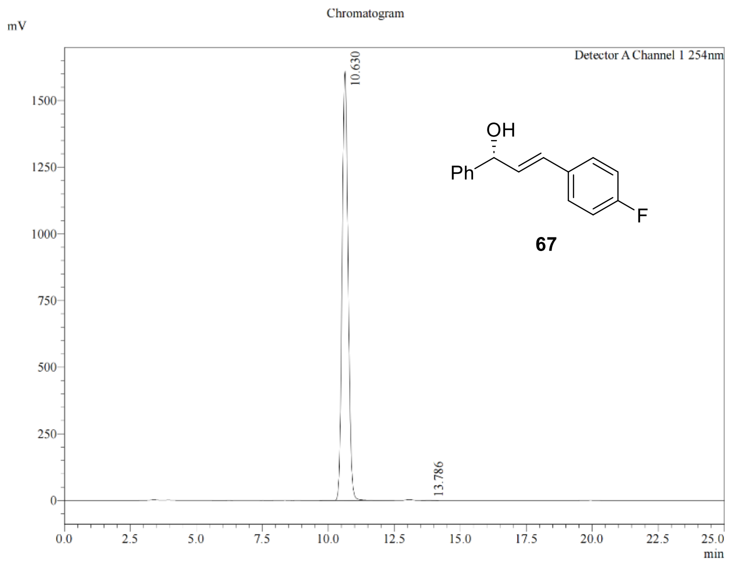

Peak Table

Detector A Channel 1 254nm

Peak#	Ret. Time	Area	Height	Area%	Height%
1	10.630	25377586	1608868	99.920	99.926
2	13.786	20264	1195	0.080	0.074
Total		25397851	1610063	100.000	100.000

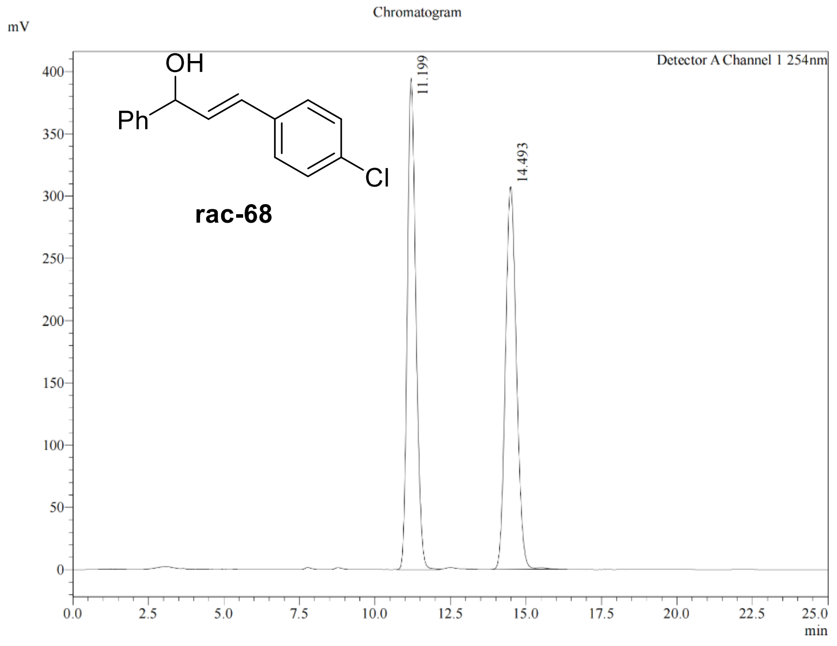

Peak Table

Detector A Channel 1 254nm

Peak#	Ret. Time	Area	Height	Area%	Height%
1	11.199	7783909	394165	49.960	56.175
2	14.493	7796464	307514	50.040	43.825
Total		15580373	701679	100.000	100.000

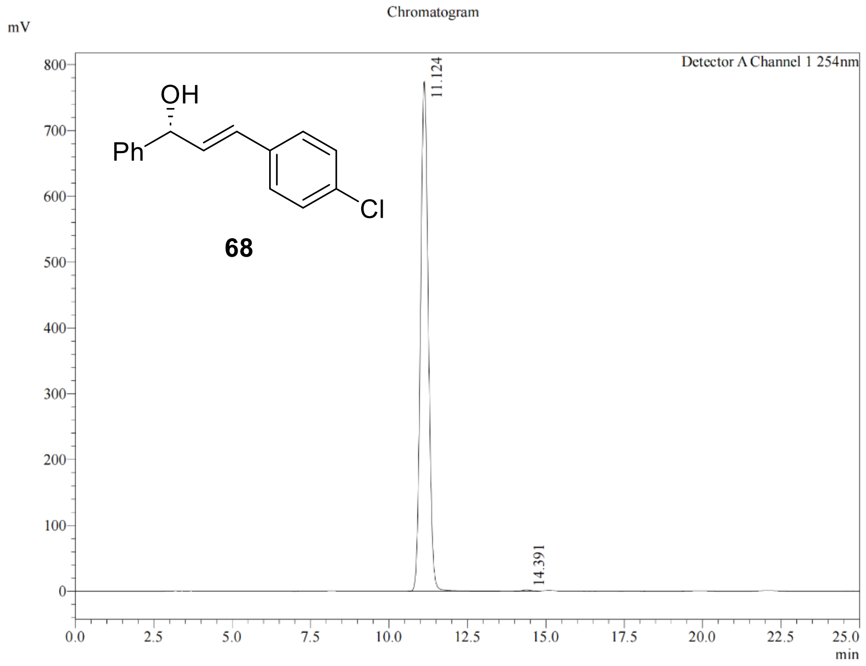

Peak Table

Detector A Channel 1 254nm

Peak#	Ret. Time	Area	Height	Area%	Height%
1	11.124	13422717	774668	99.731	99.767
2	14.391	36261	1810	0.269	0.233
Total		13458979	776478	100.000	100.000

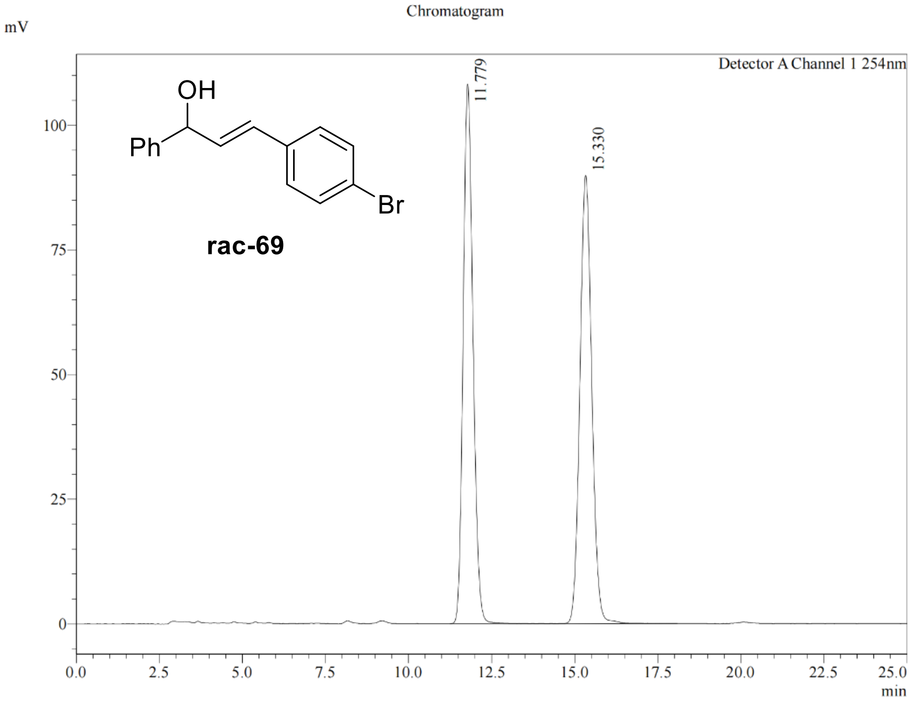

Peak Table

Detector A Channel 1 254nm

Peak#	Ret. Time	Area	Height	Area%	Height%
1	11.779	2221452	108200	49.864	54.613
2	15.330	2233588	89922	50.136	45.387
Total		4455041	198122	100.000	100.000

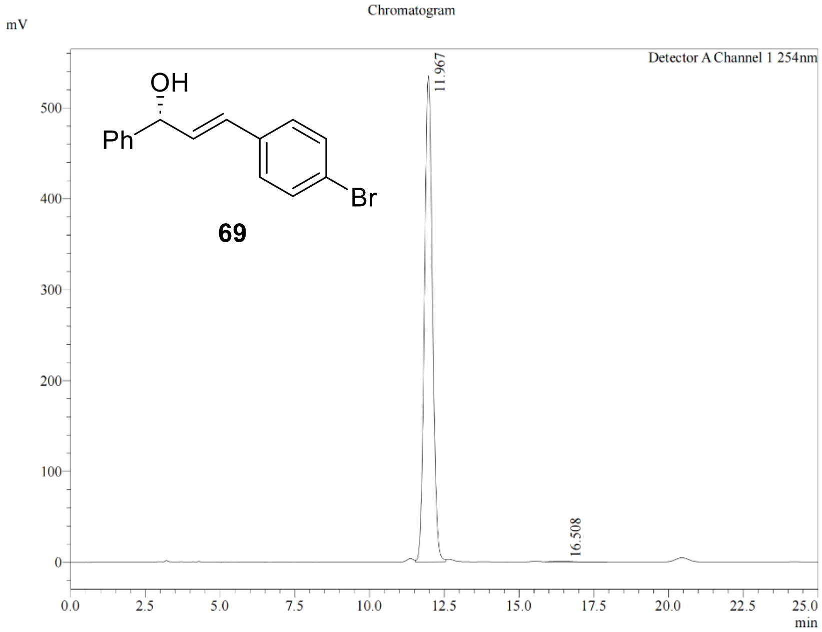

Peak Table

Detector A Channel 1 254nm

Peak#	Ret. Time	Area	Height	Area%	Height%
1	11.967	9948649	534379	99.643	99.855
2	16.508	35647	777	0.357	0.145
Total		9984296	535157	100.000	100.000

Chromatogram

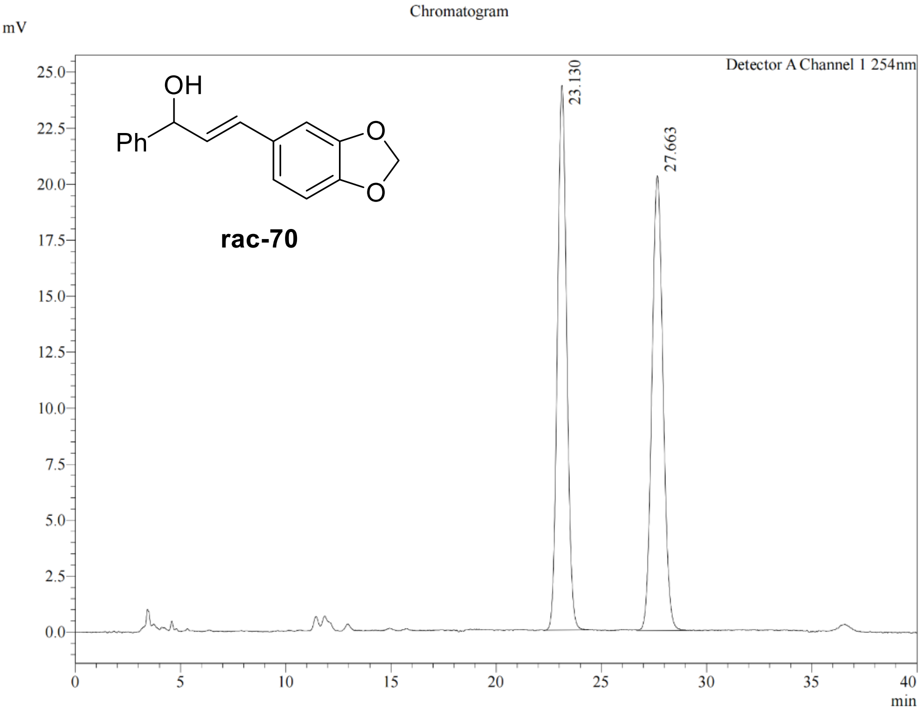

Peak Table

Detector A Channel 1 254nm

Peak#	Ret. Time	Area	Height	Area%	Height%
1	23.130	747168	24310	49.972	54.492
2	27.663	748019	20302	50.028	45.508
Total		1495187	44612	100.000	100.000

Chromatogram

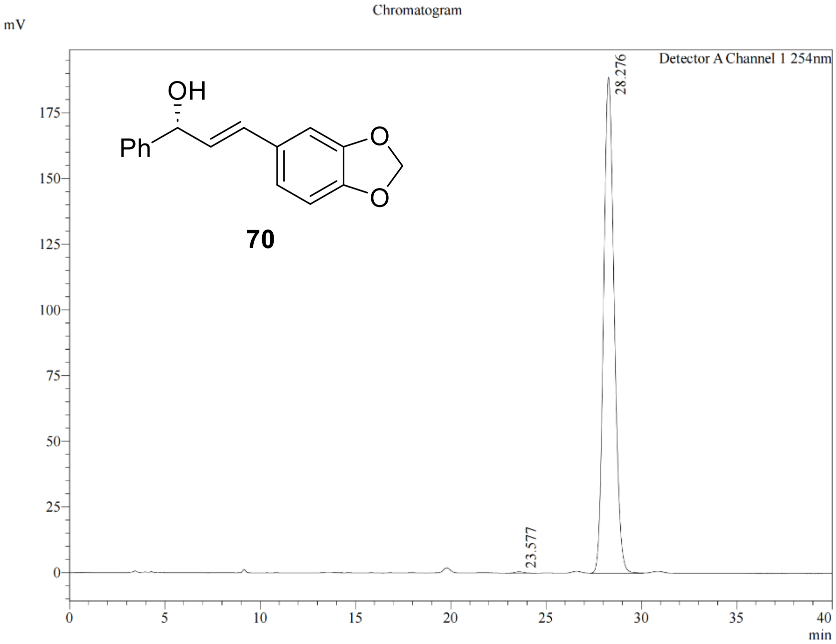

Peak Table

Detector A Channel 1 254nm

Peak#	Ret. Time	Area	Height	Area%	Height%
1	23.577	15056	502	0.206	0.266
2	28.276	7285806	188666	99.794	99.734
Total		7300863	189169	100.000	100.000

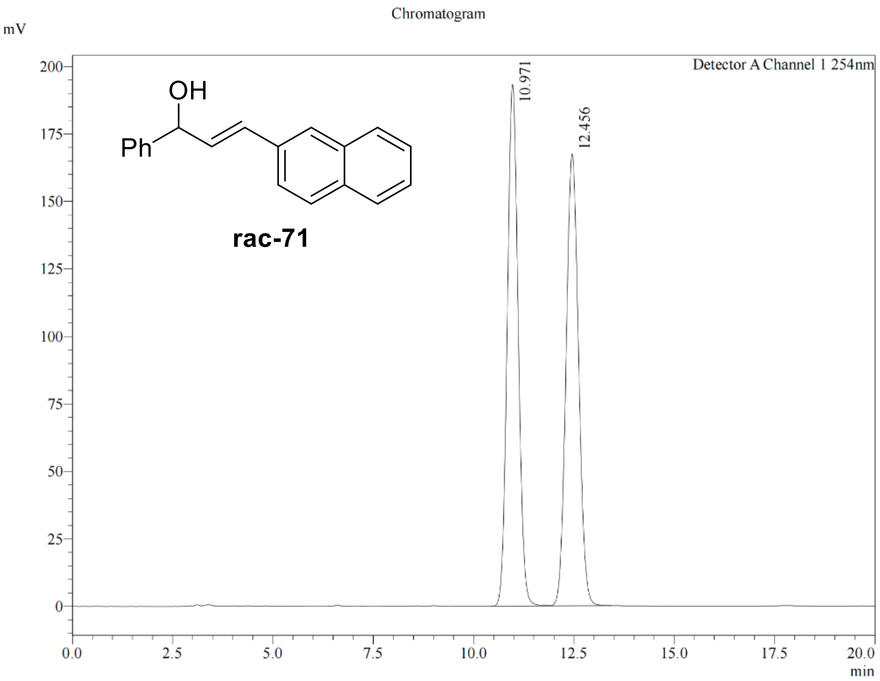

Peak Table

Detector A Channel 1 254nm

Peak#	Ret. Time	Area	Height	Area%	Height%
1	10.971	3653526	193201	50.193	53.588
2	12.456	3625485	167329	49.807	46.412
Total		7279011	360530	100.000	100.000

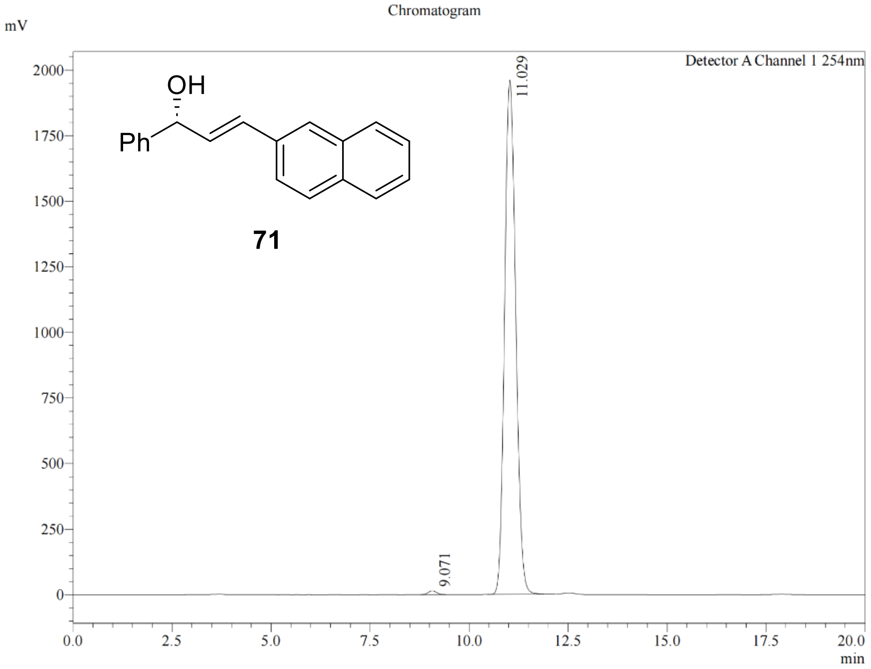

Peak Table

Detector A Channel 1 254nm

Peak#	Ret. Time	Area	Height	Area%	Height%
1	9.071	211795	14080	0.550	0.713
2	11.029	38329751	1959366	99.450	99.287
Total		38541546	1973446	100.000	100.000

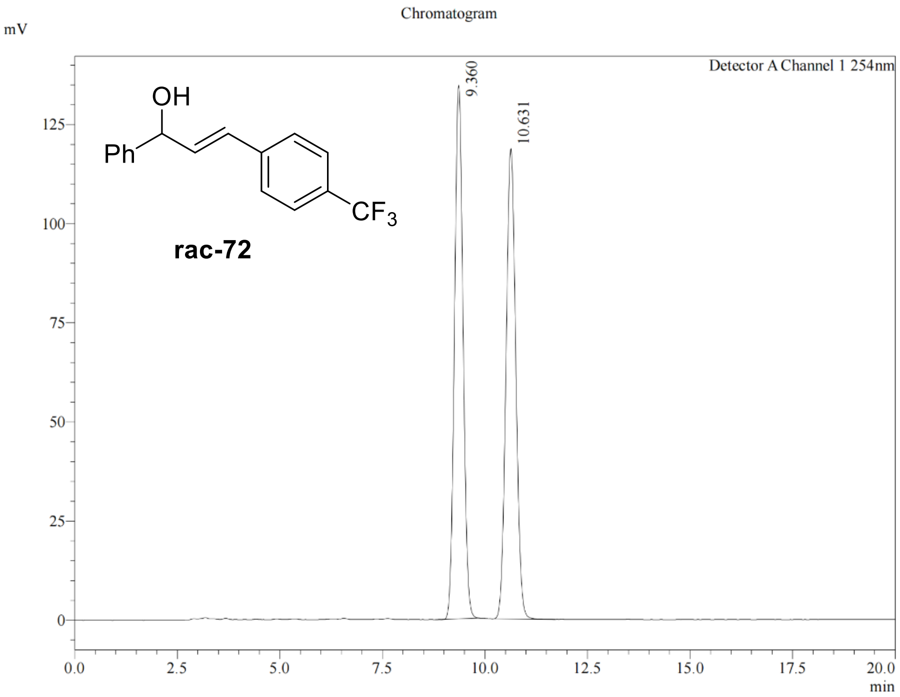

Peak Table

Detector A Channel 1 254nm

Peak#	Ret. Time	Area	Height	Area%	Height%
1	9.360	1956526	134386	49.929	53.131
2	10.631	1962061	118550	50.071	46.869
Total		3918587	252936	100.000	100.000

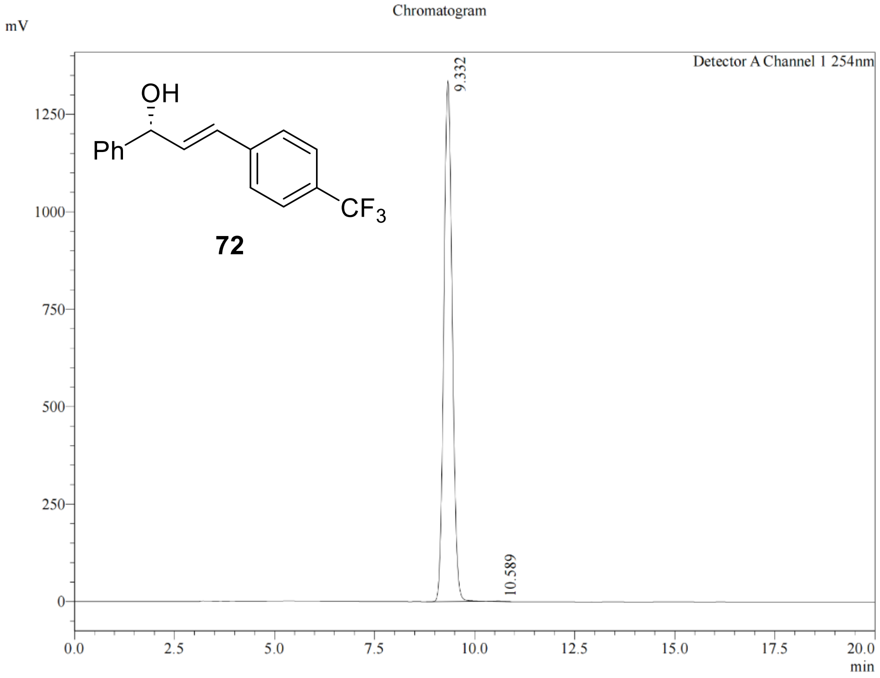

Peak Table

Detector A Channel 1 254nm

Peak#	Ret. Time	Area	Height	Area%	Height%
1	9.332	19556219	1334667	99.880	99.885
2	10.589	23489	1534	0.120	0.115
Total		19579708	1336201	100.000	100.000

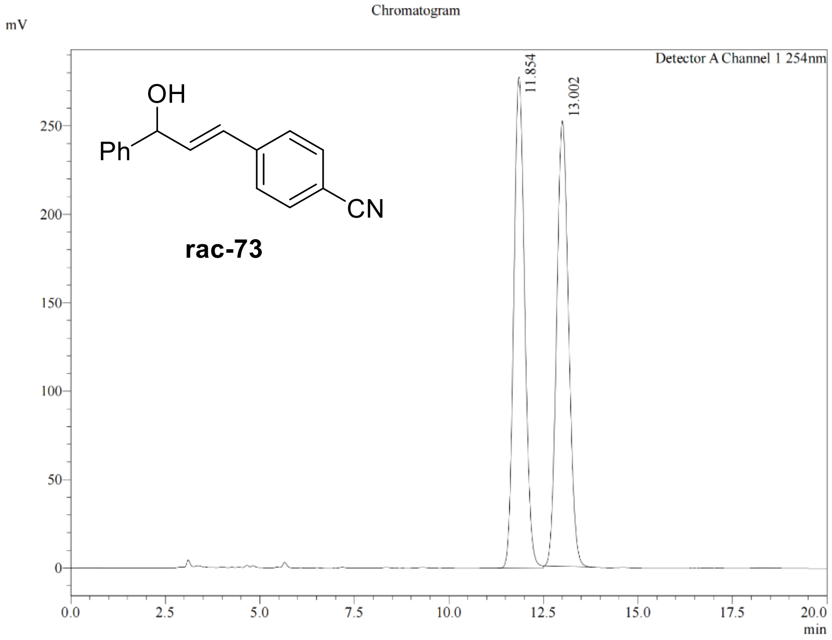

Peak Table

Detector A Channel 1 254nm

Peak#	Ret. Time	Area	Height	Area%	Height%
1	11.854	5590342	277741	50.237	52.439
2	13.002	5537668	251905	49.763	47.561
Total		11128011	529645	100.000	100.000

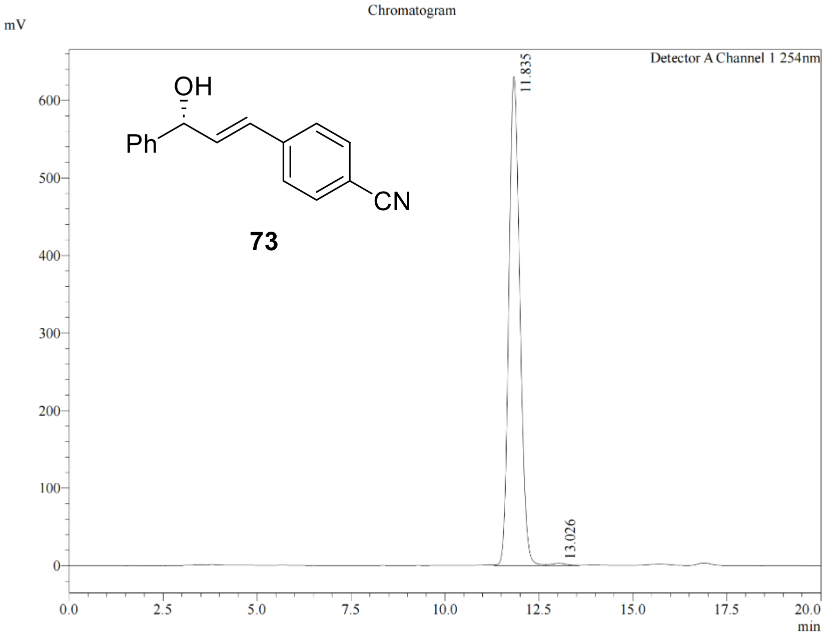

Peak Table

Detector A Channel 1 254nm

Peak#	Ret. Time	Area	Height	Area%	Height%
1	11.835	12862047	630485	99.287	99.518
2	13.026	92309	3053	0.713	0.482
Total		12954355	633538	100.000	100.000

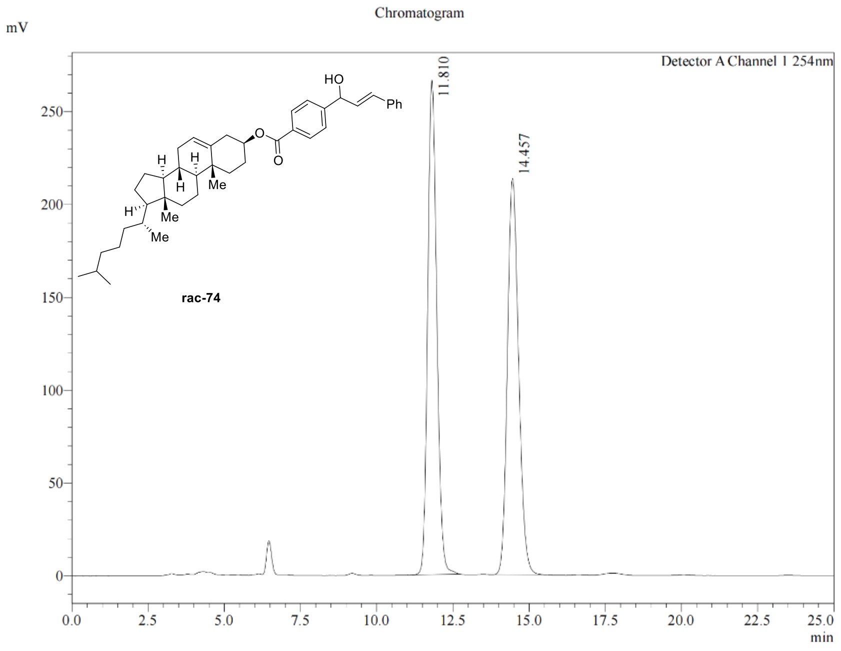

Peak Table

Detector A Channel 1 254nm

Peak#	Ret. Time	Area	Height	Area%	Height%
1	11.810	5515663	266282	50.291	55.506
2	14.457	5451927	213453	49.709	44.494
Total		10967590	479735	100.000	100.000

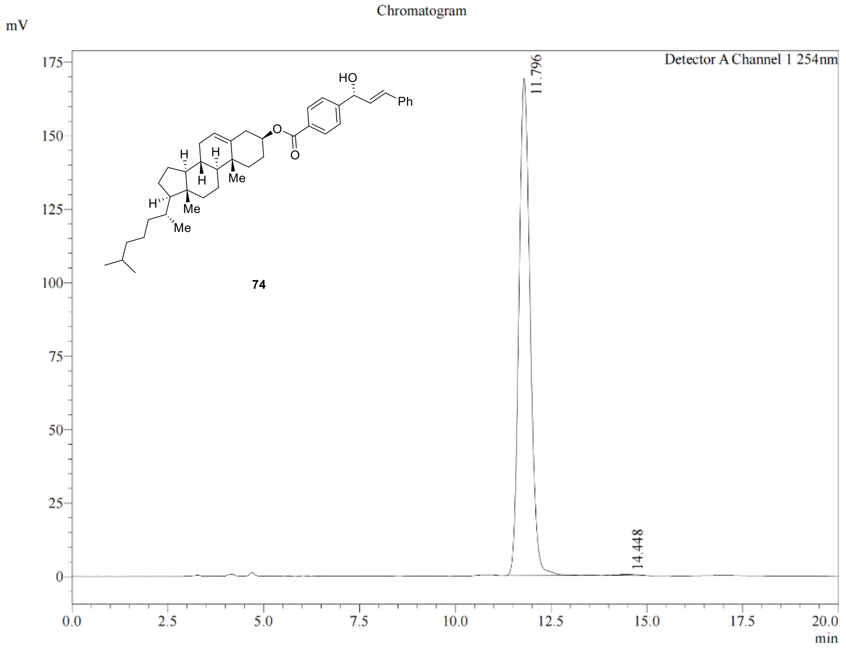

Peak Table

Detector A Channel 1 254nm

Peak#	Ret. Time	Area	Height	Area%	Height%
1	11.796	3445140	169211	99.639	99.754
2	14.448	12480	417	0.361	0.246
Total		3457620	169628	100.000	100.000

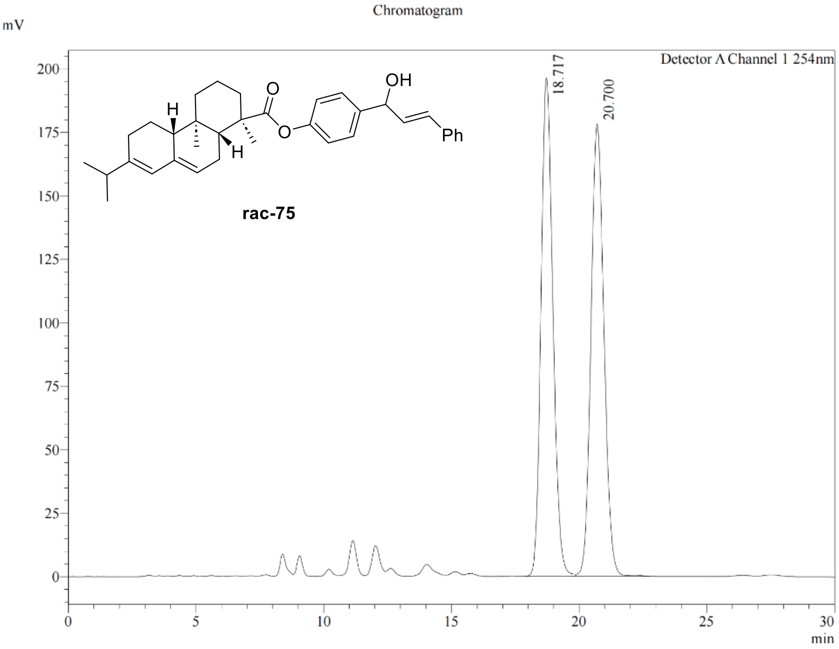

Peak Table

Detector A Channel 1 254nm

Peak#	Ret. Time	Area	Height	Area%	Height%
1	18.717	6352838	196277	50.182	52.422
2	20.700	6306670	178139	49.818	47.578
Total		12659508	374416	100.000	100.000

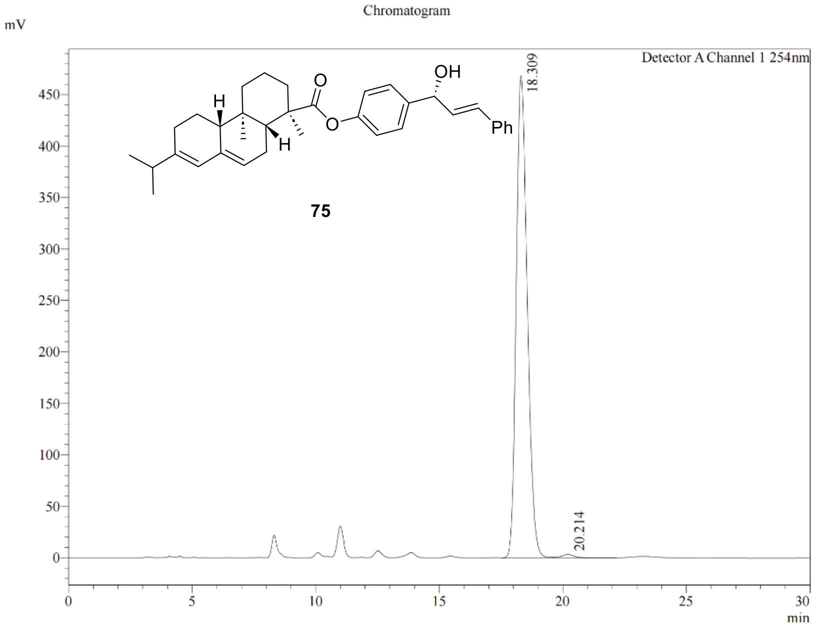

Peak Table

Detector A Channel 1 254nm

Peak#	Ret. Time	Area	Height	Area%	Height%
1	18.309	14502681	468291	99.087	99.295
2	20.214	133611	3324	0.913	0.705
Total		14636291	471615	100.000	100.000

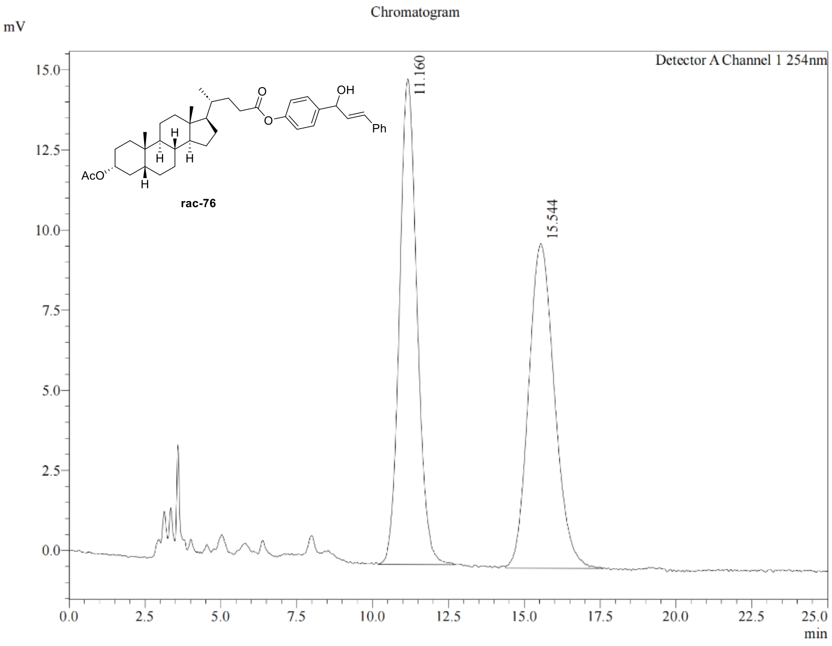

Peak Table

Detector A Channel 1 254nm

Peak#	Ret. Time	Area	Height	Area%	Height%
1	11.160	609916	15154	50.312	59.933
2	15.544	602359	10131	49.688	40.067
Total		1212276	25284	100.000	100.000

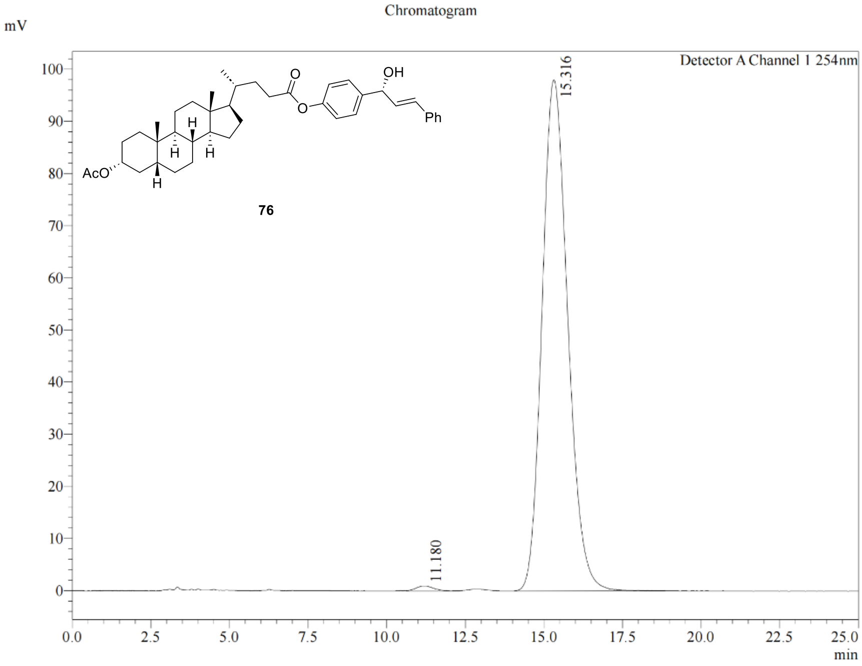

Peak Table

Detector A Channel 1 254nm

Peak#	Ret. Time	Area	Height	Area%	Height%
1	11.180	40744	993	0.727	1.002
2	15.316	5566907	98055	99.273	98.998
Total		5607650	99048	100.000	100.000

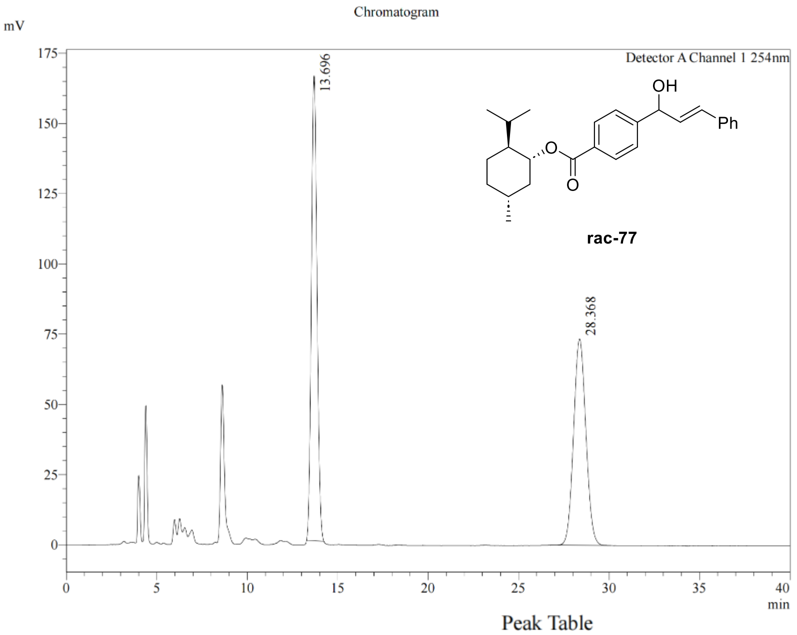

Peak Table

Detector A Channel 1 254nm

Peak#	Ret. Time	Area	Height	Area%	Height%
1	13.696	3592582	165483	50.817	69.251
2	28.368	3477030	73477	49.183	30.749
Total		7069612	238961	100.000	100.000

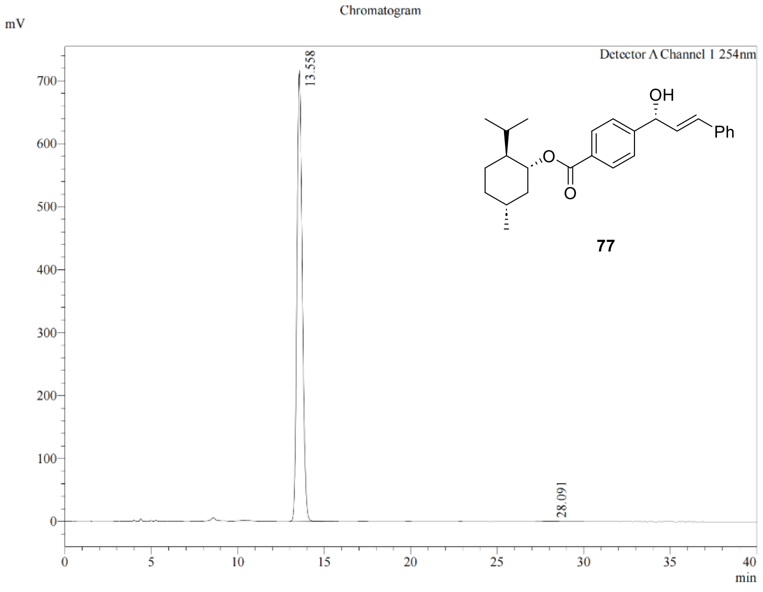

Peak Table

Detector A Channel 1 254nm

Peak#	Ret. Time	Area	Height	Area%	Height%
1	13.558	15472090	714798	99.718	99.877
2	28.091	43831	878	0.282	0.123
Total		15515922	715677	100.000	100.000

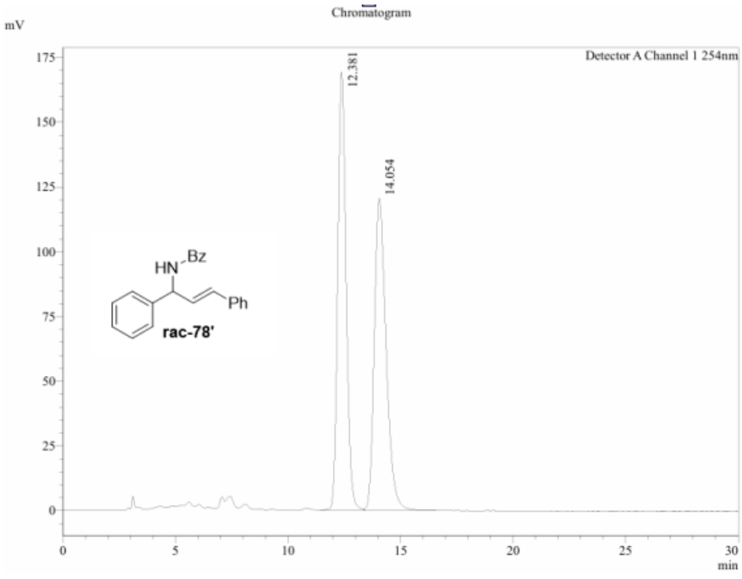

Peak Table

Detector A Channel 1 254nm

Peak#	Ret. Time	Area	Height	Area%	Height%
1	12.381	4384650	169273	49.885	58.411
2	14.054	4404820	120526	50.115	41.589
Total		8789470	289800	100.000	100.000

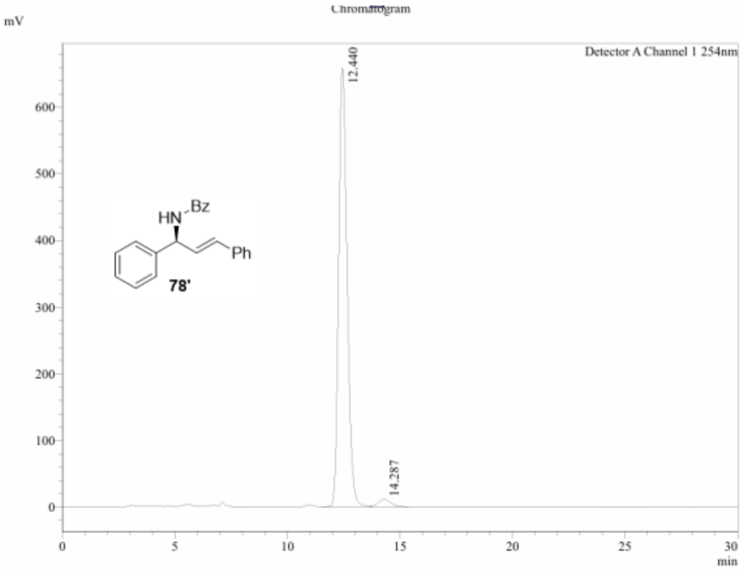

Peak Table

Detector A Channel 1 254nm

Peak#	Ret. Time	Area	Height	Area%	Height%
1	12.440	17312963	658827	97.539	98.350
2	14.287	436788	11051	2.461	1.650
Total		17749751	669879	100.000	100.000

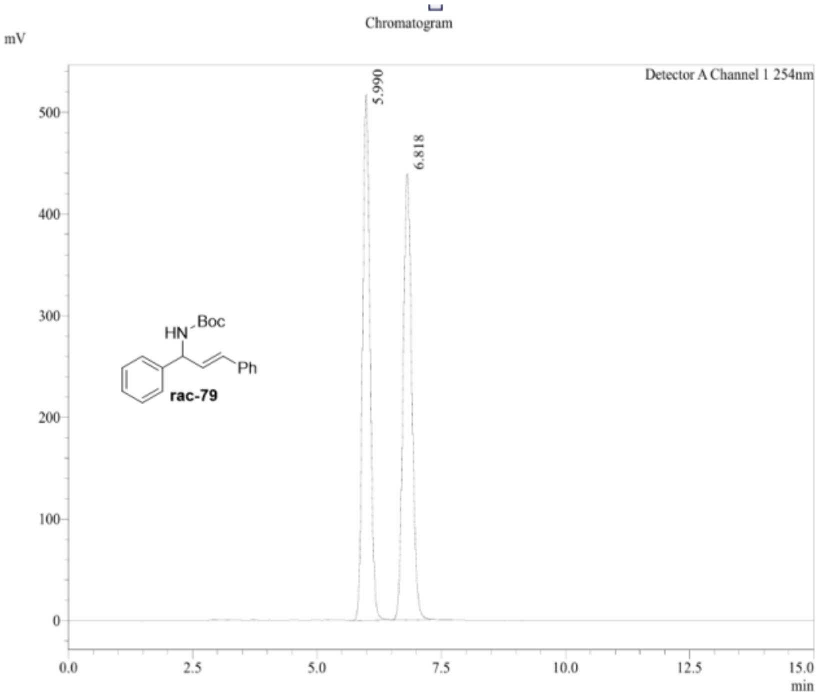

Peak Table

Detector A Channel 1 254nm

Peak#	Ret. Time	Area	Height	Area%	Height%
1	5.990	5355917	517092	50.038	54.115
2	6.818	5347755	438459	49.962	45.885
Total		10703672	955551	100.000	100.000

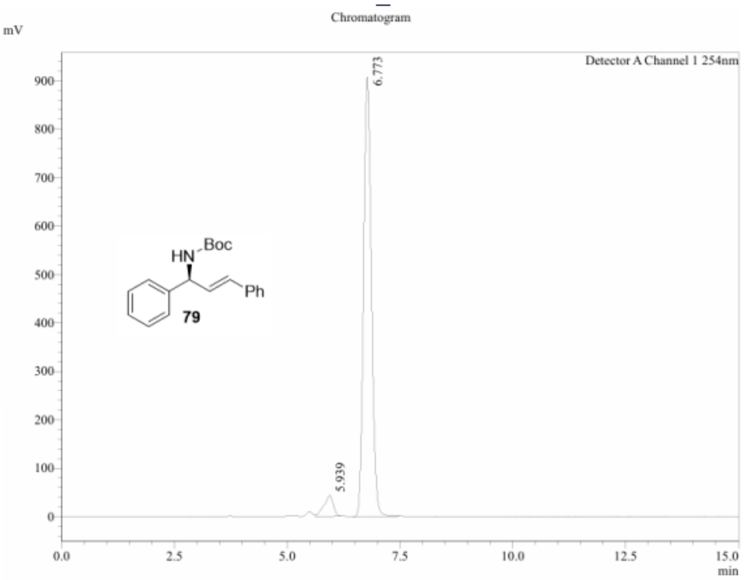

Peak Table

Detector A Channel 1 254nm

Peak#	Ret. Time	Area	Height	Area%	Height%
1	5.939	630776	43096	5.459	4.539
2	6.773	10923955	906467	94.541	95.461
Total		11554731	949563	100.000	100.000

Chromatogram

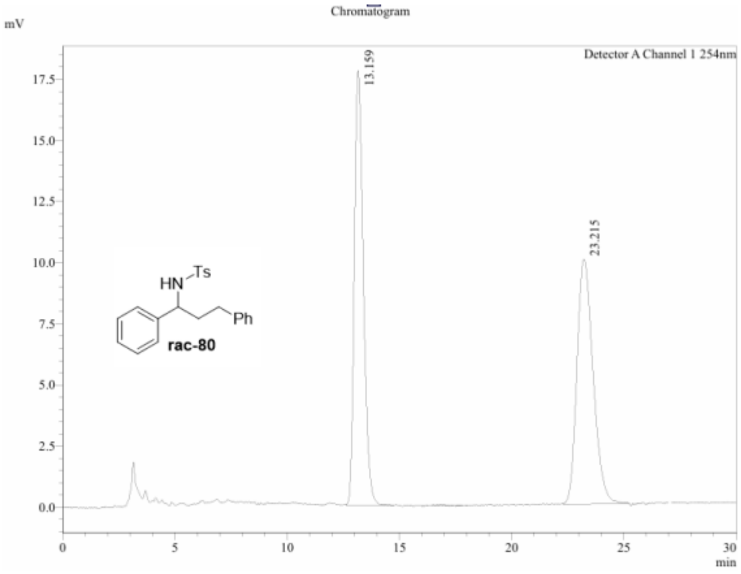

Peak Table

Detector A Channel 1 254nm

Peak#	Ret. Time	Area	Height	Area%	Height%
1	13.159	497876	17777	49.909	63.988
2	23.215	499699	10004	50.091	36.012
Total		997574	27781	100.000	100.000

Chromatogram

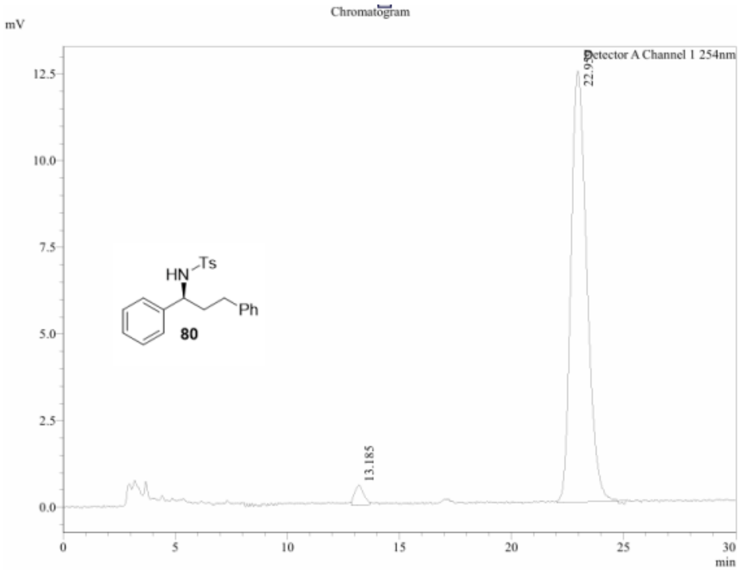

Peak Table

Detector A Channel 1 254nm

Peak#	Ret. Time	Area	Height	Area%	Height%
1	13.185	16040	573	2.562	4.404
2	22.959	610127	12434	97.438	95.596
Total		626167	13007	100.000	100.000

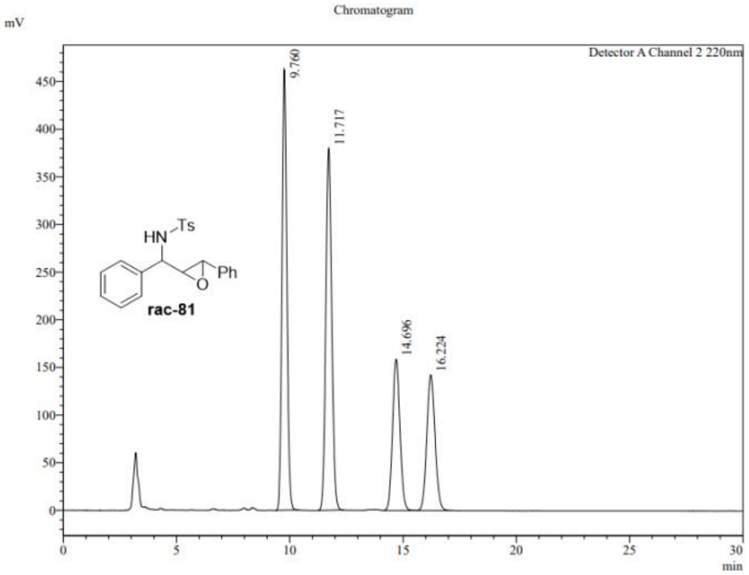

Peak Table

Detector A Channel 2 220nm

Peak#	Ret. Time	Area	Height	Area%	Height%
1	9.760	6862650	462182	32.766	40.430
2	11.717	6876718	379590	32.833	33.205
3	14.696	3600114	158887	17.189	13.899
4	16.224	3604812	142513	17.211	12.466
Total		20944295	1143172	100.000	100.000

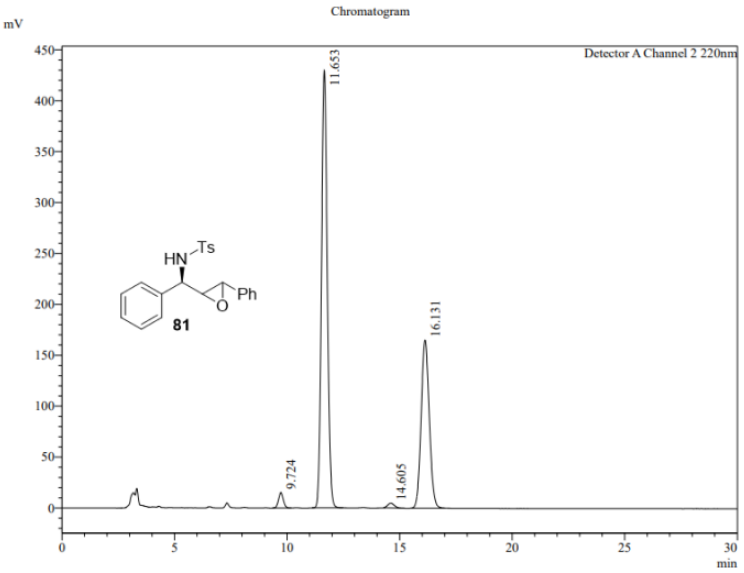

Peak Table

Detector A Channel 2 220nm

Peak#	Ret. Time	Area	Height	Area%	Height%
1	9.724	226639	15130	1.859	2.463
2	11.653	7727852	429440	63.386	69.916
3	14.605	93780	4576	0.769	0.745
4	16.131	4143380	165075	33.985	26.875
Total		12191651	614221	100.000	100.000

Chromatogram

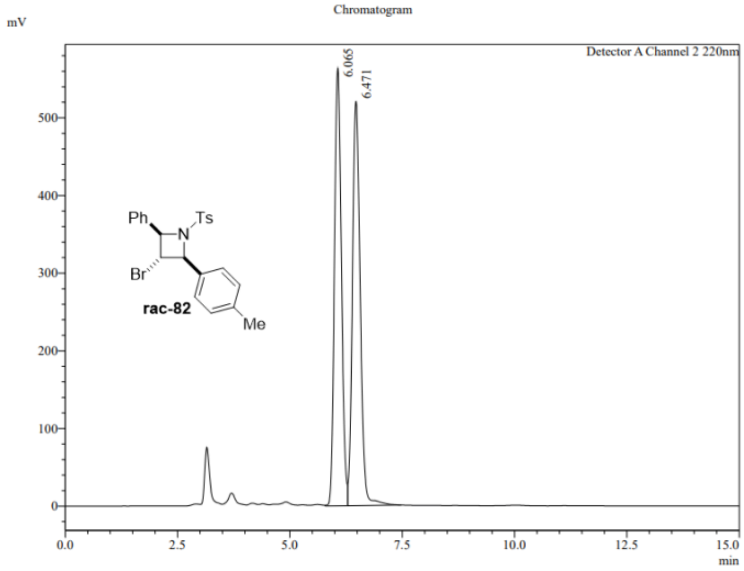

Peak Table

Detector A Channel 2 220nm

Peak#	Ret. Time	Area	Height	Area%	Height%
1	6.065	6099255	562921	49.268	51.973
2	6.471	6280532	520186	50.732	48.027
Total		12379787	1083107	100.000	100.000

Chromatogram

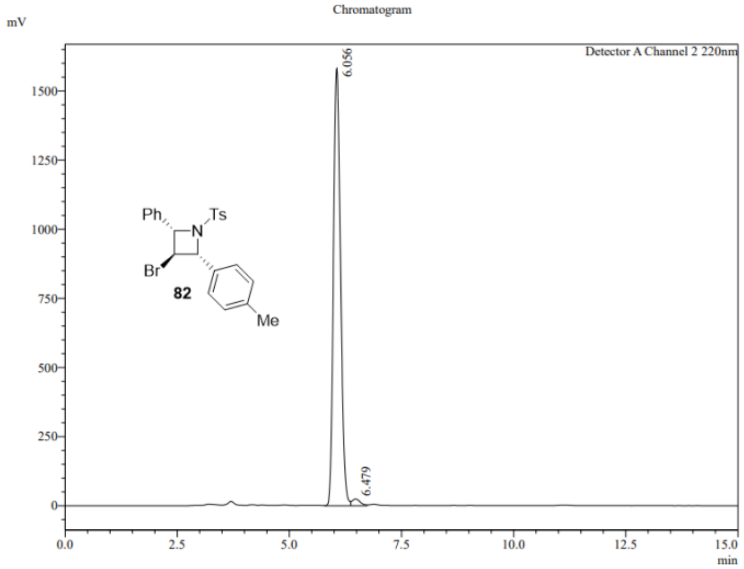

Peak Table

Detector A Channel 2 220nm

Peak#	Ret. Time	Area	Height	Area%	Height%
1	6.056	17366842	1580570	98.247	98.486
2	6.479	309822	24297	1.753	1.514
Total		17676665	1604867	100.000	100.000

Chromatogram

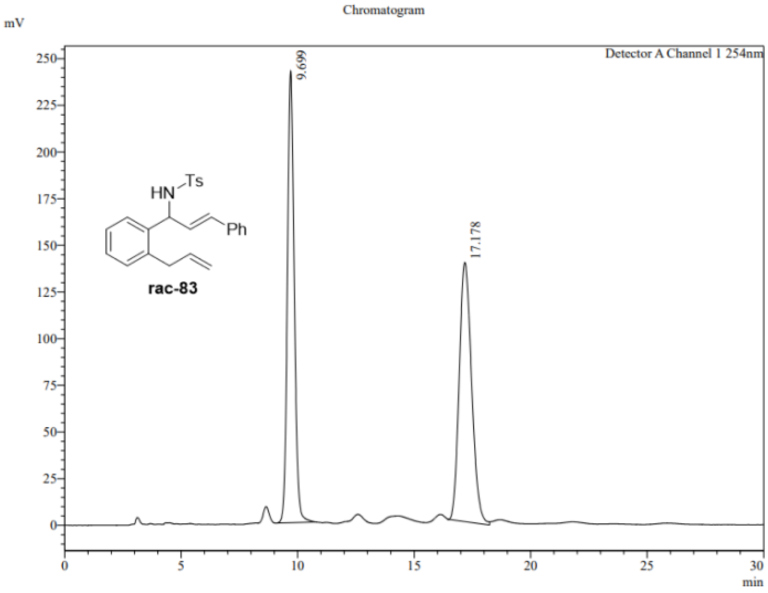

Peak Table

Detector A Channel 1 254nm

Peak#	Ret. Time	Area	Height	Area%	Height%
1	9.699	4947676	241808	49.137	63.527
2	17.178	5121501	138832	50.863	36.473
Total		10069177	380640	100.000	100.000

Chromatogram

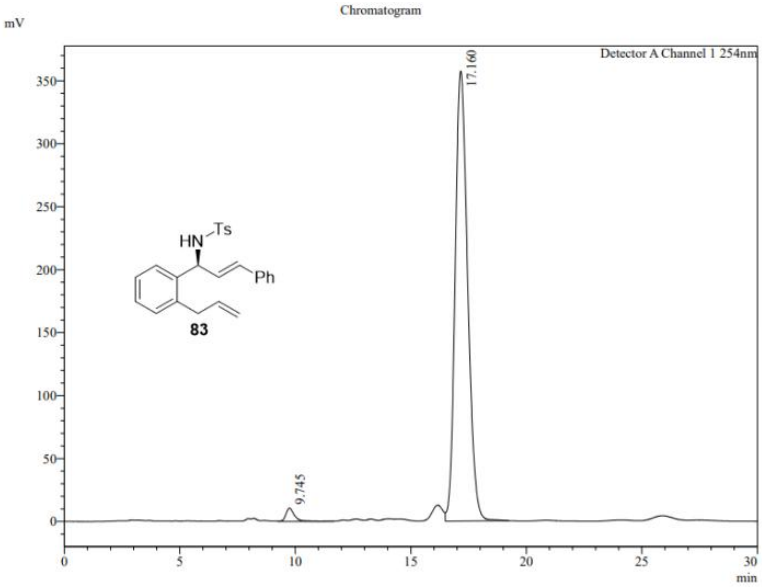

Peak Table

Detector A Channel 1 254nm

Peak#	Ret. Time	Area	Height	Area%	Height%
1	9.745	284532	10601	2.075	2.880
2	17.160	13429570	357435	97.925	97.120
Total		13714102	368036	100.000	100.000

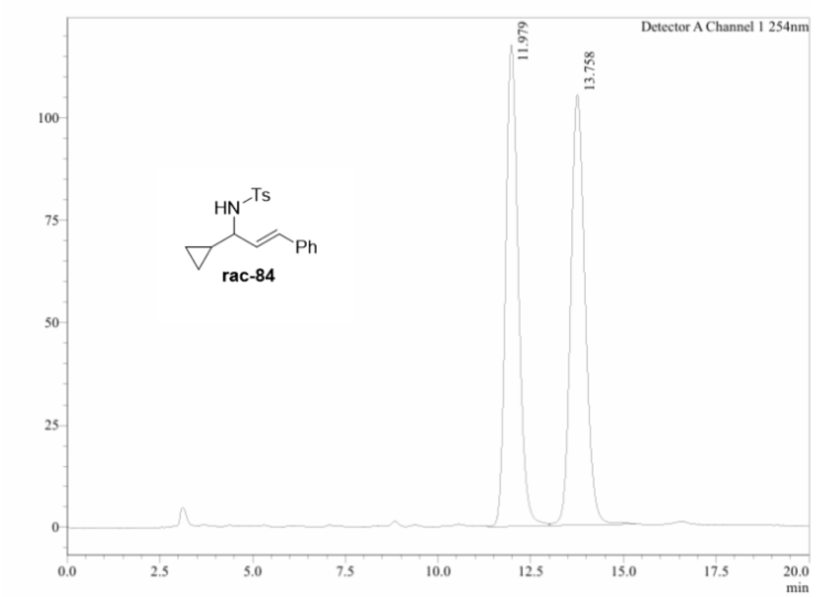

Peak Table

Detector A Channel 1 254nm

Peak#	Ret. Time	Area	Height	Area%	Height%
1	11.979	2793937	117583	49.635	52.800
2	13.758	2835006	105111	50.365	47.200
Total		5628943	222694	100.000	100.000

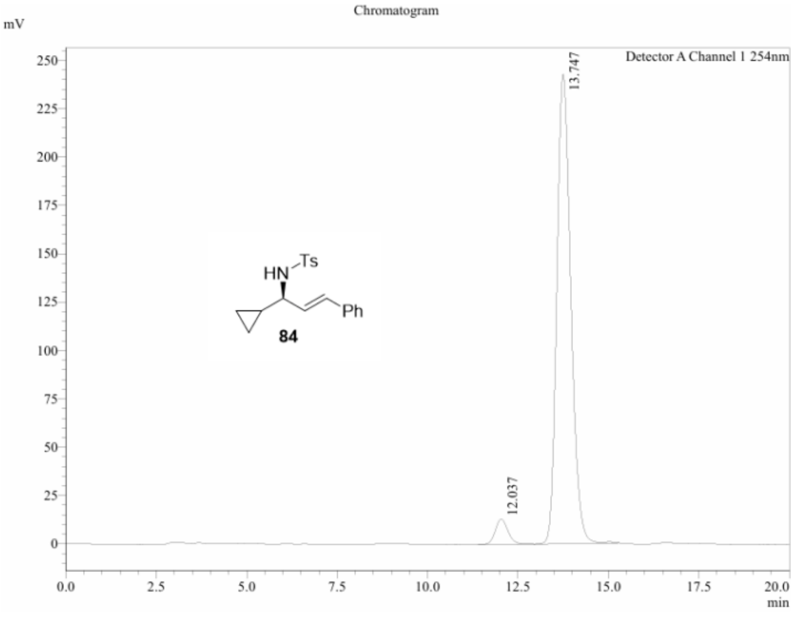

Peak Table

Detector A Channel 1 254nm

Peak#	Ret. Time	Area	Height	Area%	Height%
1	12.037	310623	12909	4.558	5.053
2	13.747	6504972	242580	95.442	94.947
Total		6815595	255489	100.000	100.000

14. Supplementary Reference

1. Morales, S.; Guijarro, F. G.; Ruano, J. L. G.; Cid, M. B. A General Aminocatalytic Method for the Synthesis of Aldimines. *J. Am. Chem. Soc.* **136**, 1082-1089 (2014).
2. Trost, B. M.; Silverman, S. M. Enantioselective Construction of Pyrrolidines by Palladium-Catalyzed Asymmetric [3 + 2] Cycloaddition of Trimethylenemethane with Imines. *J. Am. Chem. Soc.* **134**, 4941-4954 (2012).
3. Cui, Z.; Yu, H.-J.; Yang, R.-F.; Gao, W.-Y.; Feng, C.-G.; Lin, G.-Q. Highly Enantioselective Arylation of *N*-Tosylalkylaldimines Catalyzed by Rhodium-Diene Complexes. *J. Am. Chem. Soc.* **133**, 12394-12397 (2011).
4. Deng, Y.; Wei, X.; Wang, X.; Sun, Y.; Noël, T. Iron-Catalyzed Cross-Coupling of Alkynyl and Styrenyl Chlorides with Alkyl Grignard Reagents in Batch and Flow. *Chem. Eur. J.* **25**, 14532-14535 (2019).
5. Nitelet, A.; Evano, G. A General Copper-Catalyzed Vinylic Halogen Exchange Reaction. *Org. Lett.* **18**, 1904-1907 (2016).
6. Behlen, M. J.; Uyeda, C. C_2-Symmetric Dinickel Catalysts for Enantioselective [4 + 1] Cycloadditions. *J. Am. Chem. Soc.* **142**, 17294-17300 (2020).
7. Tak, R. K.; Noda, H.; Shibasaki, M. Ligand-Enabled, Copper-Catalyzed Electrophilic Amination for the Asymmetric Synthesis of *β*-Amino Acids. *Org. Lett.* **23**, 8617-8621 (2021).
8. Zhang, Y.; Sigman, M. S. Palladium(II)-Catalyzed Enantioselective Aerobic Dialkoxylation of 2-Propenyl Phenols: A Pronounced Effect of Copper Additives on Enantioselectivity. *J. Am. Chem. Soc.* **129**, 3076-3077 (2007).
9. Sun, S.-Z.; Cai, Y.-M.; Zhang, D.-L.; Wang, J.-B.; Yao, H.-Q.; Rui, X.-Y.; Martin, R.; Shang, M. Enantioselective Deaminative Alkylation of Amino Acid Derivatives with Unactivated Olefins. *J. Am. Chem. Soc.* **144**, 1130-1137 (2022).
10. Xiao, L.-J.; Zhao, C.-Y.; Cheng, L.; Feng, B.-Y.; Feng, W.-M.; Xie, J.-H.; Xu, X.-F.; Zhou, Q.-L. Nickel(0)-Catalyzed Hydroalkenylation of Imines with Styrene and Its Derivatives. *Angew. Chem. Int. Ed.* **57**, 3396-3400 (2018).
11. Wang, Y.; Xu, Y.-N.; Fang, G.-S.; Kang, H.-J.; Gu, Y.-H.; Tian, S.-K.; Kinetic resolution of primary allylic amines via palladium-catalyzed asymmetric allylic alkylation of malononitriles. *Org. Biomol. Chem.* **13**, 5367–5371 (2015).
12. Li, L.; Liu, Y.-C.; Shi, H. Nickel-Catalyzed Enantioselective *α*-Alkenylation of *N*-Sulfonyl Amines: Modular Access to Chiral *α*-Branched Amines. *J. Am. Chem. Soc.* **143**, 4154-4161 (2021).
13. Xiao, W.-G.; Xuan, B.; Xiao, L.-J.; Zhou, Q.-L. Practical synthesis of allylic amines via nickel-catalysed multicomponent coupling of alkenes, aldehydes, and amides. *Chem. Sci.* **14**, 8644-8650 (2023).
14. Gopula, B.; Chiang, C.-W.; Lee, W.-Z.; Kuo, T.-S.; Wu, P.-Y. ; Henschke, J. P.; Wu, H.-L. Highly Enantioselective Rh-Catalyzed Alkenylation of Imines: Synthesis of Chiral Allylic Amines via Asymmetric Addition of Potassium Alkenyltrifluoroborates to *N*-Tosyl Imines. *Org. Lett.* **16**, 632-635 (2014).
15. Ghorai, M. K.; Kumar, A.; Das, K. Lewis Acid-Mediated Unprecedented Ring-Opening Rearrangement of 2-Aryl-*N*-tosylazetidines to Enantiopure (*E*)-Allylamines. *Org. Lett.* **9**, 5441-5444 (2007).

16. Chen, F.-L.; Zhang, Y.; Yu, L.; Zhu, S.-L. Enantioselective NiH/Pmrox-Catalyzed 1,2-Reduction of *α*, *β*-Unsaturated Ketones. *Angew. Chem. Int. Ed.* **56**, 2022-2025 (2017).

17. Gaussian 09, Revision C.09, Frisch, M. J., Trucks, G. W., Schlegel, H. B., Scuseria, G. E., Robb, M. A., Cheeseman, J. R., Scalmani, G., Barone, V., Petersson, G. A., Nakatsuji, H., Li, X., Caricato, M., Marenich, A. V., Bloino, J., Janesko, B. G., Gomperts, R., Mennucci, B., Hratchian, H. P., Ortiz, J. V., Izmaylov, A. F., Sonnenberg, J. L., Williams-Young, D., Ding, F., Lipparini, F., Egidi, F., Goings, J., Peng, B., Petrone, A., Henderson, T., Ranasinghe, D., Zakrzewski, V. G., Gao, J., Rega, N., Zheng, G., Liang, W., Hada, M., Ehara, M., Toyota, K., Fukuda, R., Hasegawa, J., Ishida, M., Nakajima, T., Honda, Y., Kitao, O., Nakai, H., Vreven, T., Throssell, K., Montgomery, J. A. Jr., Peralta, J. E., Ogliaro, F., Bearpark, M. J., Heyd, J. J., Brothers, E. N., Kudin, K. N., Staroverov, V. N., Keith, T. A., Kobayashi, R., Normand, J., Raghavachari, K., Rendell, A. P., Burant, J. C., Iyengar, S. S., Tomasi, J., Cossi, M., Millam, J. M., Klene, M., Adamo, C., Cammi, R., Ochterski, J. W., Martin, R. L., Morokuma, K., Farkas, O., Foresman, J. B., Fox, D. J. *Gaussian, Inc.*, *Wallingford CT*, **2013**.

18. Zhao, Y. & Truhlar, D. G. A new local density functional for main-group thermochemistry, transition metal bonding, thermochemical kinetics, and noncovalent interactions. *J. Chem. Phys.* **125**, 194101 (2006).

19. Weigend, F. & Ahlrichs, R. Balanced basis sets of split valence, triple zeta valence and quadruple zeta valence quality for H to Rn: Design and assessment of accuracy. *Phys. Chem. Chem. Phys.* **7**, 3297-3305 (2005).

20. Weigend, F. Accurate Coulomb-fitting basis sets for H to Rn. *Phys. Chem. Chem. Phys.* **8**, 1057-1065 (2006).

21. (a) Hay, P. J.; Wadt, W. R. Ab initio effective core potentials for molecular calculations. Potentials for the transition metal atoms Sc to Hg. *J. Chem. Phys.* **82**, 270-283 (1985). (b) Wadt, W. R.; Hay, P. J. Ab initio effective core potentials for molecular calculations. Potentials for main group elements Na to Bi. *J. Chem. Phys.* **82**, 284-298 (1985).

22. Grimme, S., Antony, J., Ehrlich, S. & Krieg, H. A consistent and accurate ab initio parametrization of density functional dispersion correction (DFT-D) for the 94 elements H-Pu. *J. Chem. Phys.* **132**, 154104 (2010).

23. C. Y. Legault, CYLview, 1.0b, *Université de Sherbrooke*, 2009. (http://www.cylview.org)

24. Lu, T. & Chen, F. Multiwfn: A multifunctional wavefunction analyzer. *J. Comput. Chem.* **33**, 580-592 (2012).

25. Humphrey, W., Dalke, A. & Schulten, K. VMD: Visual molecular dynamics. *J. Mol. Graphics* **14**, 33-38 (1996).

26. Chen, P.-P. et al. How Solvents Control the Stereospecificity of Ni-Catalyzed Miyaura Borylation of Allylic Pivalates. *ACS Catal.* **9**, 9589-9598 (2019).

27. (a) Kwon, D. H., Proctor, M., Mendoza, S., Uyeda, C., Ess, D. H. Catalytic Dinuclear Nickel Spin Crossover Mechanism and Selectivity for Alkyne Cyclotrimerization. *ACS Catal.* **7**, 4796-4804, (2017). (b) Steiman, T. J., Kalb, A. E., Coombs, J. C. Kirkland, J. K., Torres, H., Ess, D. H., Uyeda, C. Dinickel-Catalyzed Vinylidene-Alkene Cyclization Reactions. *ACS Catal.* **11**, 14408-1441 (2021).
