# Supplementary material for: Dinickel-catalyzed enantioselective reductive addition of imines with vinyl halides
Source: Nat Commun. 2025 Oct 6;16:8871. doi: 10.1038/s41467-025-63940-y (PMC12501250; doi:10.1038/s41467-025-63940-y)
Supplement: Supplementary file 2 — Description of Additional Supplementary Files [file 41467_2025_63940_MOESM2_ESM.docx]

Description of Additional Supplementary Files

**File Name**: Supplementary Data 1

**Description**: The Cartesian coordinates of optimized geometries including starting materials, transition states, intermediates, and products associated with the DFT calculations are included in this file.
